# Supplementary material for: Unsymmetrical polysulfidation via designed bilateral disulfurating reagents
Source: Nat Commun. 2020 Aug 20;11:4170. doi: 10.1038/s41467-020-18029-z (PMC7441163; doi:10.1038/s41467-020-18029-z)
Supplement: Supplementary file 1 — Supplementary Information [file 41467_2020_18029_MOESM1_ESM.pdf]

SUPPLEMENTARY INFORMATION

Unsymmetrical Polysulfidation via Designed Bilateral Disulfurating Reagents

Xue et al.

Supplementary Methods

Reagents

All experiments were conducted under air atmosphere unless otherwise noted. Anhydrous CH_2ClCH_2Cl and CH_2Cl_2 was prepared by first distillation over P_2O_5 and then from CaH_2. 1,4-dioxane, toluene and THF were prepared by distillation over sodium-benzophenone ketyl prior to use. Sulfur monochloride (S_2Cl_2) was distilled according to procedures adapted from Fieser and Fieser (S_2Cl_2:sulfur:charcoal = 100:4:1).[1] Et_3N was dried with KOH. Other solvents were undried solvents.

Instruments

^1H, ^{19}F and ^{13}C NMR spectra were collected on 300 MHz, 400 MHz or 500 MHz NMR spectrometers (Bruker AVANCE) using $CDCl_3$, DMSO-d_6, Acetone-d_6, CD_3CN and CD_3OD. Chemical shifts are reported in parts per million (ppm). Chemical shifts for protons are reported in parts per million downfield and are referenced to residual protium in the NMR solvent ($CHCl_3$ = δ 7.26, DMSO = δ 2.50, Acetone = δ 2.05, CH_3CN = δ 1.94, CH_3OH = δ 3.31). Chemical shifts for carbon are reported in parts per million downfield and are referenced to the carbon resonances of the solvent ($CDCl_3$ = δ 77.0, DMSO-d_6 = δ 39.5, Acetone-d_6 = δ 29.8, 206.2, CD_3CN = δ 1.3, 118.2, CD_3OD = δ 49.0). Data are represented as follows: chemical shift, multiplicity (br = broad, s = singlet, d = doublet, t = triplet, q = quartet, m = multiplet), coupling constants in Hertz (Hz), integration. Unless otherwise noted, commercially available reagents purchased from Adamas-beta, TCI, or Energy Chemical were used as received. Mass spectra were in general recorded on a Shimadzu GCMS-QP2010 Ultra and a HP 5989A mass selective detector. Column chromatography was performed with silica gel (300-400 mesh ASTM). Infrared spectrum was recorded on IRtracer-100 Series FT-IR Spectrometers.

Synthesis and spectra data of disulfurating reagents

1,2-Bis(benzyloxy)disulfane (**1a**)

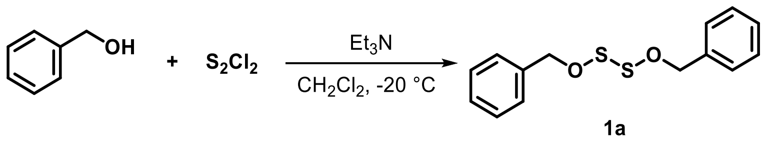

1,2-Bis(benzyloxy)disulfane (**1a**) was prepared according to literature procedures[2]: A solution of $PhCH_2OH$ (20 mmol) and NEt_3 (2.8 mL, 20 mmol) in 20 mL of CH_2Cl_2 was allowed to stir under

N_2 at -20 °C. A solution of S_2Cl_2 (0.8 mL, 10 mmol) in 20 mL of CH_2Cl_2 was added dropwise over 5 min. The reaction mixture was allowed to stir for a further 30 min at -20 °C. The reaction mixture was quenched with 20 mL of saturated $NaHCO_3$ and allowed to warm to room temperature. The organic phase was washed with 3 × 20 mL of H_2O. The organic phase was dried over Na_2SO_4. This mixture was vacuum filtered, and the solvent was removed under vacuum. Purification of the residue by flash column chromatography (silica gel, PE/EA = 20:1) yielded the **1a** (2.7 g, 97%) as white solid. R_f (5% EtOAc/hexane) = 0.5. ^{1}H NMR (400 MHz, $CDCl_3$) δ 7.62 – 7.55 (m, 10H), 5.16 (d, J_{AB} = 11.4 Hz, 2H), 5.05 (d, J_{AB} = 11.4 Hz, 2H). ^{13}C NMR (100 MHz, $CDCl_3$) δ 137.0, 129.0, 128.9, 128.8, 77.1. IR (film) 3032, 2939, 2870, 1496, 1454, 1363, 1269, 1211, 951, 744, 696, 656, 577. HRMS (EI) Calcd for $C_{14}H_{14}O_2S_2$ 278.0435, found 278.0430.

Di(1-phthalimidyl)disulfane (**1b**)

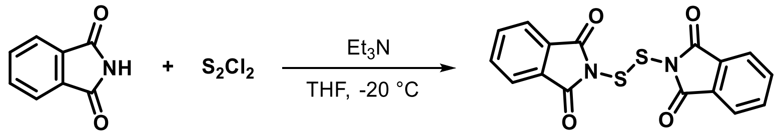

1b

Di(1-phthalimidyl)disulfane (**1b**) was prepared according to a literature procedure[3]: A solution of phthalimide (5.8 g, 40 mmol) and NEt_3 (8.2 mL, 60 mmol) in 80 mL of THF was allowed to stir under N_2 at -20 °C. A solution of S_2Cl_2 (1.6 mL, 20 mmol) in 5 mL of THF was added dropwise over 5 min. The reaction mixture was allowed to stir for a further 30 min at -20 °C. The reaction mixture was quenched with 100 mL of H_2O and allowed to warm to room temperature. The resulting precipitate was filtered and washed with 20 mL of diethyl ether. Crystallization with $CHCl_3$: MeOH (2:1) yielded di(1-phthalimidyl)disulfane **1b** (6.4 g, 90%) as a white solid. ^{1}H NMR (400 MHz, $CDCl_3$) δ 7.98 (dd, J = 5.5, 3.1 Hz, 1H), 7.85 (dd, J = 5.5, 3.1 Hz, 1H). ^{13}C NMR (100 MHz, $CDCl_3$) δ 166.6, 135.0, 132.2, 124.4. IR (film) 3088, 3055, 1788, 1747, 1604, 1465, 1265, 1035, 864, 740, 709, 571. HRMS (ESI) Calcd for $C_{16}H_9N_2O_4S_2$ (M+H$^+$) 356.9998, found 356.9988.

Di-tert-butyl benzo[*d*][1,2,3,6]dithiadiazine-1,4-dicarboxylate (**1c**)

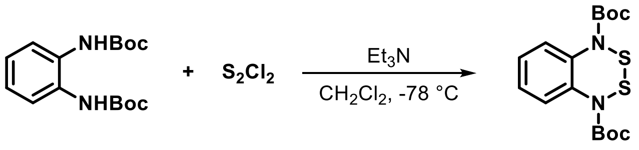

1c

Preparation of di-tert-butyl benzo[*d*][1,2,3,6]dithiadiazine-1,4-dicarboxylate (**1c**): A solution of di-tert-butyl 1,2-phenylenedicarbamate (3.08 g, 10 mmol) and NEt_3 (2.8 mL, 20 mmol) in 200 mL of CH_2Cl_2 was allowed to stir under N_2 at -78 °C. A solution of S_2Cl_2 (0.8 mL, 10 mmol) in 100 mL of CH_2Cl_2 was added dropwise over 60 min. The reaction mixture was allowed to stir for a further 5 min at -78 °C. The reaction mixture was quenched with 50 mL of saturated $NaHCO_3$ and allowed to warm to room temperature. The organic phase was washed with 3 × 60 mL of H_2O. The organic phase was dried over Na_2SO_4. This mixture was vacuum filtered, and the solvent was removed under vacuum. Purification of the residue by flash column chromatography (silica gel, PE/EA = 20:1→10:1) yielded the **1c** (2.60 g, 70%) as a white solid. R_f (5% EA/hexane) = 0.2. ^1H NMR (400 MHz, $CDCl_3$) δ 7.46 – 7.40 (m, 2H), 7.12 – 7.06 (m, 2H), 1.45 (s, 18H). ^{13}C NMR (100 MHz, $CDCl_3$) δ 154.5, 130.6, 127.9, 124.2, 84.0, 28.0. IR (film) 2980, 1730, 1485, 1369, 1294, 1269, 1249, 1145, 962, 841, 752. HRMS (EI) Calcd for $C_{16}H_{22}N_2O_4S_2$ 370.1021, found 370.1024.

1,6-Dihydrobenzo[*f*][1,4,2,3]dioxadithiocine (**1d**-**1e**)

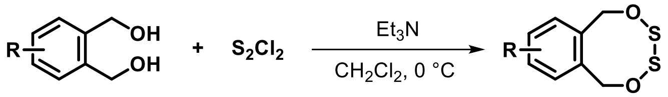

1,6-Dihydrobenzo[*f*][1,4,2,3]dioxadithiocines (**1d**-**1e**) were prepared according to modified literature procedures[4]: A solution of 1,2-phenylenedimethanol (1.38 g, 10 mmol) and NEt_3 (2.8 mL, 20 mmol) in 100 mL of CH_2Cl_2 was allowed to stir under N_2 at 0 °C. A solution of S_2Cl_2 (0.8 mL, 10 mmol) in 30 mL of CH_2Cl_2 was added dropwise over 20 min. The reaction mixture was allowed to stir for a further 5 min at 0 °C. The reaction mixture was quenched with 50 mL of saturated $NaHCO_3$ and allowed to warm to room temperature. The organic phase was washed with 3 × 60 mL of saturated $NaHCO_3$. The organic phase was dried over Na_2SO_4. This mixture was vacuum filtered, and the solvent was removed under vacuum. Purification of the residue by flash column chromatography (silica gel, PE/EA = 20:1) yielded the **1d**-**1e**. The NMR spectra are consistent with literature reports.[4]

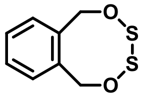

1d

Compound **1d** was isolated in 95% yield as a white solid. R_f (5% EtOAc/hexane) = 0.5. ^1H NMR (400 MHz, $CDCl_3$) δ 7.42 – 7.33 (m, 4H), ABq system 5.01 (d, J_{AB} = 12.2 Hz, 2H), 4.88 (d, J_{AB} = 12.2 Hz, 2H). ^{13}C NMR (100 MHz, $CDCl_3$) δ 136.3, 132.3, 130.3, 72.3. IR (film) 3078, 1660, 1558, 1387, 1205, 1128, 1076, 1018, 864. HRMS (EI) Calcd for $C_8H_8O_2S_2$ 199.9966, found 199.9960.

5,10-Dihydrodibenzo[*f*,*h*][1,4,2,3]dioxadithiecine (**1f**)

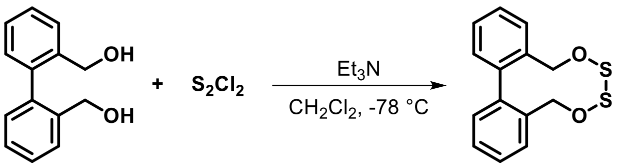

1f

5,10-Dihydrodibenzo[*f*,*h*][1,4,2,3]dioxadithiecine (**1f**) was prepared according to a modified literature procedure[4]: A solution of 2,2'-biphenyldimethanol (10.7 g, 50 mmol) and NEt_3 (14 mL, 100 mmol) in 1000 mL of CH_2Cl_2 was allowed to stir under N_2 at -78 °C. A solution of S_2Cl_2 (4 mL, 50 mmol) in 200 mL of CH_2Cl_2 was added dropwise over 4 h. The reaction mixture was allowed to stir for a further 2 h at -78 °C. The reaction mixture was quenched with 250 mL of saturated $NaHCO_3$ and allowed to warm to room temperature. The organic phase was washed with 3 × 300 mL of H_2O. The organic phase was dried over Na_2SO_4. This mixture was vacuum filtered, and the solvent was removed under vacuum. Purification of the residue by flash column chromatography (silica gel, PE/DCM = 5:1→3:1) yielded the **1f** (10.8 g, 78%) as a white to light yellow solid. R_f (5% EA/hexane) = 0.5. ^{1}H NMR (300 MHz, $CDCl_3$) δ 7.57 – 7.34 (m, 6H), 7.27 – 7.15 (m, 2H), AB system 5.11 (dd, J = 49.9, J_{AB} = 12.7 Hz, 2H), 4.60 (dd, J_{AB} = 12.6, J = 9.4 Hz, 2H). ^{13}C NMR (100 MHz, $CDCl_3$) δ 140.8, 139.6, 136.0, 134.8, 131.5, 130.0, 129.7, 129.5, 129.1, 128.4, 127.9, 127.8, 76.6, 70.4. 5:1 of two diastereomers as detected by ^{13}C NMR, ^{1}H NMR. IR (film) 3003, 2943, 1637, 1443, 1375, 1273, 1197, 1041, 920, 738, 669. HRMS (ESI) Calcd for $C_{14}H_{12}O_2S_2Na$ (M+Na$^+$) 299.0171, found 299.0167.

Disulurating reagents that we failed to synthesize

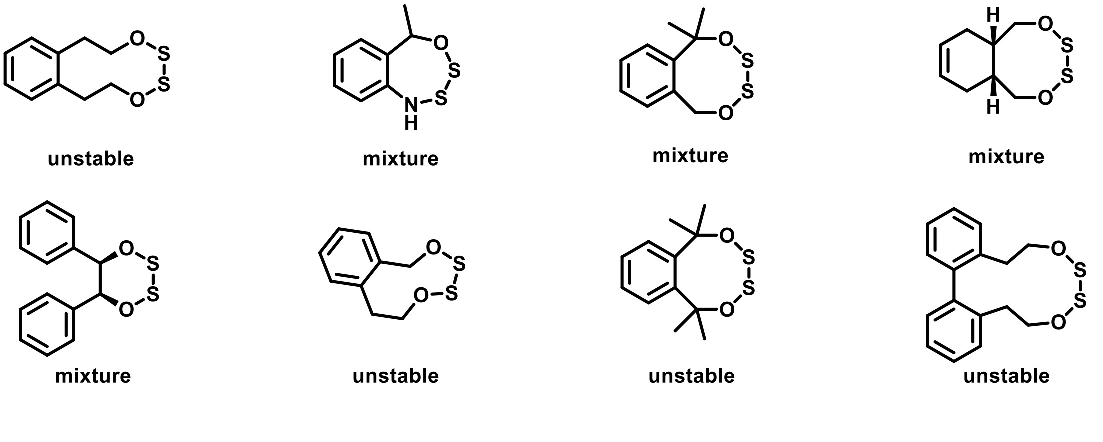

Optimization of polysulfuration with Amine

Supplementary Table 1. Optimization of disulfurating reagent with aniline[a][b].

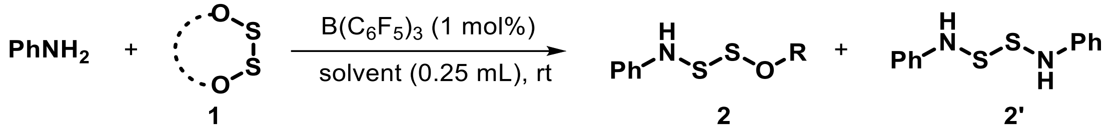

Entry	**1**	Solvent	2	2'
1	**1f**	1,4-dioxane	99%	-
2	**1f**	THF	95%	<5%
3	**1f**	acetone	86	<5%
4	**1f**	MeCN	70%	10%
5	**1f**	toluene	71%	13%
6	**1f**	CH_2Cl_2	50%	45%
7	**1d**	1,4-dioxane	trace	trace
8	**1e**	1,4-dioxane	trace	trace
9	**1c**	1,4-dioxane	N.R.	N.R.
10	**1a**	1,4-dioxane	60%	30%
11	**1b**	1,4-dioxane	30%	60%

[a]Condition: aniline (0.10 mmol), **1** (0.105 mmol), $B(C_6F_5)_3$ (0.001 mmol, 1 mol%), 1,4-dioxane (0.25 mL), rt, under air, 4 h. [b]Isolated yield.

To a Schlenk tube were added aniline (9.3 mg, 0.1 mmol, 1.0 equiv), **1** (0.105 mmol, 1.05 equiv), $B(C_6F_5)_3$ (0.5 mg, 0.001 mmol, 1 mol%), and distilled 1,4-dioxane (0.25 mL), the mixture was stirred at r.t. for 4 hours under air before it was concentrated under vacuum. Purification of the residue by flash column chromatography (silica gel, PE/DCM = 1:1→PE/DCM/EA = 10:4:1) yielded the **2** as a colorless oil. R_f (20% EA/hexane) = 0.3.

(2'-((((phenylamino)disulfanyl)oxy)methyl)-[1,1'-biphenyl]-2-yl)methanol (**2a**)

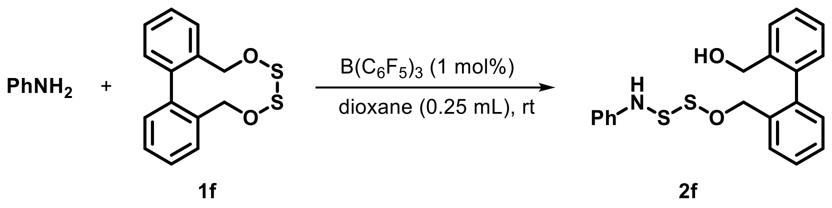

To a Schlenk tube were added aniline (9.3 mg, 0.1 mmol, 1.0 equiv), **1f** (29.0 mg, 0.105 mmol, 1.05 equiv), $B(C_6F_5)_3$ (0.5 mg, 0.001 mmol, 1 mol%), and distilled 1,4-dioxane (0.25 mL), the mixture was stirred at r.t. for 4 hours under air before it was concentrated under vacuum. Purification of the residue by flash column chromatography (silica gel, PE/DCM = 1:1→PE/DCM/EA = 10:4:1) yielded the **2f** (36.9 mg, 99%) as a colorless oil. R_f (20% EA/hexane) = 0.3. ^{1}H NMR (400 MHz, DMSO-d_6) δ 8.00 (s, 1H), 7.59 (d, *J* = 7.5 Hz, 1H), 7.46 (dd, *J* = 6.2, 3.0 Hz, 1H), 7.41 (tt, *J* = 7.0, 3.6 Hz, 3H), 7.30 (t, *J* = 7.4 Hz, 1H), 7.23 – 7.15 (m, 3H), 7.04 (d, *J* = 7.4 Hz, 1H), 6.97 (d, *J* = 7.7 Hz, 2H), 6.84 (t, *J* = 7.3 Hz, 1H), 5.06 (t, *J* = 4.8 Hz, 1H), 4.49 (dd, *J* = 28.6, 11.2 Hz, 2H), 4.14 (ddd, *J* = 40.1, 13.5, 4.7 Hz, 2H). ^{13}C NMR (100 MHz, DMSO-d_6) δ 146.2, 140.6, 140.2, 138.1, 134.9, 130.1, 130.0, 129.6, 129.5, 128.7, 128.1, 128.0, 127.5, 126.7, 120.9, 116.6, 76.2, 61.2. IR (film) 3350, 2978, 2885, 1690, 1410, 1329, 1092, 1051, 885, 737. HRMS (ESI) Calcd for $C_{20}H_{19}NO_2S_2Na$ (M+Na$^+$) 392.0749, found 392.0745.

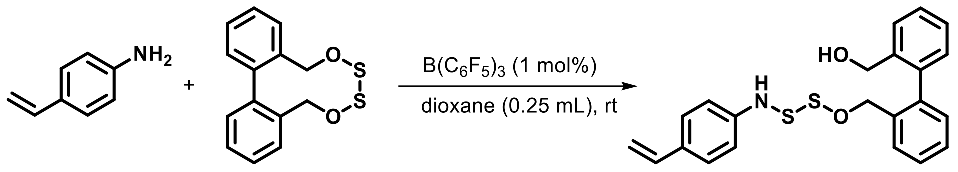

1f **S3**

To a Schlenk tube were added 4-vinylaniline (11.9 mg, 0.1 mmol, 1.0 equiv), **1f** (29.0 mg, 0.105 mmol, 1.05 equiv), $B(C_6F_5)_3$ (0.5 mg, 0.001 mmol, 1 mol%), and distilled 1,4-dioxane (0.25 mL), the mixture was stirred at r.t. for 4 hours under air. Purification of the residue by flash column chromatography (silica gel, PE/EA = 10:1) yielded the **S3** (24.5 mg, 62%) as a colorless oil. R_f (20% EA/hexane) = 0.3.

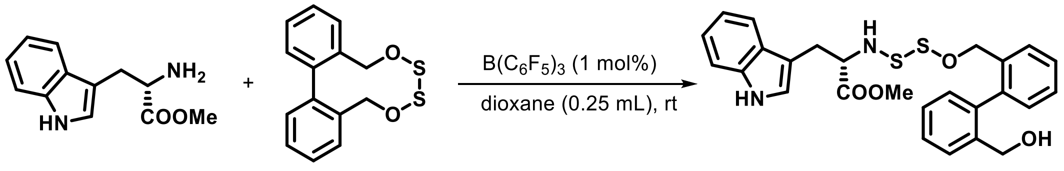

1f **S4**

To a Schlenk tube were added methyl L-tryptophanate (21.8 mg, 0.1 mmol, 1.0 equiv), **1f** (29.0 mg, 0.105 mmol, 1.05 equiv), $B(C_6F_5)_3$ (0.5 mg, 0.001 mmol, 1 mol%), and distilled MeCN (0.5 mL), the mixture was stirred at r.t. for 12 hours under air. Purification of the residue by flash column chromatography (silica gel, PE/ EA = 5:1) yielded the **S4** (34.8 mg, 36%) as a colorless oil. R_f (50% EA/hexane) = 0.3.

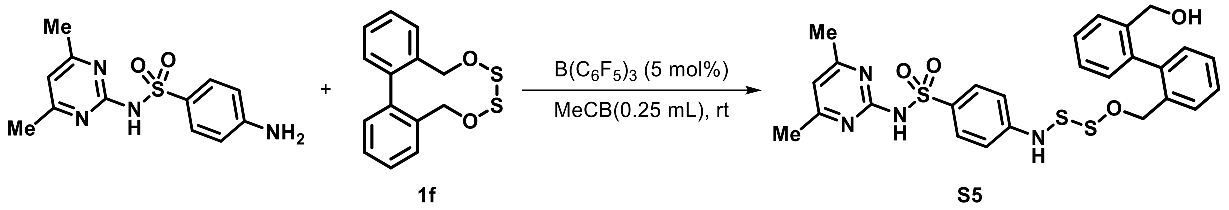

To a Schlenk tube were added sulfamethazine (27.8 mg, 0.1 mmol, 1.0 equiv), **1f** (29.0 mg, 0.105 mmol, 1.05 equiv), $B(C_6F_5)_3$ (2.5 mg, 0.005 mmol, 5 mol%), and distilled 1,4-dioxane (0.25 mL), the mixture was stirred at r.t. for 24 hours under air. Purification of the residue by flash column chromatography (silica gel, DCM/MeOH = 100:1→50:1) yielded the **S5** (47.1 mg, 85%) as a viscous gel. R_f (5% DCM/hexane) = 0.3.

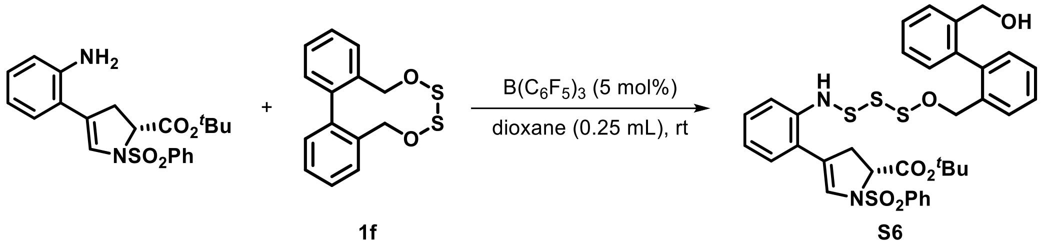

To a Schlenk tube were added tert-butyl (R)-4-(2-aminophenyl)-1-(phenylsulfonyl)-2,3-dihydro-1H-pyrrole-2-carboxylate (40.0 mg, 0.1 mmol, 1.0 equiv), **1f** (29.0 mg, 0.105 mmol, 1.05 equiv), $B(C_6F_5)_3$ (2.5 mg, 0.005 mmol, 5 mol%), and distilled 1,4-dioxane (0.25 mL), the mixture was stirred at r.t. for 12 hours under air. Purification of the residue by flash column chromatography (silica gel, EA/PE = 2:1→1:1) yielded the **S6** (53.1 mg, 75%) as a viscous gel. R_f (50% EA/hexane) = 0.2.

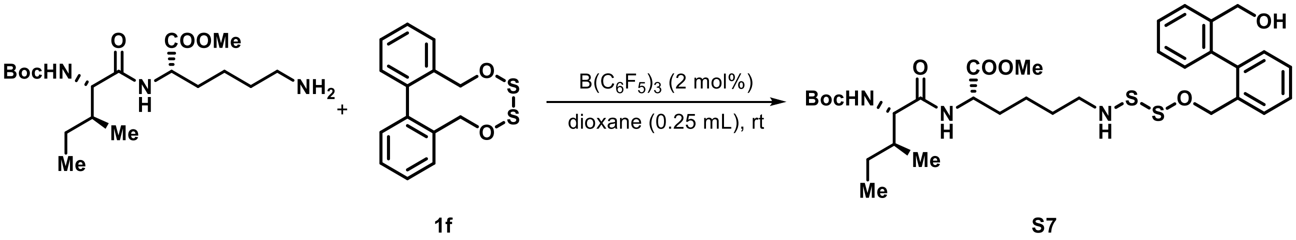

To a Schlenk tube were added methyl (tert-butoxycarbonyl)-L-isoleucyl-L-lysinate (37.3 mg, 0.1 mmol, 1.0 equiv), **1f** (29.0 mg, 0.105 mmol, 1.05 equiv), $B(C_6F_5)_3$ (1.0 mg, 0.002 mmol, 2 mol%), and distilled 1,4-dioxane (0.25 mL), the mixture was stirred at r.t. for 4 hours under air. Purification of the residue by flash column chromatography (silica gel, MeOH/DCM = 50:1) yielded the **S7** (36.4 mg, 56%) as a colorless oil. R_f (2% MeOH/DCM) = 0.4.

General Procedure and Spectra Data of Polysulfuration with Amine.

Synthesis of compound **3**

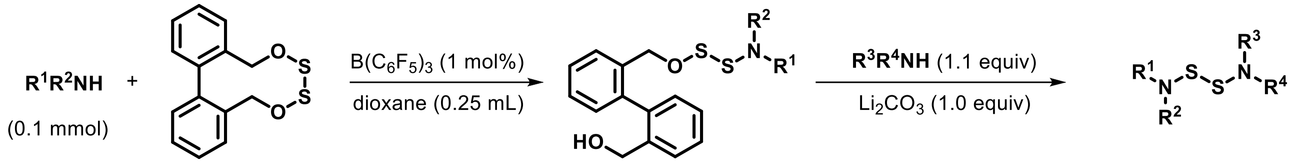

To a Schlenk tube were added amine (0.1 mmol, 1.0 equiv), $B(C_6F_5)_3$ (0.5 mg, 0.001 mmol, 1 mol%),**1f** (29.0 mg, 0.105 mmol, 1.05 equiv), and 1,4-dioxane (0.25 mL), the mixture was stirred at r.t. for 4 hours to obtain **2**.

After amine was consumed, another amine (0.12 mmol, 1.2 equiv) and Li_2CO_3 (7.4 mg, 0.1 mmol, 1.0 equiv) were added to the mixture. The mixture was stirred at r.t. for 12 hours before it was concentrated under vacuum. Purification by column chromatography afforded the desired product **3**.

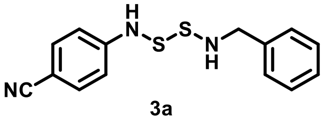

3a

The reaction of 4-aminobenzonitrile (11.8 mg, 0.1 mmol, 1.0 equiv), $B(C_6F_5)_3$ (1.0 mg, 0.002 mmol, 2 mol%) and **1f** (29.0 mg, 0.105 mmol, 1.05 equiv) in 1,4-dioxane (0.25 mL) at r.t. for 24 hours, then benzylamine (12.8 mg, 0.12 mmol, 1.2 equiv) and Li_2CO_3 (7.4 mg, 0.1 mmol, 1.0 equiv) at r.t. for 12 hours afforded compound **3a** in 71% yield as a white solid by chromatography on silica gel eluting with PE/EA (5:1). R_f (20% EA/PE) = 0.3. ^1H NMR (400 MHz, Acetone-d_6) δ 7.61 (d, *J* = 8.7 Hz, 2H), 7.33 (d, *J* = 4.0 Hz, 4H), 7.29 (dt, *J* = 9.4, 4.8 Hz, 1H), 7.23 (d, *J* = 8.8 Hz, 2H), 4.64 (t, *J* = 5.0 Hz, 1H), 4.19 (d, *J* = 5.3 Hz, 2H), 2.10 (s, 1H). ^{13}C NMR (100 MHz, Acetone-d_6) δ 151.2, 138.8, 133.3, 128.5, 128.3, 127.3, 119.2, 116.0, 102.3, 55.0. IR (film) 3361, 2974, 2926, 2222, 1383, 1335, 1093, 1053, 883, 804, 574. HRMS (EI) Calcd for $C_{14}H_{13}N_3S_2$ 287.0551, found 287.0549.

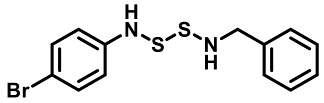

3b

The reaction of 4-bromoaniline (17.2 mg, 0.1 mmol, 1.0 equiv), $B(C_6F_5)_3$ (0.5 mg, 0.001 mmol, 1 mol%) and **1f** (29.0 mg, 0.105 mmol, 1.05 equiv) in 1,4-dioxane (0.25 mL) at r.t. for 4 hours, then benzylamine (12.8 mg, 0.12 mmol, 1.2 equiv) and Li_2CO_3 (7.4 mg, 0.1 mmol, 1.0 equiv) at r.t. for 12 hours afforded compound **3b** in 78% yield as a pale yellow solid by chromatography on silica gel eluting with PE/EA (20:1→10:1). R_f (5% EA/PE) = 0.3. ^1H NMR (400 MHz, $CDCl_3$) δ 7.25 (dt, *J* = 11.8, 4.9 Hz, 5H), 7.19 (d, *J* = 7.8 Hz, 2H), 6.79 (d, *J* = 8.5 Hz, 2H), 4.77 (s, 1H), 3.99 (d, *J* = 3.3 Hz, 2H), 3.07 (s, 1H). ^{13}C NMR (100 MHz, $CDCl_3$) δ 145.4, 138.5, 132.1, 128.7, 128.4, 127.8, 118.0,

113.3, 55.8. IR (film) 3360, 3294, 1585, 1481, 1435, 1365, 1276, 1228, 1113, 1001, 887, 812, 748, 696, 632, 578. HRMS (EI) Calcd for $C_{13}H_{13}BrN_2S_2$ 339.9704, found 339.9709.

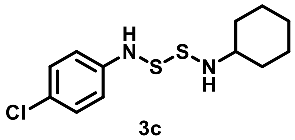
3c

The reaction of 4-chloroaniline (12.7 mg, 0.1 mmol, 1.0 equiv), $B(C_6F_5)_3$ (0.5 mg, 0.001 mmol, 1 mol%) and **1f** (29.0 mg, 0.105 mmol, 1.05 equiv) in 1,4-dioxane (0.25 mL) at r.t. for 4 hours, then cyclohexanamine (11.9 mg, 0.12 mmol, 1.2 equiv) and Li_2CO_3 (7.4 mg, 0.1 mmol, 1.0 equiv) at r.t. for 12 hours afforded compound **3c** in 75% yield as a colorless oil by chromatography on silica gel eluting with PE/EA (20:1→10:1). R_f (5% EA/PE) = 0.3. ^{1}H NMR (400 MHz, $CDCl_3$) δ 7.12 (d, *J* = 8.8 Hz, 2H), 6.89 (d, *J* = 8.8 Hz, 2H), 5.01 (s, 1H), 2.80 (s, 1H), 2.73 – 2.63 (m, 1H), 1.79 (d, *J* = 11.7 Hz, 2H), 1.61 (dd, *J* = 8.5, 4.5 Hz, 2H), 1.15 – 0.93 (m, 6H). ^{13}C NMR (100 MHz, $CDCl_3$) δ 145.3, 129.1, 125.9, 117.6, 57.7, 32.6, 25.8, 24.4. IR (film) 3329, 2935, 2856, 1774, 1595, 1489, 1448, 1276, 1232, 825, 739. HRMS (EI) Calcd for $C_{12}H_{17}ClN_2S_2$ 288.0522, found 288.0525.

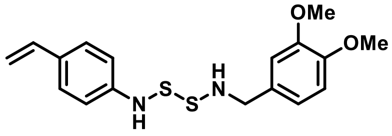
3d

The reaction of 4-vinylaniline (11.9 mg, 0.1 mmol, 1.0 equiv), $B(C_6F_5)_3$ (0.5 mg, 0.001 mmol, 1 mol%) and **1f** (29.0 mg, 0.105 mmol, 1.05 equiv) in 1,4-dioxane (0.25 mL) at r.t. for 4 hours, then (3,4-dimethoxyphenyl)methanamine (20.0 mg, 0.12 mmol, 1.2 equiv) and Li_2CO_3 (7.4 mg, 0.1 mmol, 1.0 equiv) at r.t. for 12 hours afforded compound **3d** in 55% yield as a white solid by chromatography on silica gel eluting with PE/EA (10:1→5:1). R_f (10% EA/PE) = 0.3. ^{1}H NMR (400 MHz, $CDCl_3$) δ 7.24 (dd, *J* = 17.5, 7.4 Hz, 2H), 6.92 (dd, *J* = 16.7, 8.3 Hz, 2H), 6.81 – 6.66 (m, 3H), 6.64 – 6.49 (m, 1H), 5.54 (t, *J* = 17.3 Hz, 1H), 5.13 – 4.89 (m, 1H), 3.93 (d, *J* = 17.4 Hz, 1H), 4.01 – 3.41 (m, 2H), 3.80 (dd, *J* = 16.8, 7.5 Hz, 6H), 3.01 (s, 1H). ^{13}C NMR (100 MHz, DMSO-d_6) δ 149.0, 148.4, 147.4, 136.8, 131.7, 129.5, 127.4, 120.9, 116.3, 112.6, 112.0, 111.3, 56.0, 55.8, 54.9. IR (film) 3200, 3055, 2835, 1604, 1508, 1325, 1269, 1238, 1139, 1026, 738. HRMS (ESI) Calcd for $C_{17}H_{20}N_2O_2S_2$ $(M+H^+)$ 349.1039, found 349.1037.

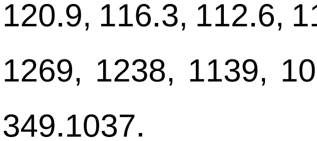
3e

The reaction of 4-chloroaniline (12.7 mg, 0.1 mmol, 1.0 equiv), $B(C_6F_5)_3$ (0.5 mg, 0.001 mmol, 1 mol%) and **1f** (29.0 mg, 0.105 mmol, 1.05 equiv) in 1,4-dioxane (0.25 mL) at r.t. for 4 hours, then 2,2-diphenylethan-1-amine (23.6 mg, 0.12 mmol, 1.2 equiv) and Li_2CO_3 (7.4 mg, 0.1 mmol, 1.0 equiv) at r.t. for 12 hours afforded compound **3e** in 90% yield as a light yellow oil by chromatography on silica gel eluting with PE/EA (20:1). R_f (5% EA/PE) = 0.3. ^{1}H NMR (400 MHz, $CDCl_3$) δ 7.23 (t, *J* = 7.5 Hz, 4H), 7.15 (d, *J* = 6.7 Hz, 2H), 7.09 (d, *J* = 7.7 Hz, 4H), 7.06 (d, *J* = 8.6 Hz, 2H), 6.77 (d, *J* = 8.4

Hz, 2H), 4.82 (s, 1H), 4.11 (t, J = 7.6 Hz, 1H), 3.45 – 3.34 (m, 2H), 2.70 (s, 1H). ^{13}C NMR (100 MHz, $CDCl_3$) δ 144.8, 142.0, 129.2, 128.8, 128.1, 126.9, 126.1, 117.7, 56.8, 51.1. IR (film) 3358, 3061, 2924, 2852, 1593, 1487, 1275, 1230, 889, 823, 738, 702. HRMS (EI) Calcd for $C_{20}H_{19}ClN_2S_2$ 386.0678, found 386.0681.

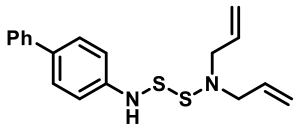

3f

The reaction of [1,1'-biphenyl]-4-amine (16.9 mg, 0.1 mmol, 1.0 equiv), $B(C_6F_5)_3$ (0.5 mg, 0.001 mmol, 1 mol%) and **1f** (29.0 mg, 0.105 mmol, 1.05 equiv) in 1,4-dioxane (0.25 mL) at r.t. for 4 hours, then diallylamine (11.6 mg, 0.12 mmol, 1.2 equiv) and Li_2CO_3 (7.4 mg, 0.1 mmol, 1.0 equiv) at r.t. for 12 hours afforded compound **3f** in 67% yield as a light yellow oil by chromatography on silica gel eluting with PE/EA (20:1). R_f (5% EA/PE) = 0.4. ^{1}H NMR (400 MHz, $CDCl_3$) δ 7.50 – 7.40 (m, 4H), 7.36 – 7.28 (m, 2H), 7.24 – 7.17 (m, 1H), 6.99 – 6.92 (m, 2H), 5.87 – 5.64 (m, 2H), 5.10 – 4.94 (m, 4H), 4.83 (s, 1H), 3.38 – 3.34 (m, 4H). ^{13}C NMR (100 MHz, $CDCl_3$) δ 145.9, 140.8, 134.9, 134.0, 128.8, 127.9, 126.7, 126.6, 118.1, 116.7, 60.3. IR (film) 3373, 3030, 2920, 1714, 1606, 1516, 1485, 1361, 1282, 1265, 1224, 991, 925, 889, 833, 761, 698. HRMS (EI) Calcd for $C_{18}H_{20}N_2S_2$ 328.1068, found 328.1070.

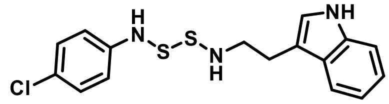

3g

The reaction of 4-chloroaniline (12.7 mg, 0.1 mmol, 1.0 equiv), $B(C_6F_5)_3$ (0.5 mg, 0.001 mmol, 1 mol%) and **1f** (29.0 mg, 0.105 mmol, 1.05 equiv) in 1,4-dioxane (0.25 mL) at r.t. for 4 hours, then tryptamine (19.2 mg, 0.12 mmol, 1.2 equiv) and Li_2CO_3 (7.4 mg, 0.1 mmol, 1.0 equiv) at r.t. for 12 hours afforded compound **3g** in 72% yield as a pale yellow solid by chromatography on silica gel eluting with PE/EA (5:1). R_f (10% EA/PE) = 0.3. ^{1}H NMR (400 MHz, $CDCl_3$) δ 7.91 (s, 1H), 7.57 (t, J = 7.2 Hz, 1H), 7.33 (t, J = 6.7 Hz, 1H), 7.23 – 7.15 (m, 1H), 7.10 (dd, J = 14.6, 7.3 Hz, 1H), 6.94 – 6.83 (m, 3H), 6.72 – 6.63 (m, 2H), 4.70 (s, 1H), 3.20 (s, 3H), 2.99 – 2.84 (m, 3H), 2.76 (s, 1H). ^{13}C NMR (100 MHz, $CDCl_3$) δ 144.8, 136.5, 129.0, 127.2, 125.7, 122.4, 122.4, 119.6, 118.8, 117.3, 112.8, 111.5, 51.2, 25.6. IR (film) 3314, 3061, 2924, 2868, 1593, 1487, 1275, 1230, 889, 823, 739. 702. HRMS (ESI) Calcd for $C_{16}H_{16}ClN_3S_2$ ($M+H^+$) 350.0547, found 350.0546.

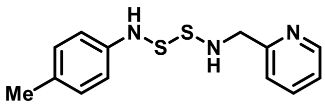

3h

The reaction of p-toluidine (10.7 mg, 0.1 mmol, 1.0 equiv), $B(C_6F_5)_3$ (0.5 mg, 0.001 mmol, 1 mol%) and **1f** (29.0 mg, 0.105 mmol, 1.05 equiv) in 1,4-dioxane (0.25 mL) at r.t. for 4 hours, then pyridin-2-ylmethanamine (13.0 mg, 0.12 mmol, 1.2 equiv) and Li_2CO_3 (7.4 mg, 0.1 mmol, 1.0 equiv) at r.t. for 12 hours afforded compound **3h** in 73% yield as a brown oil by chromatography on silica gel

eluting with PE/EA (5:1→3:1). R_f (20% EA/PE) = 0.3. ^{1}H NMR (400 MHz, DMSO-d_6) δ 8.48 (d, *J* = 4.2 Hz, 1H), 7.72 (td, *J* = 7.7, 1.8 Hz, 1H), 7.54 (s, 1H), 7.33 (d, *J* = 7.8 Hz, 1H), 7.27 – 7.21 (m, 1H), 7.00 (d, *J* = 8.3 Hz, 2H), 6.90 (d, *J* = 8.4 Hz, 2H), 5.14 (t, *J* = 5.5 Hz, 1H), 4.13 (d, *J* = 5.5 Hz, 2H), 2.18 (s, 3H). ^{13}C NMR (100 MHz, DMSO-d_6) δ 159.0, 149.3, 144.8, 137.0, 129.9, 129.0, 122.8, 122.7, 116.4, 56.8, 20.6. IR (film) 2920, 2848, 1714, 1633, 1508, 1431, 1361, 1093, 763, 723. HRMS (ESI) Calcd for $C_{13}H_{16}N_3S_2$ (M+H$^+$) 278.0780, found 278.0782.

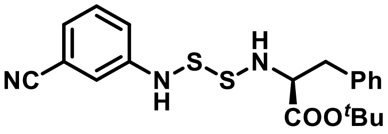

3i

The reaction of 3-aminobenzonitrile (11.8 mg, 0.1 mmol, 1.0 equiv), $B(C_6F_5)_3$ (1.0 mg, 0.002 mmol, 2 mol%) and **1f** (29.0 mg, 0.105 mmol, 1.05 equiv) in 1,4-dioxane (0.25 mL) at r.t. for 12 hours, then tert-butyl L-phenylalaninate (26.5 mg, 0.12 mmol, 1.2 equiv) and Li_2CO_3 (7.4 mg, 0.1 mmol, 1.0 equiv) at r.t. for 12 hours afforded compound **3i** in 58% yield as a colorless oil by chromatography on silica gel eluting with DCM. R_f (50% EA/PE) = 0.3. ^{1}H NMR (300 MHz, $CDCl_3$) δ 7.22 (ddd, *J* = 11.2, 4.9, 2.3 Hz, 5H), 7.14 – 7.06 (m, 4H), 5.37 (s, 1H), 3.66 (dt, *J* = 8.5, 6.9 Hz, 1H), 3.56 (d, *J* = 8.6 Hz, 1H), 2.89 (d, *J* = 6.7 Hz, 2H), 1.38 (s, 9H). ^{13}C NMR (100 MHz, $CDCl_3$) δ 169.4, 141.6, 131.6, 125.2, 124.7, 123.7, 122.2, 119.7, 116.2, 114.6, 114.2, 108.3, 77.8, 61.9, 34.7, 23.2. IR (film) 3348, 3030, 2980, 2229, 1726, 1598, 1583, 1495, 1456, 1392, 1369, 1269, 1151, 740, 702. HRMS (ESI) Calcd for $C_{20}H_{23}N_3O_2S_2$ (M+Na$^+$) 424.1124, found 424.1119.

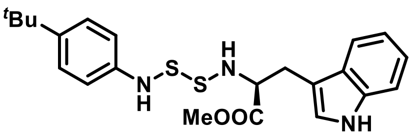

3j

The reaction of 4-(tert-butyl)aniline (14.9 mg, 0.1 mmol, 1.0 equiv), $B(C_6F_5)_3$ (0.5 mg, 0.001 mmol, 1 mol%) and **1f** (29.0 mg, 0.105 mmol, 1.05 equiv) in 1,4-dioxane (0.25 mL) at r.t. for 4 hours, then methyl L-tryptophanate (26.1 mg, 0.12 mmol, 1.2 equiv) and Li_2CO_3 (7.4 mg, 0.1 mmol, 1.0 equiv) at r.t. for 12 hours afforded compound **3j** in 66% yield as a pale yellow solid by chromatography on silica gel eluting with PE/EA (8:1→5:1). R_f (20% EA/PE) = 0.4. ^{1}H NMR (400 MHz, $CDCl_3$) δ 7.98 (s, 1H), 7.55 (d, *J* = 7.8 Hz, 1H), 7.28 (d, *J* = 8.1 Hz, 1H), 7.12 (dd, *J* = 8.6, 2.3 Hz, 3H), 7.06 (t, *J* = 7.4 Hz, 1H), 6.92 (d, *J* = 2.1 Hz, 1H), 6.72 (d, *J* = 8.6 Hz, 2H), 4.62 (s, 1H), 3.97 (dd, *J* = 13.2, 6.4 Hz, 1H), 3.65 (s, 3H), 3.45 (d, *J* = 6.4 Hz, 1H), 3.11 (ddd, *J* = 22.3, 14.6, 6.6 Hz, 2H), 1.19 (s, 9H). ^{13}C NMR (100 MHz, $CDCl_3$) δ 175.0, 144.0, 142.7, 136.2, 127.2, 126.0, 123.3, 122.4, 119.8, 118.8, 116.1, 111.4, 110.4, 63.7, 52.5, 52.5, 34.1, 31.5, 28.9. IR (film) 3495, 3290, 2926, 1728, 1510, 1282, 1093, 1053, 881, 827, 742, 643. HRMS (ESI) Calcd for $C_{22}H_{28}N_3O_2S_2$ (M+H$^+$) 430.1617, found 430.1616.

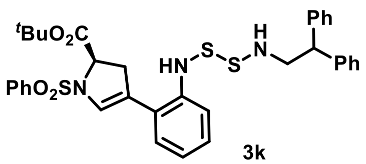

3k

The reaction of tert-butyl (R)-4-(2-aminophenyl)-1-(phenylsulfonyl)-2,3-dihydro-1H-pyrrole-2-carboxylate (40.0 mg, 0.1 mmol, 1.0 equiv), $B(C_6F_5)_3$ (2.5 mg, 0.005 mmol, 5 mol%) and **1f** (29.0 mg, 0.105 mmol, 1.05 equiv) in 1,4-dioxane (0.25 mL) at r.t. for 12 hours, then 2,2-diphenylethan-1-amine (23.6 mg, 0.12 mmol, 1.2 equiv) and Li_2CO_3 (7.4 mg, 0.1 mmol, 1.0 equiv) at r.t. for 12 hours afforded compound **3k** in 62% yield as a brown foam by chromatography on silica gel eluting with PE/EA (10:1→5:1). R_f (10% EA/PE) = 0.2. ^{1}H NMR (400 MHz, $CDCl_3$) δ 7.78 (d, *J* = 6.8 Hz, 2H), 7.54 (dd, *J* = 8.4, 6.2 Hz, 1H), 7.47 (t, *J* = 7.2 Hz, 2H), 7.17 (ddd, *J* = 20.3, 14.0, 7.0 Hz, 8H), 7.05 (d, *J* = 6.7 Hz, 4H), 6.81 (d, *J* = 7.5 Hz, 1H), 6.75 (t, *J* = 7.2 Hz, 1H), 6.53 (s, 1H), 5.22 (s, 1H), 4.21 (t, *J* = 7.6 Hz, 1H), 4.06 (t, *J* = 6.9 Hz, 1H), 3.30 (s, 2H), 2.90 – 2.73 (m, 2H), 2.69 (t, *J* = 4.8 Hz, 1H), 1.44 (s, 9H). ^{13}C NMR (100 MHz, $CDCl_3$) δ 169.8, 143.9, 142.1, 142.0, 136.7, 133.5, 129.4, 128.7, 128.4, 128.0, 127.9, 127.6, 126.8, 122.7, 121.3, 120.7, 117.3, 82.8, 61.1, 56.8, 51.1, 38.1, 28.0. IR (film) 3489, 3001, 1633, 1446, 1373, 1168, 1149, 1089, 1041, 754, 721. HRMS (ESI) Calcd for $C_{35}H_{37}N_3O_4S_3$ ($M+H^+$) 660.2019, found 660.2010.

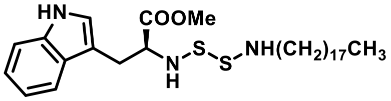

3l

The reaction of L-tryptophanate (21.8 mg, 0.1 mmol, 1.0 equiv), $B(C_6F_5)_3$ (1.0 mg, 0.002 mmol, 2 mol%) and **1f** (29.0 mg, 0.105 mmol, 1.05 equiv) in MeCN (0.5 mL) at r.t. for 12 hours, then octadecanamine (32.3 mg, 0.12 mmol, 1.2 equiv) and Li_2CO_3 (7.4 mg, 0.1 mmol, 1.0 equiv) at r.t. for 12 hours afforded compound **3l** in 35% yield as a pale yellow solid by chromatography on silica gel eluting with PE/EA (5:1→2:1). R_f (10% EA/PE) = 0.2. ^{1}H NMR (400 MHz, $CDCl_3$) δ 7.99 (s, 1H), 7.56 – 7.49 (m, 1H), 7.31 – 7.23 (m, 1H), 7.12 (dd, *J* = 13.4, 6.6 Hz, 1H), 7.05 (dd, *J* = 13.3, 6.6 Hz, 1H), 6.99 (s, 1H), 3.96 – 3.89 (m, 1H), 3.63 (d, *J* = 5.3 Hz, 3H), 3.40 (t, *J* = 5.7 Hz, 1H), 3.23 – 3.01 (m, 2H), 2.76 (s, 1H), 2.70 (s, 1H), 2.51 (s, 1H), 1.38 – 1.30 (m, 2H), 1.17 (s, 30H), 0.84 – 0.77 (m, 3H). ^{13}C NMR (100 MHz, $CDCl_3$) δ 175.4, 136.1, 127.4, 123.0, 122.3, 119.7, 118.7, 111.2, 110.7, 64.0, 52.4, 50.4, 32.0, 29.7, 29.7, 29.6, 29.6, 29.5, 29.4, 29.0, 27.0, 22.7, 14.2. IR (film) 3072, 3061, 1512, 1404, 1269, 742, 704. HRMS (ESI) Calcd for $C_{30}H_{51}N_3O_2S_2$ ($M+H^+$) 550.3495, found 550.3498.

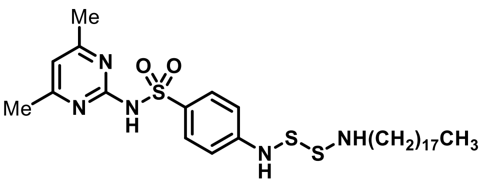

3m

The reaction of sulfamethazine (27.8 mg, 0.1 mmol, 1.0 equiv), $B(C_6F_5)_3$ (2.5 mg, 0.005 mmol, 5 mol%) and **1f** (29.0 mg, 0.105 mmol, 1.05 equiv) in 1,4-dioxane (0.25 mL) at r.t. for 24 hours, then octadecanamine (32.3 mg, 0.12 mmol, 1.2

equiv) and Li_2CO_3 (7.4 mg, 0.1 mmol, 1.0 equiv) at r.t. for 12 hours afforded compound **3m** in 40% yield as a white solid by chromatography on silica gel eluting with PE/EA (2:1→1:1). R_f (5% MeOH/DCM) = 0.3. ^{1}H NMR (400 MHz, DMSO-d$_6$) δ 8.36 (s, 1H), 7.84 (d, *J* = 8.5 Hz, 2H), 7.05 (d, *J* = 8.5 Hz, 2H), 6.75 (s, 1H), 4.88 (s, 1H), 2.79 (d, *J* = 5.1 Hz, 2H), 2.25 (s, 6H), 1.38 – 1.32 (m, 3H), 1.23 (s, 30H), 0.85 (t, *J* = 6.0 Hz, 3H). ^{13}C NMR (100 MHz, DMSO-d$_6$) δ 167.8, 157.0, 151.6, 130.8, 130.3, 114.6, 112.3, 51.1, 31.8, 29.5, 29.4, 29.3, 29.2, 28.9, 26.7, 23.5, 22.6, 14.4. IR (film) 3091, 3012, 1716, 1688, 1421, 1220, 1091, 992, 708, 686. HRMS (ESI) Calcd for $C_{30}H_{51}N_5O_2S_3$ (M+H$^+$) 610.3278, found 610.3274.

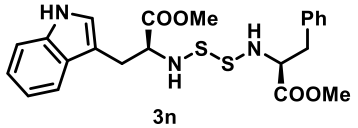

3n

The reaction of L-tryptophanate (21.8 mg, 0.1 mmol, 1.0 equiv), $B(C_6F_5)_3$ (2.5 mg, 0.005 mmol, 5 mol%) and **1f** (29.0 mg, 0.105 mmol, 1.05 equiv) in MeCN (0.5 mL) at r.t. for 12 hours, then methyl L-phenylalaninate (21.5 mg, 0.12 mmol, 1.2 equiv) and Li_2CO_3 (7.4 mg, 0.1 mmol, 1.0 equiv) at r.t. for 12 hours afforded compound **3n** in 42% yield as a white solid by chromatography on silica gel eluting with PE/EA (5:1). R_f (10% EA/PE) = 0.3. ^{1}H NMR (400 MHz, CDCl$_3$) δ 7.98 (s, 1H), 7.52 (d, *J* = 7.7 Hz, 1H), 7.25 (d, *J* = 7.7 Hz, 1H), 7.20 – 7.16 (m, 3H), 7.15 – 7.09 (m, 2H), 7.07 – 7.02 (m, 2H), 6.94 (s, 1H), 3.85 (dd, *J* = 13.6, 6.5 Hz, 1H), 3.70 – 3.63 (m, 1H), 3.59 (s, 3H), 3.57 (s, 3H), 3.49 (d, *J* = 6.8 Hz, 1H), 3.38 (d, *J* = 6.8 Hz, 1H), 3.09 (ddd, *J* = 21.9, 14.7, 6.5 Hz, 2H), 2.84 (ddd, *J* = 21.1, 13.8, 6.7 Hz, 2H). ^{13}C NMR (100 MHz, CDCl$_3$) δ 175.2, 175.0, 136.6, 136.1, 129.3, 128.4, 127.4, 126.9, 123.0, 122.2, 119.6, 118.8, 111.2, 110.6, 64.8, 63.8, 52.5, 52.4, 39.3, 29.1. IR (film) 3055, 2953, 1734, 1494, 1456, 1435, 1340, 1267, 1203, 1097, 742, 702. HRMS (ESI) Calcd for $C_{22}H_{25}N_3O_4S_2$ (M+H$^+$) 460.1359, found 460.1353.

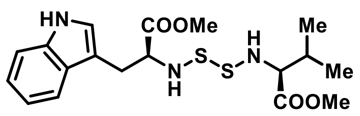

3o

The reaction of L-tryptophanate (21.8 mg, 0.1 mmol, 1.0 equiv), $B(C_6F_5)_3$ (2.5 mg, 0.005 mmol, 5 mol%) and **1f** (29.0 mg, 0.105 mmol, 1.05 equiv) in MeCN (0.5 mL) at r.t. for 12 hours, then methyl L-valinate (15.7 mg, 0.12 mmol, 1.2 equiv) and Li_2CO_3 (7.4 mg, 0.1 mmol, 1.0 equiv) at r.t. for 12 hours afforded compound **3o** in 54% yield as a white solid by chromatography on silica gel eluting with PE/EA (5:1). R_f (10% EA/PE) = 0.3. ^{1}H NMR (400 MHz, CDCl$_3$) δ 8.03 (s, 1H), 7.55 (d, *J* = 7.8 Hz, 1H), 7.27 (d, *J* = 8.0 Hz, 1H), 7.11 (t, *J* = 7.5 Hz, 1H), 7.05 (t, *J* = 7.4 Hz, 1H), 6.99 (s, 1H), 3.95 (dd, *J* = 13.3, 6.6 Hz, 1H), 3.63 (d, *J* = 2.6 Hz, 6H), 3.37 (d, *J* = 9.3 Hz, 1H), 3.12 (ddd, *J* = 22.1, 14.6, 6.6 Hz, 1H), 2.97 (s, 1H), 1.77 (td, *J* = 13.4, 6.7 Hz, 1H), 1.64 (s, 1H), 0.78 (t, *J* = 6.5 Hz, 6H). ^{13}C NMR (100 MHz, CDCl$_3$) δ 175.9, 175.4, 136.2, 127.4, 123.1, 122.2, 119.5, 118.8, 111.2, 110.7, 70.5, 63.7, 52.5, 52.3, 32.0, 29.3, 19.0, 18.2. IR (film) 3055, 1746,

1467, 1269, 1189, 1022, 740, 704. HRMS (ESI) Calcd for $C_{18}H_{25}N_3O_4S_2$ (M+H$^+$) 412.1359, found 412.1351.

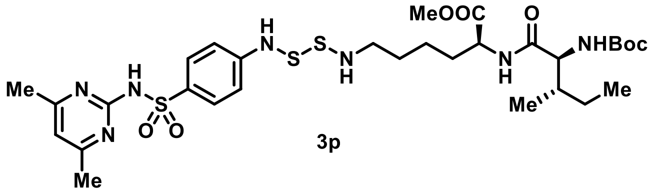

The reaction of sulfamethazine (27.8 mg, 0.1 mmol, 1.0 equiv), $B(C_6F_5)_3$ (2.5 mg, 0.005 mmol, 5 mol%) and **1f** (29.0 mg, 0.105 mmol, 1.05 equiv) in 1,4-dioxane (0.25 mL) at r.t. for 24 hours, then methyl (tert-butoxycarbonyl)-L-isoleucyl-L-lysinate (44.8 mg, 0.12 mmol, 1.2 equiv) and Li_2CO_3 (7.4 mg, 0.1 mmol, 1.0 equiv) at r.t. for 12 hours afforded compound **3p** in 65% yield as a white solid by chromatography on silica gel eluting with DCM/MeOH (100:1→50:1). R_f (10% MeOH/PE) = 0.2. ^{1}H NMR (400 MHz, $CDCl_3$) δ 7.89 (d, *J* = 8.8 Hz, 2H), 6.98 (d, *J* = 8.8 Hz, 2H), 6.76 (d, *J* = 7.4 Hz, 1H), 6.52 (s, 1H), 6.28 (s, 1H), 5.28 (d, *J* = 8.9 Hz, 1H), 4.51 (dd, *J* = 13.0, 7.8 Hz, 1H), 4.02 (t, *J* = 7.6 Hz, 1H), 3.65 (s, 3H), 3.02 (brs, 1H), 2.83 (t, *J* = 6.1 Hz, 2H), 2.27 (s, 6H), 1.84 – 1.68 (m, 2H), 1.63 – 1.54 (m, 1H), 1.51 – 1.40 (m, 3H), 1.35 (s, 9H), 1.29 – 1.17 (m, 2H), 1.15 – 1.01 (m, 2H), 0.89 (d, *J* = 6.7 Hz, 3H), 0.83 (t, *J* = 7.3 Hz, 3H). ^{13}C NMR (100 MHz, $CDCl_3$) δ 172.5, 172.1, 168.2, 156.3, 156.0, 151.1, 130.5, 114.9, 114.6, 113.3, 80.0, 59.2, 52.4, 52.1, 51.0, 37.3, 31.8, 28.7, 28.3, 24.8, 23.6, 22.5, 15.5, 11.3. IR (film) 3433, 3275, 2941, 1741, 1689, 1593, 1346, 1244, 1207, 1155, 1080, 866. HRMS (ESI) Calcd for $C_{30}H_{47}N_7O_7S_3$ (M+H$^+$) 714.2772, found 714.2764.

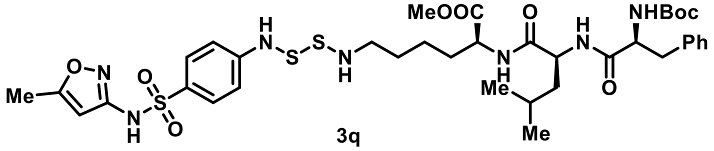

The reaction of sulfamethoxazole (25.3 mg, 0.1 mmol, 1.0 equiv), $B(C_6F_5)_3$ (2.5 mg, 0.005 mmol, 5 mol%) and **1f** (29.0 mg, 0.105 mmol, 1.05 equiv) in 1,4-dioxane (0.25 mL) at r.t. for 24 hours, then methyl (tert-butoxycarbonyl)-L-phenylalanyl-L-leucyl-L-lysinate (62.4 mg, 0.12 mmol, 1.2 equiv) and Li_2CO_3 (7.4 mg, 0.1 mmol, 1.0 equiv) at r.t. for 12 hours afforded compound **3q** in 45% yield as a white solid by chromatography on silica gel eluting with DCM/MeOH (100:1→50:1). R_f (10% MeOH/PE) = 0.2. ^{1}H NMR (400 MHz, $CDCl_3$) δ 7.63 (d, *J* = 8.7 Hz, 2H), 7.19 – 7.10 (m, 5H), 7.03 (s, 2H), 6.91 (d, *J* = 7.7 Hz, 2H), 6.20 (s, 1H), 6.12 (d, *J* = 3.9 Hz, 1H), 5.09 (d, *J* = 7.0 Hz, 1H), 4.55 – 4.41 (m, 2H), 4.32 (s, 1H), 3.66 (s, 3H), 3.09 (s, 1H), 2.95 – 2.65 (m, 4H), 2.26 (s, 3H), 1.82 – 1.70 (m, 1H), 1.58 – 1.49 (m, 3H), 1.46 – 1.34 (m, 3H), 1.26 (s, 9H), 1.22 – 1.14 (m, 3H), 0.83 – 0.76 (m, 6H). ^{13}C NMR (100 MHz, $CDCl_3$) δ 172.5, 172.2, 172.2, 170.5, 158.3, 155.5, 151.4, 136.5, 130.2, 129.4, 128.6, 128.5, 126.9, 115.6, 95.8, 80.3, 55.6, 52.4, 52.2, 52.0, 51.8, 40.8,

37.9, 31.7, 29.0, 28.2, 24.5, 22.7, 22.1, 12.6. IR (film) 3406, 3313, 3219, 2976, 1382, 1093, 1055, 883, 688. HRMS (ESI) Calcd for $C_{37}H_{53}N_7O_9S_3Na$ (M+Na$^+$) 858.2959, found 858.2952.

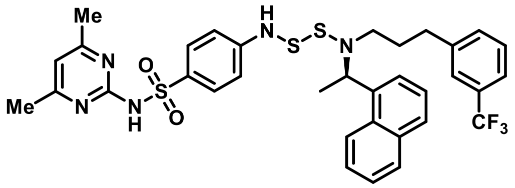

3r

The reaction of sulfamethazine (27.8 mg, 0.1 mmol, 1.0 equiv), $B(C_6F_5)_3$ (2.5 mg, 0.005 mmol, 5 mol%) and **1f** (29.0 mg, 0.105 mmol, 1.05 equiv) in 1,4-dioxane (0.25 mL) at r.t. for 24 hours, then cinacalcet (42.9 mg, 0.12 mmol, 1.2 equiv) and Li_2CO_3 (7.4 mg, 0.1 mmol, 1.0 equiv) at r.t. for 12 hours afforded compound **3r** in 74% yield as a yellow solid by chromatography on silica gel eluting with PE/EA (2:1). R_f (50% EA/PE) = 0.2. ^{1}H NMR (400 MHz, $CDCl_3$) δ 7.94 (d, J = 8.1 Hz, 2H), 7.90 (d, J = 8.3 Hz, 1H), 7.74 (d, J = 8.1 Hz, 1H), 7.64 (d, J = 7.9 Hz, 1H), 7.40 (dt, J = 14.7, 7.0 Hz, 3H), 7.33 – 7.22 (m, 3H), 7.10 (t, J = 7.6 Hz, 1H), 7.02 (s, 1H), 6.82 (d, J = 8.0 Hz, 3H), 6.42 (s, 1H), 5.34 (s, 1H), 4.66 (br, 1H), 2.72 – 2.50 (m, 2H), 2.32 – 2.25 (m, 2H), 2.22 (s, 6H), 2.08 – 1.95 (m, 2H), 1.49 (br, 3H). ^{19}F NMR (376 MHz, $CDCl_3$) δ -62.4. ^{13}C NMR (101 MHz, $CDCl_3$) δ 168.3, 156.3, 150.8, 142.6, 138.2, 134.0, 131.6, 131.4, 130.8, 130.6, 130.4 (q, $^2J_{CF}$ = 31.8 Hz), 128.9, 128.6, 128.3, 126.3, 125.7, 125.2, 124.7 (q, $^3J_{CF}$ = 3.6 Hz), 124.5, 124.2 (q, $^1J_{CF}$ = 272.5 Hz), 123.5, 122.6 (q, $^3J_{CF}$ = 3.8 Hz), 114.6, 63.2, 52.0, 32.2, 29.8, 28.7, 23.5. IR (film) 3319, 3055, 1593, 1552, 1438, 1153, 1074, 972, 868, 736, 671, 582. HRMS (ESI) Calcd for $C_{34}H_{35}F_3N_5O_2S_3$ (M+H$^+$) 698.1899, found 698.1881.

Gram scale synthesis of compound **3j**

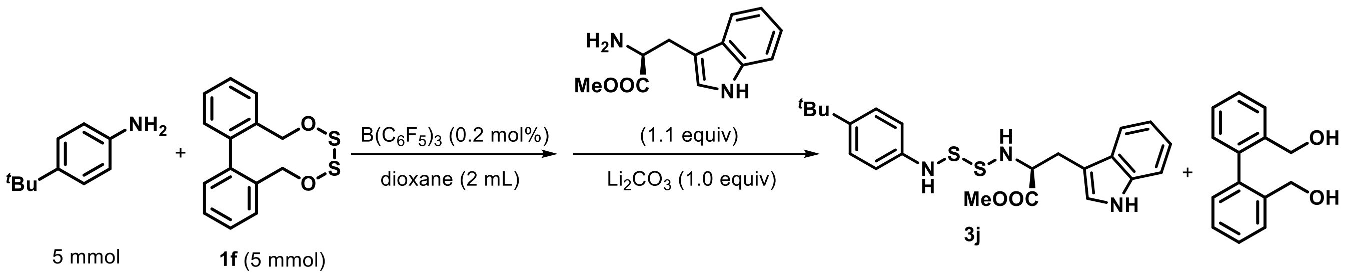

To a Schlenk tube were added 4-(tert-butyl)aniline (746.1 mg, 5 mmol, 1.0 equiv), $B(C_6F_5)_3$ (5.2 mg, 0.01 mmol, 0.2 mol%),**1f** (1.38 g, 5 mmol, 1.0 equiv), and 1,4-dioxane (5 mL), the mixture was stirred at r.t. for 8 hours.

After 4-tert-butylaniline was consumed, methyl L-tryptophanate (1.2 g, 5.5 mmol, 1.1 equiv), Li_2CO_3 (370 mg, 5 mmol, 1.0 equiv) and 1,4-dioxane (5 mL) were added to the mixture. The mixture was stirred at r.t. for 24 hours before it was concentrated under vacuum. Purification by

column chromatography afforded the desired product **3j** (1.4 g, 65%) with PE/EA (8:1→5:1) and recovery of [1,1'-biphenyl]-2,2'-diyldimethanol (940 mg, 88%) with PE/EA (2:1→1:1).

Synthesis of compound **4**

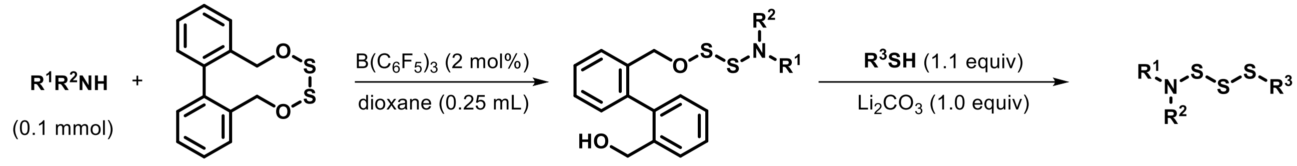

To a Schlenk tube were added amine (0.1 mmol, 1.0 equiv), $B(C_6F_5)_3$ (1.0 mg, 0.002 mmol, 2 mol%), **1f** (29.0 mg, 0.105 mmol, 1.05 equiv), and 1,4-dioxane (0.25 mL), the mixture was stirred at r.t. for 4 hours to obtain **2**.

After amine was consumed, thiol (0.11 mmol, 1.1 equiv) and Li_2CO_3 (7.4 mg, 0.1 mmol, 1.0 equiv) were added to the mixture. The mixture was stirred at r.t. for 8 hours before it was concentrated under vacuum. Purification by column chromatography afforded the desired product **4**.

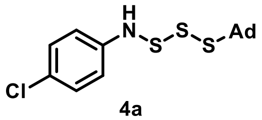

4a

The reaction of 4-chloroaniline (12.7 mg, 0.1 mmol, 1.0 equiv), $B(C_6F_5)_3$ (0.5 mg, 0.001 mmol, 1 mol%) and **1f** (29.0 mg, 0.105 mmol, 1.05 equiv) in 1,4-dioxane (0.25 mL) at r.t. for 4 hours, then 1-adamantylthiol (20.1 mg, 0.12 mmol, 1.2 equiv) and Li_2CO_3 (7.4 mg, 0.1 mmol, 1.0 equiv) at r.t. for 8 hours afforded compound **4a** in 93% yield as a white solid by chromatography on silica gel eluting with PE/DCM (5:1). R_f (5% EA/PE) = 0.3. ^{1}H NMR (400 MHz, CD_3CN) δ 7.23 – 7.10 (m, 2H), 7.07 – 6.97 (m, 2H), 6.61 (s, 1H), 1.97 (s, 3H), 1.77 (d, J = 2.6 Hz, 6H), 1.60 (q, J = 12.4 Hz, 6H). ^{13}C NMR (100 MHz, CD_3CN) δ 144.9, 129.6, 126.2, 118.5, 117.8, 50.3, 43.1, 36.2, 30.5. IR (film) 2906, 1591, 1487, 1228, 1093, 891, 819, 659. HRMS (EI) Calcd for $C_{16}H_{20}ClNS_3$ 357.0446, found 357.0437.

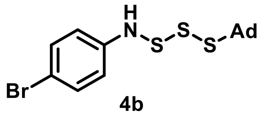

4b

The reaction of 4-bromoaniline (17.2 mg, 0.1 mmol, 1.0 equiv), $B(C_6F_5)_3$ (0.5 mg, 0.001 mmol, 1 mol%) and **1f** (29.0 mg, 0.105 mmol, 1.05 equiv) in 1,4-dioxane (0.25 mL) at r.t. for 4 hours, then 1-adamantylthiol (20.1 mg, 0.12 mmol, 1.2 equiv) and Li_2CO_3 (7.4 mg, 0.1 mmol, 1.0 equiv) at r.t. for 8 hours afforded compound **4b** in 98% yield as a white solid by chromatography on silica gel eluting with PE/DCM (10:1). R_f (5% EA/PE) = 0.3. ^{1}H NMR (400 MHz, Acetone-d_6) δ 7.50 (s, 1H), 7.28 (d, J = 8.8 Hz, 2H), 7.04 (d, J = 8.8 Hz, 2H), 1.93 (s, 3H), 1.74 (s, 6H), 1.56 (q, J = 12.3 Hz, 6H). ^{13}C NMR (100 MHz, Acetone-d_6) δ 145.1, 131.9, 118.4, 112.7, 49.6, 42.6, 35.8, 29.9. IR (film) 3360, 2903,

2849, 1587, 1483, 1296, 1273, 1228, 1037, 1003, 895, 818, 684. HRMS (EI) Calcd for $C_{16}H_{20}BrNS_3$ 400.9941, found 400.9945.

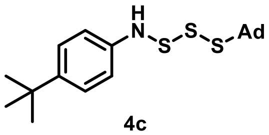

4c

The reaction of 4-(tert-butyl)aniline (14.9 mg, 0.1 mmol, 1.0 equiv), $B(C_6F_5)_3$ (0.5 mg, 0.001 mmol, 1 mol%) and **1f** (29.0 mg, 0.105 mmol, 1.05 equiv) in 1,4-dioxane (0.25 mL) at r.t. for 4 hours, then 1-adamantylthiol (20.1 mg, 0.12 mmol, 1.2 equiv) and Li_2CO_3 (7.4 mg, 0.1 mmol, 1.0 equiv) at r.t. for 8 hours afforded compound **4c** in 75% yield as a white solid by chromatography on silica gel eluting with PE/EA (20:1). R_f (5% EA/PE) = 0.3. ^{1}H NMR (400 MHz, Acetone-d_6) δ 7.24 (s, 1H), 7.20 – 7.16 (m, 2H), 7.03 – 6.98 (m, 2H), 1.95 – 1.89 (m, 3H), 1.74 (d, *J* = 2.7 Hz, 6H), 1.55 (q, *J* = 12.3 Hz, 6H), 1.16 (s, 9H). ^{13}C NMR (100 MHz, Acetone-d_6) δ 143.8, 142.9, 125.8, 116.27, 49.4, 42.6, 35.8, 33.8, 31.0, 29.9. IR (film) 3344, 2960, 2903, 2848, 1510, 1450, 1296, 1284, 1234, 1184, 1039, 904, 825. HRMS (EI) Calcd for $C_{20}H_{29}NS_3$ 379.1462, found 379.1464.

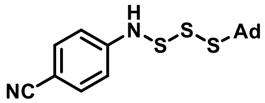

4d

The reaction of 4-aminobenzonitrile (11.8 mg, 0.1 mmol, 1.0 equiv), $B(C_6F_5)_3$ (0.5 mg, 0.001 mmol, 1 mol%) and **1f** (29.0 mg, 0.105 mmol, 1.05 equiv) in 1,4-dioxane (0.25 mL) at r.t. for 4 hours, then 1-adamantylthiol (20.1 mg, 0.12 mmol, 1.2 equiv) and Li_2CO_3 (7.4 mg, 0.1 mmol, 1.0 equiv) at r.t. for 8 hours afforded compound **4d** in 86% yield as a white solid by chromatography on silica gel eluting with PE/EA (5:1). R_f (20% EA/PE) = 0.3. ^{1}H NMR (400 MHz, Acetone-d_6) δ 8.02 (s, 1H), 7.58 – 7.48 (m, 2H), 7.27 – 7.19 (m, 2H), 1.94 (s, 3H), 1.75 (d, *J* = 2.6 Hz, 6H), 1.56 (q, *J* = 12.3 Hz, 6H). ^{13}C NMR (100 MHz, Acetone-d_6) δ 150.0, 133.4, 118.9, 116.7, 103.5, 49.8, 42.6, 35.7, 29.9. IR (film) 3361, 2904, 2212, 1624, 1448, 1309, 1170, 1045, 831, 738, 688. HRMS (EI) Calcd for $C_{17}H_{20}N_2S_3$ 348.0789, found 348.0788.

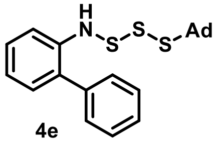

4e

The reaction of [1,1'-biphenyl]-2-amine (16.9 mg, 0.1 mmol, 1.0 equiv), $B(C_6F_5)_3$ (0.5 mg, 0.001 mmol, 1 mol%) and **1f** (29.0 mg, 0.105 mmol, 1.05 equiv) in 1,4-dioxane (0.25 mL) at r.t. for 4 hours, then 1-adamantylthiol (20.1 mg, 0.12 mmol, 1.2 equiv) and Li_2CO_3 (7.4 mg, 0.1 mmol, 1.0 equiv) at r.t. for 8 hours afforded compound **4e** in 84% yield as a white solid by chromatography on silica gel eluting with PE/DCM (20:1). R_f (5% EA/PE) = 0.4. ^{1}H NMR (400 MHz, Acetone-d_6) δ 7.50 (d, *J* = 8.2 Hz, 1H), 7.39 – 7.30 (m, 2H), 7.29 – 7.18 (m, 4H), 7.01 (d, *J* = 7.5 Hz, 1H), 6.89 (t, *J* = 7.4 Hz, 1H), 6.34 (s, 1H), 1.90 (s, 3H), 1.68 (s, 6H), 1.54 (q, *J* = 12.3 Hz, 6H). ^{13}C NMR (100 MHz, Acetone-d_6) δ 141.8, 138.4, 131.3, 130.5, 129.2, 129.0, 128.4, 127.6, 121.6, 116.6, 49.5, 42.6, 35.8, 29.9. IR (film)

3379, 2903, 2848, 1499, 1477, 1296, 1261, 1207, 1039, 896, 750. HRMS (EI) Calcd for $C_{22}H_{25}NS_3$ 399.1149, found 399.1151.

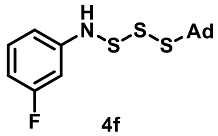

4f

The reaction of 3-fluoroaniline (11.1 mg, 0.1 mmol, 1.0 equiv), $B(C_6F_5)_3$ (0.5 mg, 0.001 mmol, 1 mol%) and **1f** (29.0 mg, 0.105 mmol, 1.05 equiv) in 1,4-dioxane (0.25 mL) at r.t. for 4 hours, then 1-adamantylthiol (20.1 mg, 0.12 mmol, 1.2 equiv) and Li_2CO_3 (7.4 mg, 0.1 mmol, 1.0 equiv) at r.t. for 8 hours afforded compound **4f** in 77% yield as a light yellow oil by chromatography on silica gel eluting with PE/EA (20:1). R_f (5% EA/PE) = 0.3.^{1}H NMR (400 MHz, Acetone-d$_6$) δ 7.61 (s, 1H), 7.14 (dd, J = 15.0, 7.7 Hz, 1H), 6.93 – 6.82 (m, 2H), 6.53 (ddd, J = 8.6, 2.6, 1.2 Hz, 1H), 1.94 (s, 3H), 1.75 (d, J = 2.7 Hz, 6H), 1.57 (q, J = 12.3 Hz, 6H). ^{19}F NMR (376 MHz, Acetone-d$_6$) δ -113.8. ^{13}C NMR (100 MHz, Acetone-d$_6$) δ 163.7 (d, $^1J_{CF}$ = 242.5 Hz), 147.9 (d, $^3J_{CF}$ = 10.2 Hz), 130.6 (d, $^3J_{CF}$ = 9.8 Hz), 112.5 (d, $^4J_{CF}$ = 2.5 Hz), 107.4 (d, $^2J_{CF}$ = 21.6 Hz), 103.2 (d, $^2J_{CF}$ = 26.0 Hz), 49.6, 42.6, 35.7, 29.9. IR (film) 3199, 2905, 1612, 1487, 1273, 1165, 1139, 1001, 968, 765, 681. HRMS (EI) Calcd for $C_{16}H_{20}FNS_3$ 341.0742, found 341.0744.

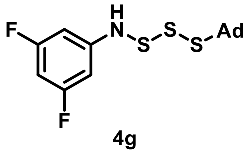

4g

The reaction of 3,5-difluoroaniline (12.9 mg, 0.1 mmol, 1.0 equiv), $B(C_6F_5)_3$ (0.5 mg, 0.001 mmol, 1 mol%) and **1f** (29.0 mg, 0.105 mmol, 1.05 equiv) in 1,4-dioxane (0.25 mL) at r.t. for 4 hours, then 1-adamantylthiol (20.1 mg, 0.12 mmol, 1.2 equiv) and Li_2CO_3 (7.4 mg, 0.1 mmol, 1.0 equiv) at r.t. for 8 hours afforded compound **4g** in 70% yield as a colorless oil by chromatography on silica gel eluting with PE/EA (20:1). R_f (10% EA/PE) = 0.4. ^{1}H NMR (400 MHz, Acetone-d$_6$) δ 7.85 (s, 1H), 6.76 – 6.61 (m, 2H), 6.39 (ddd, J = 9.2, 5.7, 2.3 Hz, 1H), 1.95 (s, 3H), 1.76 (d, J = 2.7 Hz, 6H), 1.58 (q, J = 12.4 Hz, 6H). ^{19}F NMR (282 MHz, Acetone-d$_6$) δ -105.5, -105.6. ^{13}C NMR (100 MHz, Acetone-d$_6$) δ 163.8 (dd, $^1J_{CF}$ = 244.3, $^3J_{CF}$ = 15.4 Hz), 149.2 (t, $^3J_{CF}$ = 12.7 Hz), 99.4 (d, $^2J_{CF}$ = 29.2 Hz), 95.7 (t, $^2J_{CF}$ = 26.4 Hz), 49.8, 42.6, 35.7, 29.9. IR (film) 3362, 2904, 2849, 1620, 1597, 1485, 1467, 1342, 1296, 1139, 1112, 1016, 993, 827, 671. HRMS (EI) Calcd for $C_{16}H_{19}F_2NS_3$ 359.0648, found 359.0649.

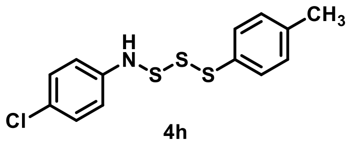

4h

The reaction of 4-chloroaniline (12.7 mg, 0.1 mmol, 1.0 equiv), $B(C_6F_5)_3$ (0.5 mg, 0.001 mmol, 1 mol%) and **1f** (29.0 mg, 0.105 mmol, 1.05 equiv) in 1,4-dioxane (0.25 mL) at r.t. for 4 hours, then 4-methylbenzenethiol (14.9 mg, 0.12 mmol, 1.2 equiv) and Li_2CO_3 (7.4 mg, 0.1 mmol, 1.0 equiv) at r.t. for 8 hours afforded compound **4h** in 92% yield as a yellow solid by chromatography on silica gel eluting with PE/DCM (20:1). R_f (5% EA/PE) = 0.3. ^{1}H NMR (400 MHz, Acetone-d$_6$) δ

7.54 (s, 1H), 7.25 (d, *J* = 8.2 Hz, 2H), 7.12 – 7.04 (m, 2H), 7.04 – 6.95 (m, 4H), 2.18 (s, 3H). ^{13}C NMR (100 MHz, Acetone-d$_6$) δ 145.2, 139.0, 134.2, 130.6, 130.4, 129.6, 126.4, 118.8, 20.9. IR (film) 3354, 2918, 2852, 1591, 1487, 1435, 1276, 1224, 1170, 1090, 817, 800. HRMS (EI) Calcd for $C_{13}H_{12}ClNS_3$ 312.9820, found 312.9821.

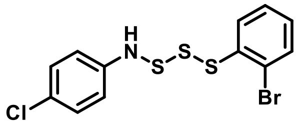
4i

The reaction of 4-chloroaniline (12.7 mg, 0.1 mmol, 1.0 equiv), $B(C_6F_5)_3$ (0.5 mg, 0.001 mmol, 1 mol%) and **1f** (29.0 mg, 0.105 mmol, 1.05 equiv) in 1,4-dioxane (0.25 mL) at r.t. for 4 hours, then 2-bromobenzenethiol (22.7 mg, 0.12 mmol, 1.2 equiv) and Li_2CO_3 (7.4 mg, 0.1 mmol, 1.0 equiv) at r.t. for 8 hours afforded compound **4i** in 77% yield as a colorless oil by chromatography on silica gel eluting with PE/DCM (15:1). R$_f$ (5% EA/PE) = 0.4. ^{1}H NMR (400 MHz, Acetone-d$_6$) δ 7.68 (s, 1H), 7.48 (dd, *J* = 13.5, 7.9 Hz, 2H), 7.12 (dd, *J* = 14.3, 6.6 Hz, 2H), 7.08 – 7.01 (m, 4H). ^{13}C NMR (100 MHz, Acetone-d$_6$) δ 145.1, 138.0, 133.8, 129.9, 129.5, 129.2, 129.1, 126.8, 122.2, 118.9. IR (film) 3414, 2953, 2922, 2851, 1651, 1462, 1377, 1080, 746, 543. HRMS (EI) Calcd for $C_{12}H_9BrClNS_3$ 376.8769, found 376.8772.

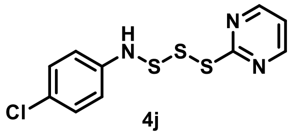
4j

The reaction of 4-chloroaniline (12.7 mg, 0.1 mmol, 1.0 equiv), $B(C_6F_5)_3$ (0.5 mg, 0.001 mmol, 1 mol%) and **1f** (29.0 mg, 0.105 mmol, 1.05 equiv) in 1,4-dioxane (0.25 mL) at r.t. for 4 hours, then pyrimidine-2-thiol (13.4 mg, 0.12 mmol, 1.2 equiv) and Li_2CO_3 (7.4 mg, 0.1 mmol, 1.0 equiv) at r.t. for 8 hours afforded compound **4j** in 66% yield as a white solid by chromatography on silica gel eluting with PE/DCM (2:1). R$_f$ (20% EA/PE) = 0.3. ^{1}H NMR (300 MHz, Acetone-d$_6$) δ 8.58 (d, *J* = 4.8 Hz, 2H), 7.72 (s, 1H), 7.26 (t, *J* = 4.8 Hz, 1H), 7.22 – 7.13 (m, 4H). ^{13}C NMR (100 MHz, Acetone-d$_6$) δ 169.8, 158.0, 144.9, 128.8, 125.6, 118.6, 118.1. IR (film) 2976, 1593, 1553, 1487, 1377, 1169, 1092, 903, 824, 770 742, 629. HRMS (EI) Calcd for $C_{10}H_8ClN_3S_3$ 300.9569, found 300.9572.

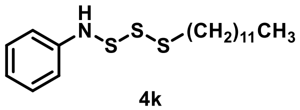
4k

The reaction of aniline (9.3 mg, 0.1 mmol, 1.0 equiv), $B(C_6F_5)_3$ (0.5 mg, 0.001 mmol, 1 mol%) and **1f** (29.0 mg, 0.105 mmol, 1.05 equiv) in 1,4-dioxane (0.25 mL) at r.t. for 4 hours, then 1-dodecanethiol (24.2 mg, 0.12 mmol, 1.2 equiv) and Li_2CO_3 (7.4 mg, 0.1 mmol, 1.0 equiv) at r.t. for 8 hours afforded compound **4k** in 81% yield as a light yellow oil by chromatography on silica gel eluting with PE/EA (100:1). R$_f$ (2% EA/PE) = 0.5. ^{1}H NMR (300 MHz, CD_3CN) δ 7.30 (dd, *J* = 8.5, 7.4 Hz, 2H), 7.16 (dd, *J* = 8.6, 1.0 Hz, 2H), 6.96 (t, *J* = 7.3 Hz, 1H), 6.59 (s, 1H), 2.81 – 2.62 (m, 2H), 1.63 (dt, *J* = 14.7, 7.2 Hz, 2H), 1.30 (s, 18H), 0.91 (t, *J* = 6.7 Hz, 3H). ^{13}C NMR (100 MHz, CD_3CN) δ 146.1, 129.8, 122.0, 117.2, 40.0, 32.7, 30.0, 29.9, 29.8, 29.7, 29.4, 29.3, 28.6, 23.0, 14.0. IR (film) 3352, 2976,

2924, 2852, 1598, 1492, 1468, 1282, 1229, 1093, 1051, 885, 750, 690. HRMS (EI) Calcd for $C_{18}H_{31}NS_3$ 357.1619, found 357.1622.

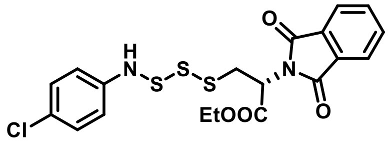

4l

The reaction of 4-chloroaniline (12.7 mg, 0.1 mmol, 1.0 equiv), $B(C_6F_5)_3$ (0.5 mg, 0.001 mmol, 1 mol%) and **1f** (29.0 mg, 0.105 mmol, 1.05 equiv) in 1,4-dioxane (0.25 mL) at r.t. for 4 hours, then ethyl (R)-2-(1,3-dioxoisoindolin-2-yl)-3-mercaptopropanoate (33.5 mg, 0.12 mmol, 1.2 equiv) and Li_2CO_3 (7.4 mg, 0.1 mmol, 1.0 equiv) at r.t. for 8 hours afforded compound **4l** in 80% yield as a light yellow oil by chromatography on silica gel eluting with PE/EA (5:1). R_f (20% EA/PE) = 0.3. ^{1}H NMR (400 MHz, Acetone-d$_6$) δ 7.78 (s, 4H), 7.58 (s, 1H), 7.11 (d, *J* = 8.8 Hz, 2H), 7.06 – 7.02 (m, 2H), 5.05 (dd, *J* = 10.2, 5.1 Hz, 1H), 4.05 (q, *J* = 7.1 Hz, 2H), 3.51 (qd, *J* = 14.5, 7.6 Hz, 2H), 1.05 (t, *J* = 7.1 Hz, 3H). ^{13}C NMR (100 MHz, Acetone-d$_6$) δ 167.6, 167.2, 144.4, 134.9, 131.6, 129.0, 125.7, 123.5, 118.0, 61.9, 51.4, 38.1, 13.5. IR (film) 3329, 2980, 1776, 1745, 1715, 1489, 1387, 1232, 1094, 1022, 875, 824, 721. HRMS (EI) Calcd for $C_{19}H_{17}ClN_2O_4S_3$ 468.0039, found 468.0037.

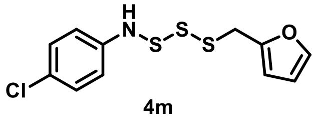

4m

The reaction of 4-chloroaniline (12.7 mg, 0.1 mmol, 1.0 equiv), $B(C_6F_5)_3$ (0.5 mg, 0.001 mmol, 1 mol%) and **1f** (29.0 mg, 0.105 mmol, 1.05 equiv) in 1,4-dioxane (0.25 mL) at r.t. for 4 hours, then furan-2-ylmethanethiol (13.7 mg, 0.12 mmol, 1.2 equiv) and Li_2CO_3 (7.4 mg, 0.1 mmol, 1.0 equiv) at r.t. for 8 hours afforded compound **4m** in 67% yield as a colorless oil by chromatography on silica gel eluting with PE/EA (20:1). R_f (5% EA/PE) = 0.3. ^{1}H NMR (400 MHz, Acetone-d$_6$) δ 7.47 (s, 1H), 7.39 (dd, *J* = 1.8, 0.8 Hz, 1H), 7.20 – 7.14 (m, 2H), 7.11 – 7.05 (m, 2H), 6.23 (dd, *J* = 3.2, 1.9 Hz, 1H), 6.10 (dd, *J* = 3.2, 0.6 Hz, 1H), 3.92 (s, 2H). ^{13}C NMR (100 MHz, Acetone-d$_6$) δ 149.7, 144.8, 143.0, 129.0, 125.6, 118.0, 110.6, 109.3, 35.9. IR (film) 3354, 1593, 1487, 1436, 1274, 1228, 1172, 1149, 1091, 1010, 935, 822 738. HRMS (EI) Calcd for $C_{11}H_{10}ClNOS_3$ 302.9613, found 302.9616.

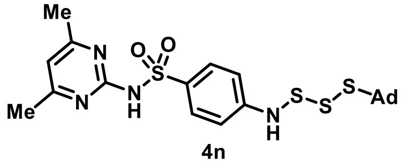

4n

The reaction of sulfamethazine (27.8 mg, 0.1 mmol, 1.0 equiv), $B(C_6F_5)_3$ (2.5 mg, 0.005 mmol, 5 mol%) and **1f** (29.0 mg, 0.105 mmol, 1.05 equiv) in 1,4-dioxane (0.25 mL) at r.t. for 24 hours, then 1-adamantylthiol (20.1 mg, 0.12 mmol, 1.2 equiv) and Li_2CO_3 (7.4 mg, 0.1 mmol, 1.0 equiv) at r.t. for 8 hours afforded compound **4n** in 74% yield as a white solid by chromatography on silica gel eluting with PE/EA (2:1→1:1). R_f (5% MeOH/DCM) = 0.4. ^{1}H NMR (400 MHz, Acetone-d$_6$) δ 7.93 (d, *J* = 8.7 Hz, 3H), 7.21 (d, *J* = 8.9 Hz, 2H), 6.60 (s, 1H),

2.16 (s, 6H), 1.87 (s, 3H), 1.76 – 1.66 (m, 6H), 1.96 (s, 1H), 1.49 (dd, *J* = 28.1, 11.9 Hz, 6H). ^{13}C NMR (100 MHz, Acetone-d$_6$) δ 168.0, 156.8, 150.0, 132.0, 130.6, 115.3, 114.3, 49.8, 42.6, 35.7, 29.9, 22.8. IR (film) 3327, 3267, 1595, 1433, 1383, 1153, 1092, 1053, 883, 582. HRMS (ESI) Calcd for $C_{22}H_{29}N_4O_2S_4$ (M+H$^+$) 509.1168, found 509.1168.

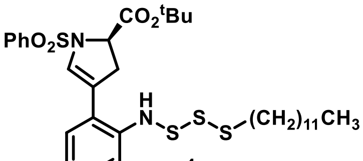

4o

The reaction of tert-butyl (R)-4-(2-aminophenyl)-1-(phenylsulfonyl)-2,3-dihydro-1H-pyrrole-2-carboxylate (40.0 mg, 0.1 mmol, 1.0 equiv), $B(C_6F_5)_3$ (2.5 mg, 0.005 mmol, 5 mol%) and **1f** (29.0 mg, 0.105 mmol, 1.05 equiv) in 1,4-dioxane (0.25 mL) at r.t. for 12 hours, then 1-dodecanethiol (24.2 mg, 0.12 mmol, 1.2 equiv) and Li_2CO_3 (7.4 mg, 0.1 mmol, 1.0 equiv) at r.t. for 8 hours afforded compound **4o** in 42% yield as a yellow solid by chromatography on silica gel eluting with PE/EA (10:1→5:1). R_f (20% EA/PE) = 0.3. ^{1}H NMR (400 MHz, Acetone-d$_6$) δ 7.83 – 7.79 (m, 2H), 7.63 (t, *J* = 7.3 Hz, 1H), 7.57 (t, *J* = 7.4 Hz, 2H), 7.32 (t, *J* = 6.3 Hz, 1H), 7.14 (t, *J* = 7.7 Hz, 1H), 6.98 (d, *J* = 7.6 Hz, 1H), 6.82 (t, *J* = 7.6 Hz, 1H), 6.63 – 6.56 (m, 1H), 6.44 (s, 1H), 4.23 (dd, *J* = 10.9, 7.3 Hz, 1H), 3.05 (ddd, *J* = 16.0, 11.0, 1.9 Hz, 1H), 2.78 (ddd, *J* = 16.2, 7.3, 1.7 Hz, 1H), 2.55 – 2.50 (m, 2H), 1.50 – 1.41 (m, 2H), 1.39 (s, 9H), 1.15 (s, 18H), 0.75 (t, *J* = 6.6 Hz, 3H). ^{13}C NMR (100 MHz, Acetone-d$_6$) δ 169.5, 143.0, 136.9, 133.6, 129.5, 129.1, 128.4, 128.0, 128.0, 127.6, 124.0, 122.2, 120.5, 118.6, 81.5, 61.4, 39.4, 37.8, 31.8, 29.5, 29.4, 28.7, 28.2, 27.3, 22.5, 13.5. IR (film) 2953, 2924, 2852, 1736, 1487, 1446, 1367, 1309, 1171, 1092, 752, 721, 690, 605, 572. HRMS (ESI) Calcd for $C_{33}H_{49}N_2O_4S_4$ (M+H$^+$) 665.2570, found 665.2551.

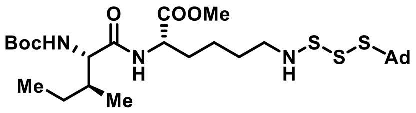

4p

The reaction of methyl (tert-butoxycarbonyl)-L-isoleucyl-L-lysinate (37.3 mg, 0.1 mmol, 1.0 equiv), $B(C_6F_5)_3$ (1.0 mg, 0.002 mmol, 2 mol%) and **1f** (29.0 mg, 0.105 mmol, 1.05 equiv) in 1,4-dioxane (0.25 mL) at r.t. for 4 hours, then 1-adamantylthiol (20.1 mg, 0.12 mmol, 1.2 equiv) and Li_2CO_3 (7.4 mg, 0.1 mmol, 1.0 equiv) at r.t. for 8 hours afforded compound **4p** in 40% yield as a white solid by chromatography on silica gel eluting with DCM/MeOH (100:1). R_f (1% MeOH/DCM) = 0.2. ^{1}H NMR (400 MHz, $CDCl_3$) δ 6.35 (d, *J* = 7.5 Hz, 1H), 4.98 (d, *J* = 8.3 Hz, 1H), 4.53 (td, *J* = 7.7, 5.4 Hz, 1H), 3.93 – 3.85 (m, 1H), 3.68 (s, 3H), 3.24 (t, *J* = 5.1 Hz, 1H), 2.95 (dd, *J* = 12.5, 6.6 Hz, 2H), 2.03 (s, 3H), 1.87 – 1.76 (m, 8H), 1.72 (s, 1H), 1.67 – 1.58 (m, 6H), 1.51 (dt, *J* = 14.2, 7.2 Hz, 2H), 1.38 (s, 9H), 1.35 – 1.28 (m, 2H), 1.27 – 1.17 (m, 2H), 0.89 – 0.83 (m, 6H). IR (film) 3319, 2970, 2910, 2854, 1743, 1658, 1520, 1452, 1369, 1292, 1246, 1167, 1088, 1045, 977, 877. ^{13}C NMR (100 MHz, $CDCl_3$) δ 172.6, 171.4, 155.8, 80.0, 59.3, 52.4, 52.0, 50.3, 49.9,

42.7, 37.1, 36.1, 32.2, 29.9, 28.6, 28.3, 24.8, 22.5, 15.5, 11.4. IR (film) 3319, 2970, 2910, 2854, 1743, 1658, 1452, 1369, 1292, 1246, 1209, 1087, 1045, 877. HRMS (ESI) Calcd for $C_{28}H_{50}N_3O_5S_3$ (M+H$^+$) 604.2907, found 604.2902.

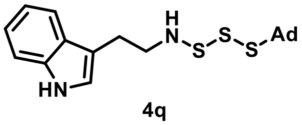

4q

The reaction of tryptamine (16.0 mg, 0.1 mmol, 1.0 equiv), $B(C_6F_5)_3$ (0.5 mg, 0.001 mmol, 1 mol%) and **1f** (29.0 mg, 0.105 mmol, 1.05 equiv) in 1,4-dioxane (0.25 mL) at r.t. for 4 hours, then 1-adamantylthiol (20.1 mg, 0.12 mmol, 1.2 equiv) and Li_2CO_3 (7.4 mg, 0.1 mmol, 1.0 equiv) at r.t. for 8 hours afforded compound **4q** in 52% yield as a colorless oil by chromatography on silica gel eluting with PE/EA (2:1). R_f (20% EA/PE) = 0.2. ^{1}H NMR (400 MHz, Acetone-d$_6$) δ 9.89 (s, 1H), 7.48 (d, *J* = 7.8 Hz, 1H), 7.24 (d, *J* = 8.0 Hz, 1H), 7.05 (d, *J* = 1.8 Hz, 1H), 6.96 (t, *J* = 7.5 Hz, 1H), 6.89 (t, *J* = 7.4 Hz, 1H), 4.42 (s, 1H), 3.25 (td, *J* = 7.3, 5.5 Hz, 2H), 2.94 (t, *J* = 7.4 Hz, 2H), 1.94 – 1.88 (m, 3H), 1.73 (d, *J* = 2.3 Hz, 6H), 1.61 – 1.48 (m, 6H). ^{13}C NMR (100 MHz, Acetone-d$_6$) δ 136.8, 127.7, 122.6, 121.2, 118.6, 118.4, 112.4, 111.3, 51.8, 49.1, 42.6, 35.8, 29.9, 25.2. IR (film) 3410, 2903, 2849, 1456, 1340, 1298, 1078, 1039, 740, 582. HRMS (ESI) Calcd for $C_{20}H_{27}N_2S_3$ (M+H$^+$) 391.1331, found 391.1328.

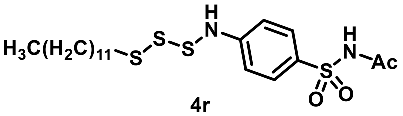

4r

The reaction of sulfacetamide (21.4 mg, 0.1 mmol, 1.0 equiv), $B(C_6F_5)_3$ (2.5 mg, 0.005 mmol, 5 mol%) and **1f** (29.0 mg, 0.105 mmol, 1.05 equiv) in 1,4-dioxane (0.25 mL) at r.t. for 24 hours, then 1-dodecanethiol (24.2 mg, 0.12 mmol, 1.2 equiv) and Li_2CO_3 (7.4 mg, 0.1 mmol, 1.0 equiv) at r.t. for 8 hours afforded compound **4r** in 69% yield as a white solid by chromatography on silica gel eluting with PE/EA (2:1→1:1). R_f (5% MeOH/DCM) = 0.4. ^{1}H NMR (400 MHz, $CDCl_3$) δ 8.84 (s, 1H), 7.87 (d, *J* = 8.8 Hz, 2H), 7.14 (d, *J* = 8.8 Hz, 2H), 5.99 (s, 1H), 2.75 (t, *J* = 7.3 Hz, 2H), 2.00 (s, 3H), 1.62 (dt, *J* = 14.8, 7.3 Hz, 2H), 1.32 – 1.25 (m, 2H), 1.19 (s, 16H), 0.81 (t, *J* = 6.8 Hz, 3H). ^{13}C NMR (100 MHz, Acetone-d$_6$) δ 173.2, 162.0, 155.4, 137.5, 135.8, 120.5, 119.5, 72.0, 44.7, 37.0, 34.7, 34.7, 34.6, 34.5, 34.1, 34.0, 33.3, 28.0, 27.7, 18.7. IR (film) 3244, 2922, 2850, 1697, 1589, 1450, 1232, 1151, 910, 736, 680. HRMS (ESI) Calcd for $C_{20}H_{35}N_2O_3S_4$ (M+H$^+$) 479.1525, found 479.1517.

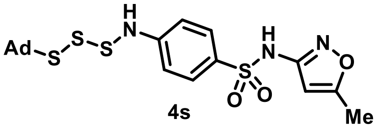

4s

The reaction of sulfamethoxazole (25.3 mg, 0.1 mmol, 1.0 equiv), $B(C_6F_5)_3$ (2.5 mg, 0.005 mmol, 5 mol%) and **1f** (29.0 mg, 0.105 mmol, 1.05 equiv) in 1,4-dioxane (0.25 mL) at r.t. for 24 hours, then 1-adamantylthiol (20.1 mg, 0.12 mmol, 1.2 equiv) and Li_2CO_3 (7.4 mg, 0.1 mmol, 1.0 equiv) at r.t. for 8 hours afforded compound **4s** in 65% yield as a white solid by chromatography on silica

gel eluting with DCM/MeOH (100:1). R_f (5% MeOH/DCM) = 0.4. ^{1}H NMR (400 MHz, Acetone-d_6) δ 9.72 (s, 1H), 8.01 (s, 1H), 7.84 – 7.55 (m, 2H), 7.37 – 7.10 (m, 2H), 6.10 (s, 1H), 2.19 (s, 3H), 1.93 – 1.90 (m, 3H), 1.73 (s, 6H), 1.54 (dd, *J* = 25.4, 12.1 Hz, 6H). ^{13}C NMR (100 MHz, Acetone-d_6) δ 170.4, 157.9, 150.4, 131.4, 128.9, 128.8, 116.1, 95.3, 49.8, 42.6, 35.7, 29.9, 11.6. IR (film) 3053, 2949, 1591, 1275, 1157, 1072, 1008, 862, 742, 702, 586. HRMS (ESI) Calcd for $C_{20}H_{26}N_3O_3S_4$ (M+H$^+$) 484.0852, found 484.0854.

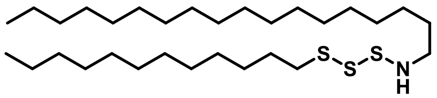

4t

The reaction of octadecanamine (26.9 mg, 0.1 mmol, 1.0 equiv), $B(C_6F_5)_3$ (1.0 mg, 0.002 mmol, 2 mol%) and **1f** (29.0 mg, 0.105 mmol, 1.05 equiv) in 1,4-dioxane (0.25 mL) at r.t. for 4 hours, then 1-dodecanethiol (24.2 mg, 0.12 mmol, 1.2 equiv) and Li_2CO_3 (7.4 mg, 0.1 mmol, 1.0 equiv) at r.t. for 8 hours afforded compound **4t** in 35% yield as a white solid by chromatography on silica gel eluting with PE/EA (50:1). R_f (2% EA/hexane) = 0.5. ^{1}H NMR (400 MHz, $CDCl_3$) δ 3.15 (s, 1H), 2.95 (dd, *J* = 11.5, 6.6 Hz, 2H), 2.78 (t, *J* = 7.3 Hz, 2H), 1.64 (dt, *J* = 14.8, 7.3 Hz, 2H), 1.53 – 1.44 (m, 2H), 1.37 – 1.29 (m, 4H), 1.19 (s, 44H), 0.81 (t, *J* = 6.6 Hz, 6H). ^{13}C NMR (100 MHz, $CDCl_3$) δ 50.9, 39.2, 31.9, 29.7, 29.7, 29.6, 29.6, 29.6, 29.5, 29.4, 29.4, 29.4, 29.3, 29.2, 29.1, 28.5, 26.9, 22.7, 14.1. IR (film) 2953, 2916, 2847, 1462, 1402, 1371, 1296, 1242, 1061, 1022, 723, 633. HRMS (EI) Calcd for $C_{30}H_{63}NS_3$ 533.4123, found 533.4133.

Synthesis of cyclic peptides

15-Membered cyclic peptide (**5a**)

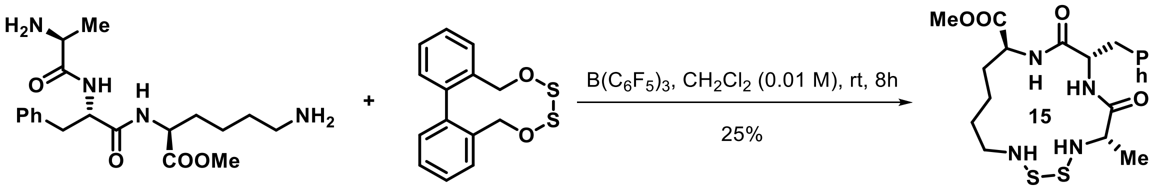

To a Schlenk tube were added H-Ala-Phe-Lys-OMe (75.6 mg, 0.2 mmol, 1.0 equiv), **1f** (55.2 mg, 0.2 mmol, 1.0 equiv) and CH_2Cl_2 (20 mL), then $B(C_6F_5)_3$ (5.1 mg, 0.01 mmol, 5 mol%) was added, the mixture was stirred at r.t. for 8 hours under air before it was concentrated under vacuum. Purification of the residue by flash column chromatography (silica gel, PE/EA = 2:1→1:2) yielded **5a** (22.0 mg, 25%) as a white solid. R_f (5% MeOH/DCM) = 0.5. ^{1}H NMR (400 MHz, CD_3OD) δ 7.26 (d, *J* = 4.4 Hz, 4H), 7.22 – 7.16 (m, 1H), 4.73 (dd, *J* = 9.2, 6.4 Hz, 1H), 4.52 (dd, *J* = 7.0, 3.9 Hz, 1H), 3.94 (q, *J* = 6.9 Hz, 1H), 3.70 (s, 3H), 3.17 (dd, *J* = 14.0, 6.4 Hz, 1H), 2.98 (ddd, *J* = 13.6, 9.4, 6.3 Hz, 2H), 2.80 (dt, *J* = 13.6, 4.5 Hz, 1H), 1.88 – 1.70 (m, 2H), 1.53 (ddd, *J* = 14.1,

10.8, 5.7 Hz, 2H), 1.31 – 1.26 (m, 6H), 1.11 (d, *J* = 6.9 Hz, 3H). ^{13}C NMR (100 MHz, CD_3OD) δ 175.9, 172.0, 170.9, 137.2, 128.9, 128.0, 126.3, 58.4, 53.6, 53.3, 51.7, 51.4, 34.7, 30.6, 28.9, 21.3, 18.5. IR (film) 3325, 2976, 2916, 1732, 1649, 1528, 1452, 1383, 1290, 1184, 1091, 1051, 885, 808, 740. HRMS (ESI) Calcd for $C_{19}H_{29}N_4O_4S_2$ (M+H$^+$) 441.1625, found 441.1622.

18-Membered cyclic peptide (**5b**)

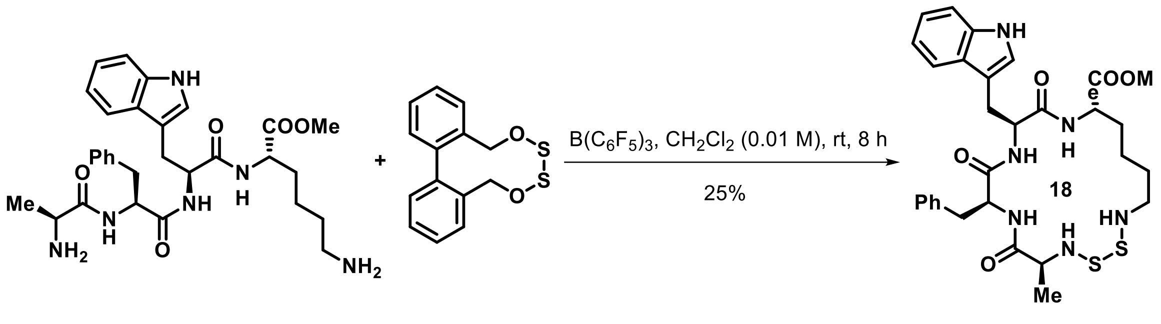

To a Schlenk tube were added H-Ala-Phe-Trp-Lys-OMe (112.9 mg, 0.2 mmol, 1.0 equiv), **1f** (55.2 mg, 0.2 mmol, 1.0 equiv) and CH_2Cl_2 (20 mL), then $B(C_6F_5)_3$ (5.1 mg, 0.01 mmol, 5 mol%) was added, the mixture was stirred at r.t. for 8 hours under air before it was concentrated under vacuum. Purification of the residue by flash column chromatography (silica gel, PE/EA = 2:1 →1:2) yielded **5b** (28.8 mg, 23%) as a white solid. R_f (5% MeOH/DCM) = 0.5. ^{1}H NMR (400 MHz, CD_3OD) δ 7.57 (t, *J* = 7.9 Hz, 2H), 7.39 (td, *J* = 7.6, 1.3 Hz, 1H), 7.33 (t, *J* = 7.4 Hz, 2H), 7.29 (dd, *J* = 11.5, 4.8 Hz, 2H), 7.25 – 7.19 (m, 3H), 7.13 – 7.10 (m, 2H), 7.09 (dd, *J* = 7.9, 0.8 Hz, 1H), 7.01 (t, *J* = 7.5 Hz, 1H), 6.96 (s, 1H), 4.64 (t, *J* = 6.7 Hz, 1H), 4.52 – 4.45 (m, 2H), 4.30 (s, 2H), 3.65 (dd, *J* = 9.4, 4.5 Hz, 1H), 3.62 (s, 3H), 3.36 (dd, *J* = 14.4, 6.6 Hz, 1H), 3.20 (dd, *J* = 14.5, 7.0 Hz, 1H), 3.12 (dd, *J* = 14.2, 4.4 Hz, 1H), 2.84 (dd, *J* = 14.1, 9.9 Hz, 1H), 2.76 – 2.64 (m, 1H), 1.78 (ddd, *J* = 11.1, 7.9, 3.9 Hz, 1H), 1.65 – 1.54 (m, 1H), 1.51 – 1.40 (m, 2H), 1.29 (s, 2H), 1.00 (d, *J* = 7.0 Hz, 3H). ^{13}C NMR (100 MHz, CD_3OD) δ 176.5, 172.4, 172.1, 171.8, 139.2, 138.8, 136.6, 129.2, 128.9, 128.4, 127.4, 126.7, 123.8, 121.1, 118.5, 118.3, 111.0, 109.1, 61.4, 55.3, 54.4, 51.4, 51.3, 50.0, 36.8, 30.1, 27.0, 26.4, 22.5, 18.1. IR (film) 3373, 2978, 2887, 1689, 1554, 1390, 1333, 1092, 1051, 883, 795, 739. HRMS (ESI) Calcd for $C_{30}H_{39}N_6O_5S_2$ (M+H$^+$) 627.2418, found 627.2420.

Optimization of polysulfuration with phenylboronic acid.

Supplementary Table 2. Optimization of disulfurating reagent **1f** with phenylboronic acid[a][b].

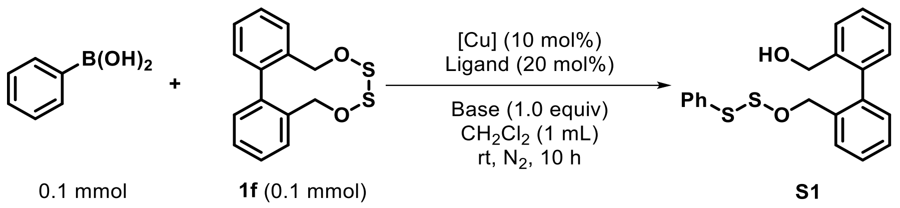

Entry	[Cu]	Ligand	Base	Solvent	Yield
1	CuI	2,2'-bpy	Li_2CO_3	toluene	N.R.
2	CuCl	2,2'-bpy	Li_2CO_3	toluene	N.R.
3	CuTc	2,2'-bpy	Li_2CO_3	toluene	N.R.
4	$CuSO_4 \cdot 5H_2O$	2,2'-bpy	Li_2CO_3	toluene	N.R.
5	$Cu(OAc)_2$	2,2'-bpy	Li_2CO_3	toluene	N.R.
6	$Cu(MeCN)_4PF_6$	2,2'-bpy	Li_2CO_3	THF	<10%
7	$Cu(MeCN)_4PF_6$	2,2'-bpy	Li_2CO_3	dioxane	<10%
8	$Cu(MeCN)_4PF_6$	2,2'-bpy	Li_2CO_3	Acetone	<10%
9	$Cu(MeCN)_4PF_6$	2,2'-bpy	Li_2CO_3	MeCN	<10%
10	$Cu(MeCN)_4PF_6$	2,2'-bpy	Li_2CO_3	DMF	trace
11	$Cu(MeCN)_4PF_6$	2,2'-bpy	Li_2CO_3	CH_2Cl_2	74%
12	$Cu(MeCN)_4PF_6$	2,2'-bpy	Li_2CO_3	$ClCH_2CH_2Cl$	72%
13	$Cu(MeCN)_4PF_6$	6,6'-diMe-2,2'-bpy	Li_2CO_3	CH_2Cl_2	N.R.
14	$Cu(MeCN)_4PF_6$	2,9-diMe-1,10-phen	Li_2CO_3	CH_2Cl_2	N.R.
15	$Cu(MeCN)_4PF_6$	4,7-diPh-1,10-phen	Li_2CO_3	CH_2Cl_2	70%
16	$Cu(MeCN)_4PF_6$	4,5-diazafluorenone	Li_2CO_3	CH_2Cl_2	N.R.
17	$Cu(MeCN)_4PF_6$	1,10-phen	Li_2CO_3	CH_2Cl_2	74%

18	$Cu(MeCN)_4PF_6$	2,2'-bpy	NaOAc	CH_2Cl_2	50%
19	$Cu(MeCN)_4PF_6$	2,2'-bpy	Et_3N	CH_2Cl_2	trace
20	$Cu(MeCN)_4PF_6$	2,2'-bpy	LiO*t*Bu	CH_2Cl_2	trace
21	$Cu(MeCN)_4PF_6$	2,2'-bpy	K_3PO_4	CH_2Cl_2	trace
22	$Cu(MeCN)_4PF_6$	2,2'-bpy	Na_3PO_4	CH_2Cl_2	68%
23	$Cu(MeCN)_4PF_6$	2,2'-bpy	$NaBF_4$	CH_2Cl_2	65%
24	$Cu(MeCN)_4PF_6$	2,2'-bpy	-	CH_2Cl_2	56%
25	$Cu(MeCN)_4PF_6$	2,2'-bpy	-	CH_2Cl_2	84%[c]
26	$Cu(MeCN)_4BF_4$	2,2'-bpy	-	CH_2Cl_2	70%[c]
27	$Cu(MeCN)_4PF_6$	2,2'-bpy	-	CH_2Cl_2	40%[c][d]
28	$Cu(MeCN)_4PF_6$	2,2'-bpy	-	CH_2Cl_2	44%[c][e]
29	$Cu(MeCN)_4PF_6$	2,2'-bpy	-	CH_2Cl_2	58%[c][f]

[a]Condition: phenylboronic acid (0.10 mmol), **1f** (0.1 mmol), $Cu(MeCN)_4PF_6$ (0.01 mmol, 10 mol%), 2,2'-bpy (0.02 mmol, 20 mol%), CH_2Cl_2 (1 mL), rt, under N_2. [b]Isolated yield. [c]$PhB(OH)_2$ (0.15 mmol), 24 h. [d]**1d** instead of **1f**. [e]$Cu(MeCN)_4PF_6$ (5 mol%). [f]0 °C instead of rt.

To a Schlenk tube were added phenylboronic acid (12.2 mg, 0.1 mmol, 1.0 equiv), **1f** (27.6 mg, 0.10 mmol, 1 equiv), $Cu(MeCN)_4PF_6$ (3.7 mg, 0.01 mmol, 10 mol%), ligand (0.02 mmol, 20 mol%), and fresh distilled CH_2Cl_2 (1 mL), the mixture was stirred at r.t. for 10 hours under N_2 atmosphere before it was concentrated under vacuum. Purification by column chromatography afforded the desired product **S1** as a colorless oil.

Synthesis of compound **S1**

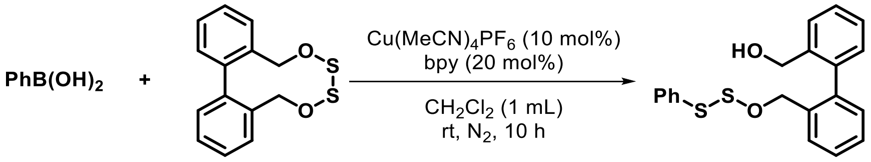

0.15 mmol **1f** (0.1 mmol) **S1**

To a Schlenk tube were added phenylboronic acid (18.3 mg, 0.15 mmol, 1.5 equiv), **1f** (27.6 mg, 0.10 mmol, 1 equiv), $Cu(MeCN)_4PF_6$ (3.7 mg, 0.01 mmol, 10 mol%), 2,2'-bpy (3.1 mg, 0.02 mmol, 20 mol%), and fresh distilled CH_2Cl_2 (1 mL), the mixture was stirred at r.t. for 10 hours under N_2 atmosphere before it was concentrated under vacuum. Purification of the residue by flash column chromatography (silica gel, PE/EA = 10:1→5:1) yielded the **S1** (29.7 mg, 84%) as a colorless oil. R_f (20% EA/hexane) = 0.3. ^{1}H NMR (400 MHz, Acetone-d$_6$) δ 7.48 (dd, J = 7.7, 0.6 Hz, 1H), 7.39 (t, J = 1.8 Hz, 1H), 7.38 – 7.36 (m, 1H), 7.35 – 7.30 (m, 1H), 7.27 (t, J = 1.7 Hz, 1H), 7.24 (ddd, J = 3.8, 3.2, 2.5 Hz, 4H), 7.22 – 7.18 (m, 1H), 7.10 (td, J = 7.5, 1.0 Hz, 1H), 7.07 – 7.02 (m, 1H), 6.92 (dd, J = 7.5, 1.1 Hz, 1H), 4.44 (q, J = 10.9 Hz, 2H), 4.16 (qd, J = 13.4, 5.4 Hz, 2H), 3.85 (t, J = 5.5 Hz, 1H). ^{13}C NMR (100 MHz, Acetone-d$_6$) δ 140.8, 139.9, 138.2, 138.2, 134.7, 129.9, 129.8, 129.4, 129.3, 128.4, 128.2, 128.2, 127.7, 127.6, 127.3, 126.4, 75.6, 61.6. IR (film) 3327, 3061, 2877, 1473, 1440, 1197, 1010, 758, 690, 600. HRMS (EI) Calcd for $C_{20}H_{18}O_2S_2$ 354.0748, found 354.0749.

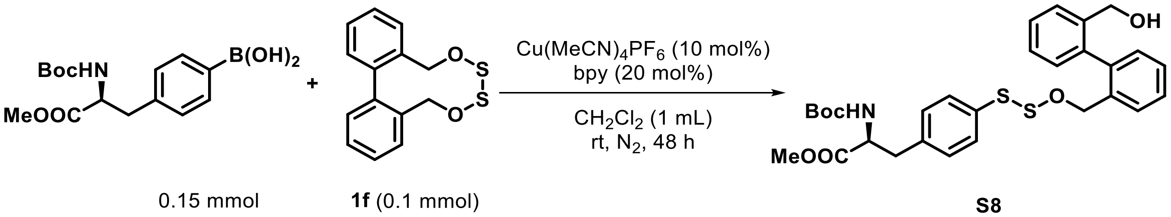

To a Schlenk tube were added (S)-(4-(2-((tert-butoxycarbonyl)amino)-3-methoxy-3-oxopropyl)phenyl)boronic acid (48.4 mg, 0.15 mmol, 1.5 equiv), **1f** (27.6 mg, 0.10 mmol, 1 equiv), $Cu(MeCN)_4PF_6$ (3.7 mg, 0.01 mmol, 10 mol%), 2,2'-bpy (3.1 mg, 0.02 mmol, 20 mol%), and fresh distilled CH_2Cl_2 (1 mL), the mixture was stirred at r.t. for 48 hours under N_2 atmosphere before it was concentrated under vacuum. Purification of the residue by flash column chromatography (silica gel, PE/EA = 5:1→2:1) yielded the **S8** (25.5 mg, 46%) as a colorless oil. R_f (50% EA/hexane) = 0.3.

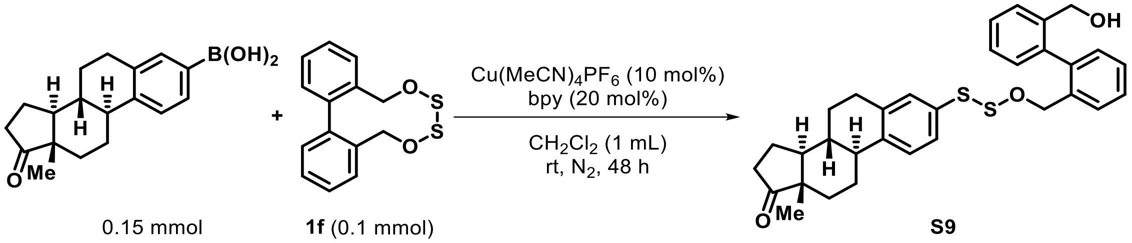

To a Schlenk tube were added arylboronic acid (44.7 mg, 0.15 mmol, 1.5 equiv), **1f** (27.6 mg, 0.10 mmol, 1 equiv), $Cu(MeCN)_4PF_6$ (3.7 mg, 0.01 mmol, 10 mol%), 2,2'-bpy (3.1 mg, 0.02 mmol, 20 mol%), and fresh distilled CH_2Cl_2 (1 mL), the mixture was stirred at r.t. for 48 hours under N_2

atmosphere before it was concentrated under vacuum. Purification of the residue by flash column chromatography (silica gel, PE/EA = 2:1) yielded the **S9** (25.5 mg, 40%) as a viscous gel. R_f (50% EA/hexane) = 0.3.

General Procedure and Spectra Data of Polysulfuration with phenylboronic acid.

Synthesis of compound **6**

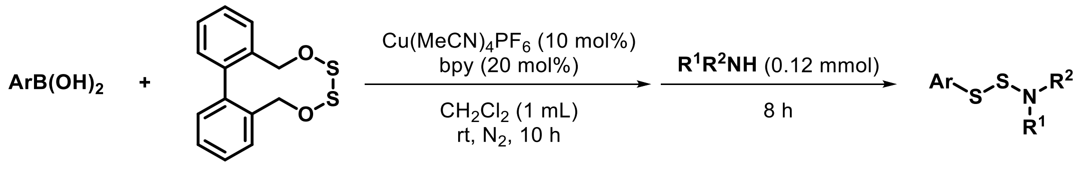

To a Schlenk tube were added arylboronic acid (0.15 mmol, 1.5 equiv), **1f** (29 mg, 0.10 mmol, 1 equiv), $Cu(MeCN)_4PF_6$ (3.7 mg, 0.01 mmol, 10 mol%), 2,2'-bpy (3.1 mg, 0.02 mmol, 20 mol%), and redistilled CH_2Cl_2 (1 mL), the mixture was stirred at r.t. for 10 hours under N_2 atmosphere. After **1f** was consumed, amine (0.12 mmol, 1.2 equiv) were added to the mixture. The mixture was stirred at r.t. for 8 hours under air before it was concentrated under vacuum. Purification by column chromatography afforded the desired product **6**.

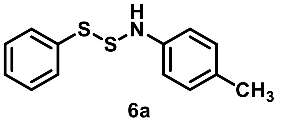

6a

The reaction of phenylboronic acid (18.3 mg, 0.15 mmol, 1.5 equiv), **1f** (29 mg, 0.10 mmol, 1 equiv), $Cu(MeCN)_4PF_6$ (3.7 mg, 0.01 mmol, 10 mol%) and 2,2'-bpy (3.1 mg, 0.02 mmol, 20 mol%) in redistilled CH_2Cl_2 (1 mL) at r.t. under N_2 for 10 hours, then p-toluidine (12.8 mg, 0.12 mmol, 1.2 equiv) at r.t. for 8 hours afforded compound **6a** in 70% yield as a colorless oil by chromatography on silica gel eluting with PE/EA (50:1→20:1). R_f (5% EA/hexane) = 0.3. ^{1}H NMR (400 MHz, DMSO-d_6) δ 8.10 (s, 1H), 7.51 (d, J = 7.5 Hz, 2H), 7.32 (dd, J = 10.2, 4.1 Hz, 2H), 7.26 (dd, J = 7.7, 5.3 Hz, 1H), 6.95 (d, J = 7.8 Hz, 2H), 6.87 (d, J = 7.8 Hz, 2H), 2.14 (s, 3H). ^{13}C NMR (100 MHz, DMSO-d_6) δ 143.4, 137.8, 130.3, 130.1, 129.9, 129.6, 128.2, 116.8, 20.6. IR (film) 3340, 2972, 2920, 1612, 1508, 1475, 1438, 1373, 1226, 1022, 812, 740, 688. HRMS (EI) Calcd for $C_{13}H_{13}NS_2$ 247.0489, found 247.0492.

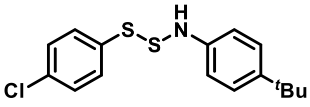

6b

The reaction of (4-chlorophenyl)boronic acid (23.4 mg, 0.15 mmol, 1.5 equiv), **1f** (29 mg, 0.10 mmol, 1 equiv), $Cu(MeCN)_4PF_6$ (3.7 mg, 0.01 mmol, 10 mol%) and 2,2'-bpy (3.1 mg, 0.02 mmol, 20 mol%) in redistilled CH_2Cl_2 (1 mL) at r.t. under N_2 for 10 hours, then 4-(tert-butyl)aniline (17.9 mg, 0.12 mmol, 1.2 equiv) at r.t. for 8 hours afforded compound **6b** in 73% yield as a light yellow oil by chromatography on silica gel eluting with PE/EA (50:1→20:1). R_f (5% EA/hexane) = 0.3. ^{1}H

NMR (400 MHz, $CDCl_3$) δ 7.32 (d, *J* = 8.5 Hz, 2H), 7.20 – 7.11 (m, 4H), 6.83 (d, *J* = 8.7 Hz, 2H), 5.19 (s, 1H), 1.21 (s, 9H). ^{13}C NMR (100 MHz, $CDCl_3$) δ 145.1, 142.0, 136.0, 134.1, 132.6, 129.1, 126.1, 116.9, 34.2, 31.5. IR (film) 3350, 2963, 1608, 1510, 1473, 1267, 1234, 1184, 1092, 1013, 899, 818, 742, 553. HRMS (EI) Calcd for $C_{16}H_{18}ClNS_2$ 323.0569, found 323.0564.

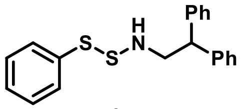
6c

The reaction of phenylboronic acid (18.3 mg, 0.15 mmol, 1.5 equiv), **1f** (29 mg, 0.10 mmol, 1 equiv), $Cu(MeCN)_4PF_6$ (3.7 mg, 0.01 mmol, 10 mol%) and 2,2'-bpy (3.1 mg, 0.02 mmol, 20 mol%) in redistilled CH_2Cl_2 (1 mL) at r.t. under N_2 for 10 hours, then 2,2-diphenylethan-1-amine (23.7 mg, 0.12 mmol, 1.2 equiv) at r.t. for 8 hours afforded compound **6c** in 75% yield as a light yellow oil by chromatography on silica gel eluting with PE/EA (50:1). R_f (20% EA/hexane) = 0.3. ^{1}H NMR (400 MHz, DMSO-d_6) δ 7.62 – 7.58 (m, 2H), 7.39 – 7.33 (m, 2H), 7.31 – 7.13 (m, 11H), 5.34 (t, *J* = 4.7 Hz, 1H), 4.24 (t, *J* = 7.8 Hz, 1H), 3.49 (dd, *J* = 7.7, 4.8 Hz, 2H). ^{13}C NMR (100 MHz, $CDCl_3$) δ 141.9, 138.2, 129.9, 129.2, 128.8, 128.2, 127.5, 126.8, 55.6, 50.6. IR (film) 3315, 3059, 2922, 2852, 1739, 1579, 1492, 1450, 1438, 1066, 1024, 1024, 1001, 739, 698. HRMS (EI) Calcd for $C_{20}H_{19}NS_2$ 337.0959, found 337.0955.

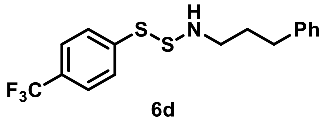
6d

The reaction of (4-(trifluoromethyl)phenyl)boronic acid (28.5 mg, 0.15 mmol, 1.5 equiv), **1f** (29 mg, 0.10 mmol, 1 equiv), $Cu(MeCN)_4PF_6$ (3.7 mg, 0.01 mmol, 10 mol%) and 2,2'-bpy (3.1 mg, 0.02 mmol, 20 mol%) in redistilled CH_2Cl_2 (1 mL) at r.t. under N_2 for 10 hours, then 3-phenylpropan-1-amine (16.2 mg, 0.12 mmol, 1.2 equiv) at r.t. for 8 hours afforded compound **6d** in 68% yield as a colorless oil by chromatography on silica gel eluting with PE/EA (20:1). R_f (5% EA/hexane) = 0.3. ^{1}H NMR (400 MHz, $CDCl_3$) δ 7.59 (d, *J* = 8.1 Hz, 2H), 7.48 (d, *J* = 8.2 Hz, 2H), 7.16 (d, *J* = 7.4 Hz, 2H), 7.09 (t, *J* = 6.9 Hz, 1H), 7.02 (d, *J* = 7.4 Hz, 2H), 3.02 (s, 1H), 2.86 (dd, *J* = 12.8, 6.3 Hz, 2H), 2.51 (t, *J* = 7.6 Hz, 2H), 1.79 – 1.67 (m, 2H). ^{19}F NMR (282 MHz, $CDCl_3$) δ -62.4.^{13}C NMR (100 MHz, $CDCl_3$) δ 143.1, 141.4, 129.0 (q, $^2J_{C\text{-}F}$ = 32.7 Hz), 128.4, 128.4, 128.3, 126.0, 125.8 (q, $^3J_{C\text{-}F}$ = 3.8 Hz), 124.1 (q, $^1J_{C\text{-}F}$ = 271.9 Hz), 50.4, 33.0, 30.7. IR (film) 3350, 2926, 2856, 1602, 1494, 1454, 1400, 1323, 1165, 1122, 1105, 1080, 1061, 1012, 830, 698. HRMS (EI) Calcd for $C_{16}H_{16}F_3NS_2$ 343.0676, found 343.0681.

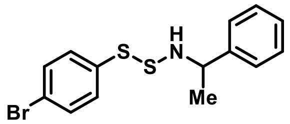
6e

The reaction of (4-bromophenyl)boronic acid (30.0 mg, 0.15 mmol, 1.5 equiv), **1f** (29 mg, 0.10 mmol, 1 equiv), $Cu(MeCN)_4PF_6$ (3.7 mg, 0.01 mmol, 10 mol%) and 2,2'-bpy (3.1 mg, 0.02 mmol, 20 mol%) in redistilled CH_2Cl_2 (1 mL) at r.t. under N_2 for 10 hours, then 1-phenylethan-1-amine (14.5 mg, 0.12 mmol,

1.2 equiv) at r.t. for 8 hours afforded compound **6e** in 83% yield as a yellow oil by chromatography on silica gel eluting with PE/EA (20:1). R_f (5% EA/hexane) = 0.3. ^{1}H NMR (400 MHz, $CDCl_3$) δ 7.30 (q, *J* = 8.5 Hz, 4H), 7.25 – 7.20 (m, 2H), 7.20 – 7.15 (m, 1H), 7.11 (d, *J* = 7.4 Hz, 2H), 4.11 (q, *J* = 6.5 Hz, 1H), 3.36 (s, 1H), 1.30 (d, *J* = 6.6 Hz, 3H). ^{13}C NMR (100 MHz, $CDCl_3$) δ 143.2, 137.3, 132.1, 131.0, 128.6, 127.6, 127.0, 121.2, 58.0, 22.7. IR (film) 3317, 3028, 2974, 2924, 2868, 1602, 1493, 1470, 1452, 1384, 1369, 1340, 1307, 1078, 1007, 812, 760, 698, 640. HRMS (EI) Calcd for $C_{14}H_{14}BrNS_2$ 338.9751, found 338.9748.

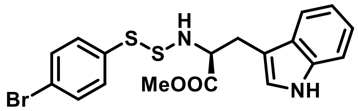

6f

The reaction of (4-bromophenyl)boronic acid (30.0 mg, 0.15 mmol, 1.5 equiv), **1f** (29 mg, 0.10 mmol, 1 equiv), $Cu(MeCN)_4PF_6$ (3.7 mg, 0.01 mmol, 10 mol%) and 2,2'-bpy (3.1 mg, 0.02 mmol, 20 mol%) in redistilled CH_2Cl_2 (1 mL) at r.t. under N_2 for 10 hours, then methyl L-tryptophanate (14.5 mg, 0.12 mmol, 1.2 equiv) in toluene (1 mL) at r.t. for 8 hours afforded compound **6f** in 67% yield as a yellow oil by chromatography on silica gel eluting with PE/EA (5:1). R_f (20% EA/hexane) = 0.2. ^{1}H NMR (400 MHz, $CDCl_3$) δ 7.96 (s, 1H), 7.50 (d, *J* = 7.9 Hz, 1H), 7.28 – 7.22 (m, 3H), 7.14 (t, *J* = 7.5 Hz, 1H), 7.09 – 7.03 (m, 3H), 6.90 (d, *J* = 1.8 Hz, 1H), 4.00 (dt, *J* = 7.5, 5.2 Hz, 1H), 3.63 (d, *J* = 5.0 Hz, 1H), 3.59 (s, 3H), 3.21 (dd, *J* = 14.6, 5.2 Hz, 1H), 3.03 (dd, *J* = 14.6, 7.7 Hz, 1H). ^{13}C NMR (100 MHz, $CDCl_3$) δ 173.4, 136.8, 136.2, 132.0, 131.7, 127.2, 123.1, 122.4, 121.8, 119.8, 118.8, 111.2, 110.2, 63.2, 52.4, 28.8. IR (film) 3414, 2976, 2949, 1734, 1470, 1456, 1437, 1340, 1211, 1093, 1006, 814, 742. HRMS (EI) Calcd for $C_{18}H_{17}BrN_2O_2S_2$ 435.9915, found 435.9908.

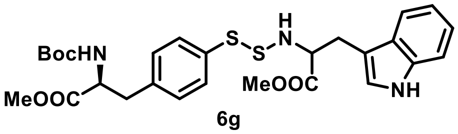

6g

The reaction of (S)-(4-(2-((tert-butoxycarbonyl)amino)-3-methoxy-3-oxopropyl)phenyl)boronic acid (48.4 mg, 0.15 mmol, 1.5 equiv), **1f** (29 mg, 0.10 mmol, 1 equiv), $Cu(MeCN)_4PF_6$ (3.7 mg, 0.01 mmol, 10 mol%) and 2,2'-bpy (3.1 mg, 0.02 mmol, 20 mol%) in redistilled CH_2Cl_2 (1 mL) at r.t. under N_2 for 48 hours, then methyl L-tryptophanate (14.5 mg, 0.12 mmol, 1.2 equiv) in toluene (1 mL) at r.t. for 8 hours afforded compound **6g** in 34% yield as a yellow oil by chromatography on silica gel eluting with PE/EA (5:1→2:1). R_f (50% EA/hexane) = 0.3. ^{1}H NMR (400 MHz, $CDCl_3$) δ 8.30 (s, 1H), 7.53 (d, *J* = 7.7 Hz, 1H), 7.30 (d, *J* = 7.9 Hz, 1H), 7.15 (t, *J* = 7.5 Hz, 1H), 7.10 – 7.05 (m, 1H), 7.01 (d, *J* = 6.2 Hz, 2H), 6.86 (d, *J* = 7.2 Hz, 2H), 6.78 (s, 1H), 4.93 (d, *J* = 7.4 Hz, 1H), 4.52 (s, 1H), 4.05 (dd, *J* = 14.0, 6.9 Hz, 1H), 3.68 (s, 3H), 3.65 – 3.61 (m, 3H), 3.52 (s, 1H), 3.27 (dd, *J* = 20.1, 7.0 Hz, 1H), 3.05 – 2.96 (m, 2H), 2.89 (dd, *J* = 13.8, 6.5 Hz, 1H), 1.38 (s, 9H). ^{13}C NMR (100 MHz, $CDCl_3$) δ 173.3, 172.3,

155.2, 136.4, 135.7, 135.5, 130.2, 129.9, 127.1, 123.2, 122.2, 119.6, 118.8, 111.4, 109.9, 80.4, 62.2, 54.5, 52.4, 52.3, 37.9, 28.8, 28.3. IR (film) 3416, 2972, 2926, 1736, 1697, 1491, 1437, 1365, 1213, 1165, 1101, 1051, 1016, 881, 742. HRMS (ESI) Calcd for $C_{27}H_{34}N_3O_6S_2$ ($M+H^+$) 560.1884, found 560.1882.

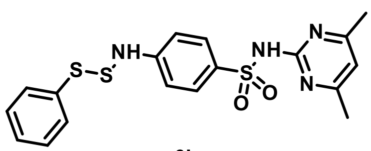

6h

The reaction of phenylboronic acid (18.3 mg, 0.15 mmol, 1.5 equiv), **1f** (29 mg, 0.10 mmol, 1 equiv), $Cu(MeCN)_4PF_6$ (3.7 mg, 0.01 mmol, 10 mol%) and 2,2'-bpy (3.1 mg, 0.02 mmol, 20 mol%) in redistilled CH_2Cl_2 (1 mL) at r.t. under N_2 for 10 hours, then sulfamethazine (33.4 mg, 0.12 mmol, 1.2 equiv) in toluene (1 mL) at r.t. for 24 hours afforded compound **6h** in 66% yield as a pale yellow solid by chromatography on silica gel eluting with PE/EA (2:1). R_f (50% EA/hexane) = 0.1. ^{1}H NMR (400 MHz, $CDCl_3$) δ 7.86 (d, J = 8.7 Hz, 2H), 7.44 – 7.40 (m, 2H), 7.24 – 7.19 (m, 3H), 6.91 (d, J = 8.8 Hz, 2H), 6.55 (s, 1H), 5.79 (s, 1H), 2.29 (s, 6H). ^{13}C NMR (100 MHz, $CDCl_3$) δ 168.4, 156.3, 149.4, 136.7, 131.6, 131.2, 130.6, 129.2, 128.5, 115.5, 115.0, 23.6, 23.6. IR (film) 3343, 2956, 2922, 1595, 1552, 1490, 1439, 1153, 1084, 679, 584. HRMS (ESI) Calcd for $C_{18}H_{19}N_4O_2S_3$ ($M+H^+$) 419.0665, found 419.0661.

Synthesis of compound **7**

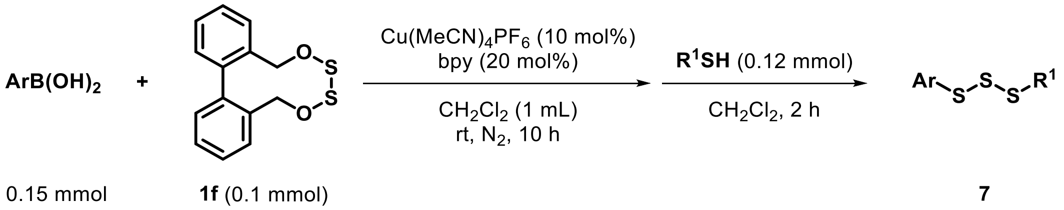

To a Schlenk tube were added arylboronic acid (0.15 mmol, 1.5 equiv), **1f** (29 mg, 0.10 mmol, 1 equiv), $Cu(MeCN)_4PF_6$ (3.7 mg, 0.01 mmol, 10 mol%), 2,2'-bpy (3.1 mg, 0.02 mmol, 20 mol%), and redistilled CH_2Cl_2 (1 mL), the mixture was stirred at r.t. for 10 hours under N_2 atmosphere. After **1f** was consumed, the mixture was filtered through a pad of silica gel. The mercaptan (0.12 mmol, 1.2 equiv) and $B(C_6F_5)_3$ (0.5 mg, 0.001 mmol, 1 mol%) was added to the mixture. The mixture was stirred at r.t. for 2 hours under air before it was concentrated under vacuum. Purification by column chromatography afforded the desired product **7**.

The reaction of phenylboronic acid (18.3 mg, 0.15 mmol, 1.5 equiv), **1f** (29 mg, 0.10 mmol, 1 equiv), $Cu(MeCN)_4PF_6$ (3.7 mg, 0.01 mmol, 10 mol%) and 2,2'-bpy (3.1 mg, 0.02 mmol, 20 mol%) in redistilled CH_2Cl_2 (1 mL) at r.t. under N_2 for 10 hours, then pyrimidine-2-thiol (13.4 mg,

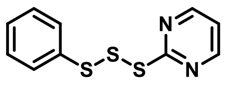
7a

0.12 mmol, 1.2 equiv) in CH_2Cl_2 (1 mL) at r.t. for 2 hours afforded compound **7a** in 75% yield as a white solid by chromatography on silica gel eluting with PE/EA (10:1). R_f (10% EA/hexane) = 0.3. ^1H NMR (400 MHz, $CDCl_3$) δ 8.50 (d, J = 4.8 Hz, 2H), 7.58 – 7.54 (m, 2H), 7.28 – 7.22 (m, 3H), 7.00 (t, J = 4.8 Hz, 1H). ^{13}C NMR (100 MHz, $CDCl_3$) δ 170.2, 157.9, 136.1, 131.2, 129.1, 128.7, 118.2. IR (film) 3190, 1658, 1554, 1379, 1168, 742, 686. HRMS (EI) Calcd for $C_{10}H_8N_2S_3$ 251.9850, found 251.9849.

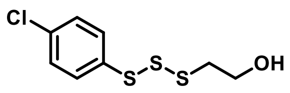
7b

The reaction of (4-chlorophenyl)boronic acid (23.4 mg, 0.15 mmol, 1.5 equiv), **1f** (29 mg, 0.10 mmol, 1 equiv), $Cu(MeCN)_4PF_6$ (3.7 mg, 0.01 mmol, 10 mol%) and 2,2'-bpy (3.1 mg, 0.02 mmol, 20 mol%) in redistilled CH_2Cl_2 (1 mL) at r.t. under N_2 for 10 hours, then 2-mercaptoethan-1-ol (9.4 mg, 0.12 mmol, 1.2 equiv) at r.t. for 2 hours afforded compound **7b** in 65% yield as a yellow oil by chromatography on silica gel eluting with PE/DCM (2:1). R_f (20% EA/hexane) = 0.3. ^1H NMR (400 MHz, $CDCl_3$) δ 7.49 – 7.45 (m, 2H), 7.31 – 7.25 (m, 2H), 3.84 (t, J = 5.8 Hz, 2H), 2.97 (t, J = 5.8 Hz, 2H), 1.90 (s, 1H). ^{13}C NMR (100 MHz, $CDCl_3$) δ 135.0, 134.8, 131.8, 129.4, 59.9, 41.7. IR (film) 3354, 2922, 2872, 1641, 1570, 1472, 1387, 1089, 1043, 1010, 814. 742. HRMS (EI) Calcd for $C_8H_9ClOS_3$ 251.9504, found 251.9503.

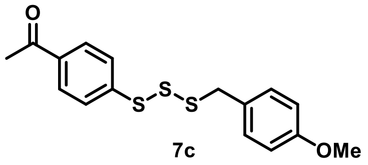
7c

The reaction of (4-acetylphenyl)boronic acid (24.5 mg, 0.15 mmol, 1.5 equiv), **1f** (29 mg, 0.10 mmol, 1 equiv), $Cu(MeCN)_4PF_6$ (3.7 mg, 0.01 mmol, 10 mol%) and 2,2'-bpy (3.1 mg, 0.02 mmol, 20 mol%) in redistilled CH_2Cl_2 (1 mL) at r.t. under N_2 for 24 hours, then (4-methoxyphenyl)methanethiol (18.5 mg, 0.12 mmol, 1.2 equiv) at r.t. for 2 hours afforded compound **7c** in 63% yield as a colorless oil by chromatography on silica gel eluting with PE/EA (10:1). R_f (5% EA/hexane) = 0.2. ^1H NMR (400 MHz, $CDCl_3$) δ 7.84 (d, J = 8.4 Hz, 2H), 7.56 (d, J = 8.4 Hz, 2H), 7.12 (d, J = 8.6 Hz, 2H), 6.77 (d, J = 8.6 Hz, 2H), 3.99 (s, 2H), 3.72 (s, 3H), 2.51 (s, 3H). ^{13}C NMR (100 MHz, $CDCl_3$) δ 197.1, 159.3, 143.2, 136.0, 130.6, 128.9, 128.3, 127.8, 114.1, 55.3, 43.0, 26.6. IR (film) 3001, 2955, 1680, 1585, 1462, 1392, 1248, 1086, 956, 820, 617, 588. HRMS (EI) Calcd for $C_{16}H_{16}O_2S_3$ 336.0312, found 336.0316.

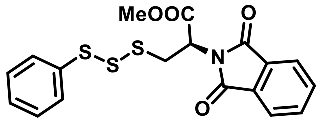
7d

The reaction of phenylboronic acid (18.3 mg, 0.15 mmol, 1.5 equiv), **1f** (29 mg, 0.10 mmol, 1 equiv), $Cu(MeCN)_4PF_6$ (3.7 mg, 0.01 mmol, 10 mol%) and 2,2'-bpy (3.1 mg, 0.02 mmol, 20 mol%) in redistilled CH_2Cl_2 (1 mL) at r.t. under N_2 for 10 hours, then methyl (R)-2-(1,3-dioxoisoindolin-2-yl)-3-mercaptopropanoate (31.8 mg, 0.12 mmol, 1.2 equiv) at r.t. for 6 hours

afforded compound **7d** in 80% yield as a yellow oil by chromatography on silica gel eluting with PE/EA (5:1). R_f (20% EA/hexane) = 0.3. ^{1}H NMR (400 MHz, $CDCl_3$) δ 7.79 (dd, *J* = 5.5, 3.0 Hz, 2H), 7.67 (dd, *J* = 5.4, 3.1 Hz, 2H), 7.50 (dd, *J* = 7.9, 1.4 Hz, 2H), 7.28 – 7.18 (m, 3H), 5.19 (dd, *J* = 10.7, 4.7 Hz, 1H), 3.66 (s, 3H), 3.65 – 3.46 (m, 2H). ^{13}C NMR (100 MHz, $CDCl_3$) δ 168.4, 167.4, 136.4, 134.3, 131.8, 130.6, 129.2, 128.4, 123.7, 53.1, 51.0, 36.9. IR (film) 3068, 1745, 1713, 1467, 1437, 1385, 1240, 1172, 1068, 914, 866, 788, 688. HRMS (ESI) Calcd for $C_{18}H_{16}NO_4S_3$ (M+H$^+$) 406.0236, found 406.0231.

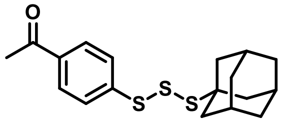

7e

The reaction of (4-acetylphenyl)boronic acid (24.5 mg, 0.15 mmol, 1.5 equiv), **1f** (29 mg, 0.10 mmol, 1 equiv), $Cu(MeCN)_4PF_6$ (3.7 mg, 0.01 mmol, 10 mol%) and 2,2'-bpy (3.1 mg, 0.02 mmol, 20 mol%) in redistilled CH_2Cl_2 (1 mL) at r.t. under N_2 for 24 hours, then 1-adamantylthiol (20.2 mg, 0.12 mmol, 1.2 equiv) at r.t. for 6 hours afforded compound **7e** in 52% yield as a pale yellow solid by chromatography on silica gel eluting with PE/EA (20:1). R_f (10% EA/hexane) = 0.3. ^{1}H NMR (400 MHz, $CDCl_3$) δ 7.89 – 7.82 (m, 2H), 7.60 – 7.53 (m, 2H), 2.52 (s, 3H), 2.02 (s, 3H), 1.84 (d, *J* = 2.5 Hz, 6H), 1.67 – 1.55 (m, 6H). ^{13}C NMR (100 MHz, $CDCl_3$) δ 197.2, 143.8, 135.7, 128.9, 127.9, 51.2, 42.5, 36.0, 29.9, 26.6. IR (film) 2905, 2848, 1684, 1587, 1392, 1259, 1089, 1051, 883, 819, 617. HRMS (EI) Calcd for $C_{18}H_{22}OS_3$ 350.0833, found 350.0835.

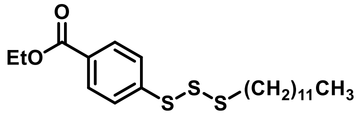

7g

The reaction of (4-(ethoxycarbonyl)phenyl)boronic acid (29.1 mg, 0.15 mmol, 1.5 equiv), **1f** (29 mg, 0.10 mmol, 1 equiv), $Cu(MeCN)_4PF_6$ (3.7 mg, 0.01 mmol, 10 mol%) and 2,2'-bpy (3.1 mg, 0.02 mmol, 20 mol%) in redistilled CH_2Cl_2 (1 mL) at r.t. under N_2 for 24 hours, then 1-dodecanethiol (24.2 mg, 0.12 mmol, 1.2 equiv) at r.t. for 2 hours afforded compound **7g** in 60% yield as a yellow oil by chromatography on silica gel eluting with PE/EA (20:1). R_f (5% EA/hexane) = 0.3. ^{1}H NMR (400 MHz, $CDCl_3$) δ 7.96 – 7.91 (m, 2H), 7.58 – 7.54 (m, 2H), 4.31 (q, *J* = 7.1 Hz, 2H), 2.85 – 2.71 (m, 2H), 1.68 – 1.56 (m, 2H), 1.32 (t, *J* = 7.1 Hz, 3H), 1.29 – 1.24 (m, 2H), 1.18 (s, 16H), 0.81 (t, *J* = 6.8 Hz, 3H). ^{13}C NMR (100 MHz, $CDCl_3$) δ 166.0, 142.9, 130.1, 129.5, 128.1, 61.1, 39.2, 31.9, 29.6, 29.6, 29.6, 29.5, 29.4, 29.1, 28.9, 28.5, 22.7, 14.3, 14.1. IR (film) 2922, 2853, 1718, 1591, 1564, 1487, 1396, 1269, 1103, 846, 758, 688. HRMS (EI) Calcd for $C_{21}H_{34}O_2S_3$ 414.1721, found 414.1723.

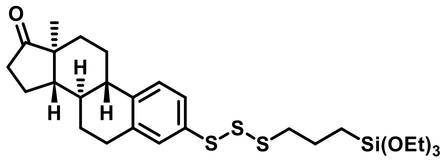

7h

The reaction of arylboronic acid (44.7 mg, 0.15 mmol, 1.5 equiv), **1f** (29 mg, 0.10 mmol, 1 equiv), $Cu(MeCN)_4PF_6$ (3.7 mg, 0.01 mmol, 10 mol%) and 2,2'-bpy (3.1 mg, 0.02 mmol, 20 mol%) in redistilled CH_2Cl_2 (1 mL) at r.t. under N_2 for 48 hours, then 3-(triethoxysilyl)propane-1-thiol (28.6 mg, 0.12 mmol, 1.2 equiv) at r.t. for 4 hours afforded compound **7h** in 34% yield as a yellow solid by chromatography on silica gel eluting with PE/EA (5:1). R_f (20% EA/hexane) = 0.3. ^1H NMR (400 MHz, $CDCl_3$) δ 7.32 (d, J = 8.1 Hz, 1H), 7.27 (s, 1H), 7.21 (s, 1H), 3.75 (q, J = 7.0 Hz, 6H), 2.88 – 2.79 (m, 4H), 2.44 (dd, J = 18.7, 8.7 Hz, 1H), 2.35 (dd, J = 12.9, 4.6 Hz, 1H), 2.24 (dd, J = 20.7, 10.6 Hz, 1H), 2.09 (dd, J = 18.5, 9.3 Hz, 1H), 2.04 – 1.88 (m, 3H), 1.84 – 1.73 (m, 2H), 1.61 – 1.31 (m, 10H), 1.16 (t, J = 7.0 Hz, 9H), 0.84 (s, 3H), 0.68 – 0.62 (m, 2H). ^{13}C NMR (100 MHz, $CDCl_3$) δ 140.3, 137.6, 133.9, 130.9, 127.9, 126.2, 58.4, 50.5, 47.9, 44.4, 41.8, 38.0, 35.8, 31.6, 29.7, 29.3, 26.4, 25.7, 22.4, 21.6, 18.3, 13.8, 9.6. IR (film) 3030, 2945, 1741, 1639, 1375, 1124, 1072, 921, 866, 557. HRMS (ESI) Calcd for $C_{27}H_{42}O_4S_3SiNa$ (M+Na$^+$) 577.1907, found 577.1902.

Synthesis of compound **8**

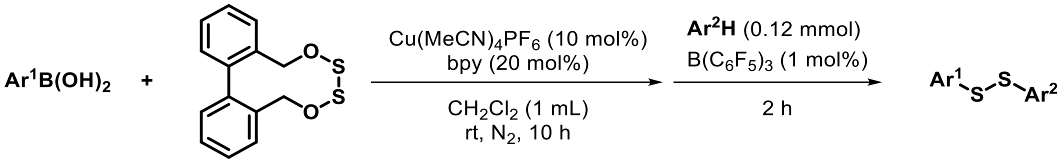

To a Schlenk tube were added arylboronic acid (0.15 mmol, 1.5 equiv), **1f** (29 mg, 0.10 mmol, 1 equiv), $Cu(MeCN)_4PF_6$ (3.7 mg, 0.01 mmol, 10 mol%), 2,2'-bpy (3.1 mg, 0.02 mmol, 20 mol%), and redistilled CH_2Cl_2 (1 mL), the mixture was stirred at r.t. for 24 hours under N_2 atmosphere. After **1f** was consumed, the mixture was filtered through a pad of silica gel. The Ar^2H (0.12 mmol, 1.2 equiv) and $B(C_6F_5)_3$ (0.5 mg, 0.001 mmol, 1 mol%) was added to the mixture. The mixture was stirred at r.t. for 2 hours under air before it was concentrated under vacuum. Purification by column chromatography afforded the desired product **8**.

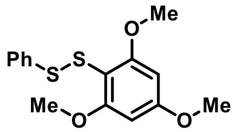

8a

The reaction of phenylboronic acid (18.3 mg, 0.15 mmol, 1.5 equiv), **1f** (29 mg, 0.10 mmol, 1 equiv), $Cu(MeCN)_4PF_6$ (3.7 mg, 0.01 mmol, 10 mol%) and 2,2'-bpy (3.1 mg, 0.02 mmol, 20 mol%) in redistilled CH_2Cl_2 (1 mL) at r.t. under N_2 for 10 hours, then 1,3,5-trimethoxybenzene (20.1 mg, 0.12 mmol, 1.2 equiv) and $B(C_6F_5)_3$ (0.5 mg, 0.001 mmol, 1 mol%) at r.t. for 2 hours afforded compound **8a** in 56% yield as a colorless oil by chromatography on silica gel eluting with PE/EA (20:1). R_f (10%

EA/hexane) = 0.3. ^{1}H NMR (400 MHz, $CDCl_3$) δ 7.56 – 7.51 (m, 2H), 7.24 – 7.17 (m, 2H), 7.11 (t, *J* = 7.3 Hz, 1H), 6.00 (s, 2H), 3.73 (s, 3H), 3.65 (s, 6H). ^{13}C NMR (100 MHz, $CDCl_3$) δ 163.1, 162.3, 138.8, 129.0, 128.4, 126.6, 104.6, 91.0, 55.9, 55.4. IR (film) 2940, 2837, 1580, 1466, 1410, 1337, 1227, 1159, 1088, 812, 740. HRMS (EI) Calcd for $C_{15}H_{16}O_3S_2$ 308.0541, found 308.0537.

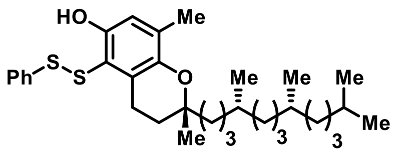

8b

The reaction of phenylboronic acid (18.3 mg, 0.15 mmol, 1.5 equiv), **1f** (29 mg, 0.10 mmol, 1 equiv), $Cu(MeCN)_4PF_6$ (3.7 mg, 0.01 mmol, 10 mol%) and 2,2'-bpy (3.1 mg, 0.02 mmol, 20 mol%) in redistilled CH_2Cl_2 (1 mL) at r.t. under N_2 for 10 hours, then (+)-δ-tocopherol (48.2 mg, 0.12 mmol, 1.2 equiv) and $B(C_6F_5)_3$ (0.5 mg, 0.001 mmol, 1 mol%) at r.t. under N_2 for 5 hours afforded compound **8b** in 59% yield as a colorless oil by chromatography on silica gel eluting with PE/EA (5:1). R_f (20% EA/hexane) = 0.3. ^{1}H NMR (400 MHz, $CDCl_3$) δ 7.43 (d, *J* = 6.5 Hz, 2H), 7.29 – 7.20 (m, 3H), 6.57 (s, 1H), 5.74 (s, 1H), 2.62 – 2.43 (m, 2H), 2.07 (s, 3H), 1.64 – 1.53 (m, 2H), 1.50 – 1.14 (m, 15H), 1.10 (s, 3H), 1.09 – 0.94 (m, 6H), 0.82 – 0.74 (m, 12H). ^{13}C NMR (100 MHz, $CDCl_3$) δ 150.4, 146.1, 137.1, 133.1, 131.9, 129.2, 129.2, 124.0, 116.5, 115.0, 75.2, 39.8, 39.4, 37.5, 37.5, 37.4, 37.3, 32.8, 32.7, 31.3, 28.0, 24.8, 24.5, 23.8, 22.7, 22.6, 22.0, 21.0, 19.8, 19.7, 16.6. IR (film) 2951, 2924, 1460, 1377, 1221, 1151, 1076, 1038, 744. HRMS (EI) Calcd for $C_{33}H_{50}O_2S_2$ 542.3252, found 542.3248.

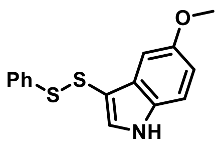

8c

The reaction of phenylboronic acid (18.3 mg, 0.15 mmol, 1.5 equiv), **1f** (29 mg, 0.10 mmol, 1 equiv), $Cu(MeCN)_4PF_6$ (3.7 mg, 0.01 mmol, 10 mol%) and 2,2'-bpy (3.1 mg, 0.02 mmol, 20 mol%) in redistilled CH_2Cl_2 (1 mL) at r.t. under N_2 for 10 hours, then 5-methoxyindole (17.6 mg, 0.12 mmol, 1.2 equiv) and $B(C_6F_5)_3$ (0.5 mg, 0.001 mmol, 1 mol%) at r.t. for 2 hours afforded compound **8c** in 76% yield as a colorless oil by chromatography on silica gel eluting with PE/EA (10:1). R_f (5% EA/hexane) = 0.3. ^{1}H NMR (400 MHz, $CDCl_3$) δ 8.09 (s, 1H), 7.53 – 7.46 (m, 1H), 7.26 – 7.21 (m, 1H), 7.19 – 7.15 (m, 1H), 7.12 (d, *J* = 8.8 Hz, 1H), 6.87 (d, *J* = 2.2 Hz, 1H), 6.77 (dd, *J* = 8.8, 2.4 Hz, 1H), 3.62 (s, 1H). ^{13}C NMR (100 MHz, $CDCl_3$) δ 155.2, 138.8, 131.0, 130.6, 130.2, 129.2, 128.9, 127.4, 113.9, 112.4, 108.1, 100.9, 55.6. IR (film) 3416, 2829, 1622, 1581, 1436, 1286, 1207, 1168, 920, 802, 742, 690. HRMS (EI) Calcd for $C_{15}H_{13}NOS_2$ 287.0439, found 287.0434.

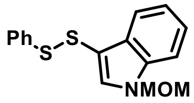

8d

The reaction of phenylboronic acid (18.3 mg, 0.15 mmol, 1.5 equiv), **1f** (29 mg, 0.10 mmol, 1 equiv), $Cu(MeCN)_4PF_6$ (3.7 mg, 0.01 mmol, 10 mol%) and 2,2'-bpy (3.1 mg, 0.02 mmol, 20 mol%) in redistilled CH_2Cl_2 (1 mL) at r.t. under N_2 for 10

hours, then 1-(methoxymethyl)-1H-indole (19.3 mg, 0.12 mmol, 1.2 equiv) and $B(C_6F_5)_3$ (0.5 mg, 0.001 mmol, 1 mol%) at r.t. for 2 hours afforded compound **8d** in 48% yield as a colorless oil by chromatography on silica gel eluting with PE/EA (20:1). R_f (5% EA/hexane) = 0.3. ^{1}H NMR (400 MHz, $CDCl_3$) δ 7.56 (d, *J* = 7.9 Hz, 1H), 7.45 (d, *J* = 7.7 Hz, 2H), 7.39 (d, *J* = 8.1 Hz, 1H), 7.27 – 7.17 (m, 5H), 7.15 – 7.09 (m, 1H), 5.29 (s, 2H), 3.11 (s, 3H). ^{13}C NMR (100 MHz, $CDCl_3$) δ 138.2, 136.8, 133.5, 130.3, 129.5, 128.9, 127.6, 123.4, 121.5, 120.1, 110.4, 108.1, 77.6, 56.0. IR (film) 3053, 2949, 1502, 1458, 1335, 1234, 1153, 1111, 1086, 972, 743, 688. HRMS (EI) Calcd for $C_{16}H_{15}NOS_2$ 301.0595, found 301.0590.

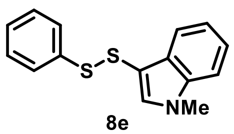

8e

The reaction of phenylboronic acid (18.3 mg, 0.15 mmol, 1.5 equiv), **1f** (29 mg, 0.10 mmol, 1 equiv), $Cu(MeCN)_4PF_6$ (3.7 mg, 0.01 mmol, 10 mol%) and 2,2'-bpy (3.1 mg, 0.02 mmol, 20 mol%) in redistilled CH_2Cl_2 (1 mL) at r.t. under N_2 for 10 hours, then 1-methyl-1H-indole (15.7 mg, 0.12 mmol, 1.2 equiv) and $B(C_6F_5)_3$ (0.5 mg, 0.001 mmol, 1 mol%) at r.t. for 2 hours afforded compound **8e** in 48% yield as a white solid by chromatography on silica gel eluting with PE/EA (20:1). R_f (5% EA/hexane) = 0.3. ^{1}H NMR (400 MHz, $CDCl_3$) δ 7.52 (d, *J* = 7.9 Hz, 1H), 7.49 – 7.45 (m, 2H), 7.26 – 7.20 (m, 3H), 7.20 – 7.15 (m, 2H), 7.11 (s, 1H), 7.10 – 7.06 (m, 1H), 3.65 (s, 3H). ^{13}C NMR (100 MHz, $CDCl_3$) δ 138.5, 137.3, 134.5, 129.9, 129.2, 128.9, 127.3, 122.7, 120.7, 119.9, 109.7, 106.2, 33.1. IR (film) 2976, 1580, 1475, 1458, 1332, 1242, 1051, 881, 741, 688. HRMS (EI) Calcd for $C_{15}H_{13}NS_2$ 271.0489, found 271.0490.

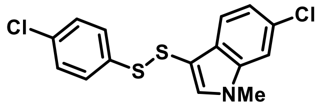

8f

The reaction of (4-chlorophenyl)boronic acid (23.4 mg, 0.15 mmol, 1.5 equiv), **1f** (29 mg, 0.10 mmol, 1 equiv), $Cu(MeCN)_4PF_6$ (3.7 mg, 0.01 mmol, 10 mol%) and 2,2'-bpy (3.1 mg, 0.02 mmol, 20 mol%) in redistilled CH_2Cl_2 (1 mL) at r.t. under N_2 for 10 hours, then 6-chloro-1-methyl-1H-indole (19.8 mg, 0.12 mmol, 1.2 equiv) and $B(C_6F_5)_3$ (0.5 mg, 0.001 mmol, 1 mol%) at r.t. for 2 hours afforded compound **8f** in 65% yield as a white solid by chromatography on silica gel eluting with PE/EA (20:1→15:1). R_f (5% EA/hexane) = 0.2. ^{1}H NMR (400 MHz, $CDCl_3$) δ 7.39 – 7.36 (m, 2H), 7.35 – 7.33 (m, 1H), 7.21 (d, *J* = 1.7 Hz, 1H), 7.20 (d, *J* = 1.9 Hz, 1H), 7.18 (dd, *J* = 4.4, 1.6 Hz, 1H), 7.07 (s, 1H), 7.05 (dd, *J* = 8.5, 1.7 Hz, 1H), 3.62 (s, 3H). ^{13}C NMR (100 MHz, $CDCl_3$) δ 137.7, 136.9, 135.1, 133.7, 131.6, 129.1, 129.0, 127.6, 121.5, 120.8, 109.9, 106.4, 33.2. IR (film) 2933, 1606, 1504, 1472, 1460, 1418, 1387, 1327, 1232, 1090, 1065, 1011, 974, 806, 642, 598. HRMS (EI) Calcd for $C_{15}H_{11}Cl_2NS_2$ 338.9710, found 338.9713.

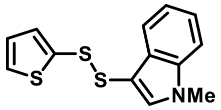

8g

The reaction of thiophen-2-ylboronic acid (19.2 mg, 0.15 mmol, 1.5 equiv), **1f** (29 mg, 0.10 mmol, 1 equiv), $Cu(MeCN)_4PF_6$ (3.7 mg, 0.01 mmol, 10 mol%) and 2,2'-bpy (3.1 mg, 0.02 mmol, 20 mol%) in redistilled CH_2Cl_2 (1 mL) at r.t. under N_2 for 10 hours, then 1-methyl-1H-indole (15.7 mg, 0.12 mmol, 1.2 equiv) and $B(C_6F_5)_3$ (0.5 mg, 0.001 mmol, 1 mol%) at r.t. for 2 hours afforded compound **8g** in 56% yield as a colorless oil by chromatography on silica gel eluting with PE/EA (20:1). R_f (5% EA/hexane) = 0.3. ^1H NMR (400 MHz, $CDCl_3$) δ 7.56 (d, J = 7.9 Hz, 1H), 7.36 (dd, J = 5.3, 1.1 Hz, 1H), 7.27 (d, J = 8.1 Hz, 1H), 7.24 – 7.19 (m, 1H), 7.15 – 7.11 (m, 1H), 7.11 – 7.08 (m, 1H), 6.96 (dd, J = 3.5, 1.2 Hz, 1H), 6.88 (dd, J = 5.3, 3.6 Hz, 1H), 3.72 (s, 3H). ^{13}C NMR (100 MHz, $CDCl_3$) δ 137.6, 137.4, 135.3, 134.7, 131.3, 129.3, 127.6, 122.8, 120.8, 119.9, 109.7, 106.2, 33.2. IR (film) 3107, 2816, 1626, 1504, 1333, 1242, 1215, 1144, 1113, 848, 742, 706. HRMS (EI) Calcd for $C_{13}H_{11}NS_3$ 277.0054, found 277.0059.

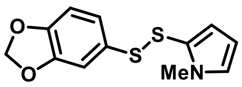

8h

The reaction of 1,3-benzodioxole-5-boronic acid (24.9 mg, 0.15 mmol, 1.5 equiv), **1f** (29 mg, 0.10 mmol, 1 equiv), $Cu(MeCN)_4PF_6$ (3.7 mg, 0.01 mmol, 10 mol%) and 2,2'-bpy (3.1 mg, 0.02 mmol, 20 mol%) in redistilled CH_2Cl_2 (1 mL) at r.t. under N_2 for 10 hours, then 1-methyl-1H-indole (15.7 mg, 0.12 mmol, 1.2 equiv) and $B(C_6F_5)_3$ (0.5 mg, 0.001 mmol, 1 mol%) at r.t. for 2 hours afforded compound **8h** in 55% yield as a yellow oil by chromatography on silica gel eluting with PE/EA (10:1). R_f (10% EA/hexane) = 0.4. ^1H NMR (400 MHz, $CDCl_3$) δ 6.93 – 6.89 (m, 1H), 6.84 – 6.79 (m, 1H), 6.76 (s, 1H), 6.67 – 6.63 (m, 1H), 6.29 – 6.24 (m, 1H), 6.04 – 5.99 (m, 1H), 5.93 – 5.88 (m, 2H), 3.54 (s, 3H). ^{13}C NMR (100 MHz, $CDCl_3$) δ 148.5, 148.0, 129.4, 127.3, 127.1, 122.0, 119.7, 113.1, 108.5, 108.4, 101.5, 34.3. IR (film) 3003, 2889, 1716, 1475, 1363, 1290, 1039, 933, 806, 731, 612. HRMS (ESI) Calcd for $C_{12}H_{12}NO_2S_2$ $(M+H^+)$ 266.0304, found 266.0302.

Optimization of Polysulfuration with Mercaptan.

Supplementary Table 3. Optimization of disulfurating reagent with mercaptan[a][b].

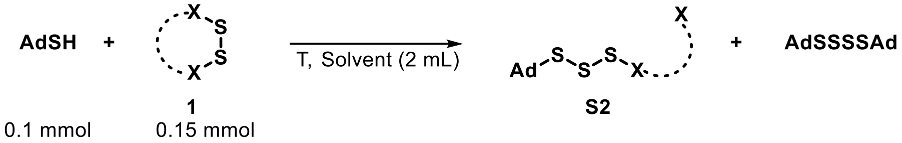

entry	**1**	Solvent	T (°C)	Yield of **S2**	Yield of tetrasulfide
1	**1a**	CH_2Cl_2	rt	12%	82%

2	**1b**	CH_2Cl_2	rt	22%	70%
3	**1d**	CH_2Cl_2	rt	63%	30%
4	**1d**	CH_2Cl_2	-78	72%	18%
5	**1d**	MeOH	-78	80%	9%
6	**1d**	Hexane	-78	50%	45%
7	**1d**	Acetone	-78	50%	35%
8	**1d**	THF	-78	54%	37%
9	**1d**	EA	-78	36%	Undetected
10	**1d**	DCE	-78	70%	20%
11	**1e**	MeOH	-78	70%	22%
12	**1f**	MeOH	-78	64%	30%
13	**1d**	MeOH	-78	76%[c]	10%

[a]Condition: AdSH (0.1 mmol), **1** (0.15 mmol), Solvent (2 mL), T, under air. [b]Isolated yield. [c]0.12 mmol of **1d**.

To a solution of disulfurating reagent **1** (0.15 mmol, 1.5 equiv) in solvent (1 mL) was added 1-adamantylthiol (16.8 mg, 0.1 mmol, 1.0 equiv) in solvent (1 mL) dropwise at -78 °C, then the mixture was stirred at -78°C for 30 min before it was concentrated under vacuum. Purification by column chromatography afforded the desired product **S2**.

Synthesis of compound **S2**

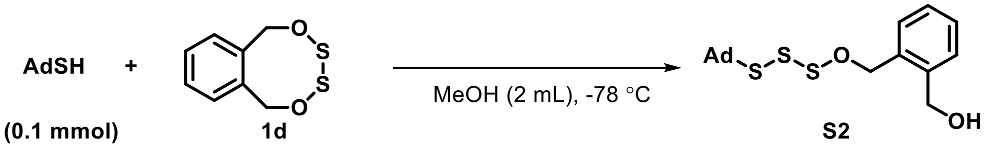

To a solution of **1d** (24.0 mg, 0.12 mmol, 1.2 equiv) in MeOH (1 mL) was added 1-adamantylthiol (16.8 mg, 0.1 mmol, 1.0 equiv) in MeOH (1 mL) dropwise at -78 °C, then the mixture was stirred at -78°C for 30 min before it was concentrated under vacuum. Purification of the residue by flash column chromatography (silica gel, PE/DCM = 4:1→2:1) yielded the **S2** (27.2 mg, 76%) as a colorless oil. R_f (20% EA/hexane) = 0.3. ^{1}H NMR (400 MHz, $CDCl_3$) δ 7.36 (d, J = 7.5 Hz, 1H),

7.29 (dd, J = 6.7, 4.9 Hz, 2H), 7.25 – 7.20 (m, 1H), 4.94 (s, 2H), 4.66 (s, 2H), 2.01 (s, 3H), 1.83 (s, 6H), 1.60 (q, J = 12.1 Hz, 6H). ^{13}C NMR (100 MHz, $CDCl_3$) δ 140.0, 134.4, 130.6, 129.4, 129.0, 128.0, 63.0, 50.6, 42.8, 36.0, 29.9. IR (film) 3691, 2964, 2904, 2848, 1294, 1242, 1180, 1039, 906, 729, 684. HRMS (ESI) Calcd for $C_{18}H_{25}O_2S_3$ (M+H$^+$) 369.1011, found 369.1012.

General Procedure and Spectra Data of Polysulfuration with Mercaptan.

Synthesis of compound **9**

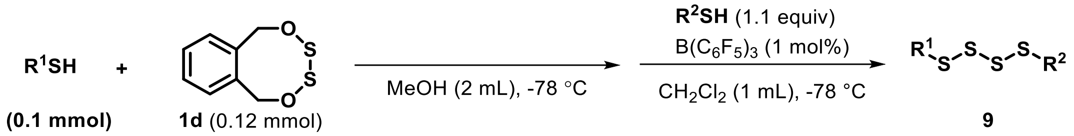

To a solution of **1d** (24.0 mg, 0.12 mmol, 1.2 equiv) in MeOH (1 mL) was added thiol (0.1 mmol, 1.0 equiv) in MeOH (1 mL) dropwise at -78 °C, then the mixture was stirred at -78°C for 30 min before MeOH was removed under vacuum. CH_2Cl_2 (1 mL), another thiol (0.11 mmol, 1.1 equiv) and $B(C_6F_5)_3$ (0.5 mg, 0.001 mmol, 1 mol%) was added to the mixture at r.t. for 4 hours under air before it was concentrated under vacuum. Purification by column chromatography afforded the desired product **9**.

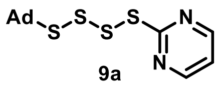
9a

The reaction of **1d** (24.0 mg, 0.12 mmol, 1.2 equiv) and 1-adamantylthiol (16.8 mg, 0.1 mmol, 1.0 equiv) in MeOH (2 mL) at -78 °C for 30 min, then pyrimidine-2-thiol (12.3 mg, 0.11 mmol, 1.1 equiv) and $B(C_6F_5)_3$ (0.5 mg, 0.001 mmol, 1 mol%) in CH_2Cl_2 (1 mL) at r.t. for 4 hours afforded compound **9a** in 70% yield as a white solid by chromatography on silica gel eluting with PE/EA (10:1). R_f (20% EA/hexane) = 0.3. ^{1}H NMR (400 MHz, $CDCl_3$) δ 8.60 (d, J = 4.8 Hz, 2H), 7.07 (t, J = 4.8 Hz, 1H), 2.04 (s, 3H), 1.91 – 1.81 (m, 6H), 1.68 – 1.57 (m, 6H). ^{13}C NMR (100 MHz, $CDCl_3$) δ 170.4, 158.0, 118.3, 51.1, 42.8, 36.0, 30.0. IR (film) 2904, 2848, 1556, 1377, 1296, 1167, 769, 742. HRMS (EI) Calcd for $C_{14}H_{18}N_2S_4$ 342.0353, found 342.0357.

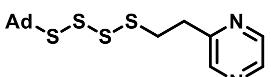
9b

The reaction of **1d** (24.0 mg, 0.12 mmol, 1.2 equiv) and 1-adamantylthiol (16.8 mg, 0.1 mmol, 1.0 equiv) in MeOH (2 mL) at -78 °C for 30 min, then 2-(pyrazin-2-yl)ethane-1-thiol (15.4 mg, 0.11 mmol, 1.1 equiv) and $B(C_6F_5)_3$ (0.5 mg, 0.001 mmol, 1 mol%) in CH_2Cl_2 (1 mL) at r.t. for 4 hours afforded compound **9b** in 50% yield as a colorless oil by chromatography on silica gel eluting with PE/EA (5:1). R_f (20% EA/hexane) = 0.2. ^{1}H NMR (400 MHz, $CDCl_3$) δ 8.46 (s, 2H), 8.37 (s, 1H), 3.39 – 3.18 (m, 4H), 2.04 (s, 3H), 1.85 (s, 6H), 1.68 – 1.56 (m, 6H). ^{13}C NMR (100 MHz, $CDCl_3$) δ 155.0, 145.1, 144.3, 142.8, 51.1, 42.8, 37.7,

36.0, 34.7, 29.9. IR (film) 2972, 1468, 1377, 1340, 1097, 1055, 887, 622. HRMS (ESI) Calcd for $C_{16}H_{23}N_2S_4$ (M+H$^+$) 371.0739, found 371.0734.

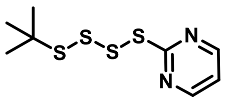

9c

The reaction of **1d** (24.0 mg, 0.12 mmol, 1.2 equiv) and 2-methylpropane-2-thiol (9.0 mg, 0.1 mmol, 1.0 equiv) in MeOH (2 mL) at -78 °C for 30 min, then pyrimidine-2-thiol (12.3 mg, 0.11 mmol, 1.1 equiv) and $B(C_6F_5)_3$ (0.5 mg, 0.001 mmol, 1 mol%) in CH_2Cl_2 (1 mL) at r.t. for 4 hours afforded compound **9c** in 65% yield as a white solid by chromatography on silica gel eluting with PE/EA (5:1). R_f (20% EA/hexane) = 0.2. ^{1}H NMR (400 MHz, $CDCl_3$) δ 8.59 (d, *J* = 4.8 Hz, 2H), 7.07 (t, *J* = 4.8 Hz, 1H), 1.35 (s, 9H). ^{13}C NMR (100 MHz, $CDCl_3$) δ 170.3, 158.0, 118.3, 49.4, 30.2. IR (film) 2982, 1641, 1070, 899, 796, 680, 565. HRMS (ESI) Calcd for $C_8H_{13}N_2S_4$ (M+H$^+$) 264.9956, found 264.9954.

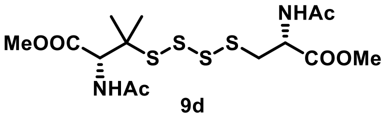

9d

The reaction of **1d** (24.0 mg, 0.12 mmol, 1.2 equiv) and methyl 2-acetamido-3-mercapto-3-methylbutanoate (20.5 mg, 0.1 mmol, 1.0 equiv) in MeOH (2 mL) at -78 °C for 30 min, then methyl acetylcysteinate (19.5 mg, 0.11 mmol, 1.1 equiv) and $B(C_6F_5)_3$ (1.0 mg, 0.002 mmol, 2 mol%) in CH_2Cl_2 (1 mL) at r.t. for 4 hours afforded compound **9d** in 53% yield as a white solid by chromatography on silica gel eluting with PE/EA (5:1→2:1). R_f (30% EA/hexane) = 0.2. ^{1}H NMR (400 MHz, $CDCl_3$) δ 6.52 (dd, *J* = 15.2, 8.0 Hz, 2H), 4.88 (dd, *J* = 12.4, 5.3 Hz, 1H), 4.73 (d, *J* = 8.7 Hz, 1H), 3.72 (s, 3H), 3.69 (s, 3H), 3.40 (ddd, *J* = 42.6, 14.3, 5.3 Hz, 2H), 2.01 (s, 3H), 2.01 (s, 3H), 1.42 (s, 3H), 1.38 (s, 3H). ^{13}C NMR (100 MHz, $CDCl_3$) δ 170.7, 170.4, 170.2, 170.1, 58.9, 53.6, 52.9, 52.4, 52.0, 41.0, 26.3, 25.0, 23.1, 23.1. IR (film) 3294, 2949, 1740, 1645, 1529, 1435, 1371, 1215, 1126, 1032, 982, 659, 584. HRMS (ESI) Calcd for $C_{14}H_{25}N_2O_6S_4$ (M+H$^+$) 445.0590, found 445.0595.

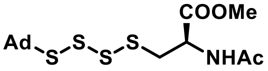

9e

The reaction of **1d** (24.0 mg, 0.12 mmol, 1.2 equiv) and 1-adamantylthiol (16.8 mg, 0.1 mmol, 1.0 equiv) in MeOH (2 mL) at -78 °C for 30 min, then methyl acetylcysteinate (19.5 mg, 0.11 mmol, 1.1 equiv) and $B(C_6F_5)_3$ (1.0 mg, 0.002 mmol, 2 mol%) in CH_2Cl_2 (1 mL) at r.t. for 4 hours afforded compound **9e** in 52% yield as a colorless oil by chromatography on silica gel eluting with PE/EA (5:1). R_f (20% EA/hexane) = 0.2. ^{1}H NMR (400 MHz, $CDCl_3$) δ 6.33 (d, *J* = 7.1 Hz, 1H), 4.89 (dt, *J* = 7.6, 5.0 Hz, 1H), 3.72 (s, 3H), 3.45 (dd, *J* = 14.2, 4.6 Hz, 1H), 3.37 (dd, *J* = 14.2, 5.4 Hz, 1H), 2.05 (s, 3H), 2.00 (s, 3H), 1.85 (d, *J* = 2.3 Hz, 6H), 1.70 – 1.58 (m, 7H). ^{13}C NMR (100 MHz, $CDCl_3$) δ 170.7, 169.8, 52.8, 51.8, 51.2, 42.8, 41.3, 36.0, 29.9, 23.2. IR (film) 3279, 2904, 1745, 1657, 1537, 1450, 1371, 1296, 1171, 1038, 976, 684, 588. HRMS (EI) Calcd for $C_{16}H_{25}NO_3S_4$ 407.0717, found 407.0713.

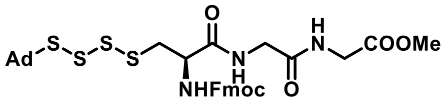

9f

The reaction of **1d** (24.0 mg, 0.12 mmol, 1.2 equiv) and 1-adamantylthiol (16.8 mg, 0.1 mmol, 1.0 equiv) in MeOH (2 mL) at -78 °C for 30 min, then tripeptide (51.8 mg, 0.11 mmol, 1.1 equiv) and $B(C_6F_5)_3$ (1.0 mg, 0.002 mmol, 2 mol%) in CH_2Cl_2 (1 mL) at r.t. for 4 hours afforded compound **9f** in 40% yield as a white solid by chromatography on silica gel eluting with DCM/MeOH (100:1). R_f (5% MeOH/DCM) = 0.6. ^{1}H NMR (400 MHz, $CDCl_3$) δ 7.69 (d, J = 7.6 Hz, 2H), 7.53 (t, J = 6.6 Hz, 2H), 7.33 (t, J = 7.5 Hz, 2H), 7.24 (t, J = 7.2 Hz, 2H), 7.07 (s, 1H), 6.89 (s, 1H), 5.83 (dd, J = 33.6, 5.8 Hz, 1H), 4.50 (s, 1H), 4.37 (d, J = 6.0 Hz, 2H), 4.16 (t, J = 6.7 Hz, 1H), 4.06 – 3.85 (m, 4H), 3.62 (s, 3H), 3.29 (dd, J = 33.9, 6.6 Hz, 2H), 2.02 (s, 3H), 1.84 (s, 6H), 1.61 (s, 6H). ^{13}C NMR (100 MHz, $CDCl_3$) δ 170.6, 170.4, 170.1, 168.8, 143.6, 141.3, 127.8, 127.1, 125.1, 120.0, 67.5, 52.4, 51.4, 47.2, 43.2, 42.8, 42.7, 42.5, 41.1, 36.0, 29.9. IR (film) 3572, 2972, 2881, 1456, 1419, 1379, 1327, 1275, 1088, 1045, 879. HRMS (ESI) Calcd for $C_{33}H_{40}N_3O_6S_4$ (M+H$^+$) 702.1794, found 702.1793.

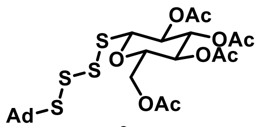

9g

The reaction of **1d** (24.0 mg, 0.12 mmol, 1.2 equiv) and 1-adamantylthiol (16.8 mg, 0.1 mmol, 1.0 equiv) in MeOH (2 mL) at -78 °C for 30 min, then glucosinolate (40.0 mg, 0.11 mmol, 1.1 equiv) and $B(C_6F_5)_3$ (1.0 mg, 0.002 mmol, 2 mol%) in CH_2Cl_2 (1 mL) at r.t. for 4 hours afforded compound **9g** in 57% yield as a viscous gel by chromatography on silica gel eluting with PE/EA (4:1). R_f (30% EA/hexane) = 0.3. ^{1}H NMR (400 MHz, $CDCl_3$) δ 5.27 – 5.04 (m, 3H), 4.73 (d, J = 9.5 Hz, 1H), 4.23 (dd, J = 12.4, 4.7 Hz, 1H), 4.13 (dd, J = 12.4, 2.2 Hz, 1H), 3.73 (ddd, J = 9.9, 4.5, 2.3 Hz, 1H), 2.06 (s, 3H), 2.03 (s, 3H), 1.98 (s, 3H), 1.97 (s, 3H), 1.95 (s, 3H), 1.85 (d, J = 2.3 Hz, 6H), 1.69 – 1.59 (m, 6H). ^{13}C NMR (100 MHz, $CDCl_3$) δ 170.6, 170.2, 169.3, 169.2, 88.2, 76.3, 73.9, 69.7, 68.0, 62.0, 51.1, 42.7, 36.0, 29.9, 20.8, 20.8, 20.7, 20.7, 20.6, 20.6. IR (film) 2906, 2851, 1747, 1452, 1365, 1298, 1211, 1035, 912, 737, 684, 598. HRMS (ESI) Calcd for $C_{24}H_{34}O_9S_4Na$ (M+Na$^+$) 617.0978, found 617.0970.

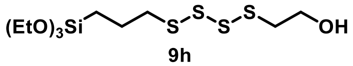

9h

The reaction of **1d** (24.0 mg, 0.12 mmol, 1.2 equiv) and 3-(triethoxysilyl)propane-1-thiol (23.8 mg, 0.1 mmol, 1.0 equiv) in MeOH (2 mL) at -78 °C for 30 min, then 2-mercaptoethan-1-ol (8.6 mg, 0.11 mmol, 1.1 equiv) and $B(C_6F_5)_3$ (0.5 mg, 0.001 mmol, 1 mol%) in CH_2Cl_2 (1 mL) at r.t. for 4 hours afforded compound **9h** in 53% yield as a colorless oil by chromatography on silica gel eluting with PE/EA (5:1). R_f (30% EA/hexane) = 0.4. ^{1}H NMR (400 MHz, $CDCl_3$) δ 3.91 (t, J = 5.8 Hz, 2H), 3.76 (q, J = 7.0 Hz, 6H), 3.05 (t, J = 5.8 Hz, 2H), 2.92 (t, J = 7.2 Hz, 2H), 1.84 (dt, J = 15.6, 7.7 Hz, 2H),

1.17 (t, *J* = 7.0 Hz, 9H), 0.73 – 0.66 (m, 2H). ^{13}C NMR (100 MHz, $CDCl_3$) δ 60.2, 58.5, 42.2, 42.0, 22.7, 18.3, 9.6. IR (film) 3379, 2980, 1637, 1089, 1047, 879, 682, 669. IR (film) 3523, 2980, 1637, 1089, 1047, 879, 721, 682, 669. HRMS (ESI) Calcd for $C_{11}H_{26}O_4S_4SiNa$ ($M+H^+$) 401.0375, found 401.0373.

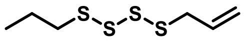
9i

The reaction of **1d** (24.0 mg, 0.12 mmol, 1.2 equiv) and propane-1-thiol (7.6 mg, 0.1 mmol, 1.0 equiv) in MeOH (2 mL) at -78 °C for 30 min, remove symmetrical tetrasulfide and **1d** by column chromatography, then prop-2-ene-1-thiol (8.1 mg, 0.11 mmol, 1.1 equiv) and $B(C_6F_5)_3$ (0.5 mg, 0.001 mmol, 1 mol%) in CH_2Cl_2 (1 mL) at r.t. for 4 hours afforded compound **9i** in 48% yield as a volatile and stench colorless oil by chromatography on silica gel eluting with PE. R_f (1% EA/hexane) = 0.7. ^{1}H NMR (400 MHz, $CDCl_3$) δ 5.83 (ddt, *J* = 17.2, 9.9, 7.3 Hz, 1H), 5.18 (dd, *J* = 21.3, 5.5 Hz, 2H), 3.52 (d, *J* = 7.3 Hz, 2H), 2.86 (t, *J* = 7.2 Hz, 2H), 1.80 – 1.66 (m, 2H), 0.96 (t, *J* = 7.3 Hz, 3H). ^{13}C NMR (100 MHz, $CDCl_3$) δ 132.6, 119.5, 42.1, 41.4, 22.4, 13.1. IR (film) 3082, 2961, 2928, 2871, 1634, 1454, 1377, 1288, 1074, 1034, 984, 918, 858, 781, 719, 578. HRMS (EI) Calcd for $C_6H_{12}S_4$ 211.9822, found 211.9824.

Supplementary Discussion

Analysis of the synthesis of compound **3l**.

Substrates with tryptophan motif, such as **3l**, **3n** and **3o**, afforded a low yield of **S4** of 36%, inconsequently lower than the total yields of **3l**, **3n** and **3o**. The LC-MS detection of the first step demonstrated that cyclic disulfide intermediate **S10** was generated due to the nucleophilicity of 2-position on indole. In the second step, cyclic disulfide intermediate **S10** disappeared from LC-MS spectrum. Obviously, intermediate **S10** generating from nucleophilic cyclization of 2-position of indole brought about the unusual experimental results. In the first step, the homocoupling intermediate **S10** yielded symmetrical product **S11**. In the second step, intermediate **S4** and **S10** afforded product **3l** together, which created the distortion between the yields of two steps.

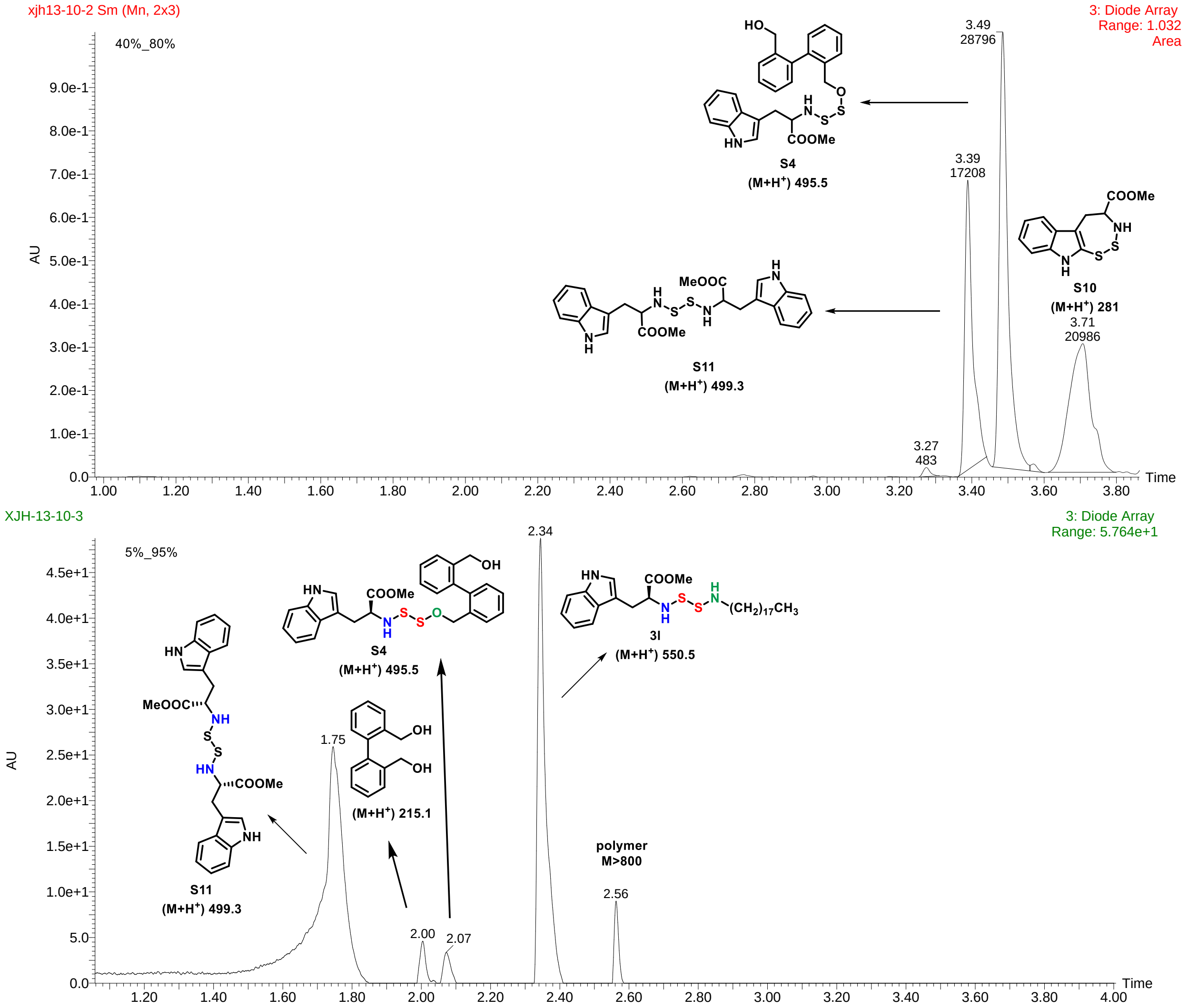

Supplementary Figure 1. LC-MS of first and second step of **3l**.

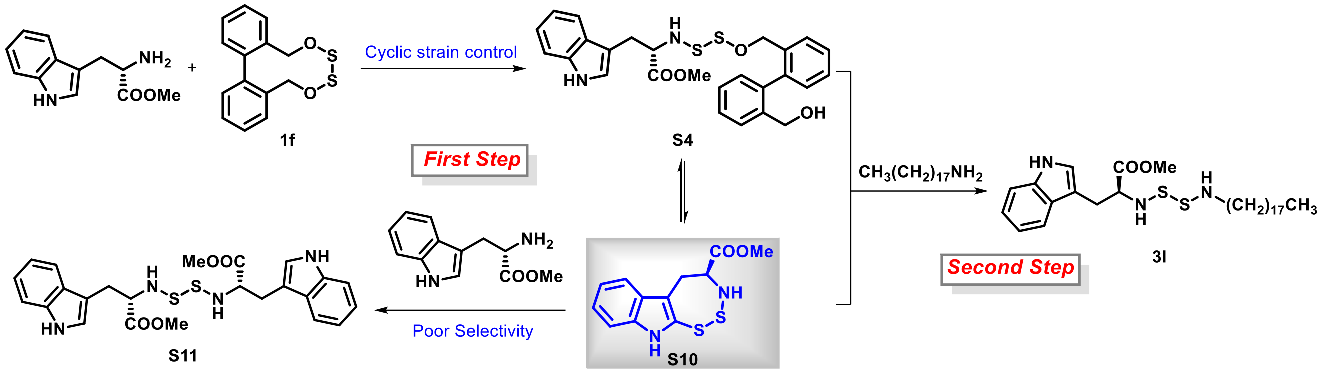

Supplementary Figure 2. Pathway of **3l** synthesis.

Methodology comparison between **1d**, **1f** and other reagents.

Synthesis of compound **9a** with S_2Cl_2.

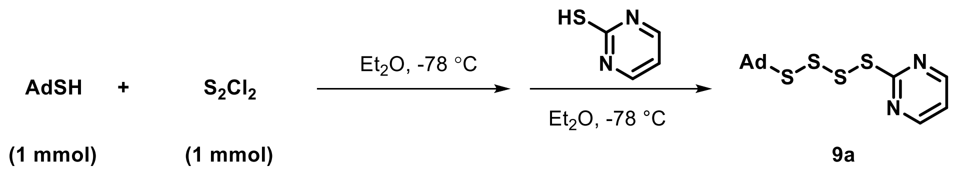

9a was prepared according to literature procedures[5]: To a solution of S_2Cl_2 (80 μL, 1 mmol, 1.0 equiv) in Et_2O (2.5 mL) was added 1-adamantylthiol (168 mg, 1 mmol, 1.0 equiv) and pyridine (79 mg, 1 mmol, 1.0 equiv) in Et_2O (1 mL) dropwise for 10 min at -78 °C. The solution was kept at −78 °C for 45 min. Then, a solution of pyrimidine-2-thiol and pyridine (79 mg, 1 mmol, 1.0 equiv) in Et_2O (1 mL) was added dropwise for 10 min at −78 °C. and then allowed to warm up to room temperature. Subsequently, the organic layers were washed with distilled water (three times) and dried over Na_2SO_4. The crude reaction was detected by LC-MS (Water corp. BEH C18 column, 2.1×150 mm, 1.7 μm). After evaporating the solvent, the product was purified as colorless oil (25 mg, yield ≈ 8%) by column chromatography on silica gel eluting with PE/EA (10:1). R_f (20% EA/hexane) = 0.3.

^{1}H NMR, comparative ^{1}H NMR and LC-MS between **S_2Cl_2** and **1d** was attached as follow. ^{1}H NMR of **9a** synthesis by S_2Cl_2 shows that the ratio of **9a** to **9a-S$_5$** is 2.5:1, much lower than 15:1 afforded by **1d**. UV detector of LC-MS exhibited that the ratio of **9a** to **9a-S$_5$** to **9a-S$_3$** given by S_2Cl_2 is 3.3:5:1, substantially different from 10:2:1 given by **1d**. Besides, there is a huge gap between the yields afforded by S_2Cl_2 and **1d** (8% vs 70%).

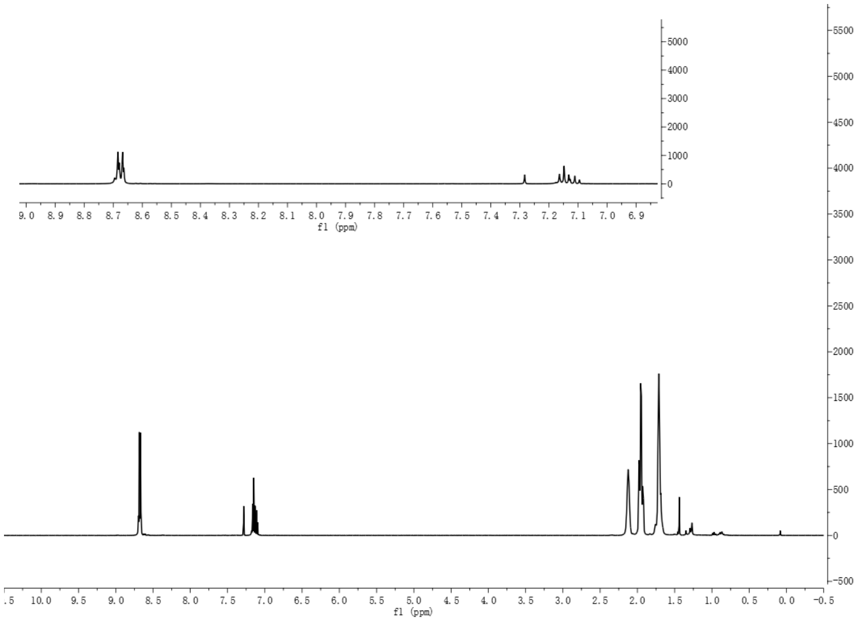

Supplementary Figure 3. ^{1}H NMR of isolated **9a** with S_2Cl_2.

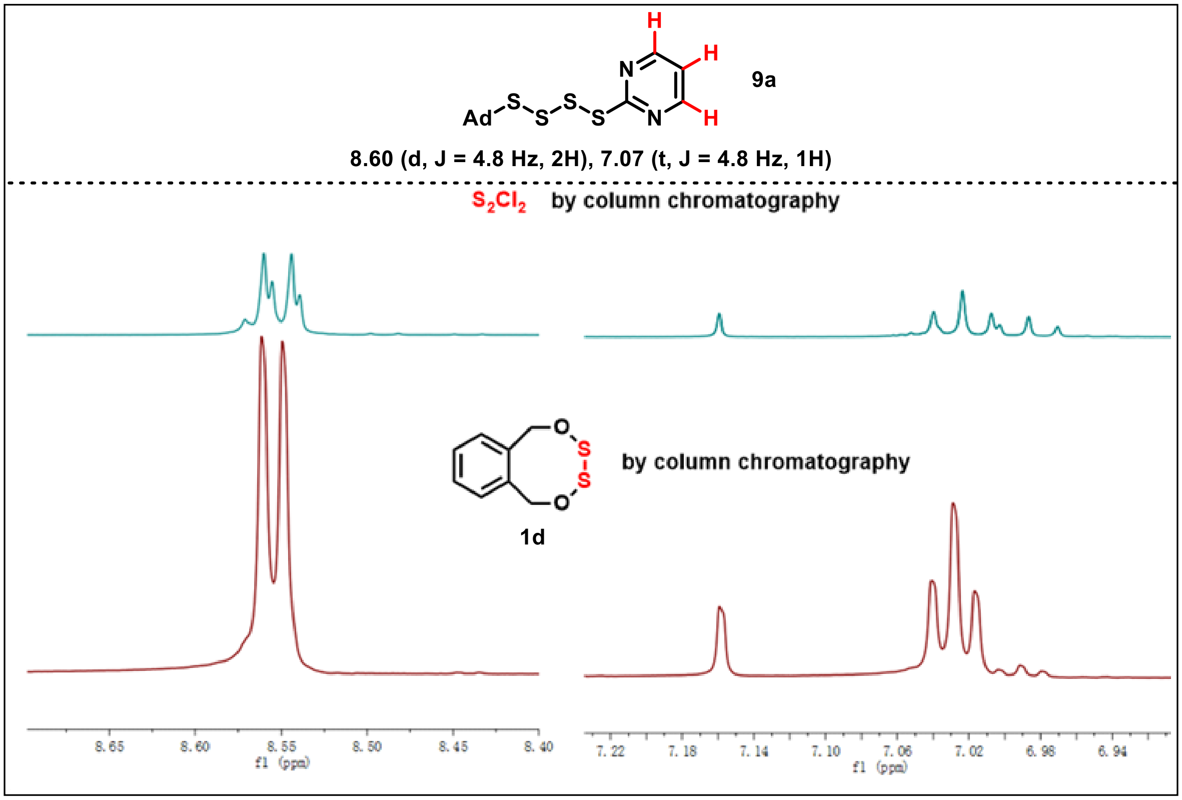

Supplementary Figure 4. Comparison of ^{1}H NMR between 1d and S_2Cl_2.

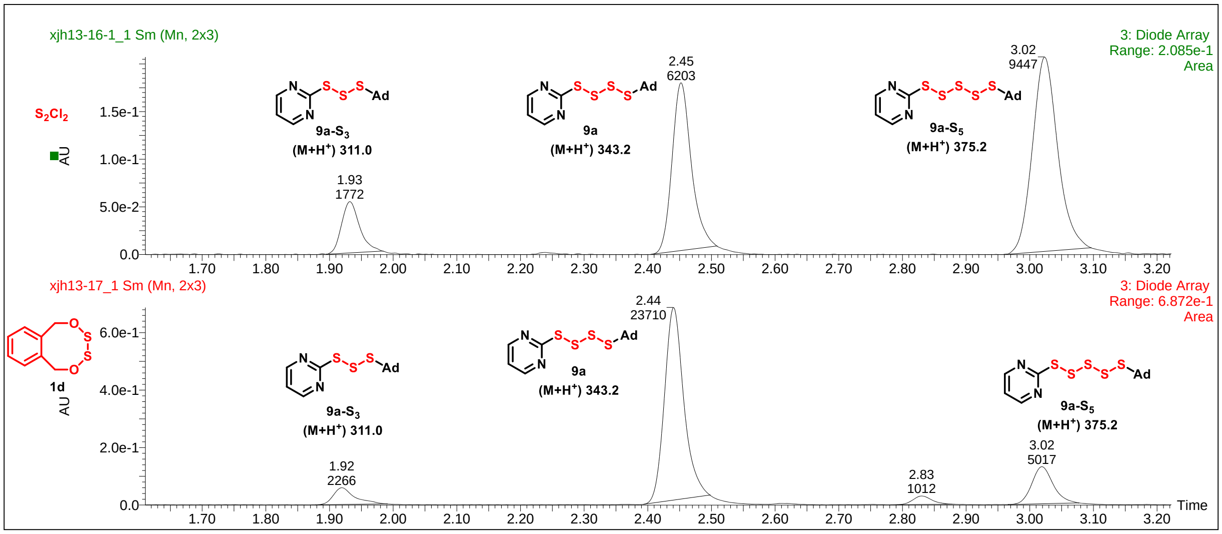

Supplementary Figure 5. Comparison of LC-MS between **1d** and S_2Cl_2.

Synthesis of compound **9h** with S_2Cl_2[5].

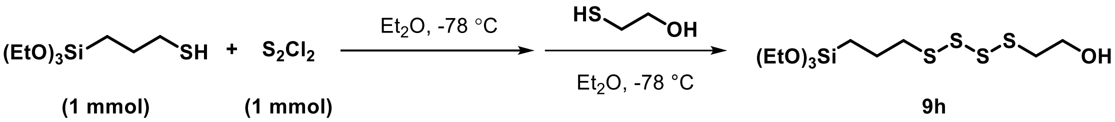

9h was prepared according to literature procedures[5]: To a solution of S_2Cl_2 (80 μL, 1 mmol, 1.0 equiv) in Et_2O (2.5 mL) was added 3-(triethoxysilyl)propane-1-thiol (238 mg, 1 mmol, 1.0 equiv) and pyridine (79 mg, 1 mmol, 1.0 equiv) in Et_2O (1 mL) dropwise for 10 min at -78 °C. The solution was kept at −78 °C for 45 min. Then, a solution of pyrimidine-2-thiol and pyridine (79 mg, 1 mmol, 1.0 equiv) in Et_2O (1 mL) was added dropwise for 10 min at −78 °C. and then allowed to warm up to room temperature. Subsequently, the organic layers were washed with distilled water (three times) and dried over Na_2SO_4. The crude reaction was detected by LC-MS (Water corp. BEH C18 column, 2.1×150 mm, 1.7 μm). After evaporating the solvent, the product was purified as colorless oil (25 mg) by column chromatography on silica gel eluting with PE/EA (5:1). R_f (30% EA/hexane) = 0.4.

LC-MS detection was attached as follow. However, S_2Cl_2 could not afford any products except a series of polymers. Prominently, **9h** was afforded as the main product when reagent **1d** applied in this reaction. S_2Cl_2 couldn't work out in tetrasulfide synthesis even with substrate possessing hydroxyl group.

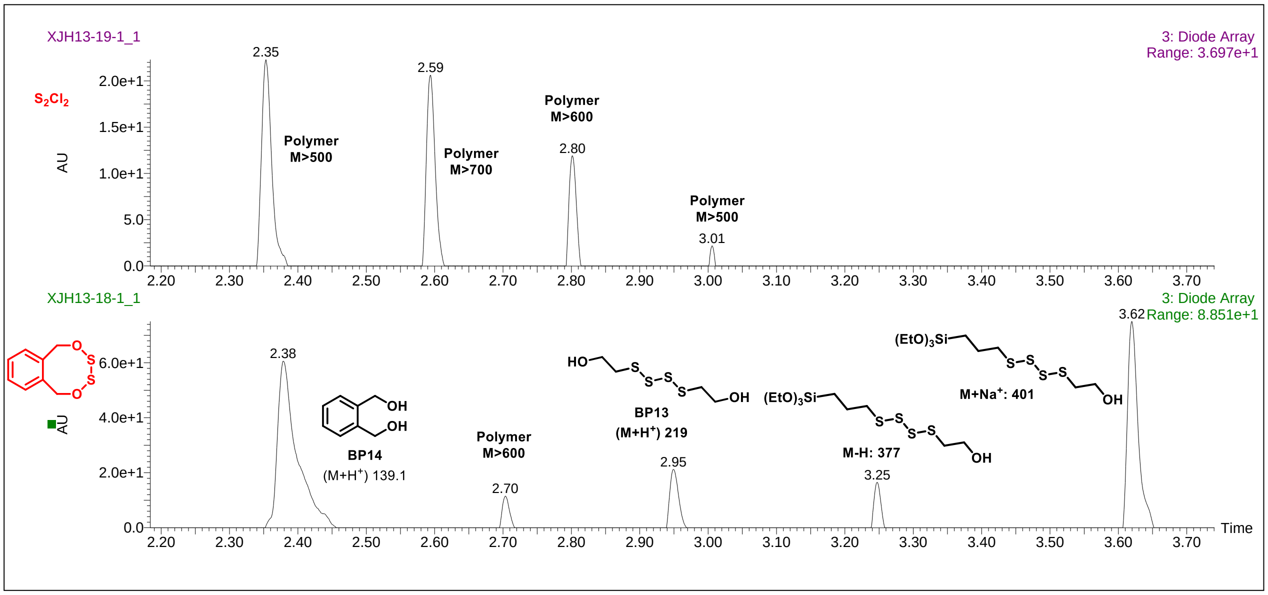

Supplementary Figure 6. Comparison of LC-MS between **1d** and S_2Cl_2.

Comparison of energy difference of two disulfuration.

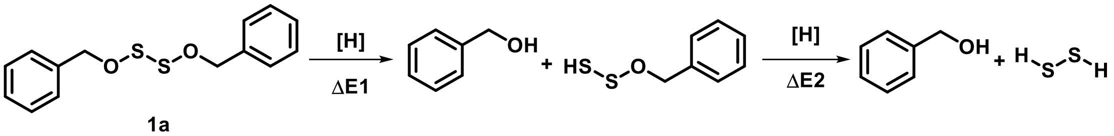

1a

ΔE1(**1a**) = E(product) - E(reactant)

= 3.790 kcal mol^{-1} + 0.780 kcal mol^{-1} - 7.253 kcal mol^{-1}

= -2.68 kcal mol^{-1}

ΔE2(**1a**) = E(product) - E(reactant)

= 0.780 kcal mol^{-1} + 0.210 kcal mol^{-1} - 3.79 kcal mol^{-1}

= -2.80 kcal mol^{-1}

ΔE2(**1a**) - ΔE1(**1a**) = -2.8 kcal mol^{-1} - (-2.68 kcal mol^{-1})

= -0.12 kcal mol^{-1}

Supplementary Figure 7. Energy imparity of **1a**.

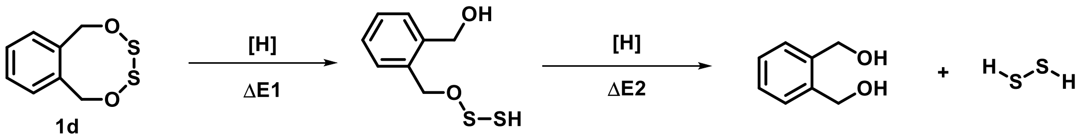

1d

ΔE1(**1d**) = E(product) - E(reactant)

= 3.518 kcal mol^{-1} - 11.347 kcal mol^{-1}

= -7.83 kcal mol^{-1}

ΔE2(**1d**) = E(product) - E(reactant)

= 5.004 kcal mol^{-1} + 0.210 kcal mol^{-1} - 3.518 kcal mol^{-1}

= 1.7 kcal mol^{-1}

ΔE2(**1d**) - ΔE1(**1d**) = = 1.7 kcal mol^{-1} - (-7.83 kcal mol^{-1})

= 9.53 kcal mol^{-1}

Supplementary Figure 8. Energy imparity of **1d**.

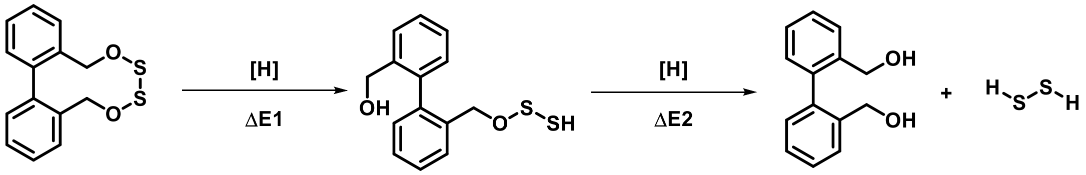

1f

ΔE1(**1f**) = E(product) - E(reactant)

= 23.737 kcal mol^{-1} - 31.484 kcal mol^{-1}

= -7.75 kcal mol^{-1}

ΔE2(**1f**) = E(product) - E(reactant)

= 20.996 kcal mol^{-1} + 0.210 kcal mol^{-1} - 23.737 kcal mol^{-1}

= -2.53 kcal mol^{-1}

ΔE2(**1f**) - ΔE1(**1f**) = -2.53 kcal mol^{-1} - (-7.75 kcal mol^{-1})

= 5.22 kcal mol^{-1}

Supplementary Figure 9. Energy imparity of **1f**.

X-ray Crystallography Analysis

Compound **1f** (CCDC-1941481)

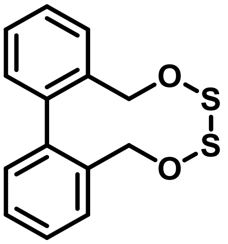

1f

≡

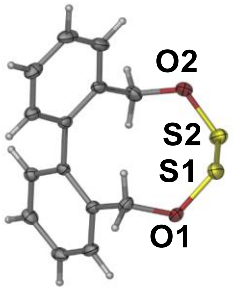

X-ray of 1f

Datablock: exp_699

Bond precision: C-C = 0.0026 A Wavelength=1.54184

Cell: a=13.1126(1) b=5.7660(1) c=17.3870(2)
alpha=90 beta=95.193(1) gamma=90

Temperature: 293 K

	Calculated	Reported
Volume	1309.19(3)	1309.19(3)
Space group	P 21/c	P 1 21/c 1
Hall group	-P 2ybc	-P 2ybc
Moiety formula	C14 H12 O2 S2	C14 H12 O2 S2
Sum formula	C14 H12 O2 S2	C14 H12 O2 S2
Mr	276.36	276.36
Dx,g cm-3	1.402	1.402
Z	4	4
Mu (mm-1)	3.610	3.610
F000	576.0	576.0
F000'	580.04	
h,k,lmax	16,7,21	16,7,21
Nref	2667	2654
Tmin,Tmax	0.221,0.273	0.333,1.000
Tmin'	0.141	

Correction method= # Reported T Limits: Tmin=0.333 Tmax=1.000 AbsCorr = MULTI-SCAN

Data completeness= 0.995 Theta(max)= 74.388

R(reflections)= 0.0419(2588) wR2(reflections)= 0.1103(2654)

S = 1.062 Npar= 163

The following ALERTS were generated. Each ALERT has the format
test-name_ALERT_alert-type_alert-level.
Click on the hyperlinks for more details of the test.

Alert level C

PLAT911_ALERT_3_C Missing FCF Refl Between Thmin & STh/L= 0.600 6 Report

Alert level G

PLAT199_ALERT_1_G Reported _cell_measurement_temperature ..... (K) 293 Check
PLAT200_ALERT_1_G Reported _diffrn_ambient_temperature ..... (K) 293 Check
PLAT395_ALERT_2_G Deviating X-O-Y Angle From 120 for O1 117.9 Degree
PLAT395_ALERT_2_G Deviating X-O-Y Angle From 120 for O2 117.7 Degree
PLAT910_ALERT_3_G Missing # of FCF Reflection(s) Below Theta(Min). 1 Note
PLAT912_ALERT_4_G Missing # of FCF Reflections Above STh/L= 0.600 7 Note
PLAT933_ALERT_2_G Number of OMIT Records in Embedded .res File ... 6 Note
PLAT978_ALERT_2_G Number C-C Bonds with Positive Residual Density. 9 Info

Supplementary Figure 10. Single-Crystal X-ray Crystallography of **1f**.

Compound **3a** (CCDC-1941479)

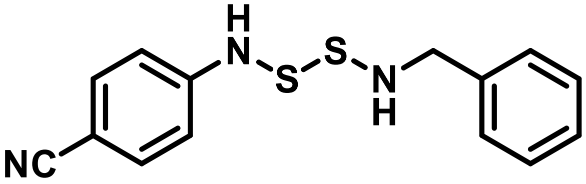

3a

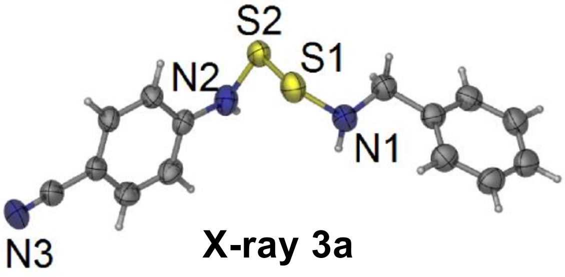

X-ray 3a

Datablock: exp_562

Bond precision: C-C = 0.0041 A Wavelength=1.54184

Cell: a=7.8734(1) b=11.4848(1) c=16.0642(2)
alpha=90 beta=90 gamma=90

Temperature: 293 K

	Calculated	Reported
Volume	1452.60(3)	1452.60(3)
Space group	P 21 21 21	P 21 21 21
Hall group	P 2ac 2ab	P 2ac 2ab
Moiety formula	C14 H13 N3 S2	C14 H13 N3 S2
Sum formula	C14 H13 N3 S2	C14 H13 N3 S2
Mr	287.39	287.39
Dx, g cm-3	1.314	1.314
Z	4	4
Mu (mm-1)	3.229	3.229
F000	600.0	600.0
F000'	603.96	
h,k,lmax	9,14,20	9,14,20
Nref	2977[1724]	2949
Tmin,Tmax	0.454,0.679	0.160,1.000
Tmin'	0.272	

Correction method= # Reported T Limits: Tmin=0.160 Tmax=1.000 AbsCorr = MULTI-SCAN

Data completeness= 1.71/0.99 Theta(max)= 74.535

R(reflections)= 0.0332(2833) wR2(reflections)= 0.0914(2949)

S = 1.016 Npar= 177

The following ALERTS were generated. Each ALERT has the format
test-name_ALERT_alert-type_alert-level.
Click on the hyperlinks for more details of the test.

Alert level C

PLAT340_ALERT_3_C Low Bond Precision on C-C Bonds .... 0.00407 Ang.
PLAT420_ALERT_2_C D-H Without Acceptor N1 --H1 . Please Check
PLAT911_ALERT_3_C Missing FCF Refl Between Thmin & STh/L= 0.600 15 Report

Alert level G

PLAT007_ALERT_5_G Number of Unrefined Donor-H Atoms .... 1 Report
PLAT142_ALERT_4_G s.u. on b - Axis Small or Missing .... 0.00010 Ang.
PLAT199_ALERT_1_G Reported _cell_measurement_temperature ..... (K) 293 Check
PLAT200_ALERT_1_G Reported _diffrn_ambient_temperature ..... (K) 293 Check
PLAT912_ALERT_4_G Missing # of FCF Reflections Above STh/L= 0.600 2 Note
PLAT933_ALERT_2_G Number of OMIT Records in Embedded .res File ... 19 Note
PLAT978_ALERT_2_G Number C-C Bonds with Positive Residual Density. 2 Info

Supplementary Figure 11. Single-Crystal X-ray Crystallography of **3a**.

Compound **4d** (CCDC-1941480)

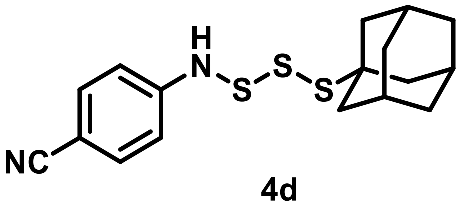

4d

≡

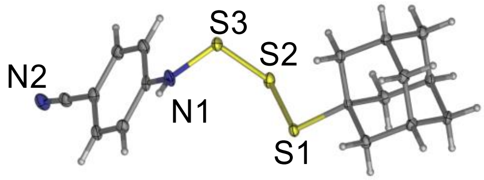

X ray of 4d

Datablock: exp_464

Bond precision: C-C = 0.0039 A Wavelength=1.54184

Cell: a=6.4849(2) b=6.8043(2) c=19.5048(4)
alpha=96.125(2) beta=92.066(2) gamma=100.815(2)

Temperature: 100 K

	Calculated	Reported
Volume	839.15(4)	839.15(4)
Space group	P -1	P -1
Hall group	-P 1	-P 1
Moiety formula	C17 H20 N2 S3	C17 H20 N2 S3
Sum formula	C17 H20 N2 S3	C17 H20 N2 S3
Mr	348.53	348.53
Dx, g cm-3	1.379	1.379
Z	2	2
Mu (mm-1)	4.003	4.003
F000	368.0	368.0
F000'	370.71	
h,k,lmax	7,8,23	7,8,23
Nref	2990	2906
Tmin,Tmax	0.183,0.353	0.753,1.000
Tmin'	0.117	

Correction method= # Reported T Limits: Tmin=0.753 Tmax=1.000 AbsCorr = MULTI-SCAN

Data completeness= 0.972 Theta(max)= 67.029

R(reflections)= 0.0725(2664) wR2(reflections)= 0.1904(2906)

S = 1.036 Npar= 200

The following ALERTS were generated. Each ALERT has the format
test-name_ALERT_alert-type_alert-level.
Click on the hyperlinks for more details of the test.

Alert level B

RINTA01_ALERT_3_B The value of Rint is greater than 0.18
Rint given 0.194
PLAT020_ALERT_3_B The Value of Rint is Greater Than 0.12 .... 0.194 Report

Alert level C

PLAT029_ALERT_3_C _diffrn_measured_fraction_theta_full value Low . 0.972 Why?
PLAT031_ALERT_4_C Refined Extinction Parameter Within Range ...... 3.000 Sigma
PLAT911_ALERT_3_C Missing FCF Refl Between Thmin & STh/L= 0.597 82 Report
PLAT975_ALERT_2_C Check Calcd Resid. Dens. 0.95A From N1 0.58 eA-3
PLAT976_ALERT_2_C Check Calcd Resid. Dens. 1.08A From N1 -0.49 eA-3

Alert level G

PLAT007_ALERT_5_G Number of Unrefined Donor-H Atoms .... 1 Report
PLAT072_ALERT_2_G SHELXL First Parameter in WGHT Unusually Large 0.15 Report
PLAT154_ALERT_1_G The s.u.'s on the Cell Angles are Equal ..(Note) 0.002 Degree
PLAT909_ALERT_3_G Percentage of I>2sig(I) Data at Theta(Max) Still 86% Note
PLAT910_ALERT_3_G Missing # of FCF Reflection(s) Below Theta(Min). 2 Note
PLAT933_ALERT_2_G Number of OMIT Records in Embedded .res File ... 73 Note
PLAT978_ALERT_2_G Number C-C Bonds with Positive Residual Density. 1 Info

Supplementary Figure 12. Single-Crystal X-ray Crystallography of **4d**.

Compound of **8f** (CCDC-1941478)

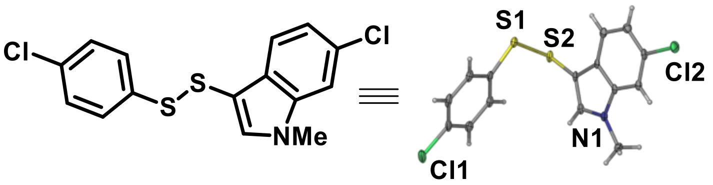

8f **X ray of 8f**

Datablock: exp_919

Bond precision: C-C = 0.0033 A Wavelength=1.54184

Cell: a=7.4494(2) b=9.8774(3) c=10.0697(3)
alpha=84.979(2) beta=86.997(2) gamma=84.968(3)

Temperature: 293 K

	Calculated	Reported
Volume	734.50(4)	734.50(4)
Space group	P -1	P -1
Hall group	-P 1	-P 1
Moiety formula	C15 H11 Cl2 N S2	C15 H11 Cl2 N S2
Sum formula	C15 H11 Cl2 N S2	C15 H11 Cl2 N S2
Mr	340.27	340.27
Dx, g cm-3	1.539	1.539
Z	2	2
Mu (mm-1)	6.521	6.521
F000	348.0	348.0
F000'	351.40	
h, k, lmax	9, 12, 12	9, 12, 12
Nref	3006	2887
Tmin, Tmax	0.223, 0.457	0.234, 1.000
Tmin'	0.076	

Correction method= # Reported T Limits: Tmin=0.234 Tmax=1.000 AbsCorr = MULTI-SCAN

Data completeness= 0.960 Theta(max)= 74.579

R(reflections)= 0.0568(2636) wR2(reflections)= 0.1609(2887)

S = 1.081 Npar= 183

The following ALERTS were generated. Each ALERT has the format
test-name_ALERT_alert-type_alert-level.
Click on the hyperlinks for more details of the test.

Alert level C

PLAT250_ALERT_2_C Large U3/U1 Ratio for Average U(i,j) Tensor .... 2.4 Note
PLAT911_ALERT_3_C Missing FCF Refl Between Thmin & STh/L= 0.600 34 Report

Alert level G

PLAT072_ALERT_2_G SHELXL First Parameter in WGHT Unusually Large 0.12 Report
PLAT199_ALERT_1_G Reported _cell_measurement_temperature ..... (K) 293 Check
PLAT200_ALERT_1_G Reported _diffrn_ambient_temperature ..... (K) 293 Check
PLAT912_ALERT_4_G Missing # of FCF Reflections Above STh/L= 0.600 85 Note
PLAT933_ALERT_2_G Number of OMIT Records in Embedded .res File ... 20 Note
PLAT978_ALERT_2_G Number C-C Bonds with Positive Residual Density. 5 Info

0 **ALERT level A** = Most likely a serious problem - resolve or explain
0 **ALERT level B** = A potentially serious problem, consider carefully
2 **ALERT level C** = Check. Ensure it is not caused by an omission or oversight
6 **ALERT level G** = General information/check it is not something unexpected

2 ALERT type 1 CIF construction/syntax error, inconsistent or missing data
4 ALERT type 2 Indicator that the structure model may be wrong or deficient
1 ALERT type 3 Indicator that the structure quality may be low
1 ALERT type 4 Improvement, methodology, query or suggestion
0 ALERT type 5 Informative message, check

Supplementary Figure 13. Single-Crystal X-ray Crystallography of **8f**.

Compound **9a** (CCDC-1941482)

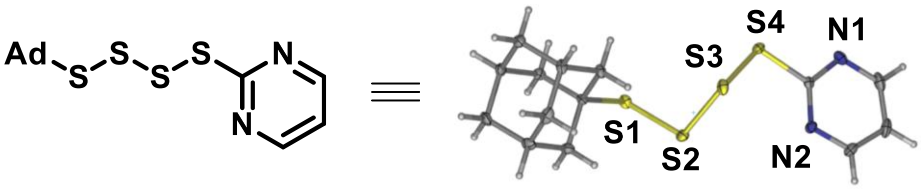

9a **X ray of 9a**

Datablock: exp_734

Bond precision: C-C = 0.0030 A Wavelength=1.54184

Cell:	a=9.4898(1)	b=15.8352(1)	c=11.3386(1)
	alpha=90	beta=113.812(1)	gamma=90

Temperature: 293 K

	Calculated	Reported
Volume	1558.84(3)	1558.84(3)
Space group	P 21/n	P 1 21/n 1
Hall group	-P 2yn	-P 2yn
Moiety formula	C14 H18 N2 S4	C14 H18 N2 S4
Sum formula	C14 H18 N2 S4	C14 H18 N2 S4
Mr	342.54	342.54
Dx, g cm-3	1.460	1.460
Z	4	4
Mu (mm-1)	5.516	5.516
F000	720.0	720.0
F000'	726.56	
h,k,lmax	11,19,14	11,19,14
Nref	3181	3145
Tmin,Tmax	0.138,0.213	0.107,1.000
Tmin'	0.047	

Correction method= # Reported T Limits: Tmin=0.107 Tmax=1.000 AbsCorr = MULTI-SCAN

Data completeness= 0.989 Theta(max)= 74.445

R(reflections)= 0.0384(3087) wR2(reflections)= 0.1069(3145)

S = 1.079 Npar= 182

The following ALERTS were generated. Each ALERT has the format
test-name_ALERT_alert-type_alert-level.
Click on the hyperlinks for more details of the test.

Alert level C

PLAT906_ALERT_3_C Large K Value in the Analysis of Variance ...... 2.063 Check
PLAT911_ALERT_3_C Missing FCF Refl Between Thmin & STh/L= 0.600 10 Report

Alert level G

PLAT142_ALERT_4_G s.u. on b - Axis Small or Missing .... 0.00010 Ang.
PLAT143_ALERT_4_G s.u. on c - Axis Small or Missing .... 0.00010 Ang.
PLAT153_ALERT_1_G The s.u.'s on the Cell Axes are Equal ..(Note) 0.0001 Ang.
PLAT199_ALERT_1_G Reported _cell_measurement_temperature ..... (K) 293 Check
PLAT200_ALERT_1_G Reported _diffrn_ambient_temperature ..... (K) 293 Check
PLAT912_ALERT_4_G Missing # of FCF Reflections Above STh/L= 0.600 27 Note
PLAT913_ALERT_3_G Missing # of Very Strong Reflections in FCF .... 1 Note
PLAT933_ALERT_2_G Number of OMIT Records in Embedded .res File ... 8 Note
PLAT961_ALERT_5_G Dataset Contains no Negative Intensities .... Please Check
PLAT978_ALERT_2_G Number C-C Bonds with Positive Residual Density. 7 Info

0 **ALERT level A** = Most likely a serious problem - resolve or explain
0 **ALERT level B** = A potentially serious problem, consider carefully
2 **ALERT level C** = Check. Ensure it is not caused by an omission or oversight
10 **ALERT level G** = General information/check it is not something unexpected

Supplementary Figure 14. Single-Crystal X-ray Crystallography of **9a**.

All these data can be obtained free of charge from Cambridge Crystallographic Data Centre via www.ccdc.cam.ac.uk/ data_request/ci.

NMR Spectra

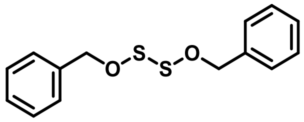

1a

^{1}H NMR ($CDCl_3$)

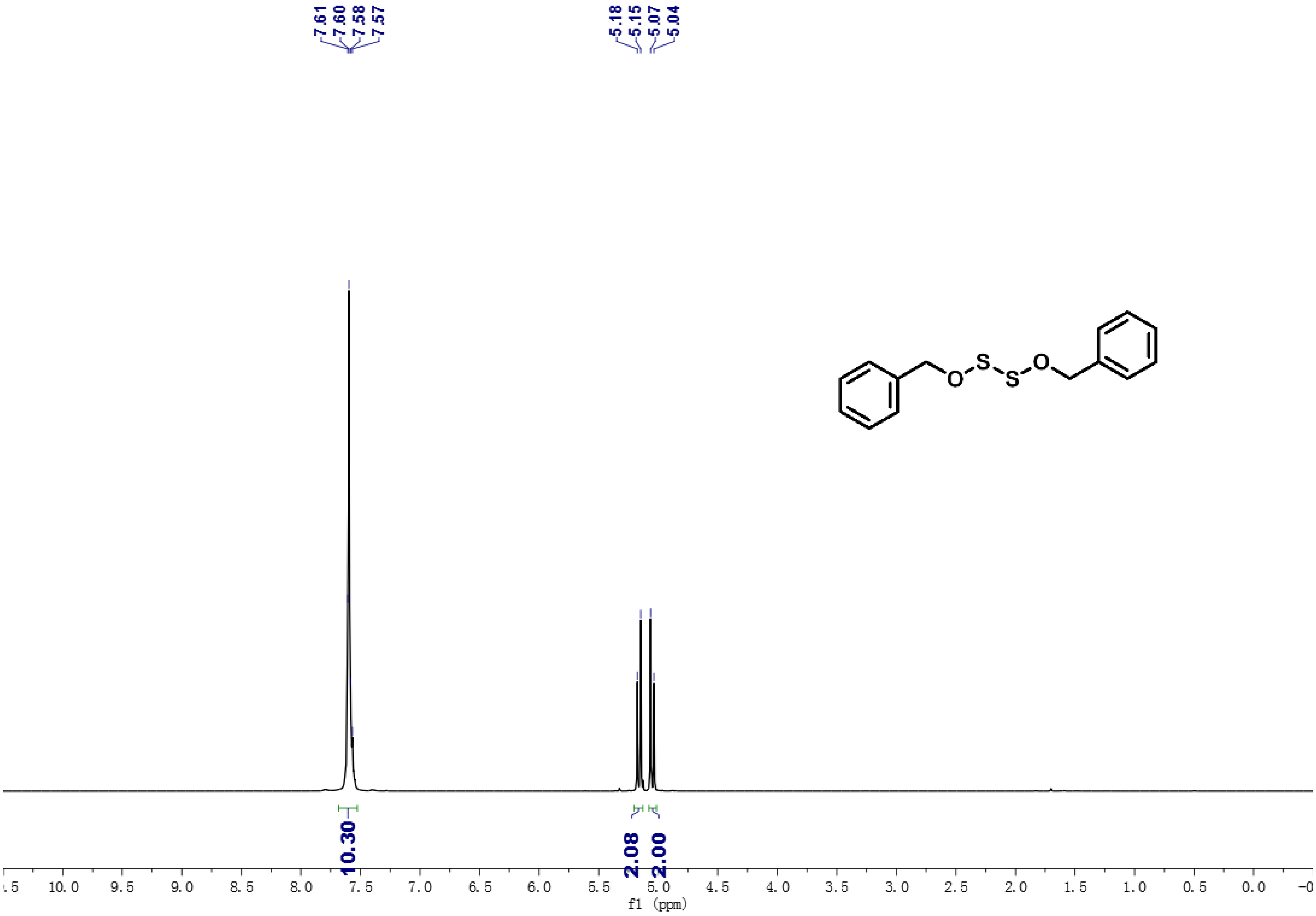

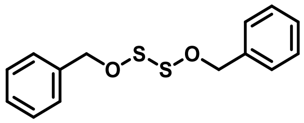

1a

^{13}C NMR ($CDCl_3$)

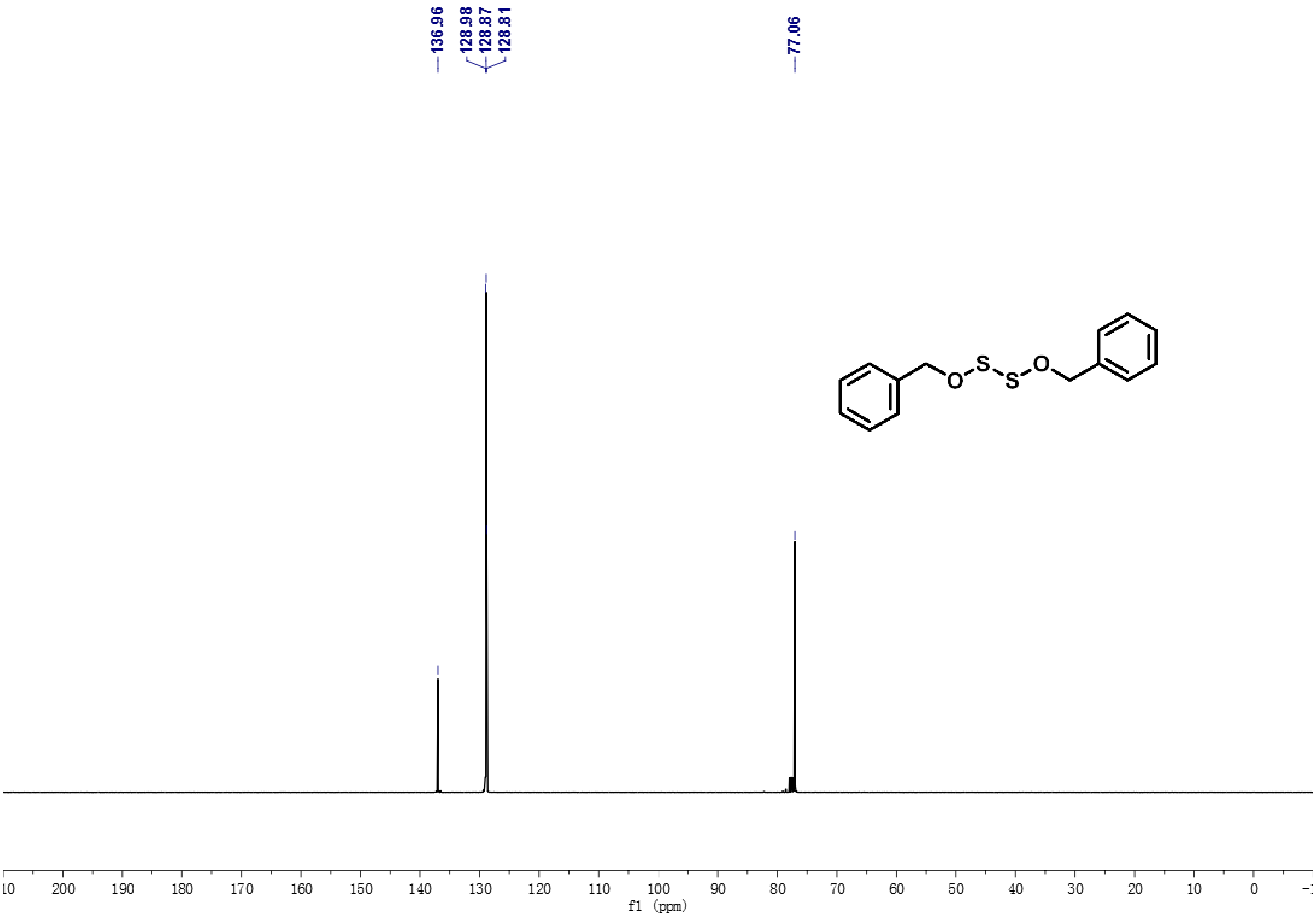

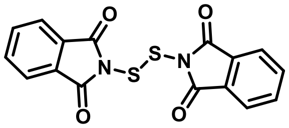

1b

^{1}H NMR (CDCl$_3$)

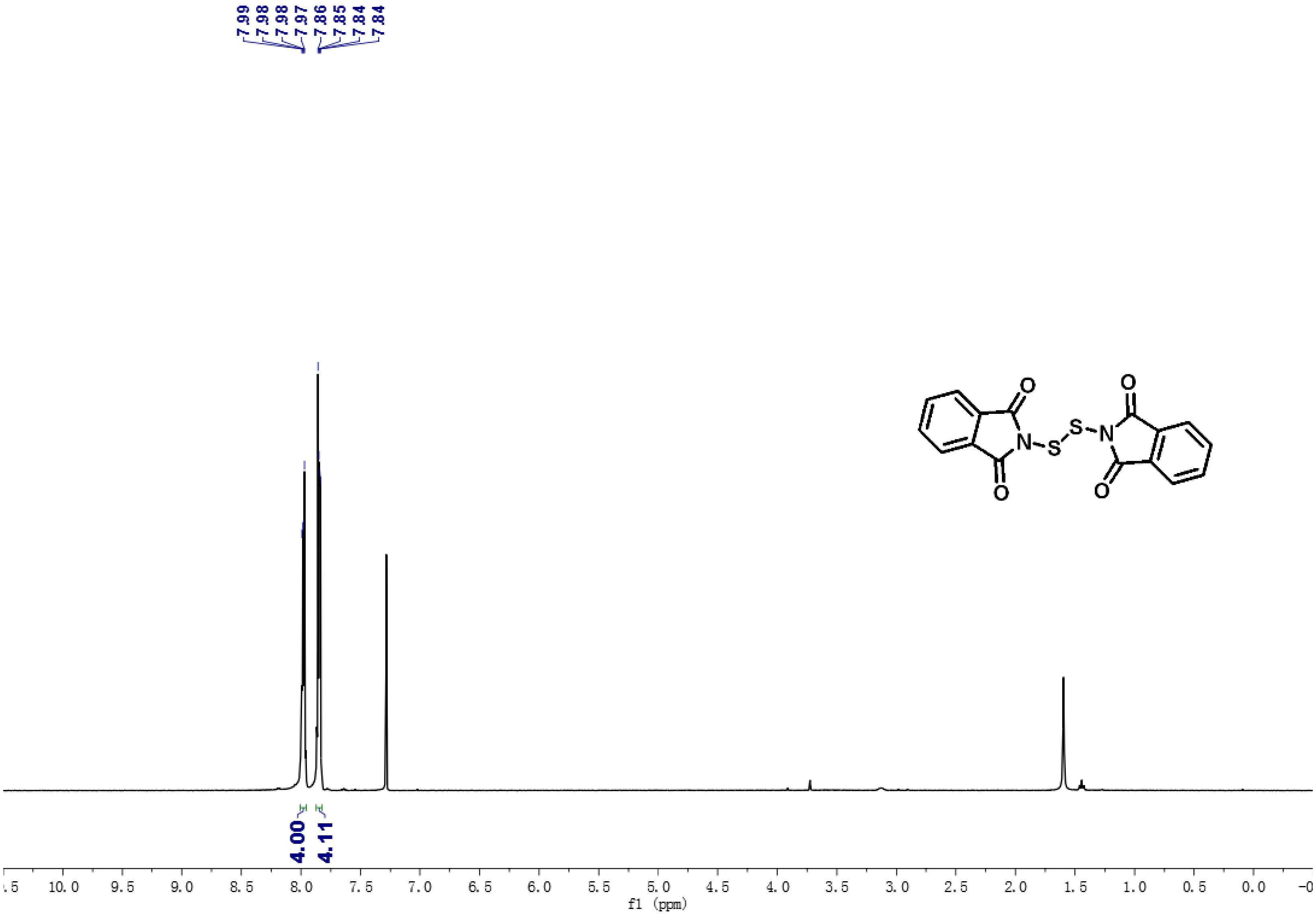

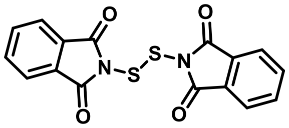

1b

^{13}C NMR ($CDCl_3$)

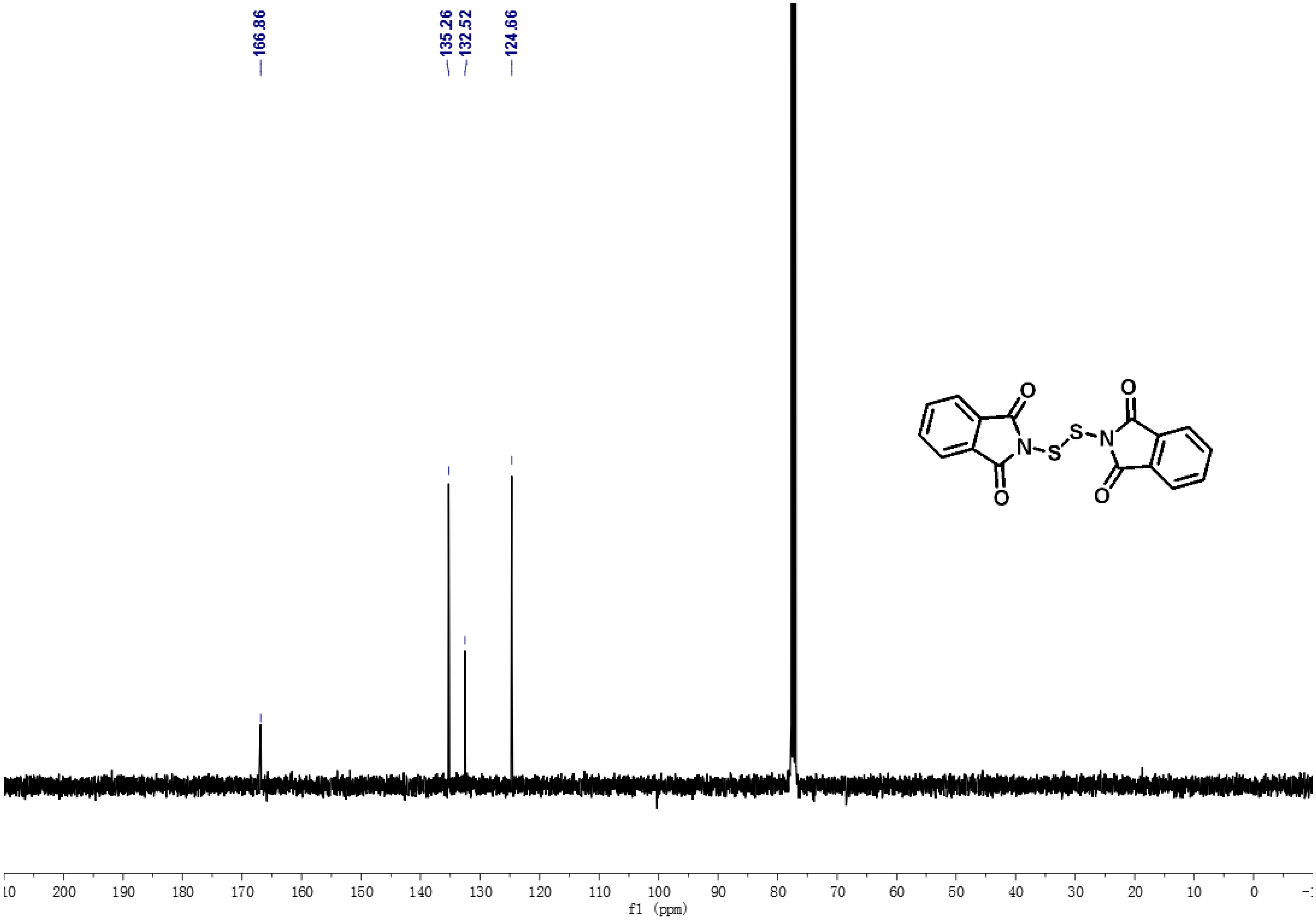

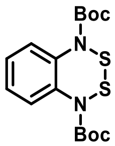

1c

^{1}H NMR ($CDCl_3$)

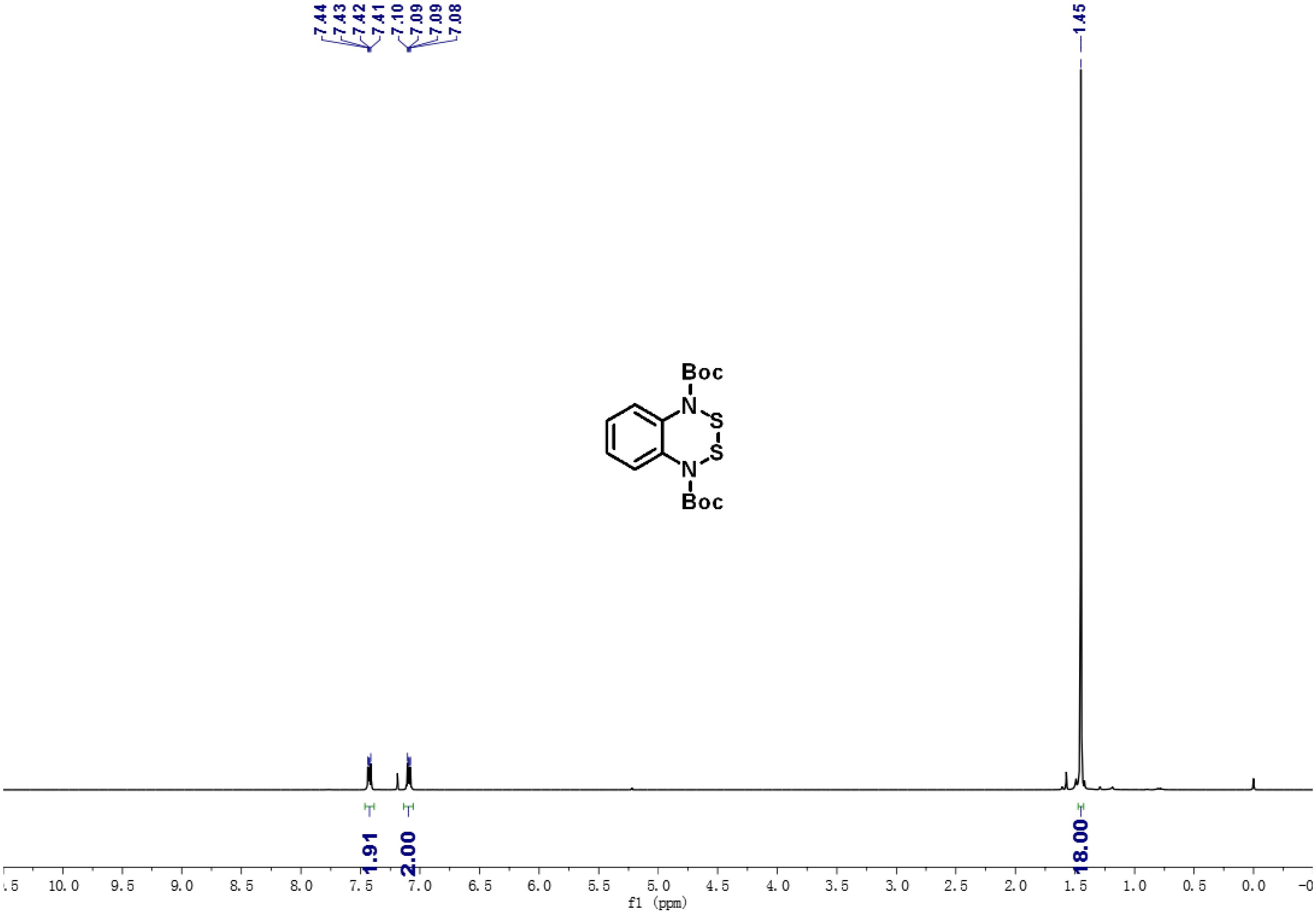

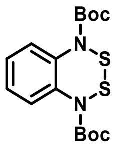

1c

^{13}C NMR ($CDCl_3$)

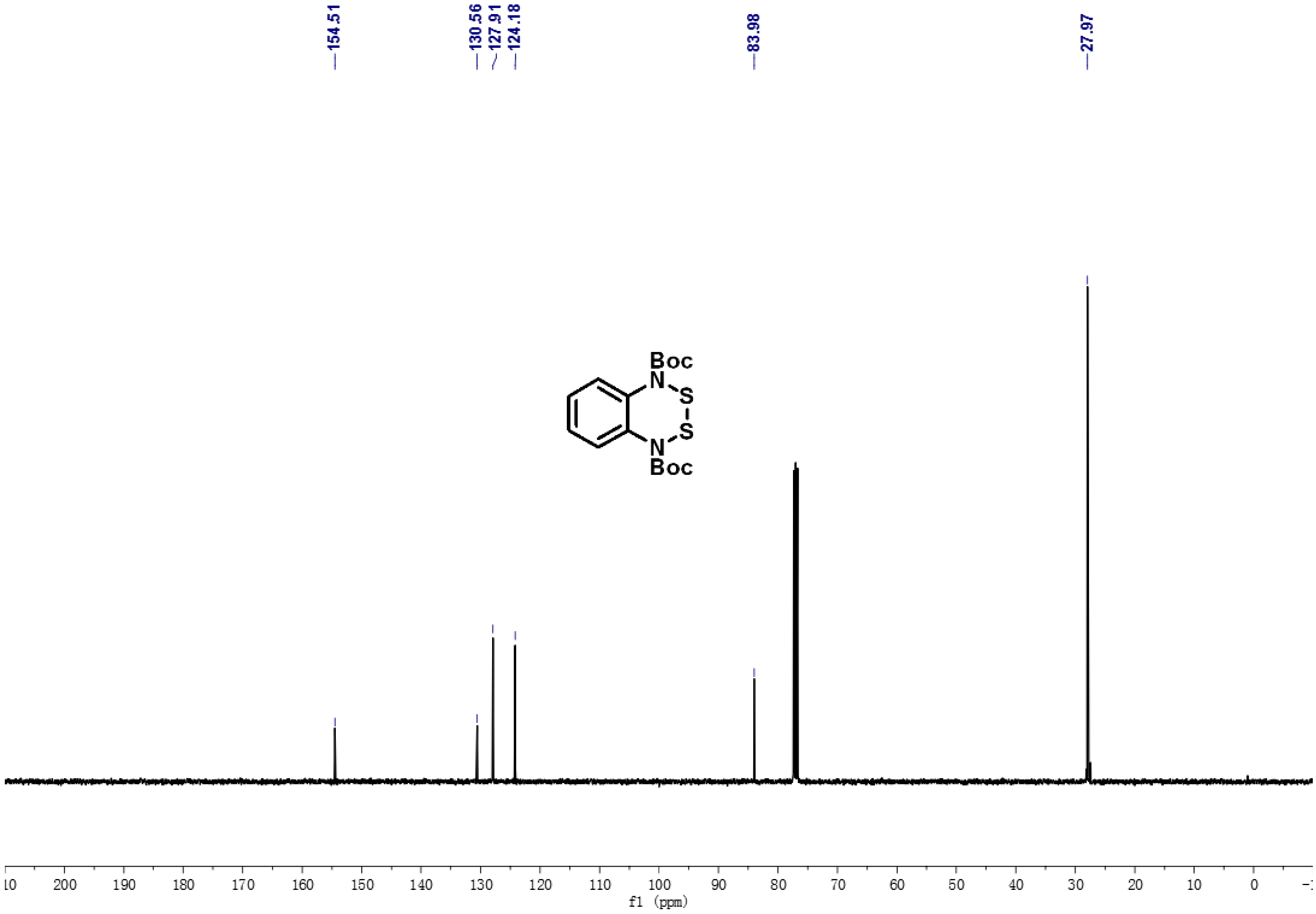

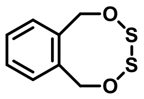

1d

^{1}H NMR ($CDCl_3$)

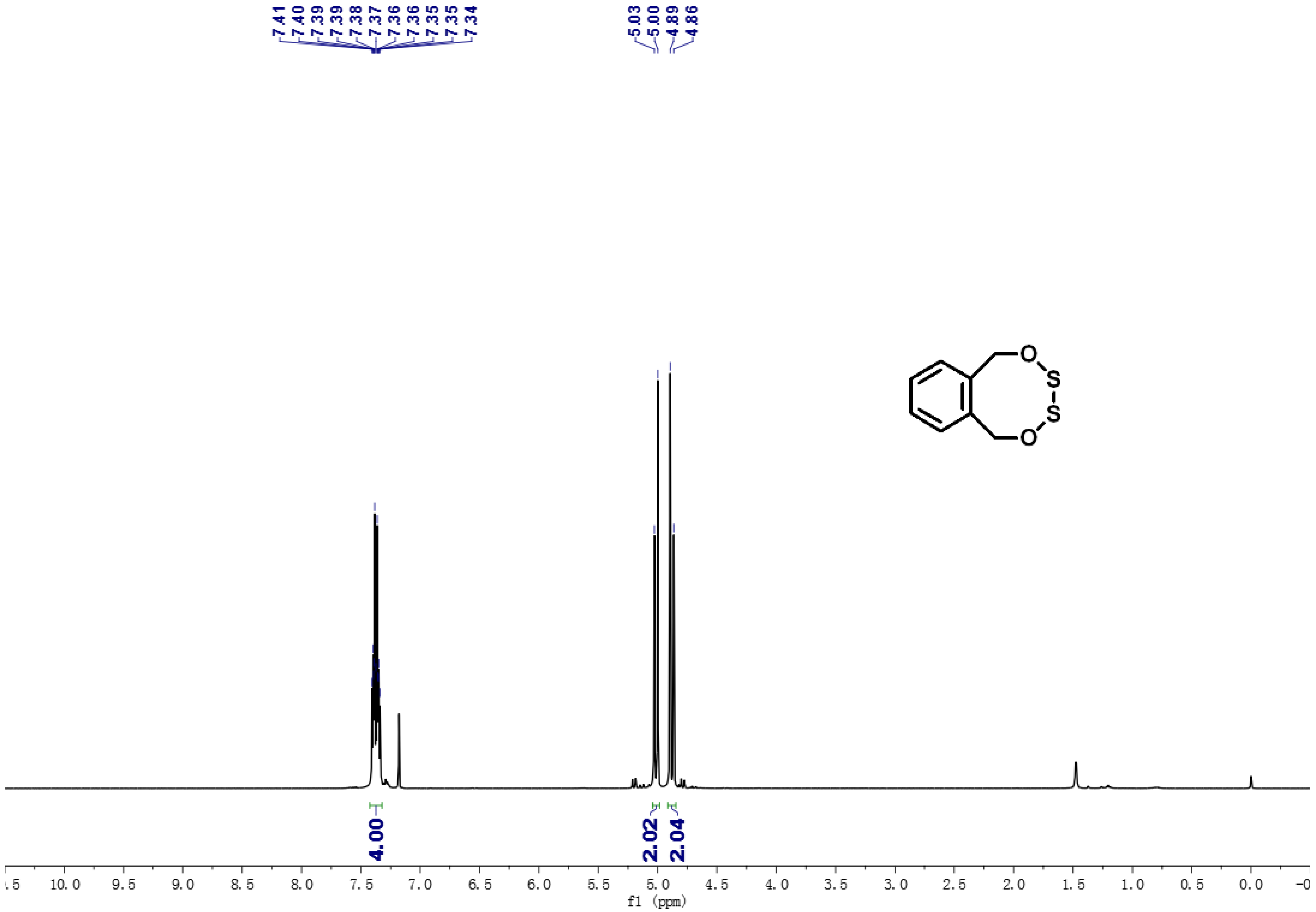

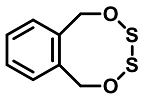

1d

^{13}C NMR ($CDCl_3$)

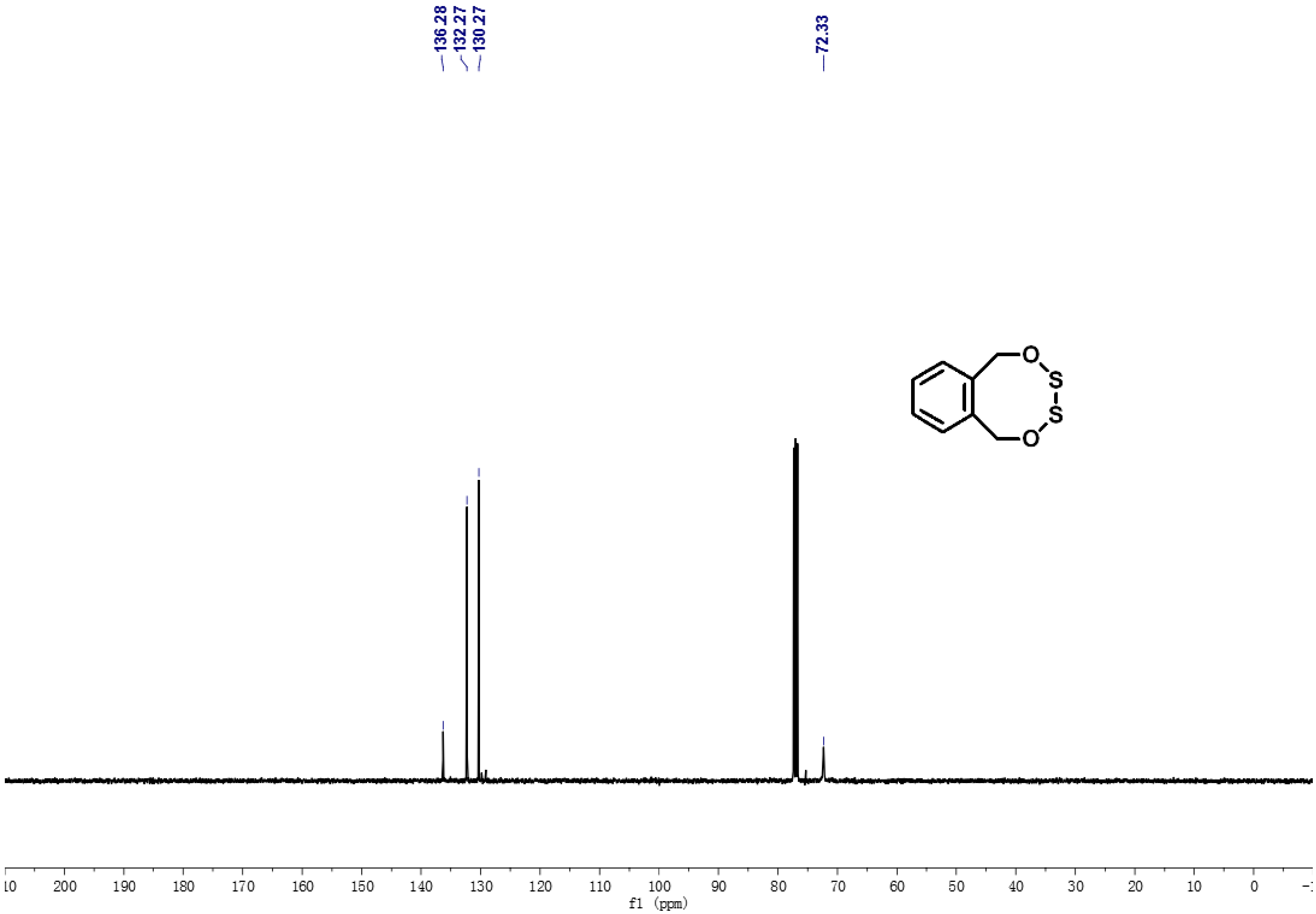

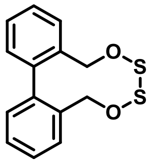

1f

^{1}H NMR ($CDCl_3$)

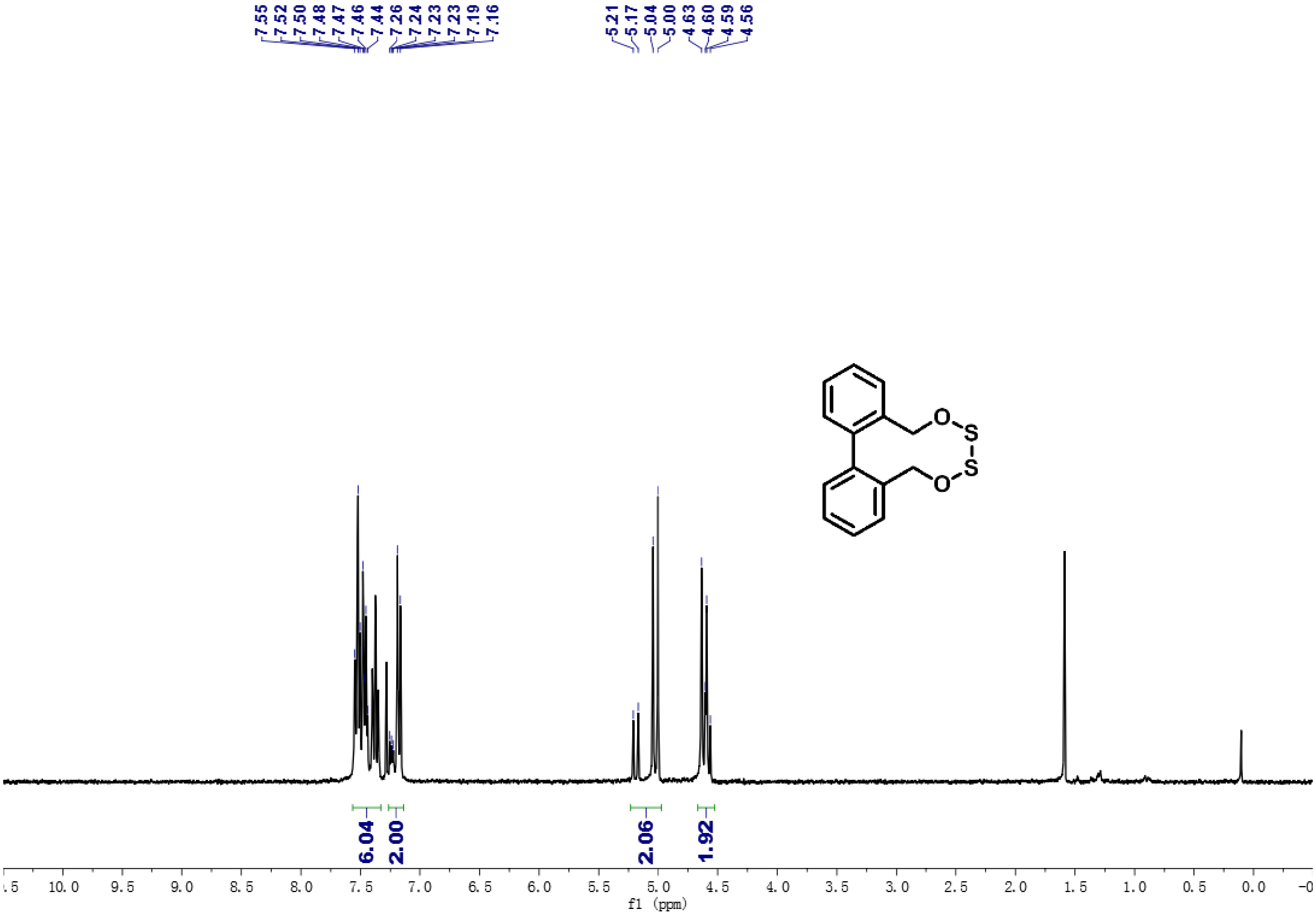

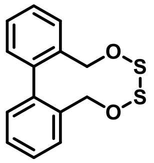

1f

^{13}C NMR ($CDCl_3$)

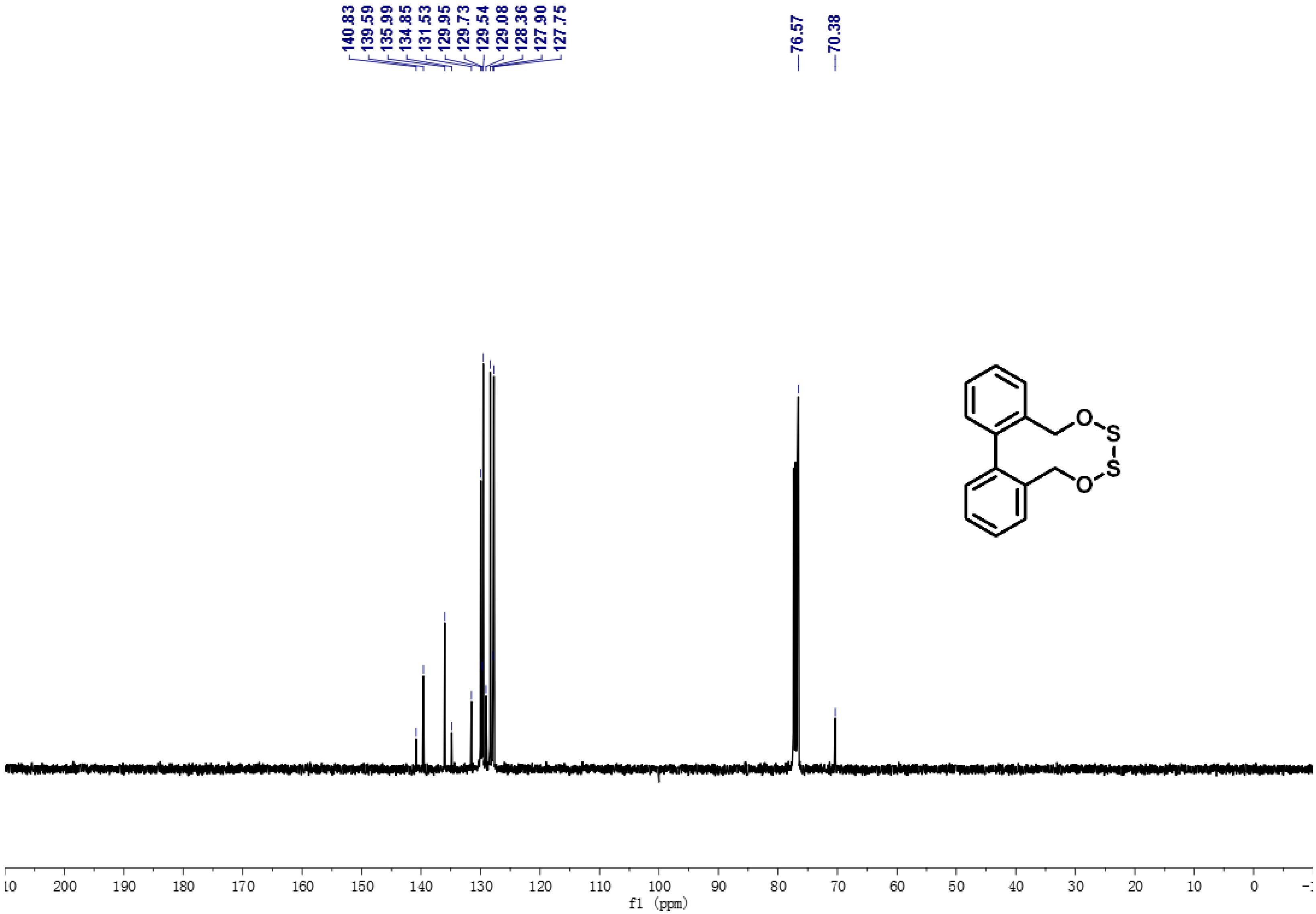

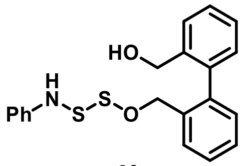

2f

^{1}H NMR (DMSO-d6)

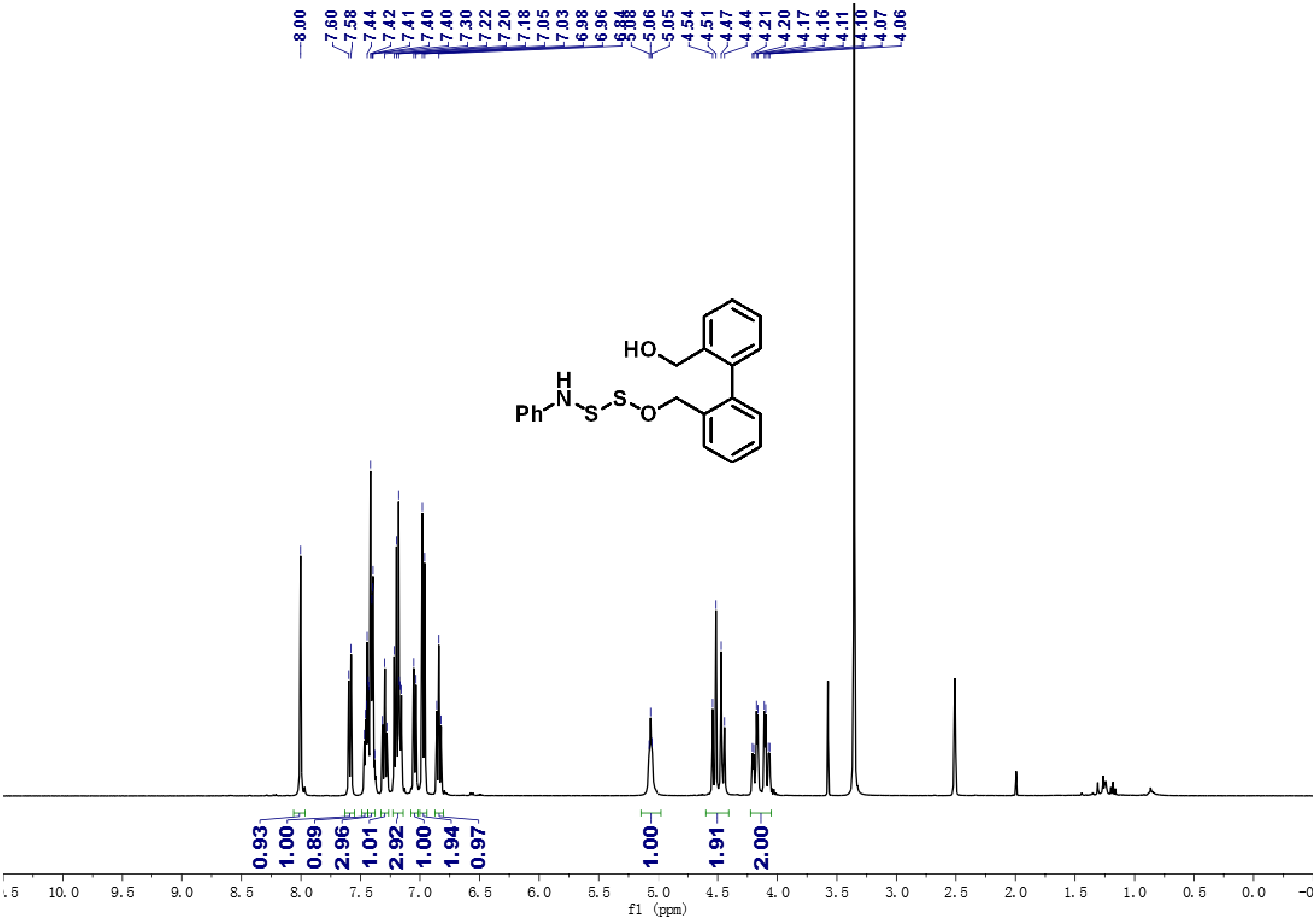

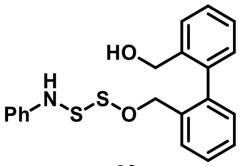

2f

^{13}C NMR (DMSO-d$_6$)

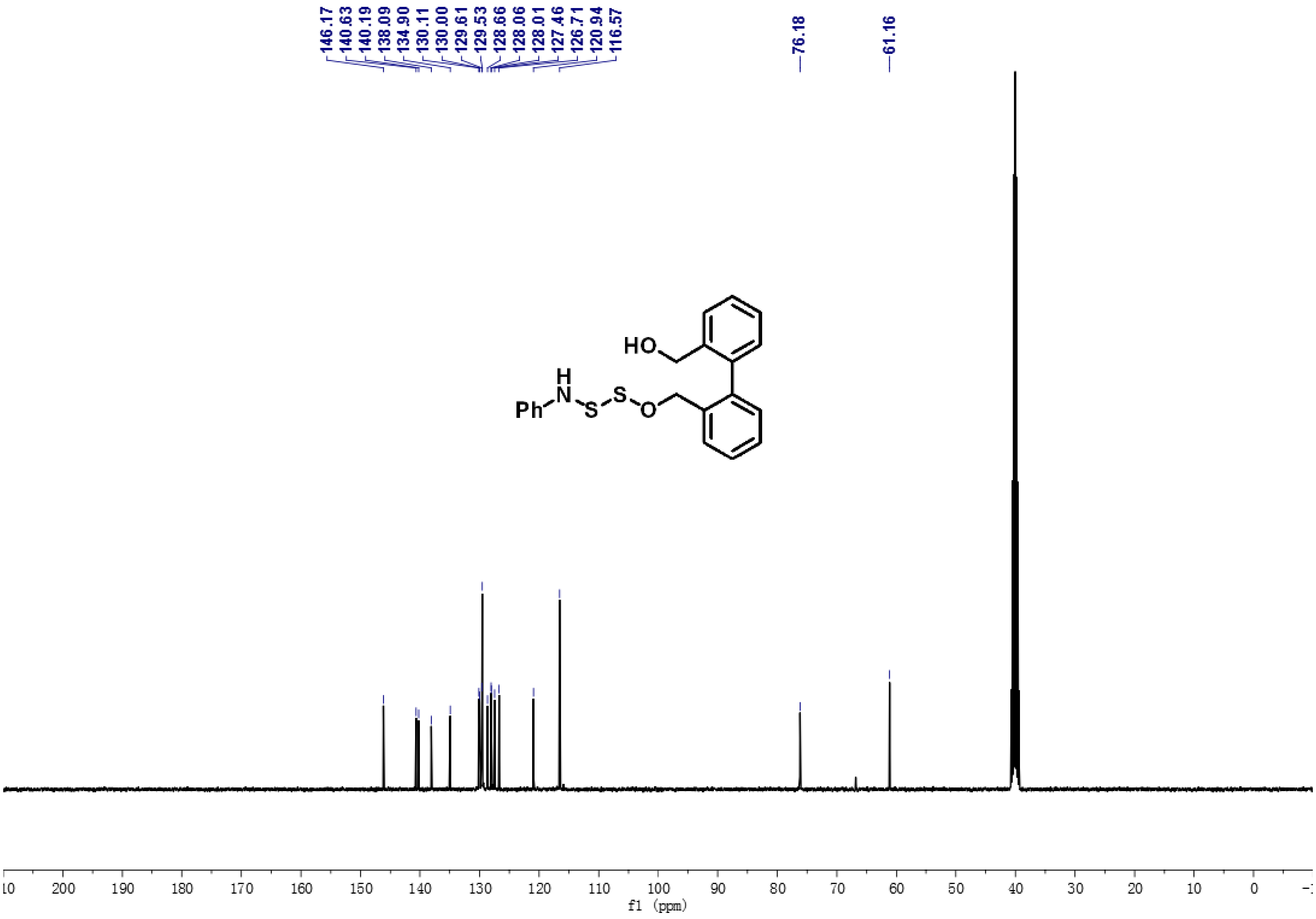

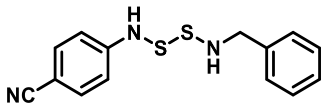

3a

^{1}H NMR (Acetone-d$_6$)

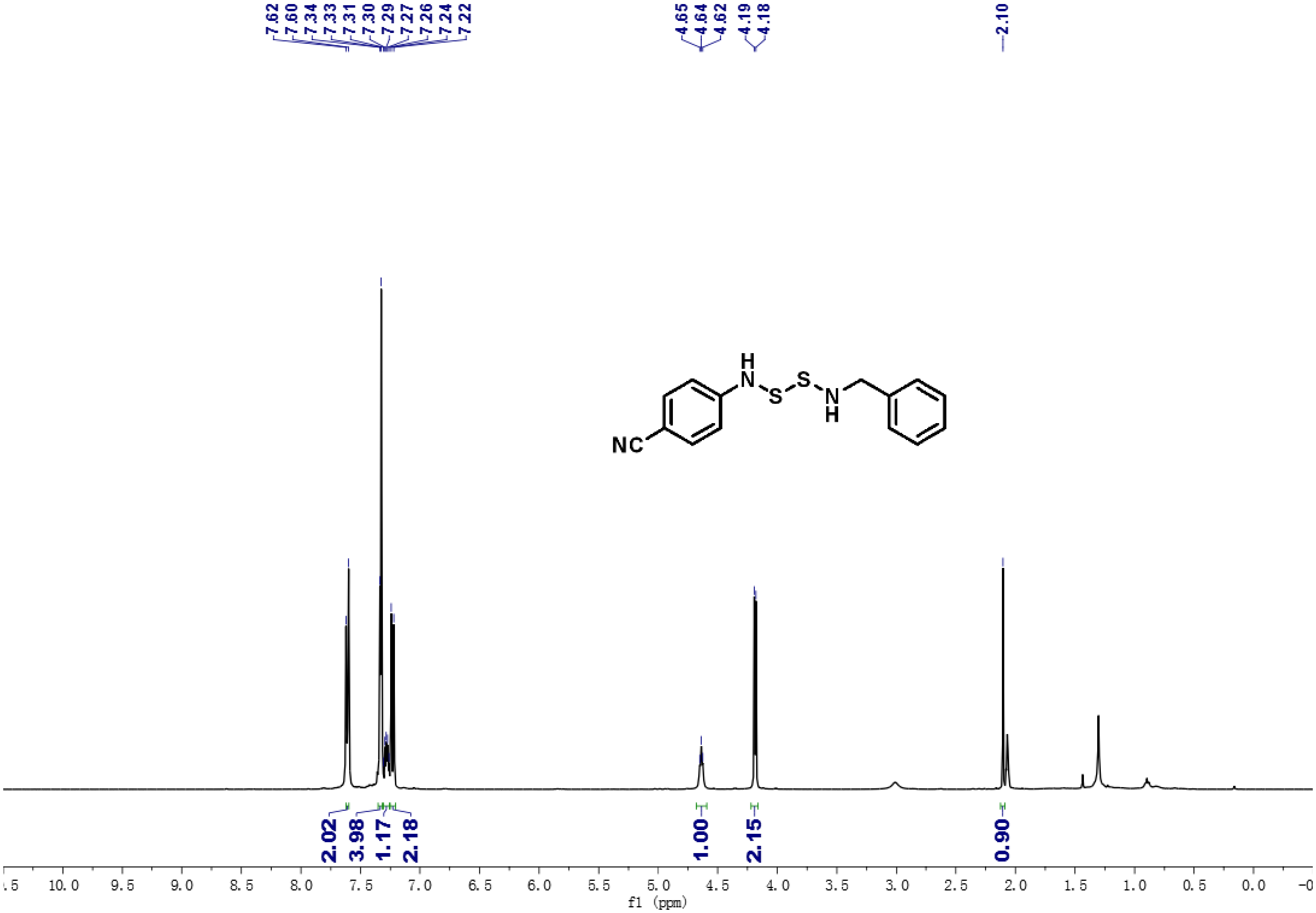

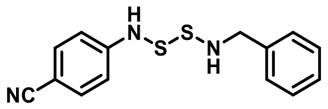

3a

^{13}C NMR (Acetone-d$_6$)

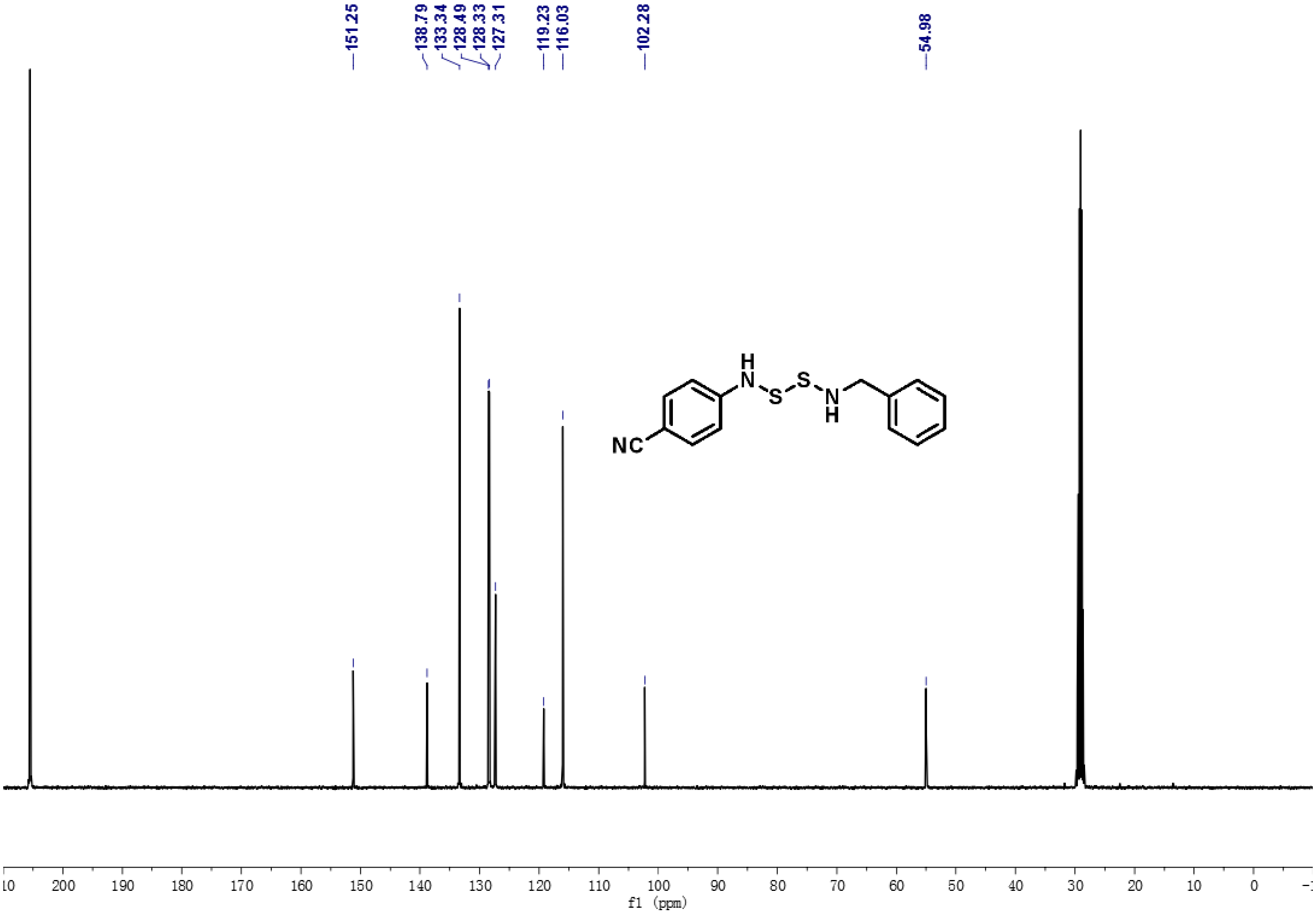

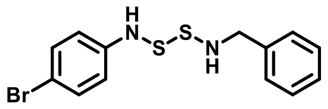

3b

^{1}H NMR ($CDCl_3$)

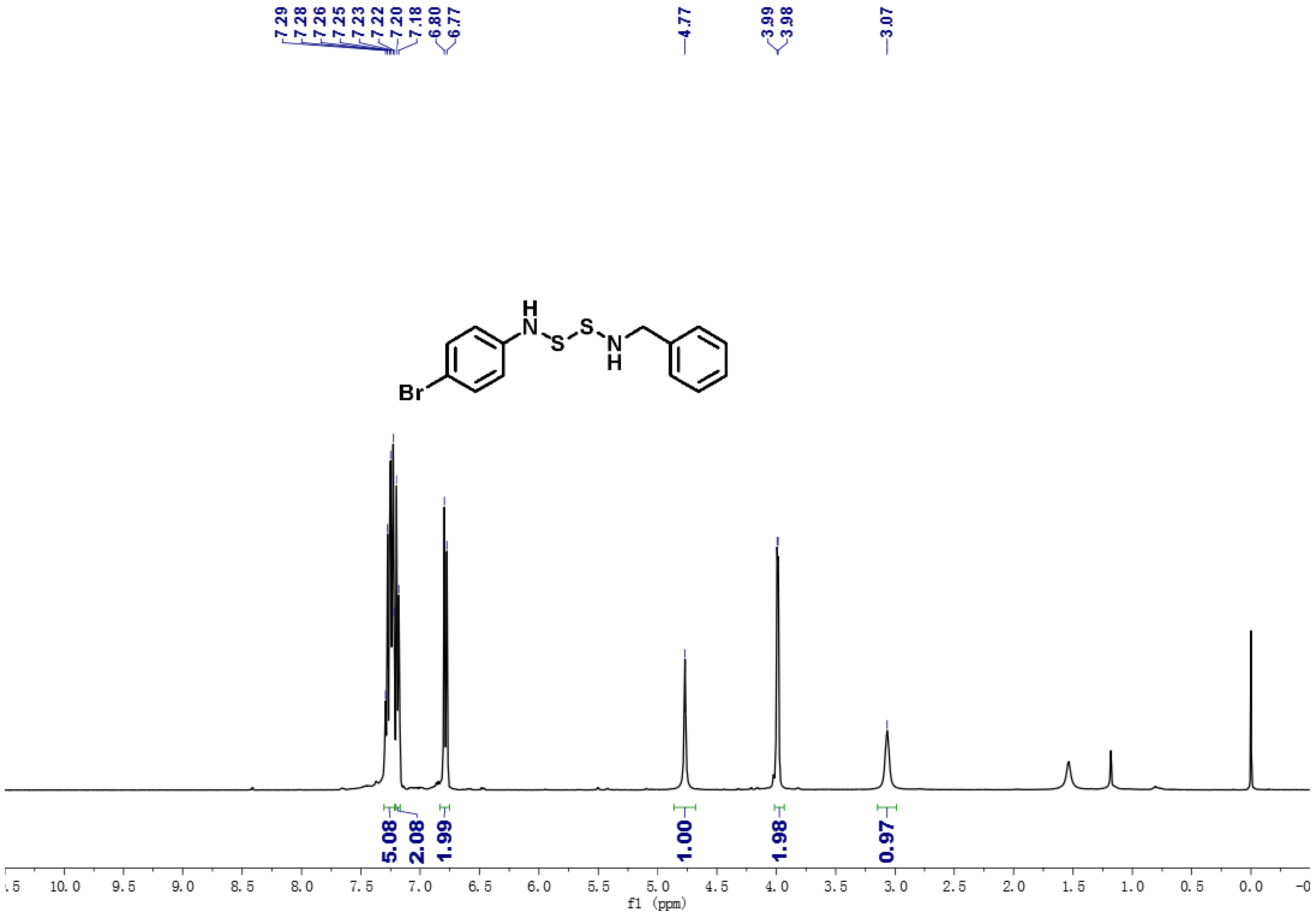

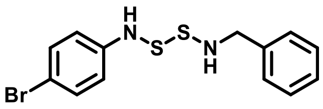

3b

^{13}C NMR ($CDCl_3$)

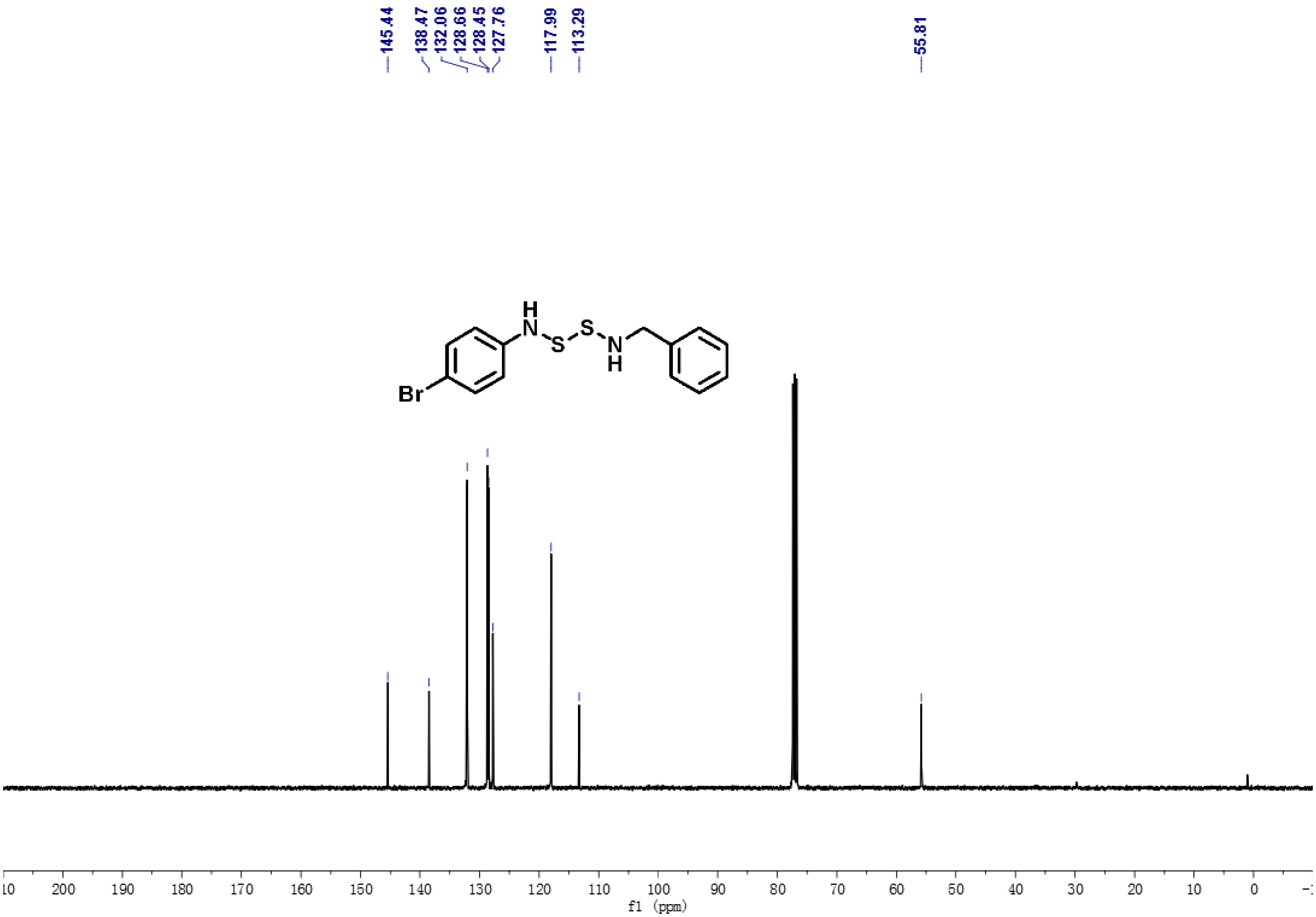

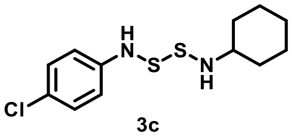

3c

^{1}H NMR ($CDCl_3$)

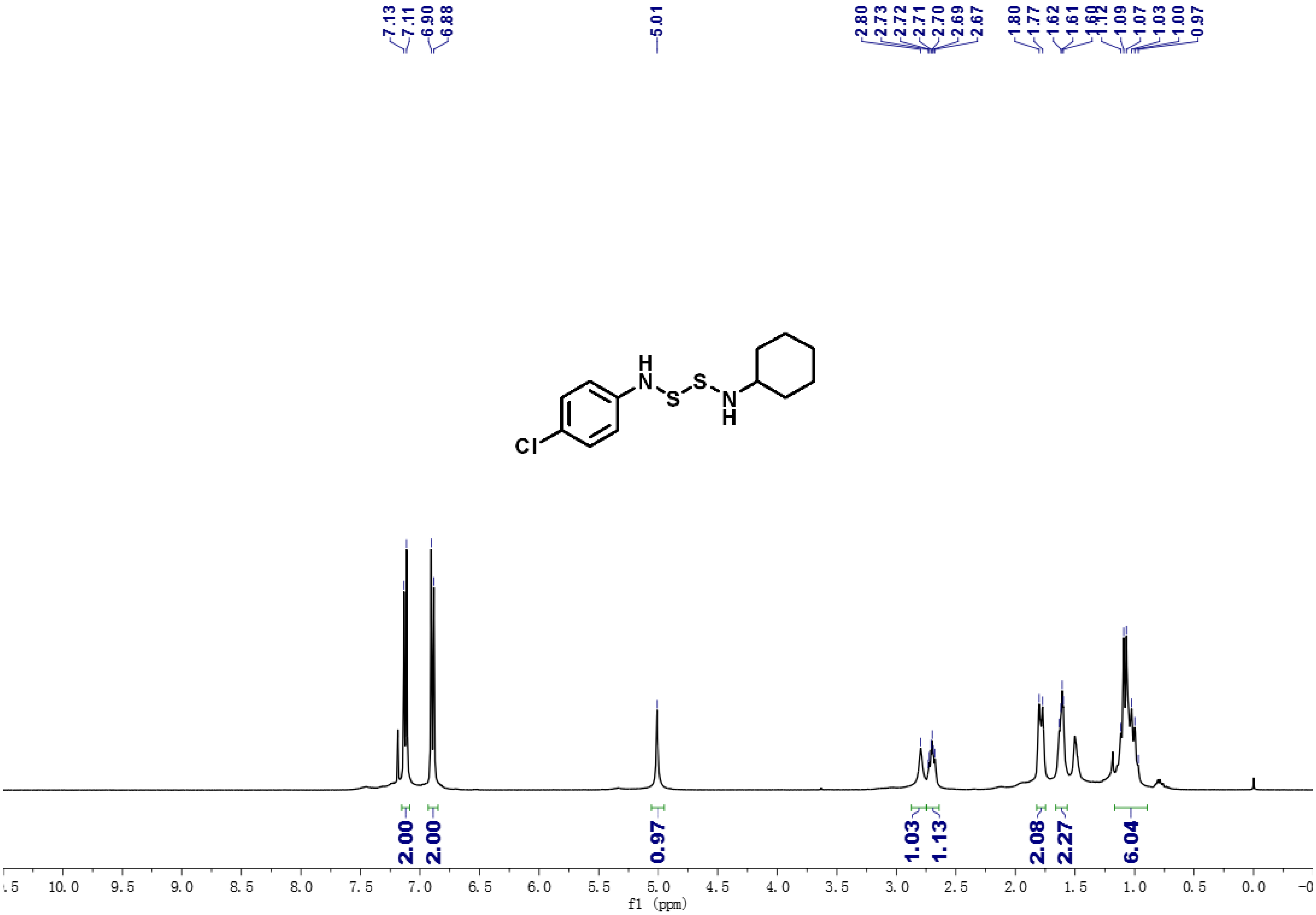

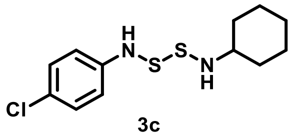

3c

^{13}C NMR ($CDCl_3$)

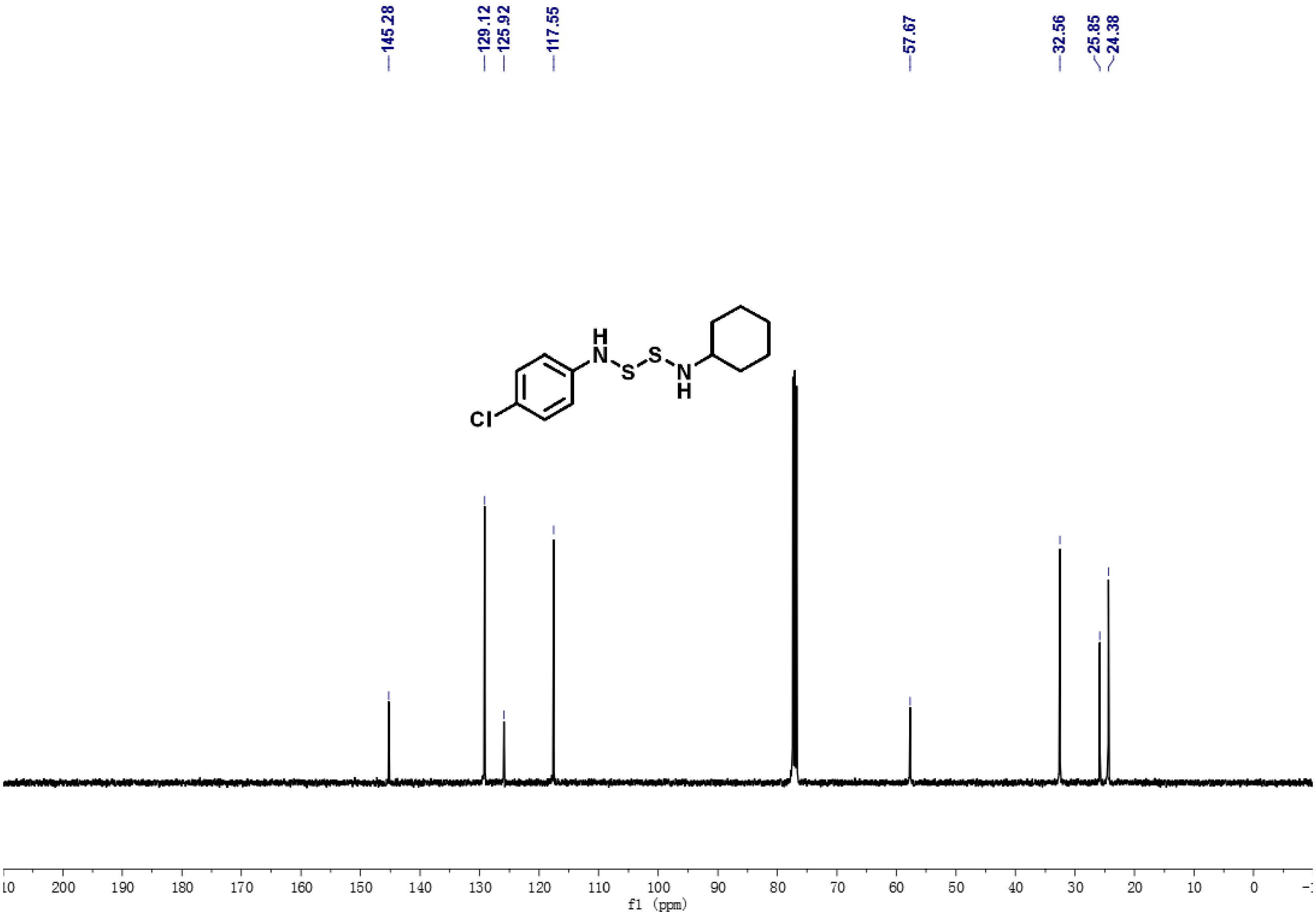

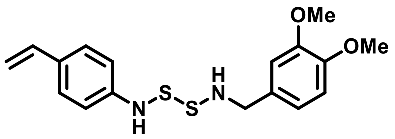

3d

^{1}H NMR ($CDCl_3$)

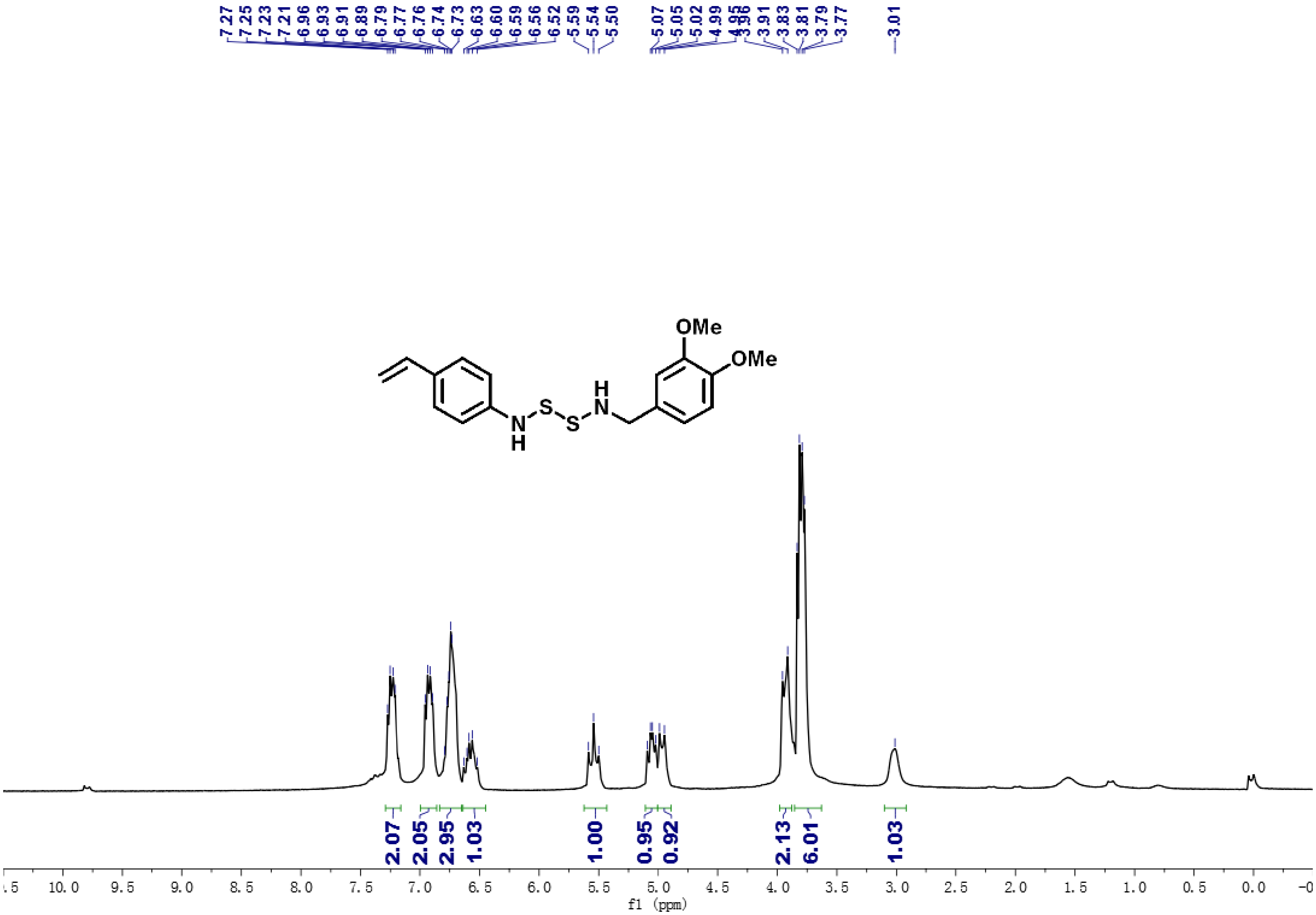

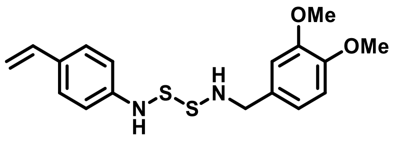

3d

^{13}C NMR (DMSO-d$_6$)

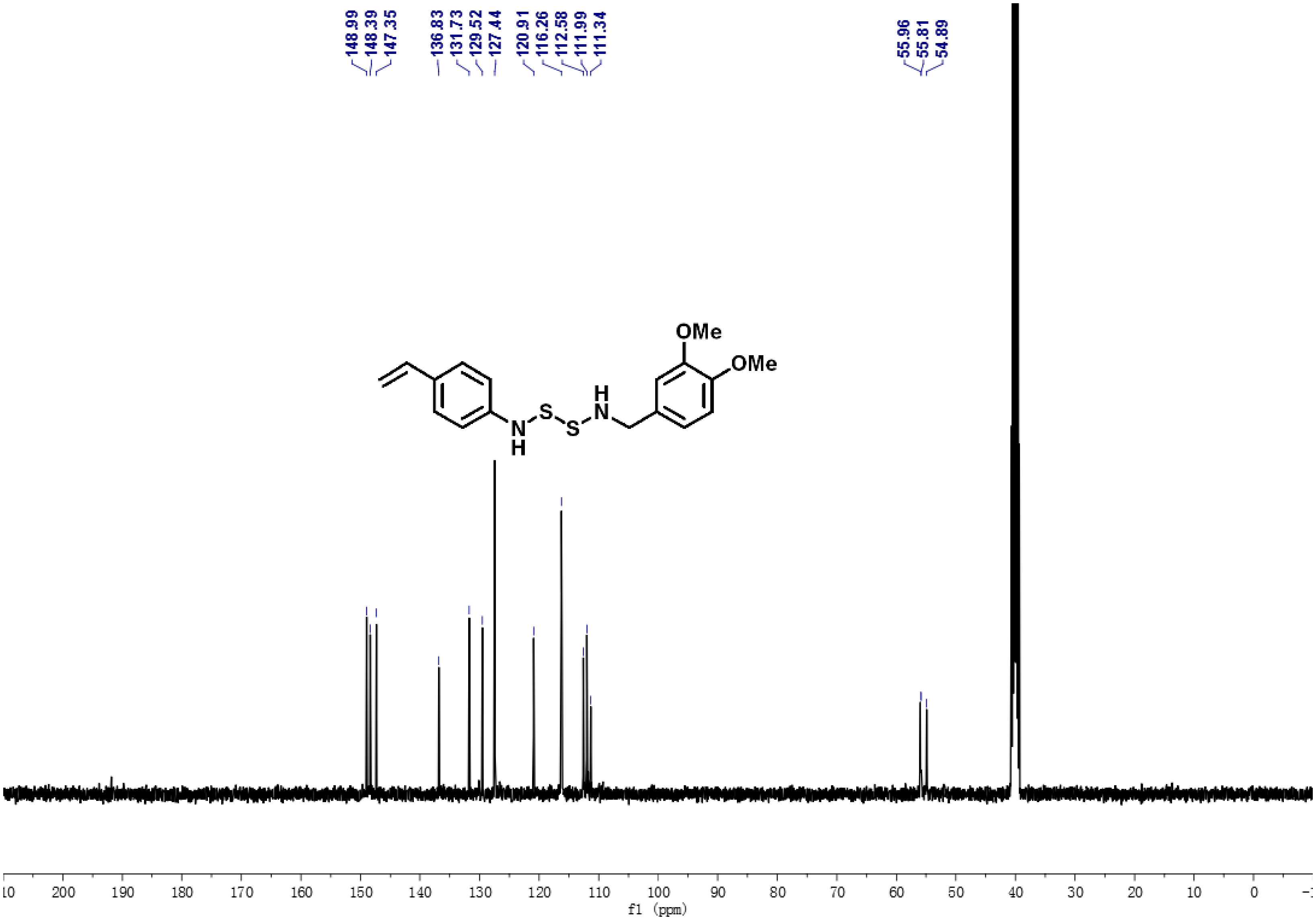

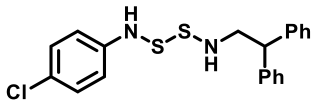

3e

^{1}H NMR ($CDCl_3$)

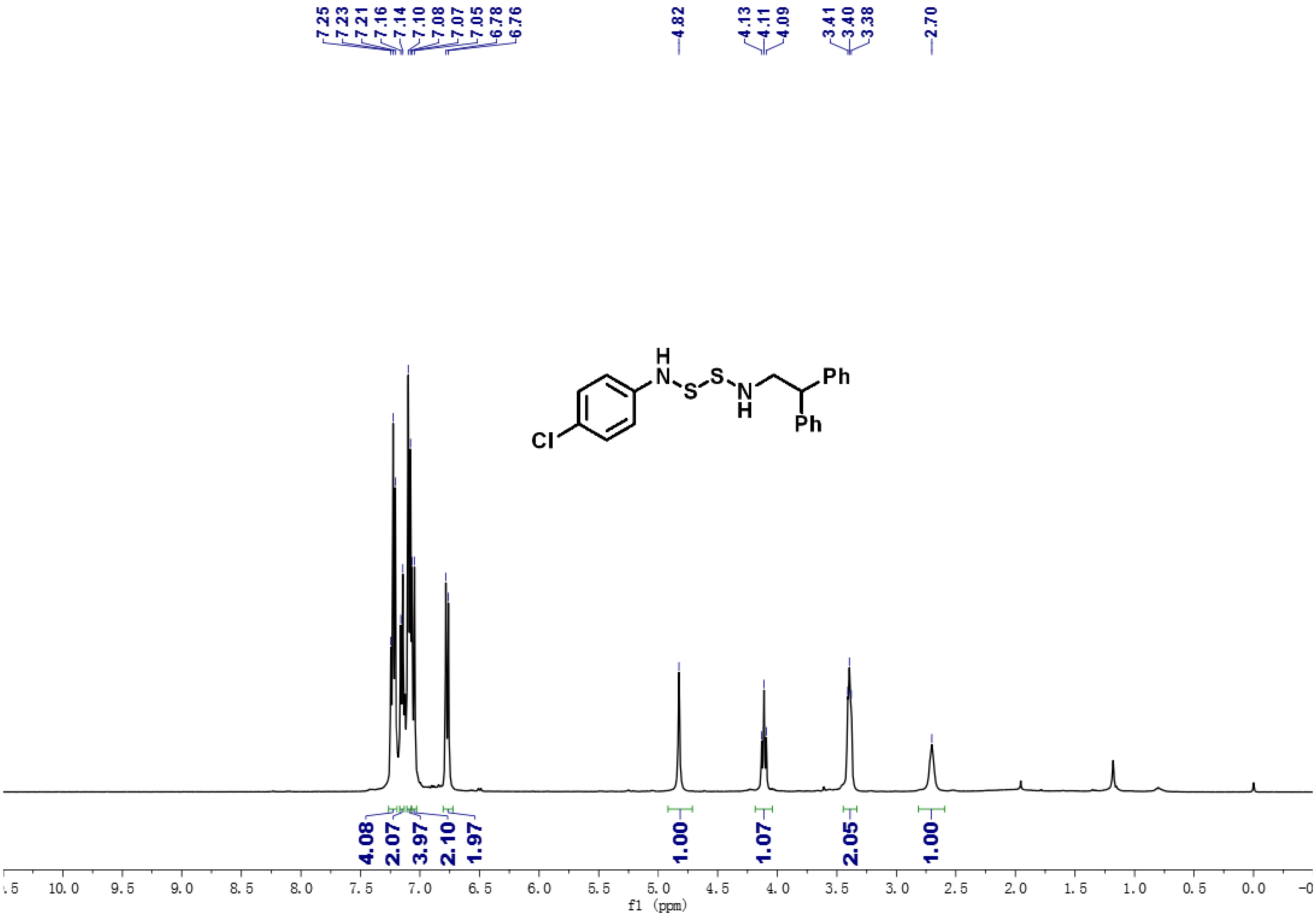

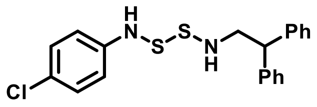

3e

^{13}C NMR ($CDCl_3$)

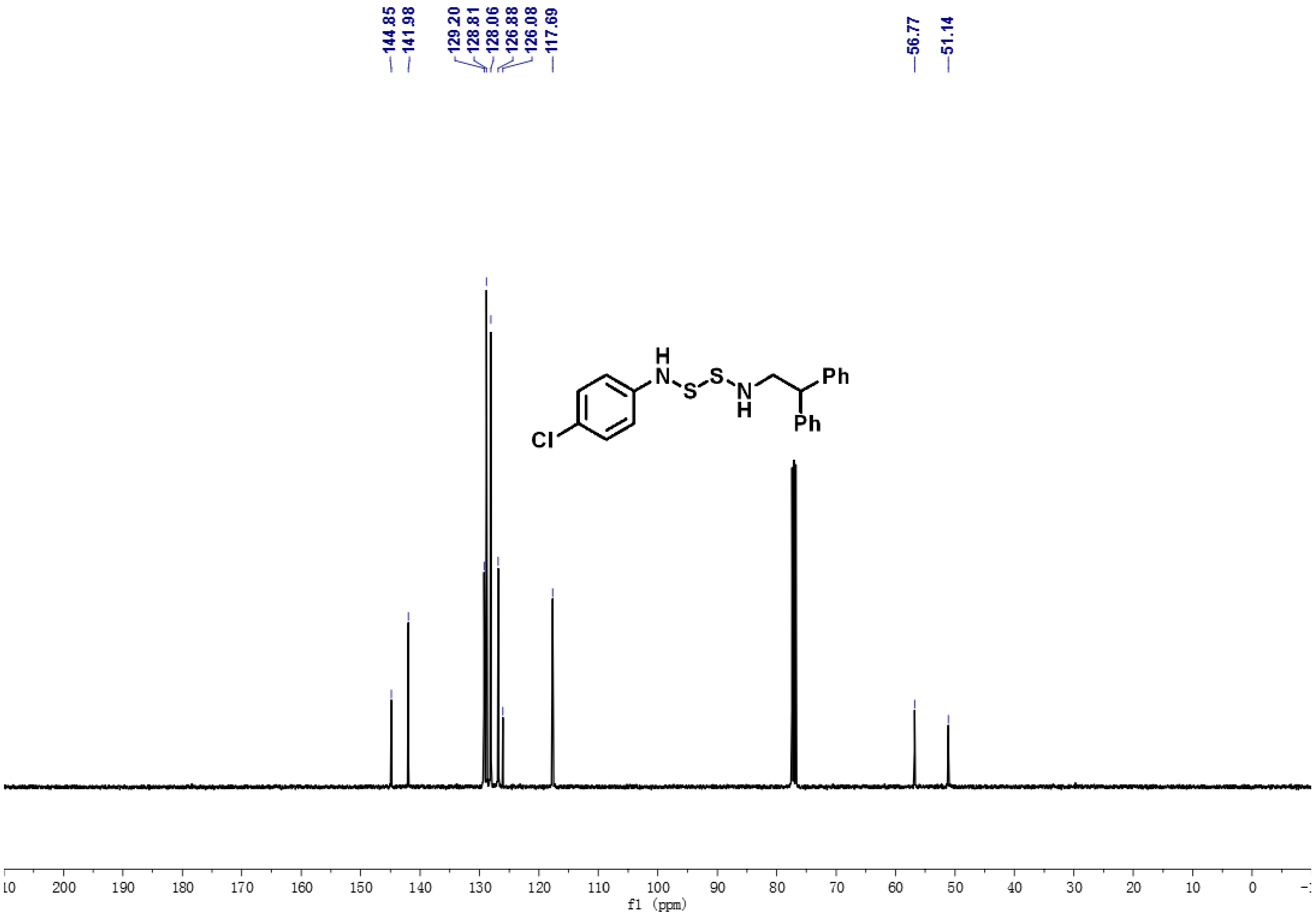

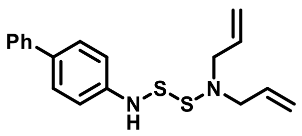

3f

^{1}H NMR ($CDCl_3$)

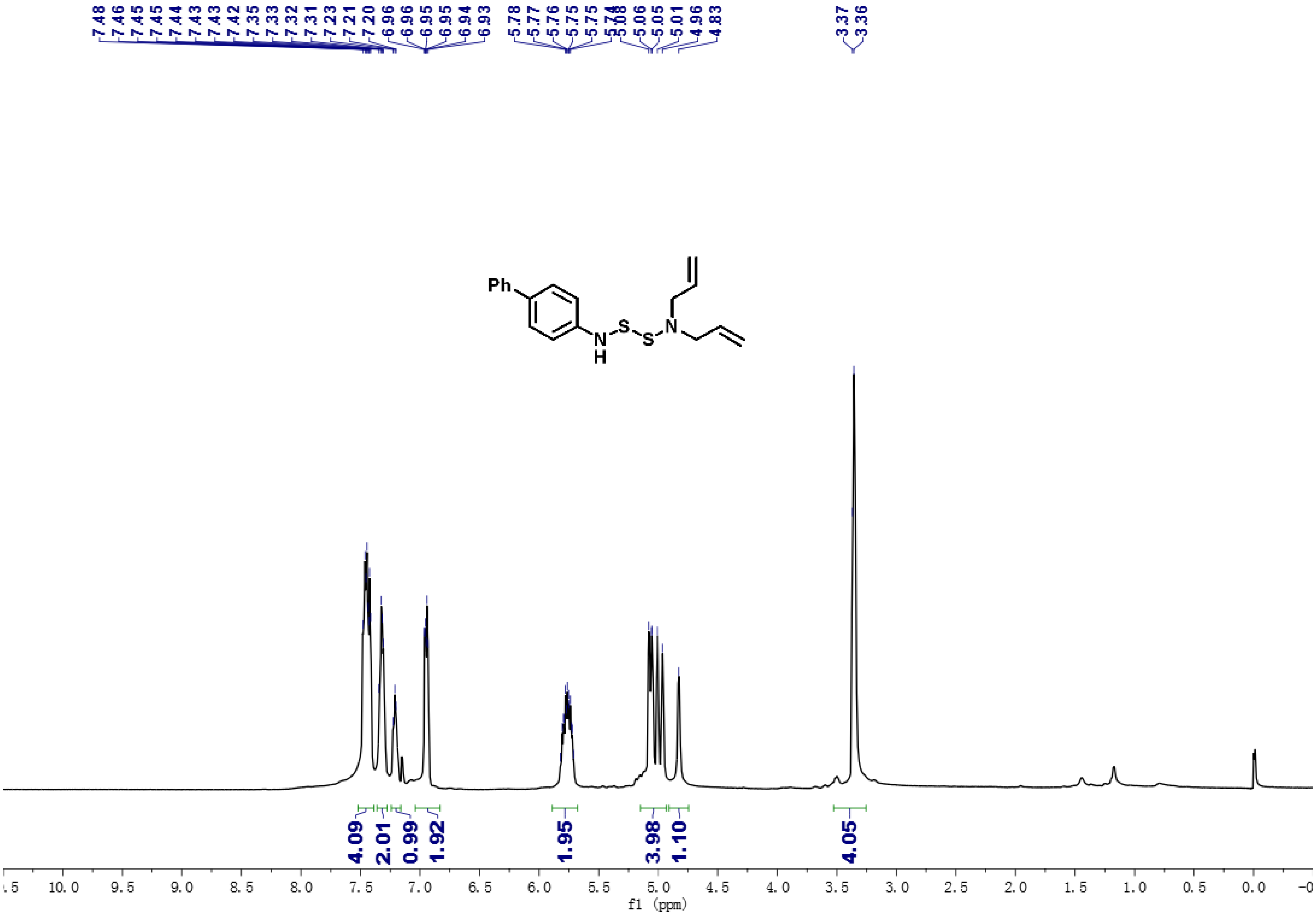

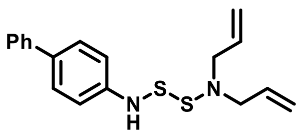

3f

^{13}C NMR ($CDCl_3$)

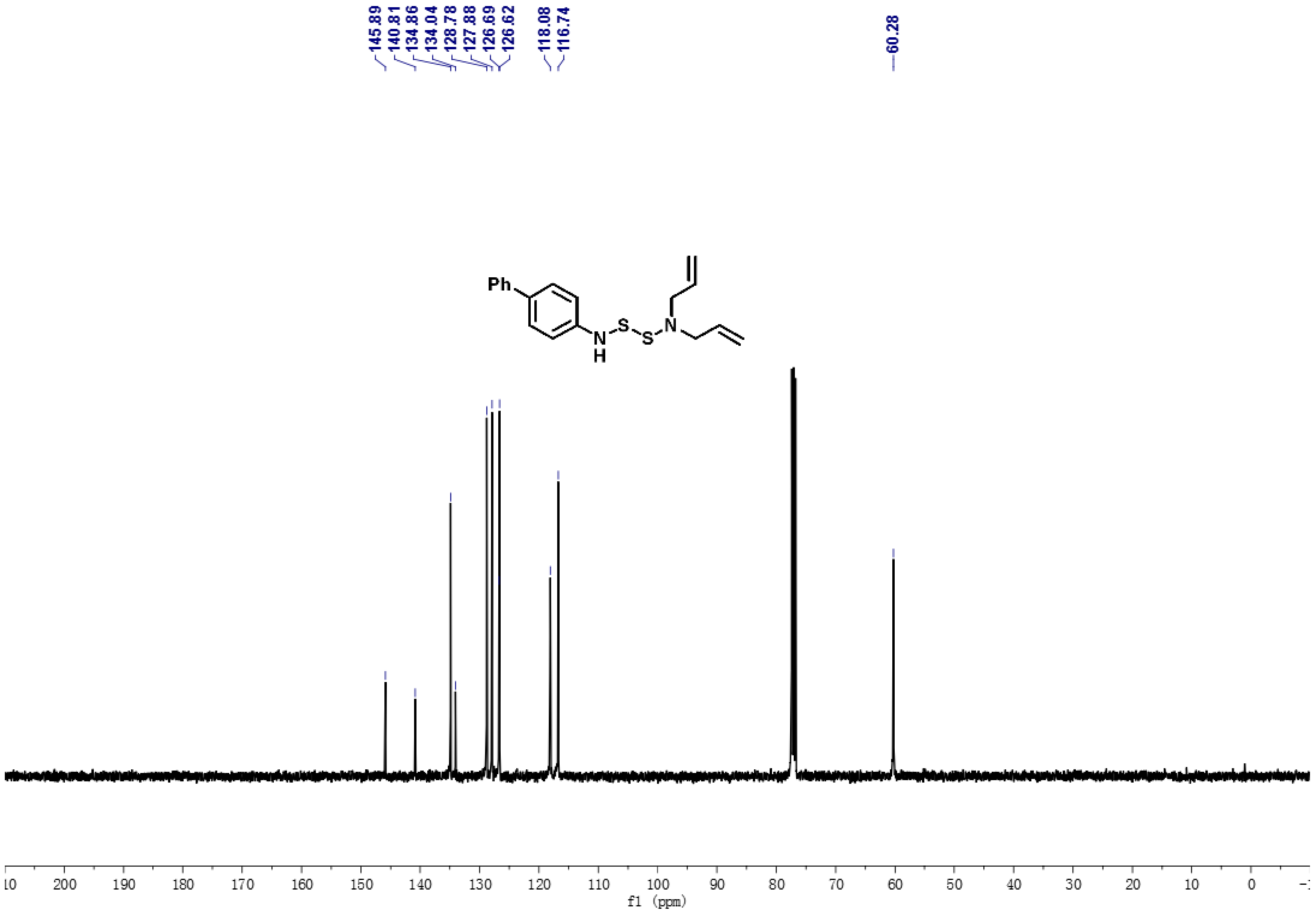

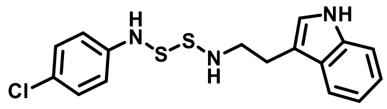

3g

^{1}H NMR ($CDCl_3$)

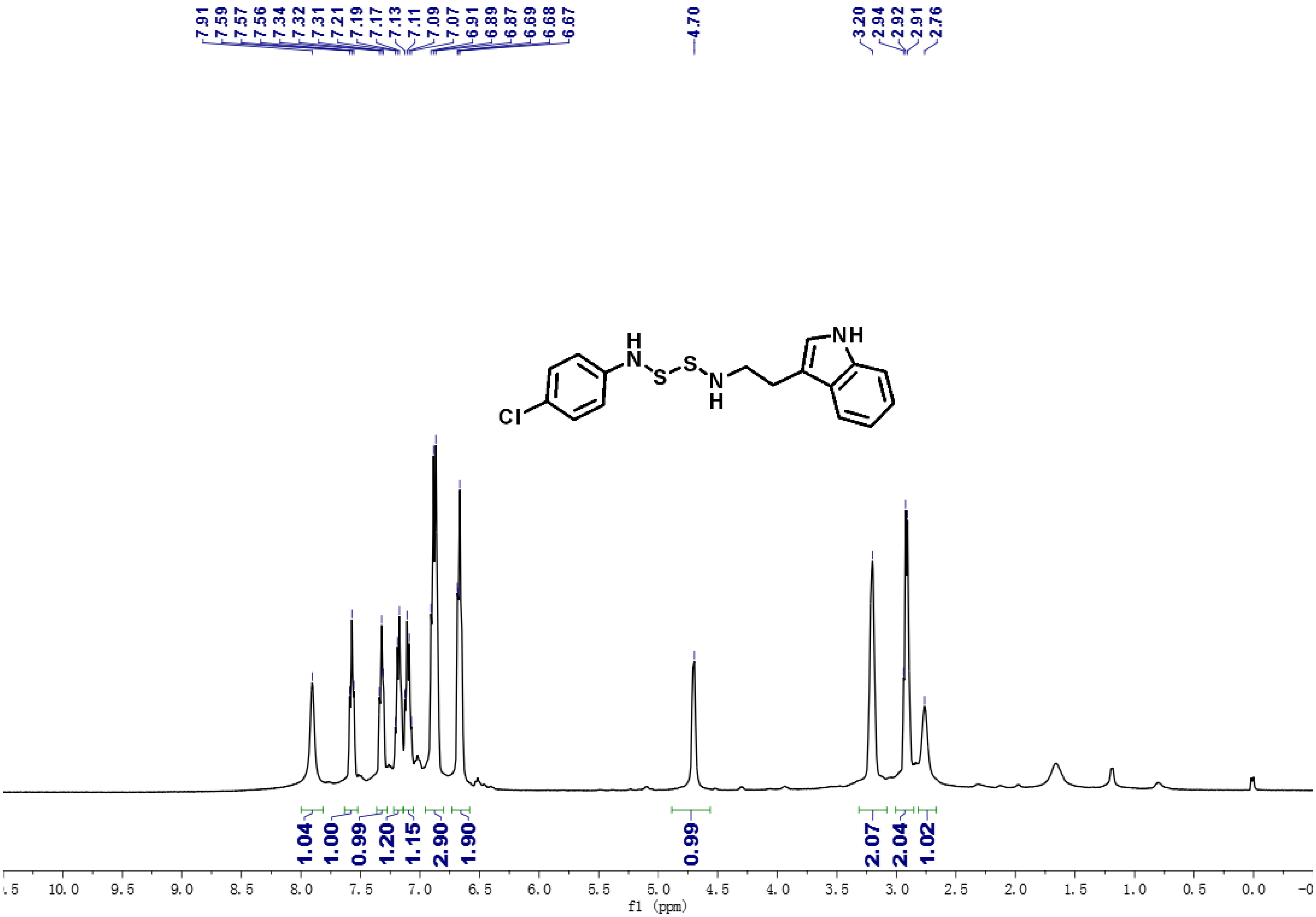

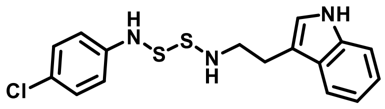

3g

^{13}C NMR ($CDCl_3$)

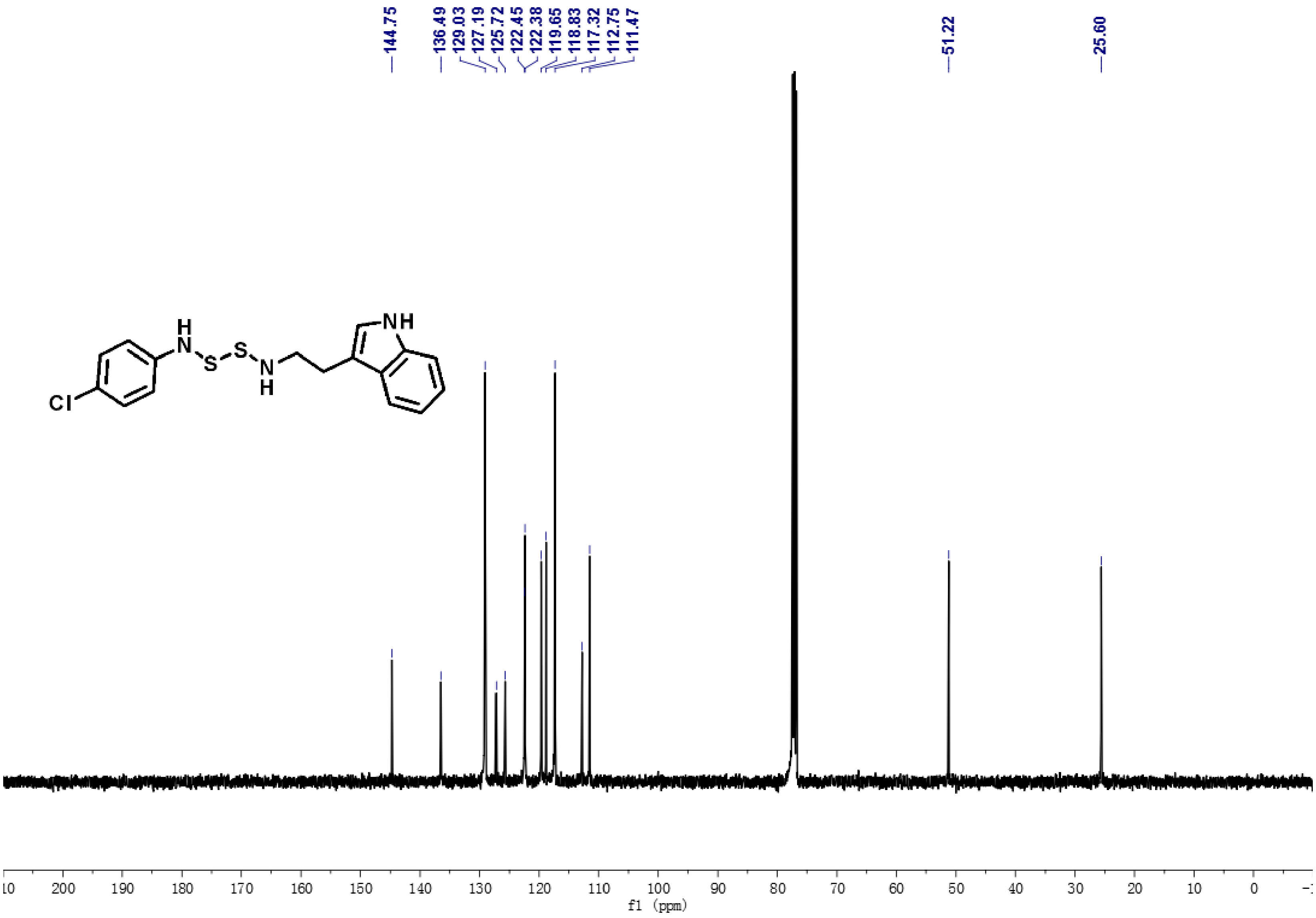

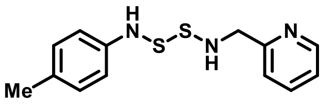

3h

^{1}H NMR (DMSO-d6)

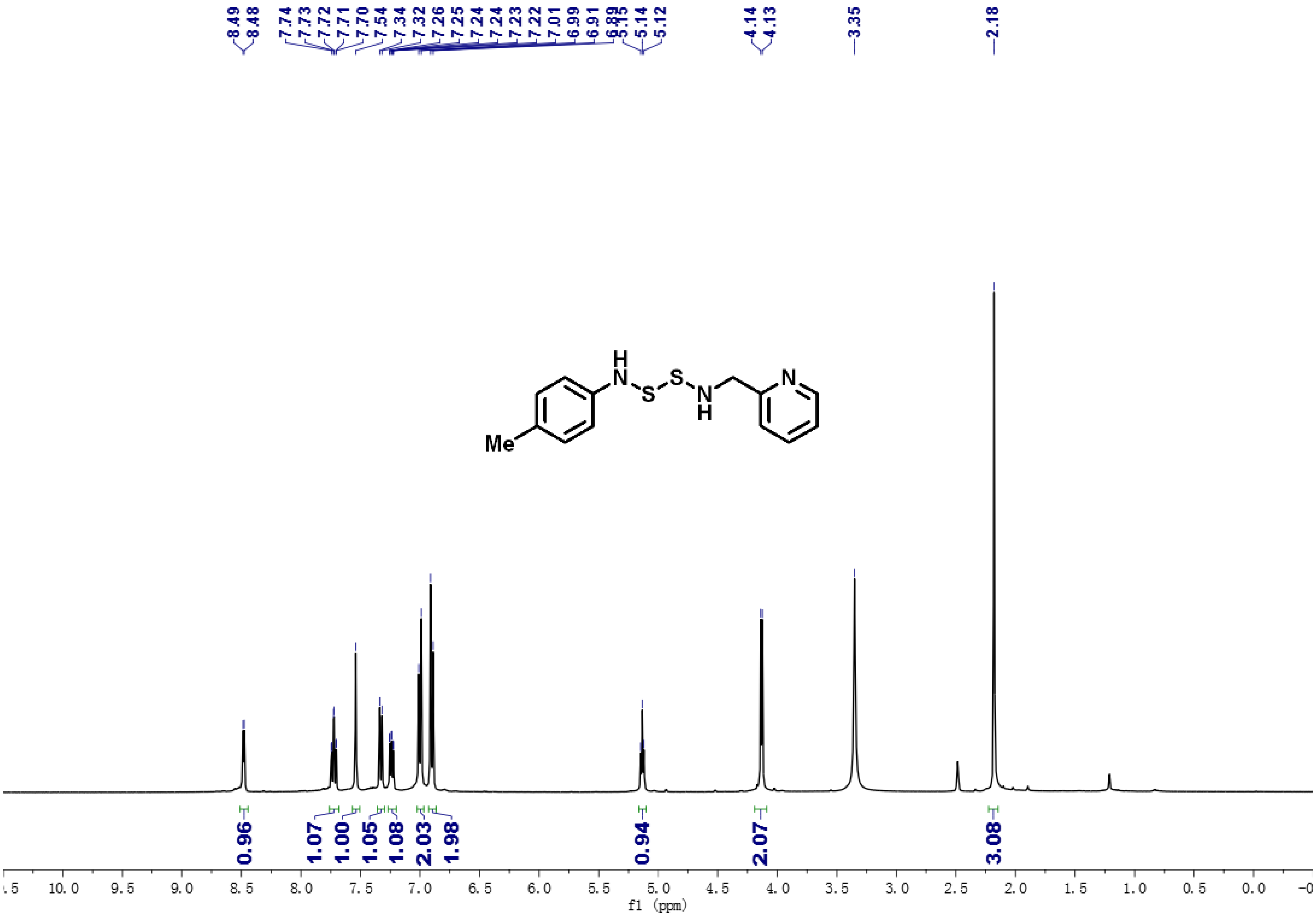

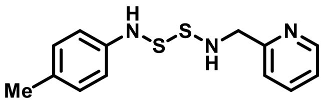

3h

^{13}C NMR (DMSO-d$_6$)

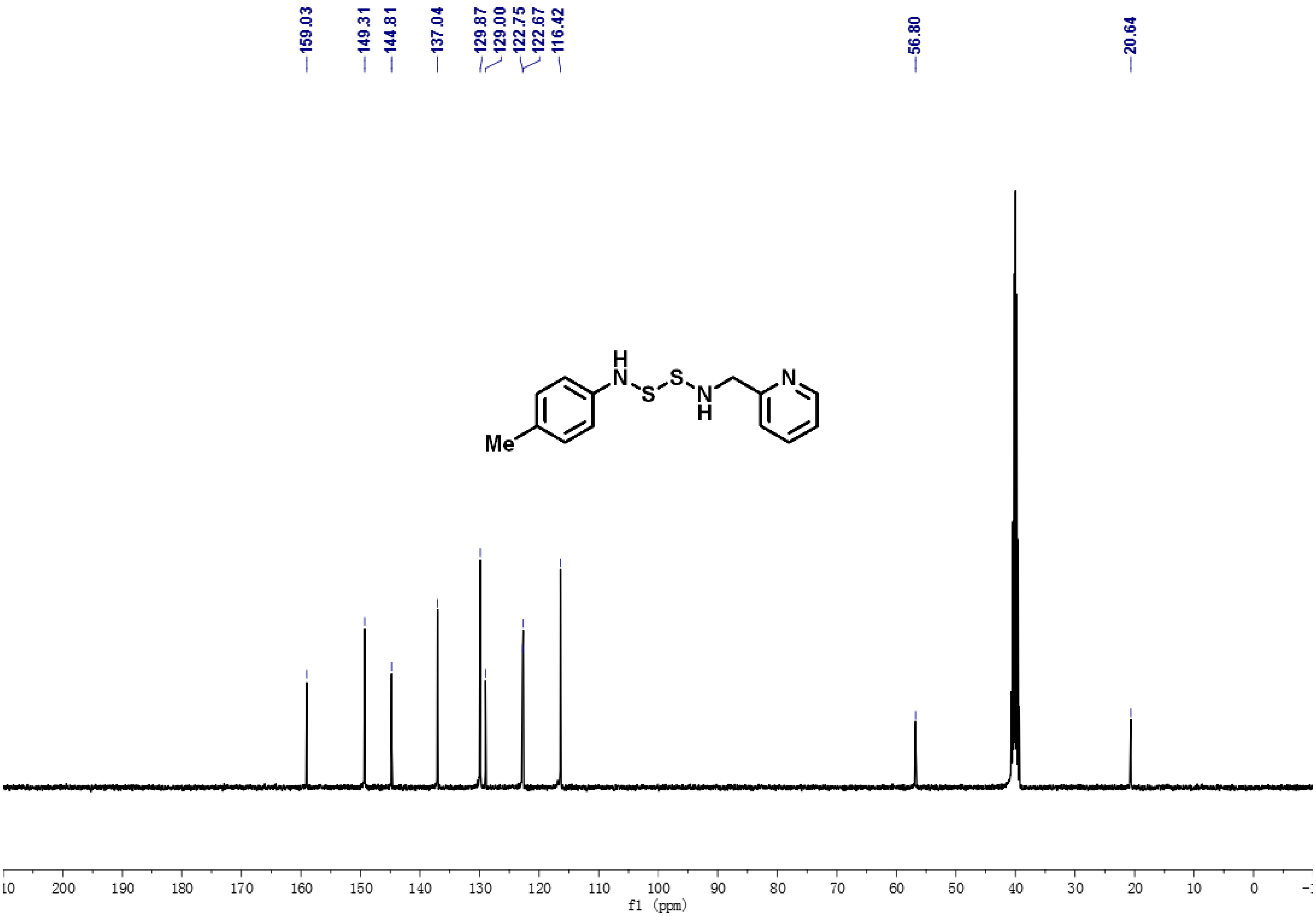

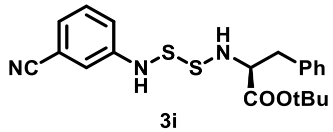

3i

^{1}H NMR ($CDCl_3$)

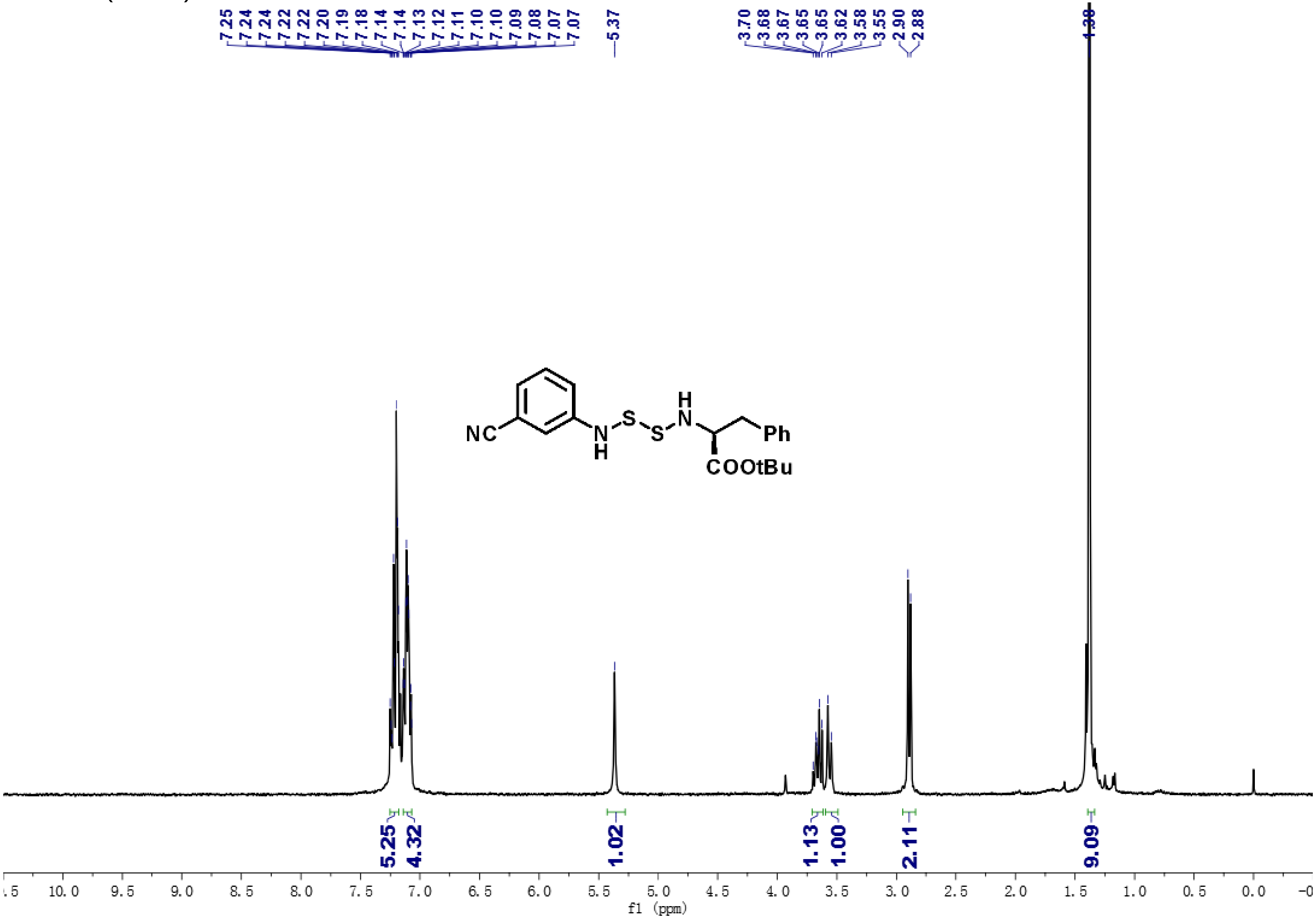

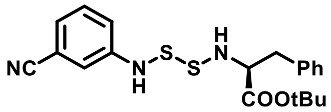

3i

^{13}C NMR ($CDCl_3$)

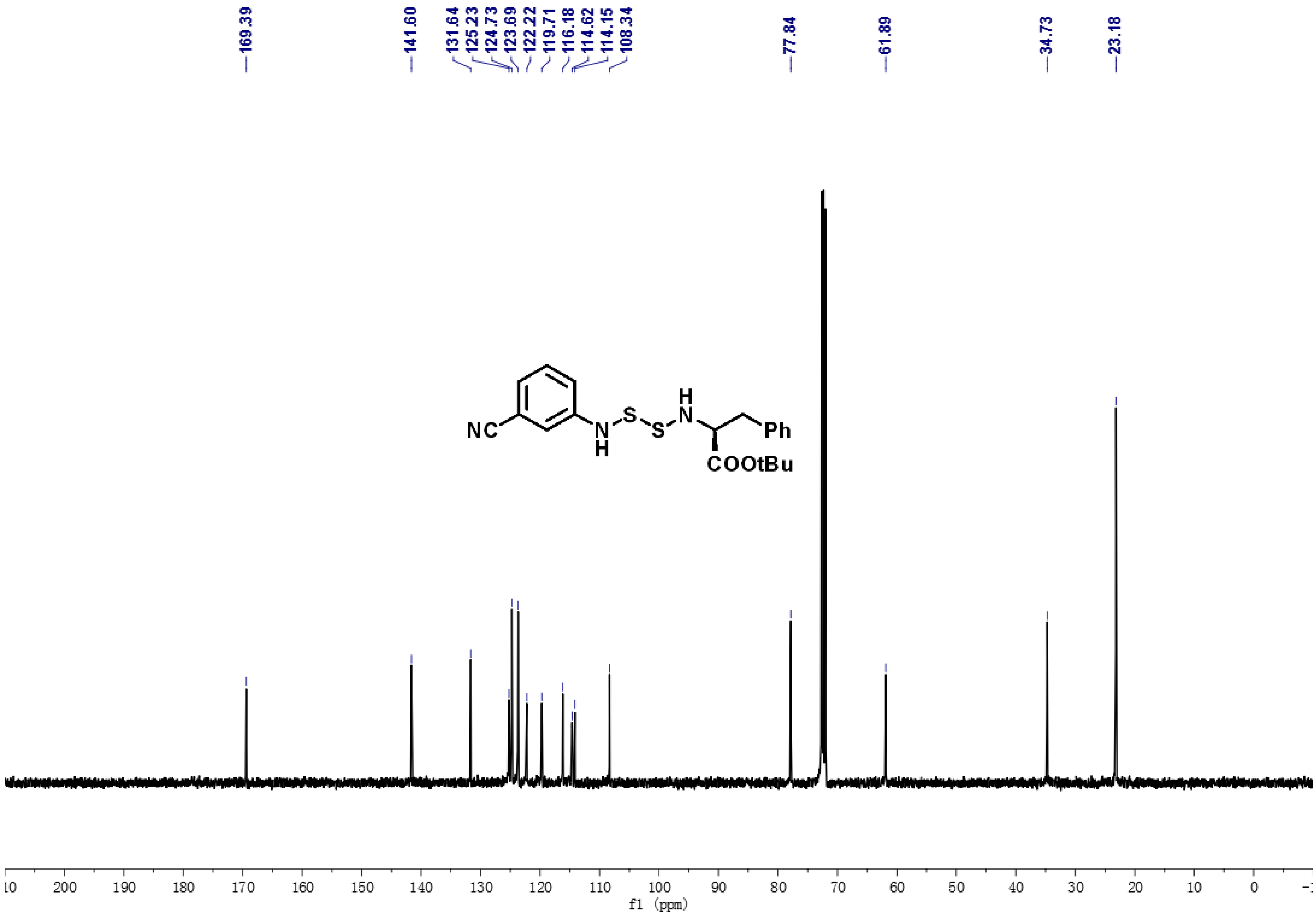

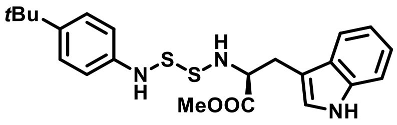

3j

¹H NMR (CDCl₃)

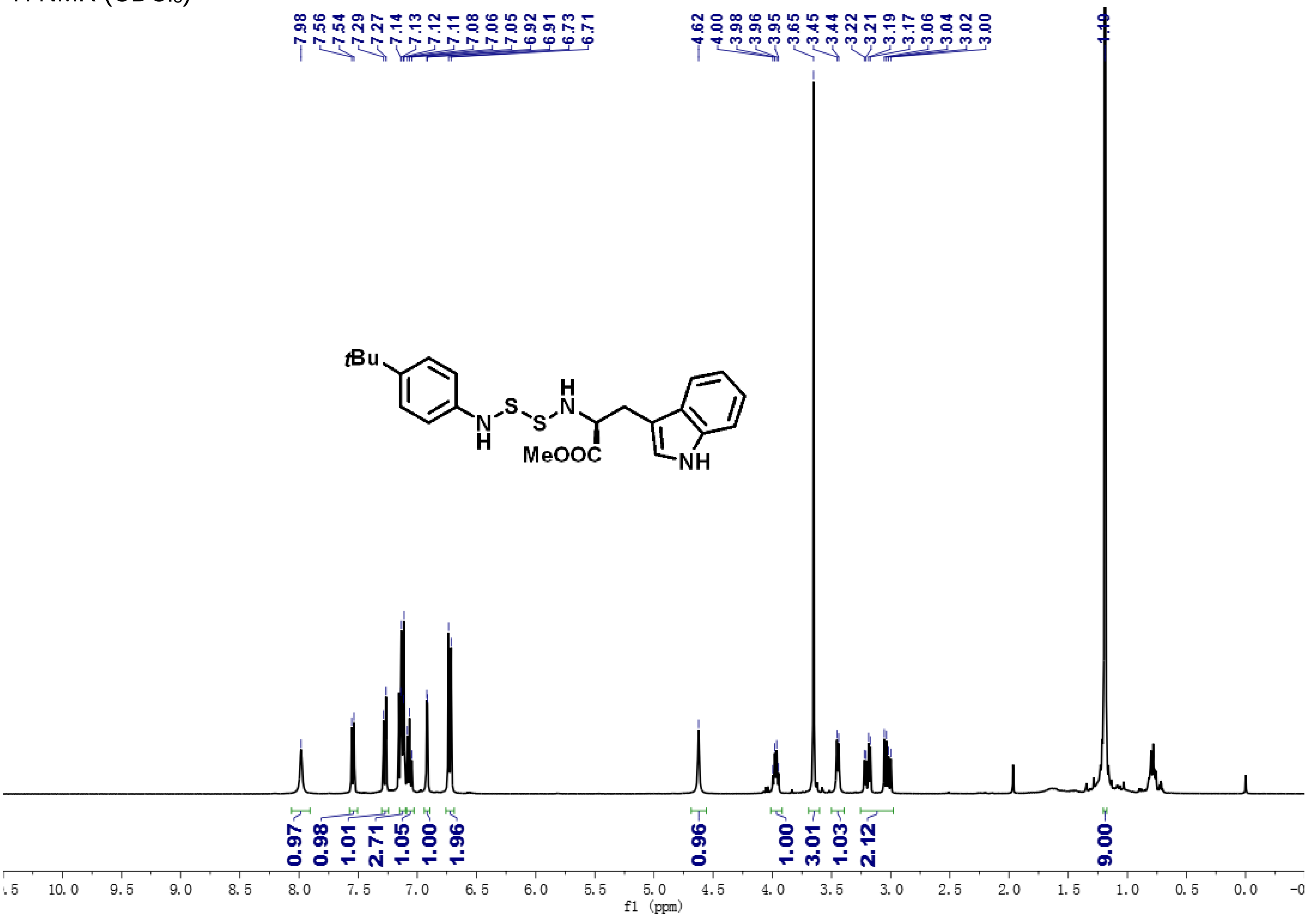

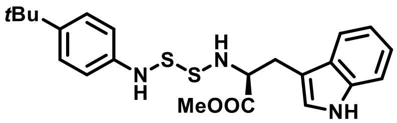

3j

^{13}C NMR ($CDCl_3$)

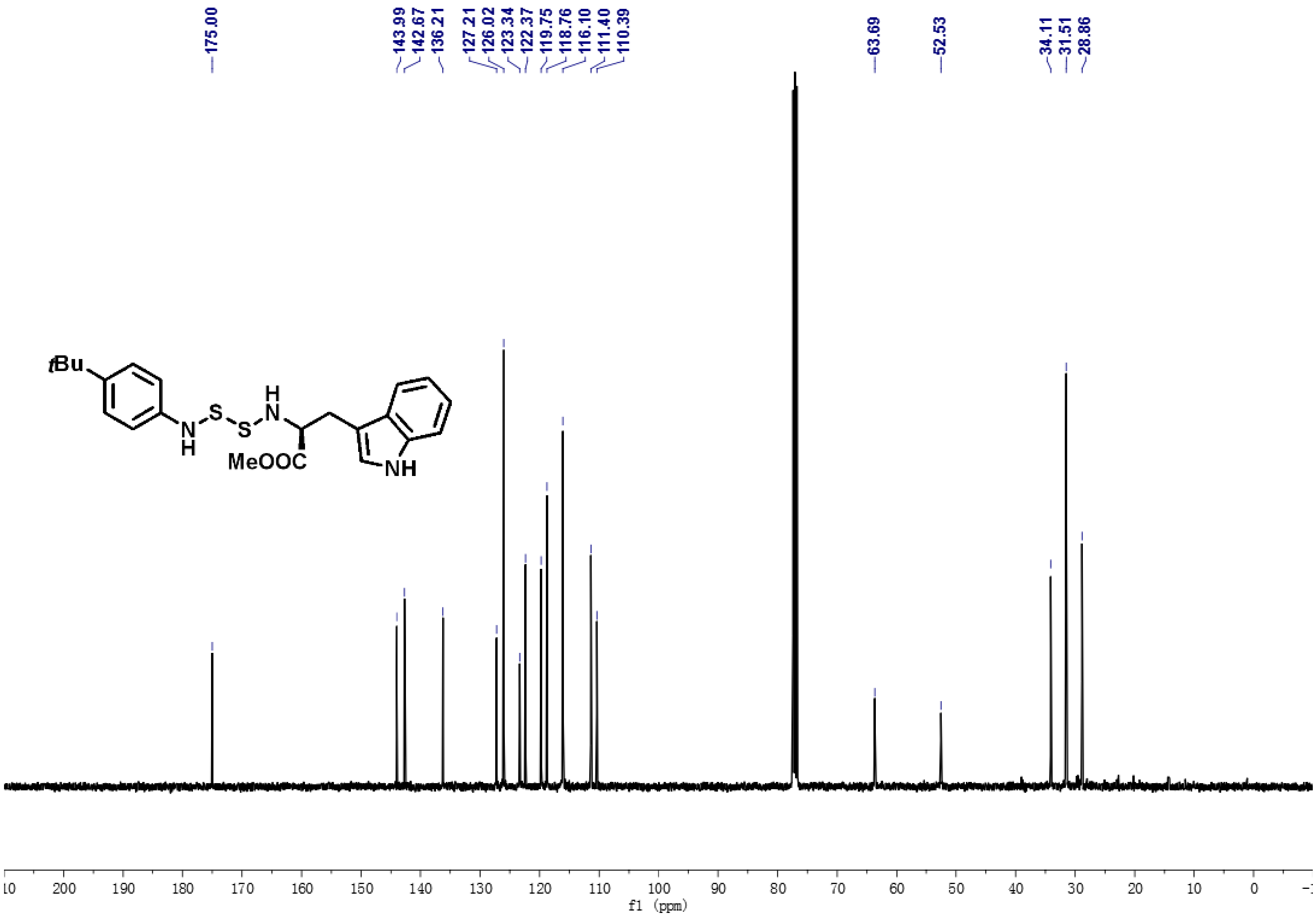

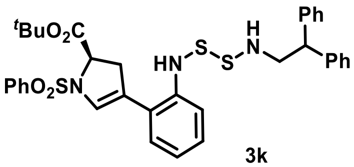

3k

^{1}H NMR (CDCl$_3$)

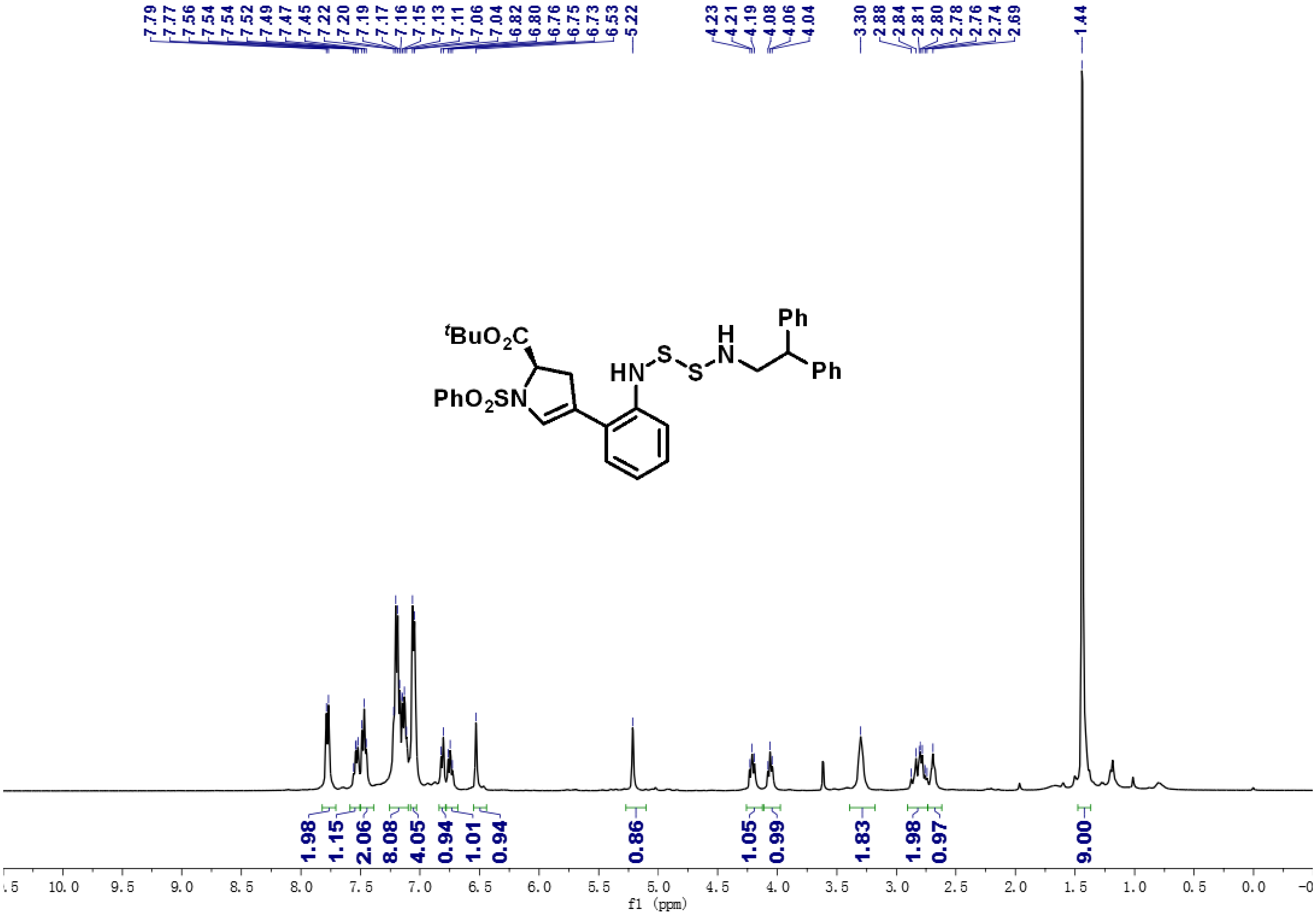

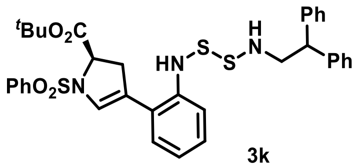

3k

^{13}C NMR (CDCl$_3$)

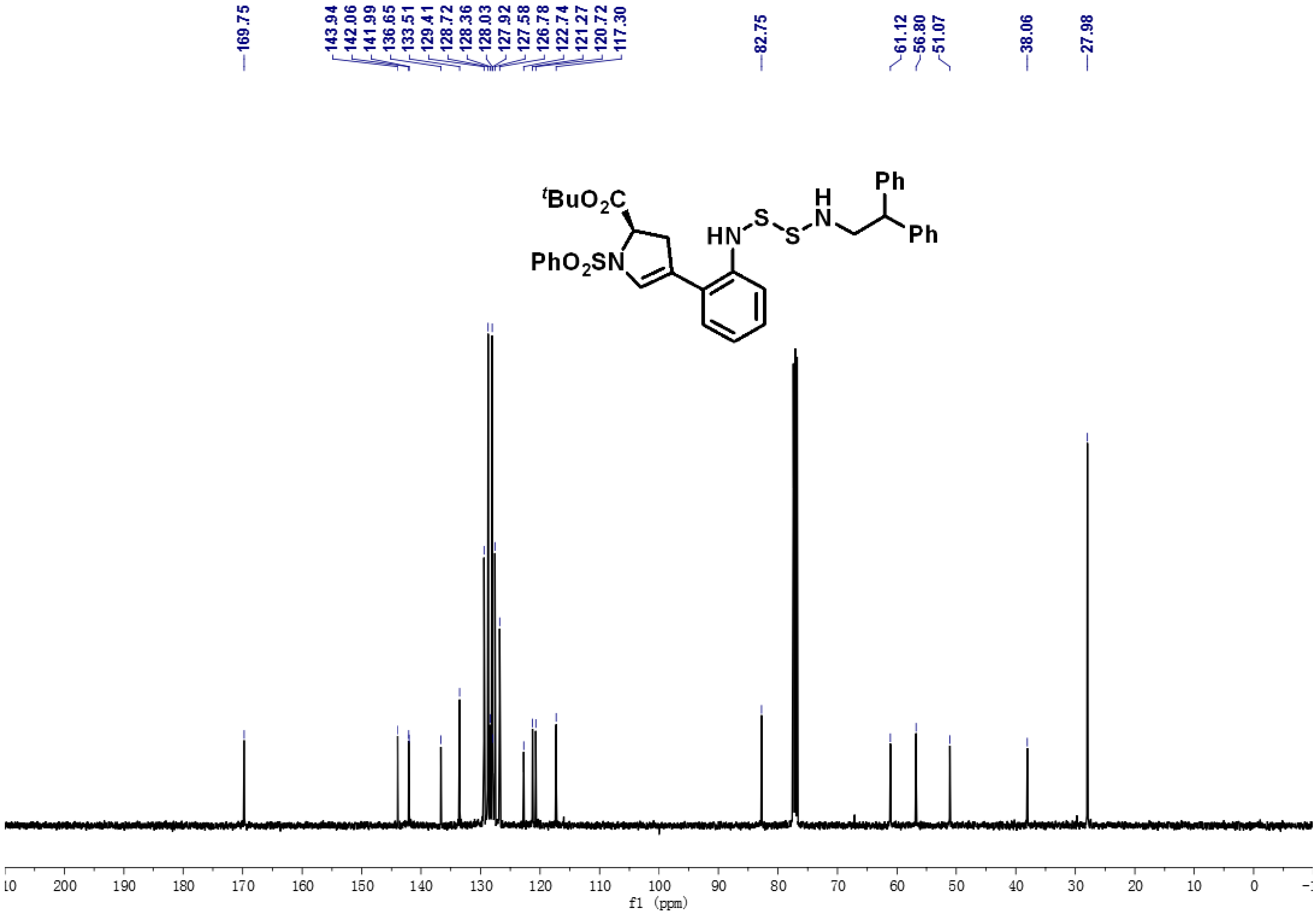

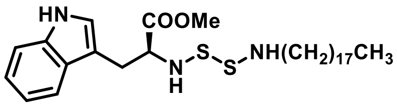

3l

^{1}H NMR ($CDCl_3$)

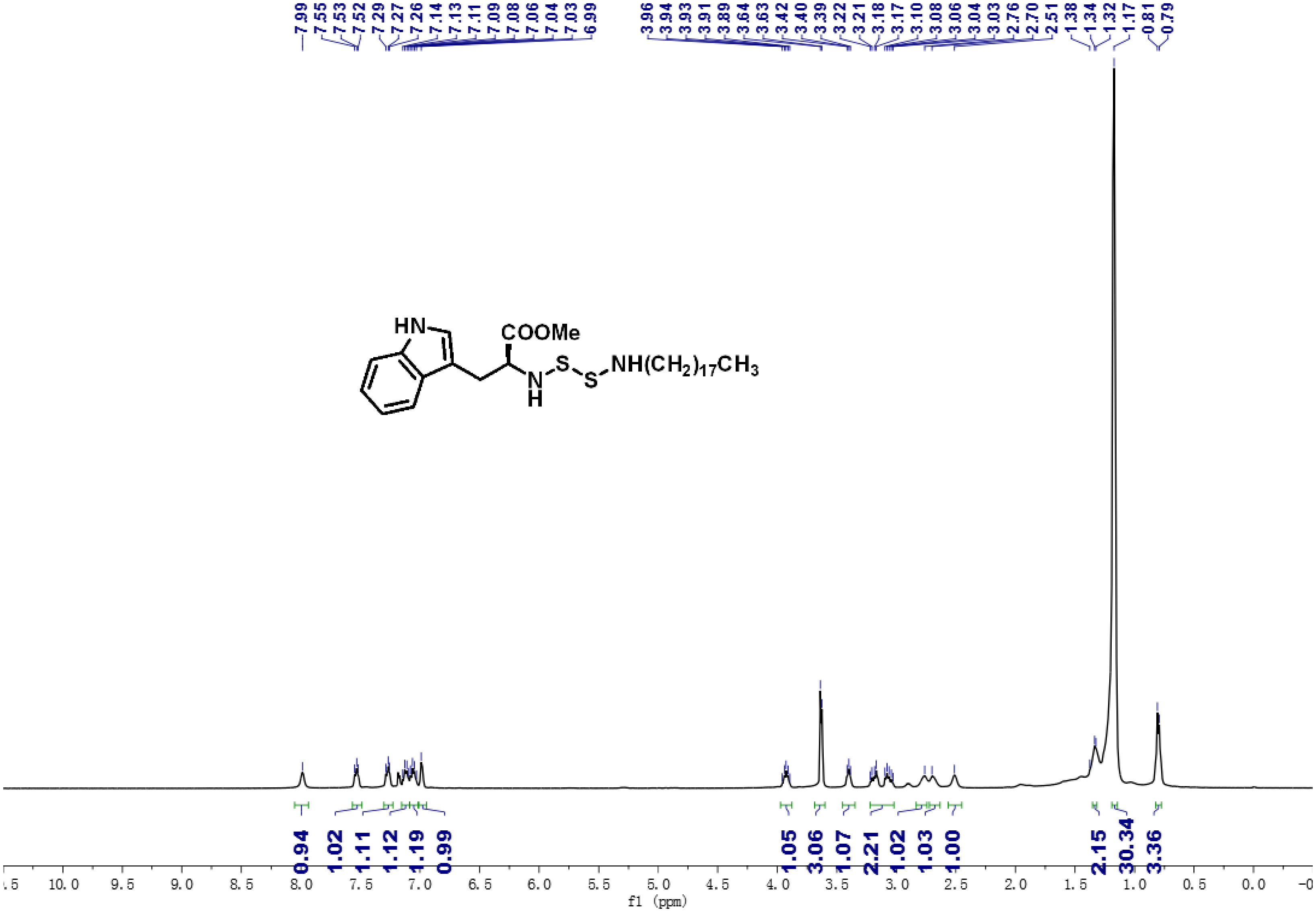

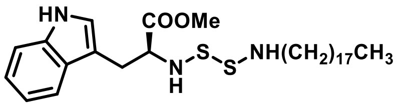

3l

^{13}C NMR ($CDCl_3$)

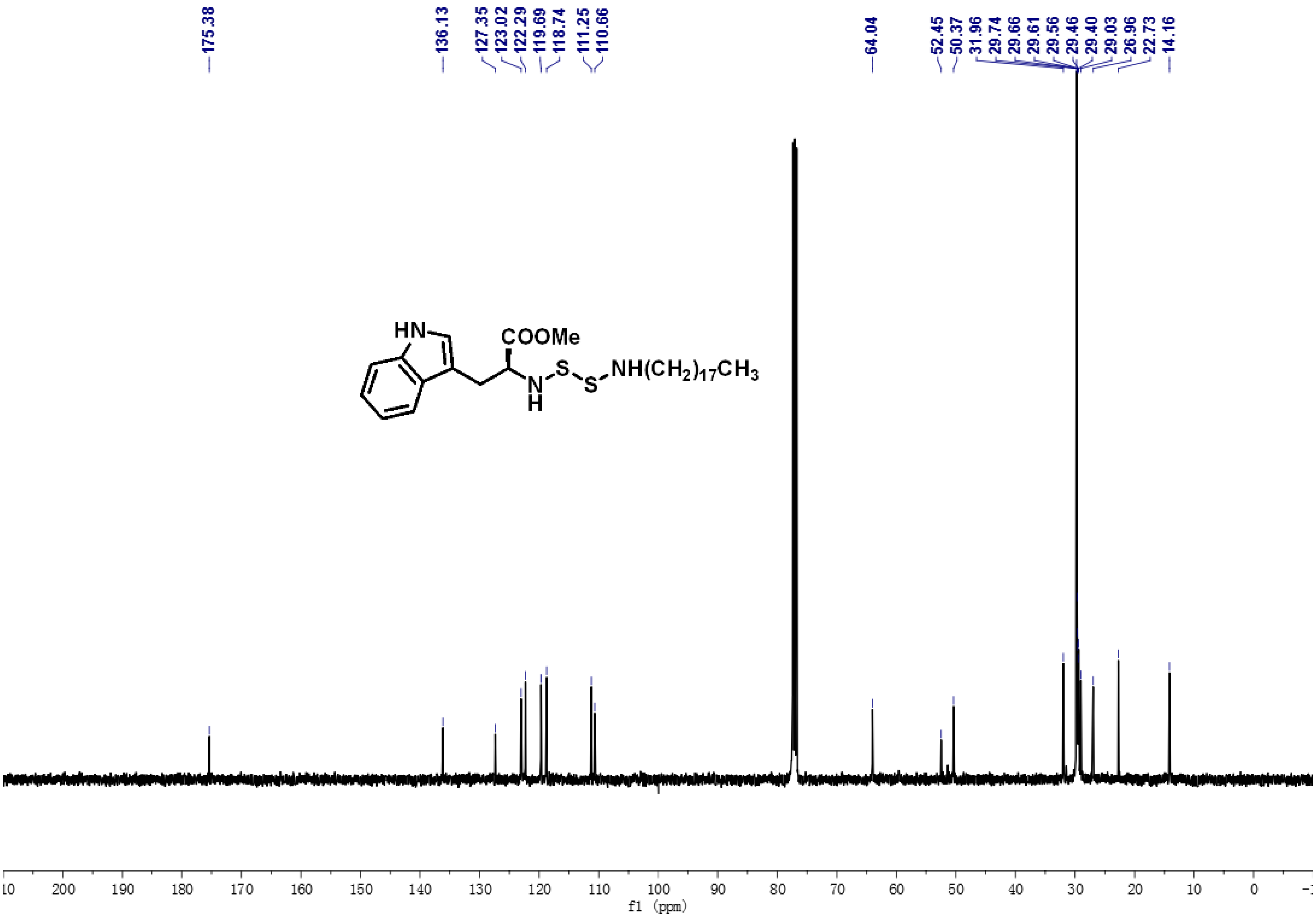

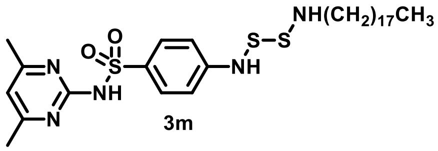

3m

^{1}H NMR (DMSO-d6)

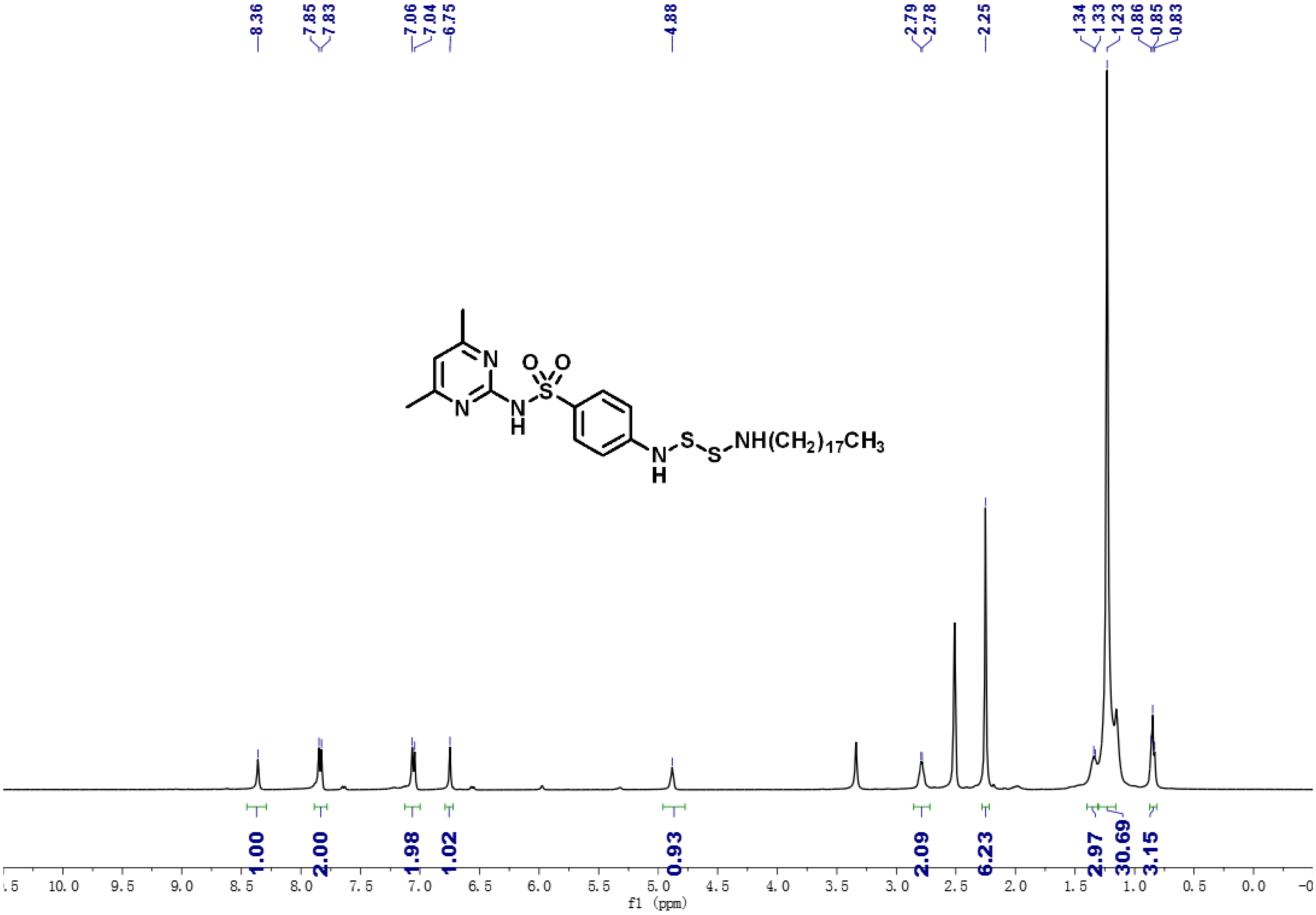

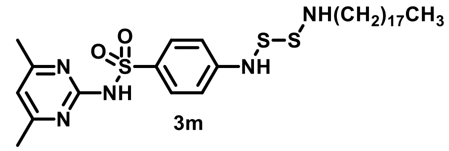

3m

^{13}C NMR (DMSO-d$_6$)

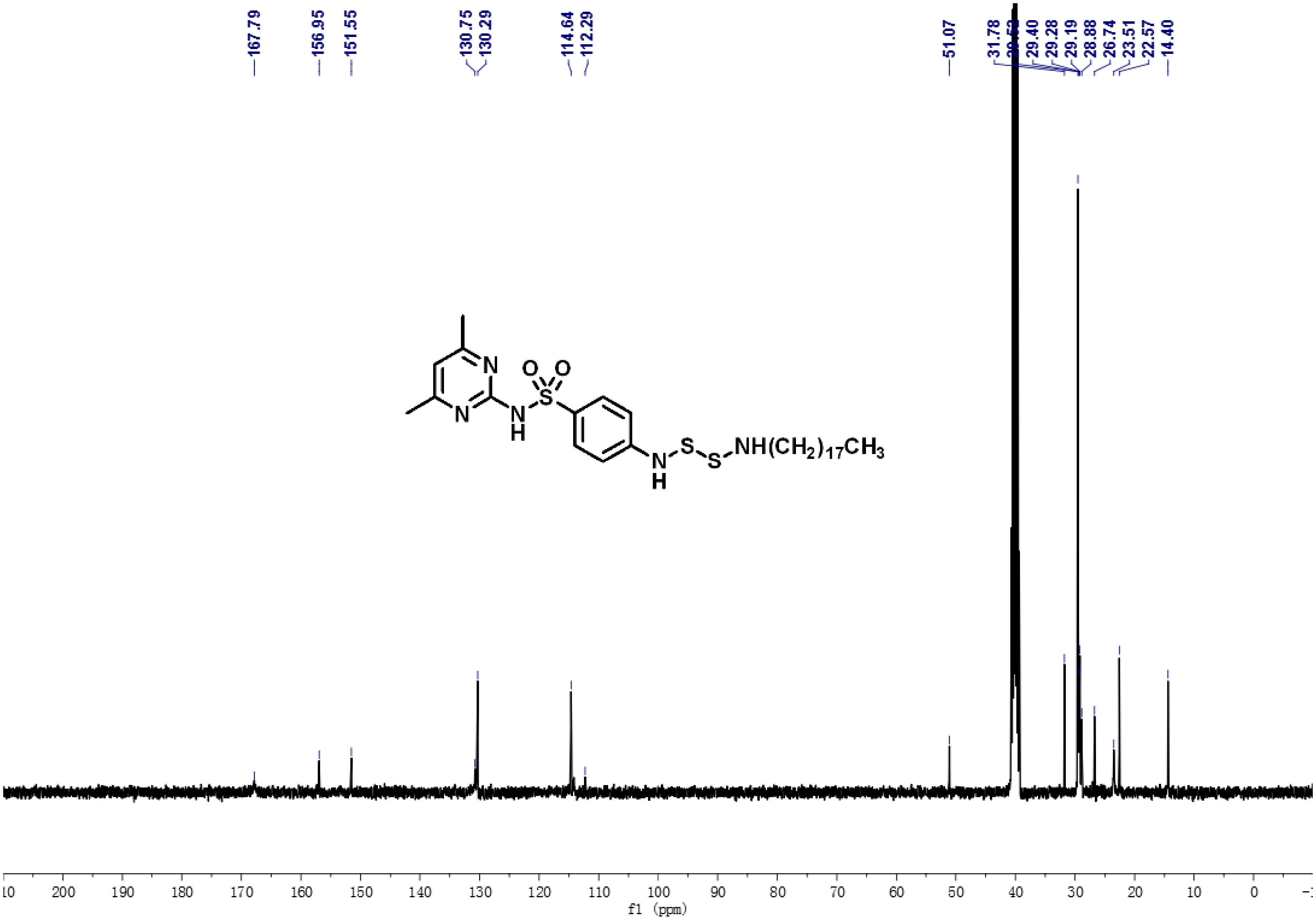

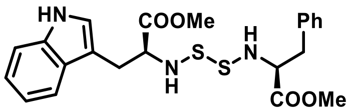

3n

^{1}H NMR ($CDCl_3$)

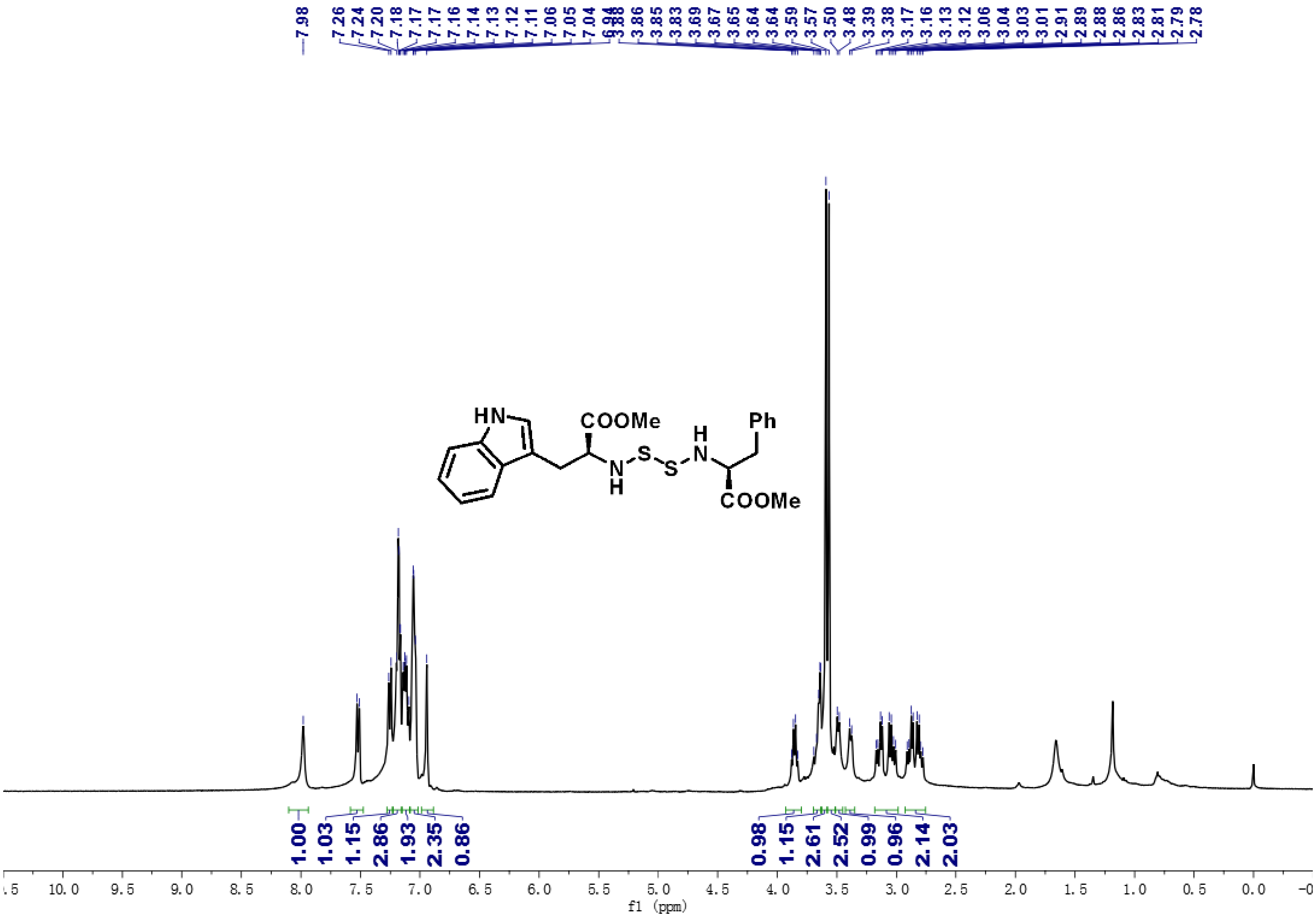

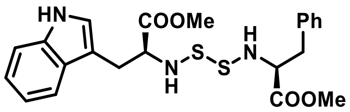

3n

^{13}C NMR ($CDCl_3$)

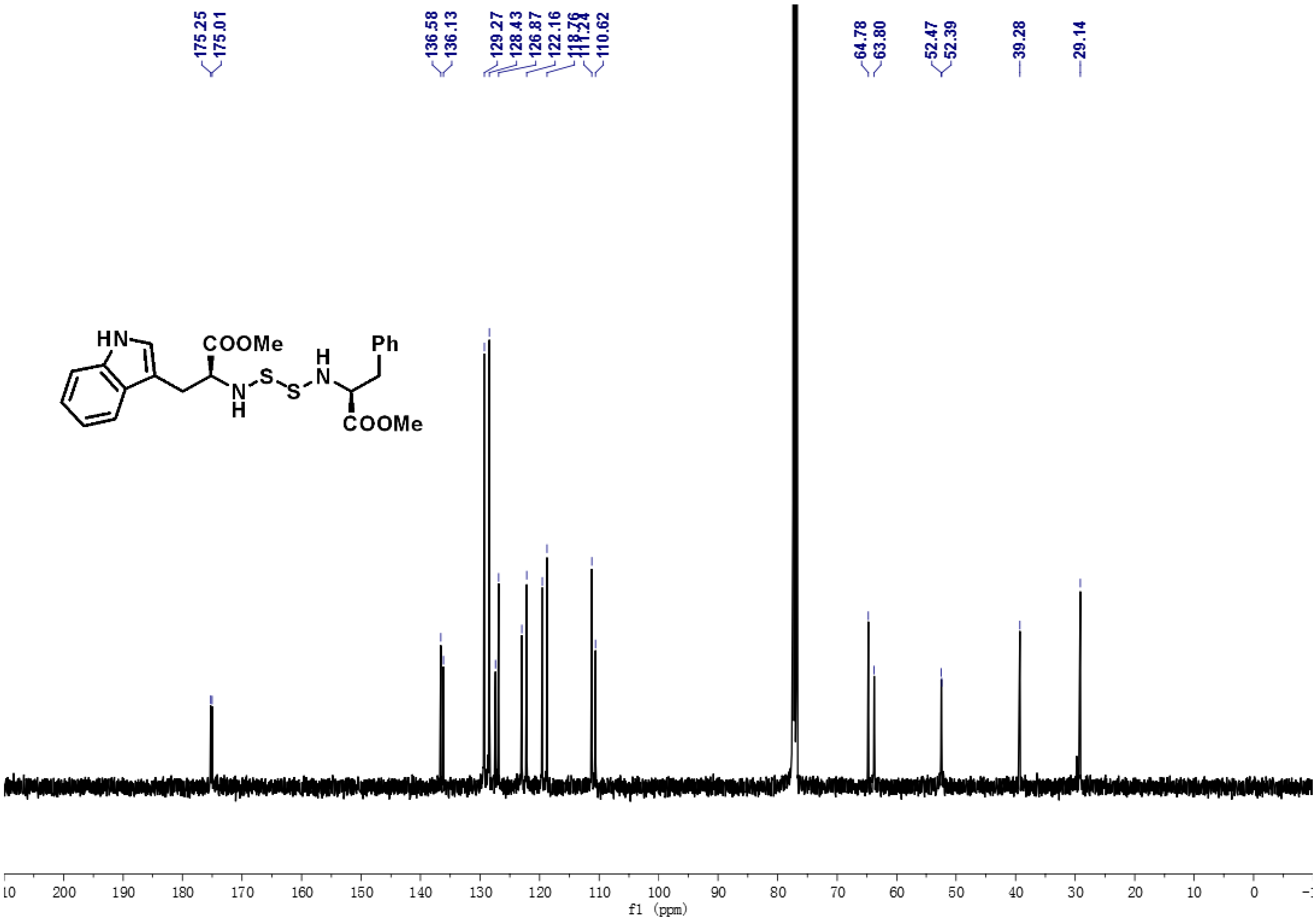

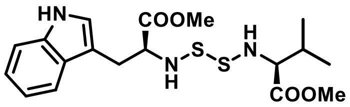

3o

^{1}H NMR ($CDCl_3$)

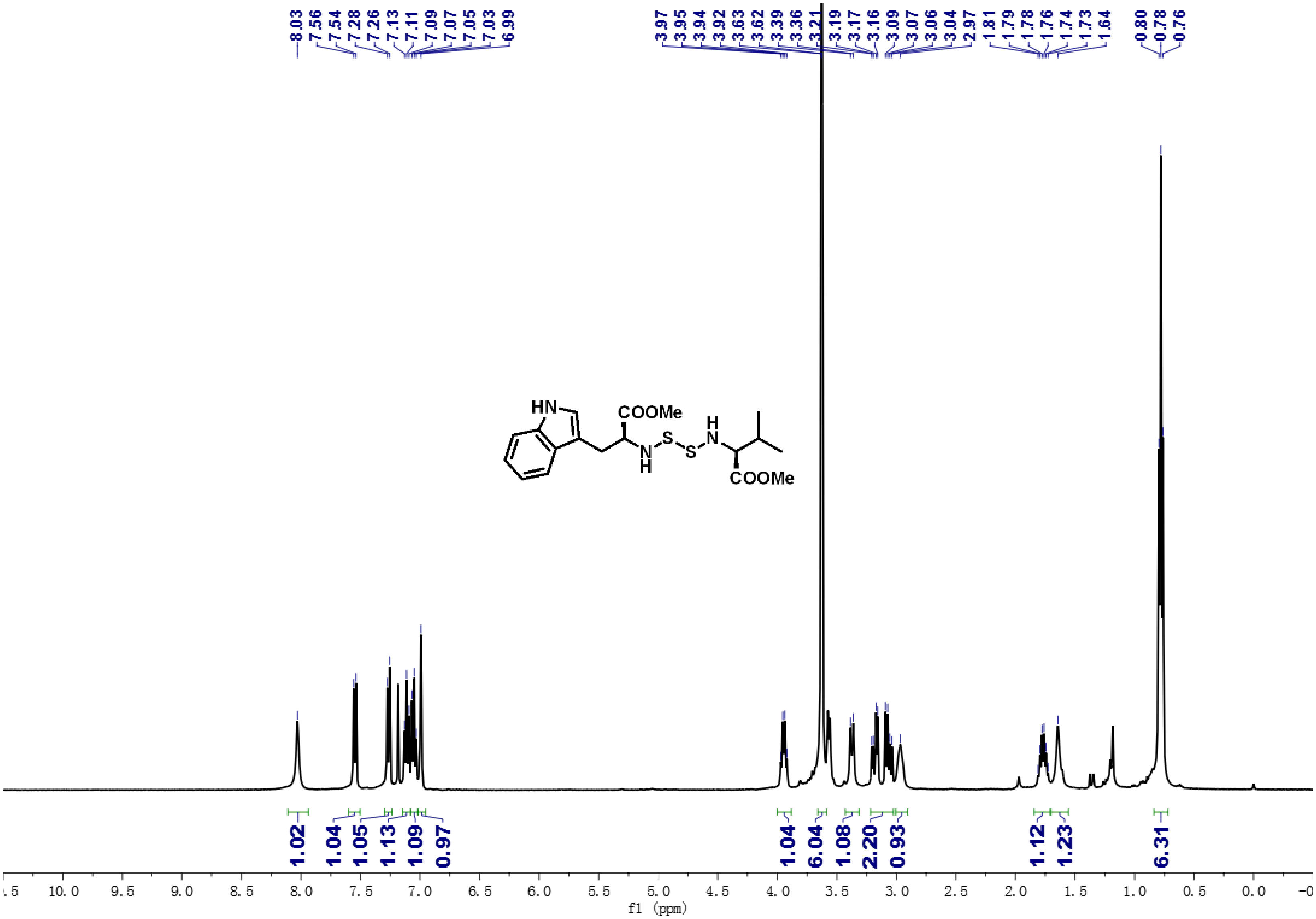

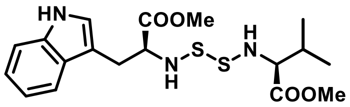

3o

^{13}C NMR ($CDCl_3$)

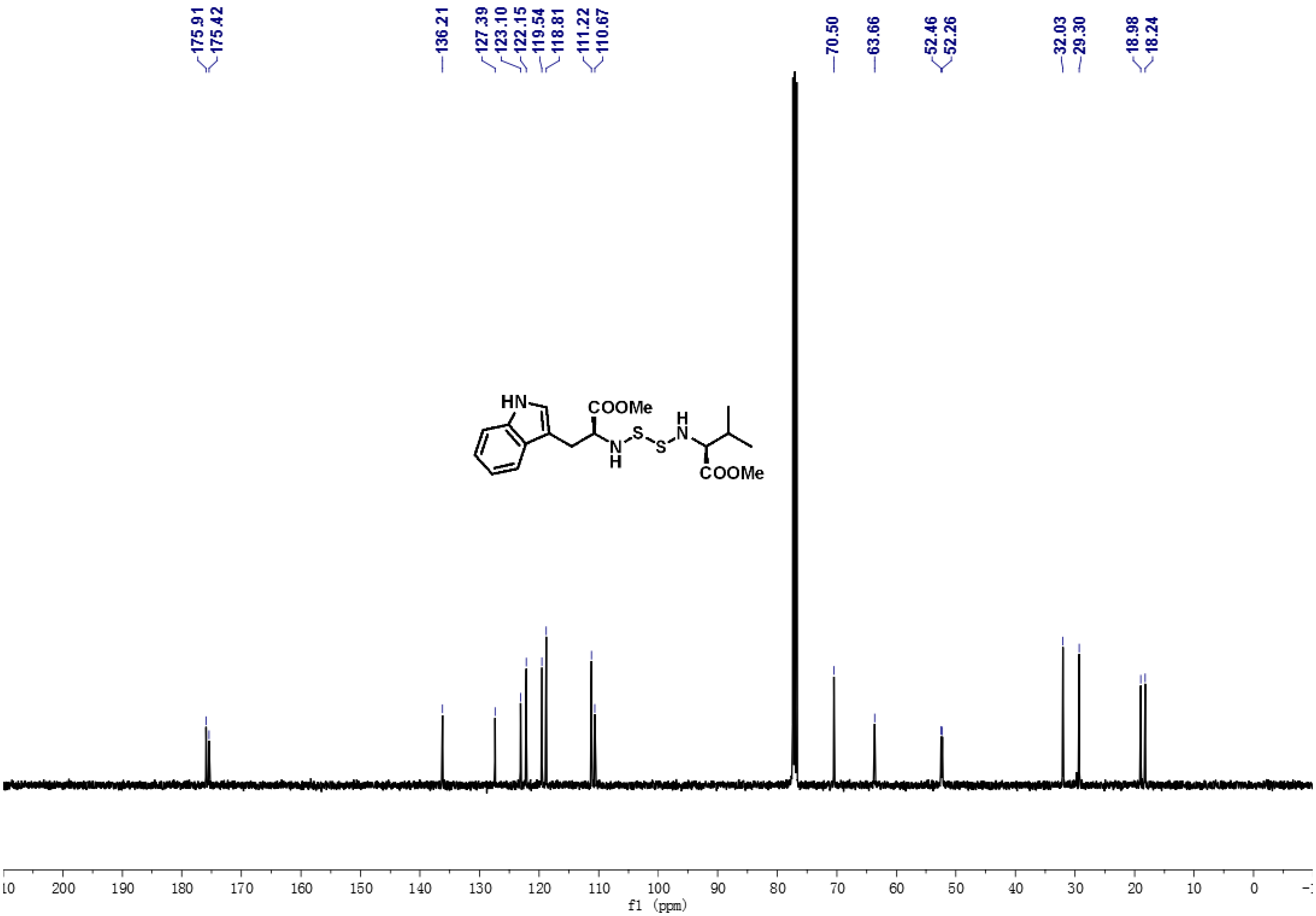

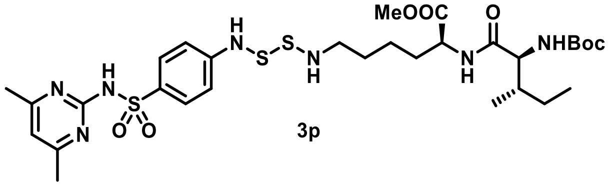

3p

^{1}H NMR ($CDCl_3$)

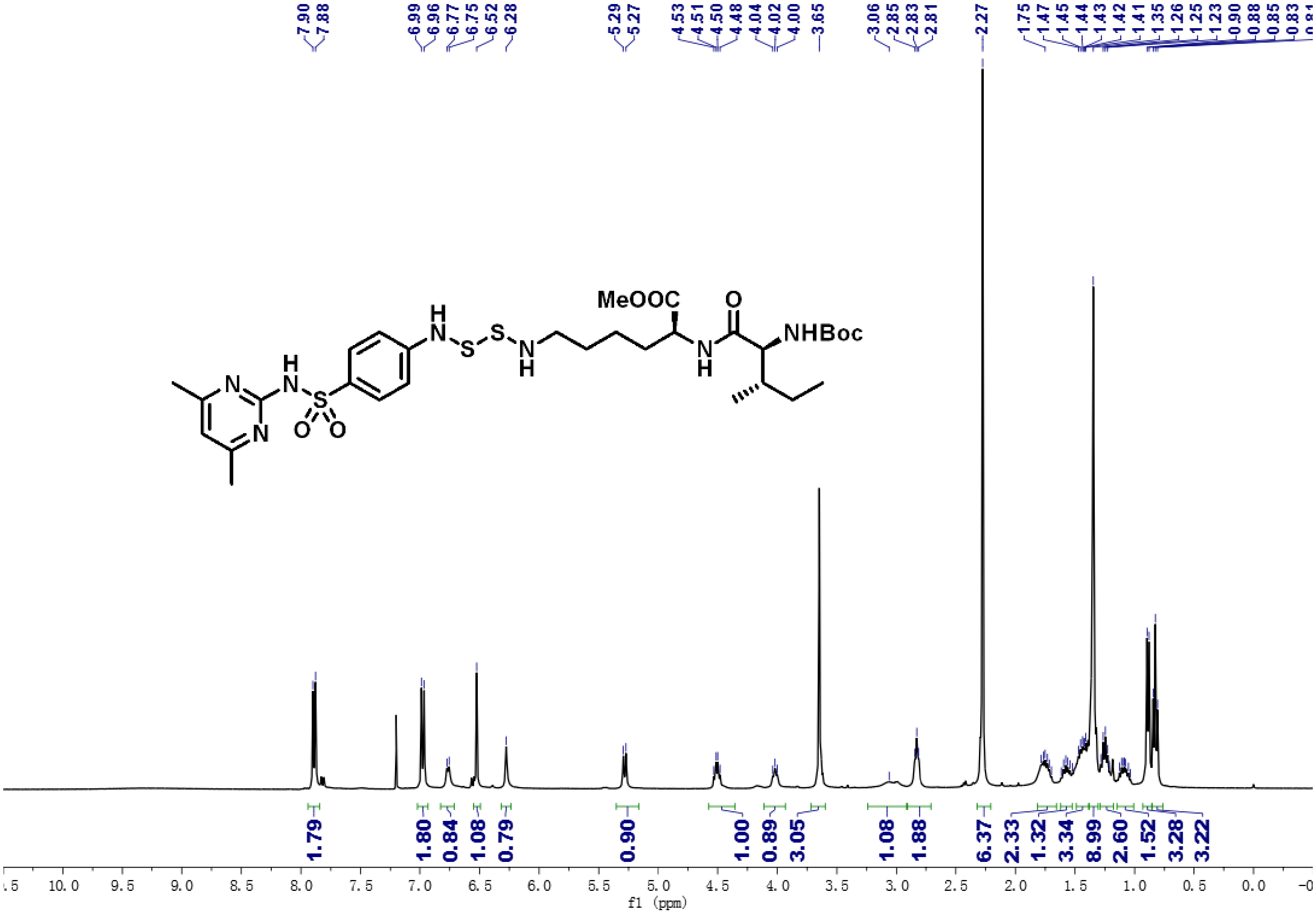

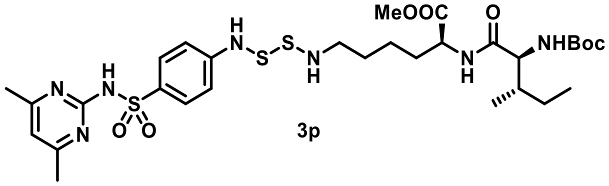

3p

^{13}C NMR ($CDCl_3$)

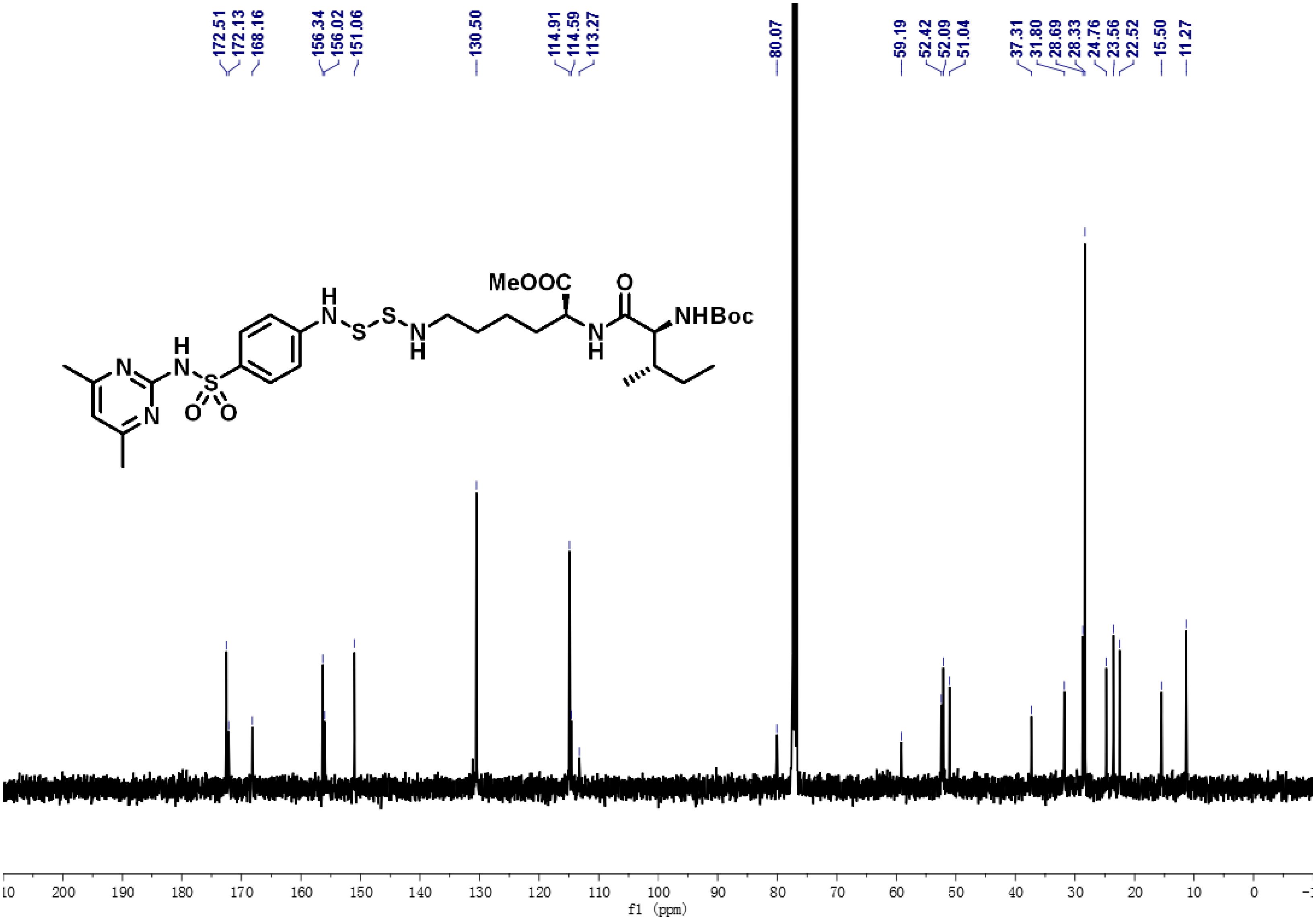

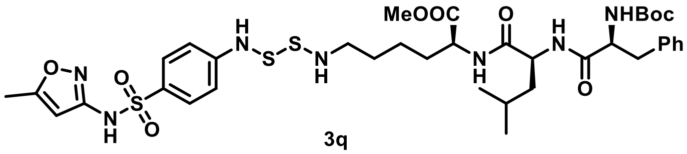

3q

^{1}H NMR ($CDCl_3$)

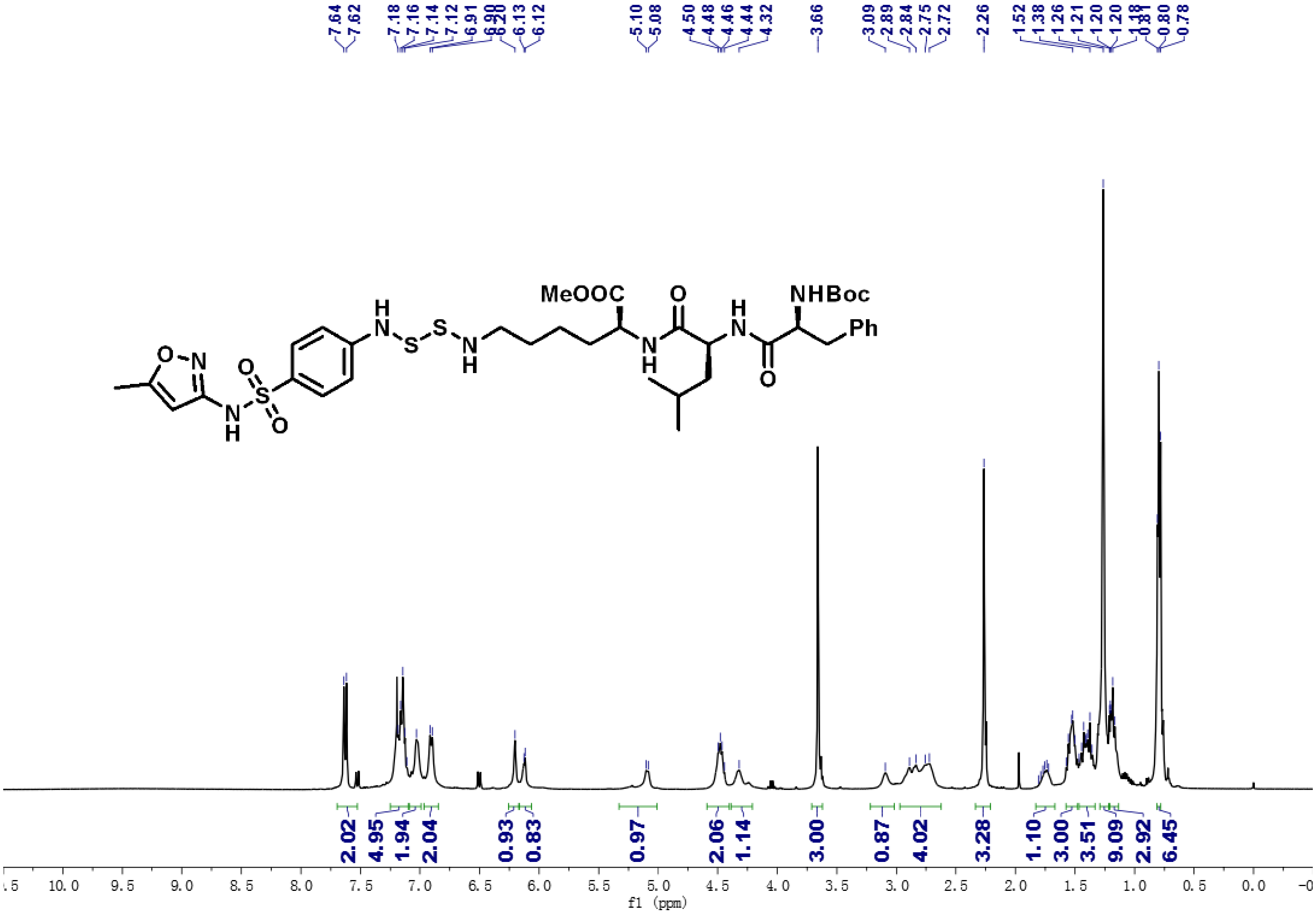

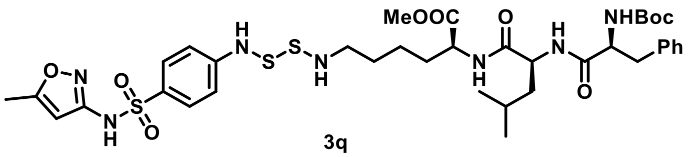

3q

^{13}C NMR ($CDCl_3$)

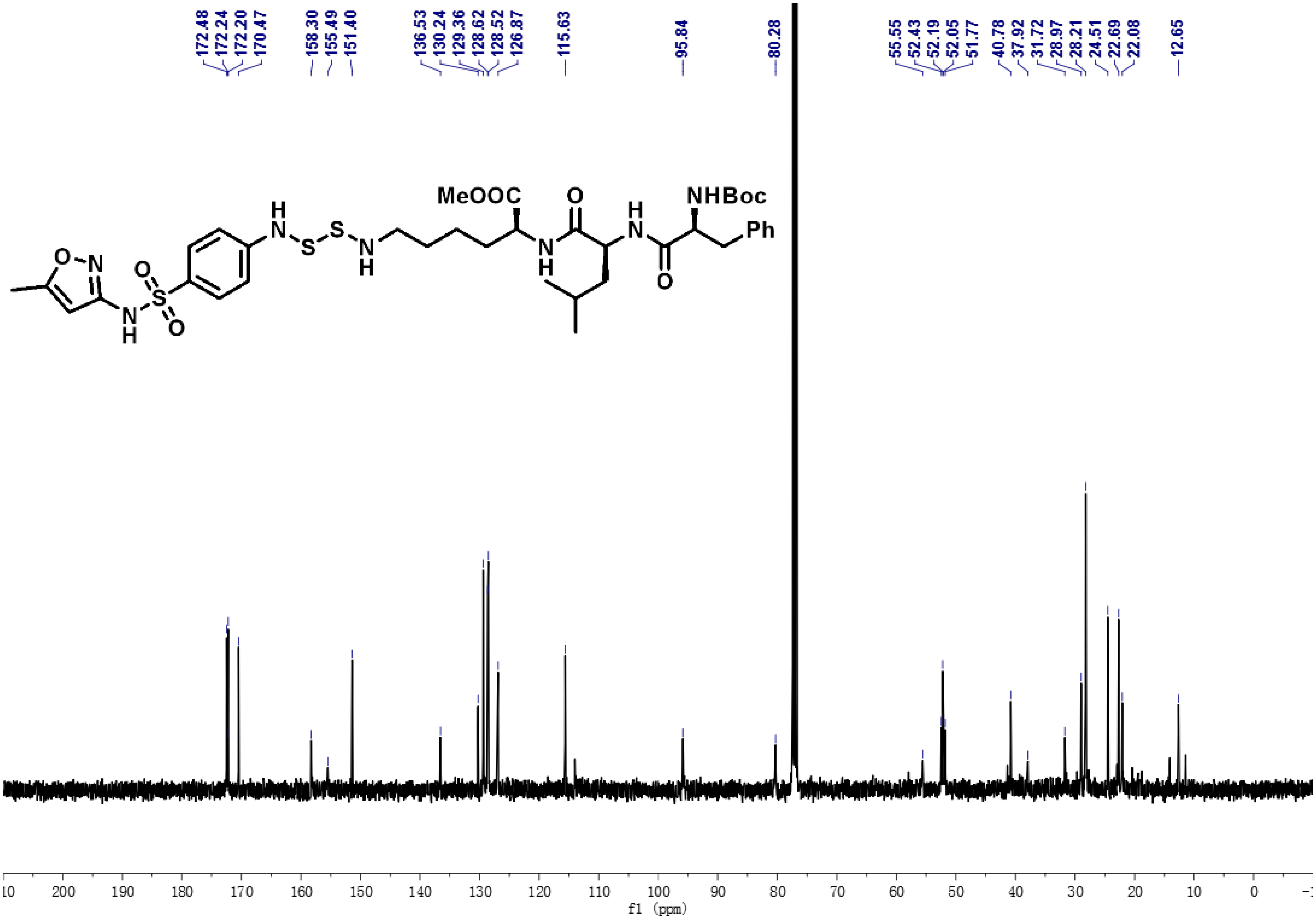

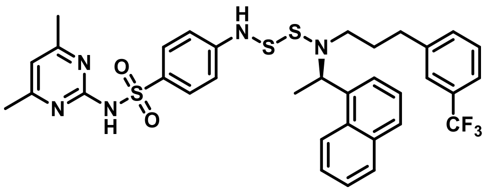

3r

^{1}H NMR ($CDCl_3$)

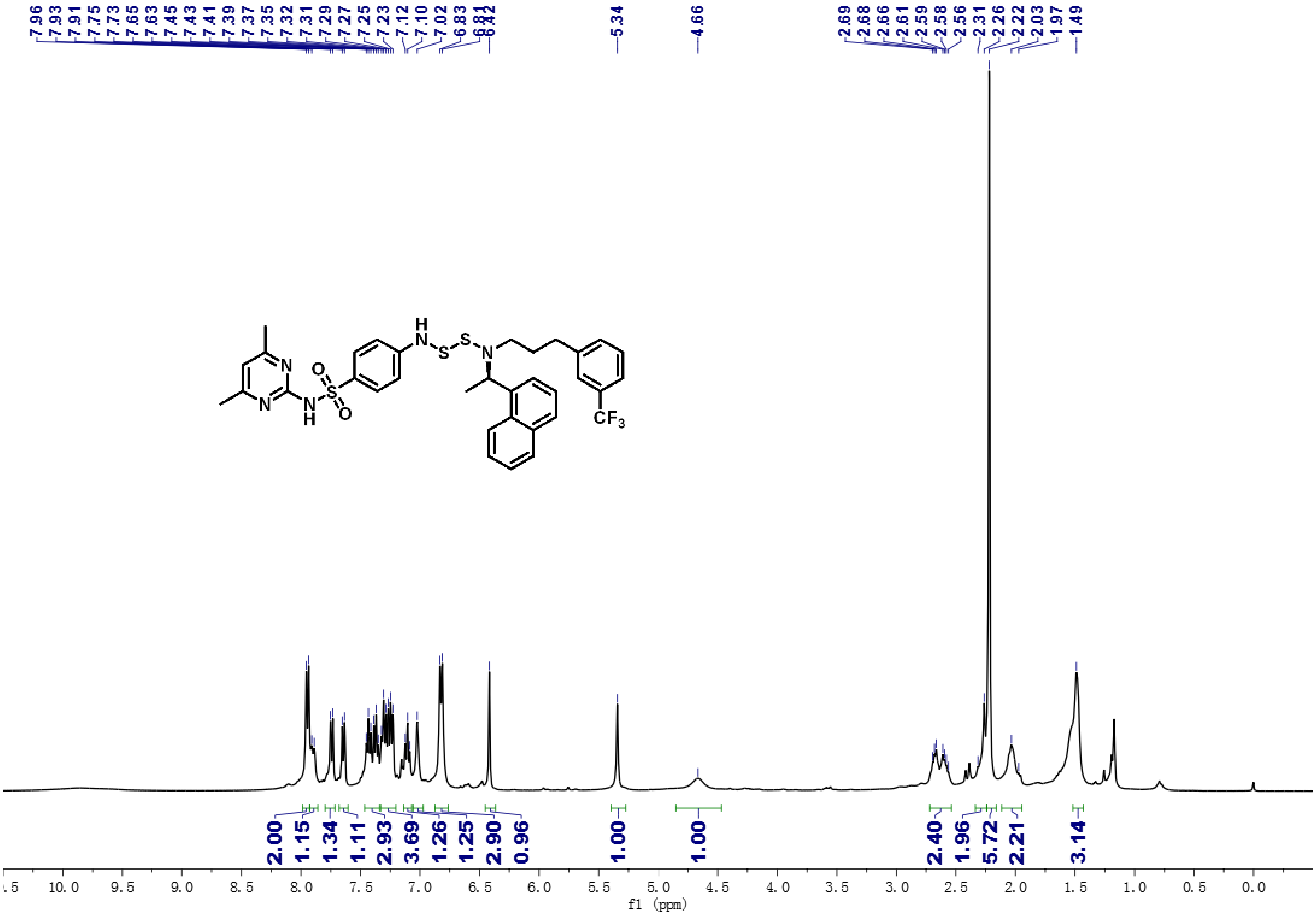

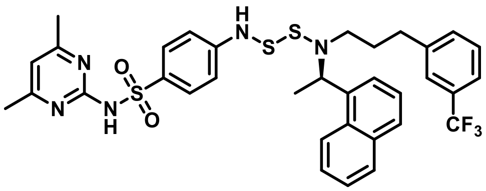

3r

^{19}F NMR ($CDCl_3$)

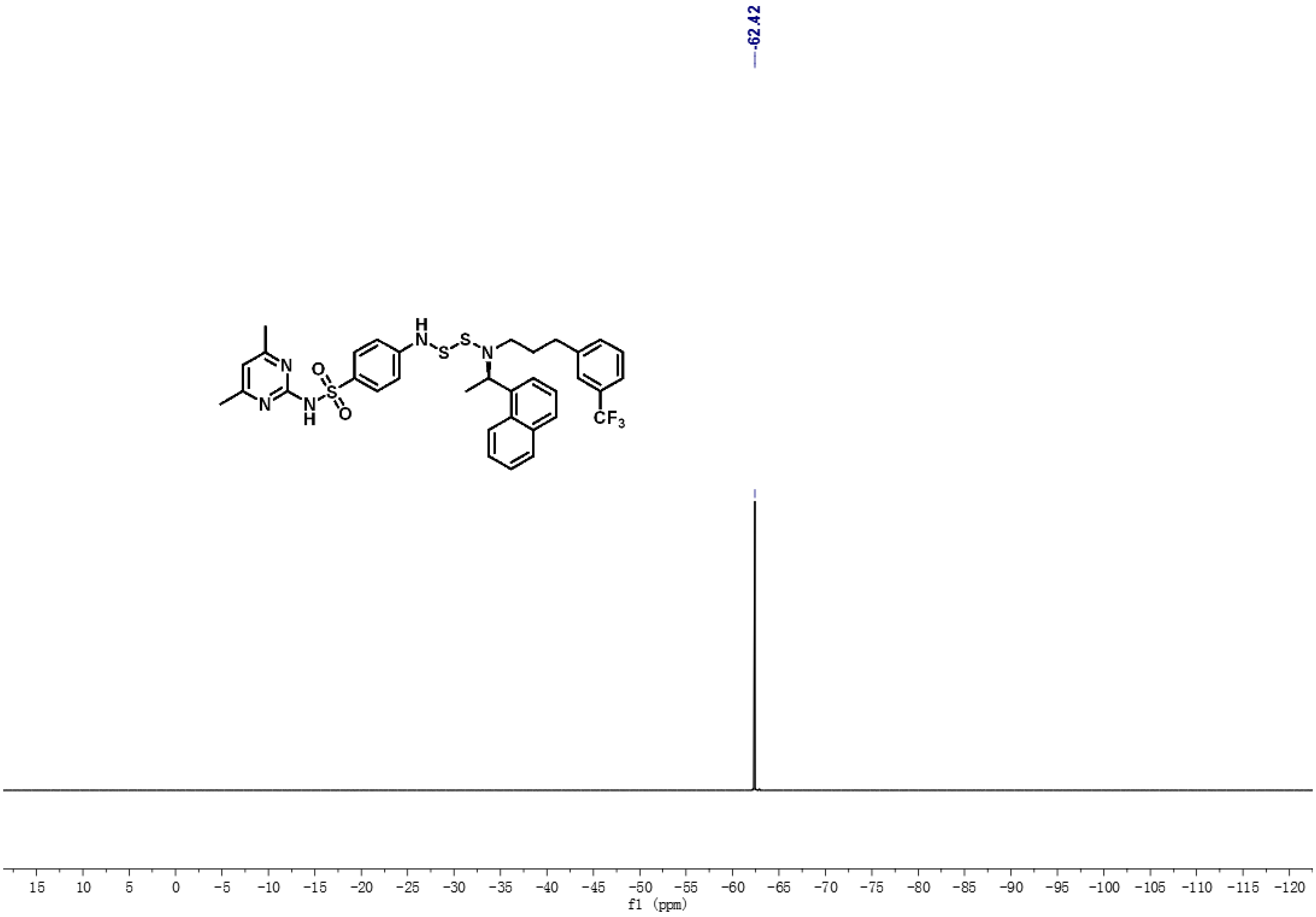

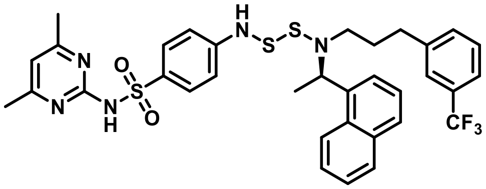

3r

^{13}C NMR ($CDCl_3$)

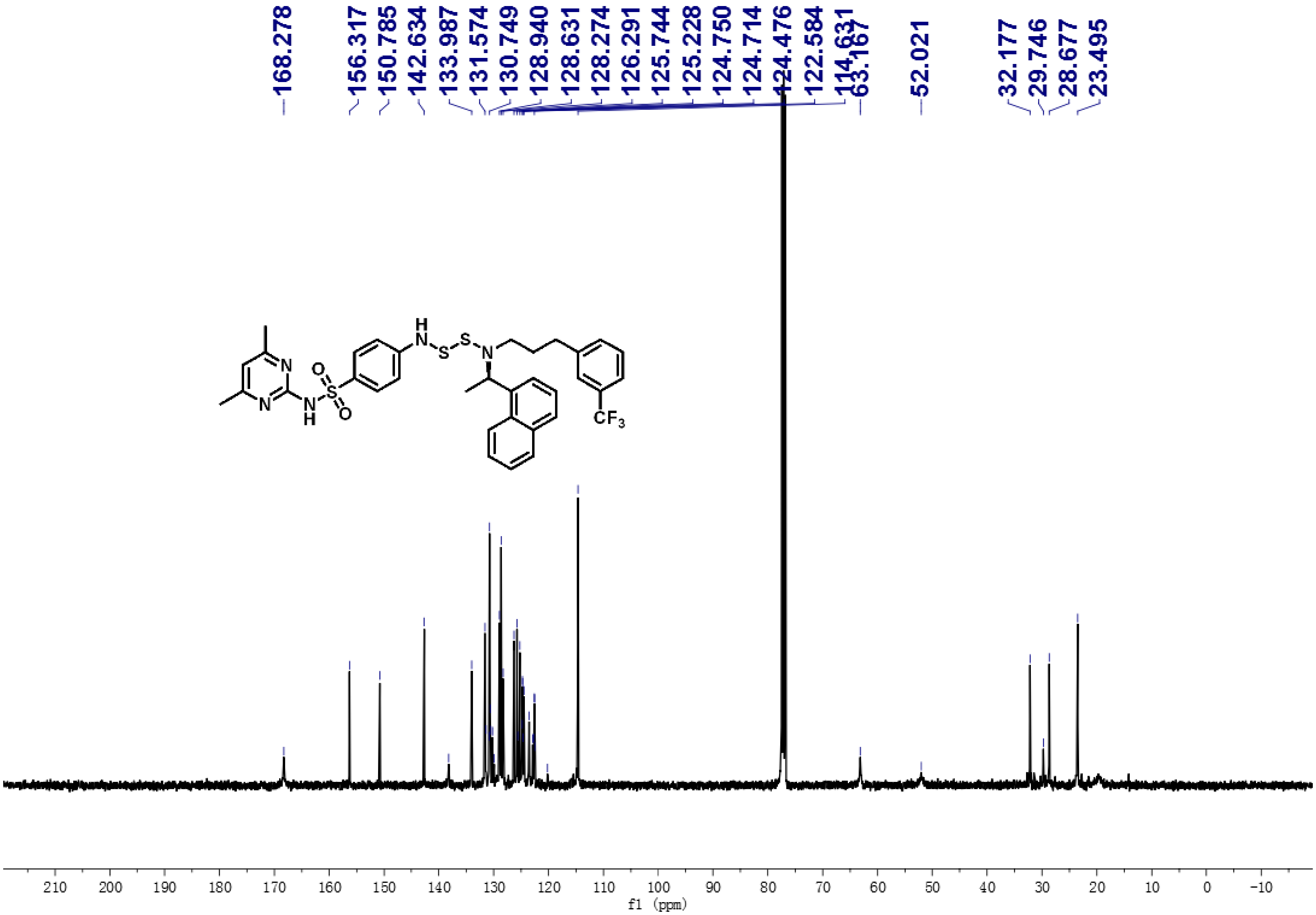

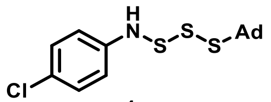

4a

^{1}H NMR (CD_3CN)

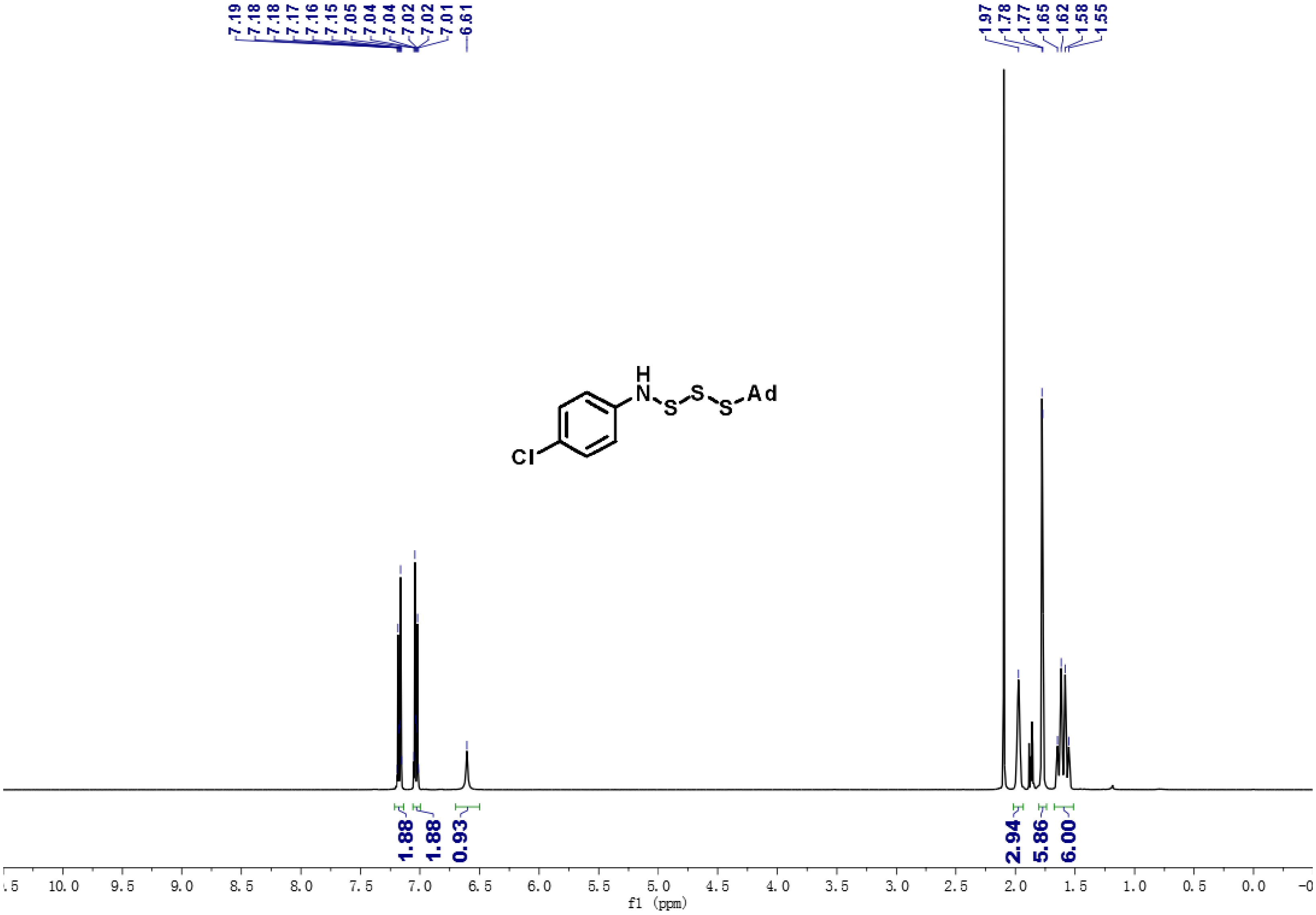

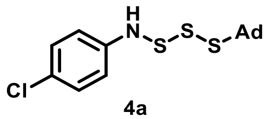

4a

^{1}H NMR (CD_3CN), ^{13}C NMR (CD_3CN)

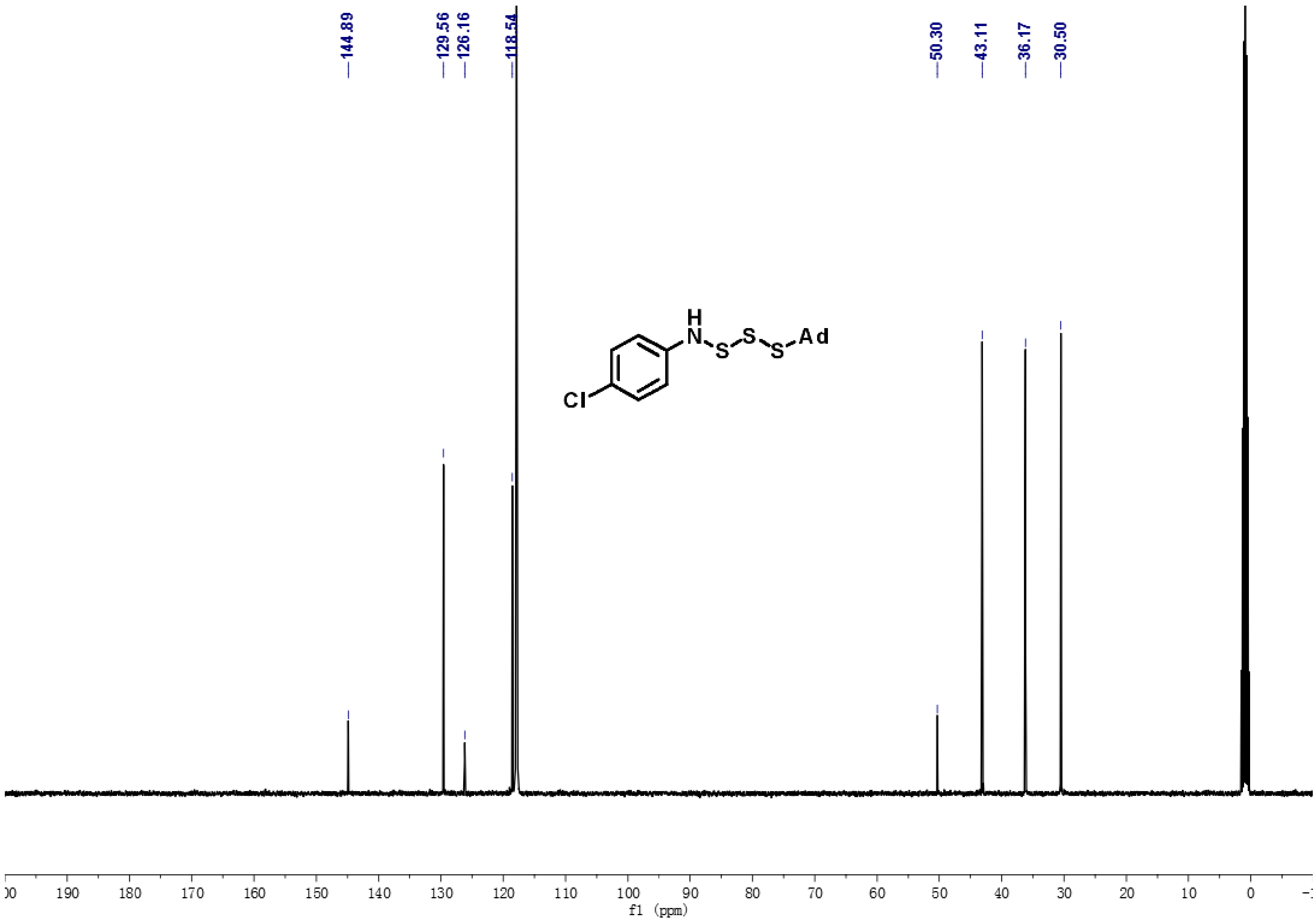

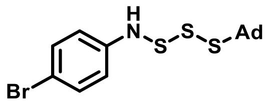

^{1}H NMR (Acetone-d_6)

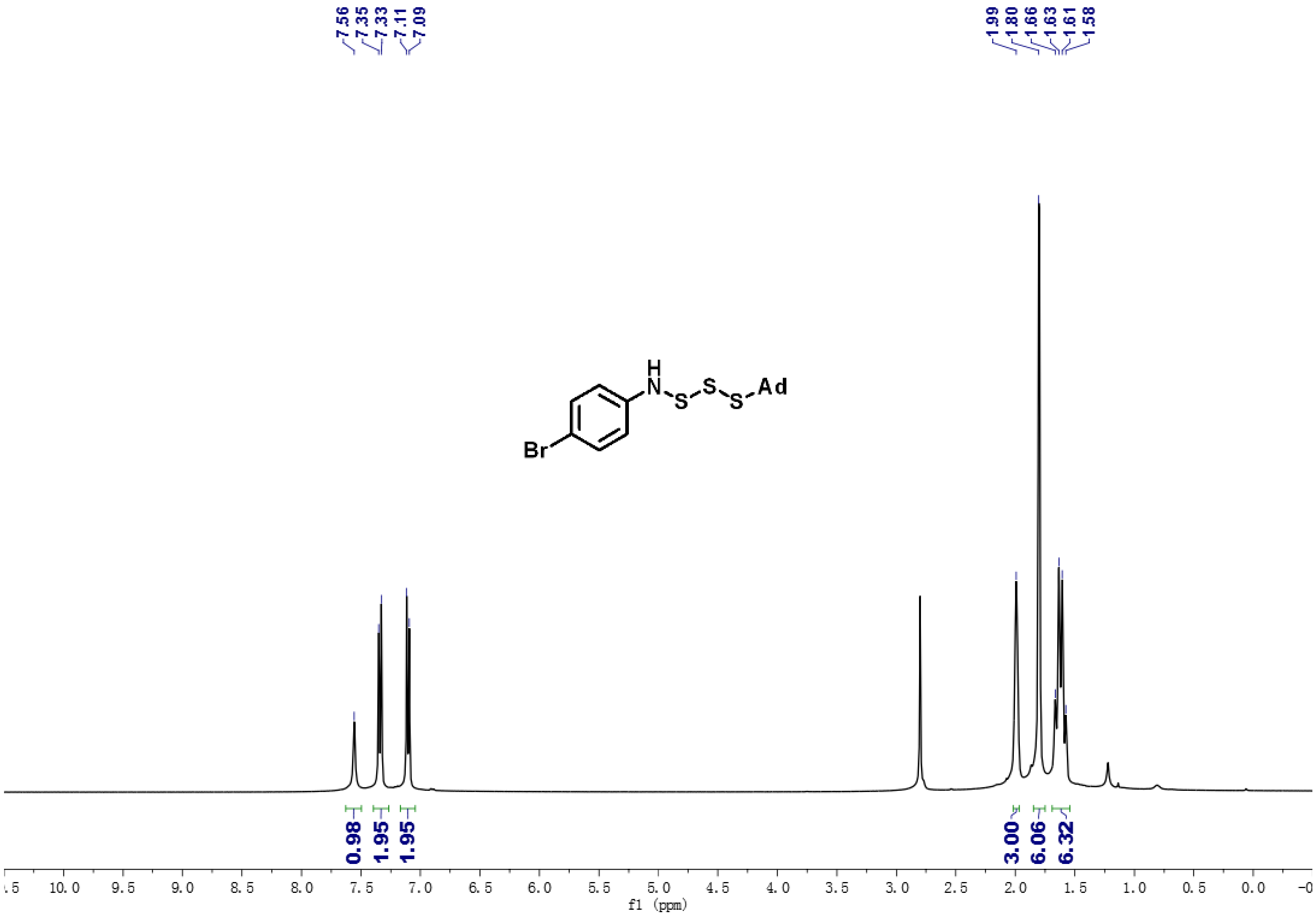

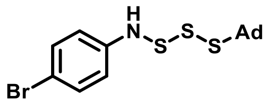

^{13}C NMR (Acetone-d$_6$)

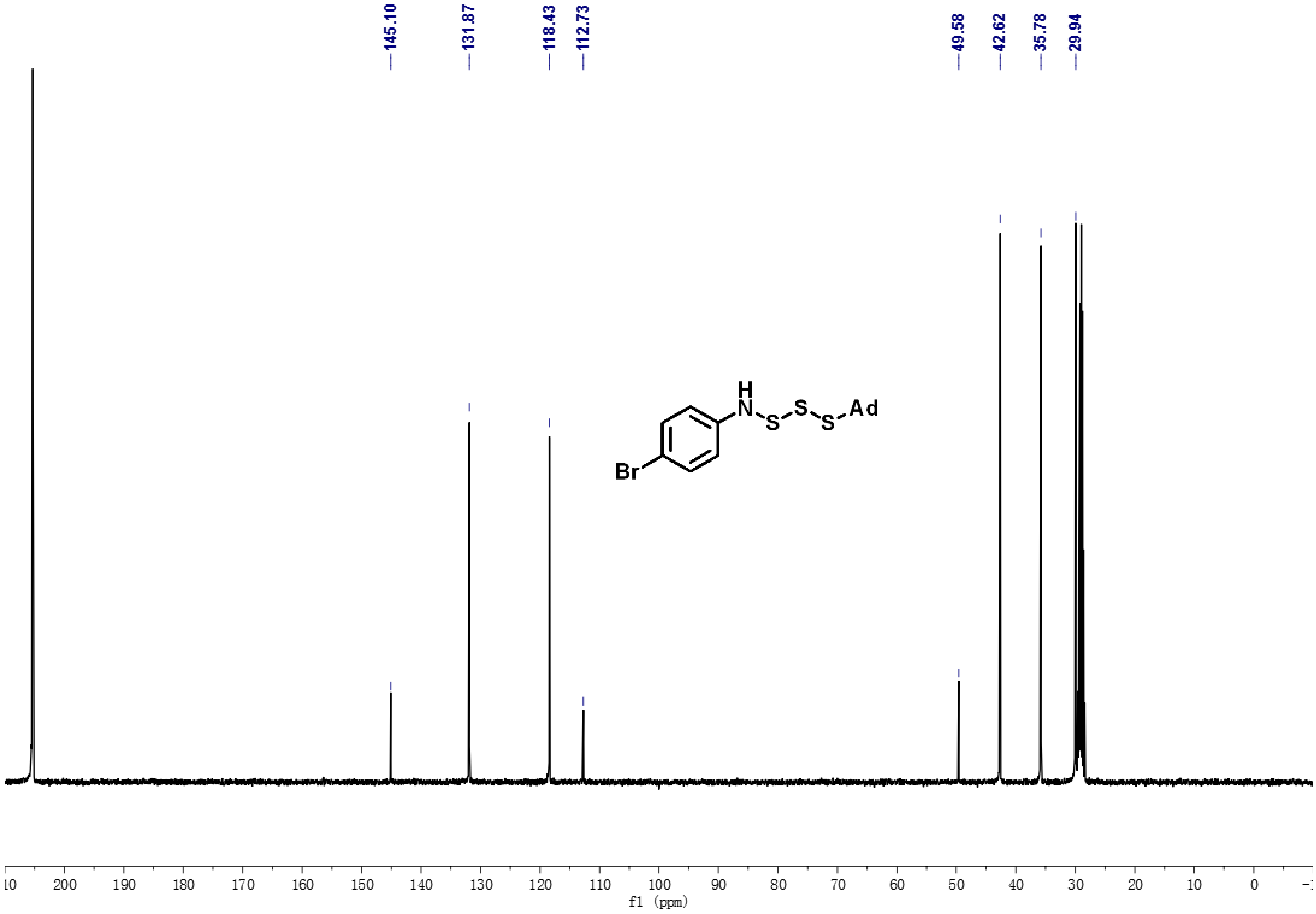

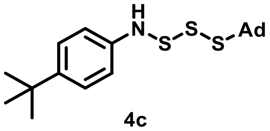

4c

^{1}H NMR (Acetone-d$_6$)

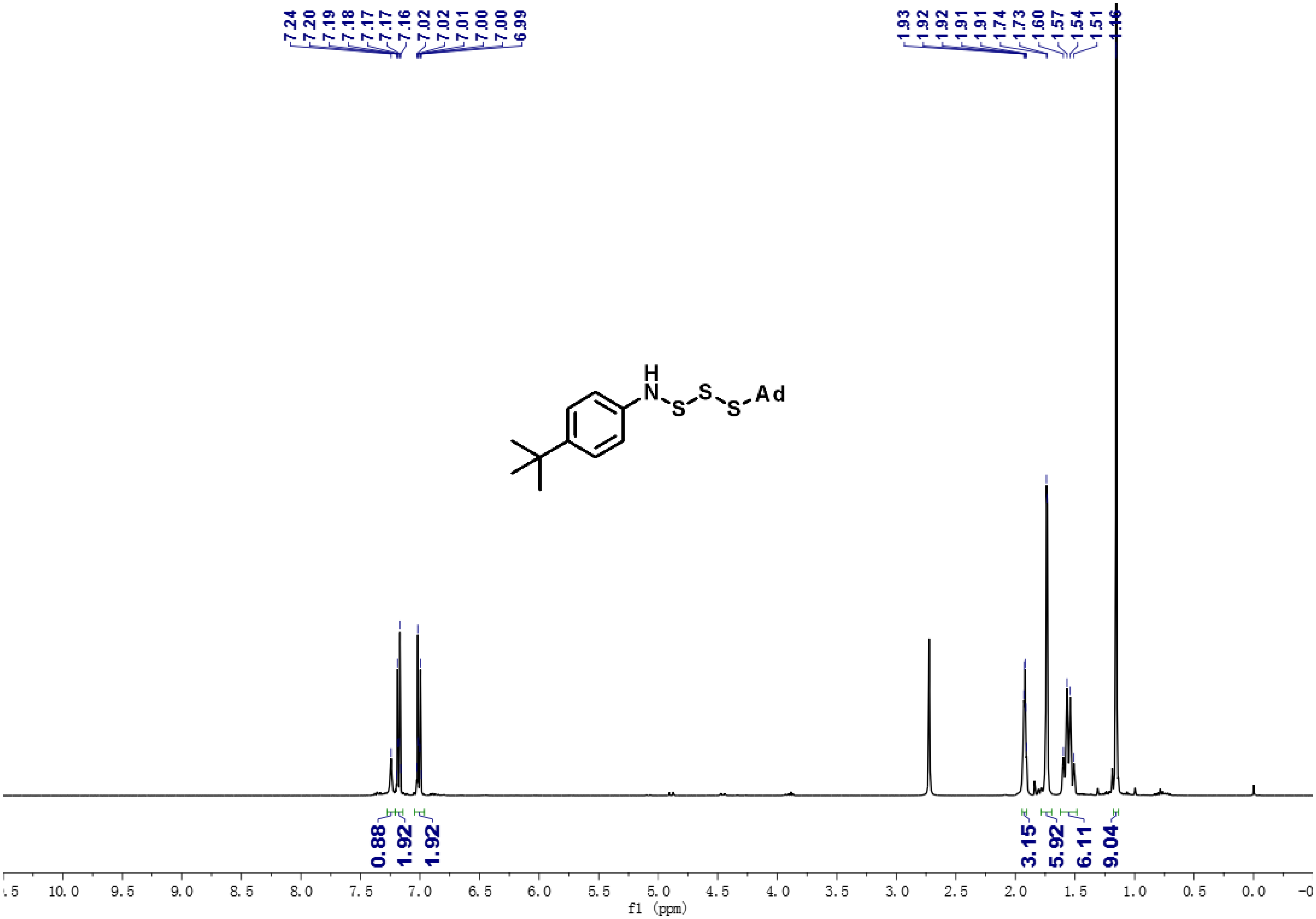

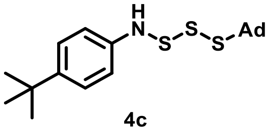

4c

^{13}C NMR (Acetone-d_6)

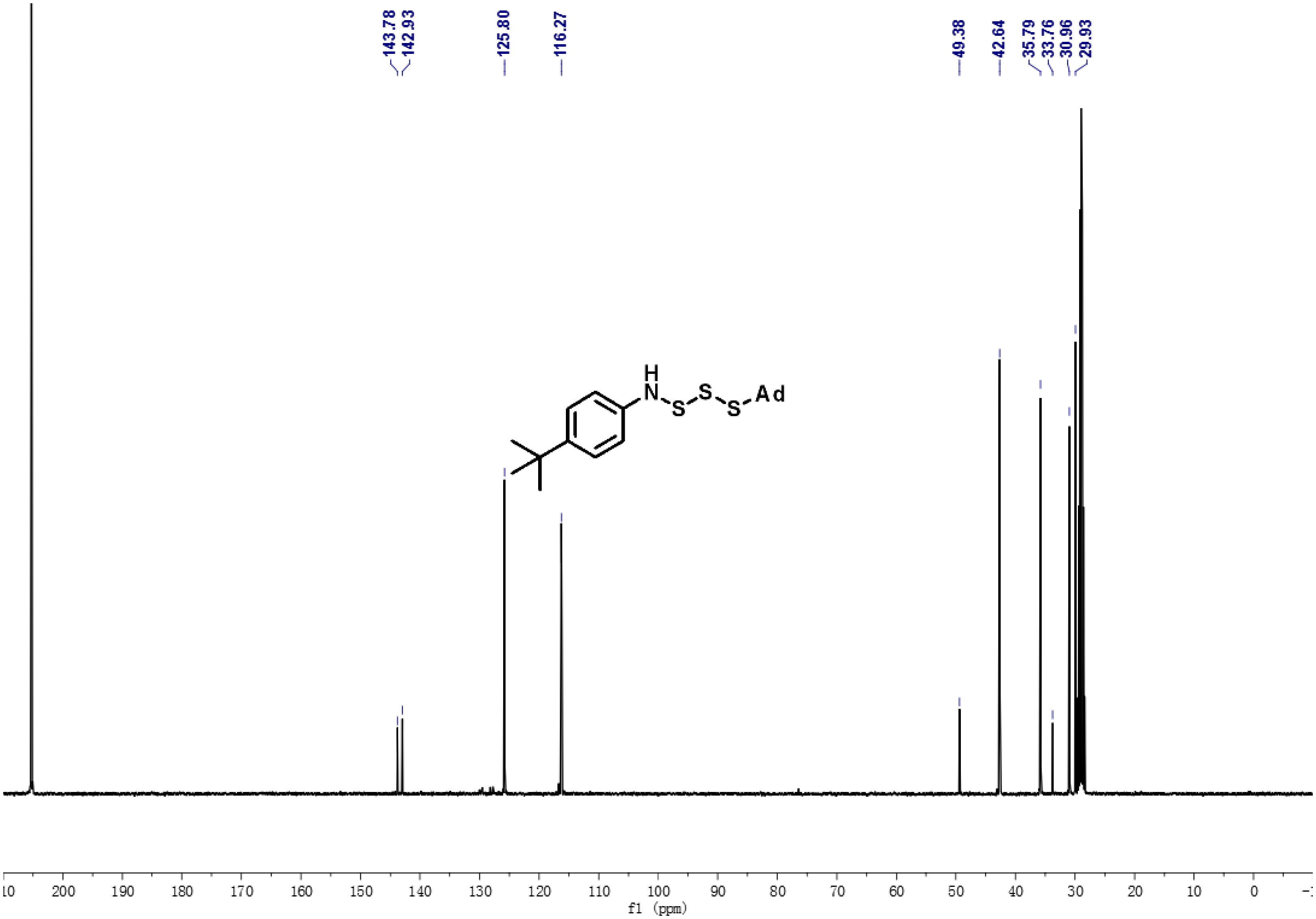

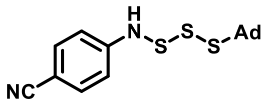

4d

^{1}H NMR (Acetone-d$_6$)

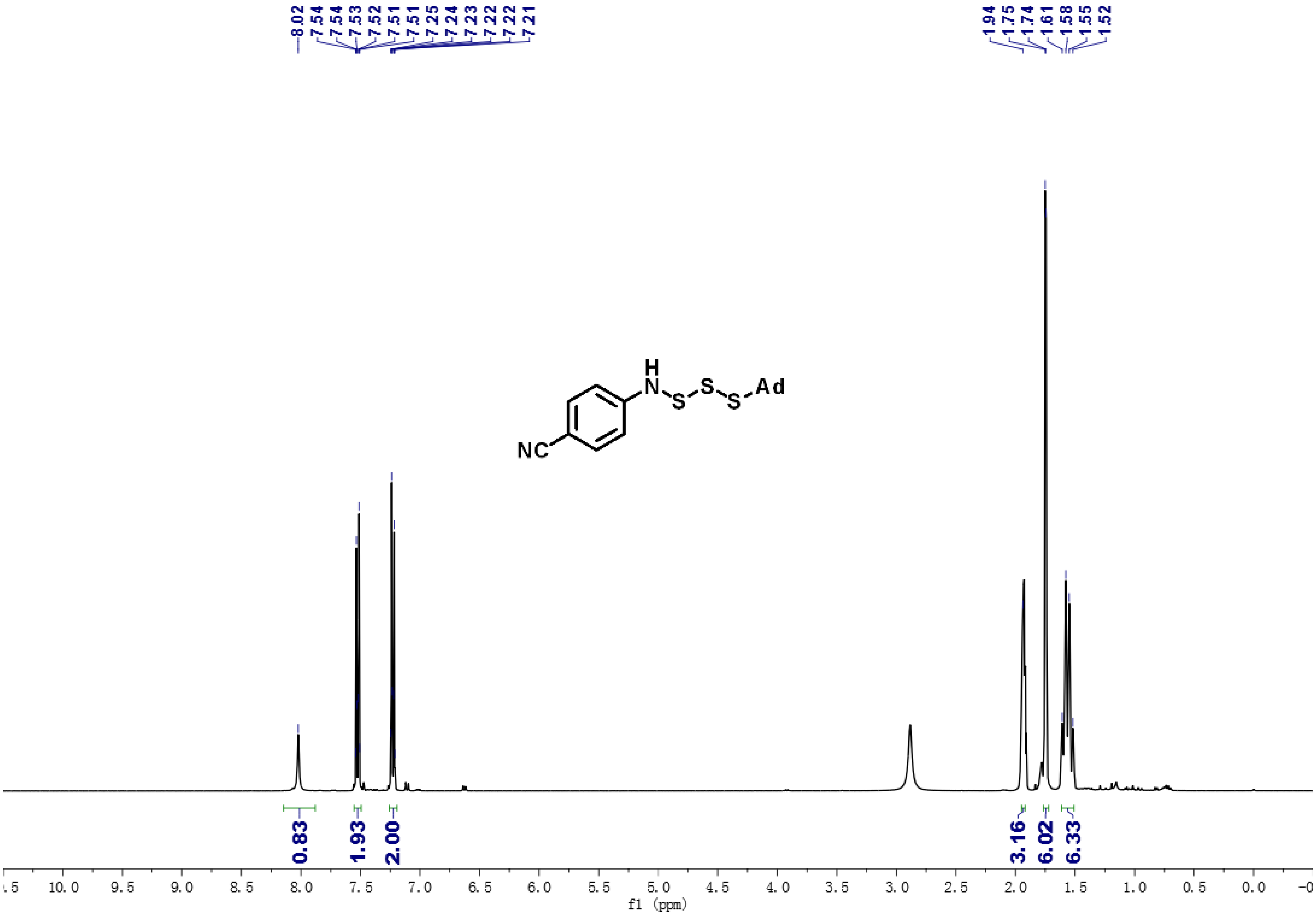

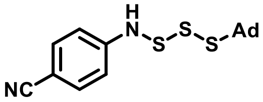

4d

^{13}C NMR (Acetone-d_6)

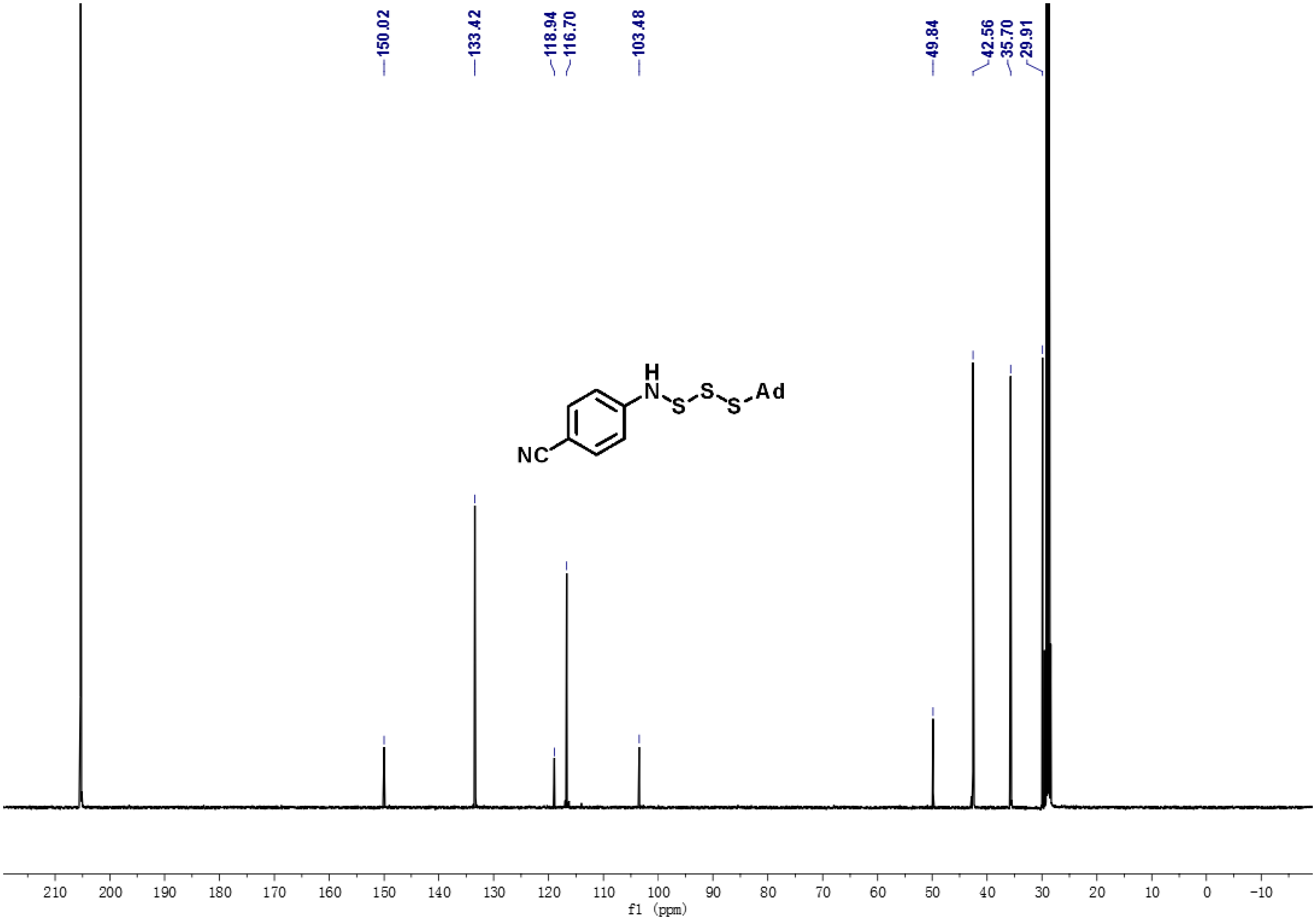

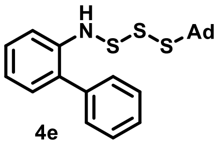

4e

^{1}H NMR (Acetone-d_6)

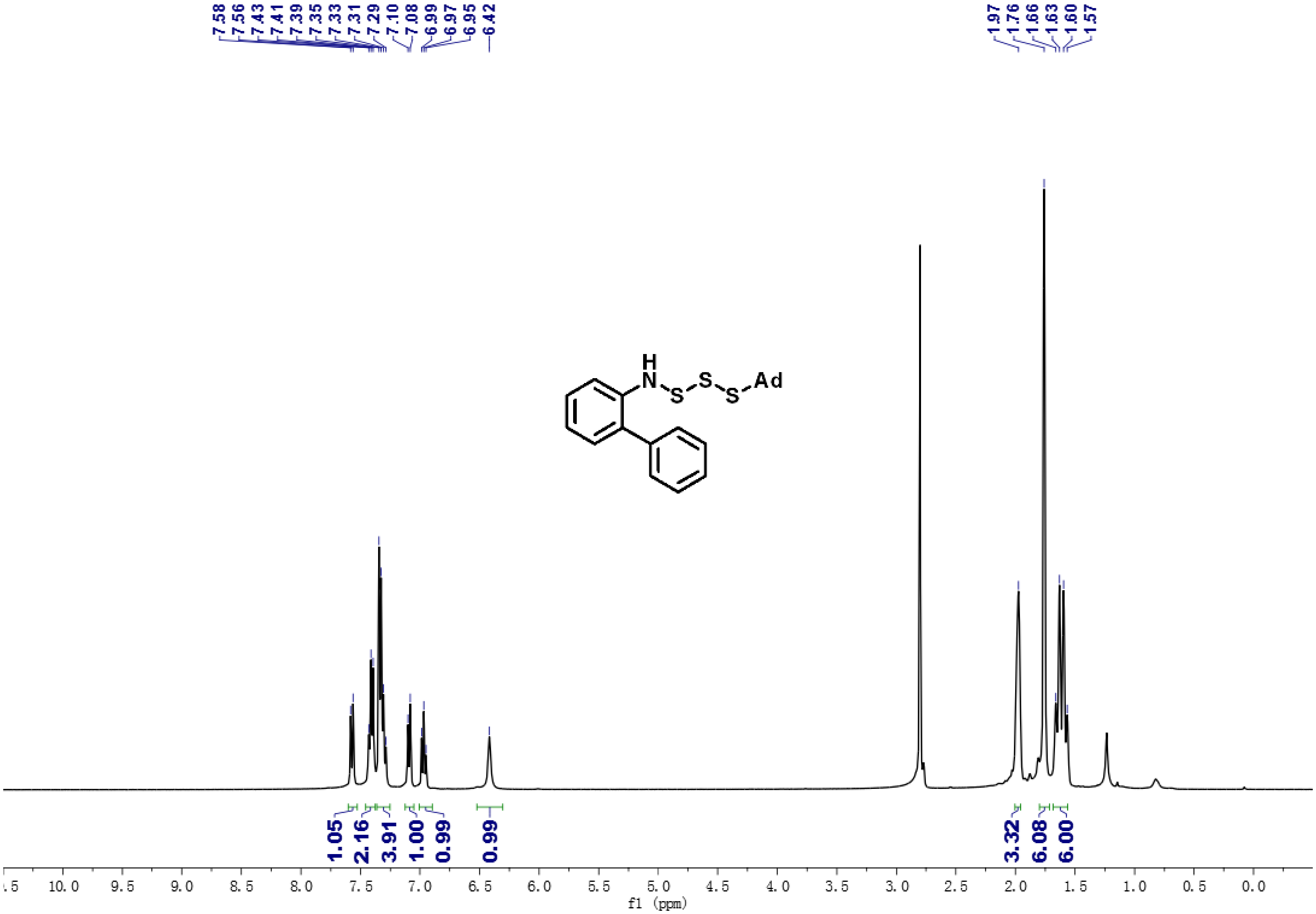

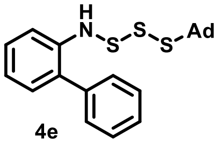

4e

^{13}C NMR (Acetone-d$_6$)

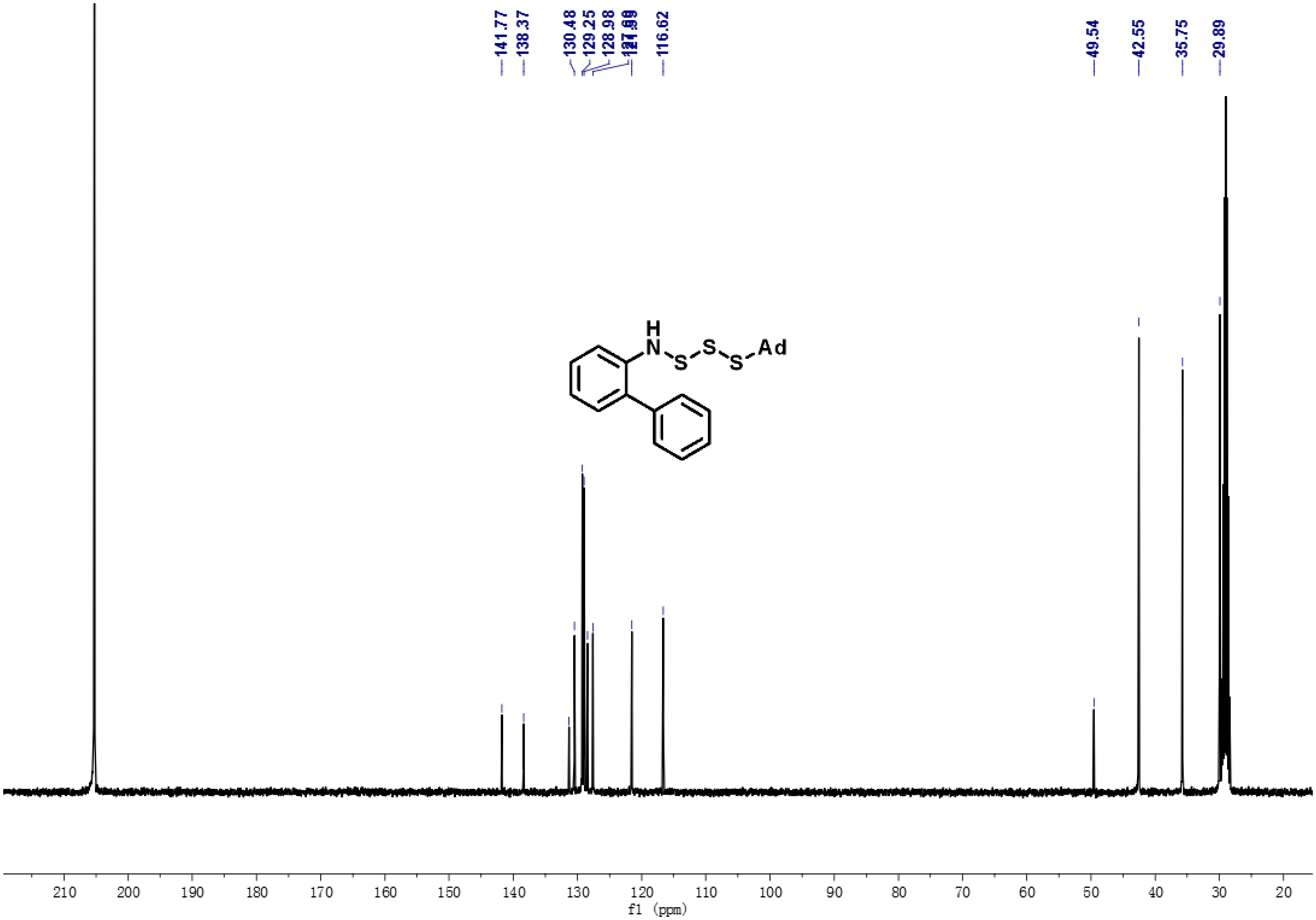

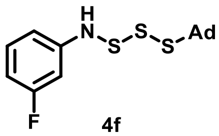

4f

^{1}H NMR (Acetone-d_6)

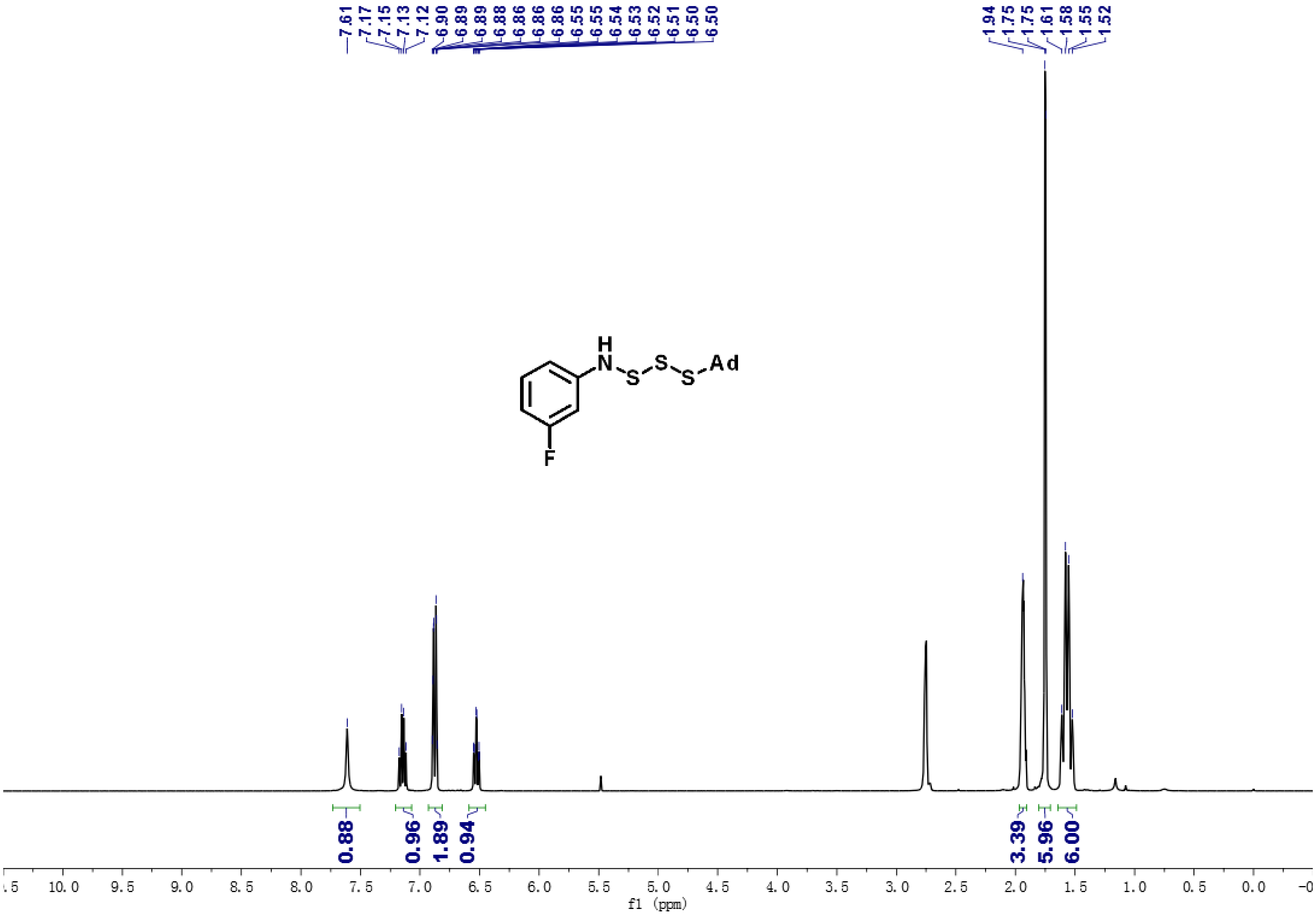

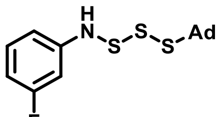

4f

^{19}F NMR (Acetone-d$_6$)

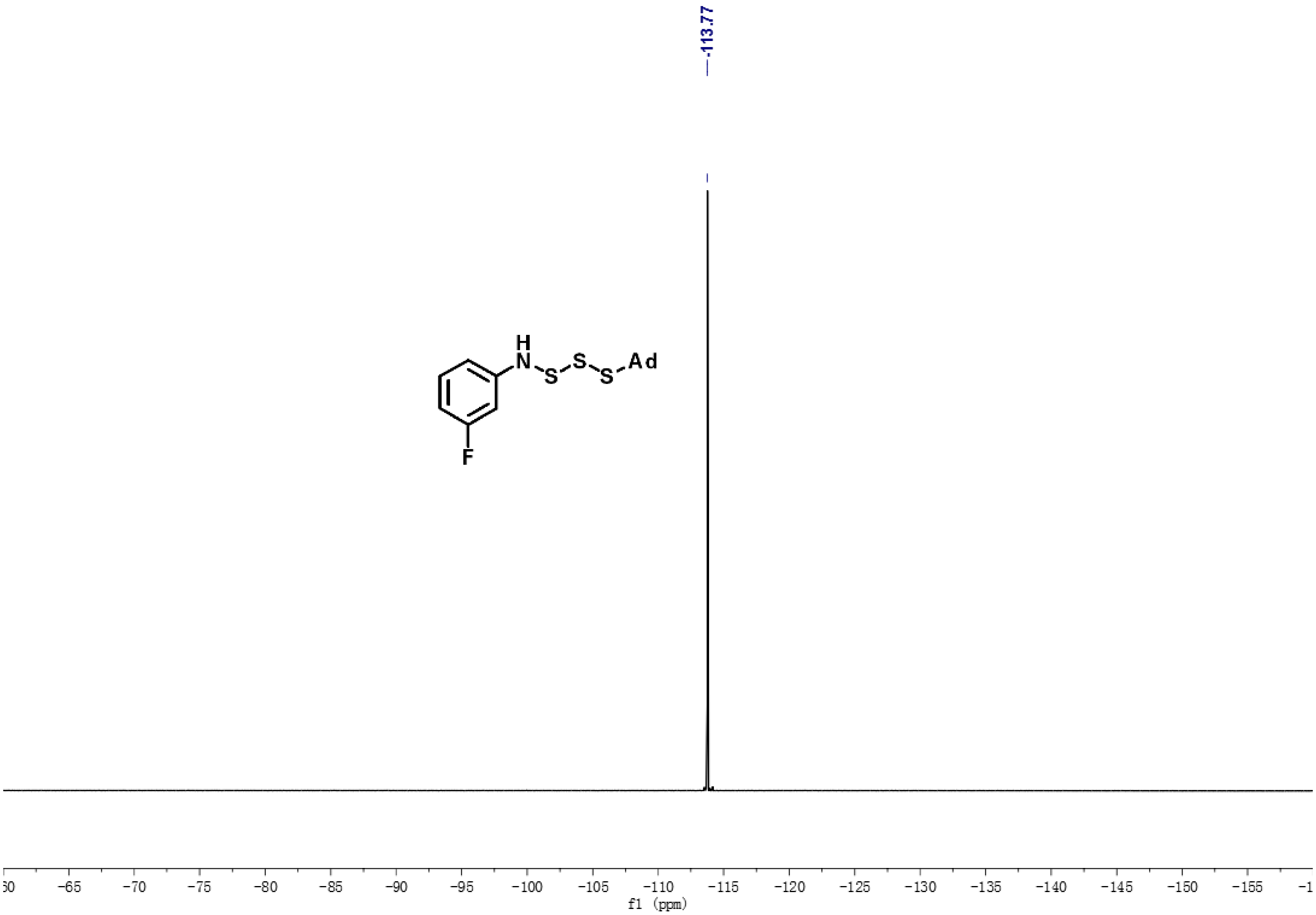

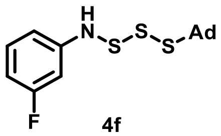

4f

^{13}C NMR (Acetone-d6)

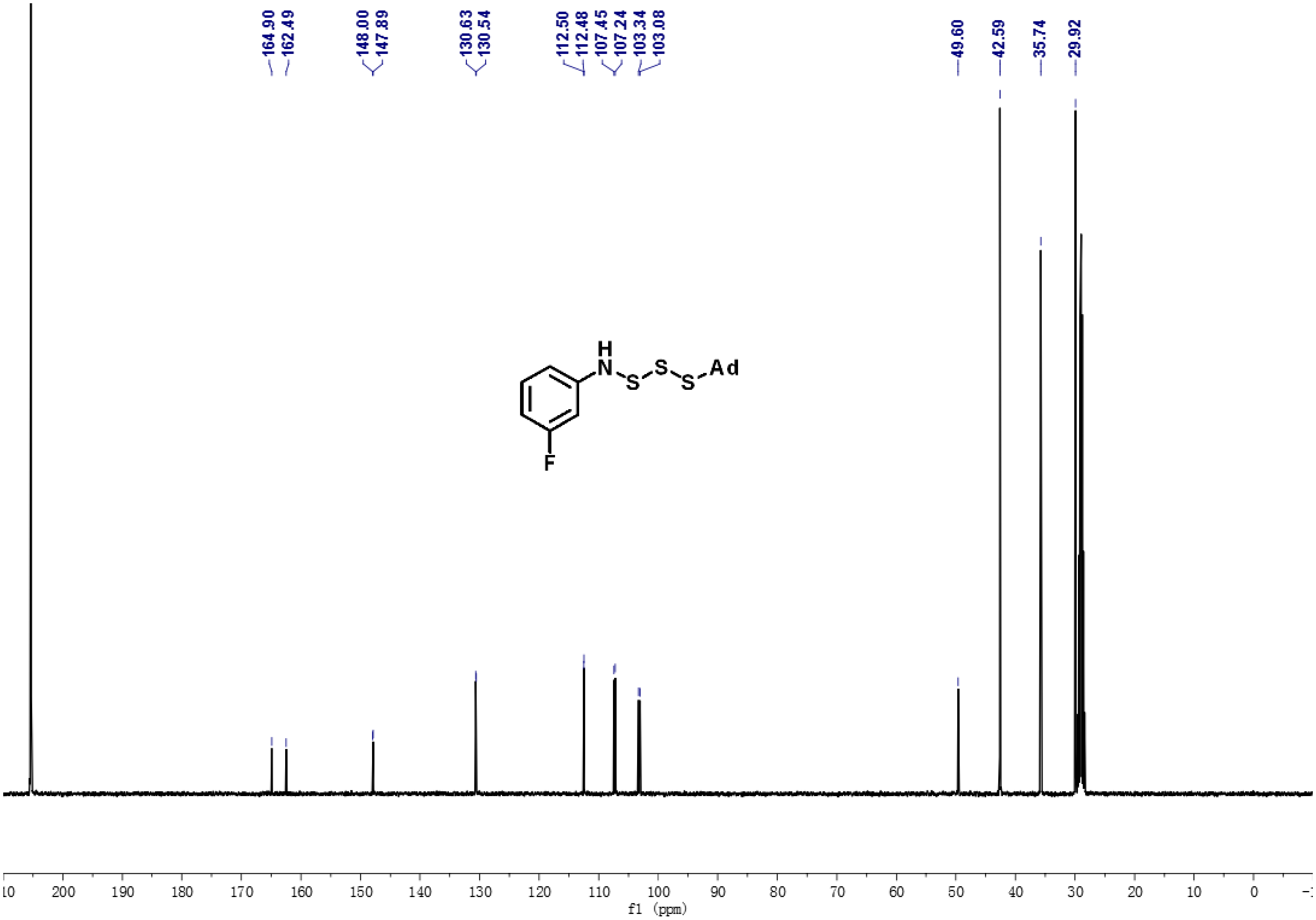

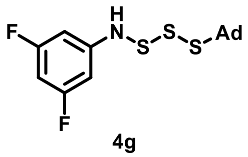

4g

^{1}H NMR (Acetone-d$_6$)

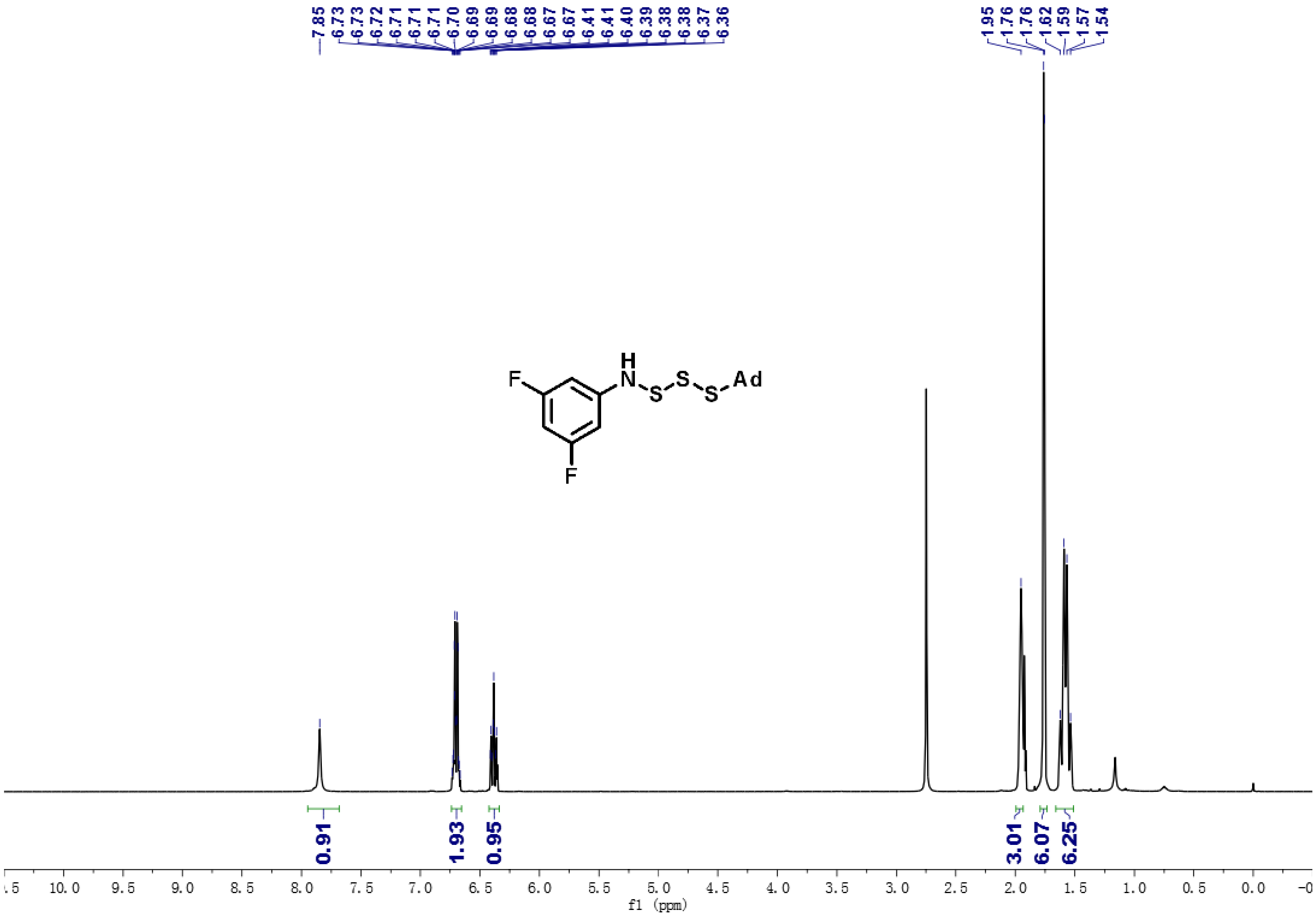

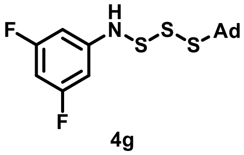

4g

^{19}F NMR (Acetone-d$_6$)

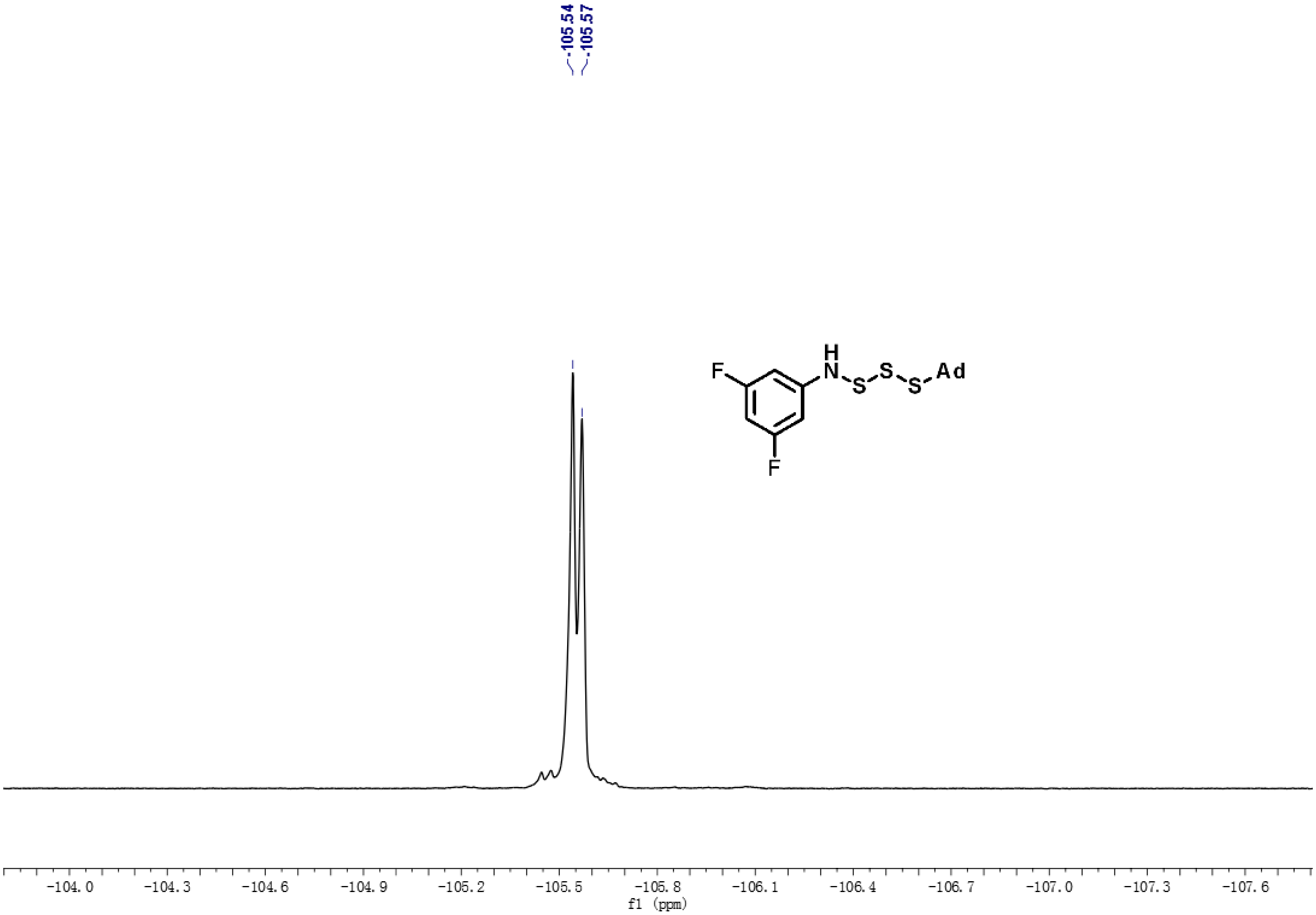

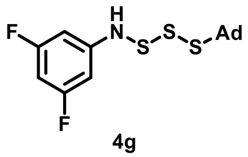

4g

^{13}C NMR (Acetone-d$_6$)

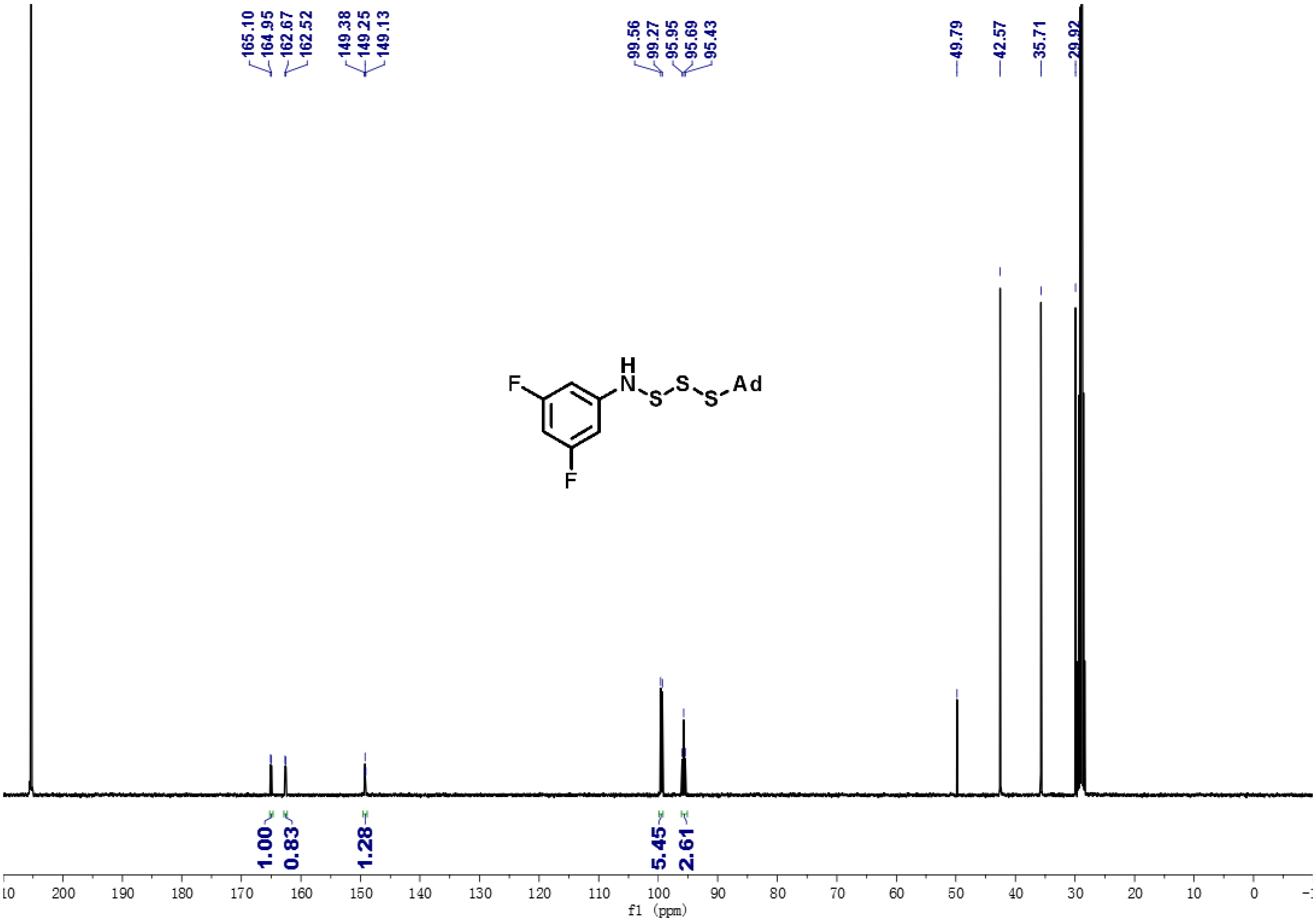

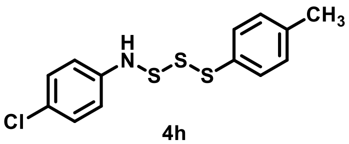

4h

^{1}H NMR (Acetone-d$_6$)

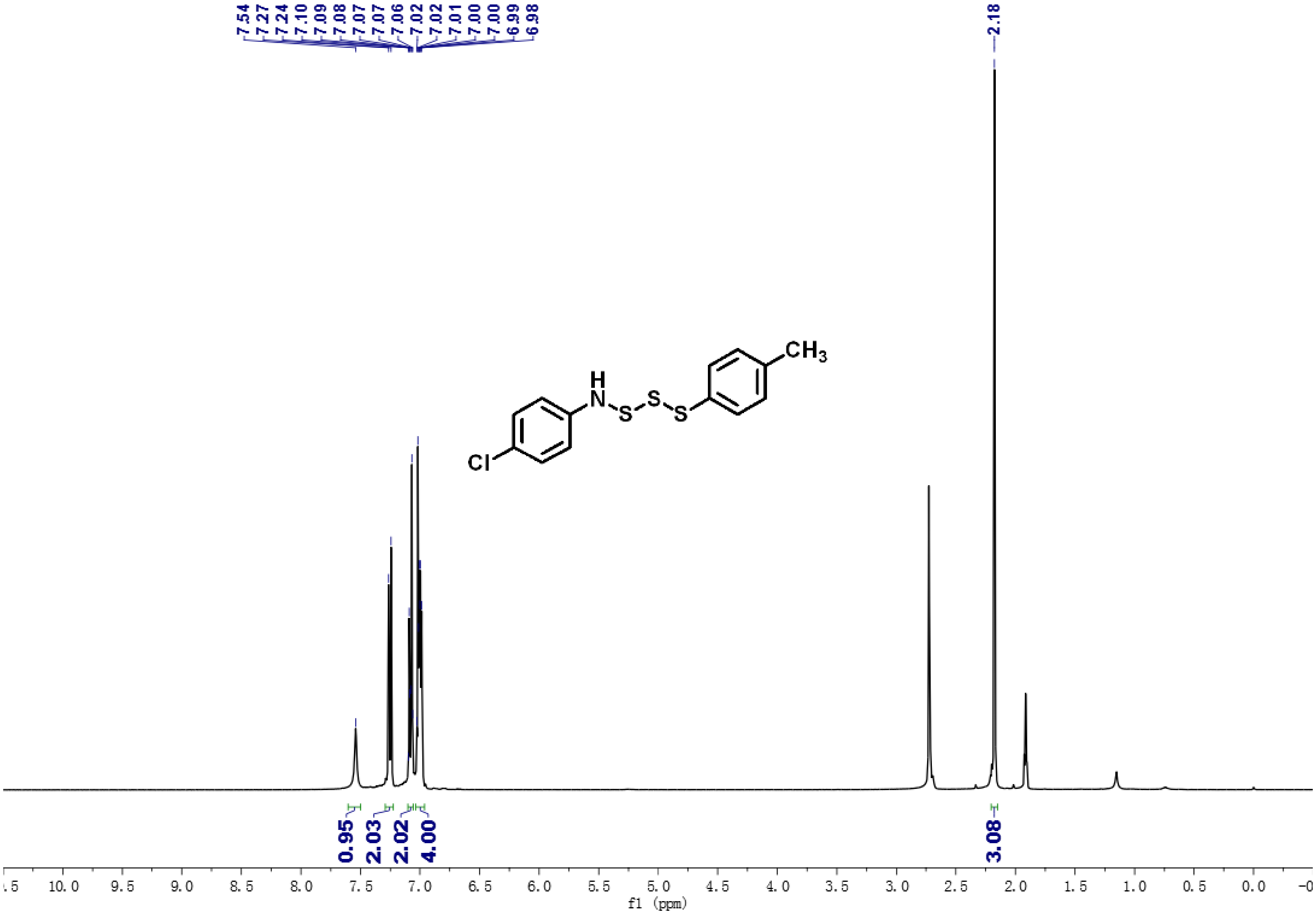

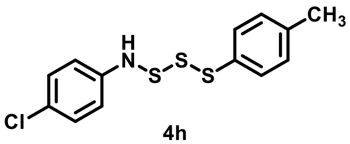

4h

^{13}C NMR (Acetone-d6)

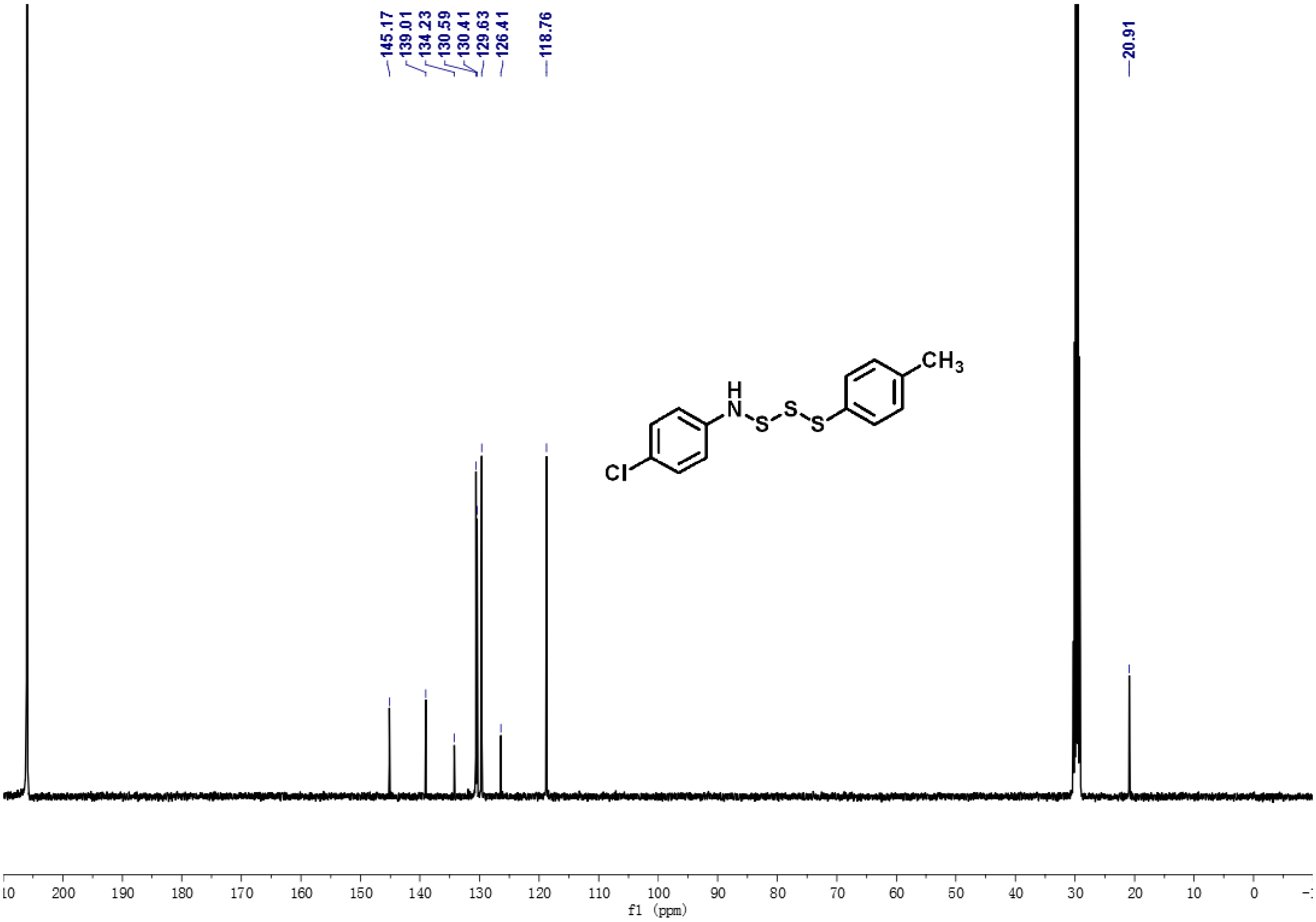

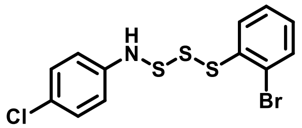

4i

^{1}H NMR (Acetone-d6)

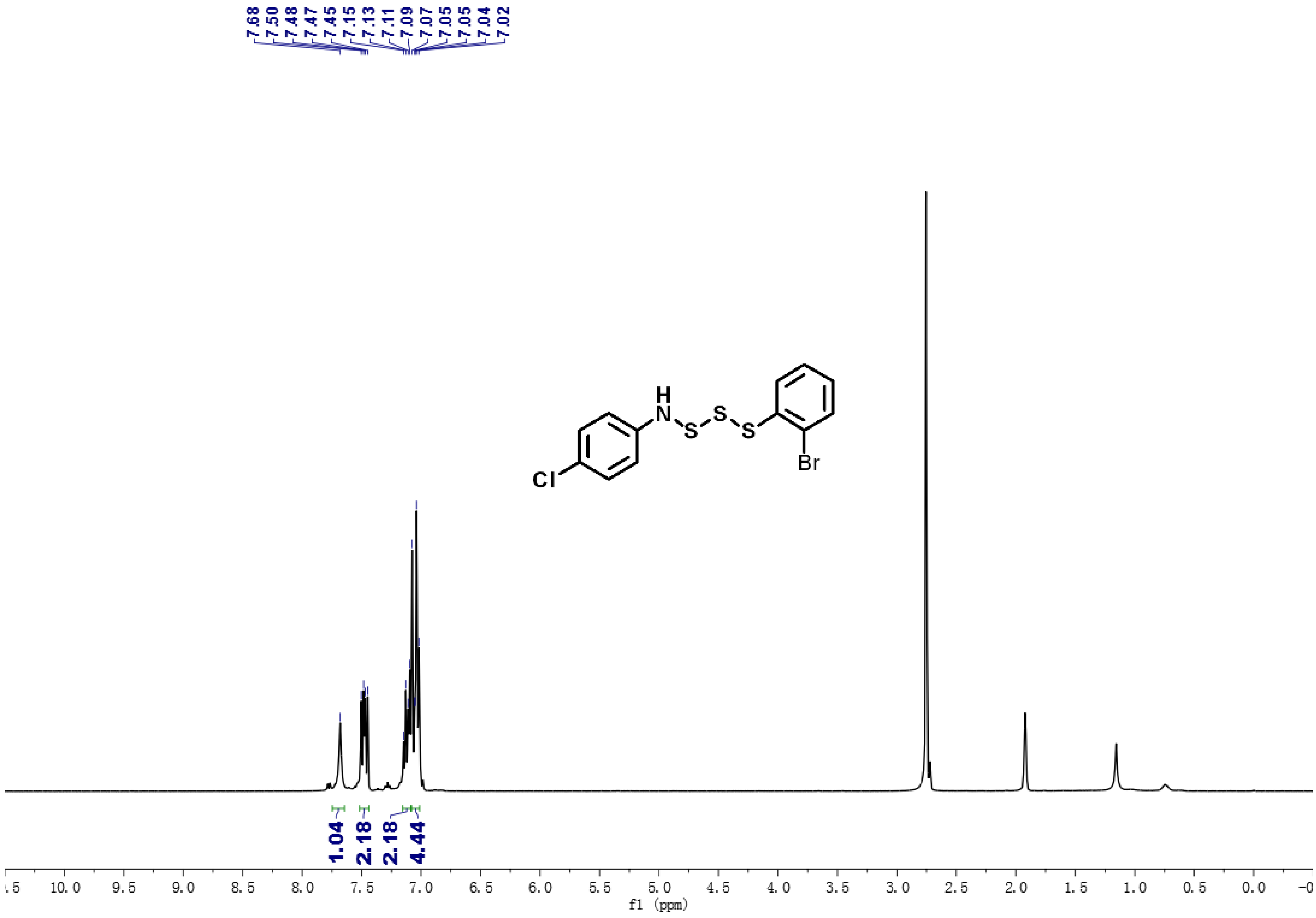

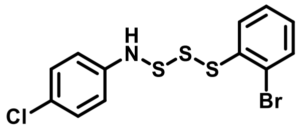

4i

^{13}C NMR (Acetone-d_6)

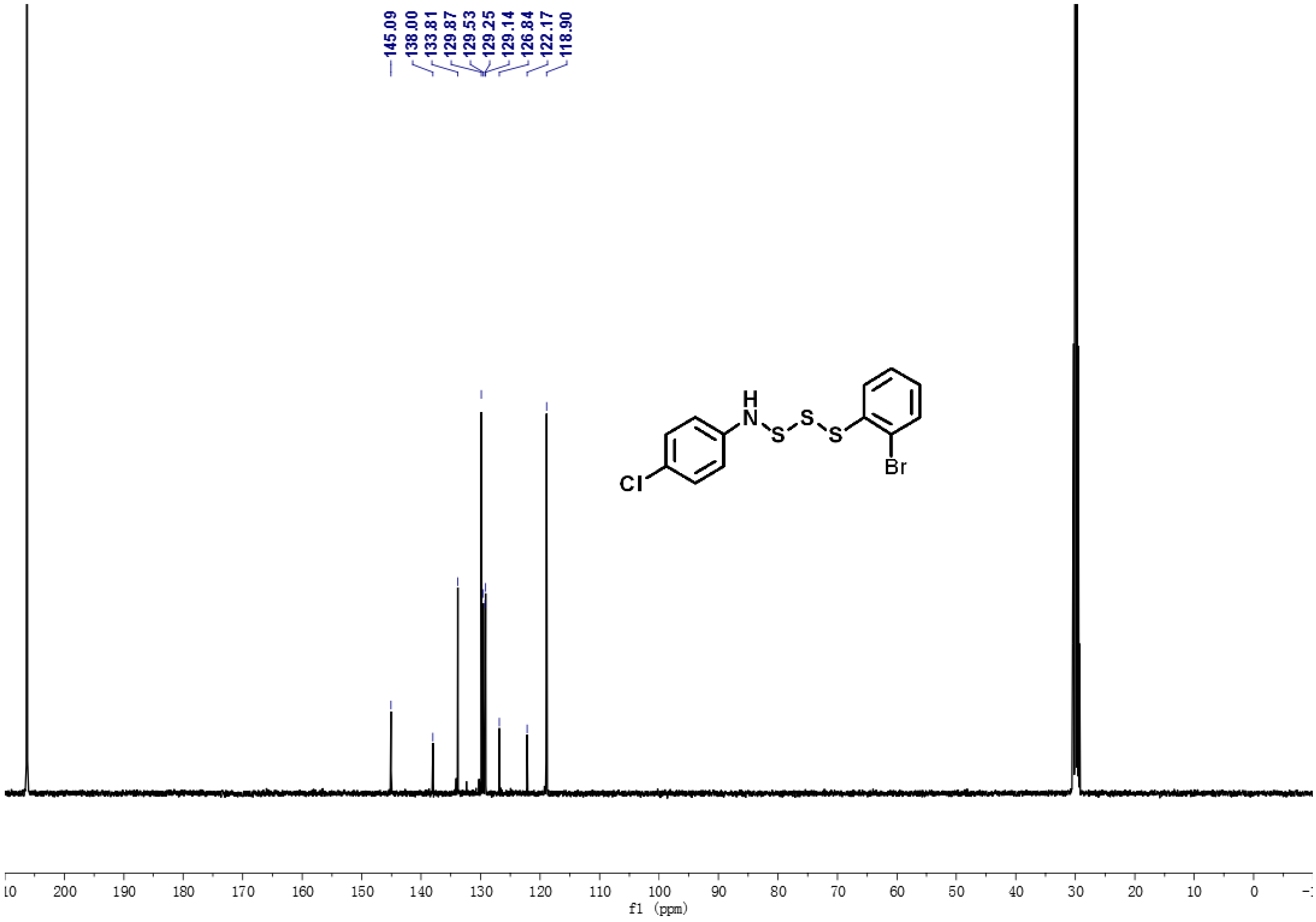

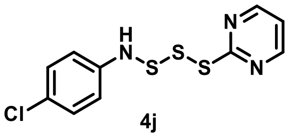

4j

^{1}H NMR (Acetone-d$_6$)

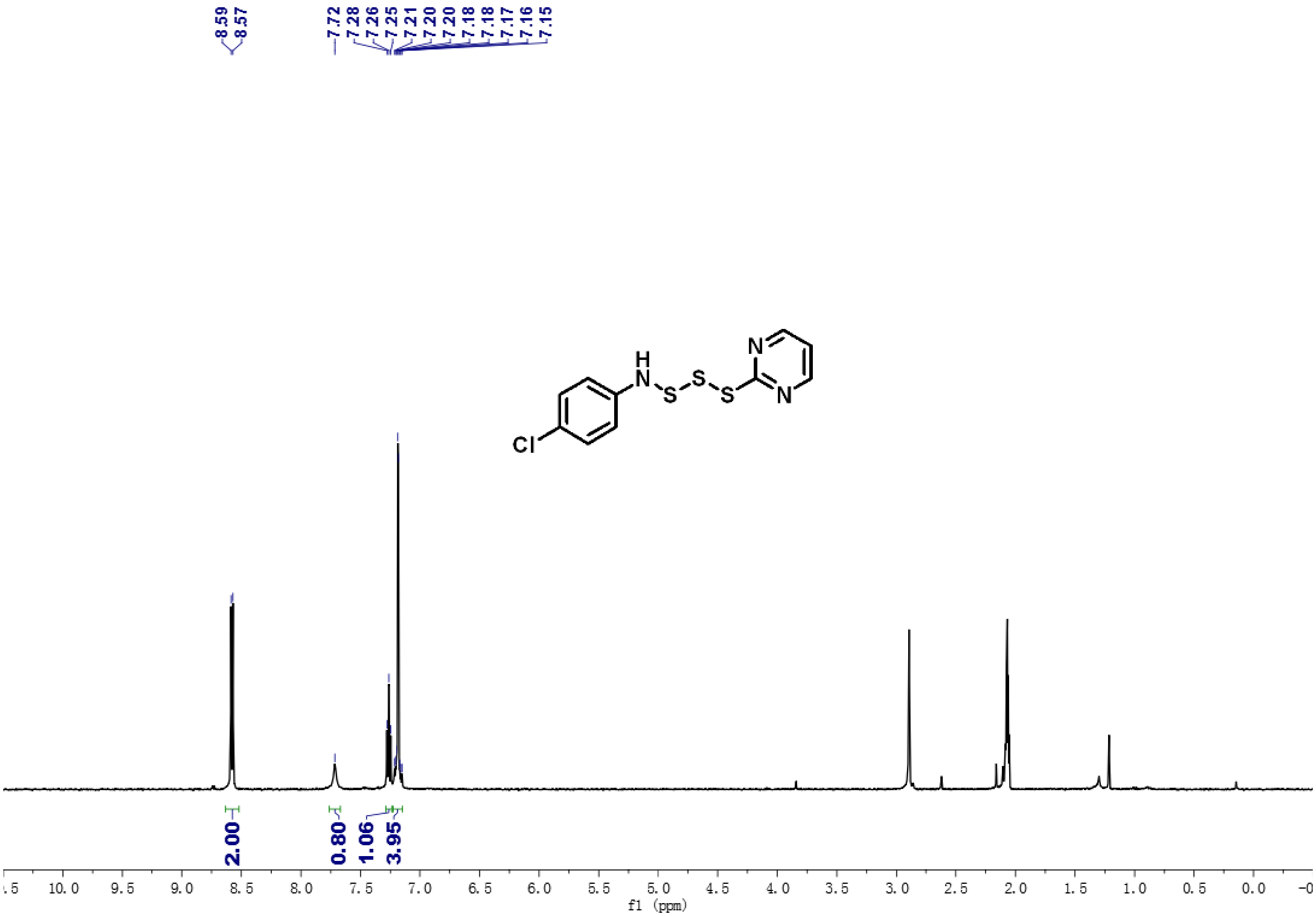

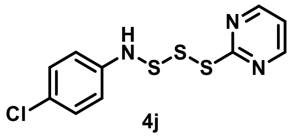

4j

^{1}H NMR (Acetone-d6), ^{13}C NMR (Acetone-d6)

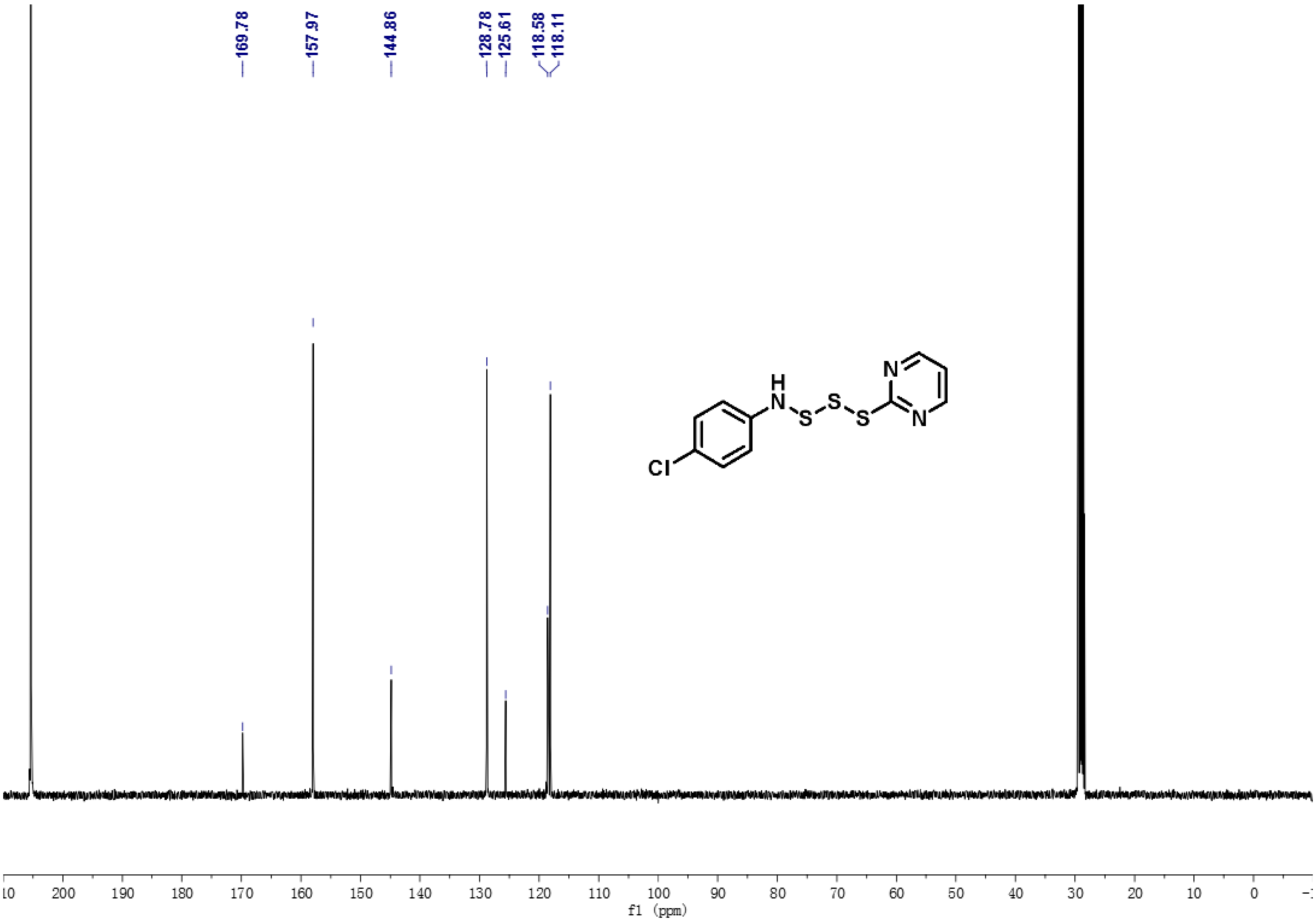

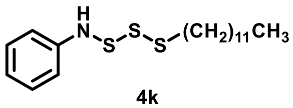

4k

^{1}H NMR (CD_3CN)

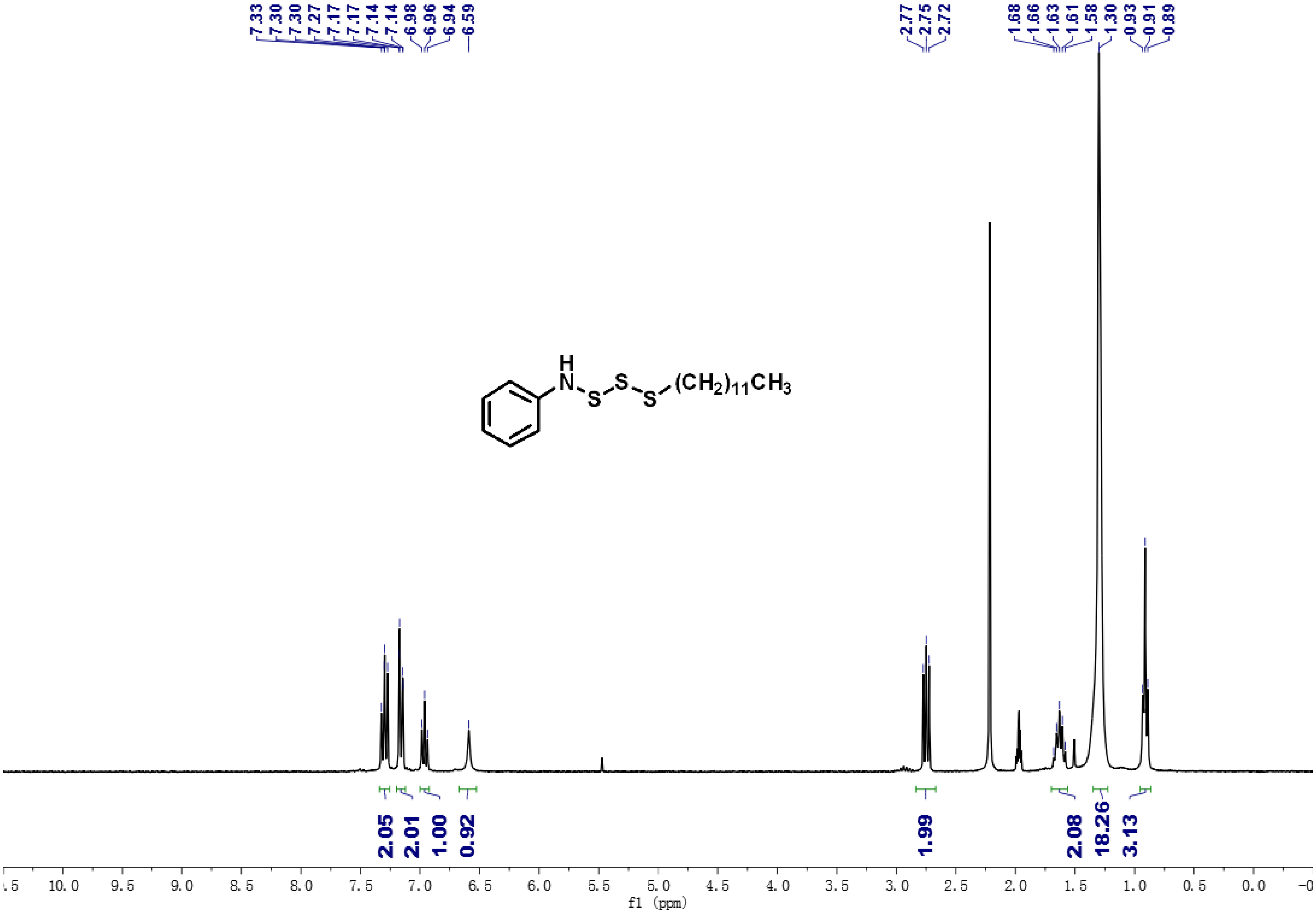

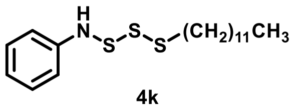

4k

^{13}C NMR (CD_3CN)

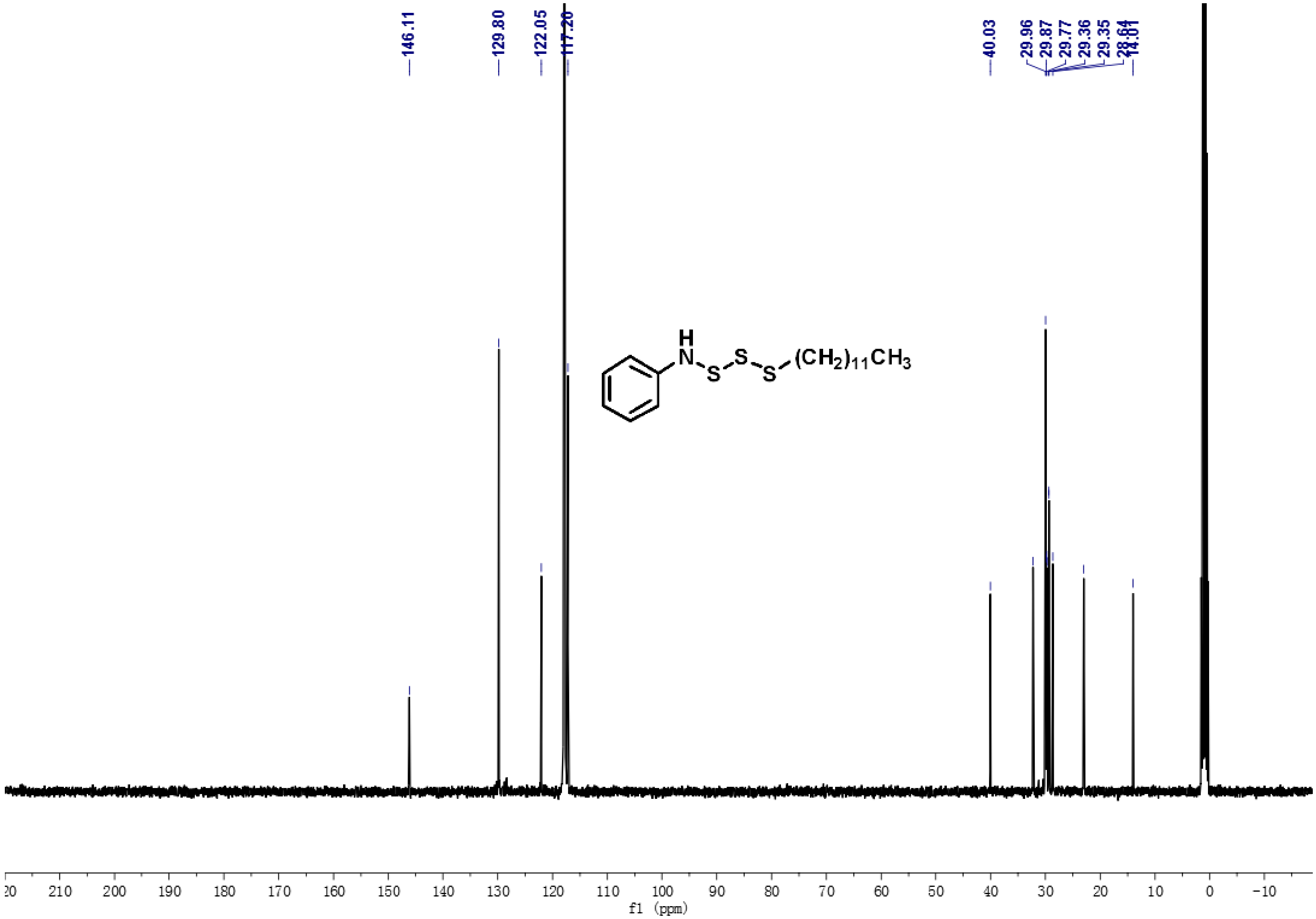

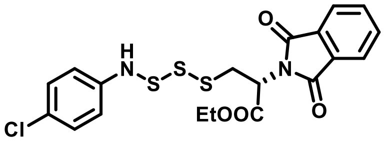

4l

^{1}H NMR (Acetone-d6)

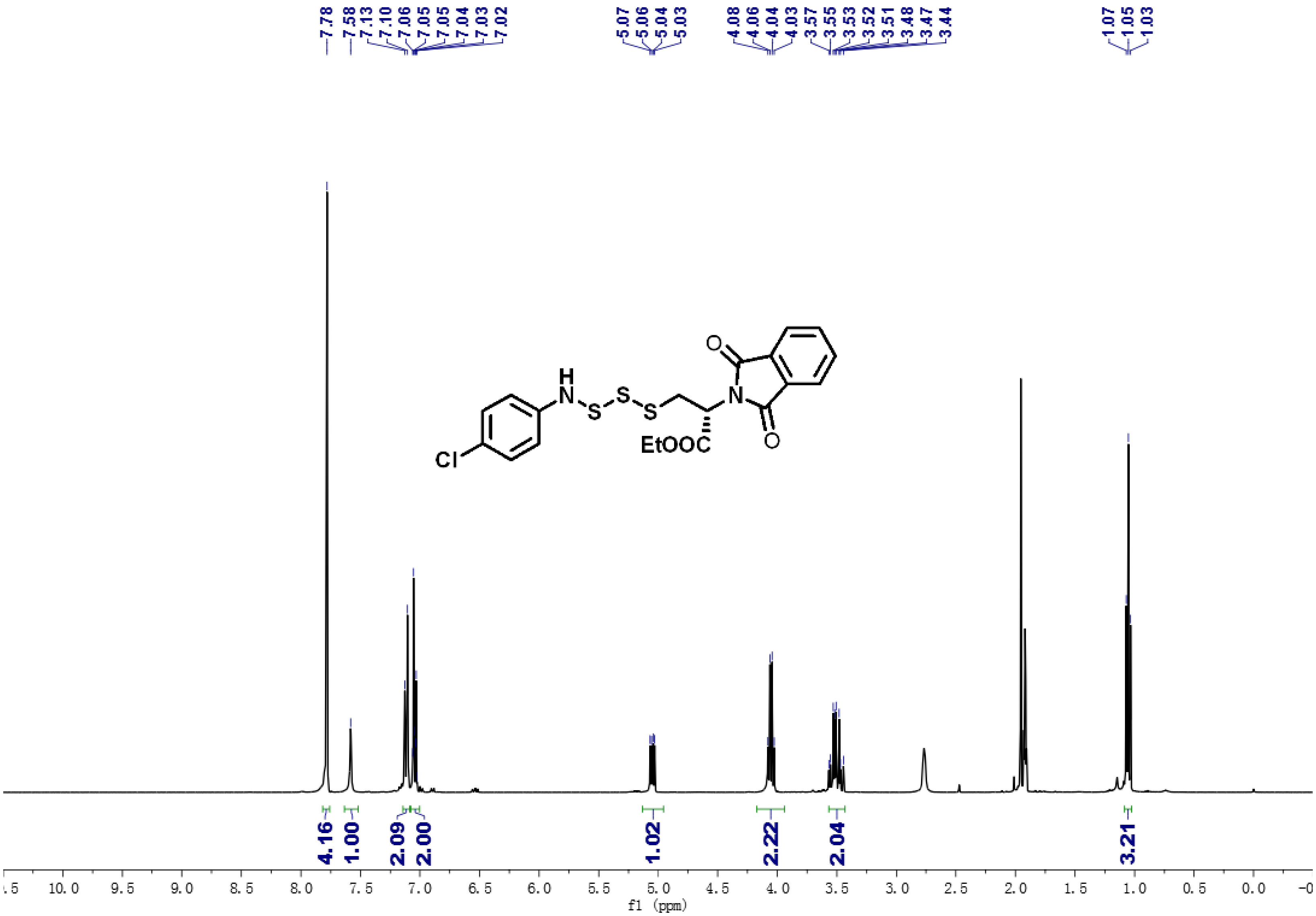

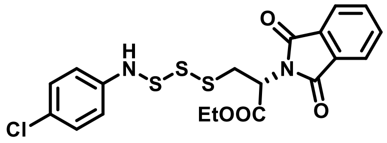

4l

^{13}C NMR (Acetone-d6)

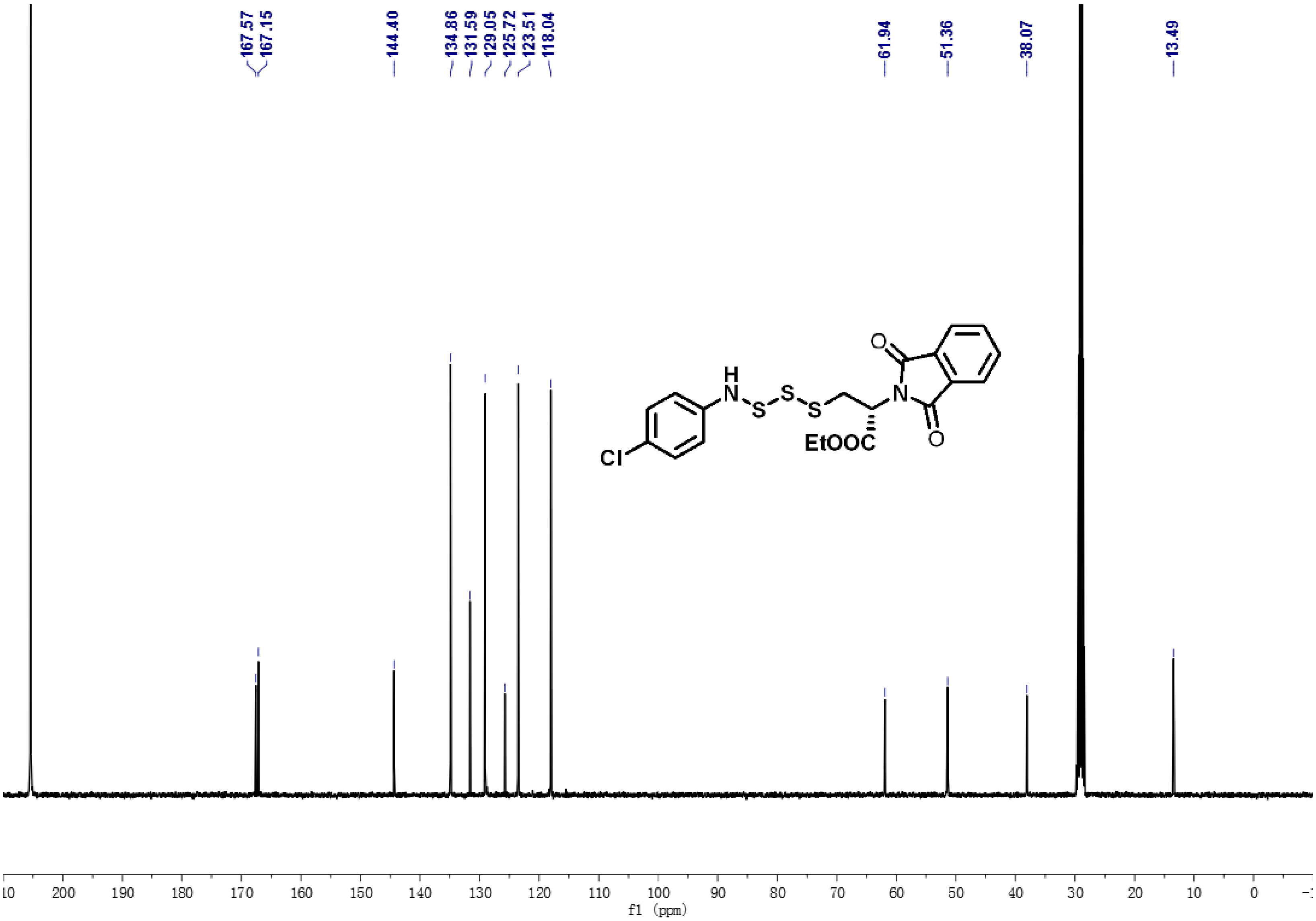

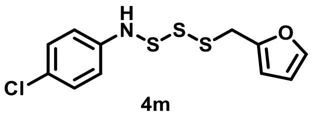

4m

^{1}H NMR (Acetone-d$_6$)

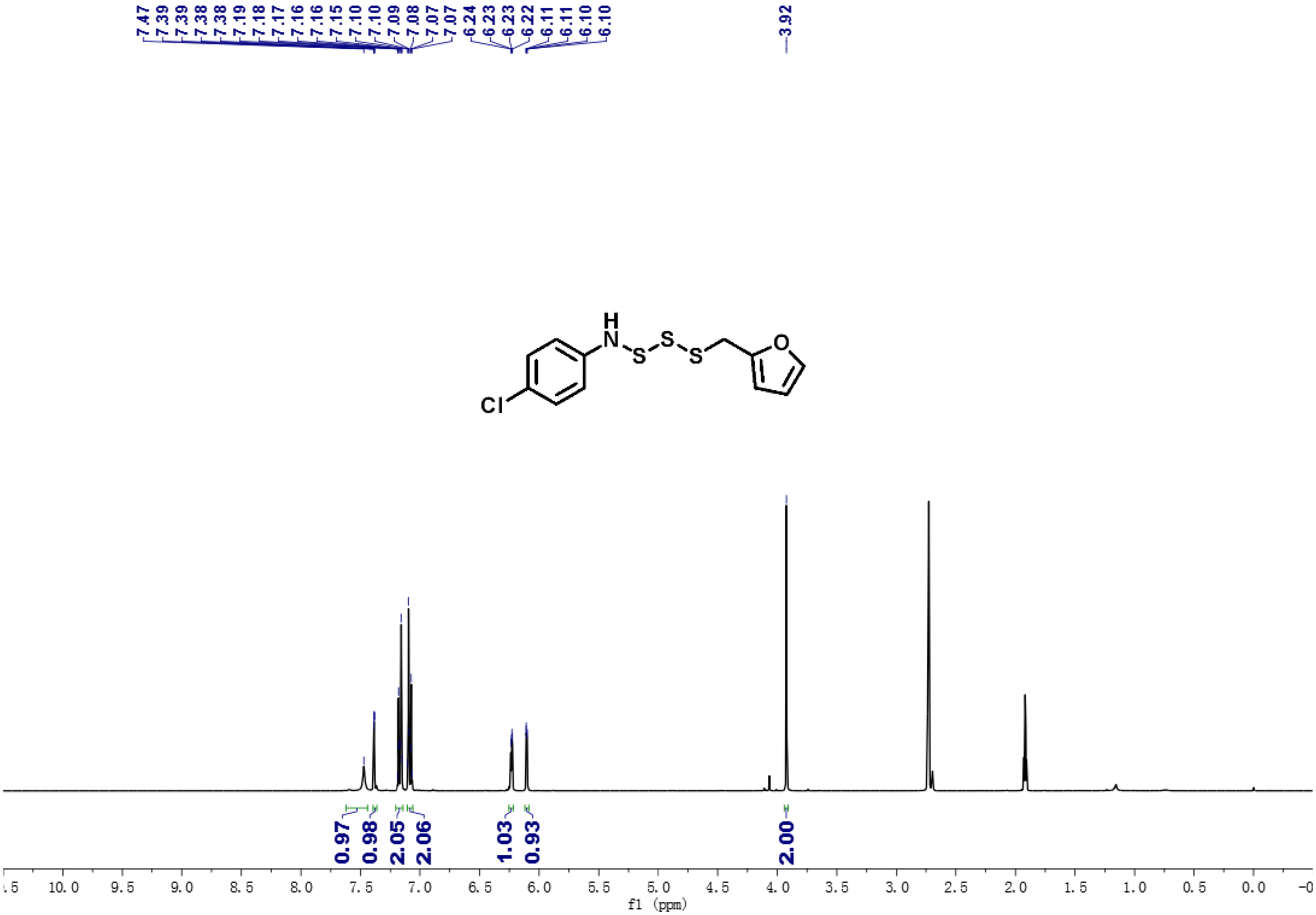

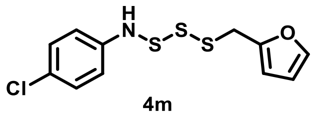

4m

^{13}C NMR (Acetone-d_6)

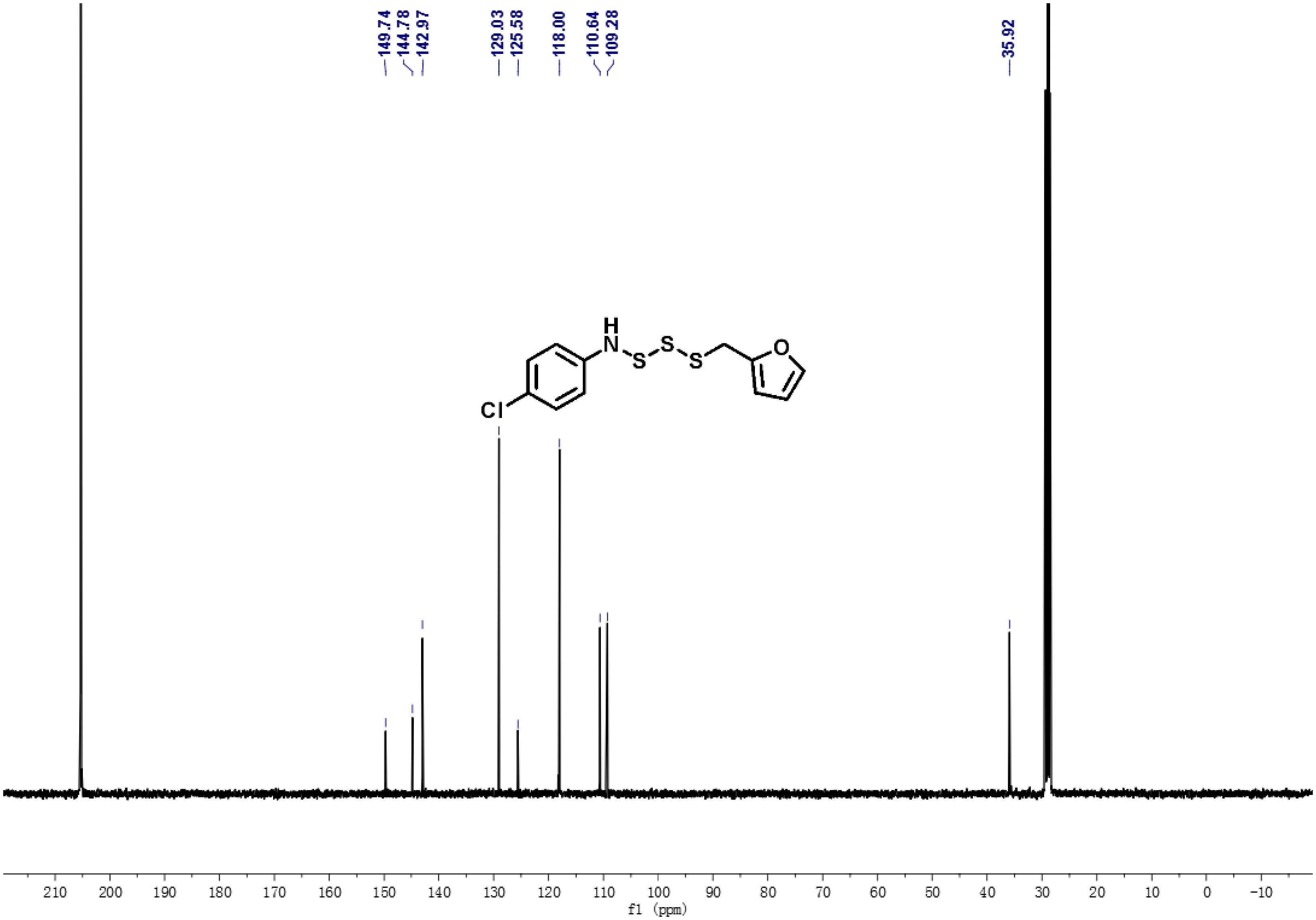

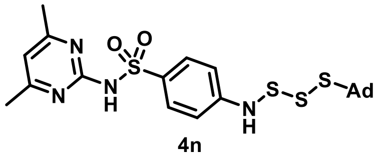

4n

^{1}H NMR (Acetone-d6)

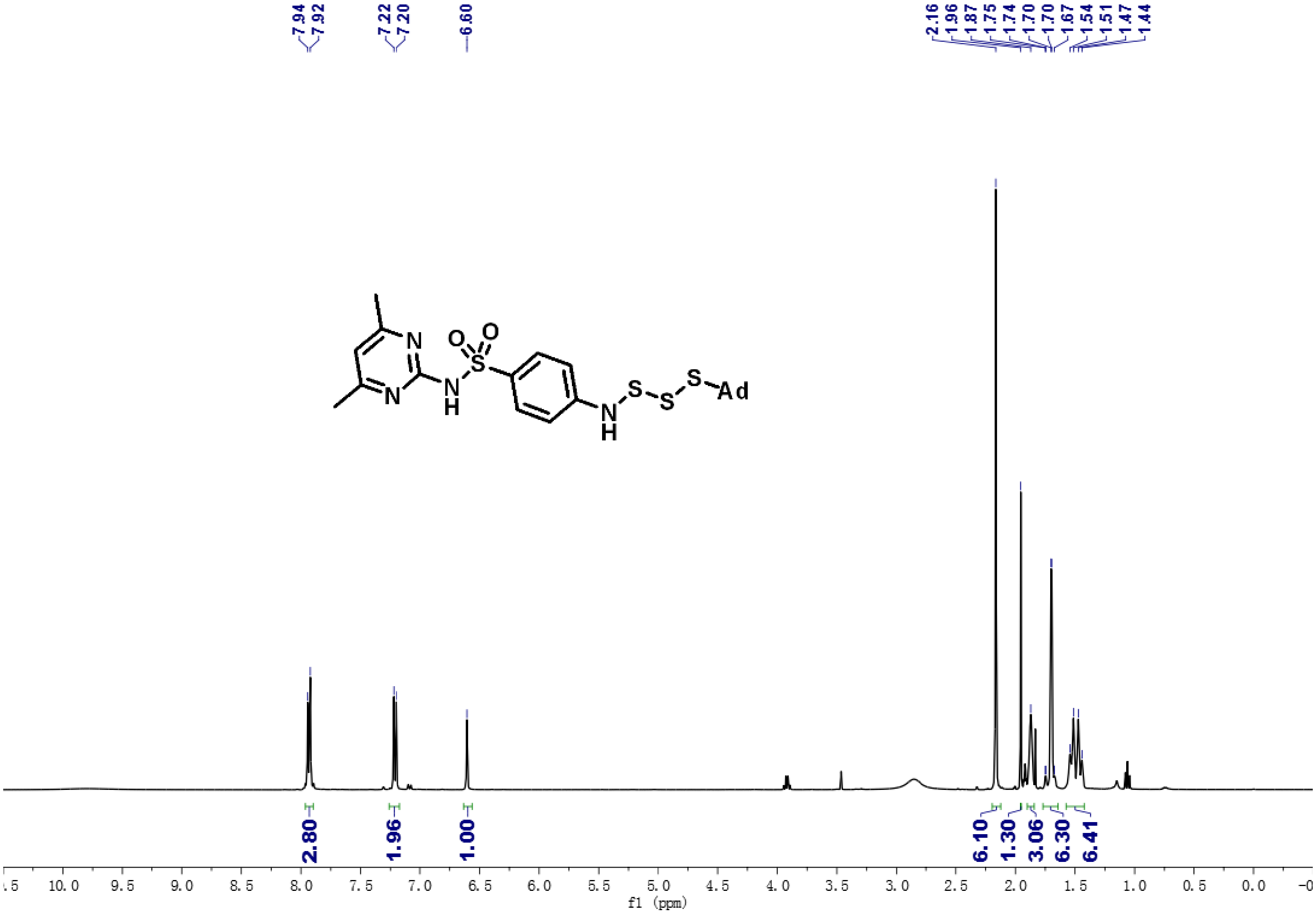

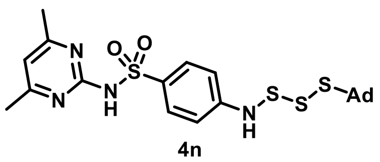

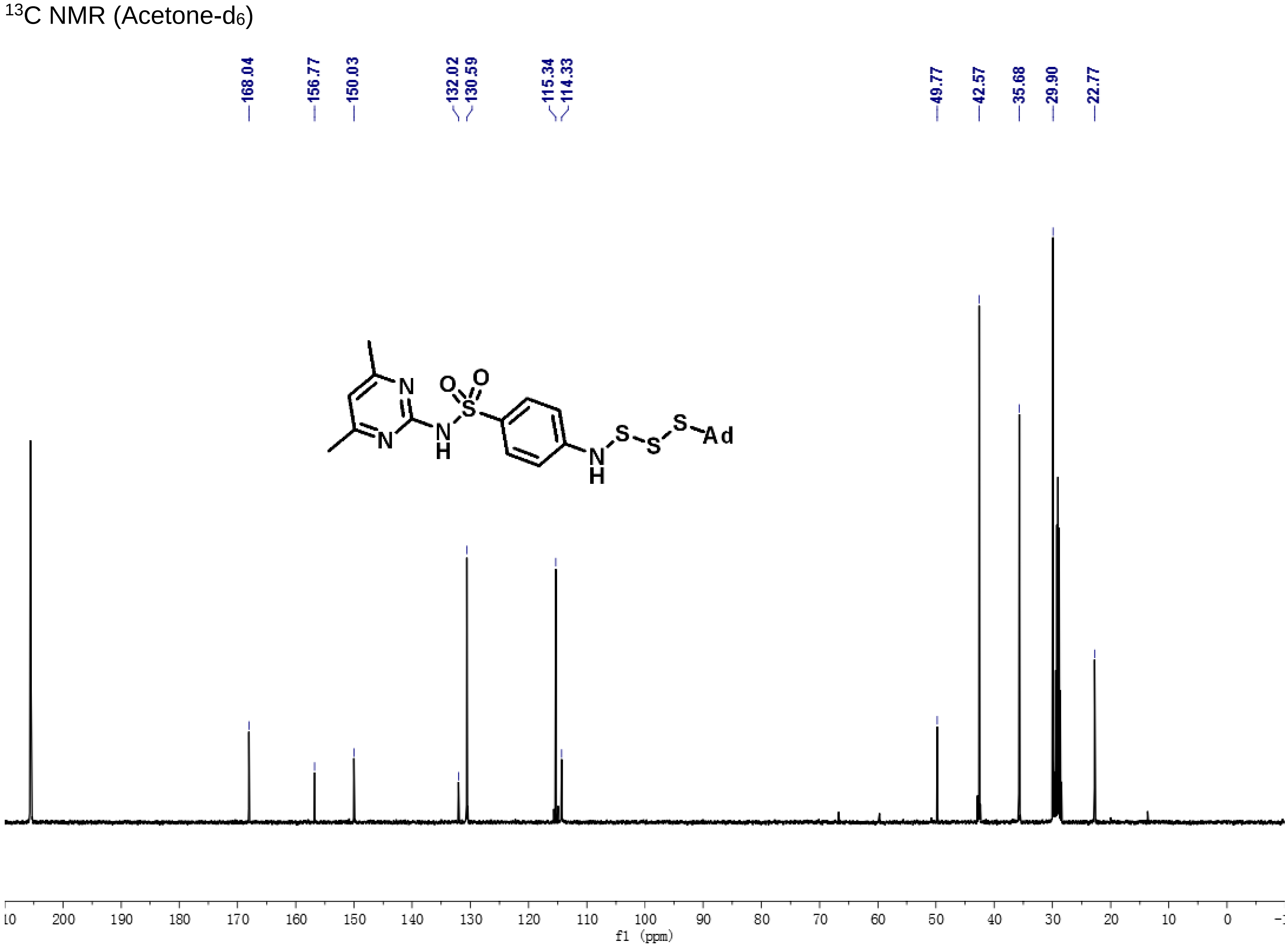

13C NMR (Acetone-d6)
168.04
156.77
150.03
132.02
130.59
115.34
114.33
49.77
42.57
35.68
29.90
22.77
f1 (ppm)

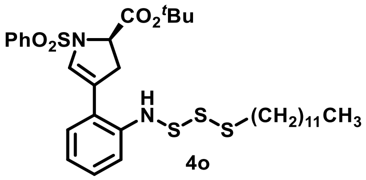

4o

^{1}H NMR (Acetone-d6)

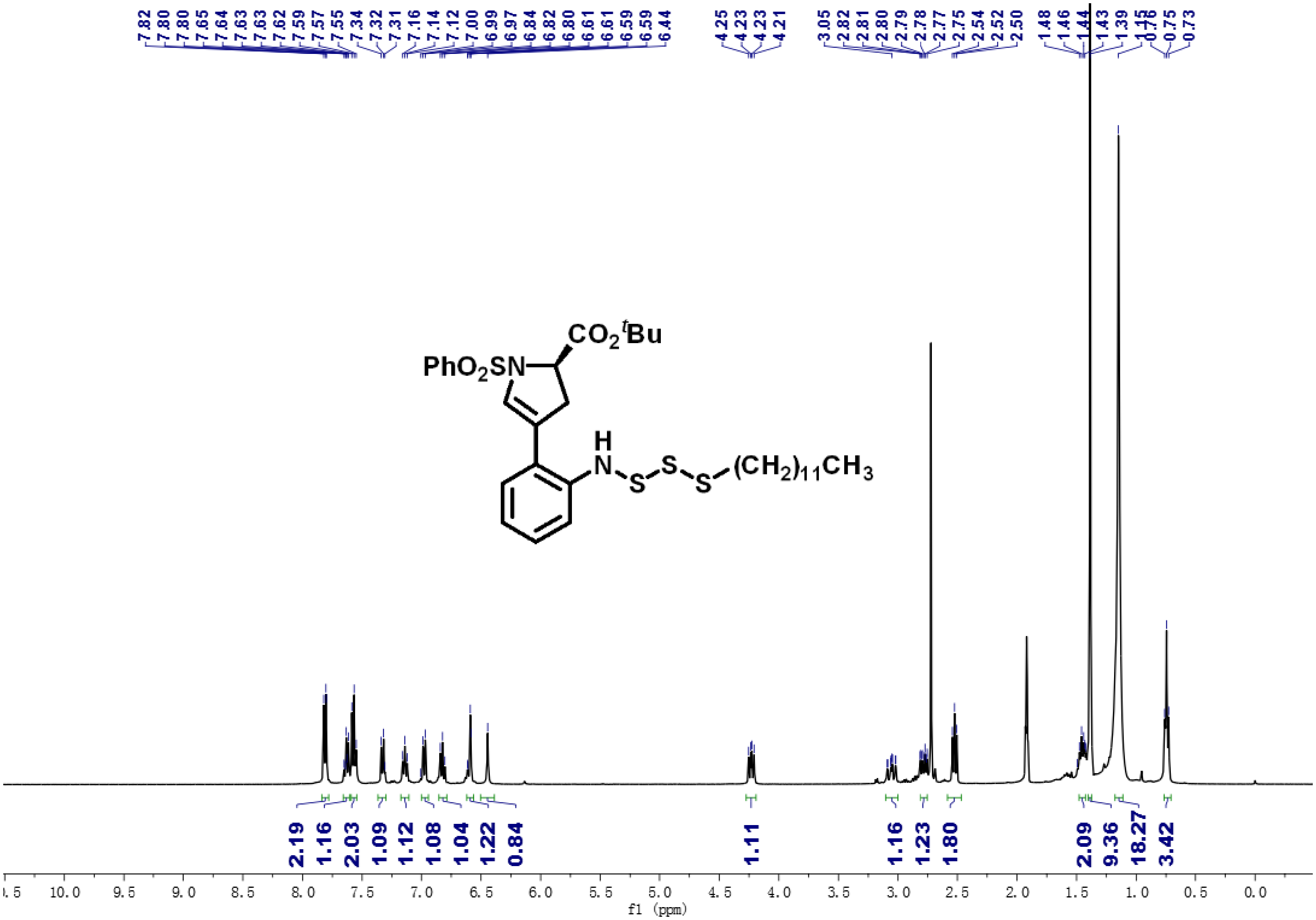

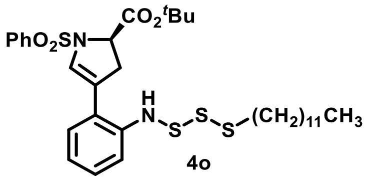

4o

^{13}C NMR (Acetone-d6)

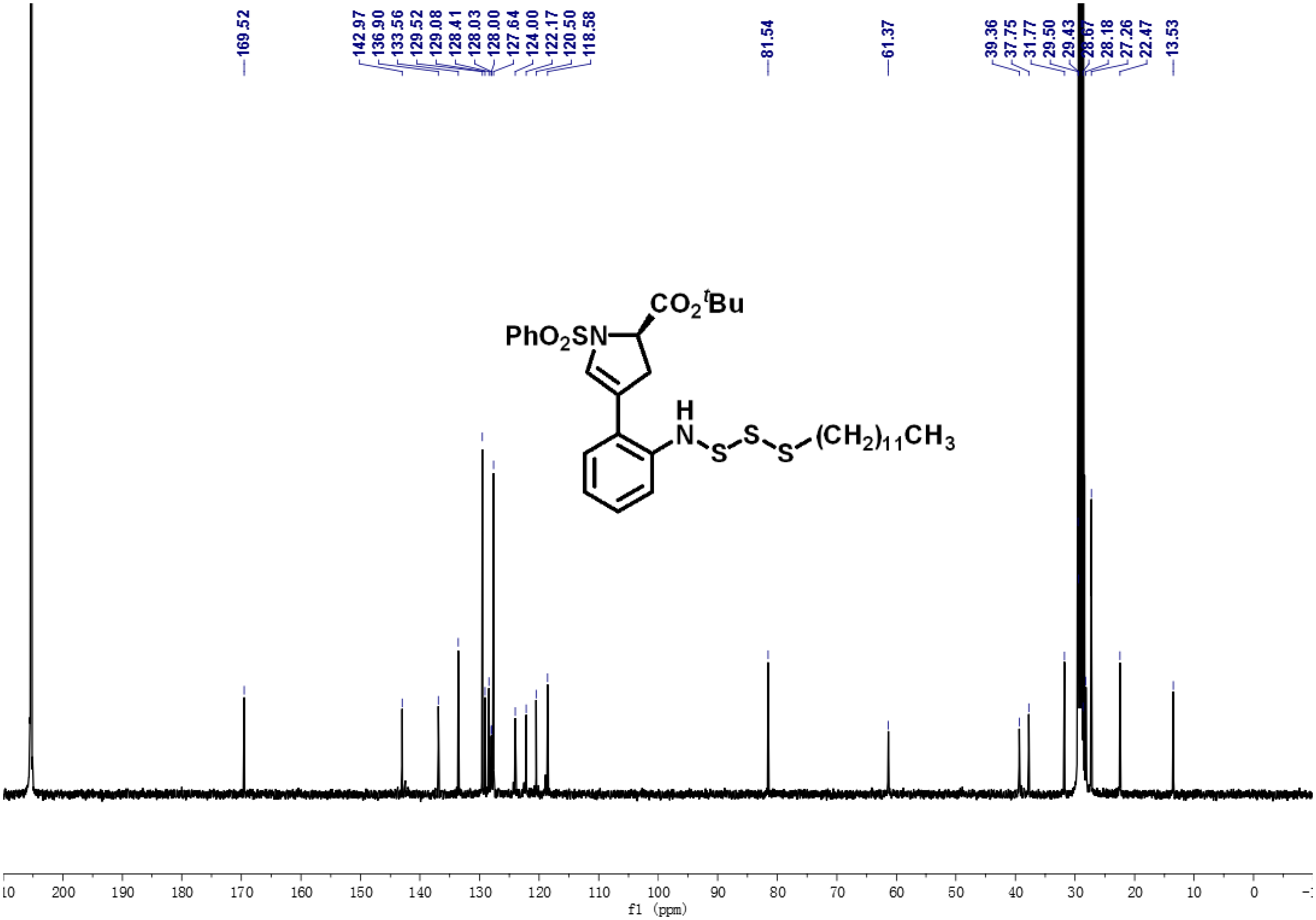

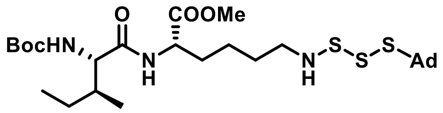

4p

^{1}H NMR (Acetone-d$_6$)

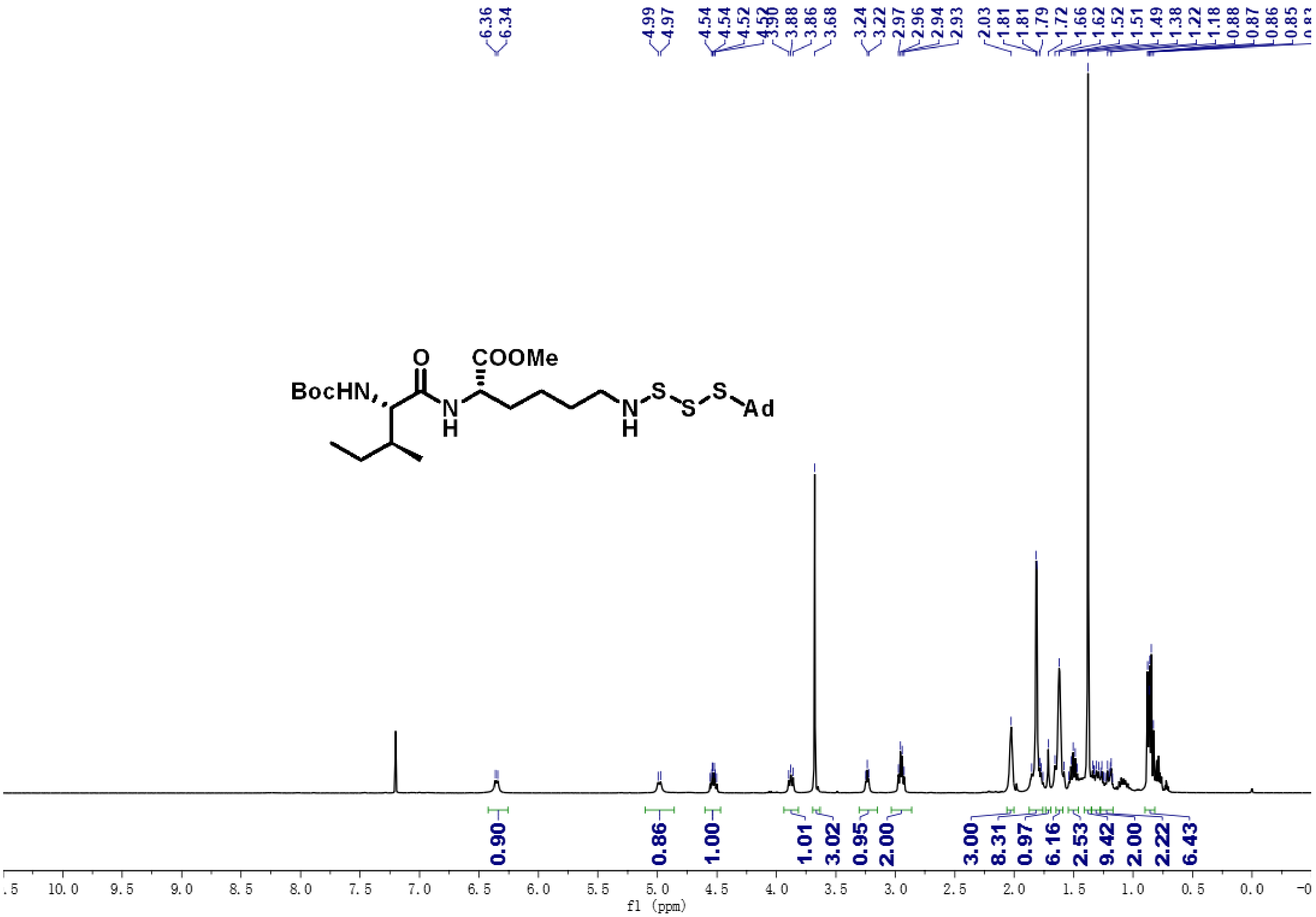

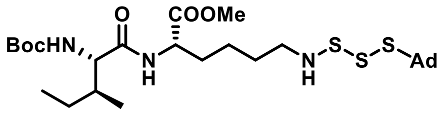

4p

^{13}C NMR (Acetone-d$_6$)

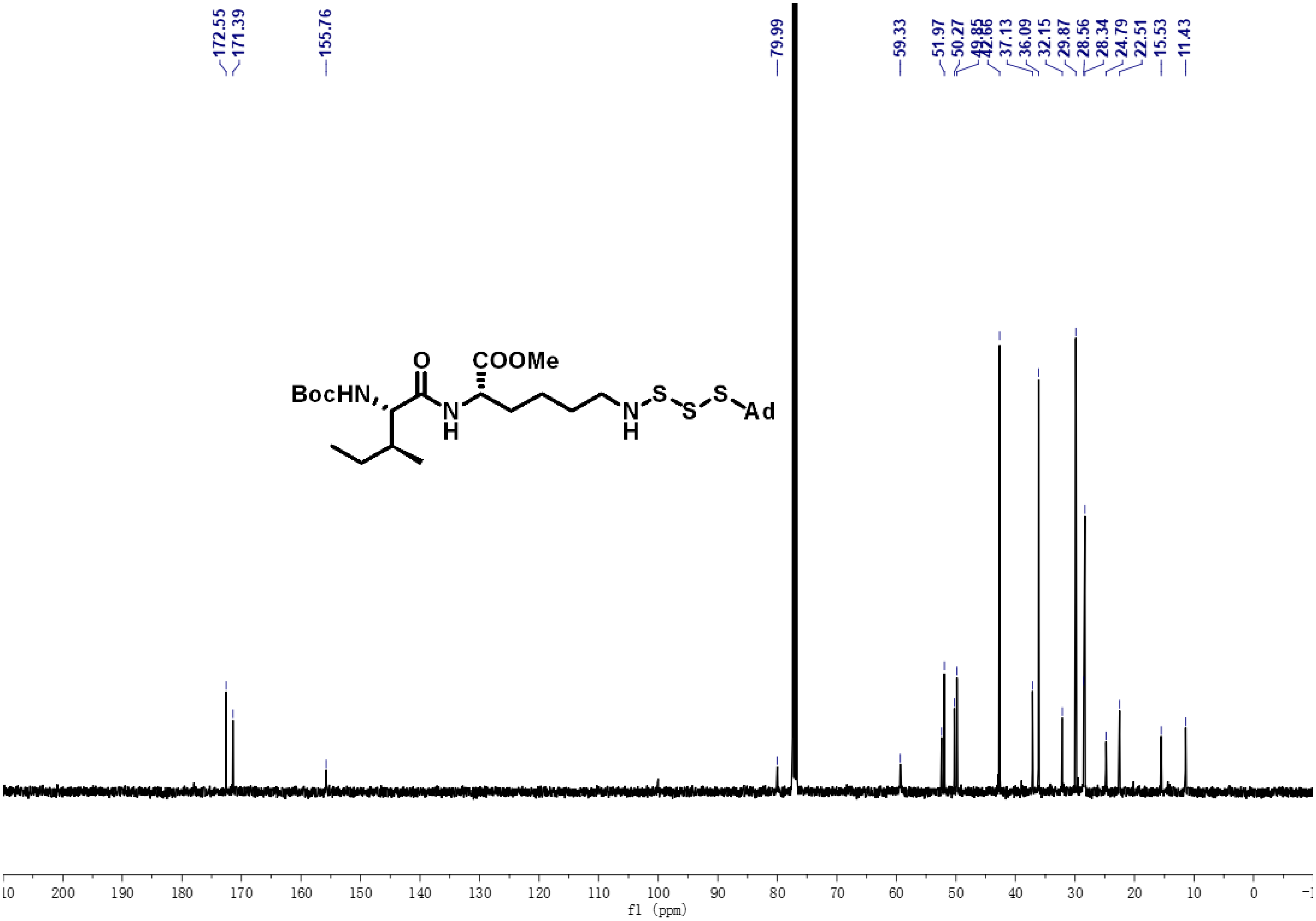

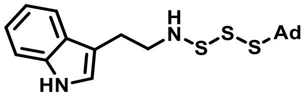

4q

^{1}H NMR (Acetone-d_6)

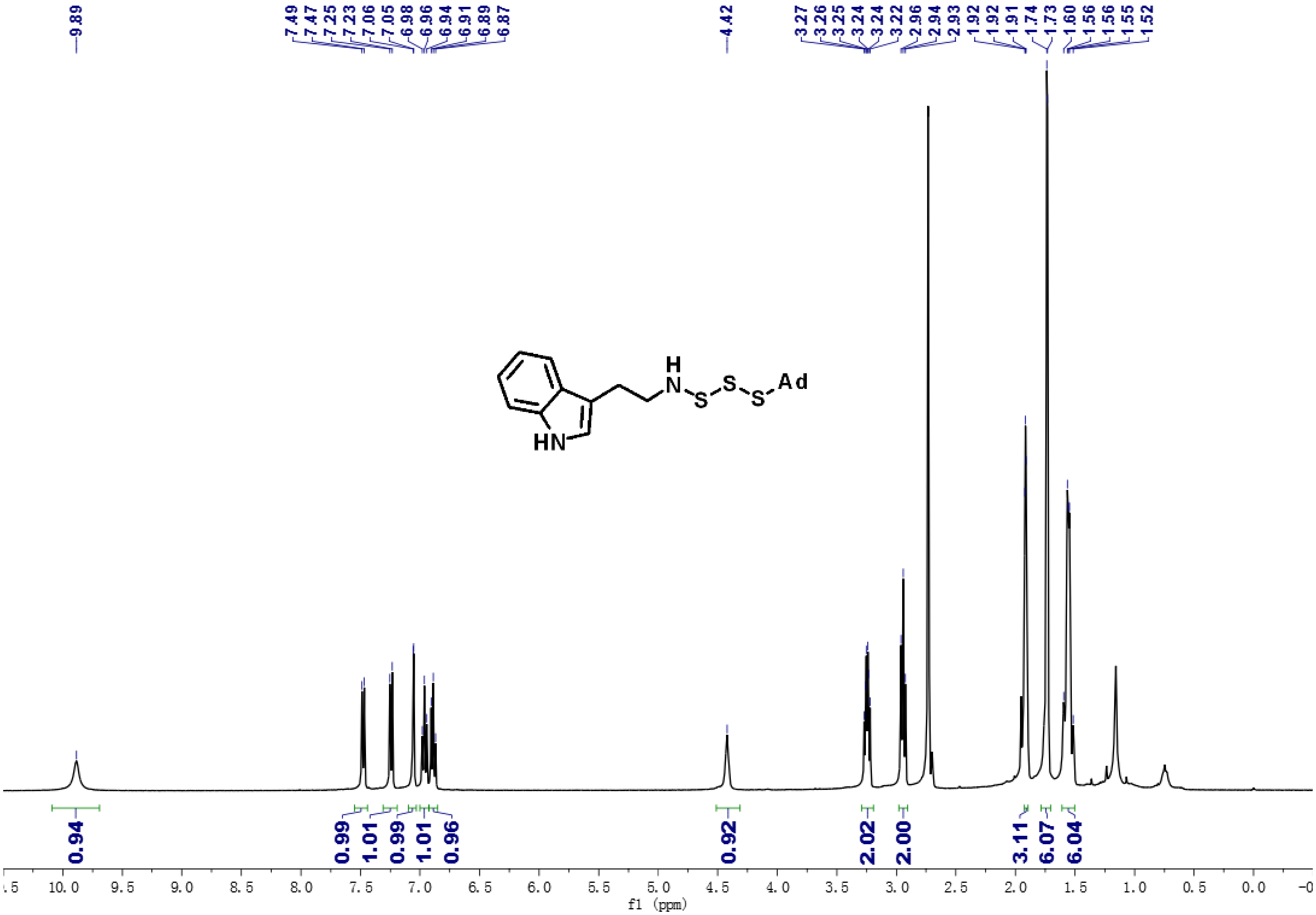

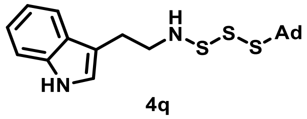

4q

^{13}C NMR (Acetone-d$_6$)

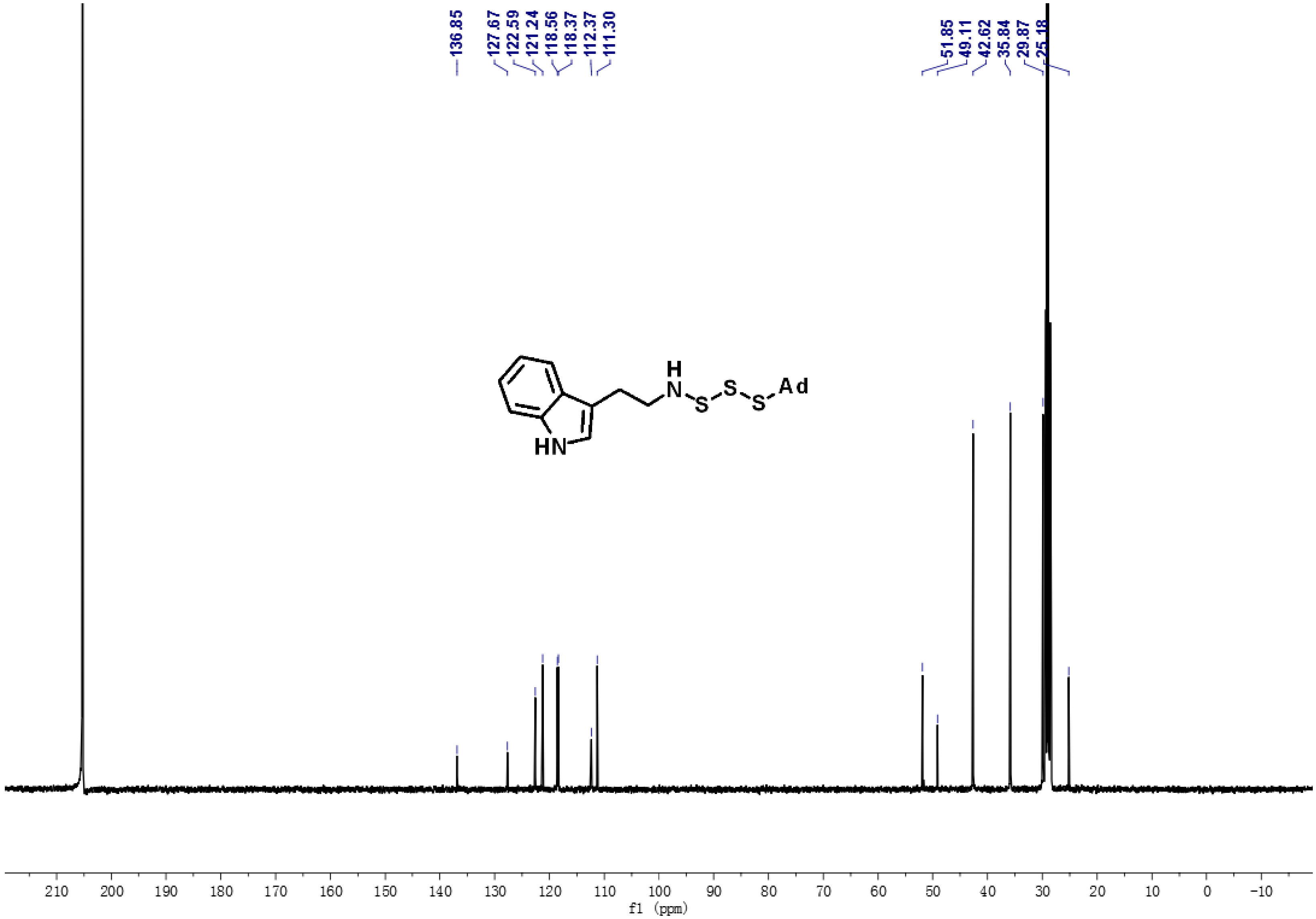

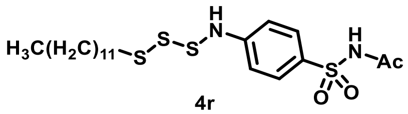

4r

^{1}H NMR ($CDCl_3$)

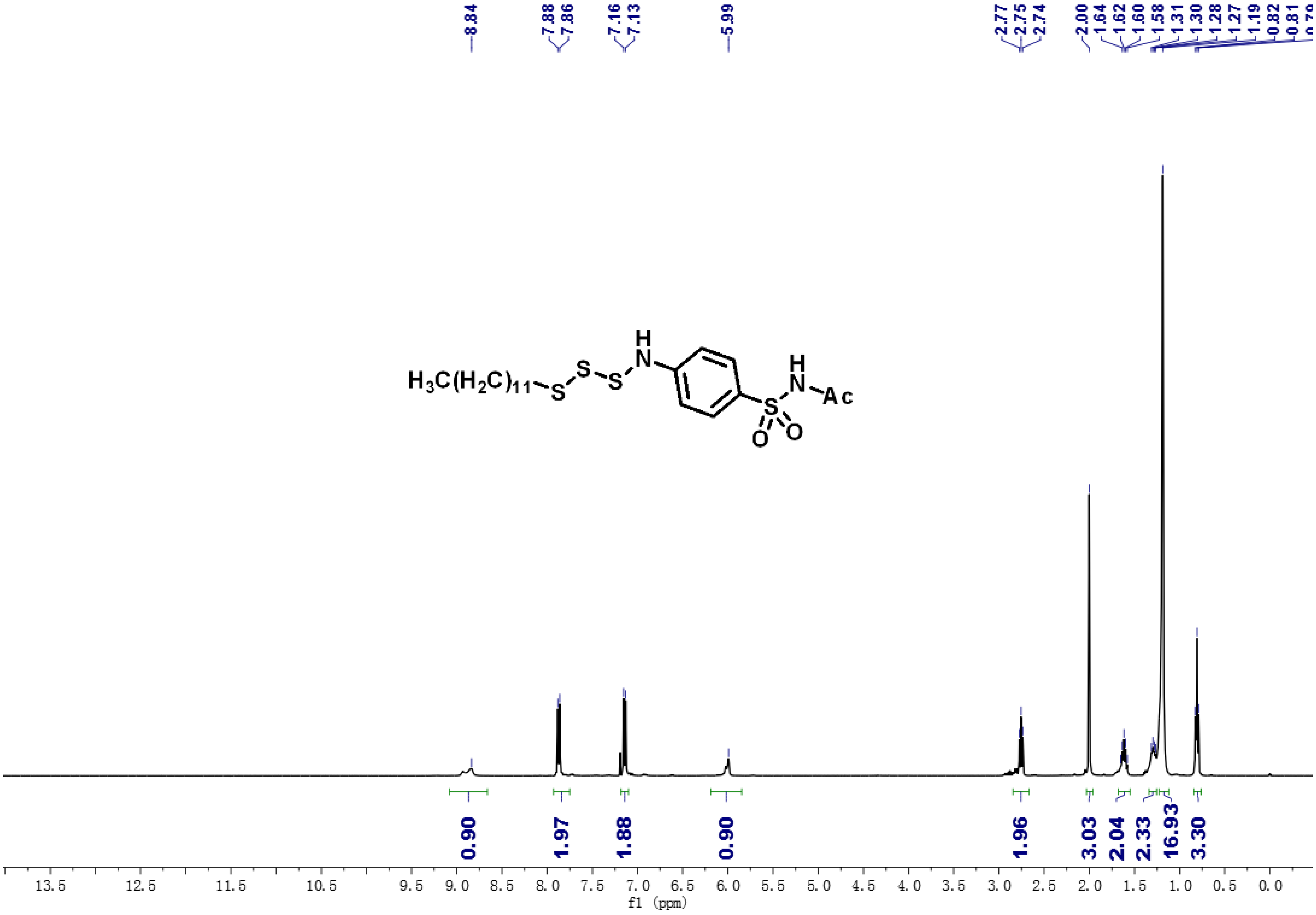

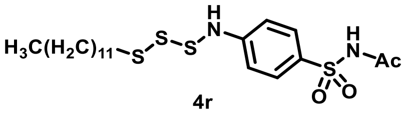

4r

^{13}C NMR (Acetone-d6)

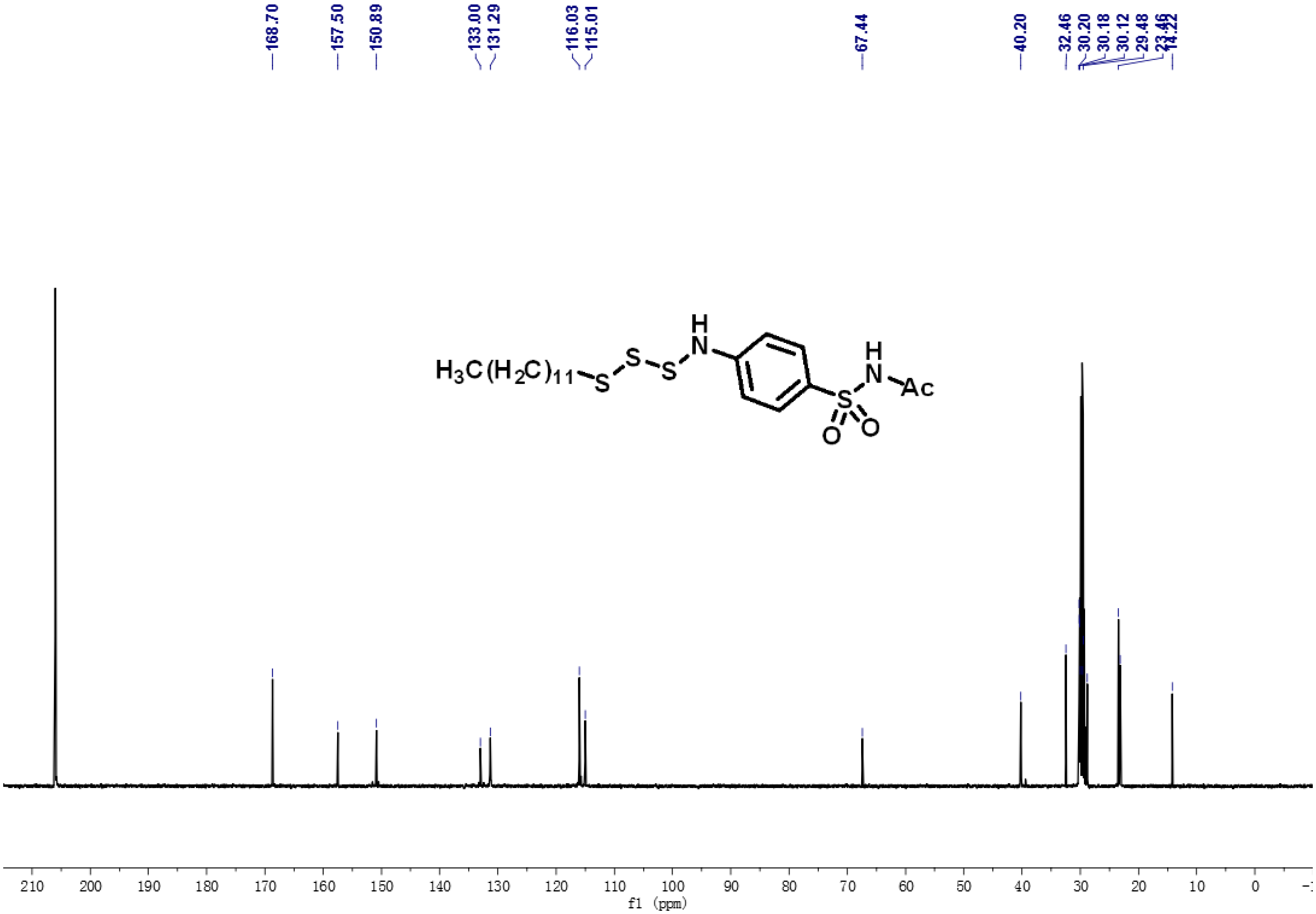

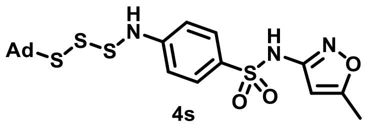

4s

^{1}H NMR (Acetone-d6)

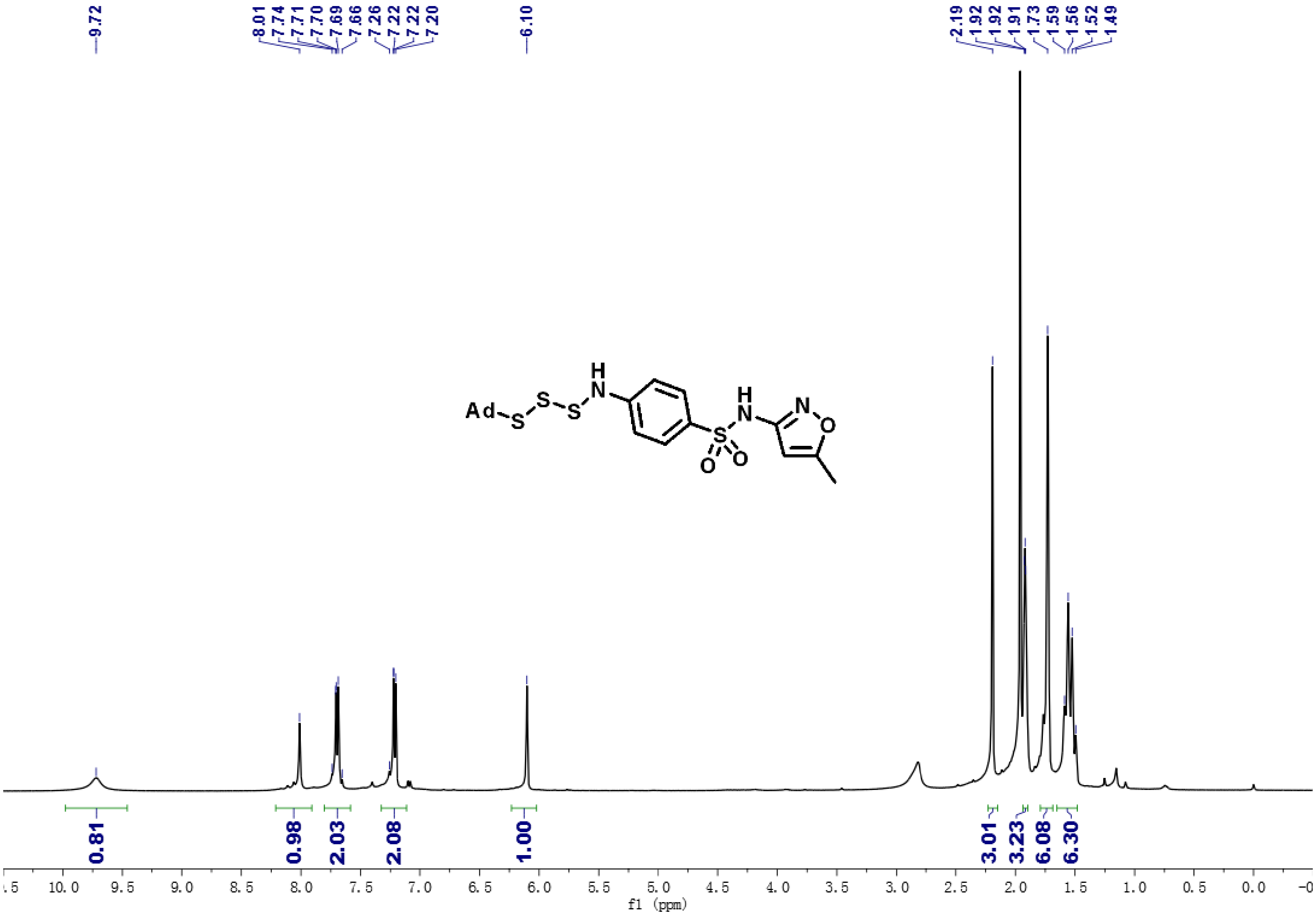

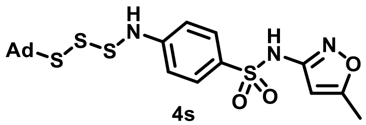

4s

^{13}C NMR (Acetone-d_6)

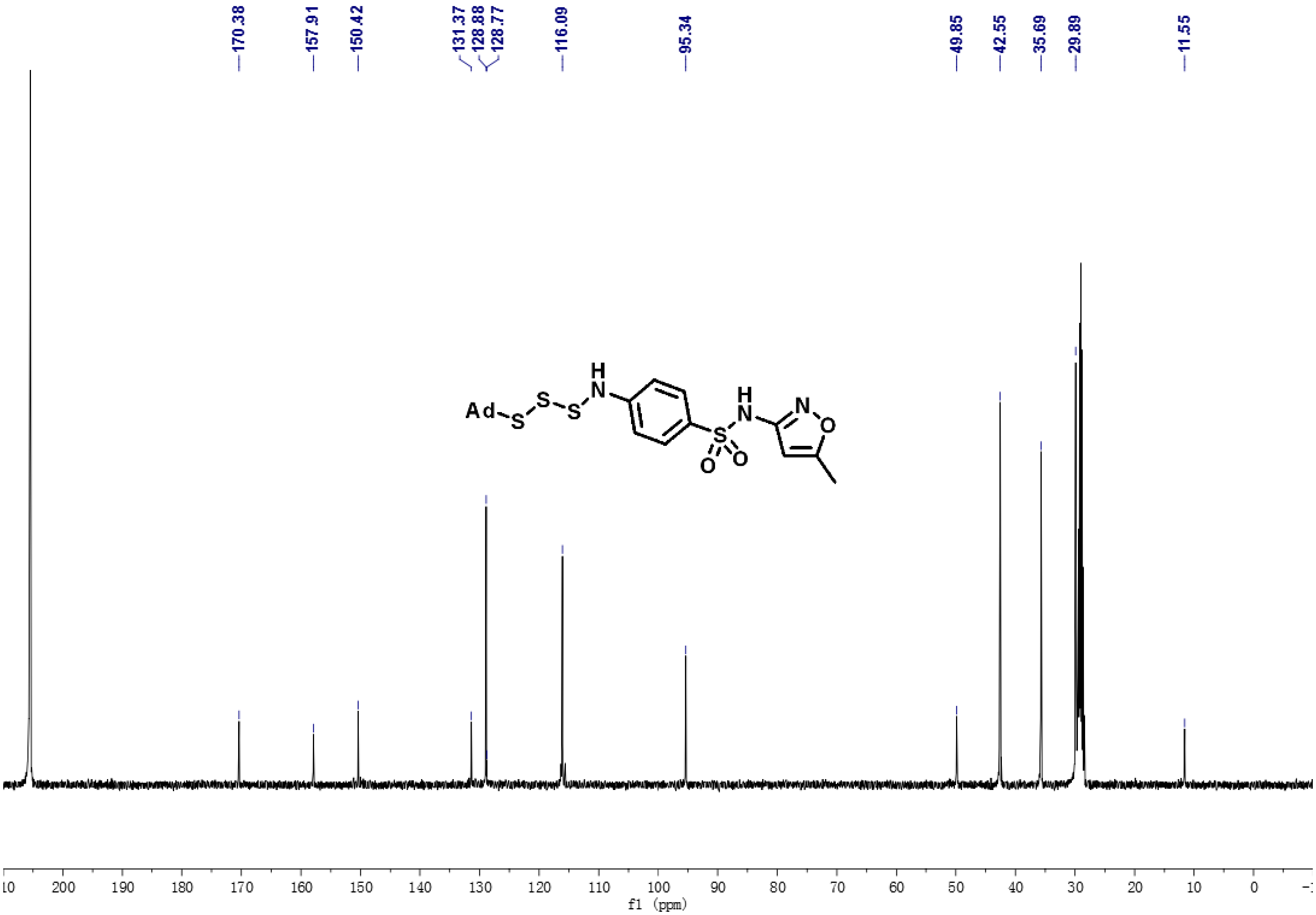

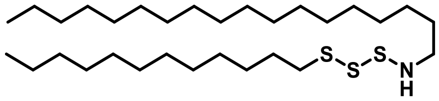

4t

^{1}H NMR ($CDCl_3$)

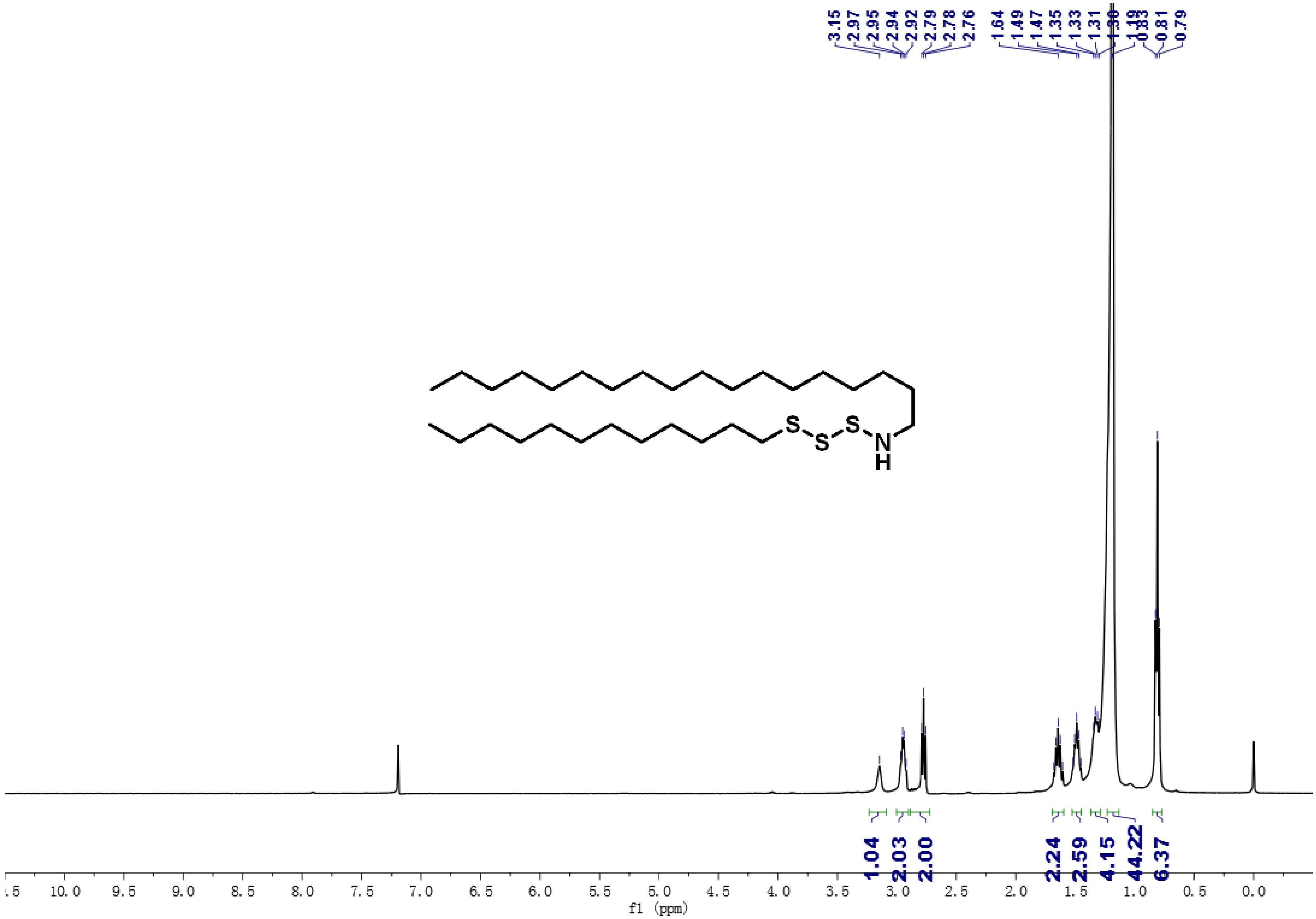

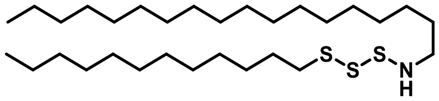

4t

^{13}C NMR ($CDCl_3$)

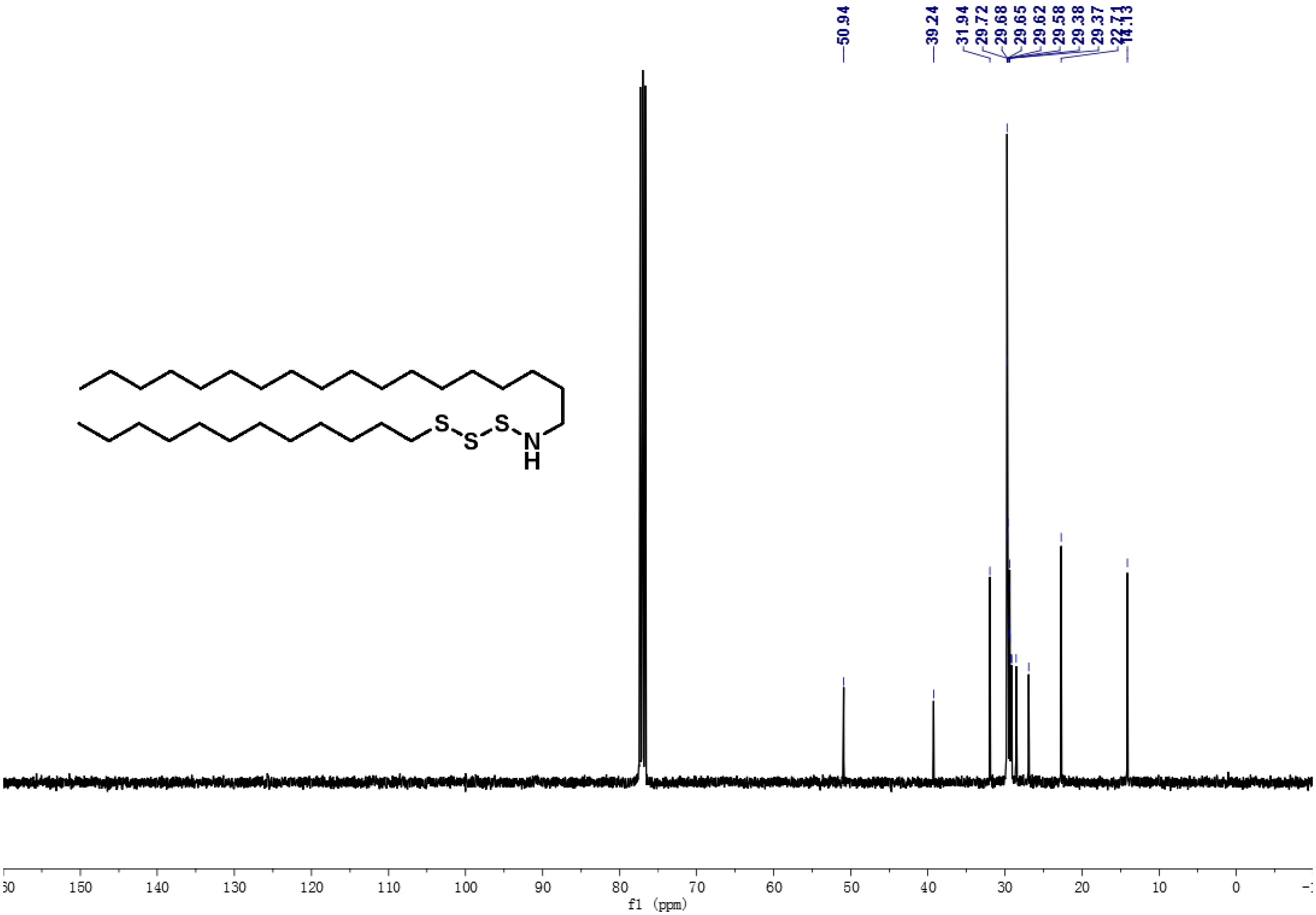

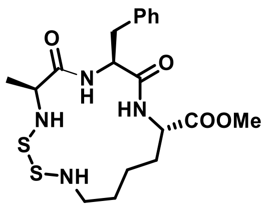

5a

^{1}H NMR (CD_3OD)

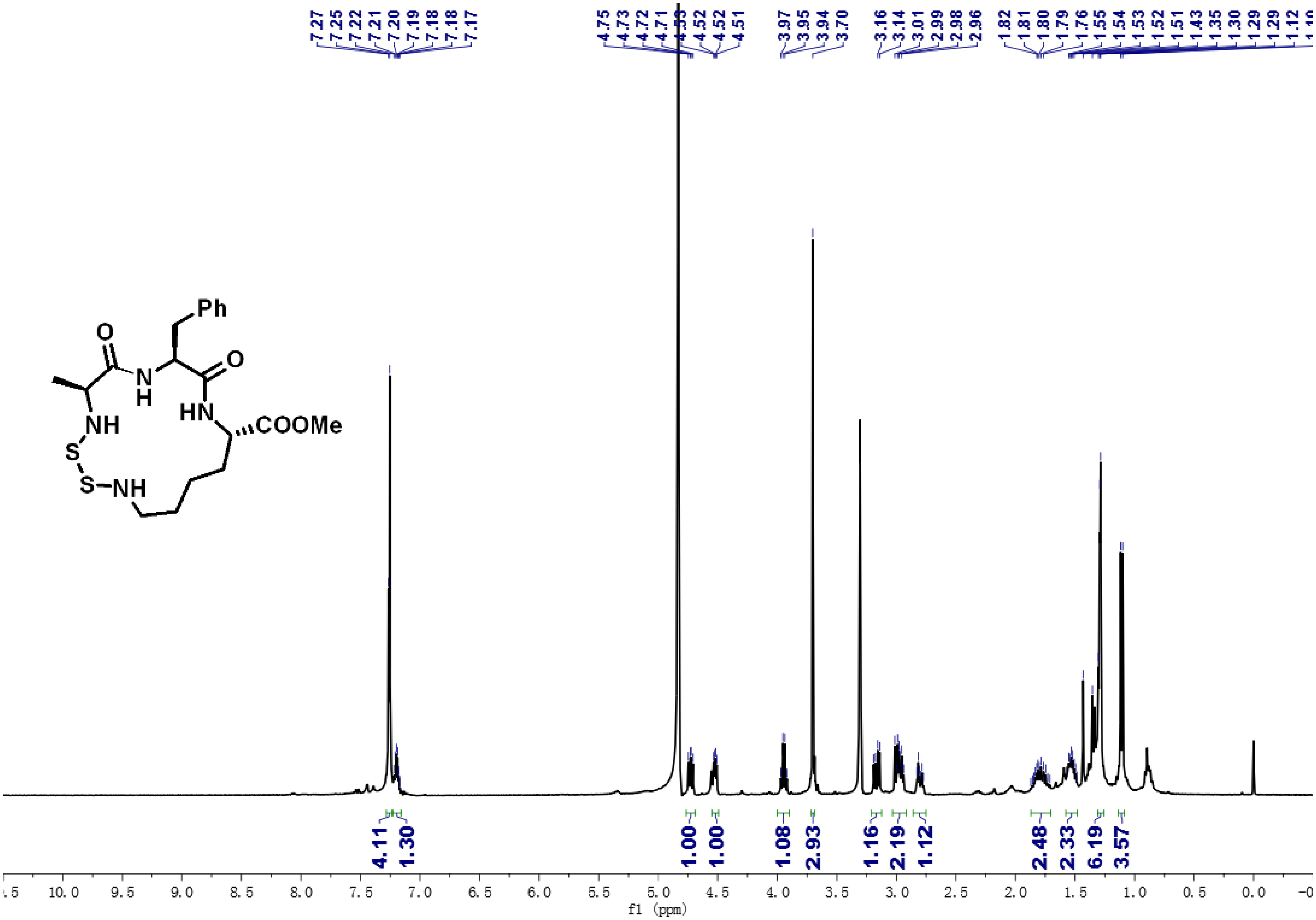

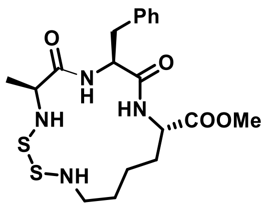

5a

^{13}C NMR (CD_3OD)

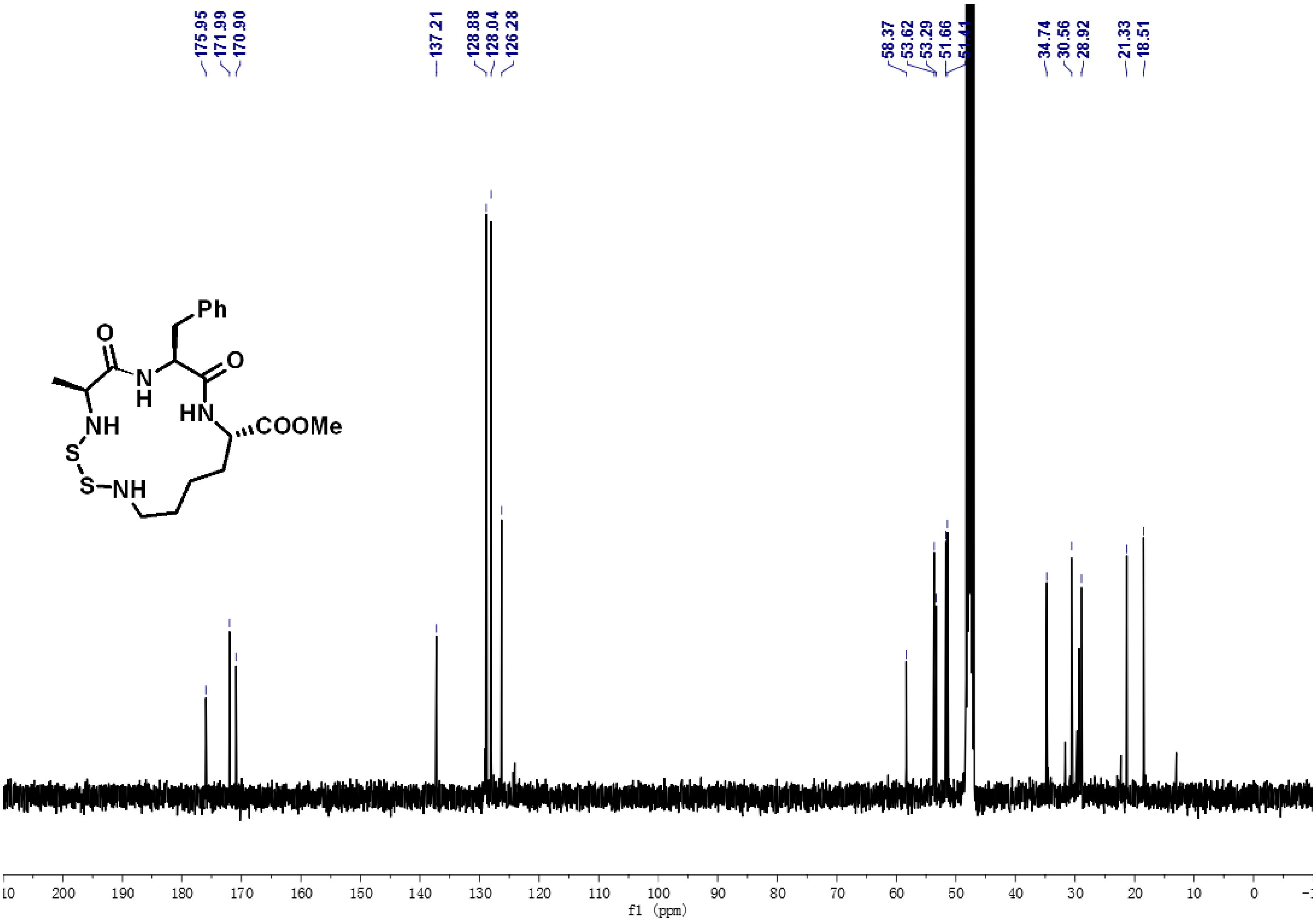

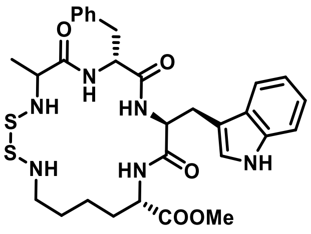

5b

^{1}H NMR (CD_3OD)

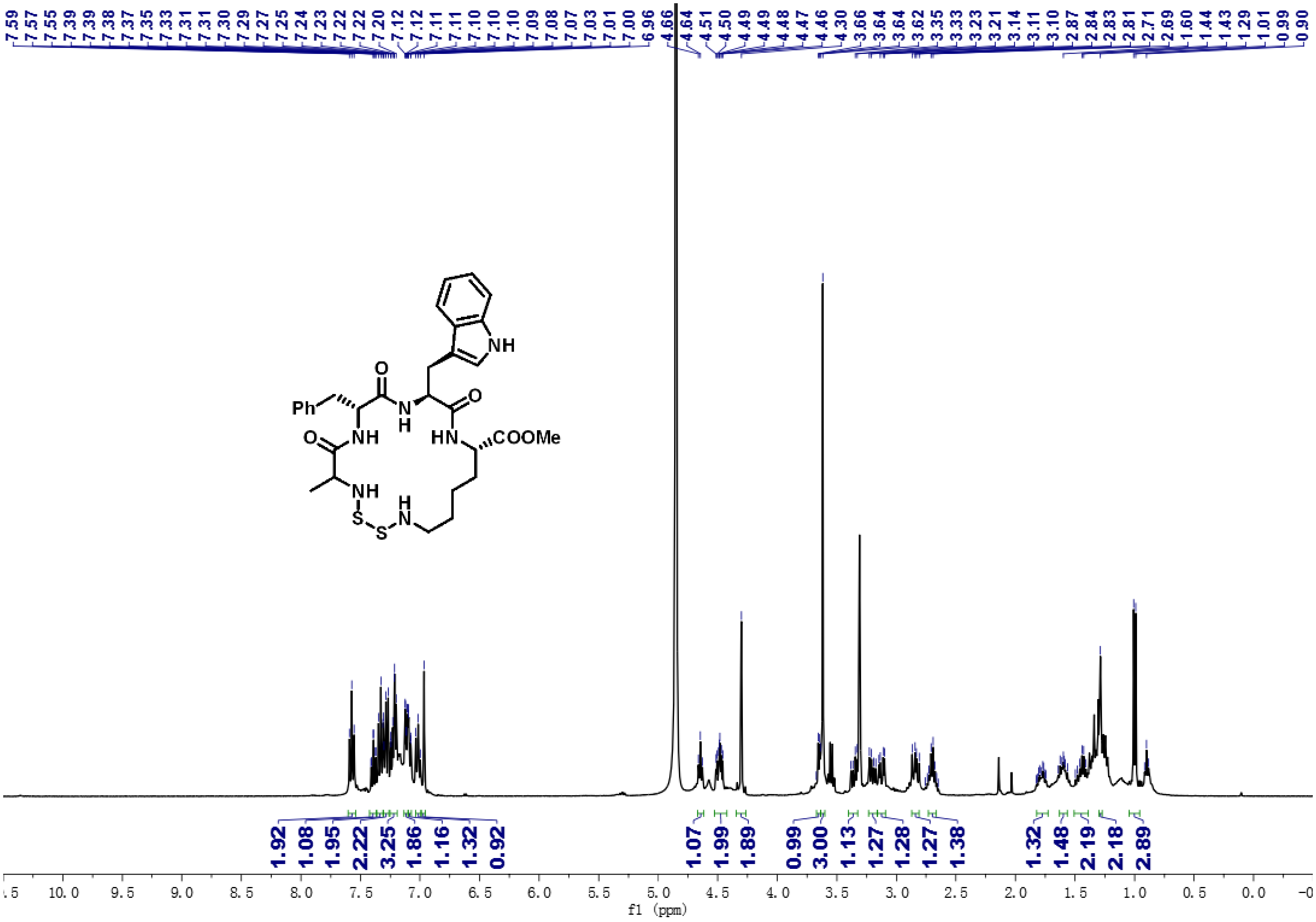

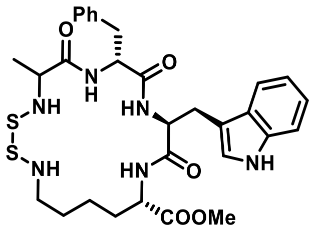

5b

^{13}C NMR (CD_3OD)

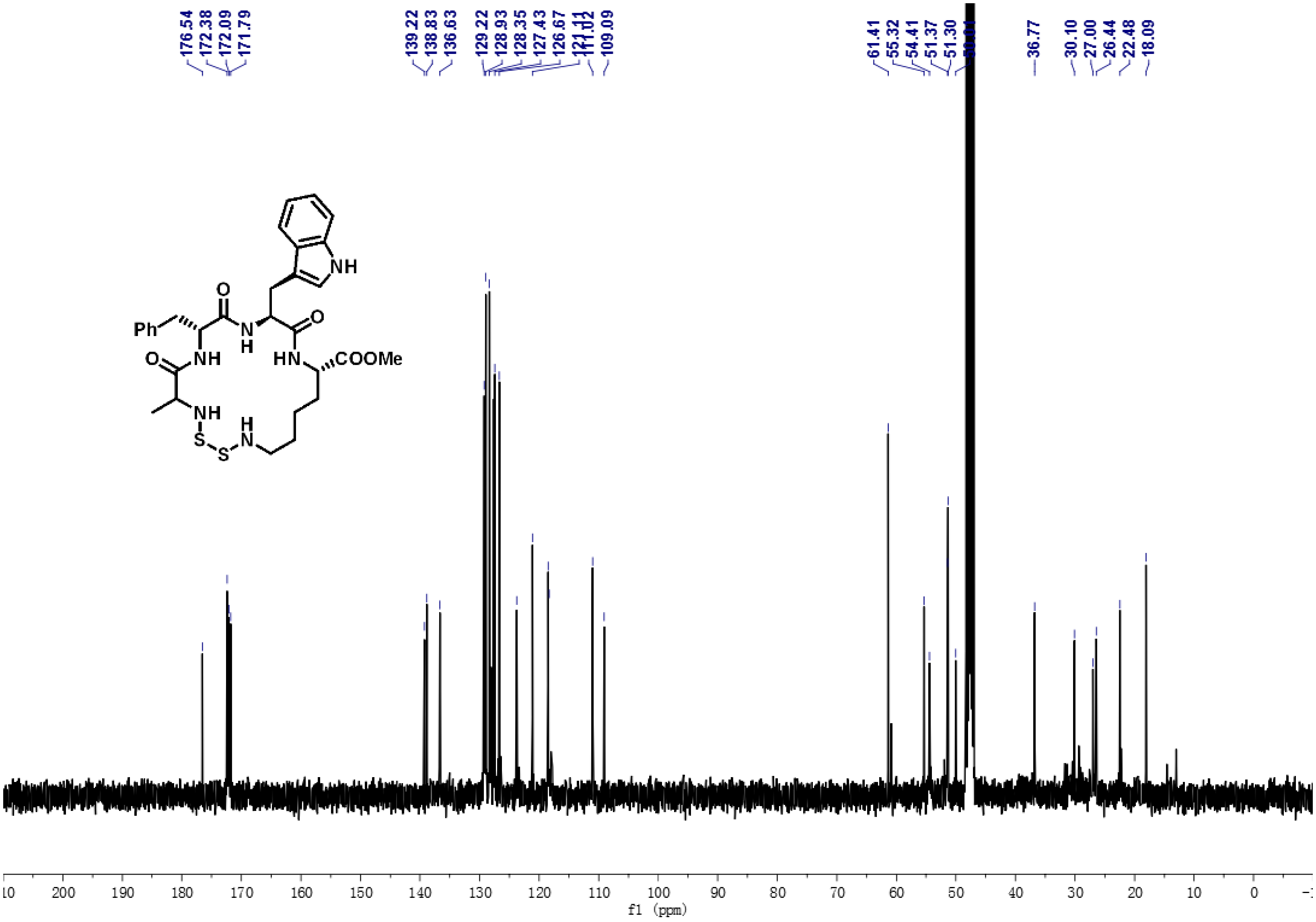

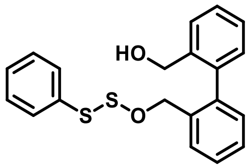

S1

^{1}H NMR (Acetone-d6)

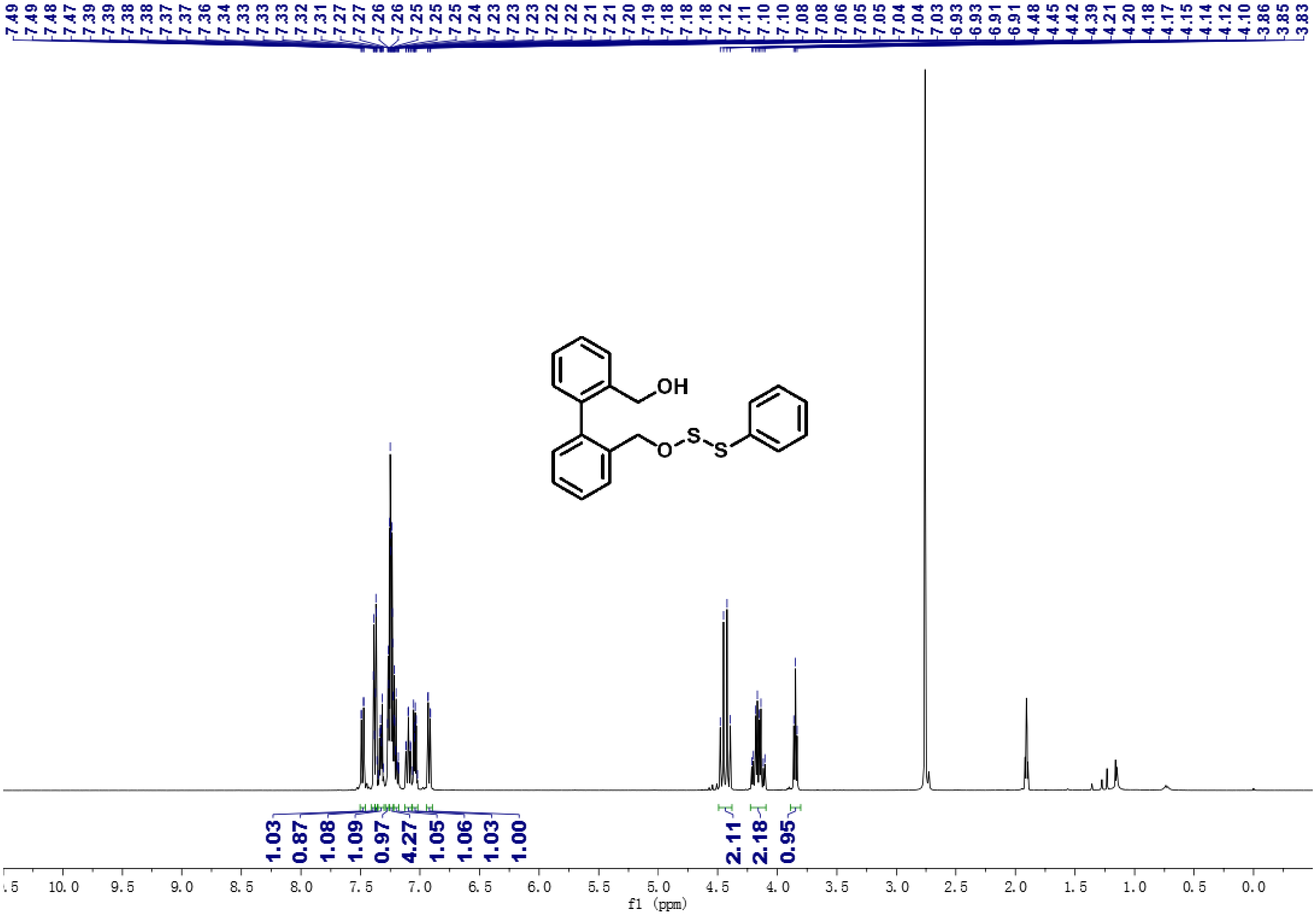

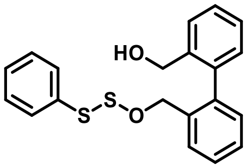

S1

^{13}C NMR (Acetone-d$_6$)

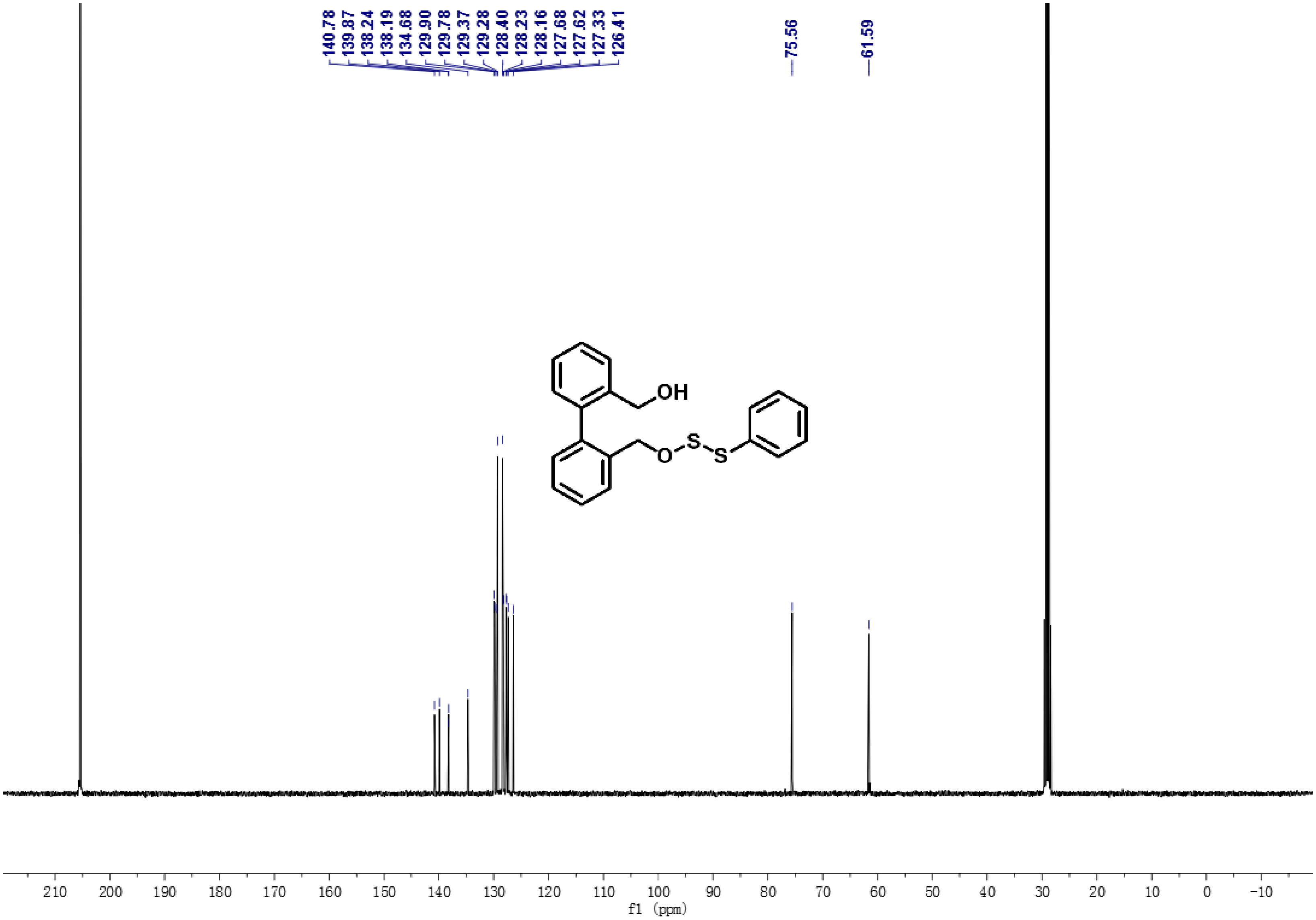

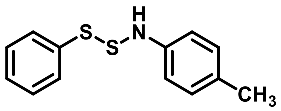

6a

^{1}H NMR (DMSO-d6)

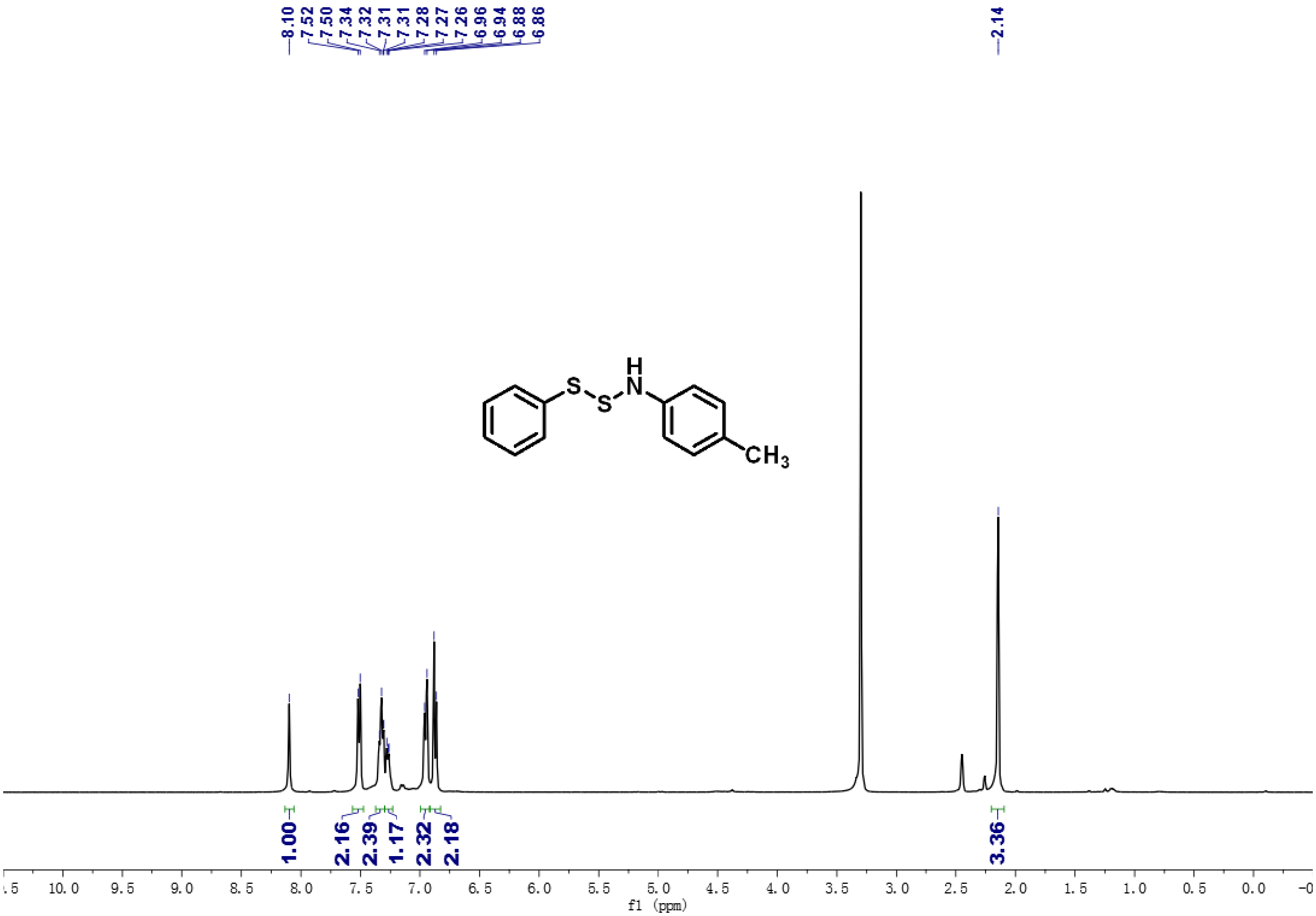

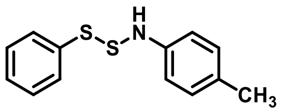

6a

^{13}C NMR (DMSO-d_6)

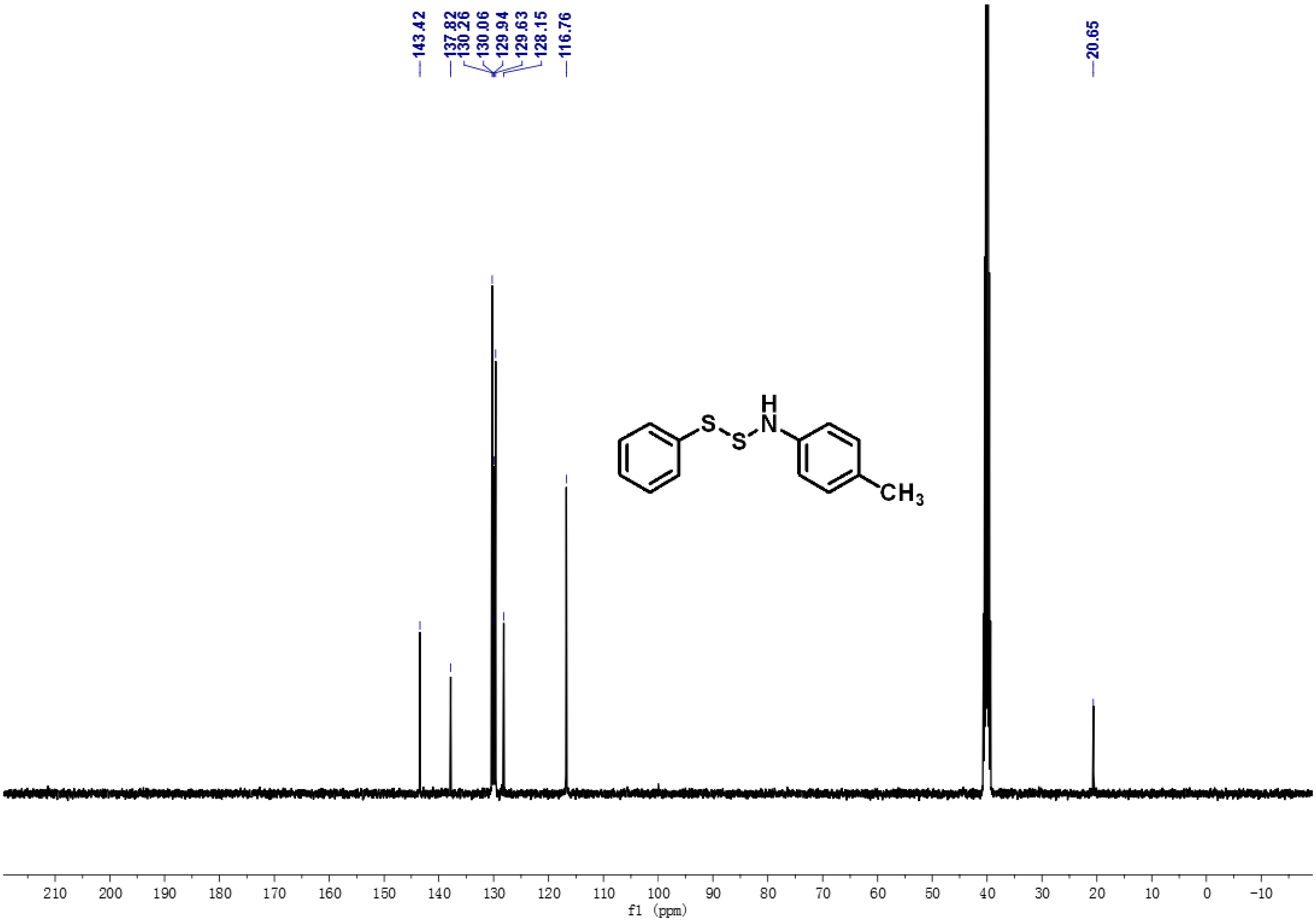

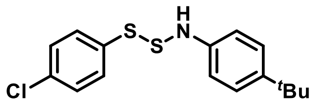

6b

^{1}H NMR ($CDCl_3$)

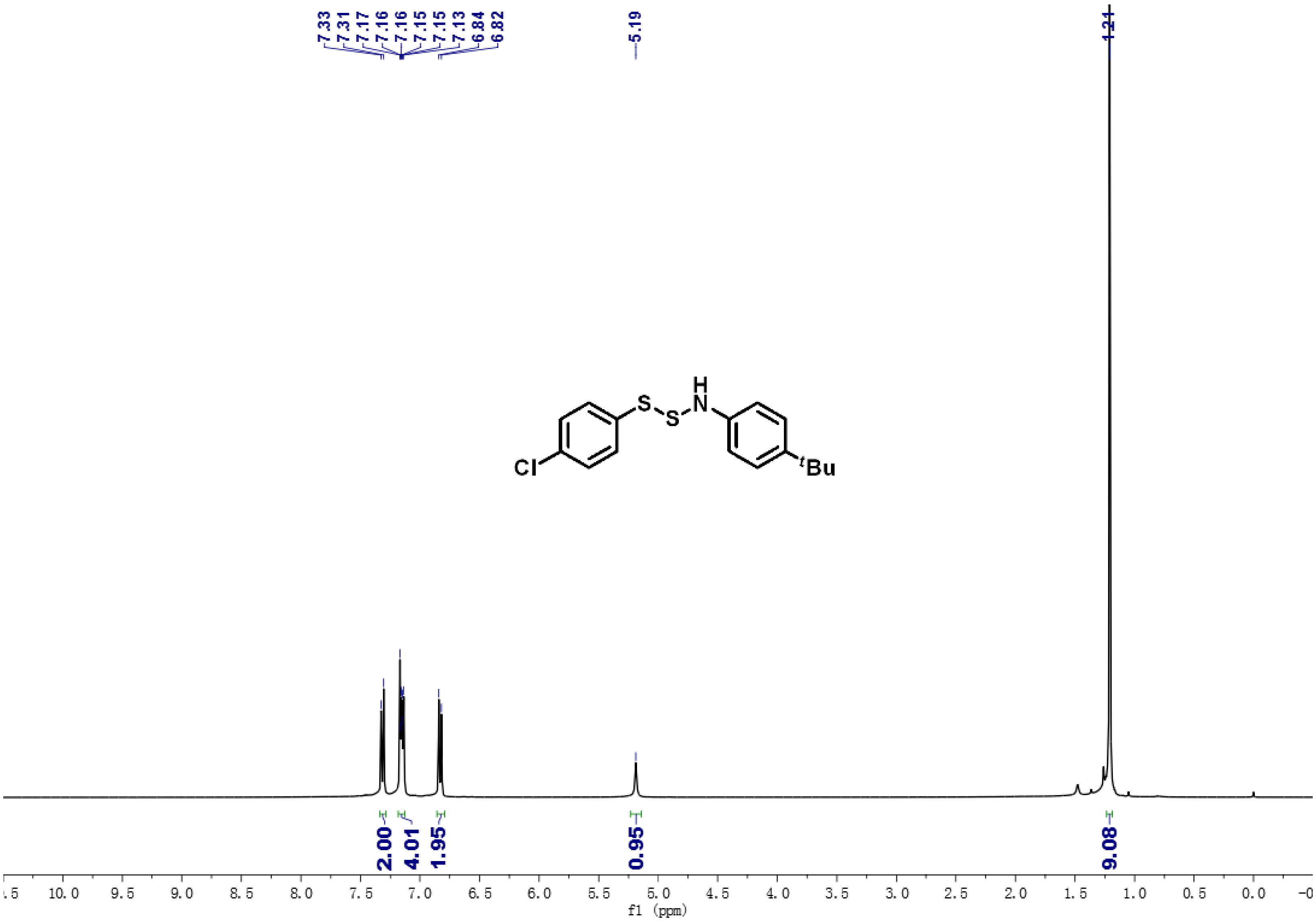

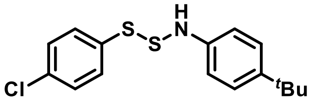

6b

^{13}C NMR ($CDCl_3$)

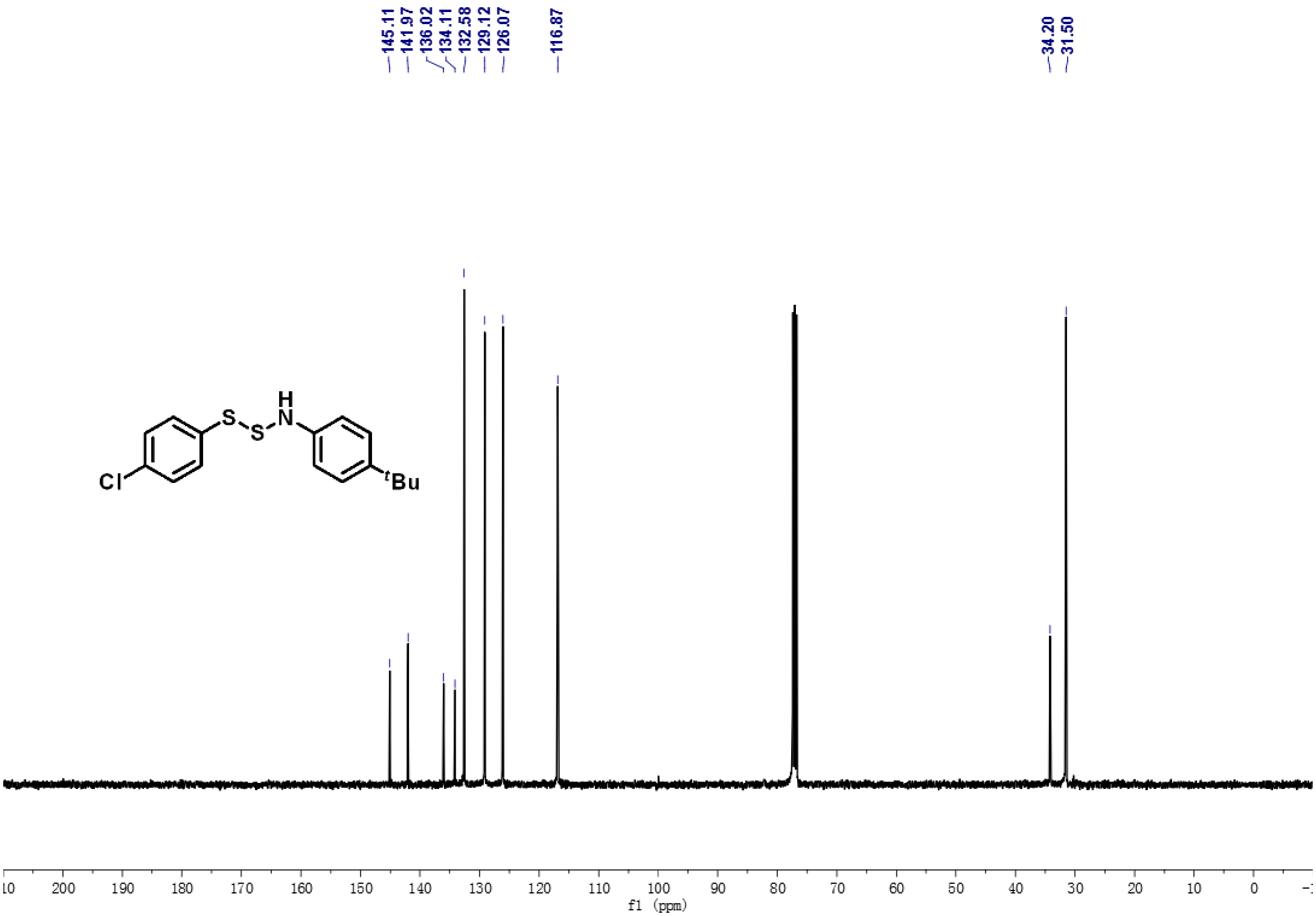

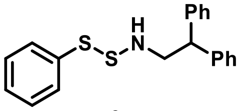

6c

^{1}H NMR (DMSO-d6)

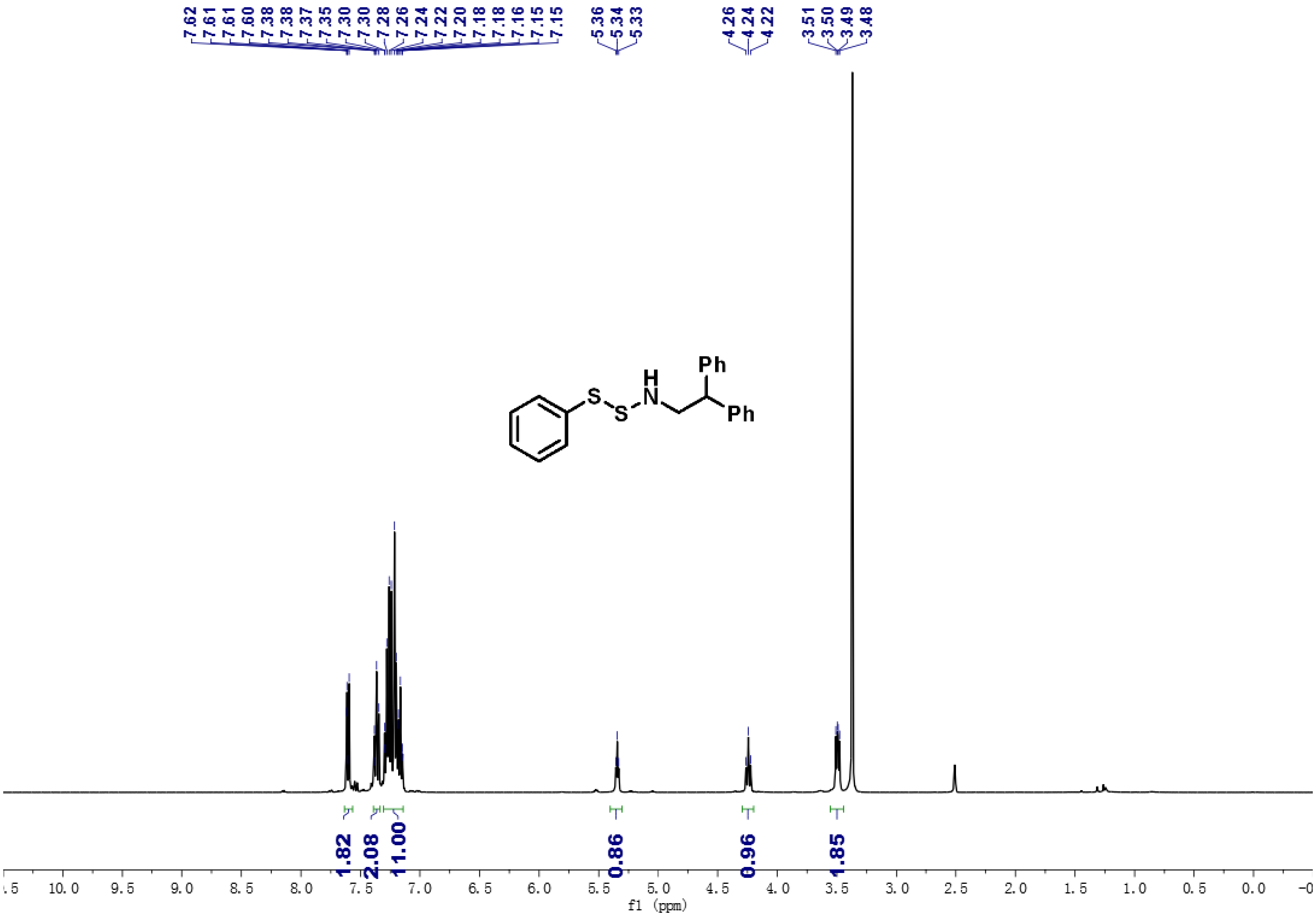

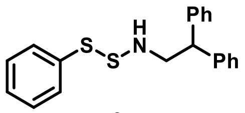

6c

^{13}C NMR ($CDCl_3$)

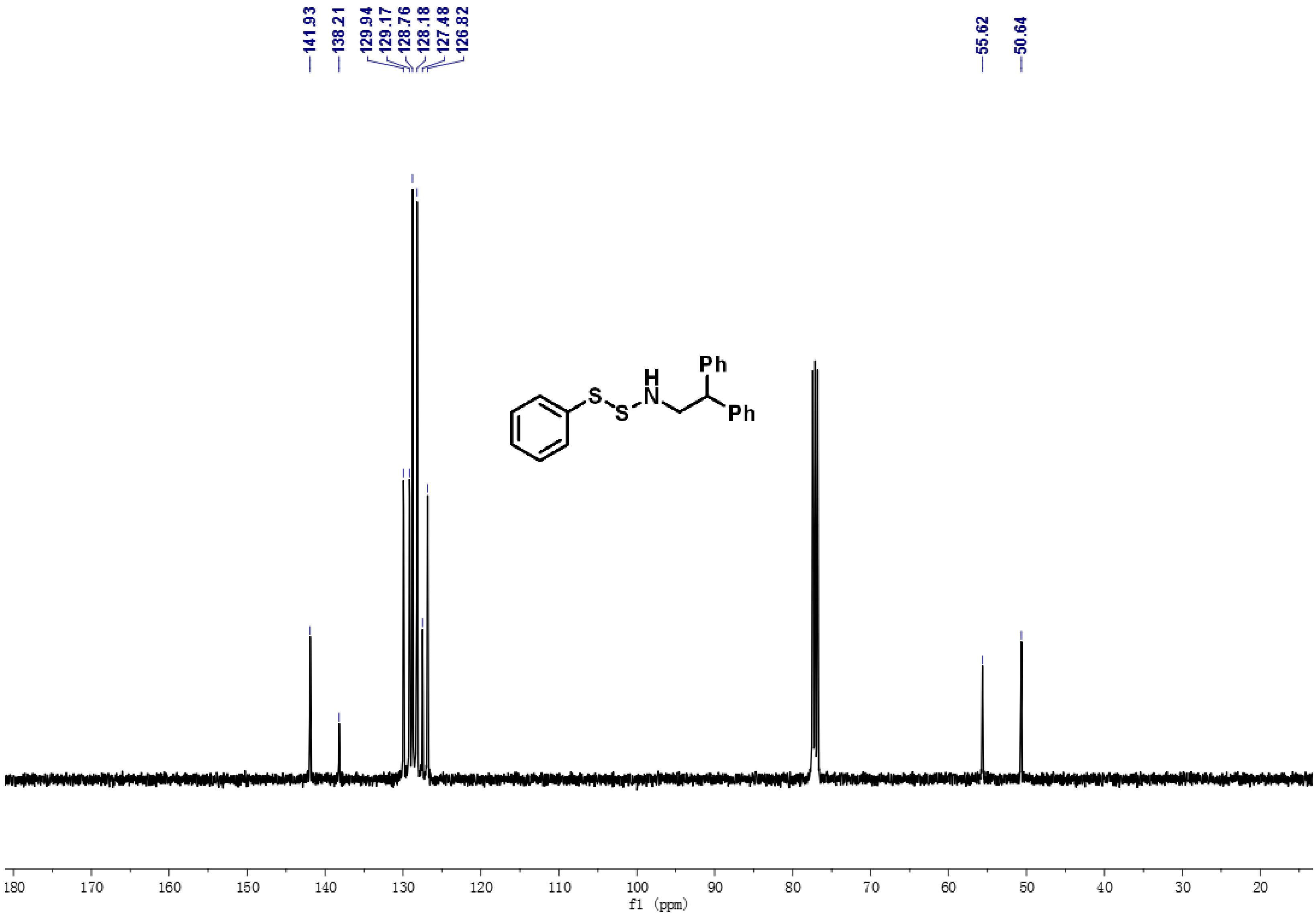

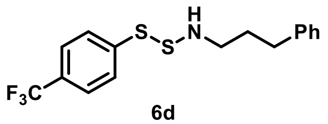

6d

^{1}H NMR ($CDCl_3$)

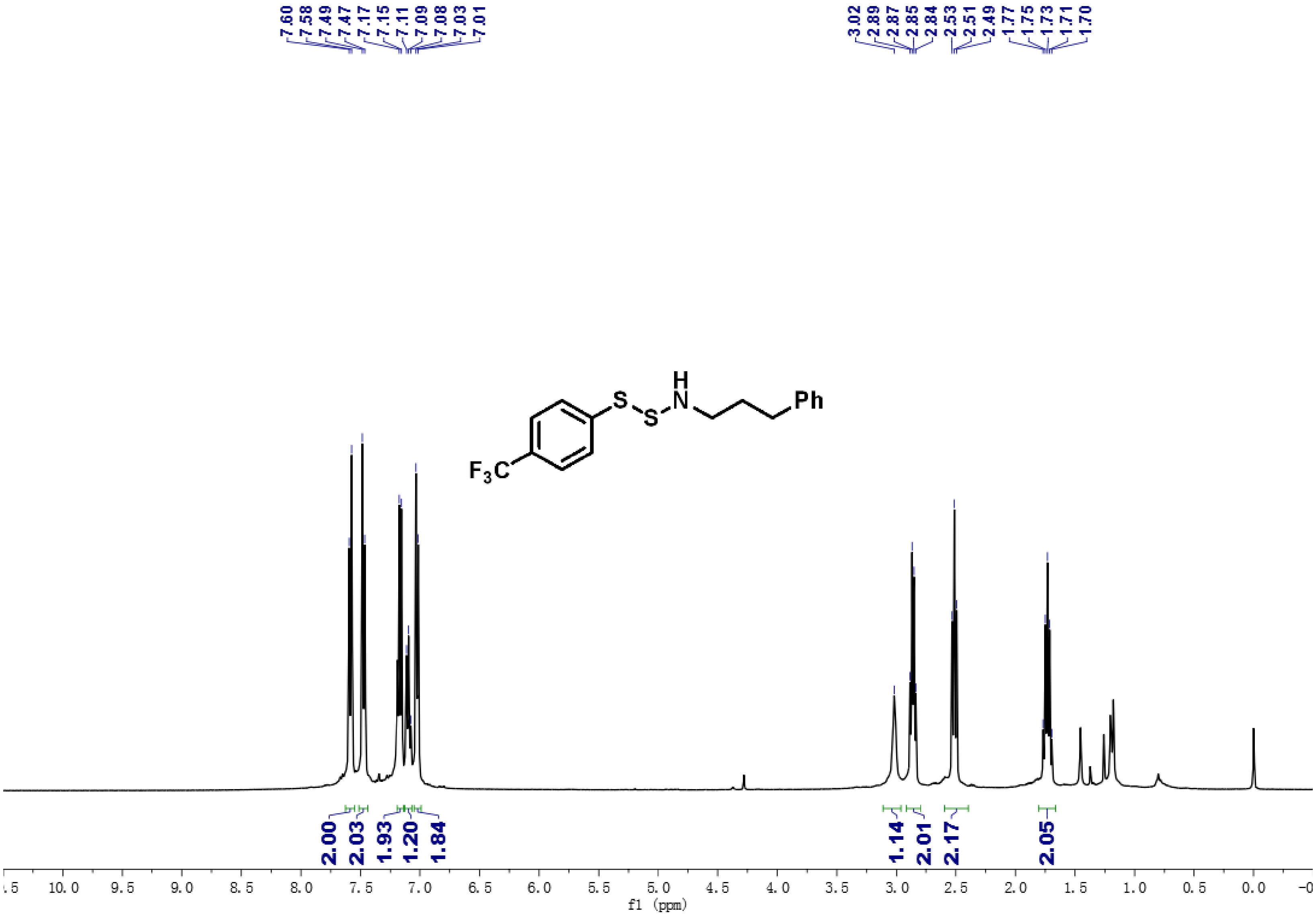

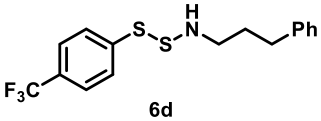

6d

^{19}F NMR ($CDCl_3$)

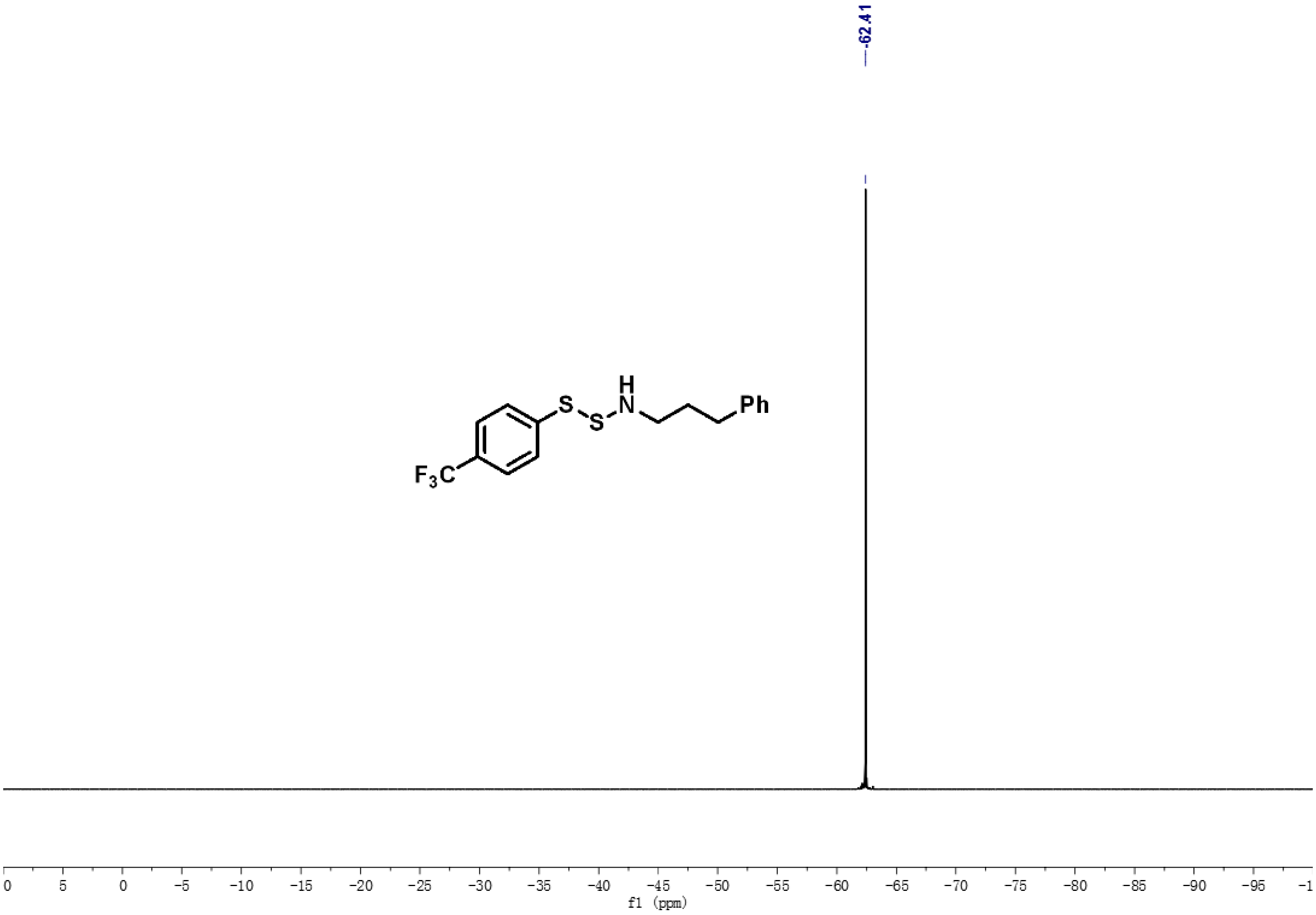

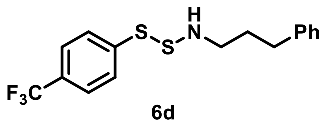

6d

^{13}C NMR ($CDCl_3$)

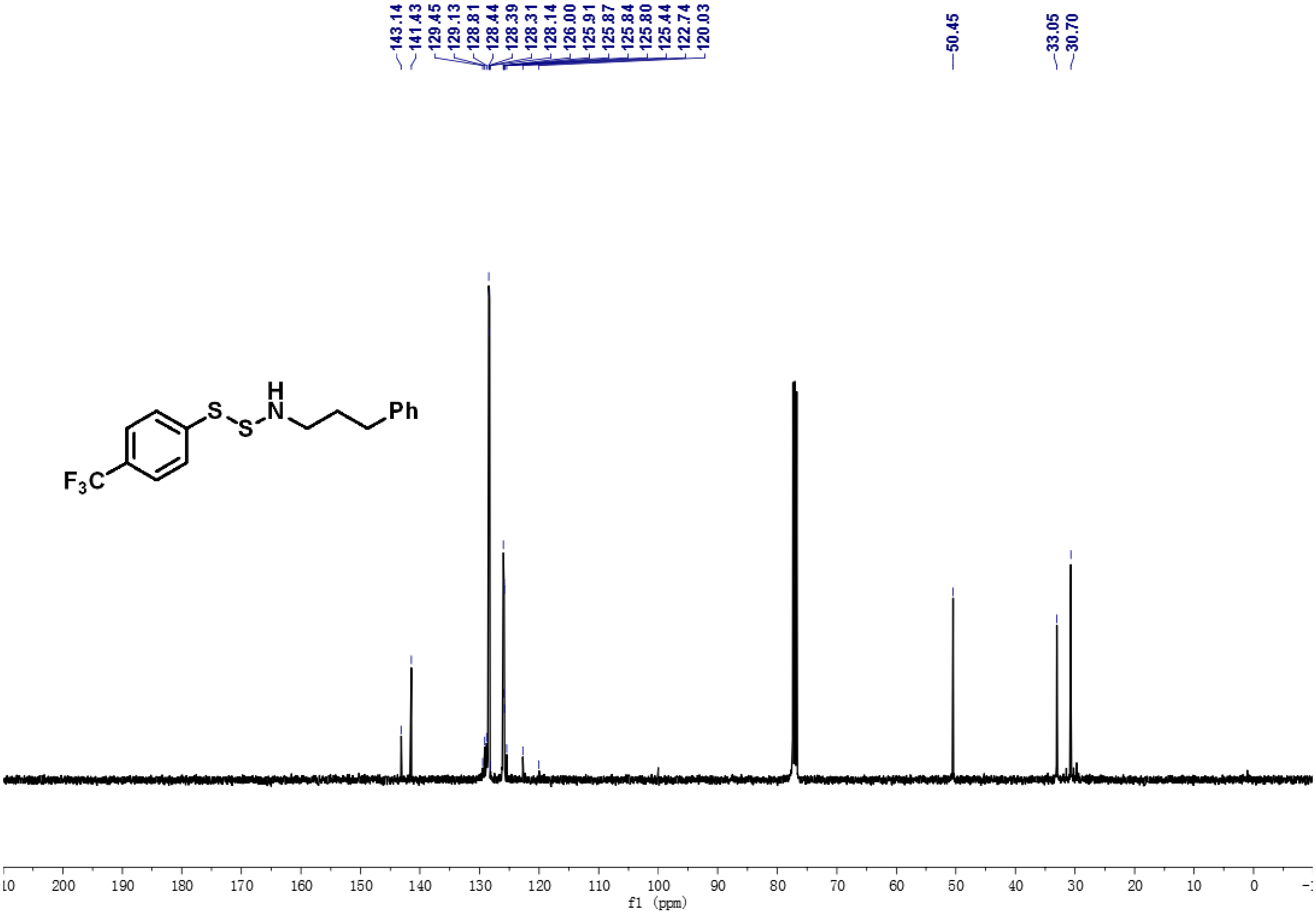

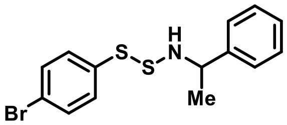

6e

^{1}H NMR ($CDCl_3$)

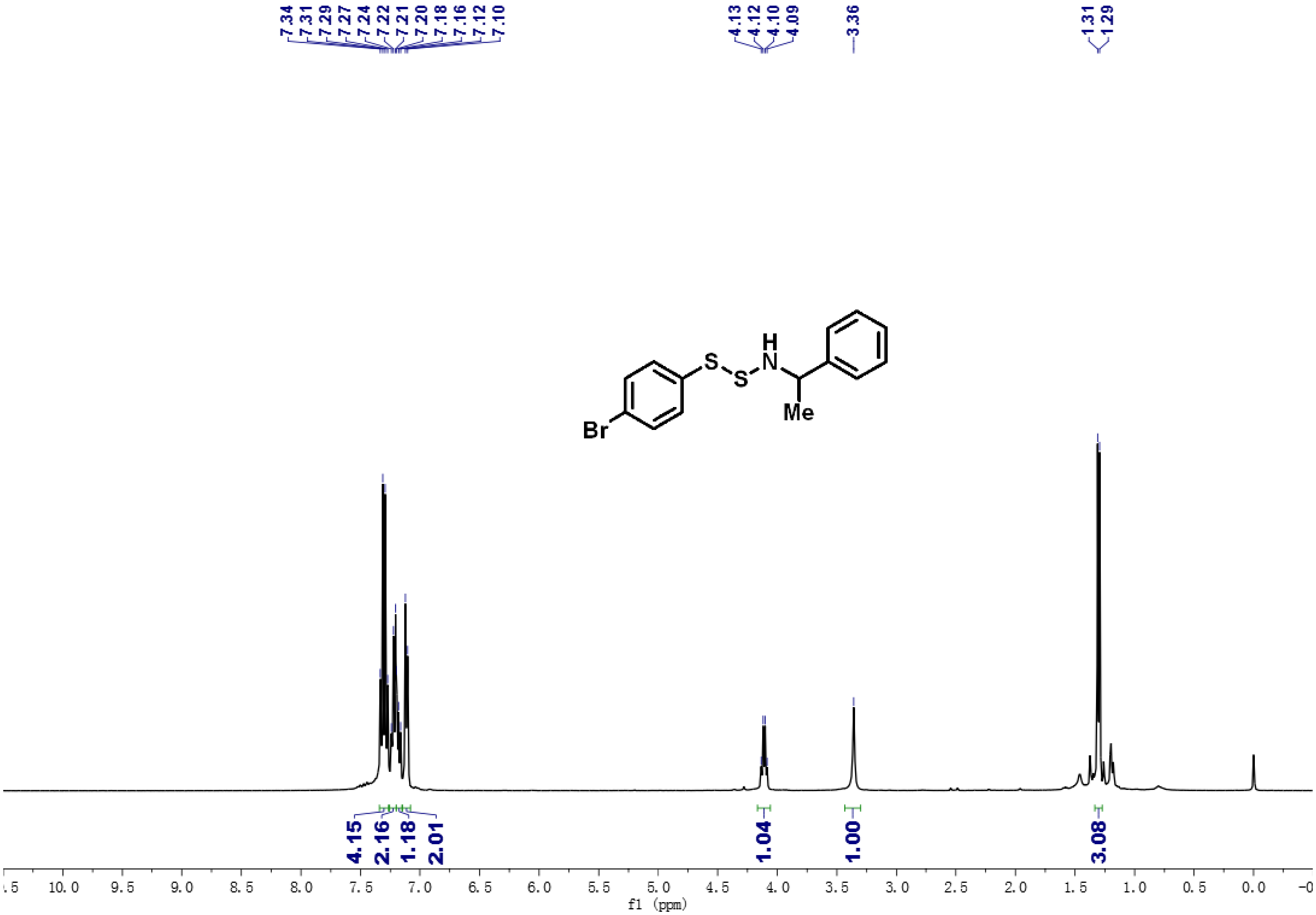

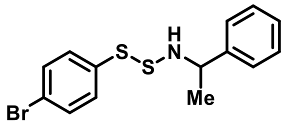

6e

^{13}C NMR ($CDCl_3$)

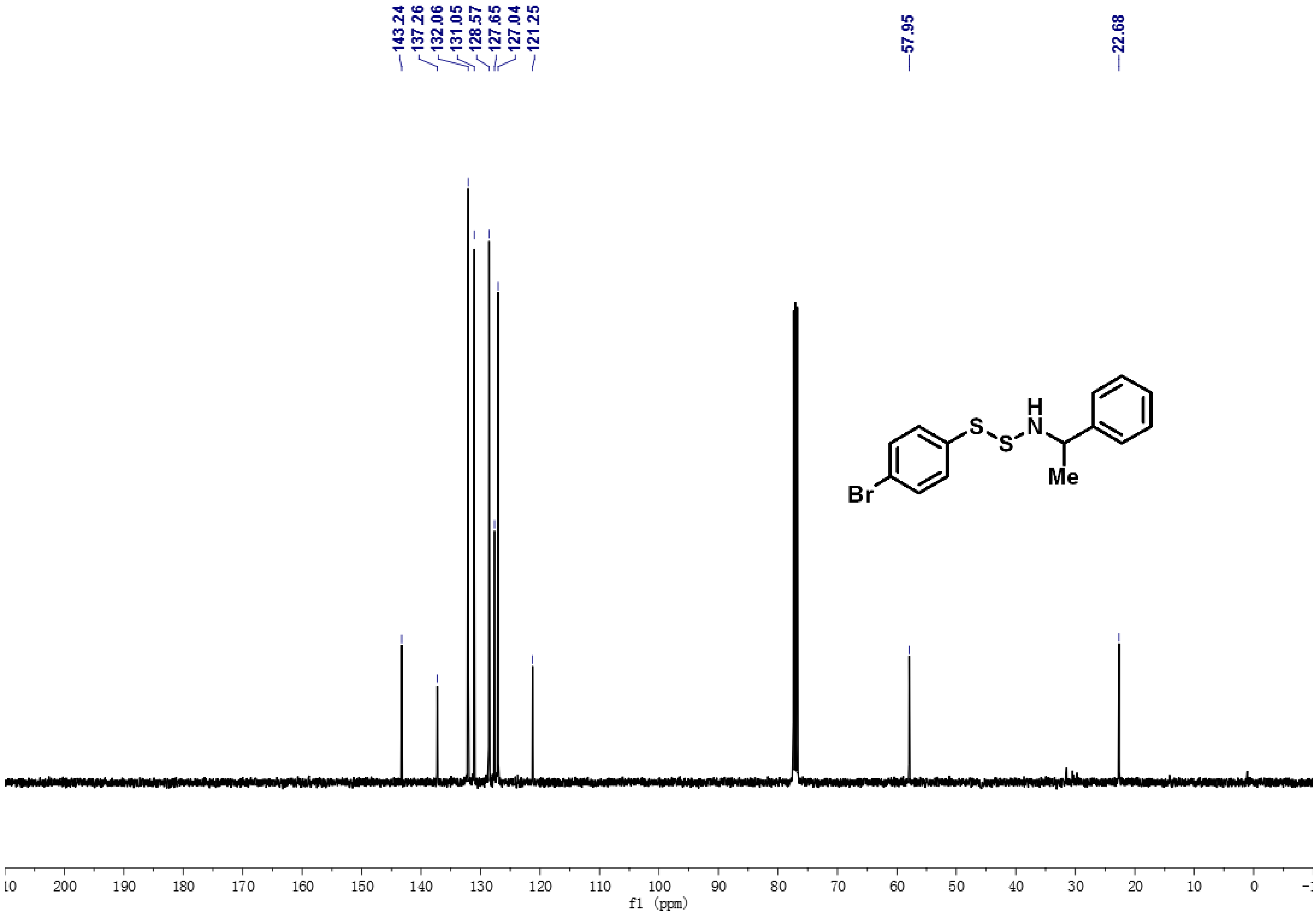

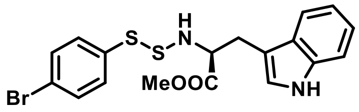

6f

^{1}H NMR ($CDCl_3$)

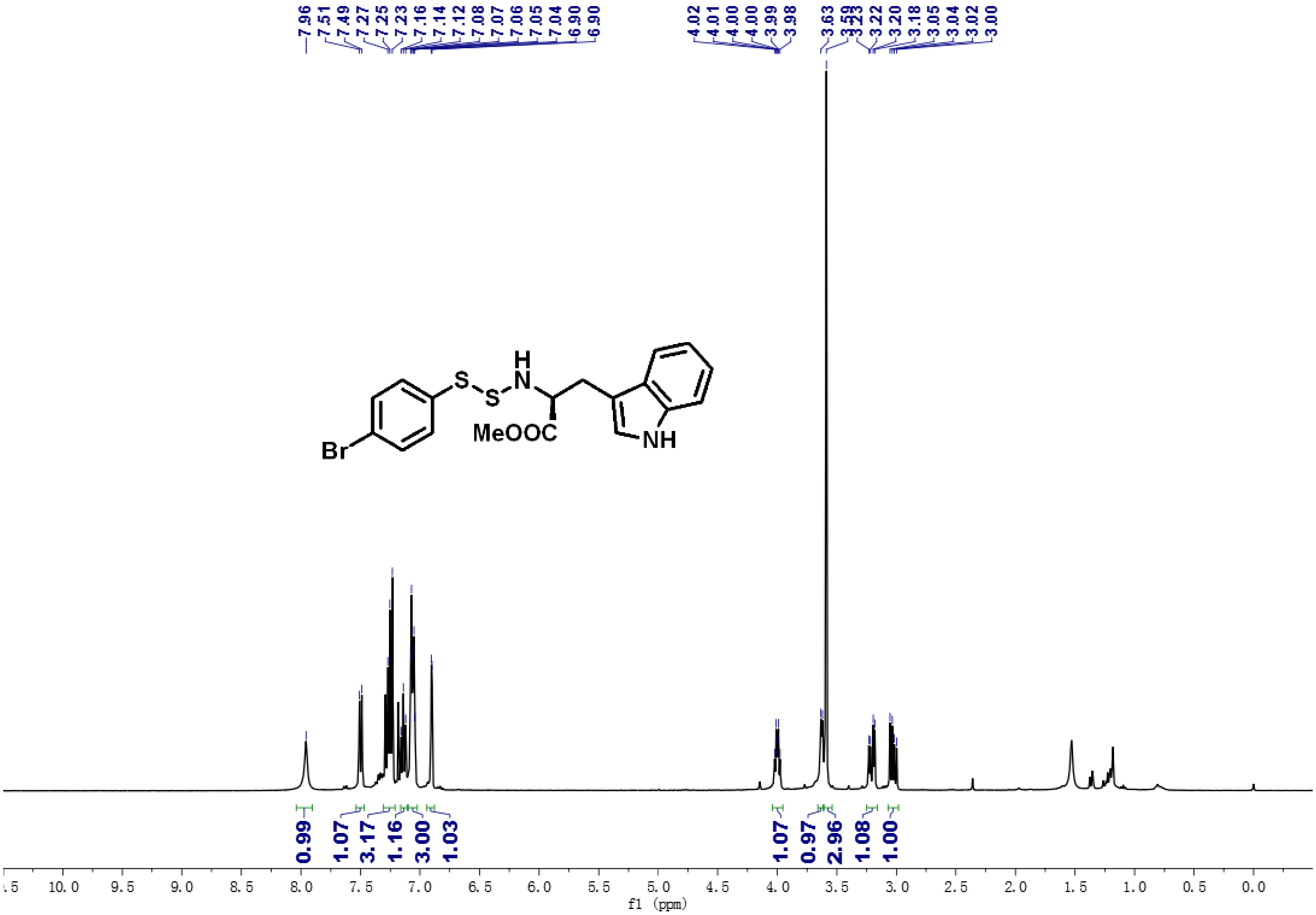

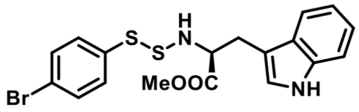

6f

^{13}C NMR ($CDCl_3$)

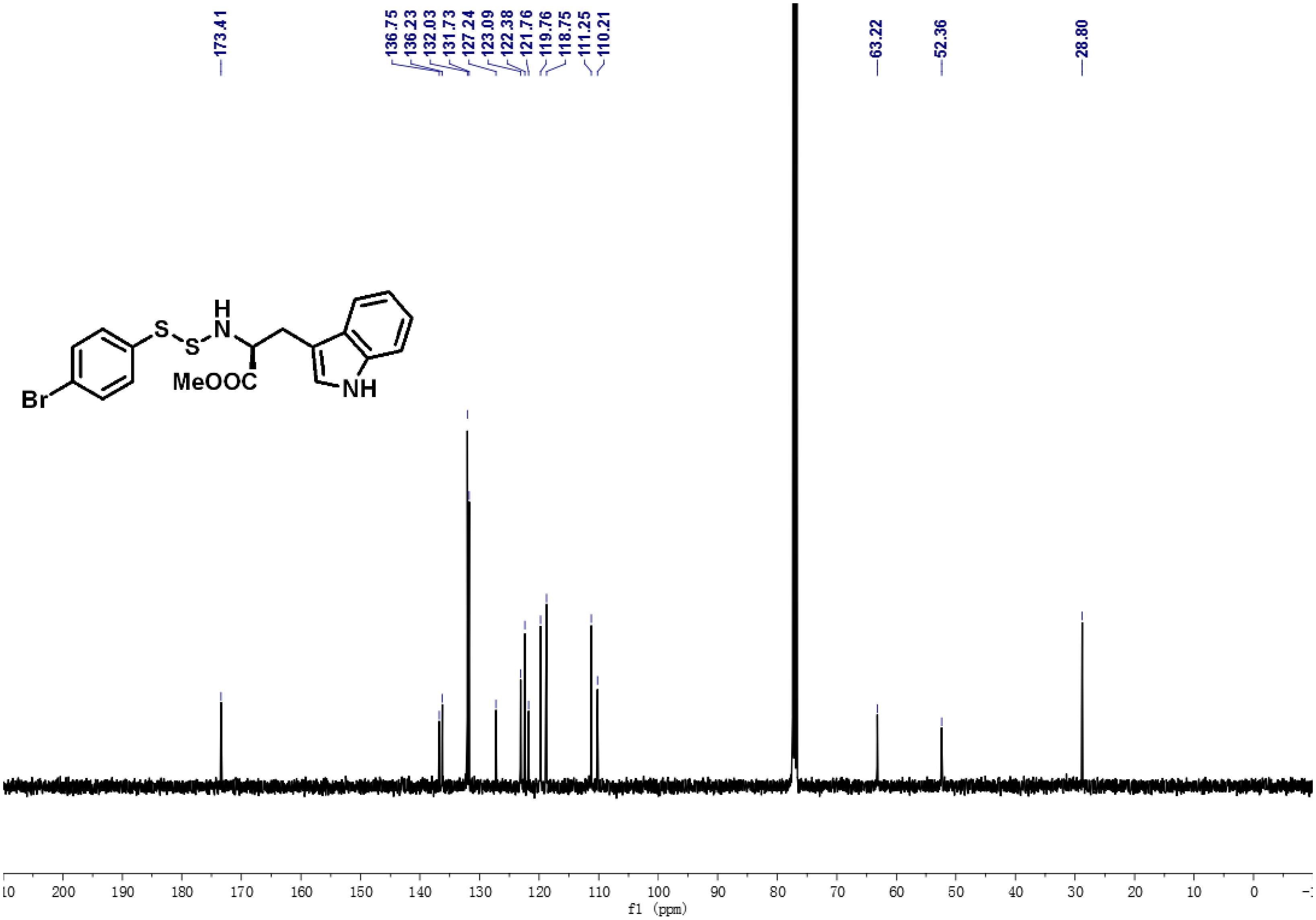

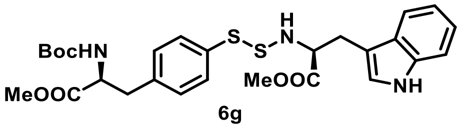

6g

^{1}H NMR ($CDCl_3$)

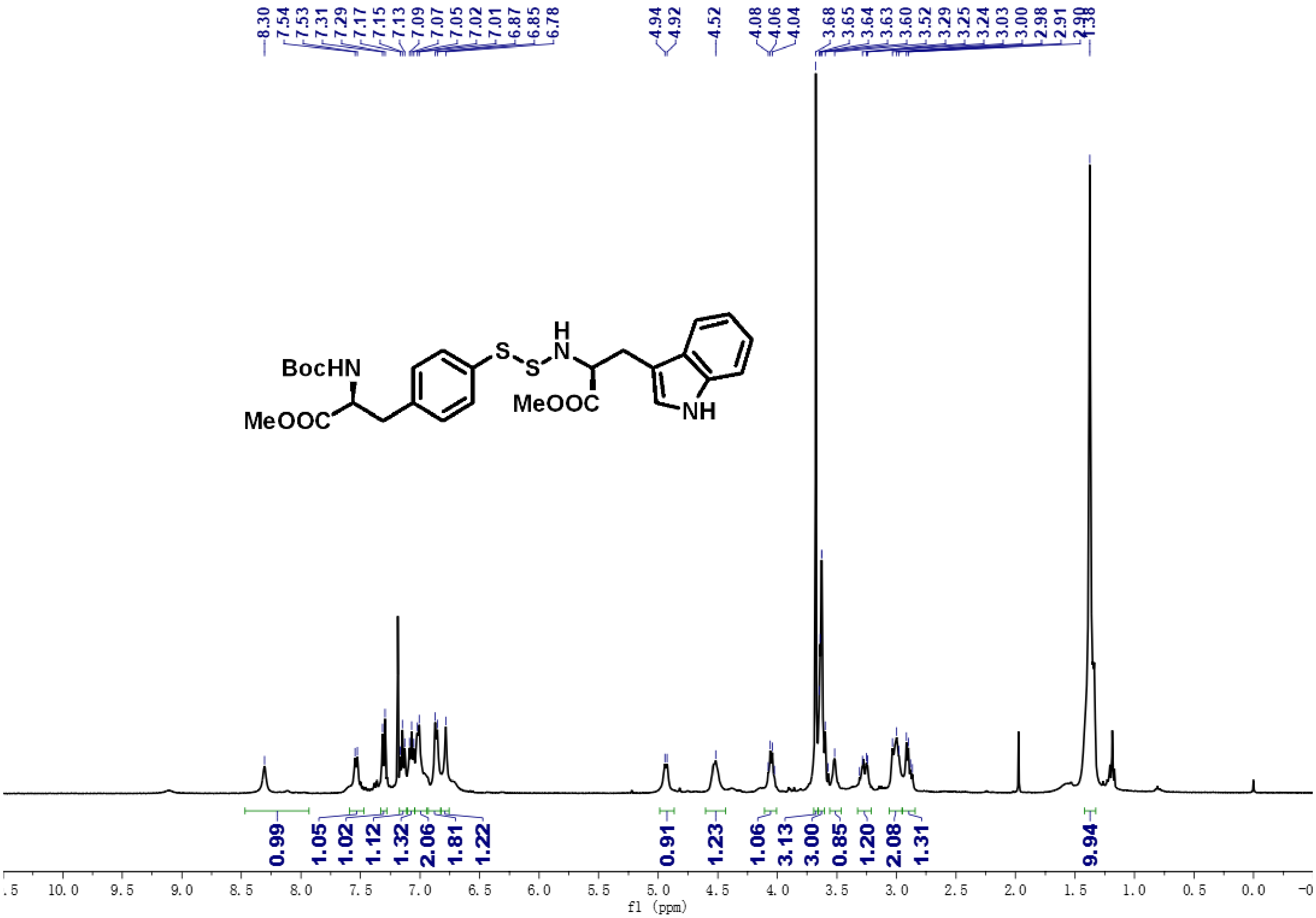

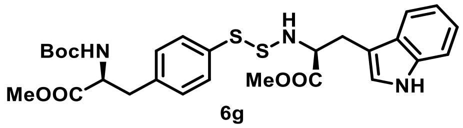

6g

^{13}C NMR ($CDCl_3$)

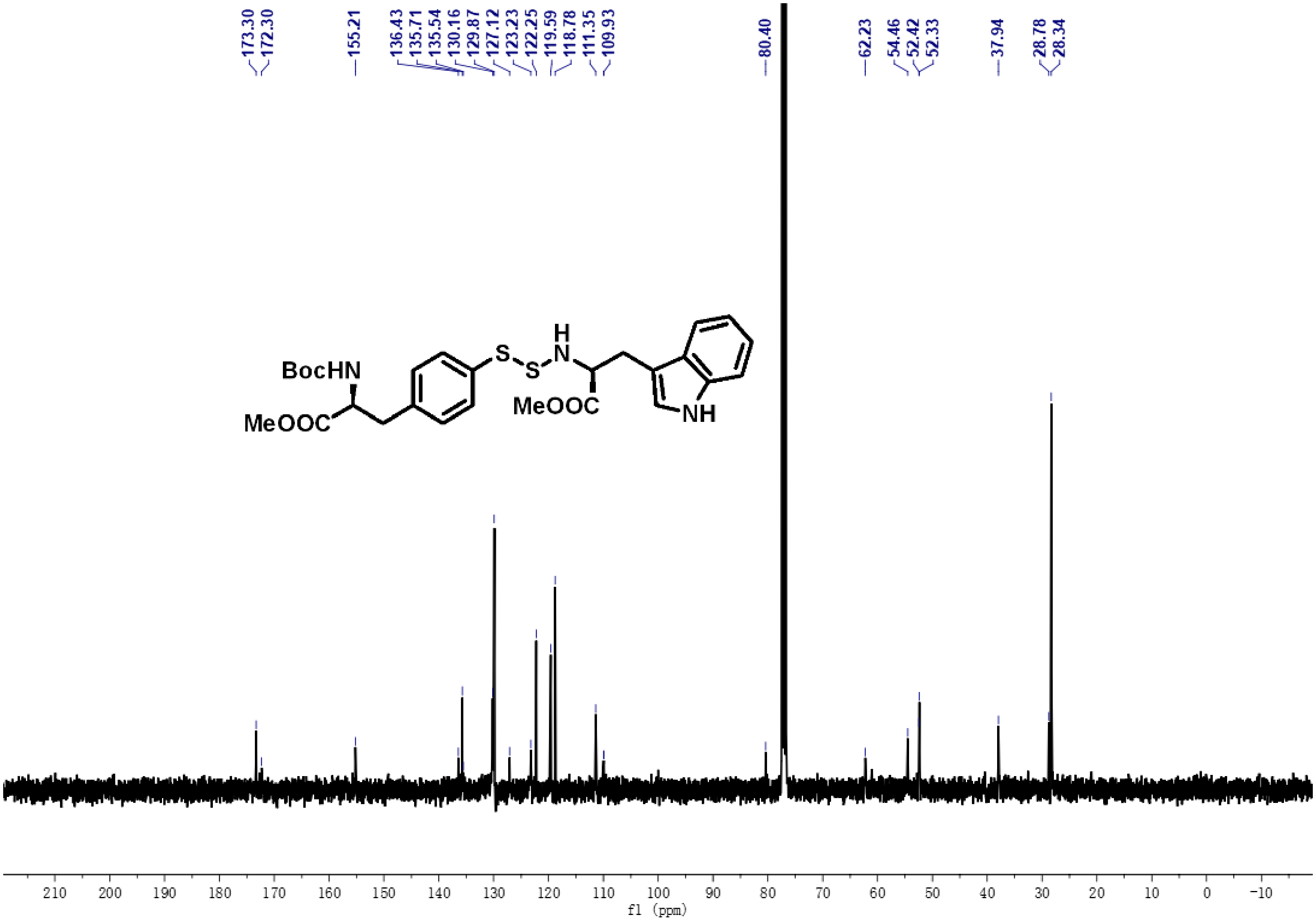

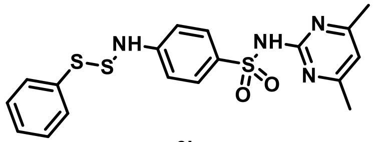

6h

^{1}H NMR ($CDCl_3$)

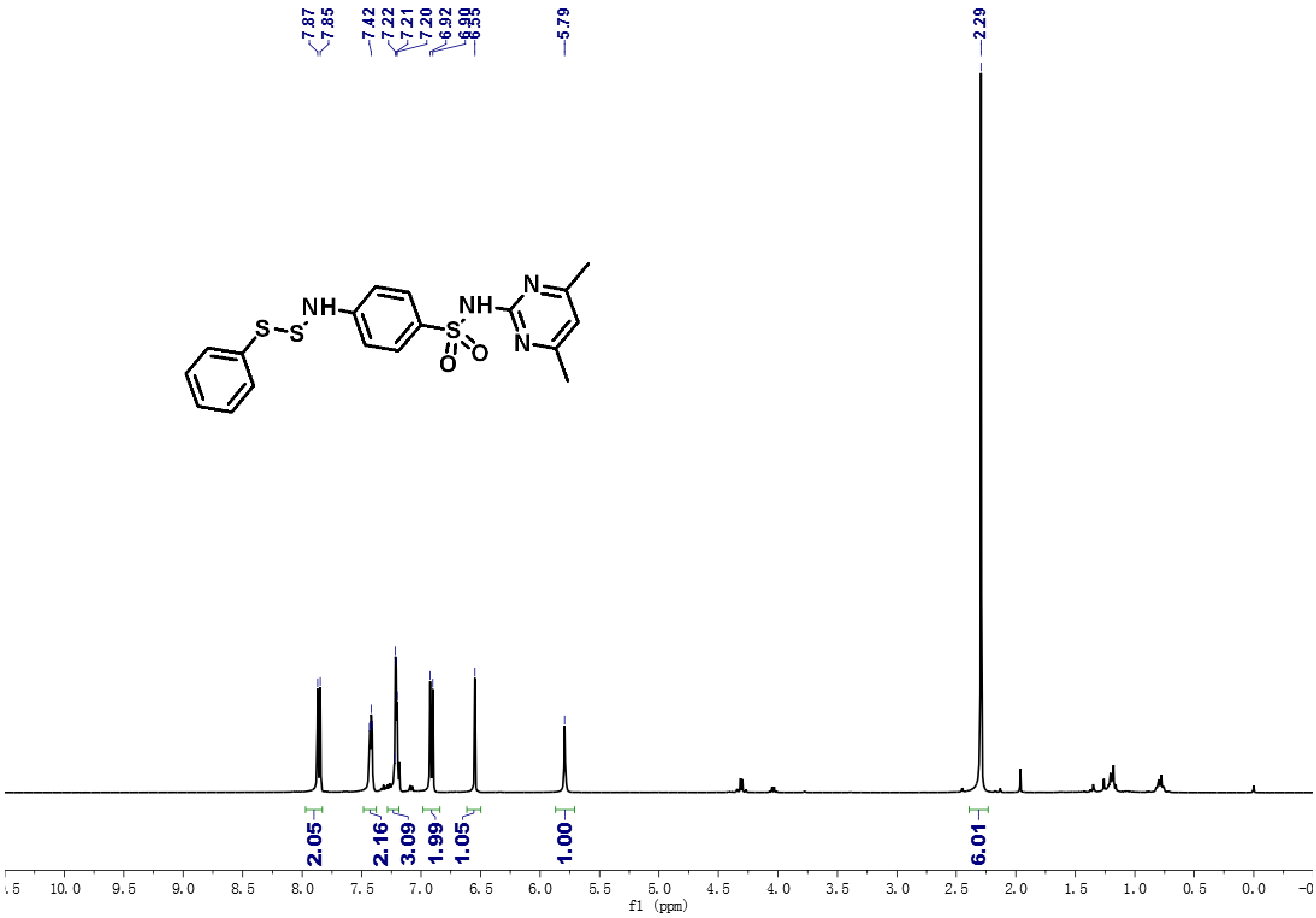

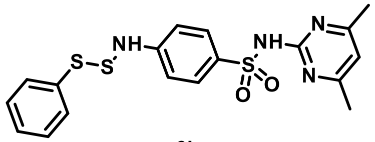

6h

^{13}C NMR ($CDCl_3$)

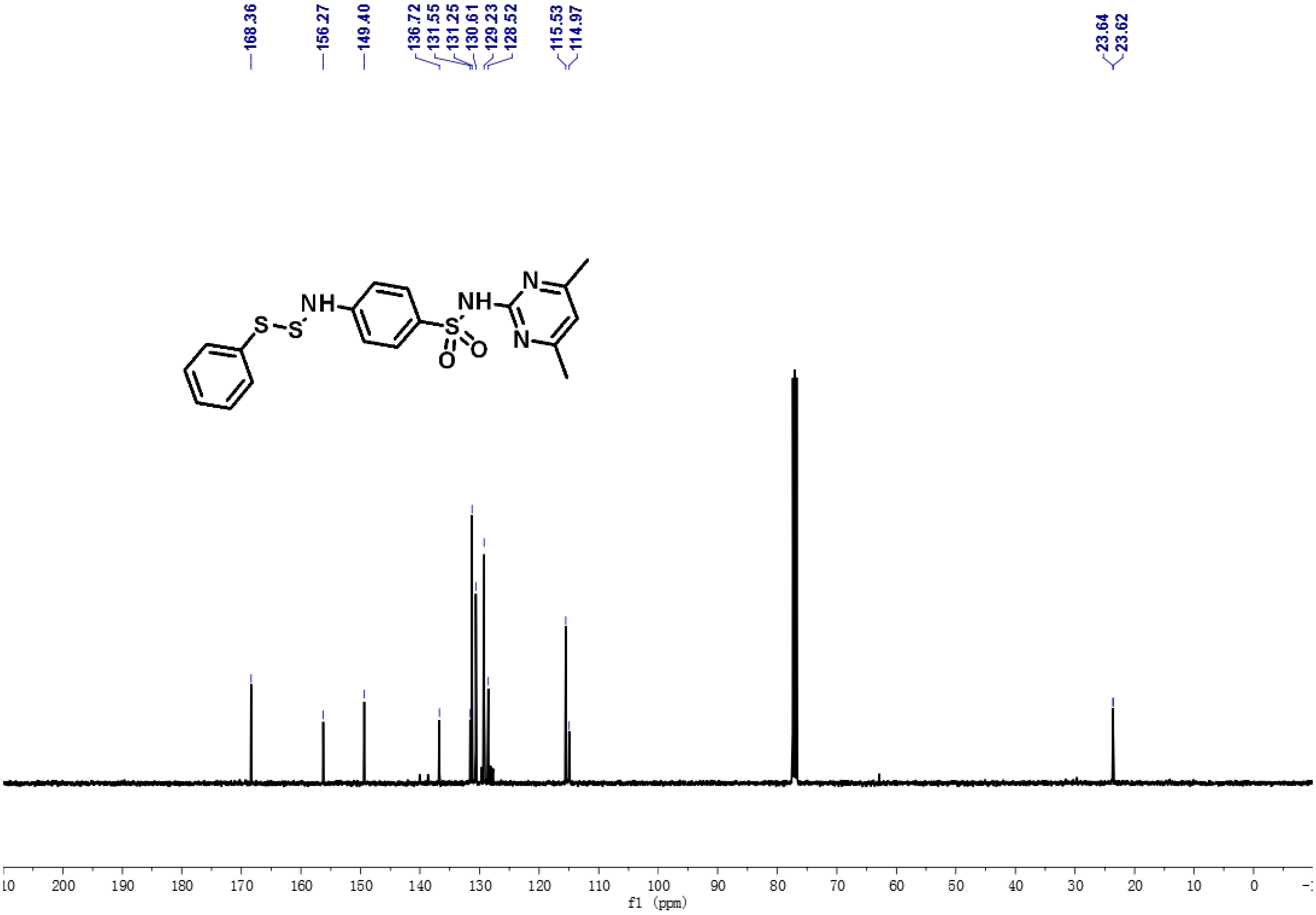

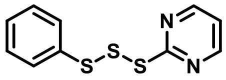

7a

^{1}H NMR ($CDCl_3$), ^{13}C NMR ($CDCl_3$)

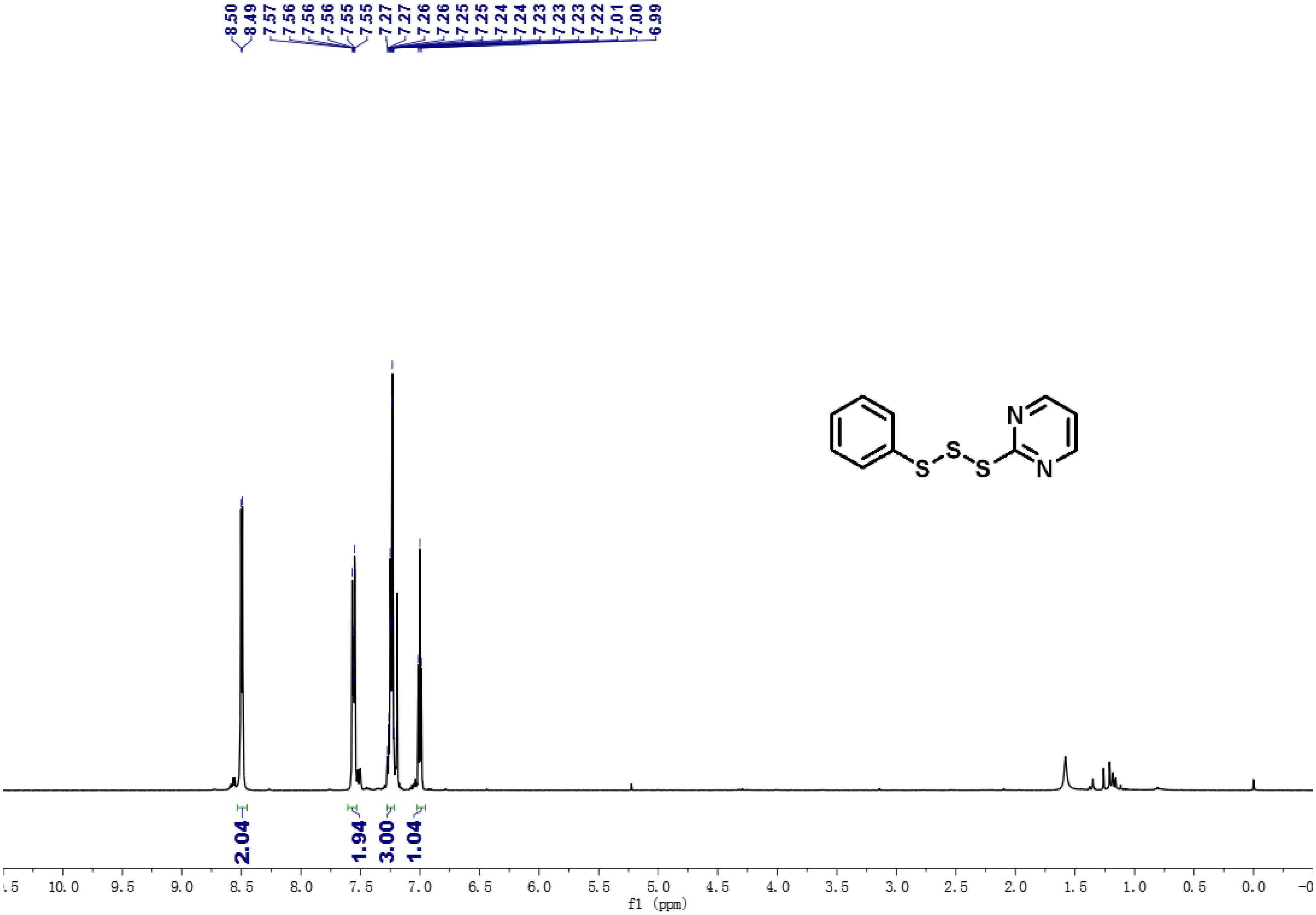

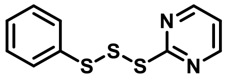

7a

^{13}C NMR ($CDCl_3$)

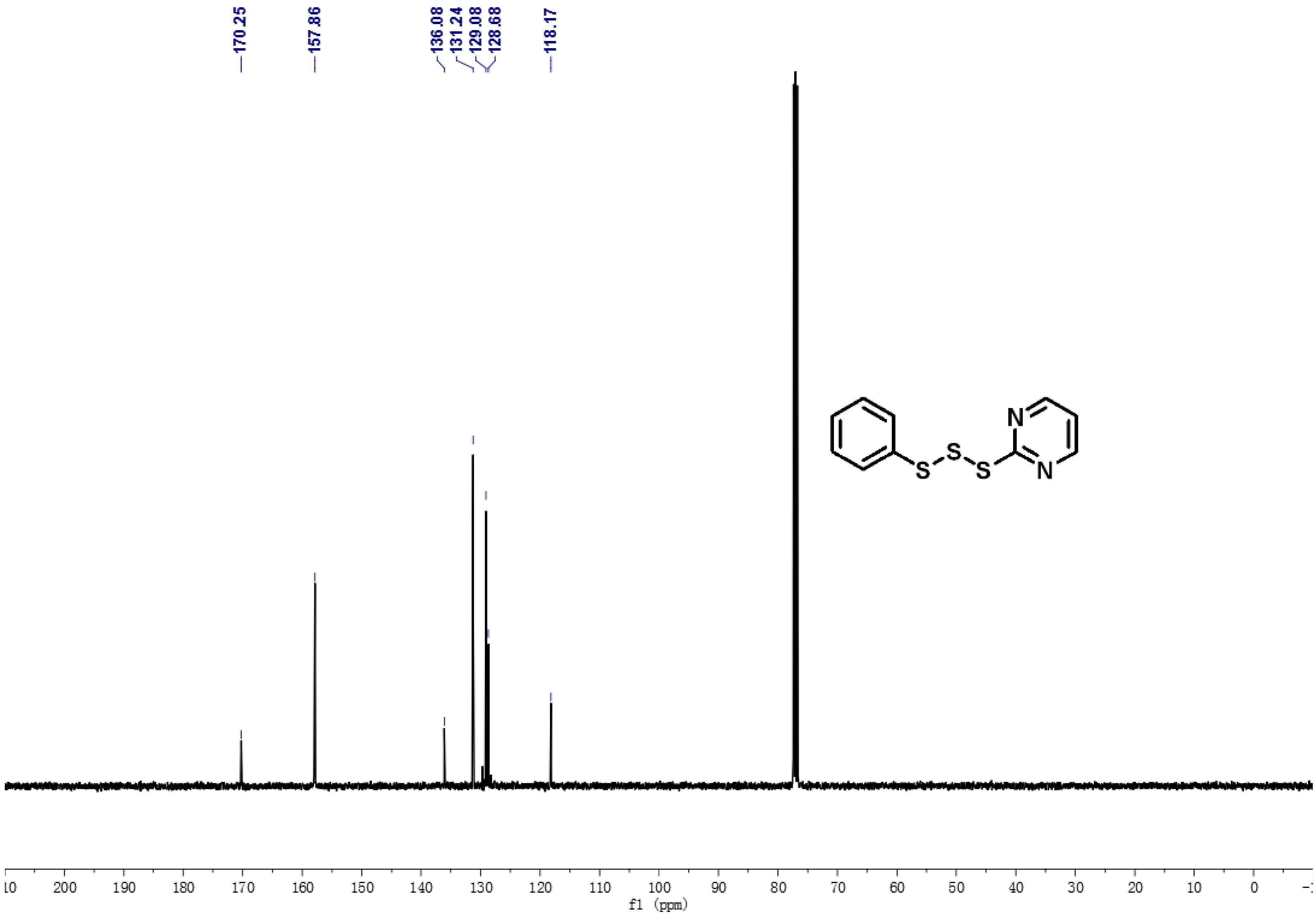

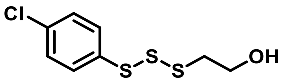

7b

^{1}H NMR ($CDCl_3$)

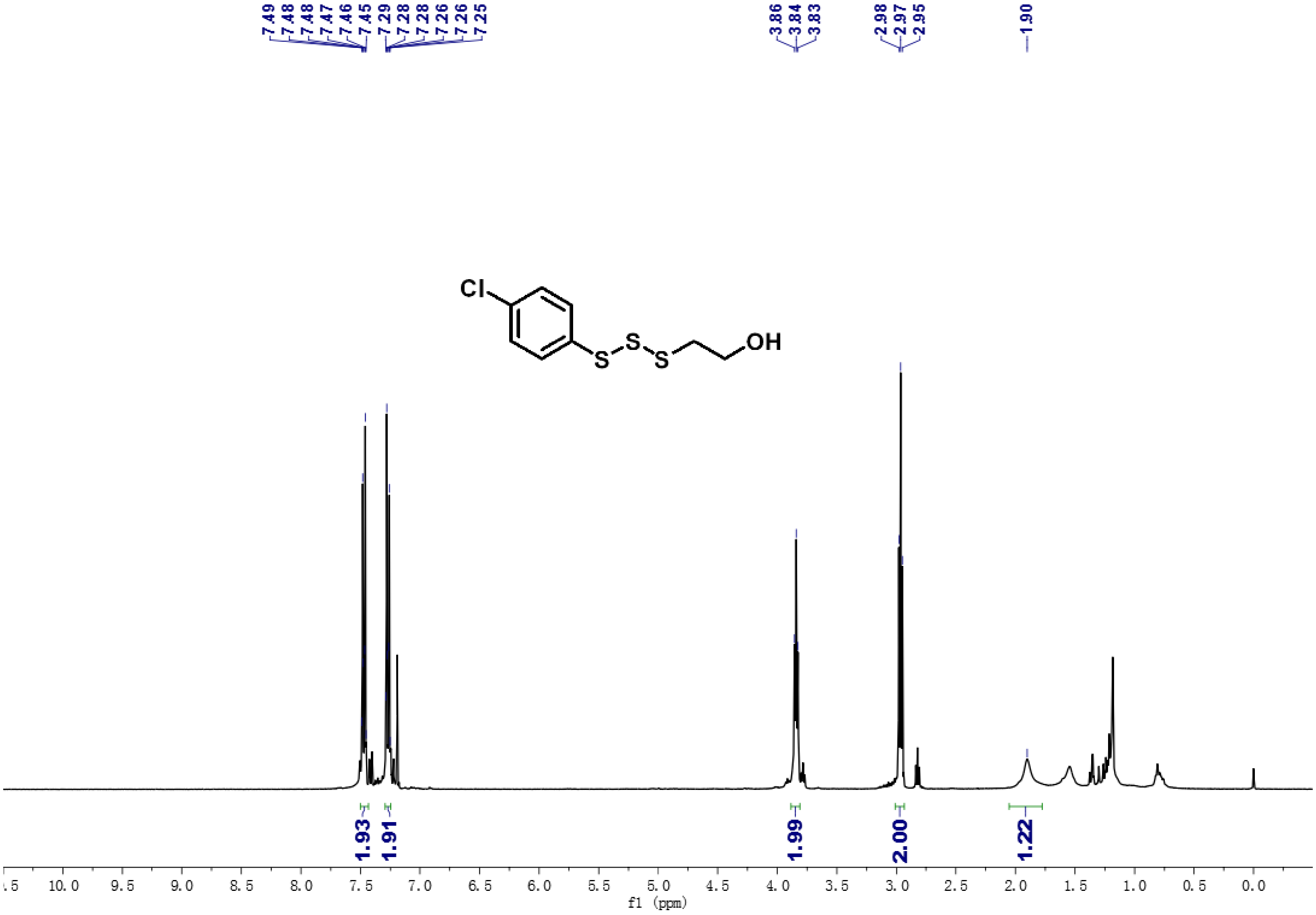

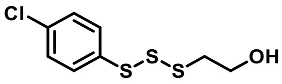

7b

^{13}C NMR ($CDCl_3$)

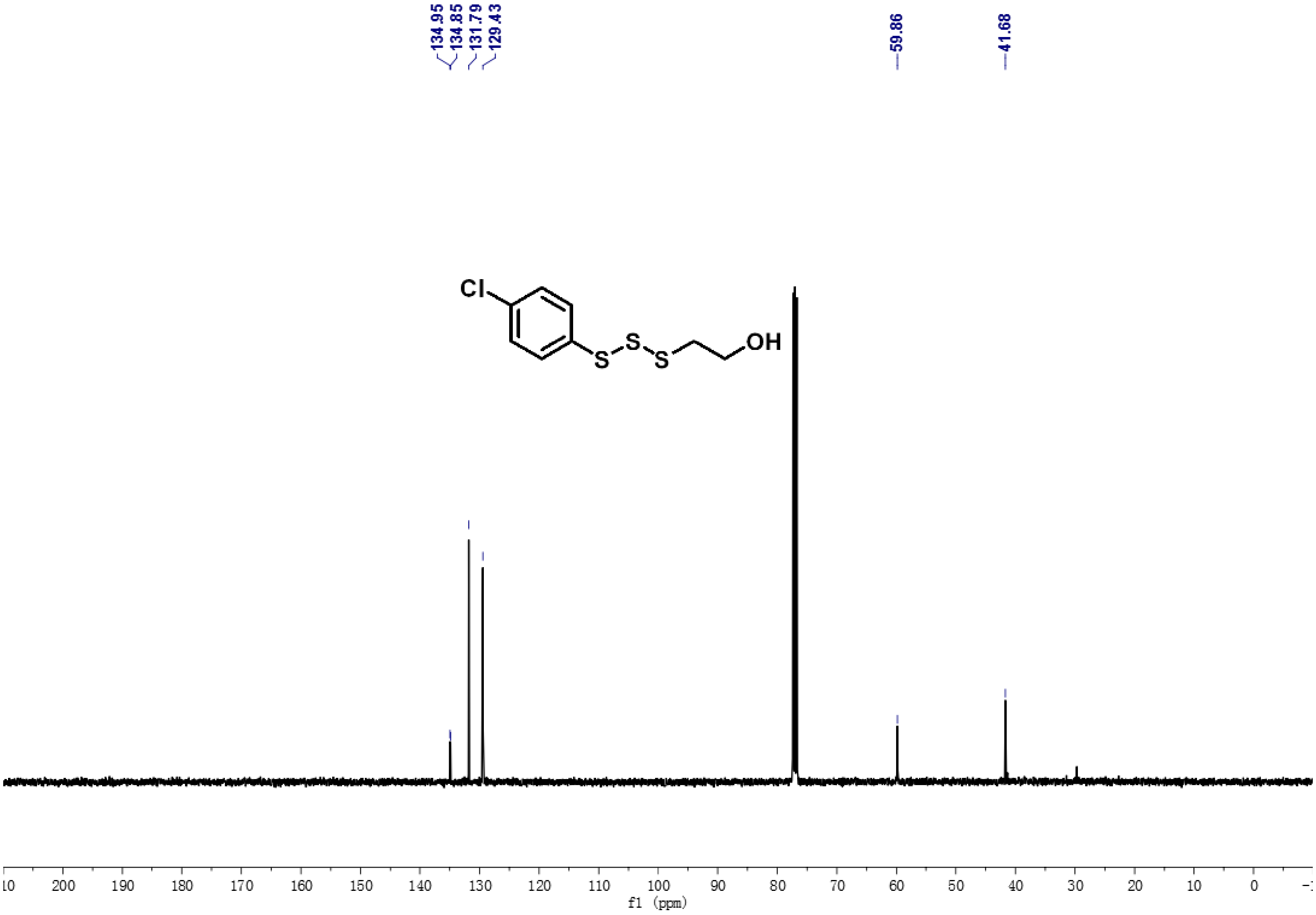

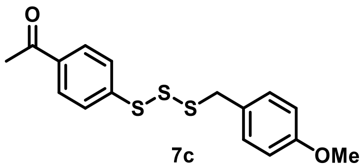

7c

^{1}H NMR (CDCl$_3$)

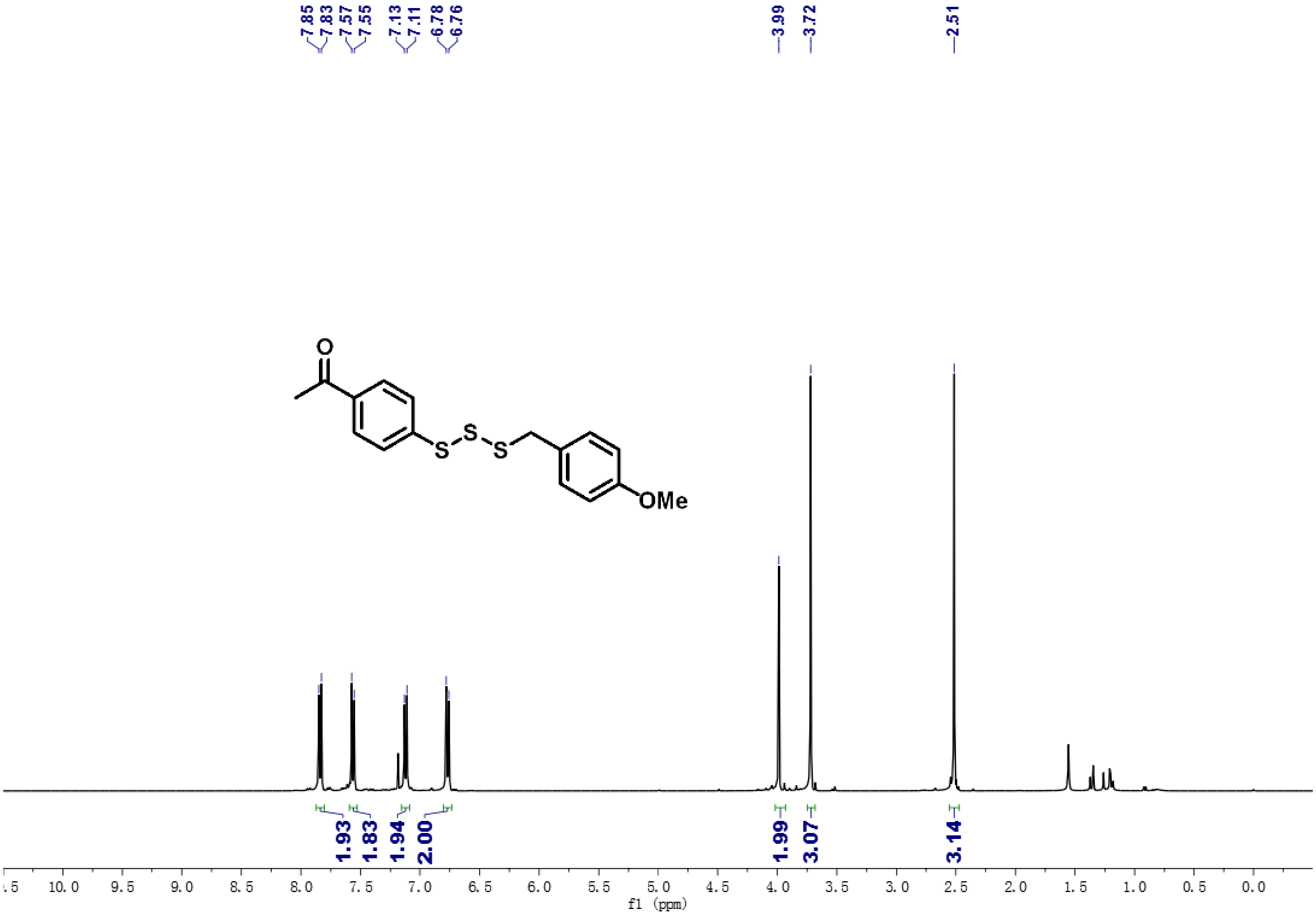

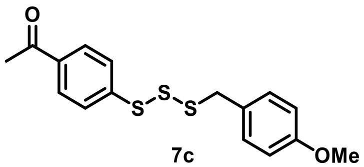

7c

^{13}C NMR ($CDCl_3$)

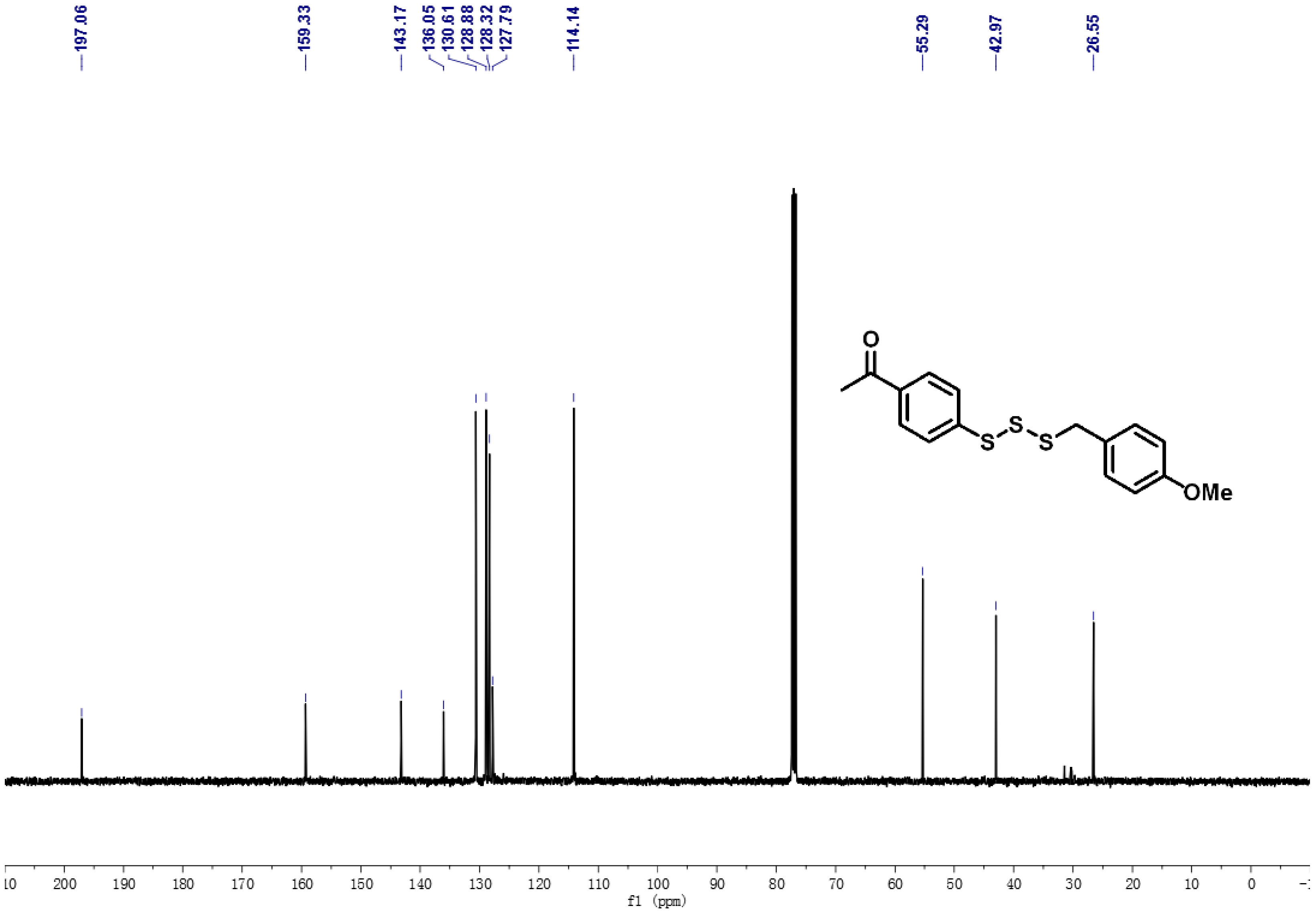

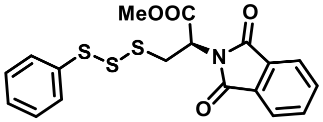

7d

^{1}H NMR ($CDCl_3$)

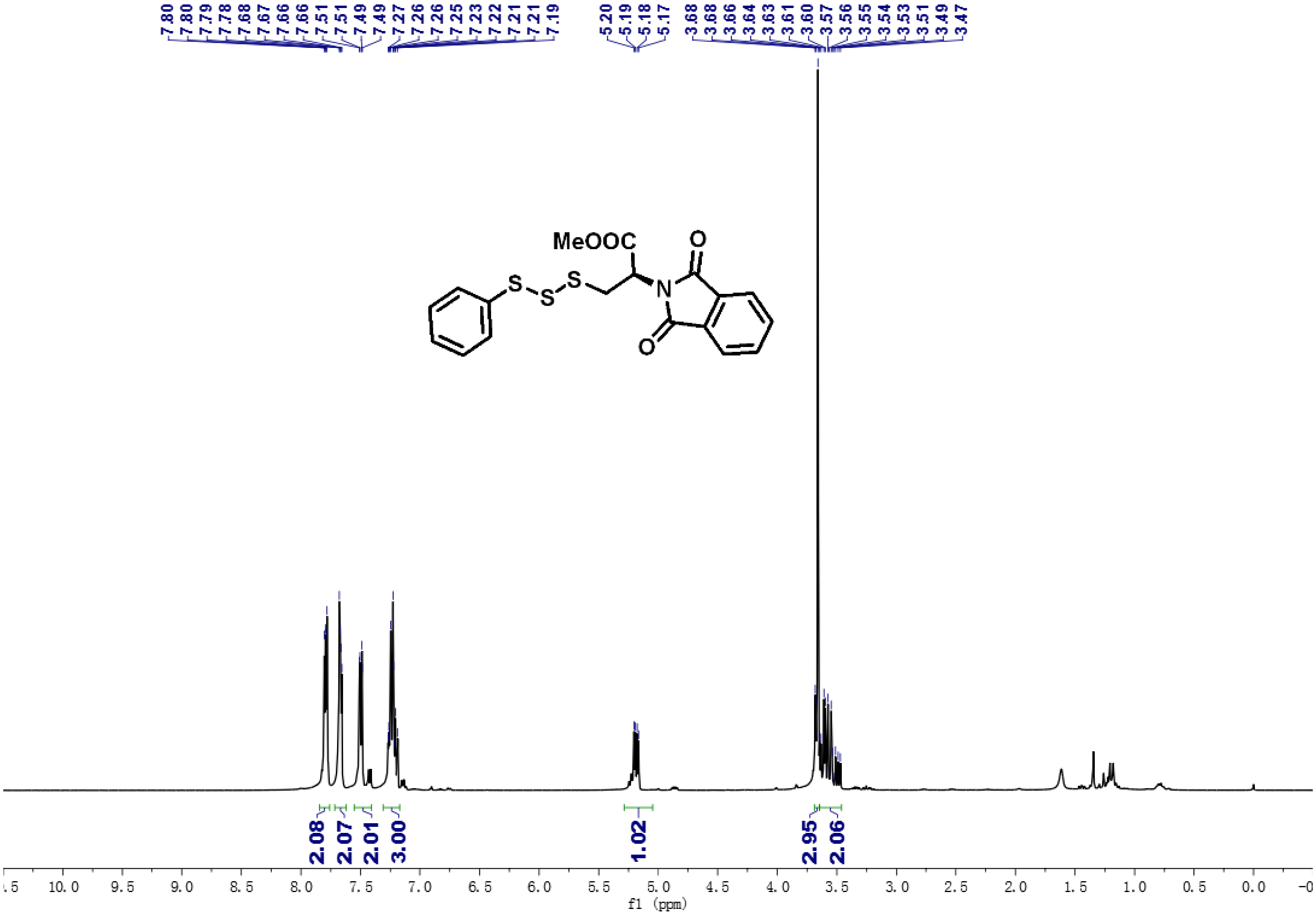

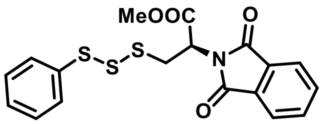

7d

^{13}C NMR ($CDCl_3$)

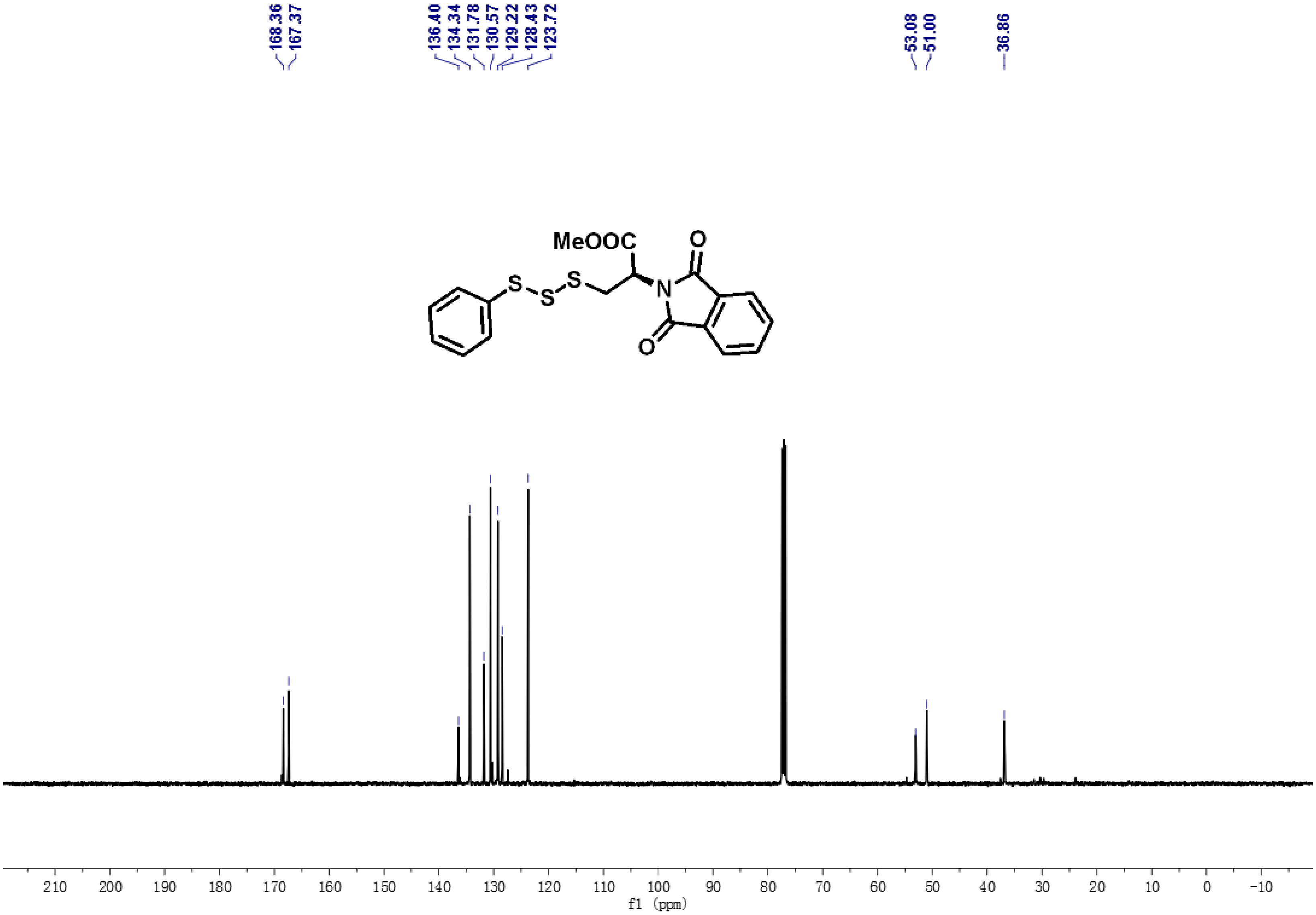

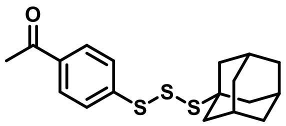

7e

^{1}H NMR ($CDCl_3$)

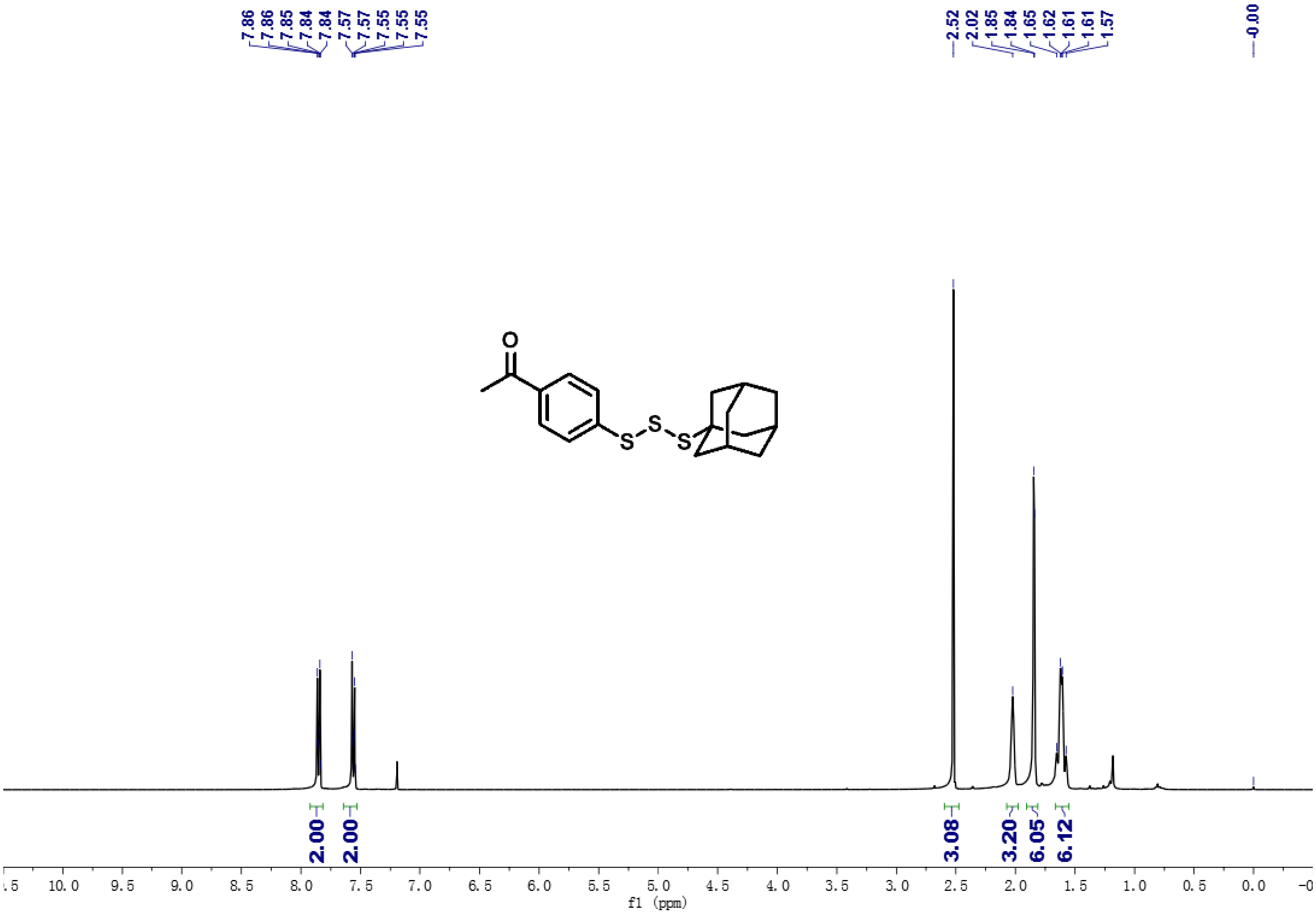

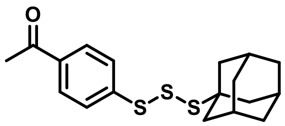

7e

^{13}C NMR ($CDCl_3$)

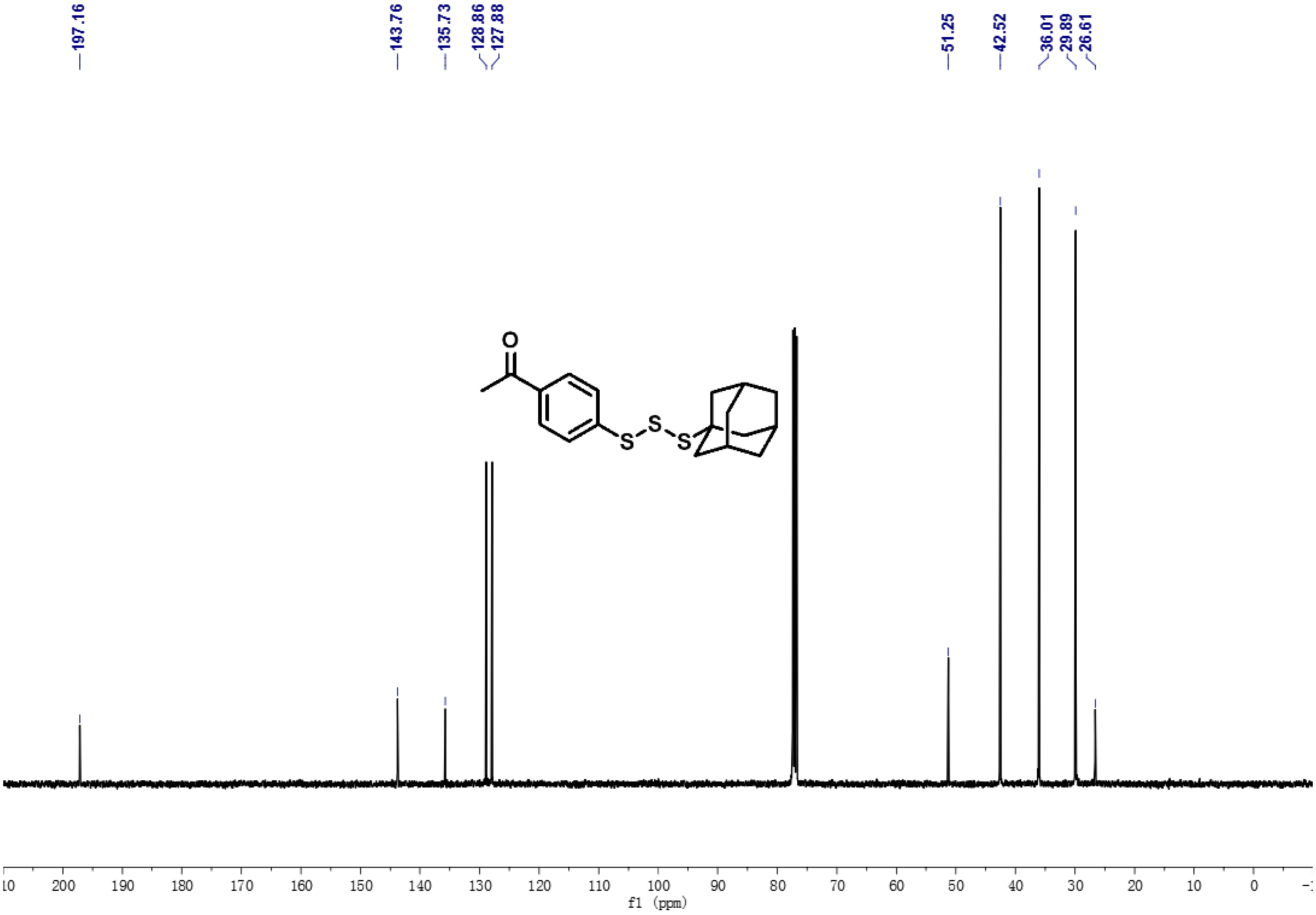

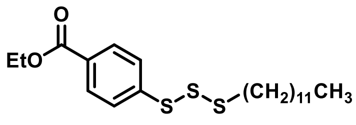

7g

^{1}H NMR ($CDCl_3$)

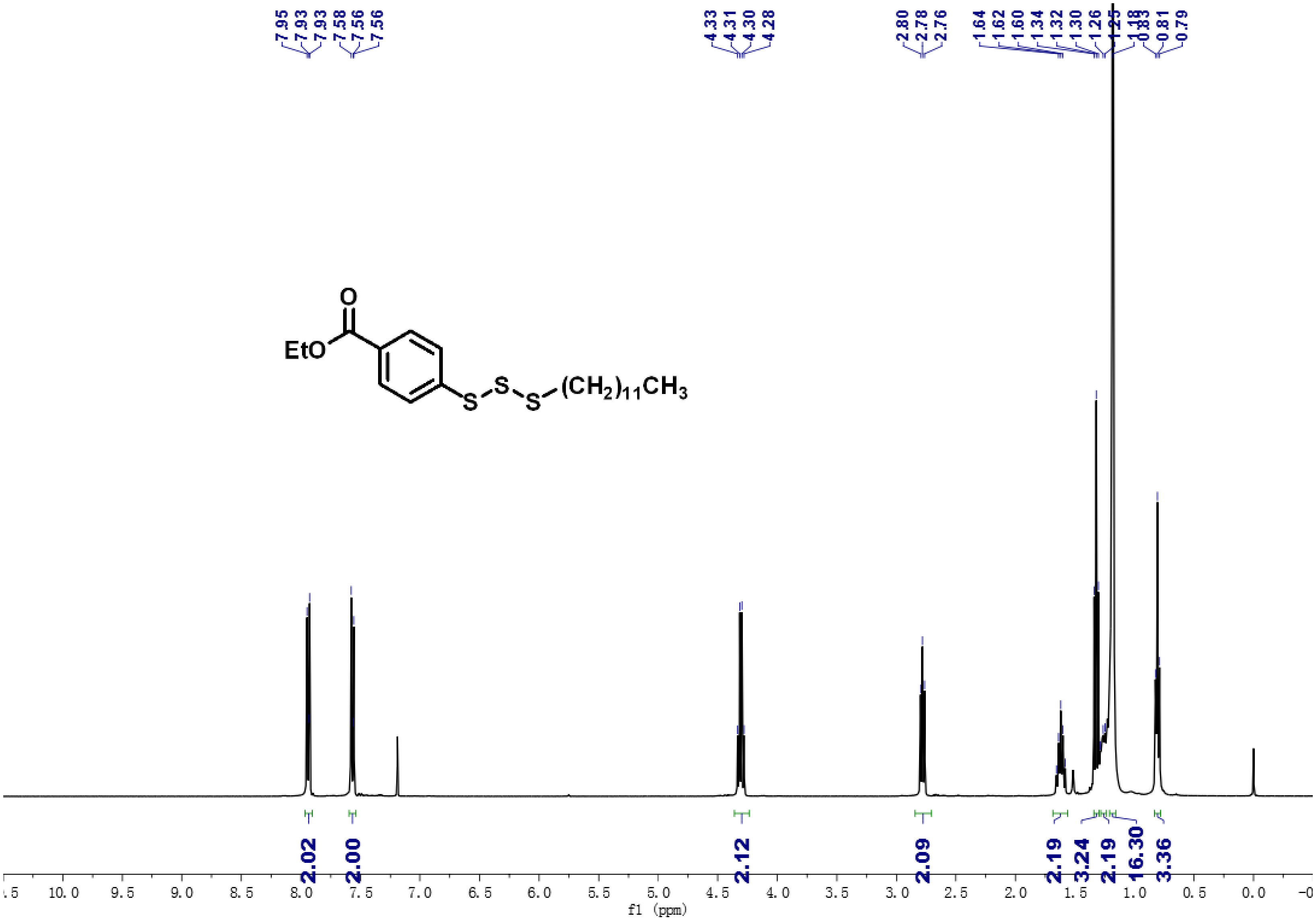

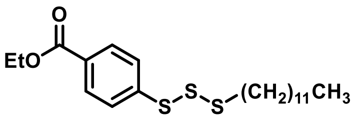

7g

^{13}C NMR ($CDCl_3$)

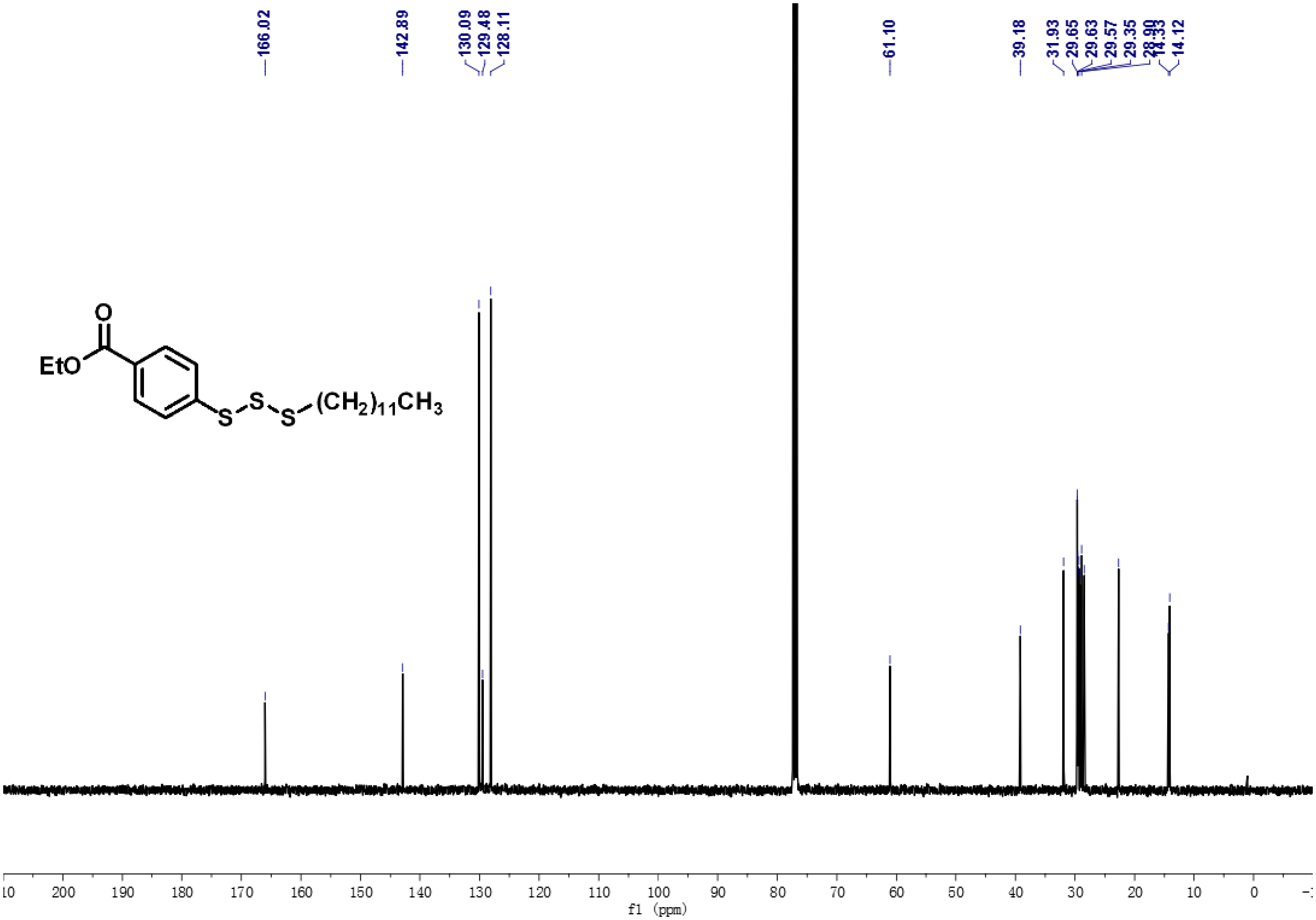

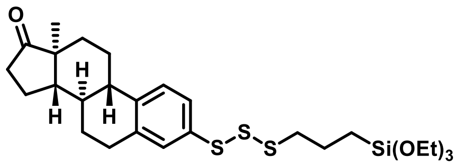

7h

^{1}H NMR ($CDCl_3$), ^{13}C NMR ($CDCl_3$)

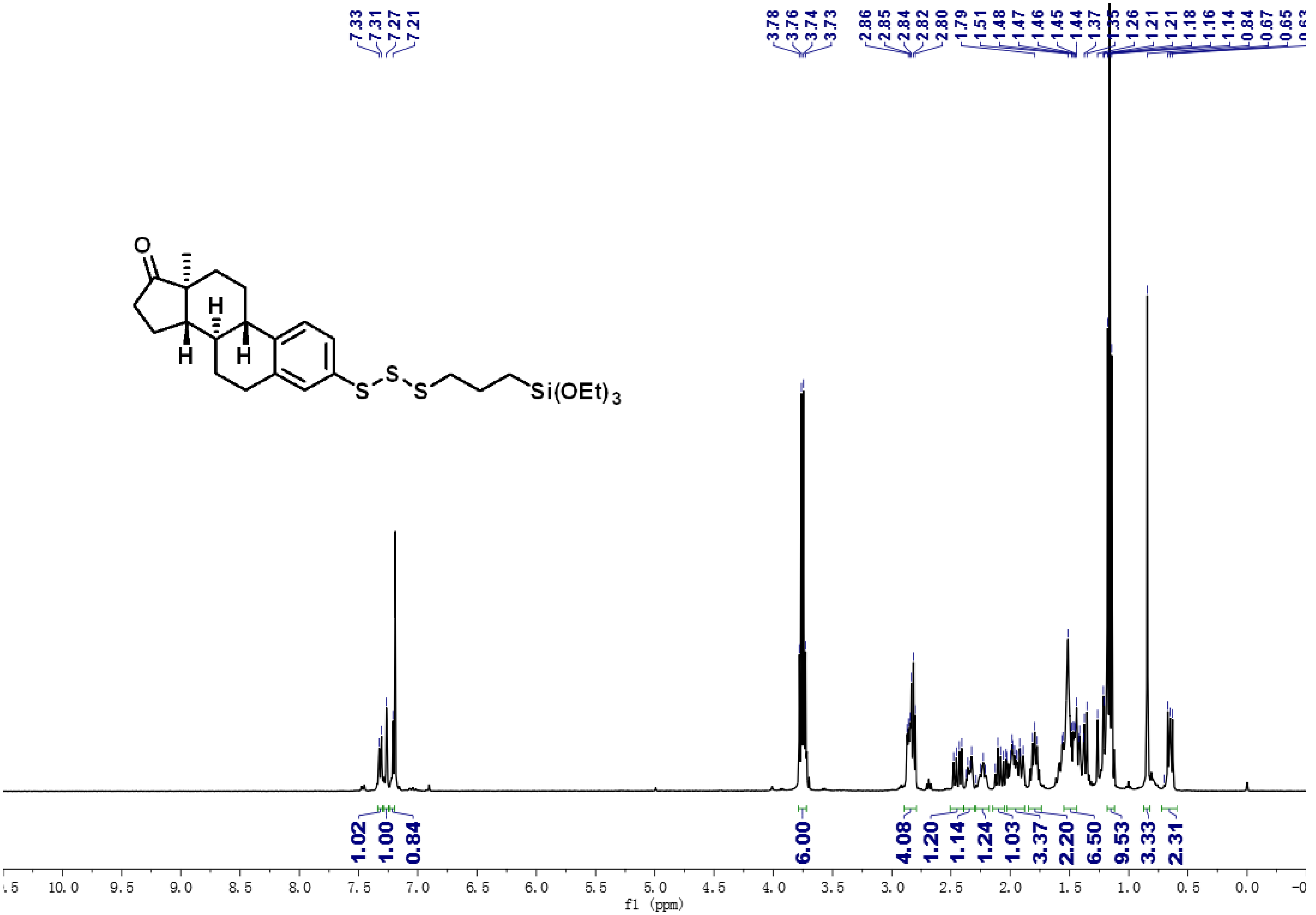

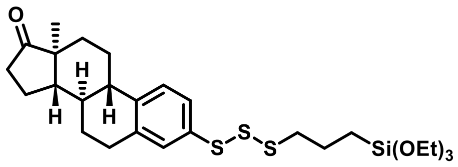

7h

^{13}C NMR (CDCl$_3$)

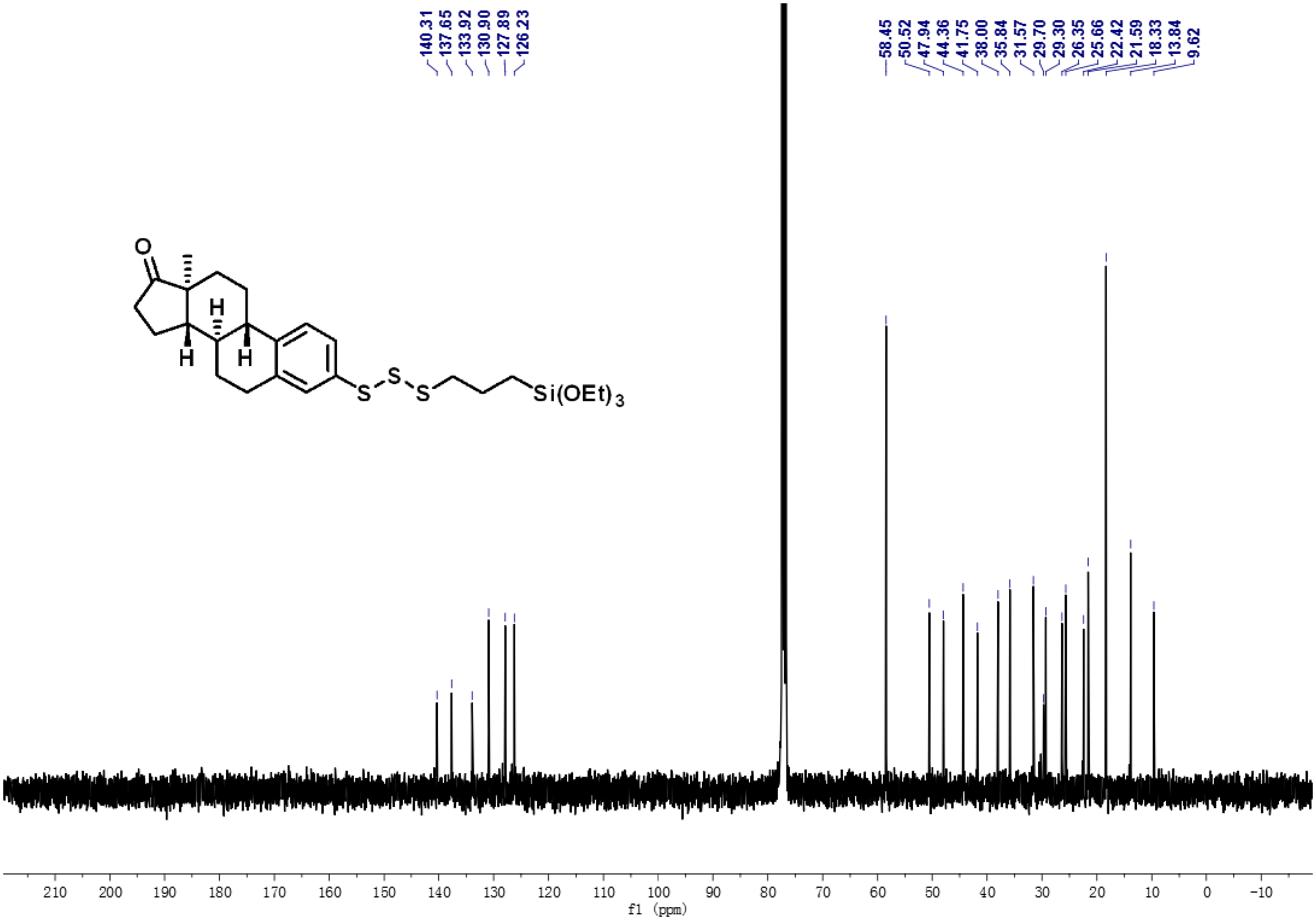

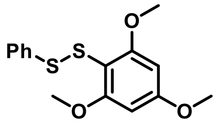

8a

^{1}H NMR ($CDCl_3$)

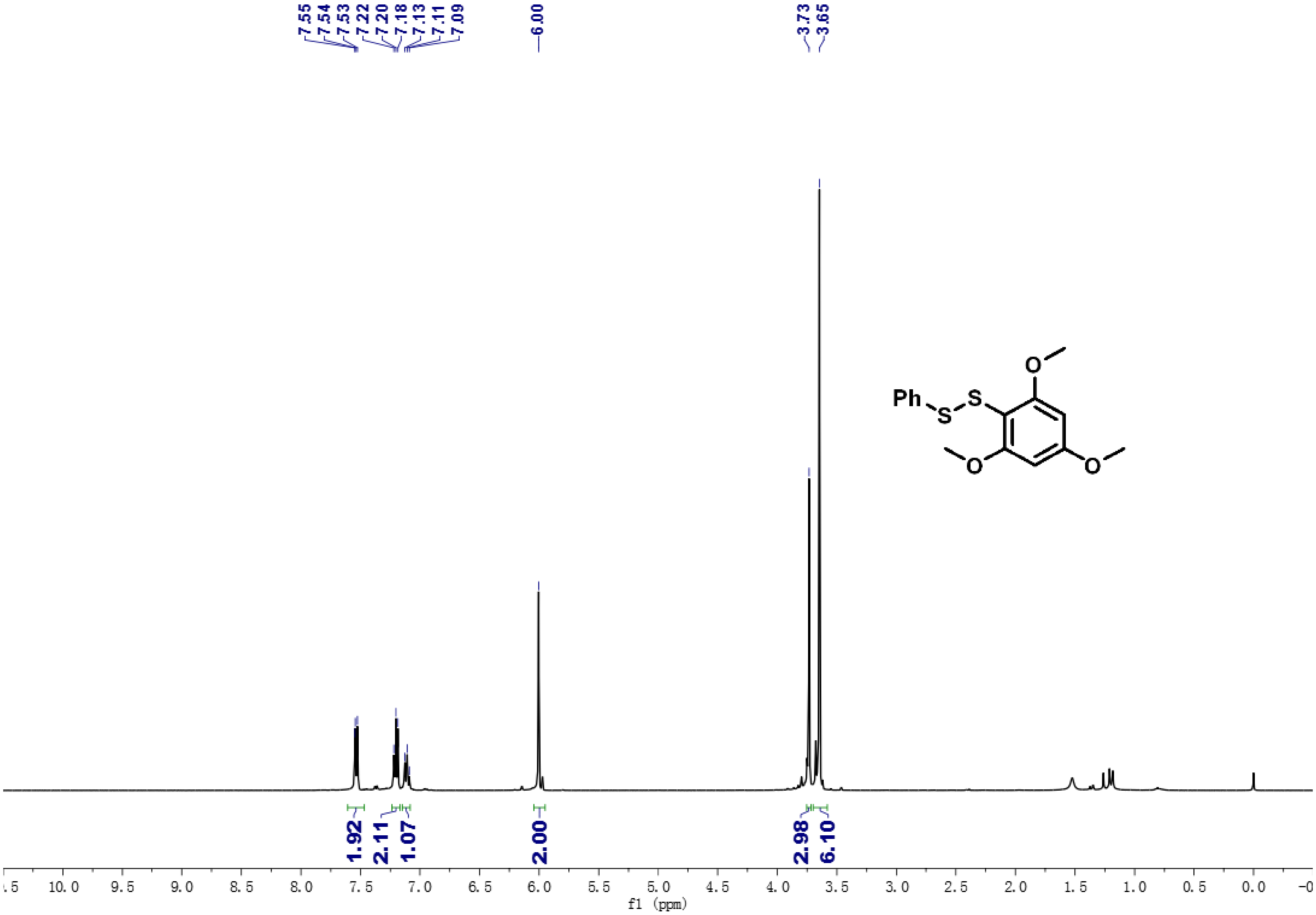

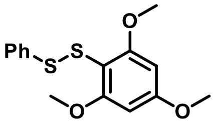

8a

^{13}C NMR ($CDCl_3$)

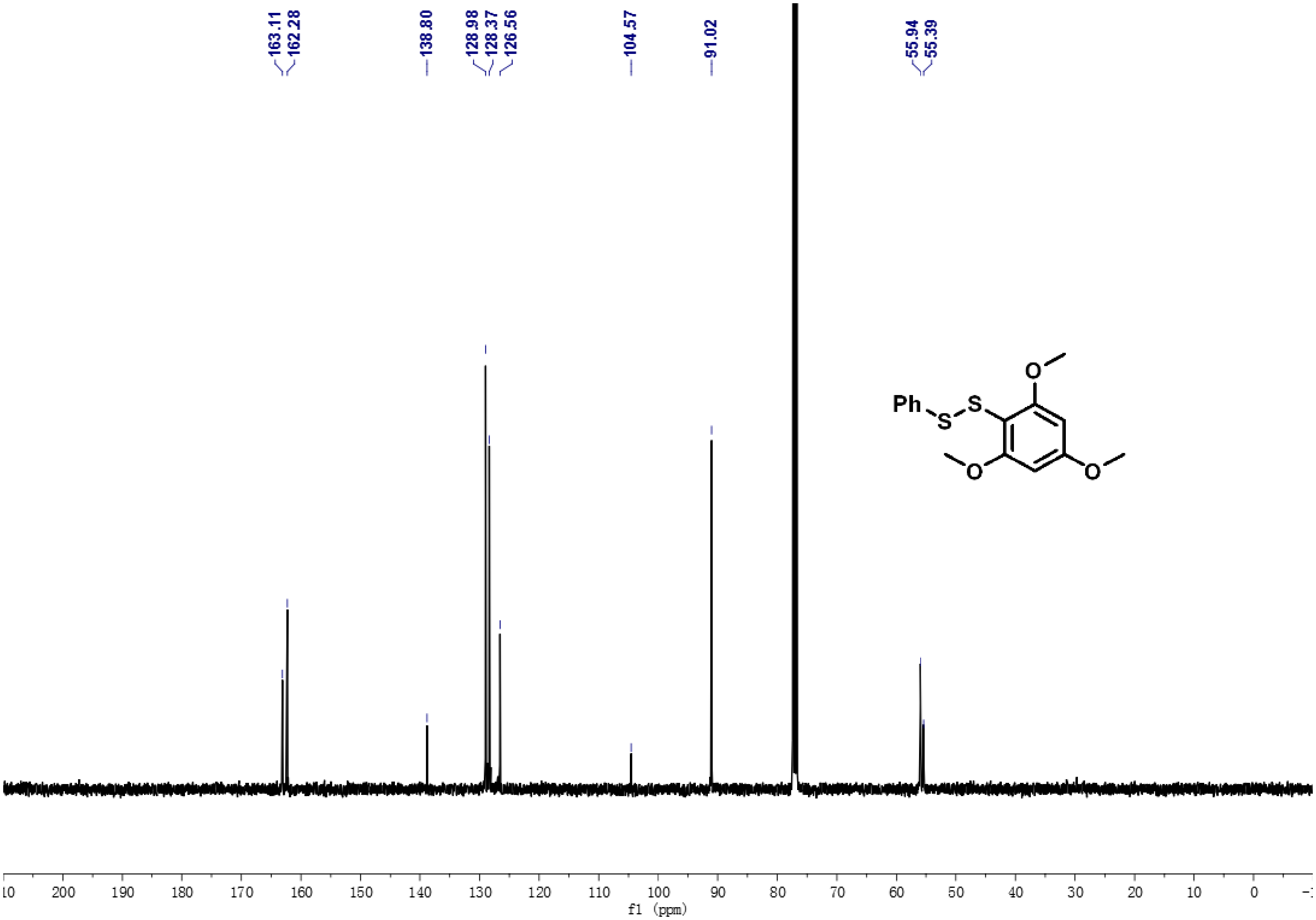

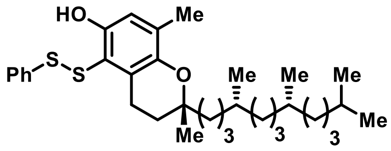

8b

^{1}H NMR ($CDCl_3$)

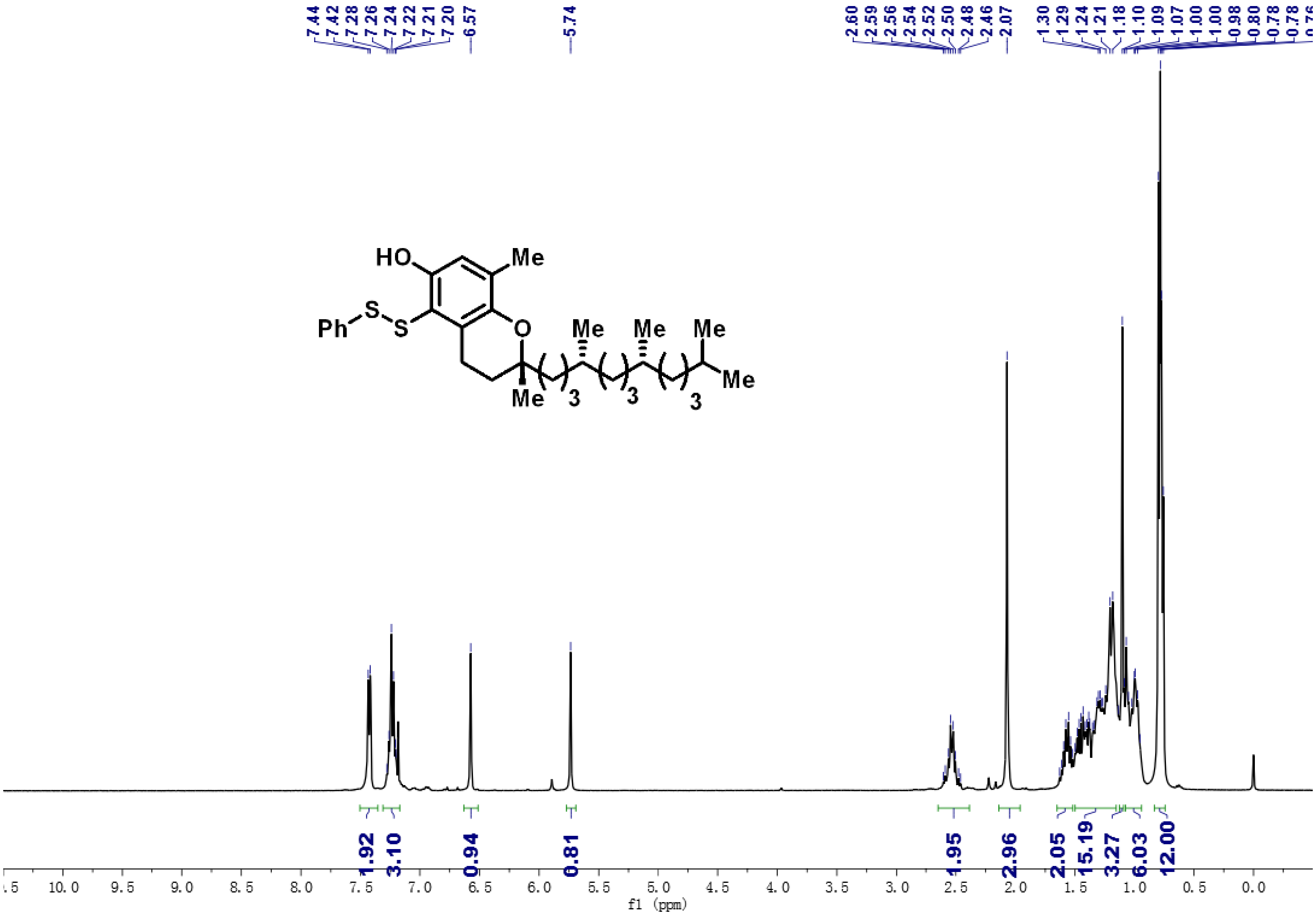

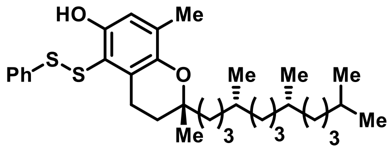

8b

^{13}C NMR ($CDCl_3$)

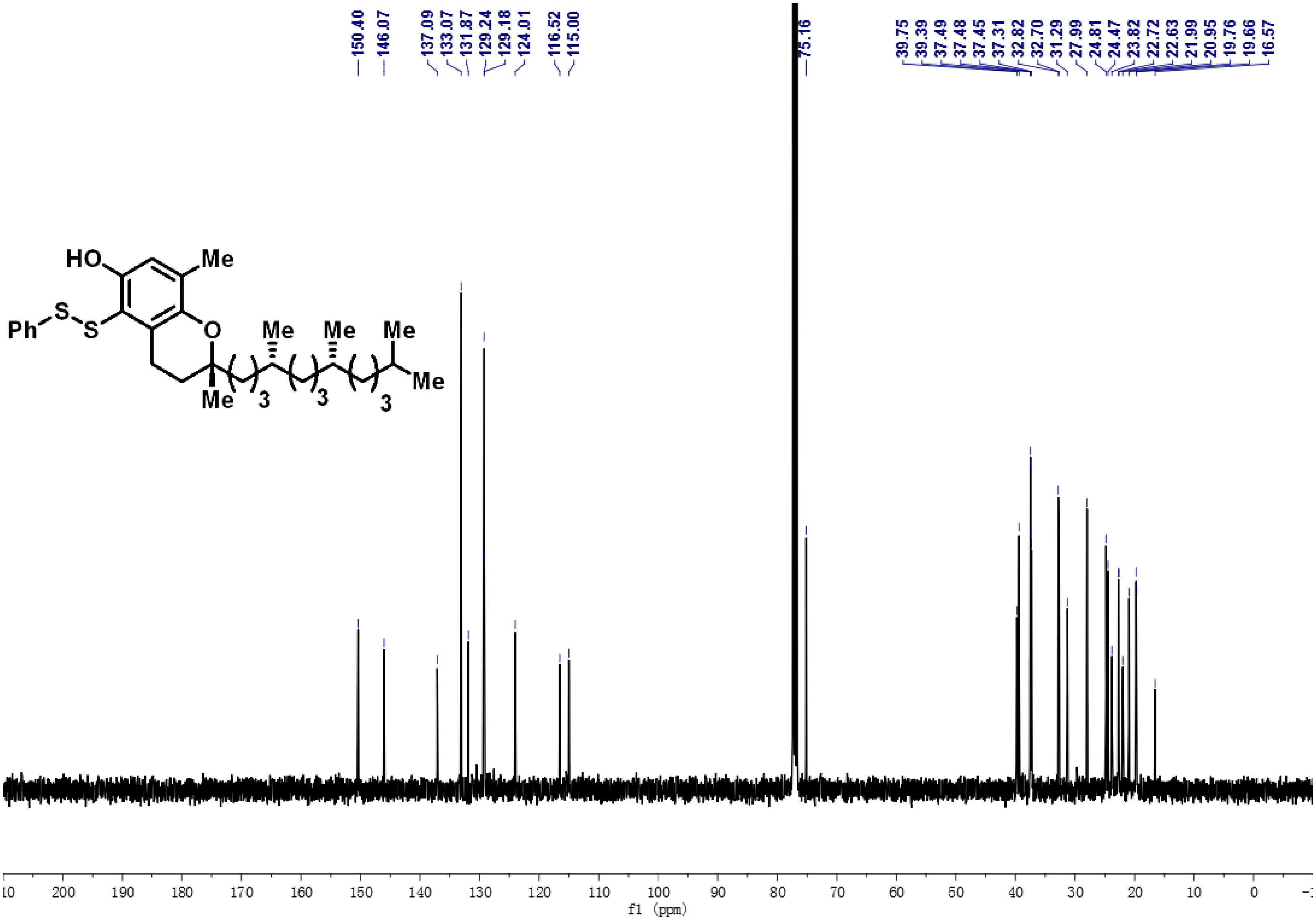

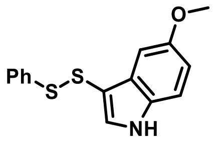

8c

^{1}H NMR ($CDCl_3$)

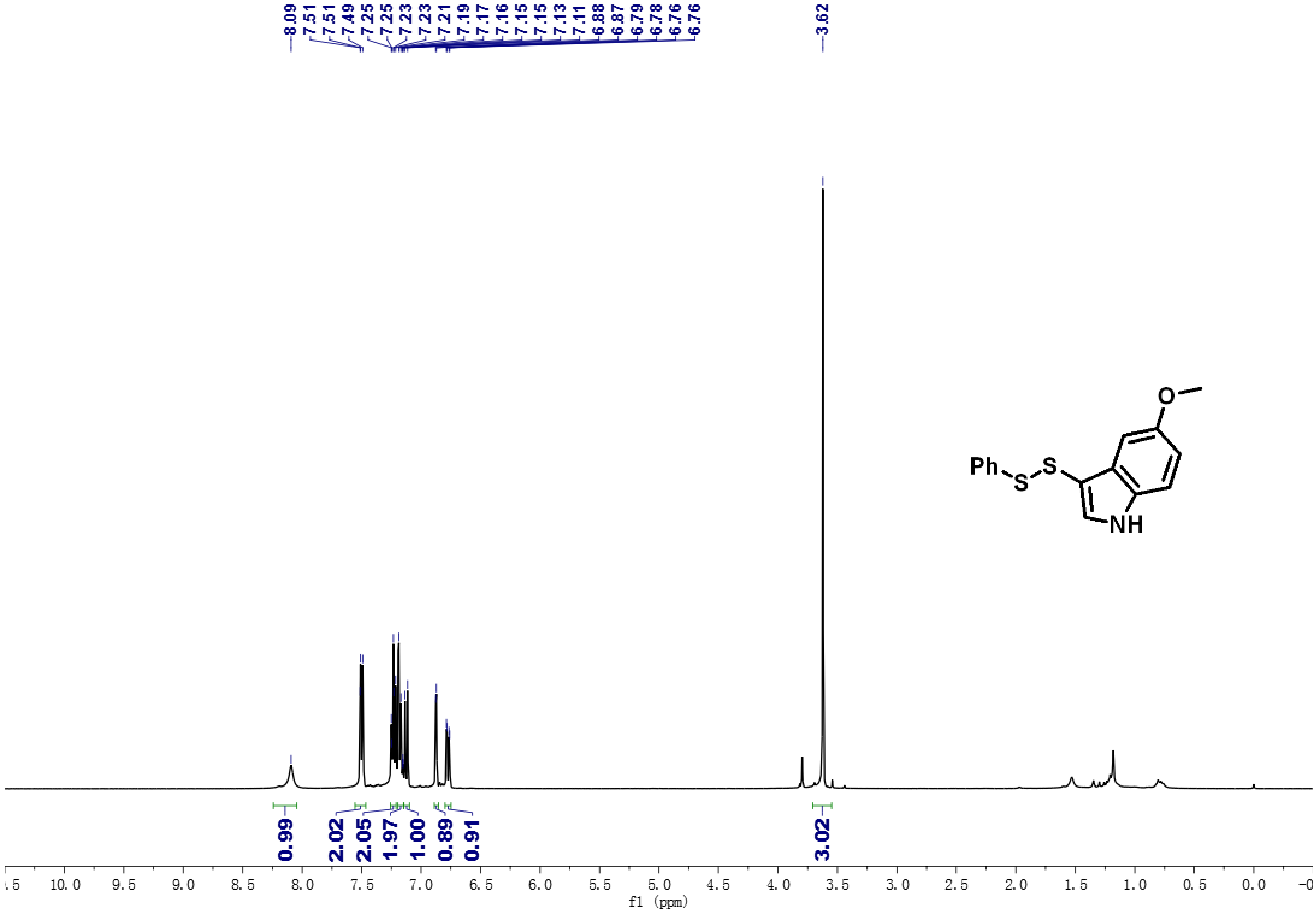

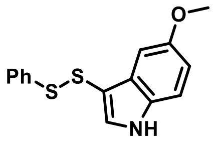

8c

^{13}C NMR ($CDCl_3$)

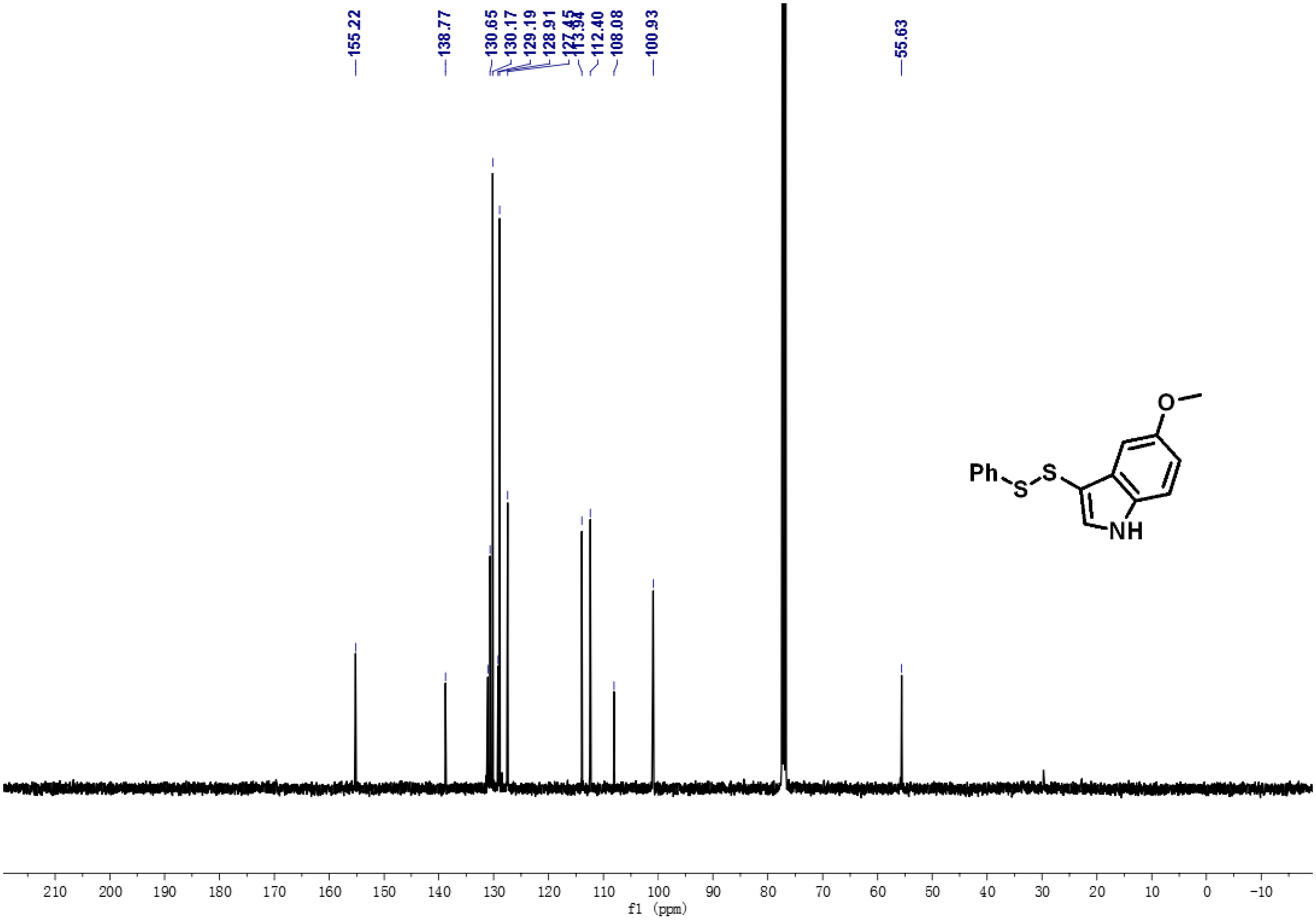

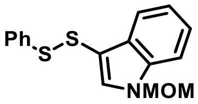

8d

^{1}H NMR ($CDCl_3$)

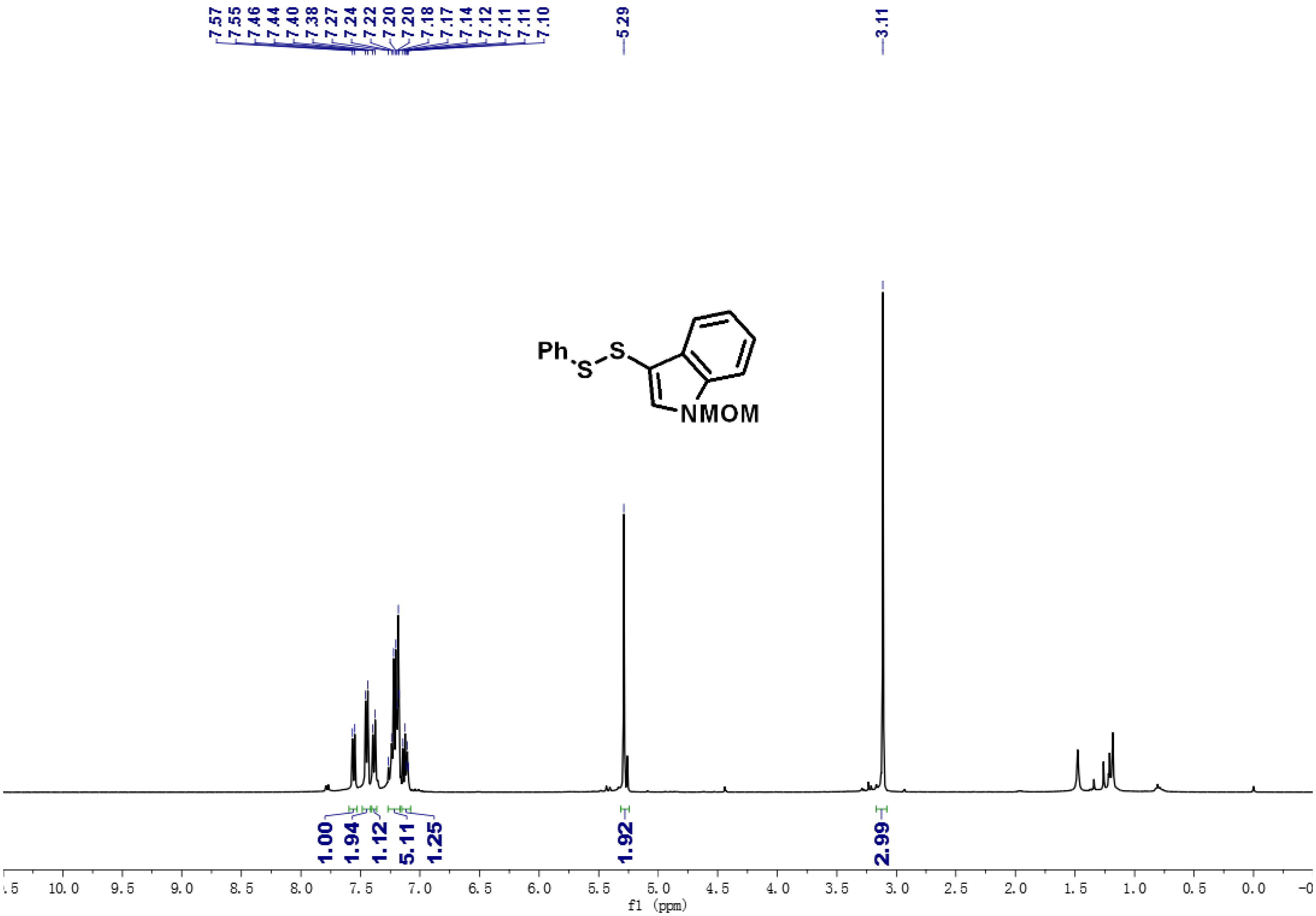

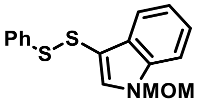

8d

^{13}C NMR ($CDCl_3$)

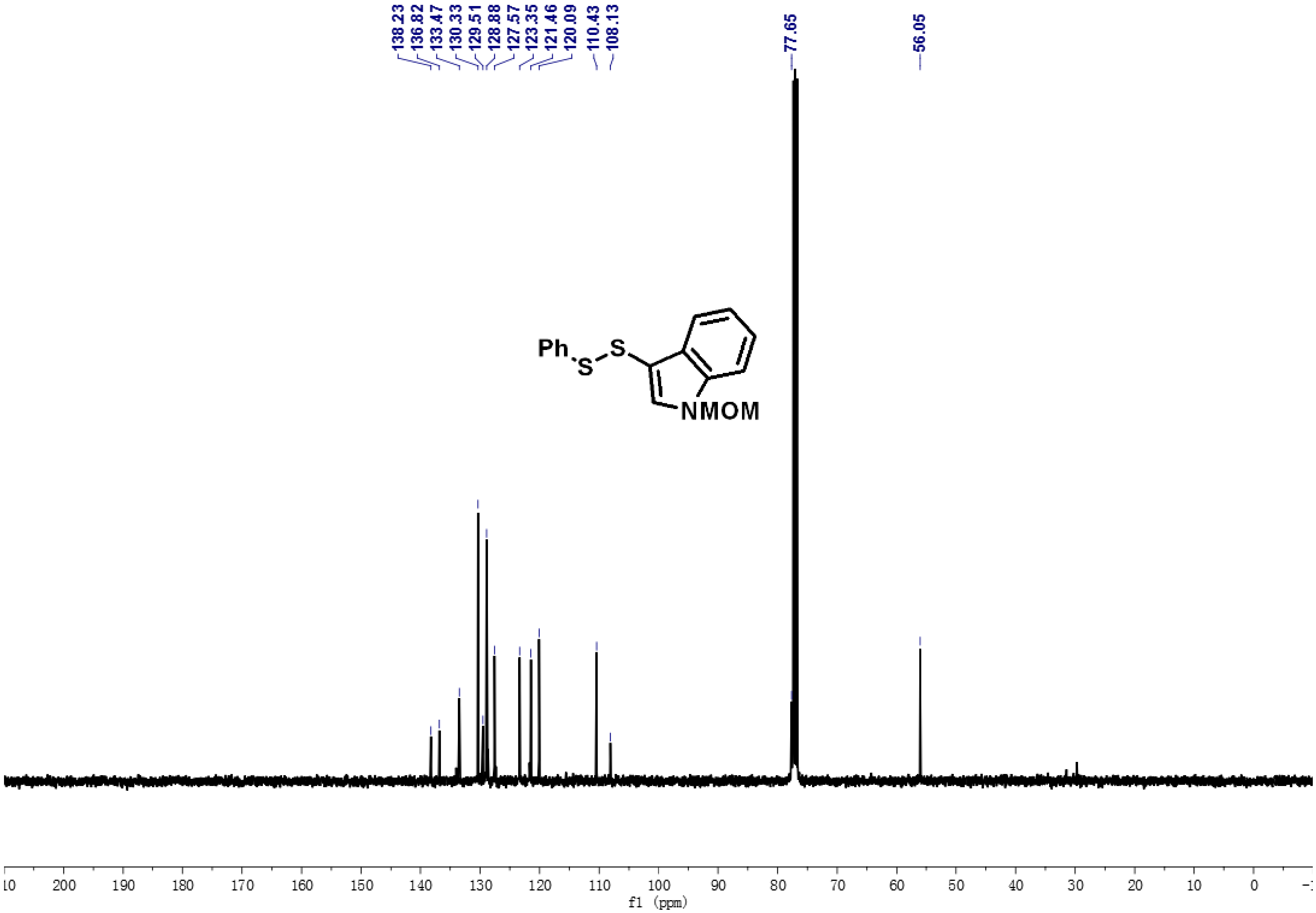

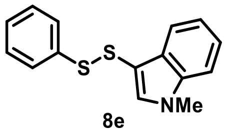

8e

^{1}H NMR ($CDCl_3$)

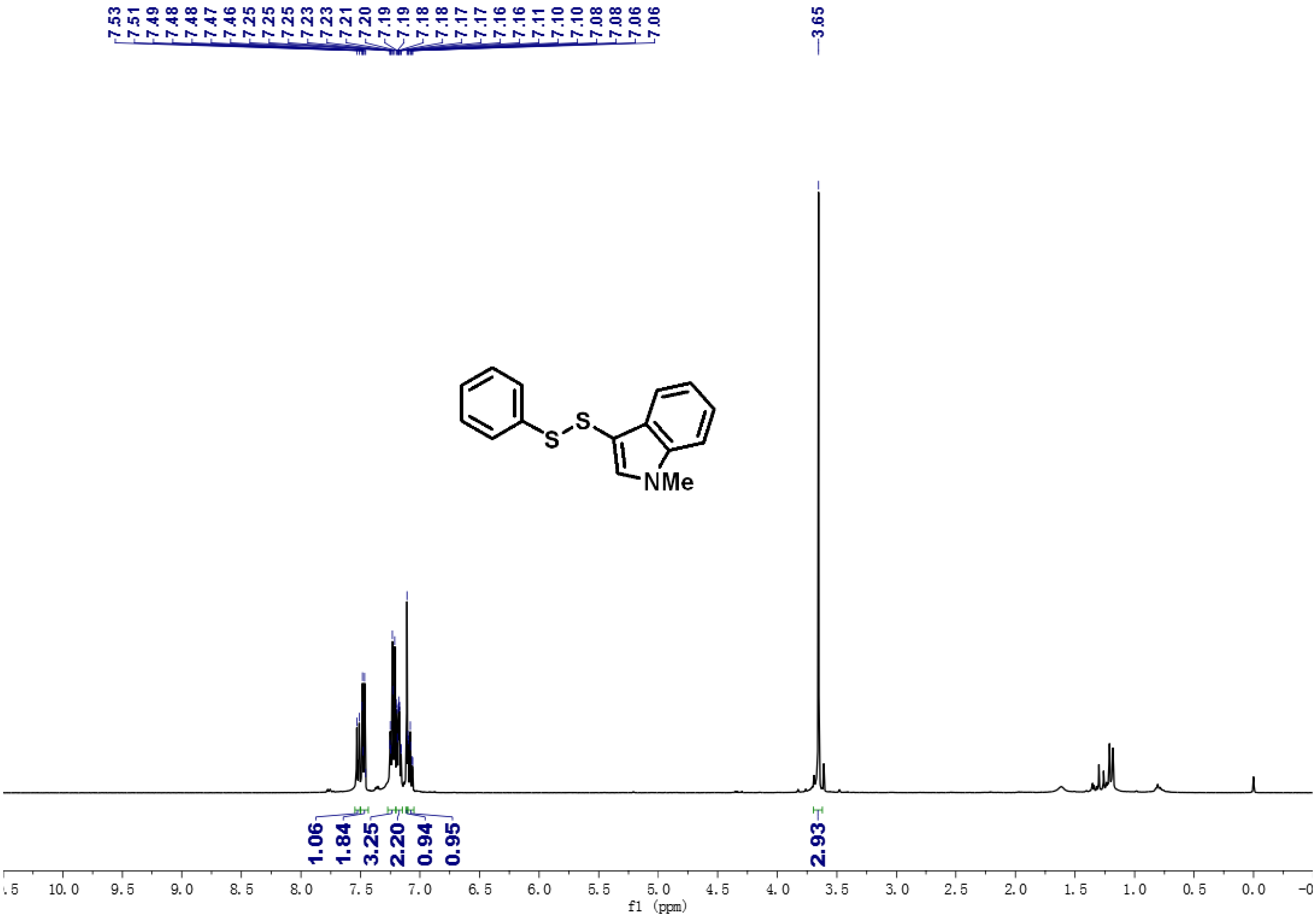

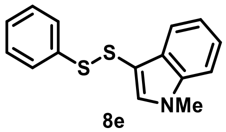

8e

^{13}C NMR ($CDCl_3$)

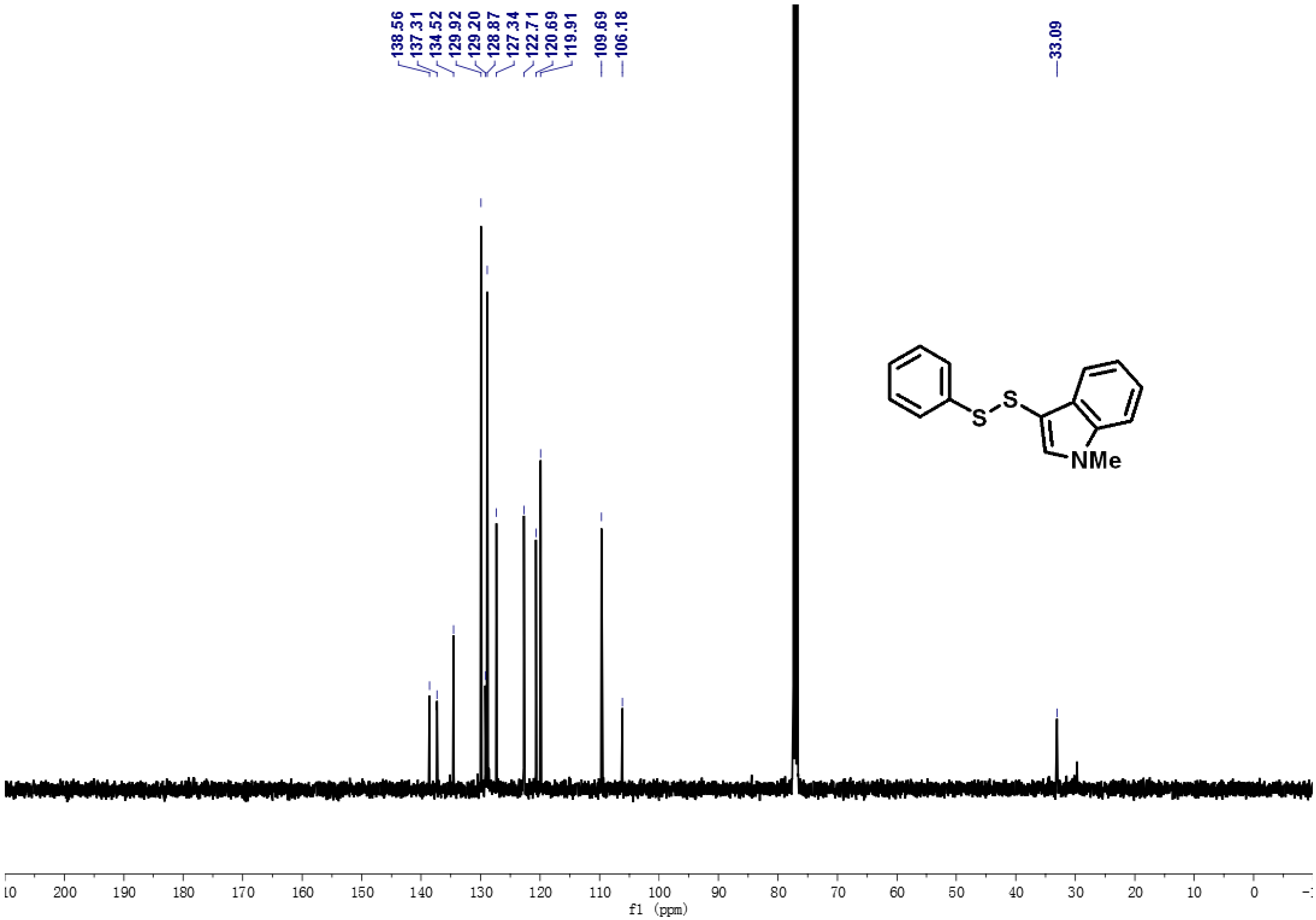

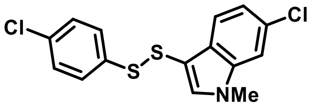

8f

^{1}H NMR ($CDCl_3$)

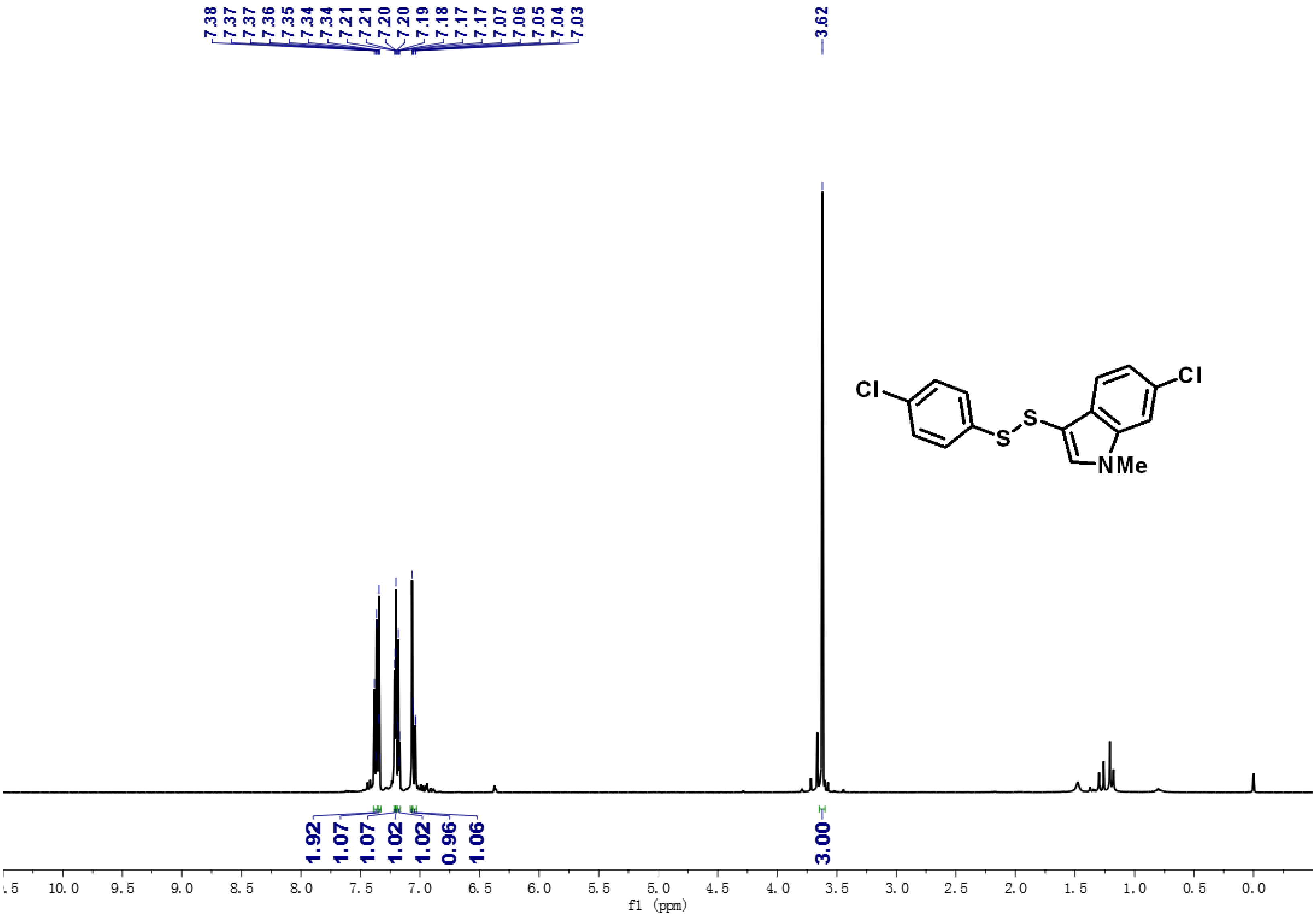

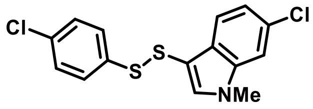

8f

^{13}C NMR ($CDCl_3$)

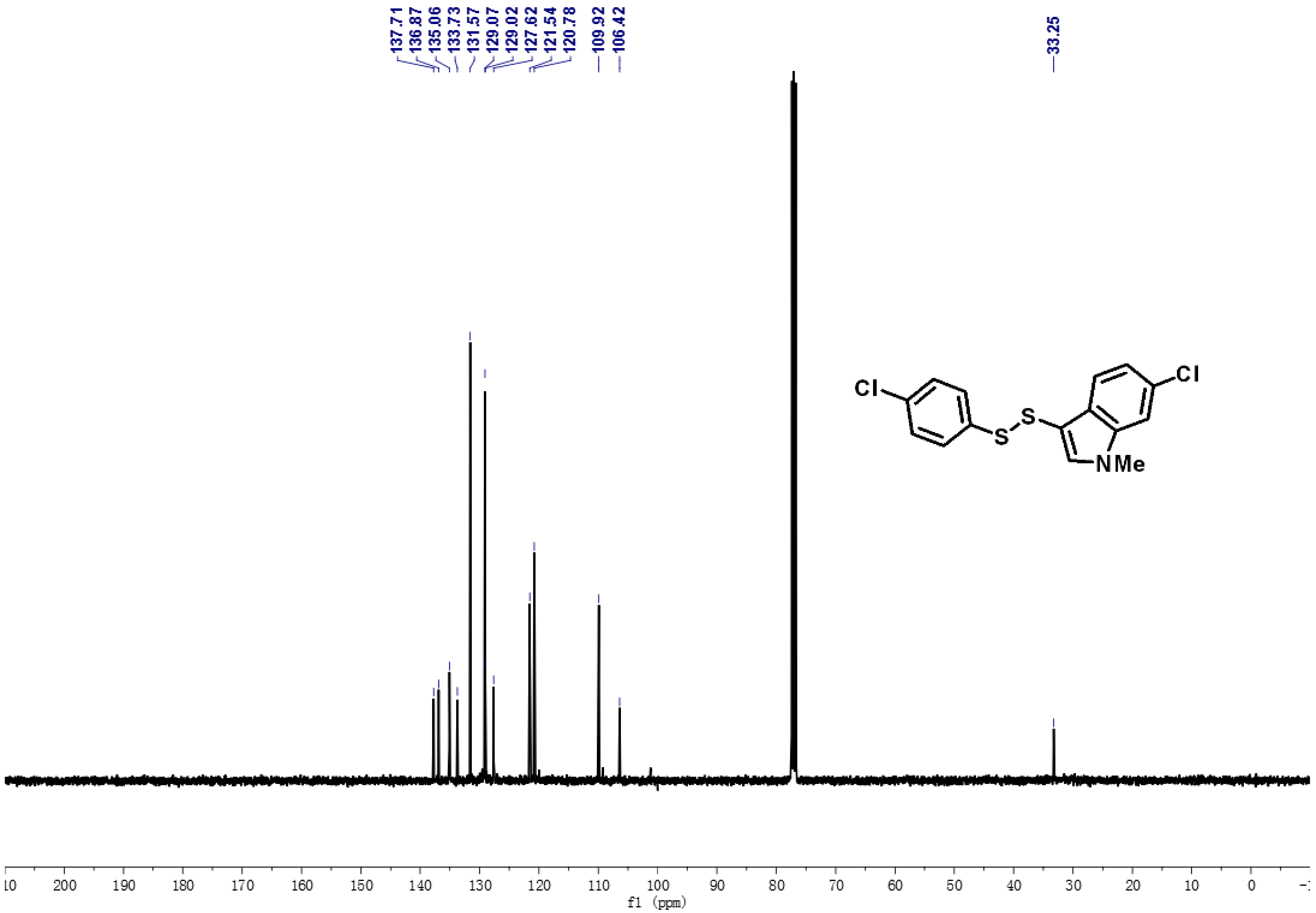

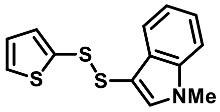

8g

^{1}H NMR ($CDCl_3$)

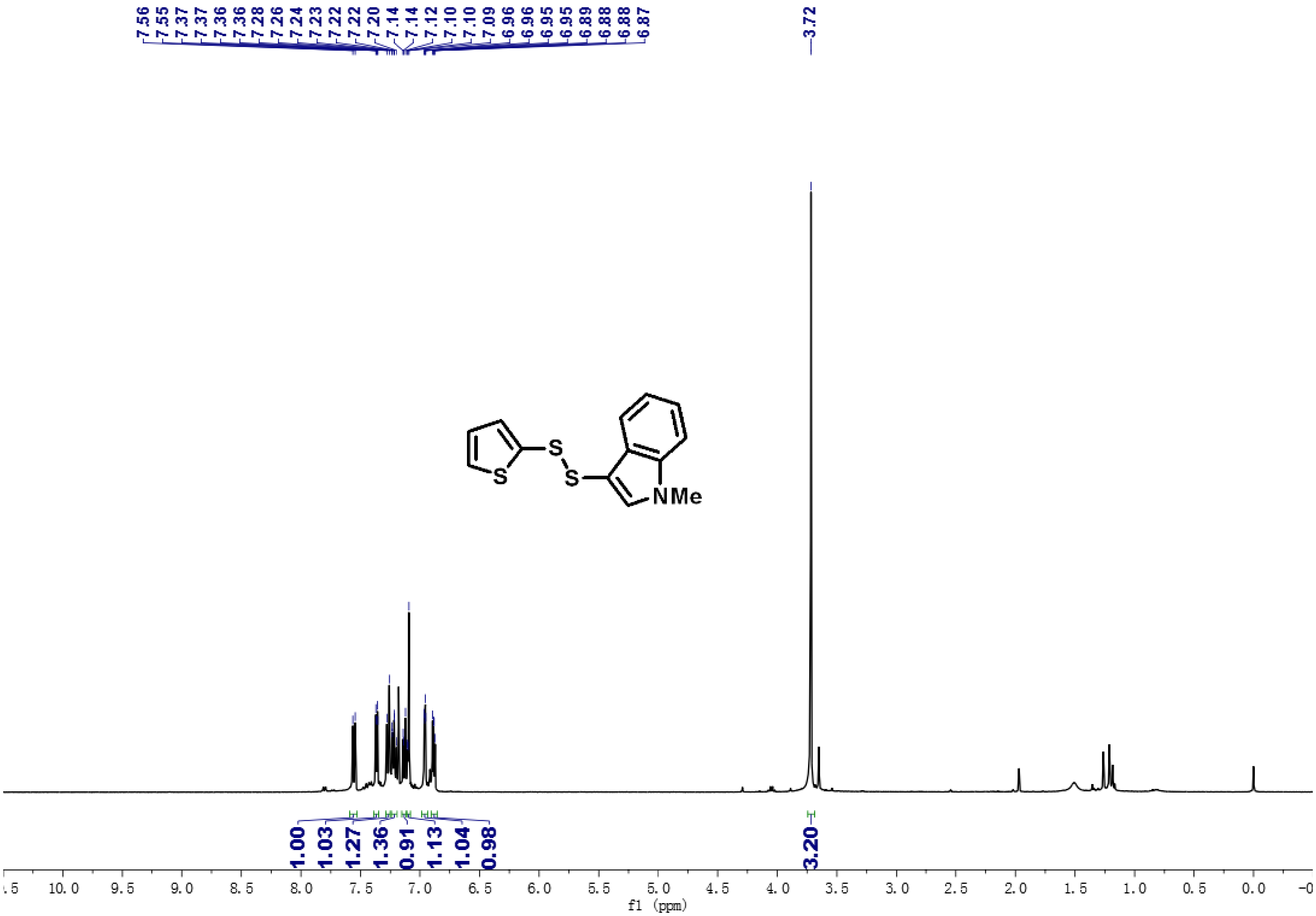

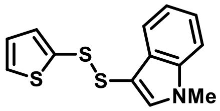

8g

^{13}C NMR ($CDCl_3$)

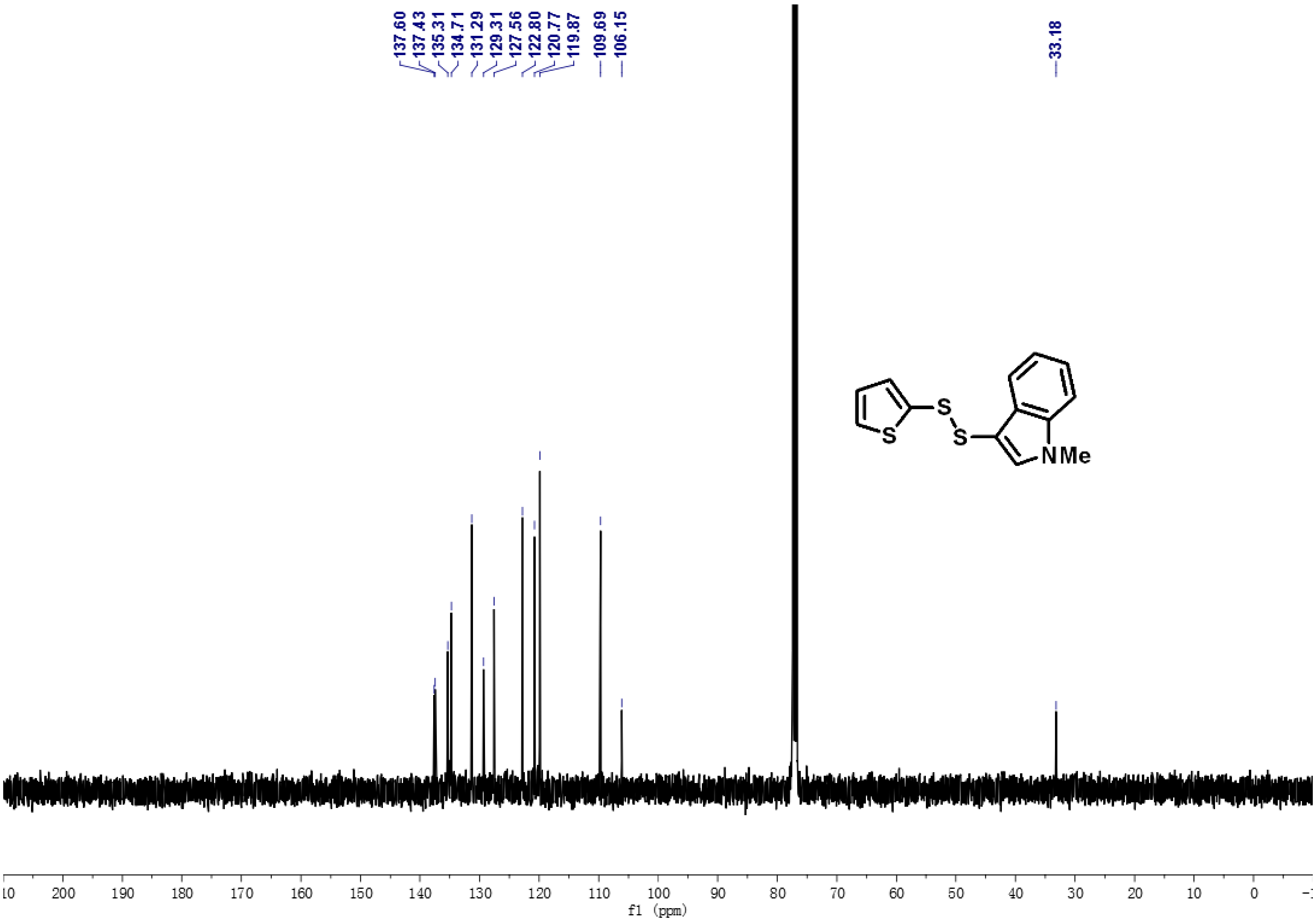

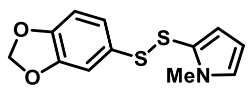

8h

^{1}H NMR ($CDCl_3$)

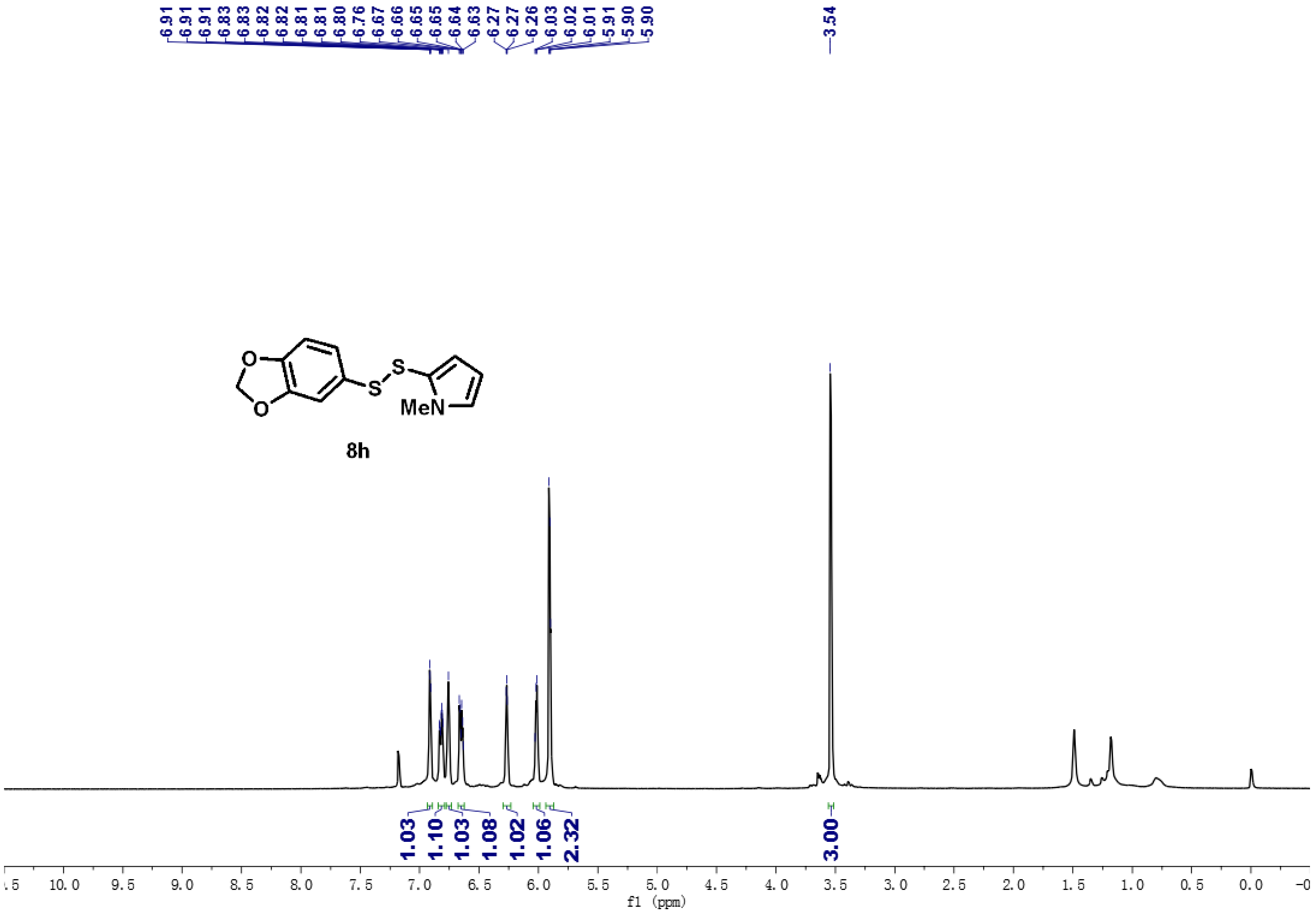

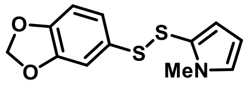

8h

^{13}C NMR ($CDCl_3$)

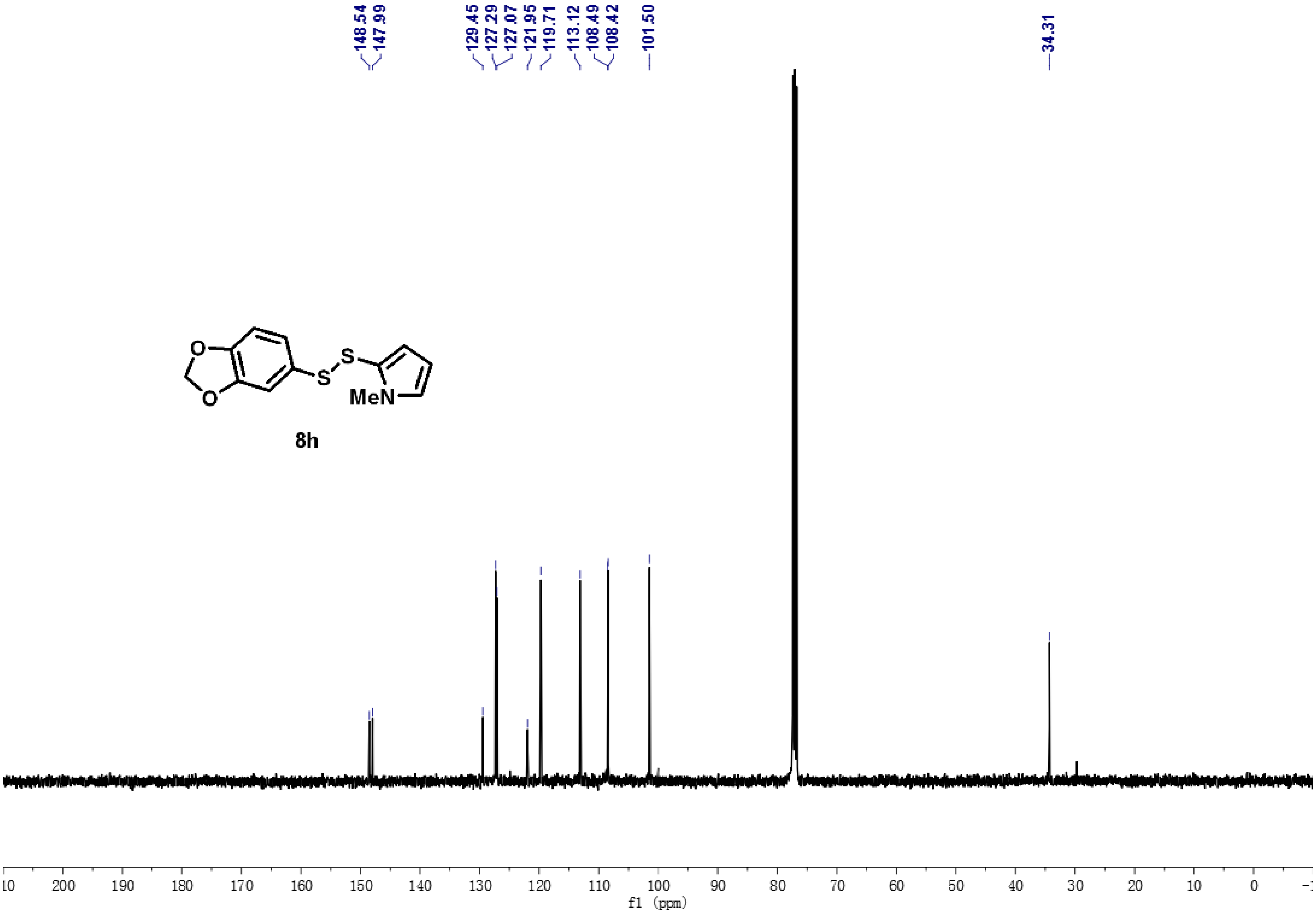

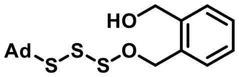

S2

^{1}H NMR ($CDCl_3$)

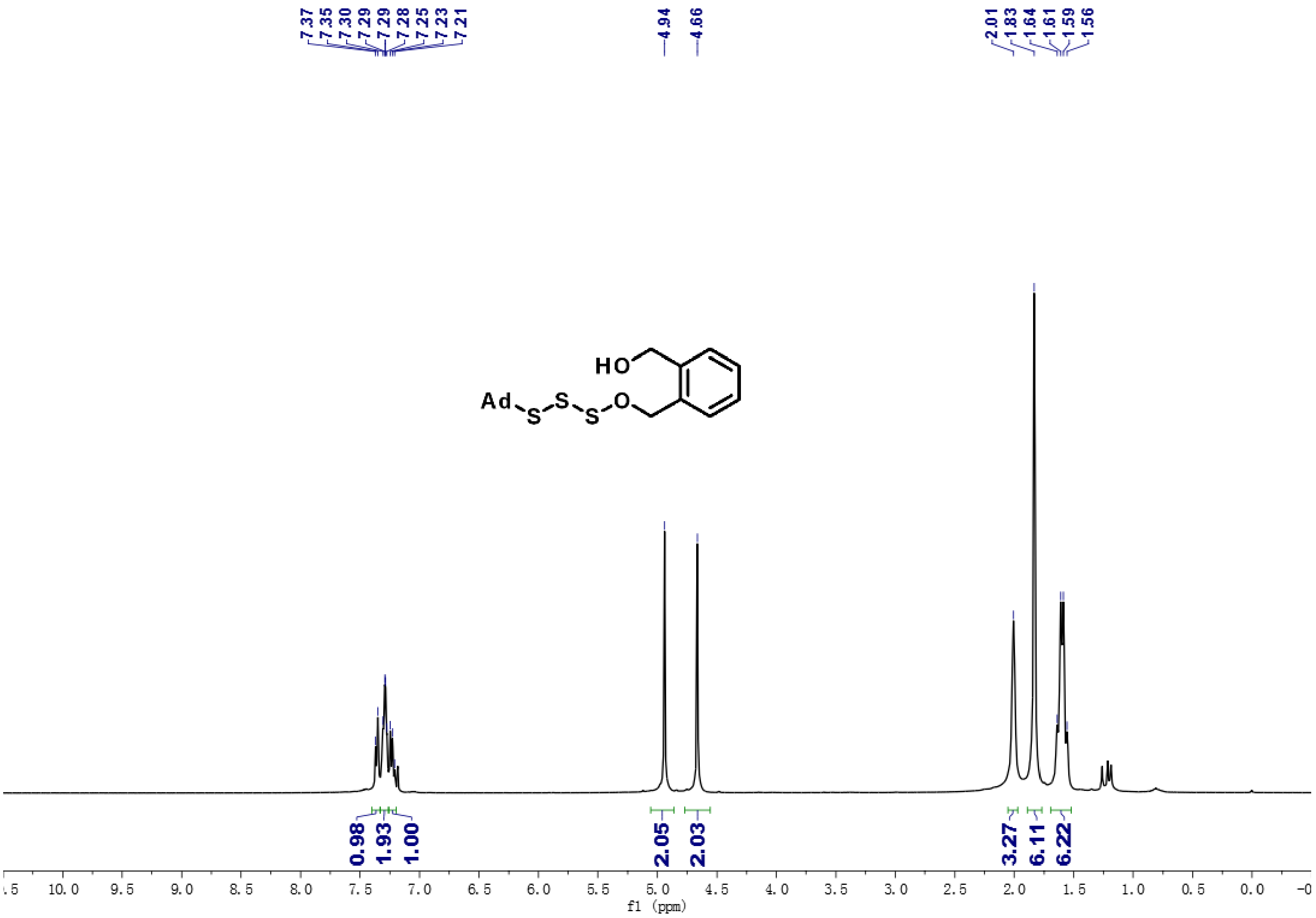

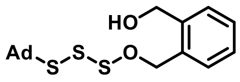

S2

^{13}C NMR ($CDCl_3$)

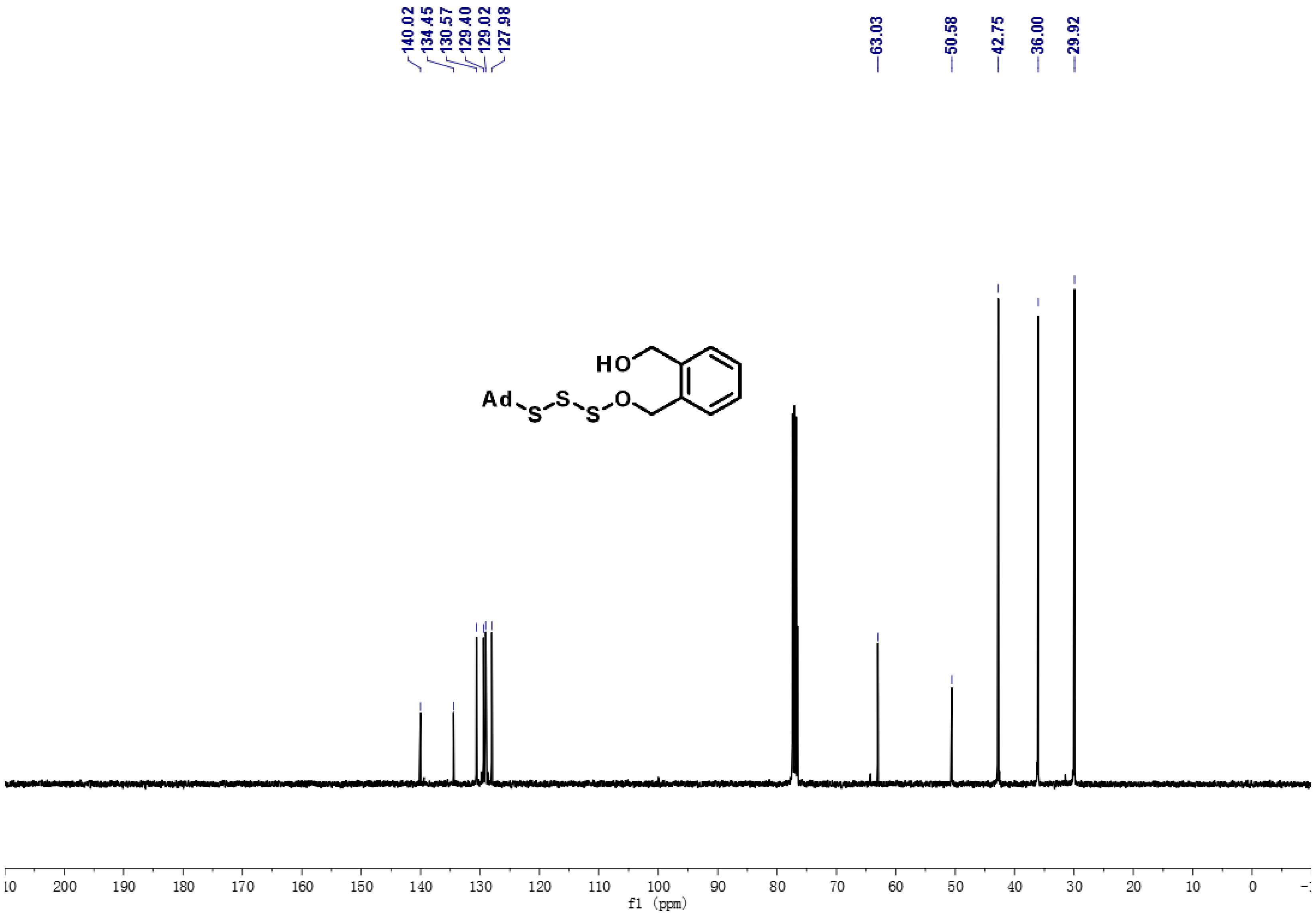
140.02
134.45
130.57
129.40
129.02
127.98
63.03
50.58
42.75
36.00
29.92
HO
Ad
S
S
S
O
f1 (ppm)

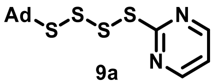

9a

^{1}H NMR ($CDCl_3$)

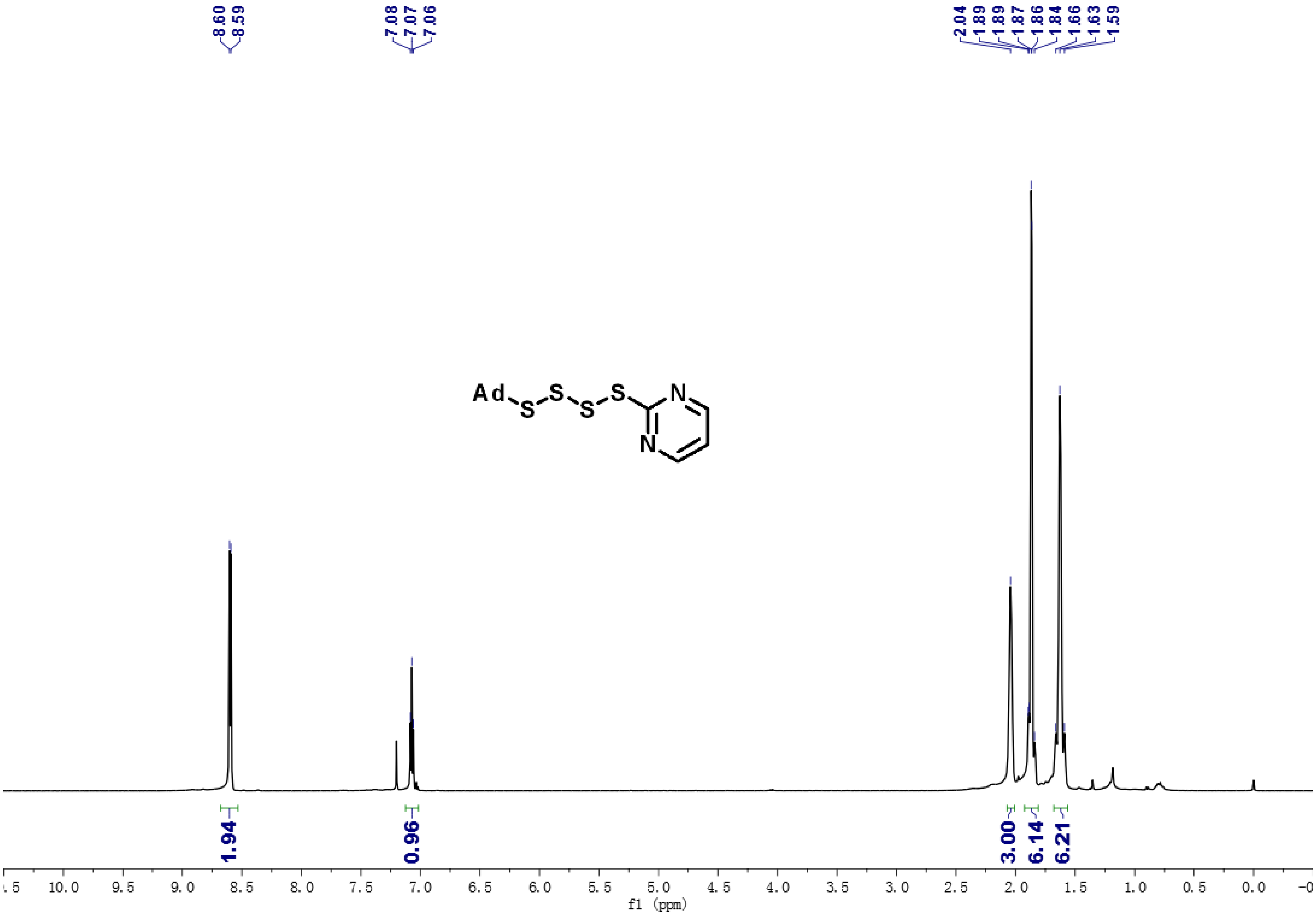

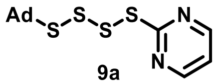

9a

^{13}C NMR ($CDCl_3$)

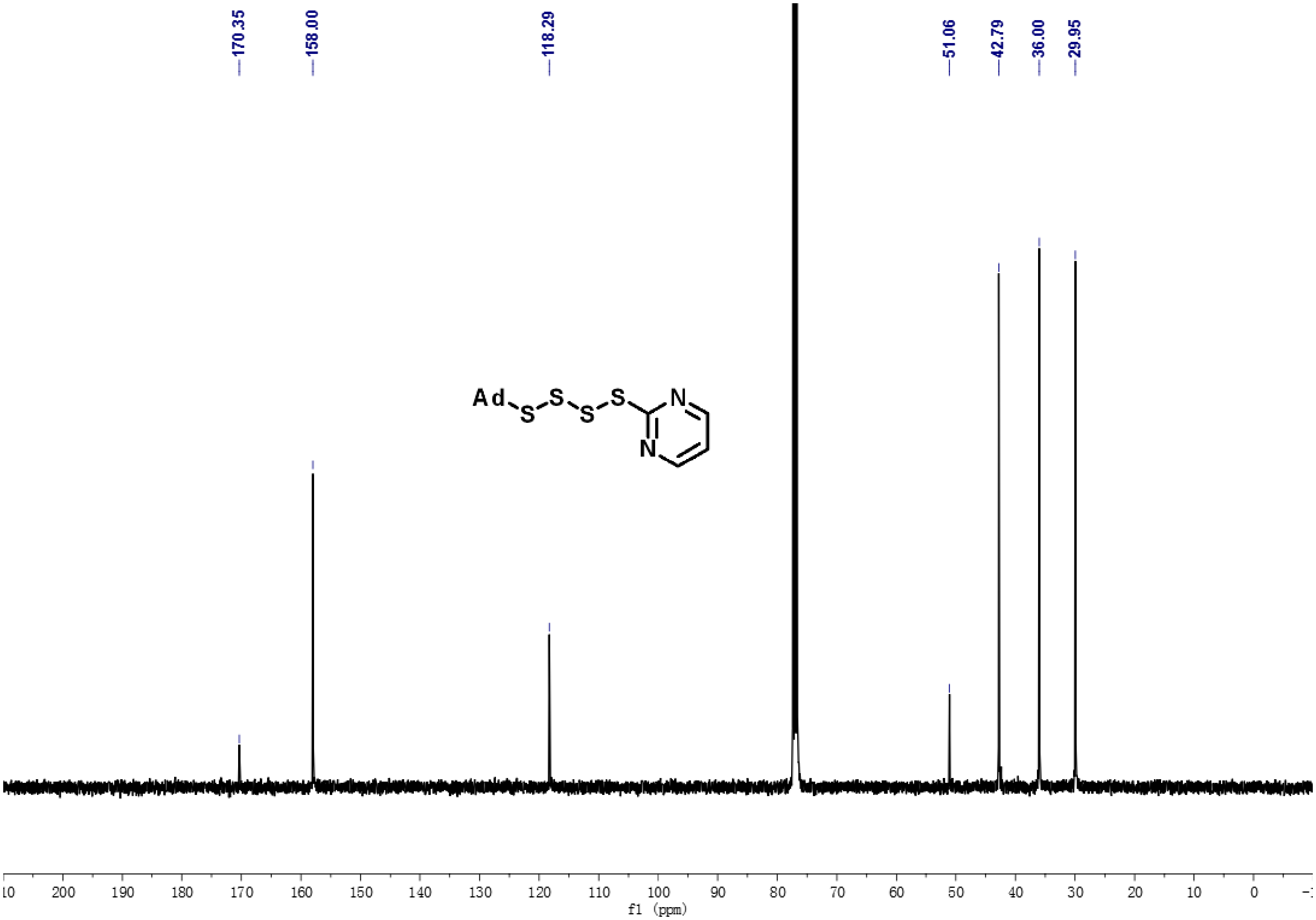

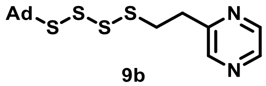

9b

^{1}H NMR ($CDCl_3$)

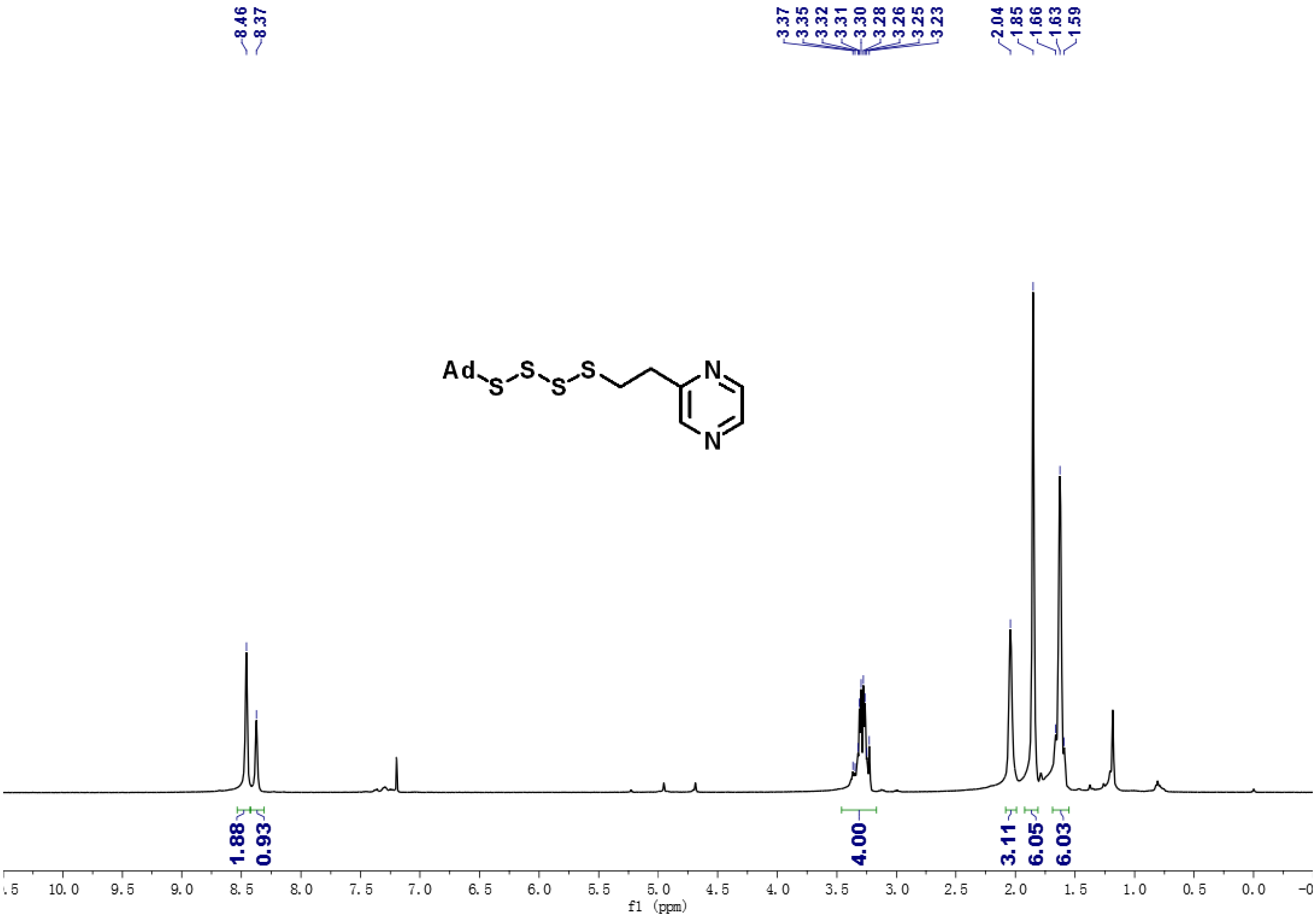

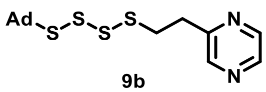

9b

^{13}C NMR ($CDCl_3$)

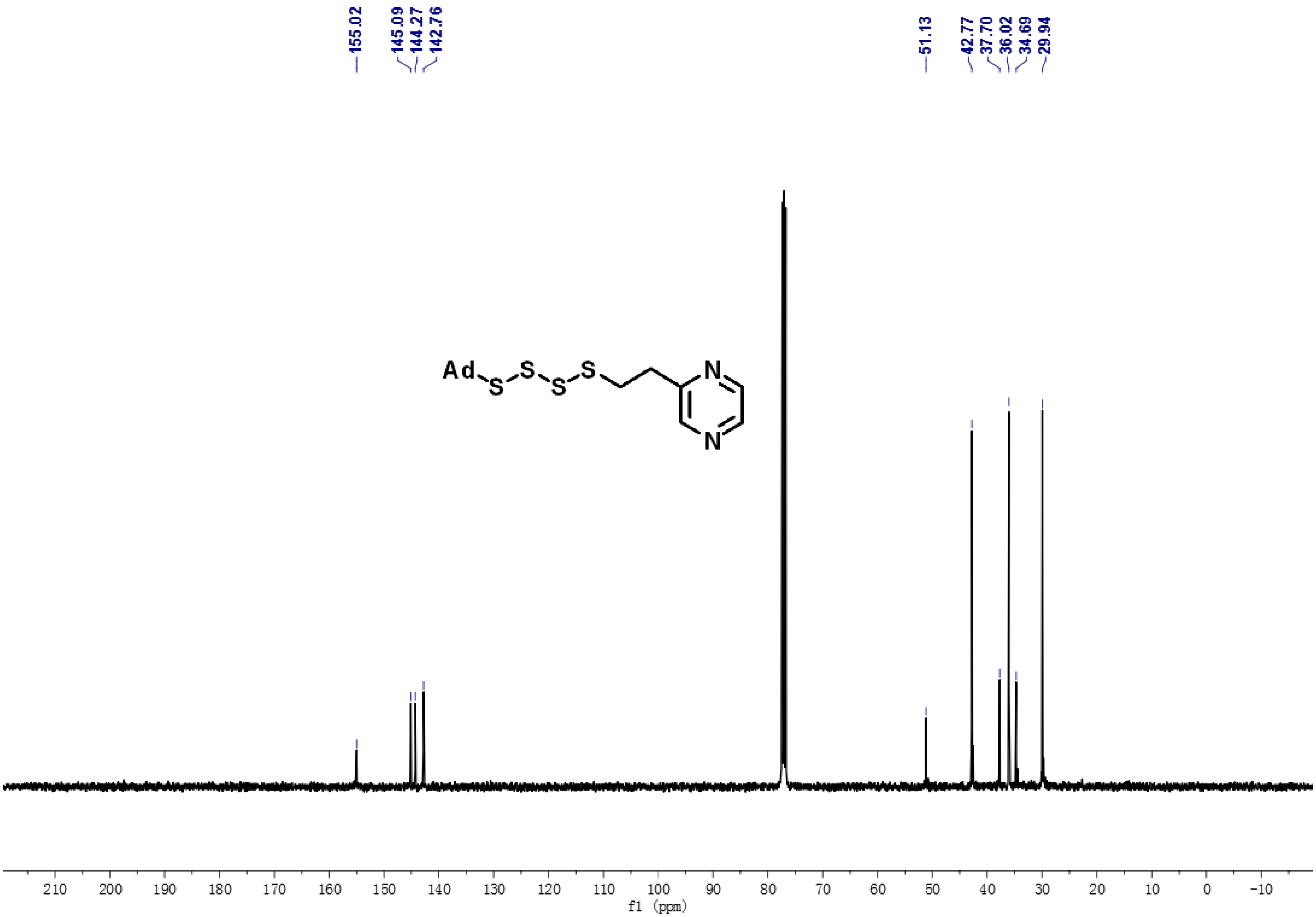

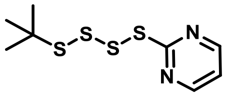

9c

^{1}H NMR (CDCl$_3$)

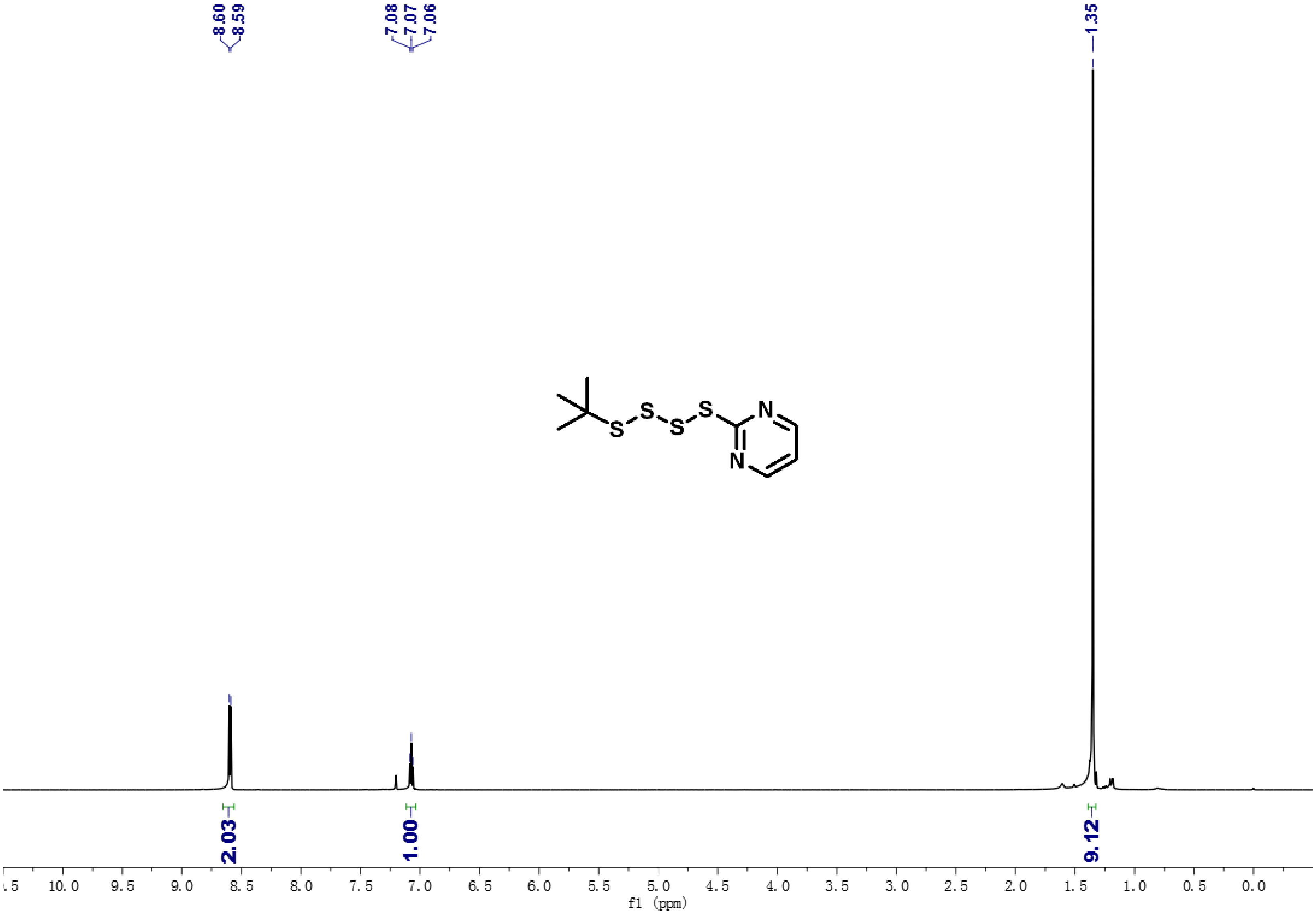

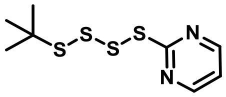

9c

^{13}C NMR ($CDCl_3$)

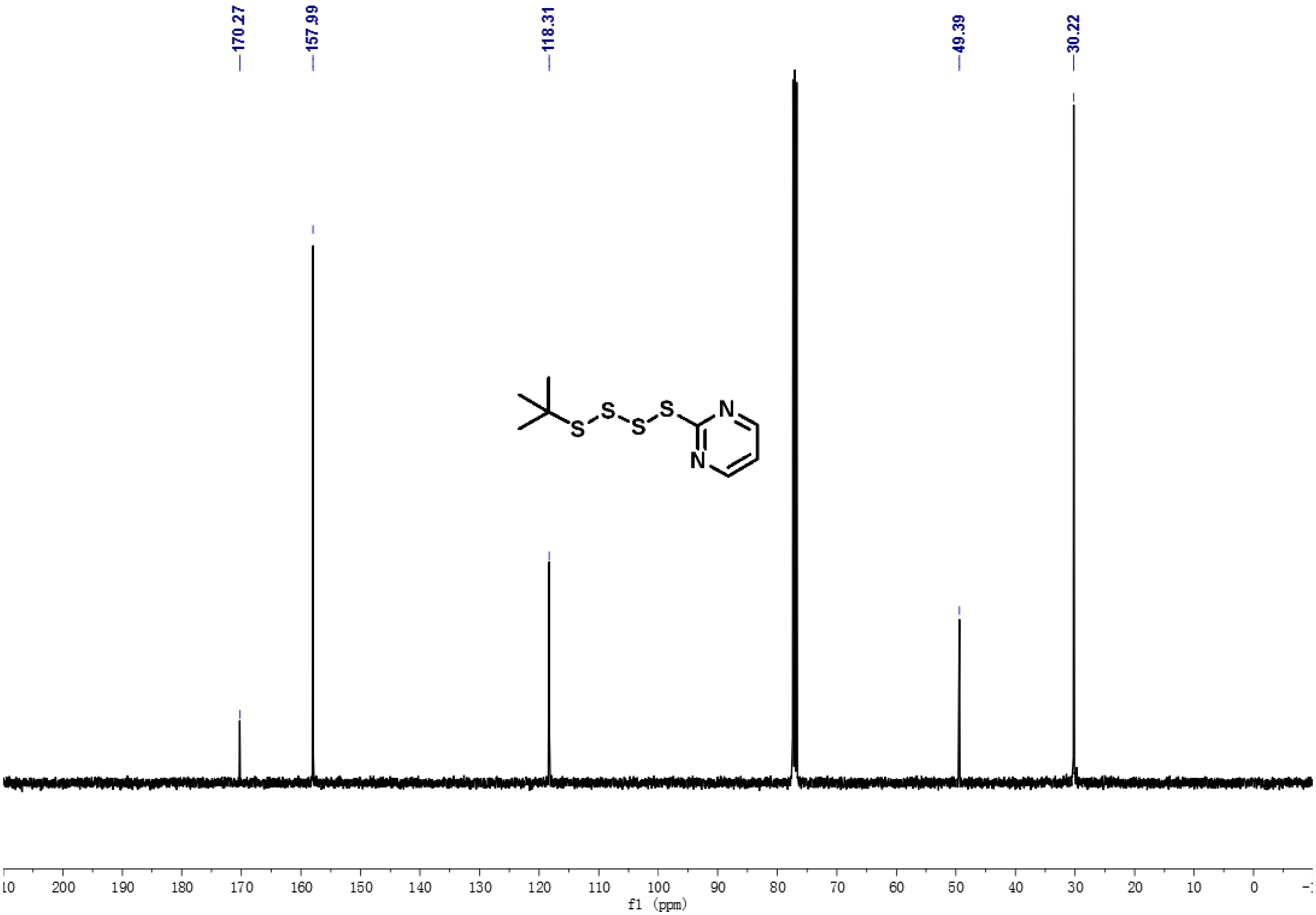

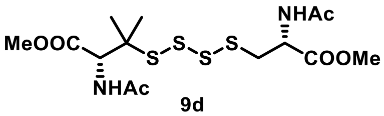

9d

^{1}H NMR ($CDCl_3$)

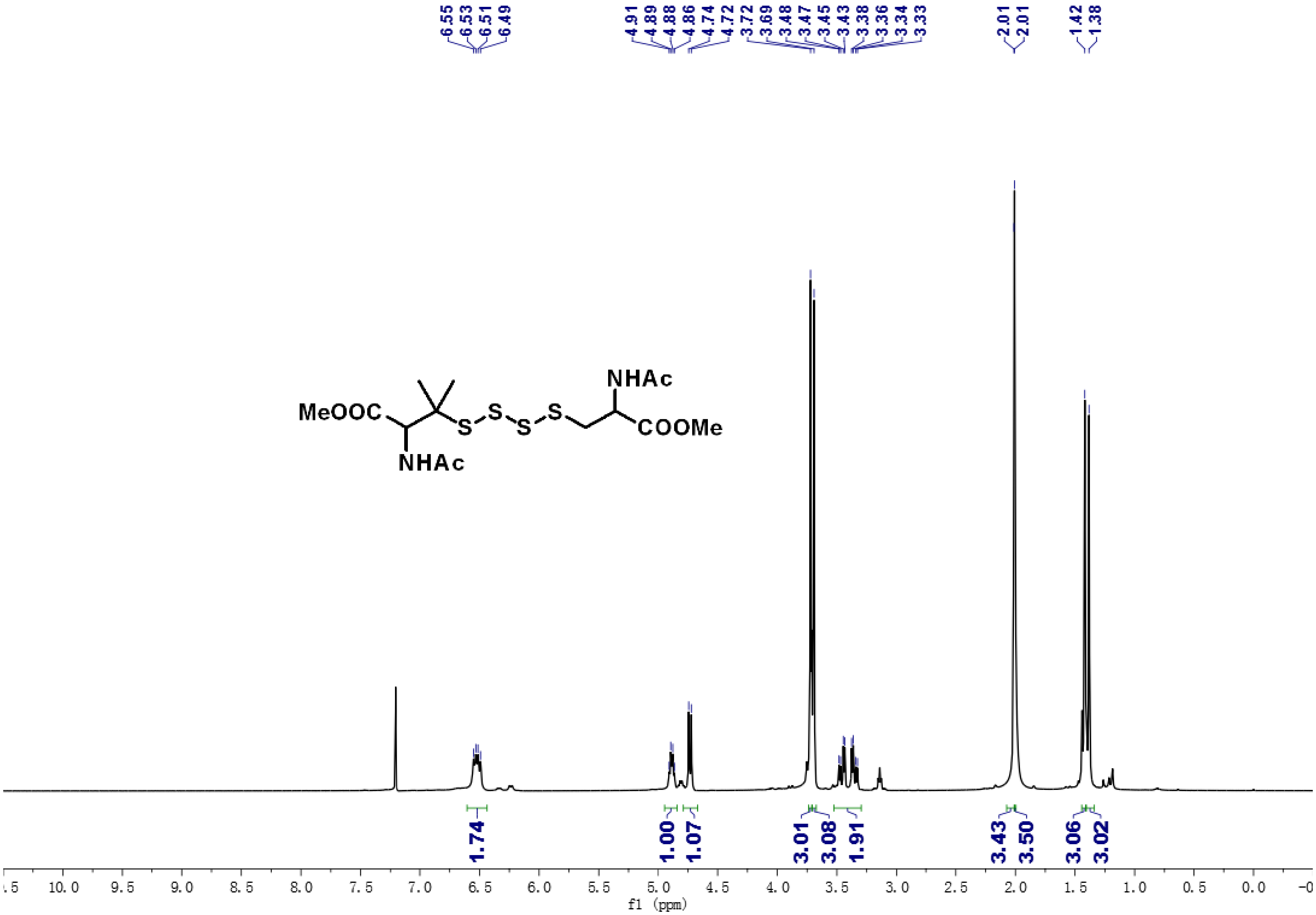

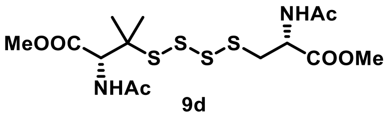

9d

^{13}C NMR ($CDCl_3$)

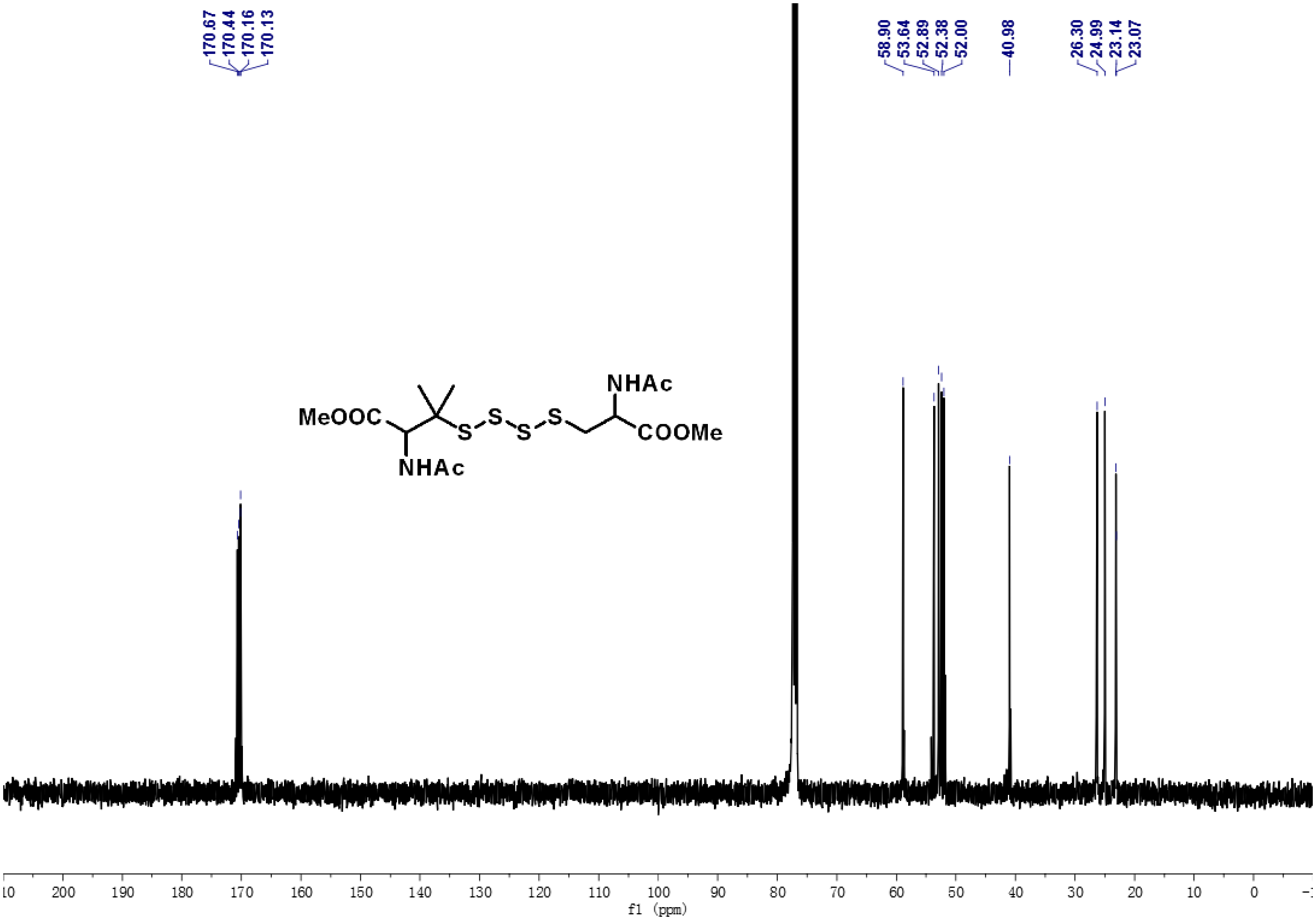

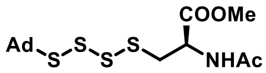

9e

^{1}H NMR ($CDCl_3$)

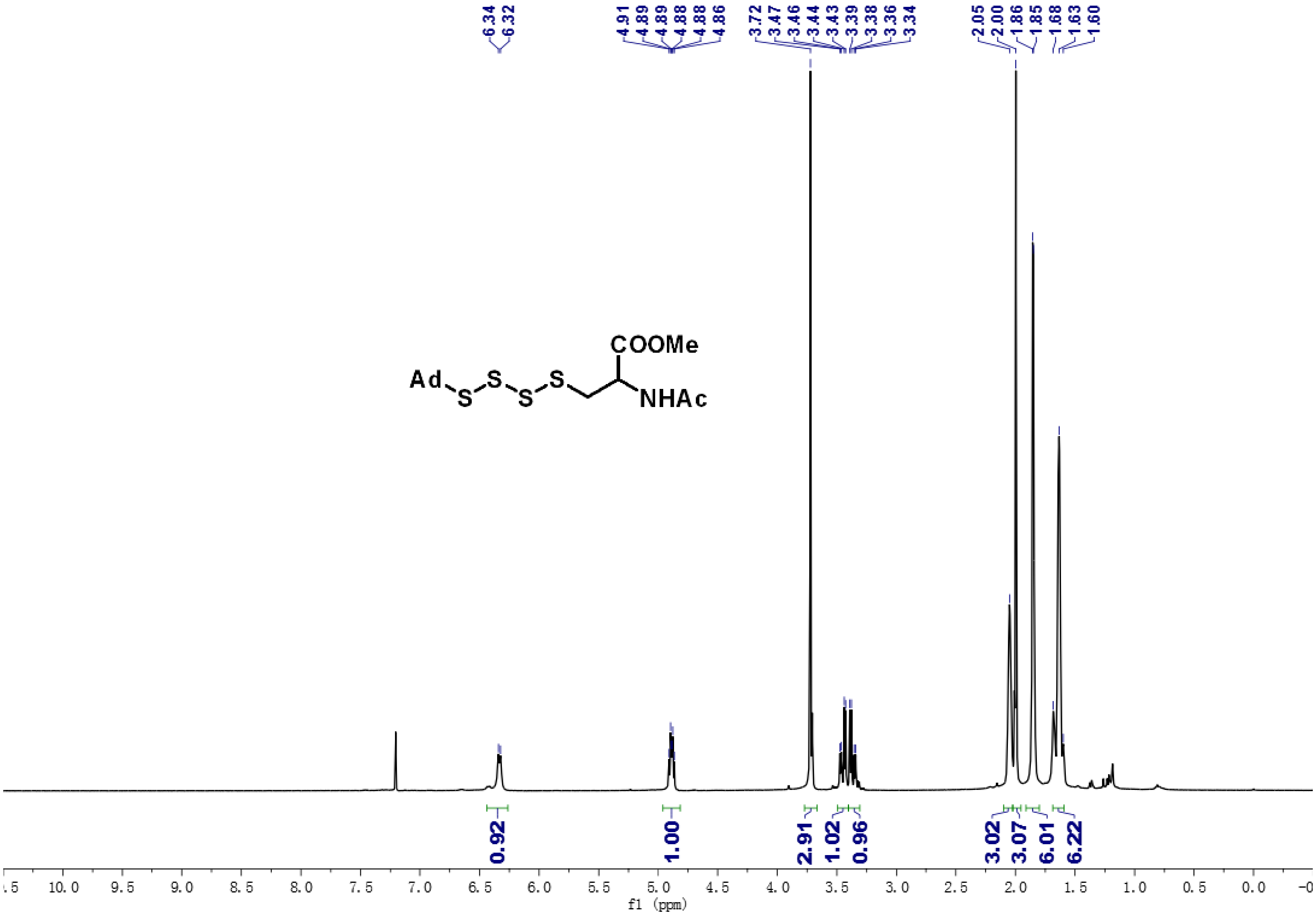

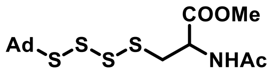

9e

^{13}C NMR ($CDCl_3$)

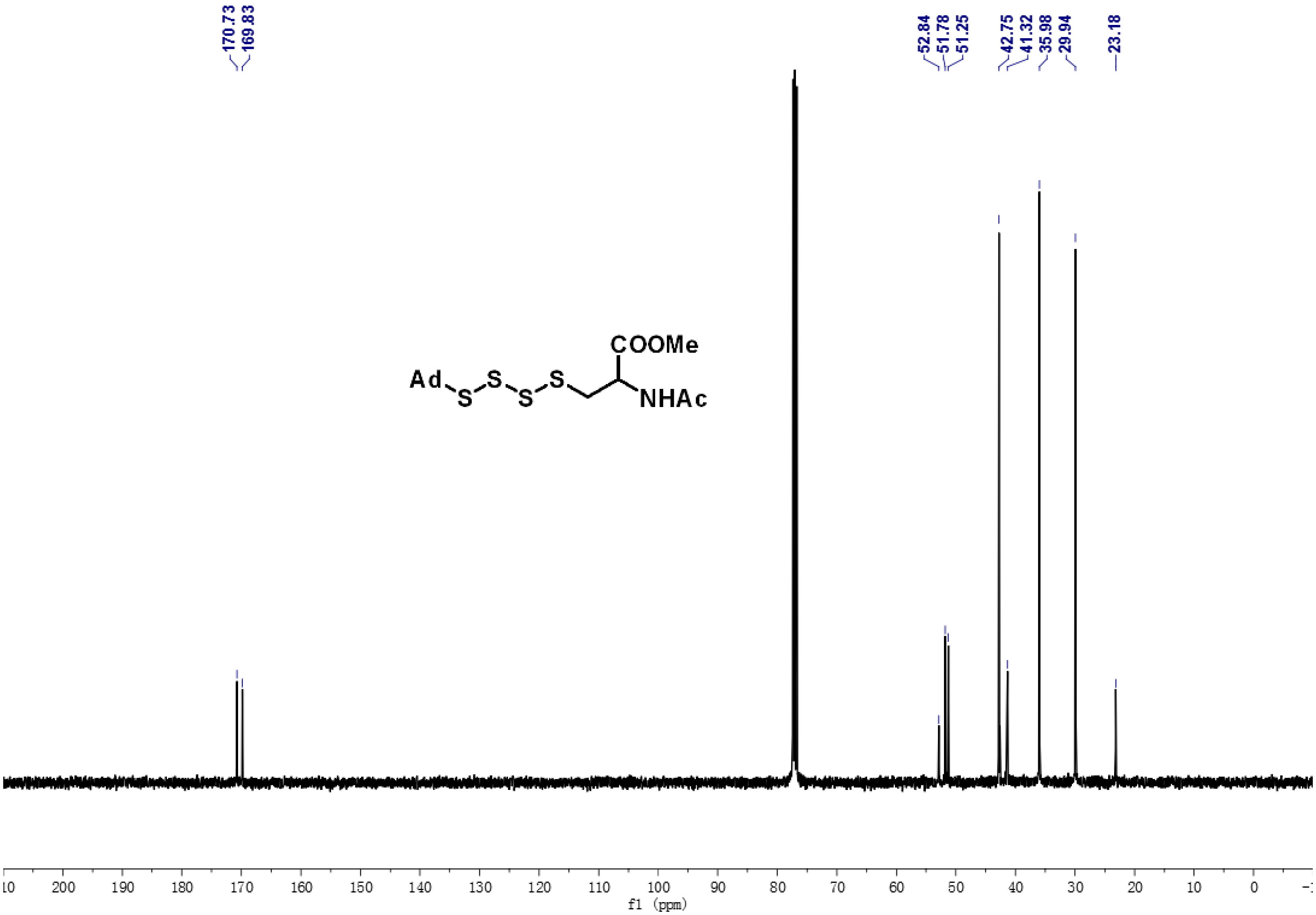

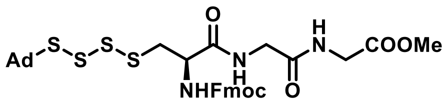

9f

H NMR ($CDCl_3$)

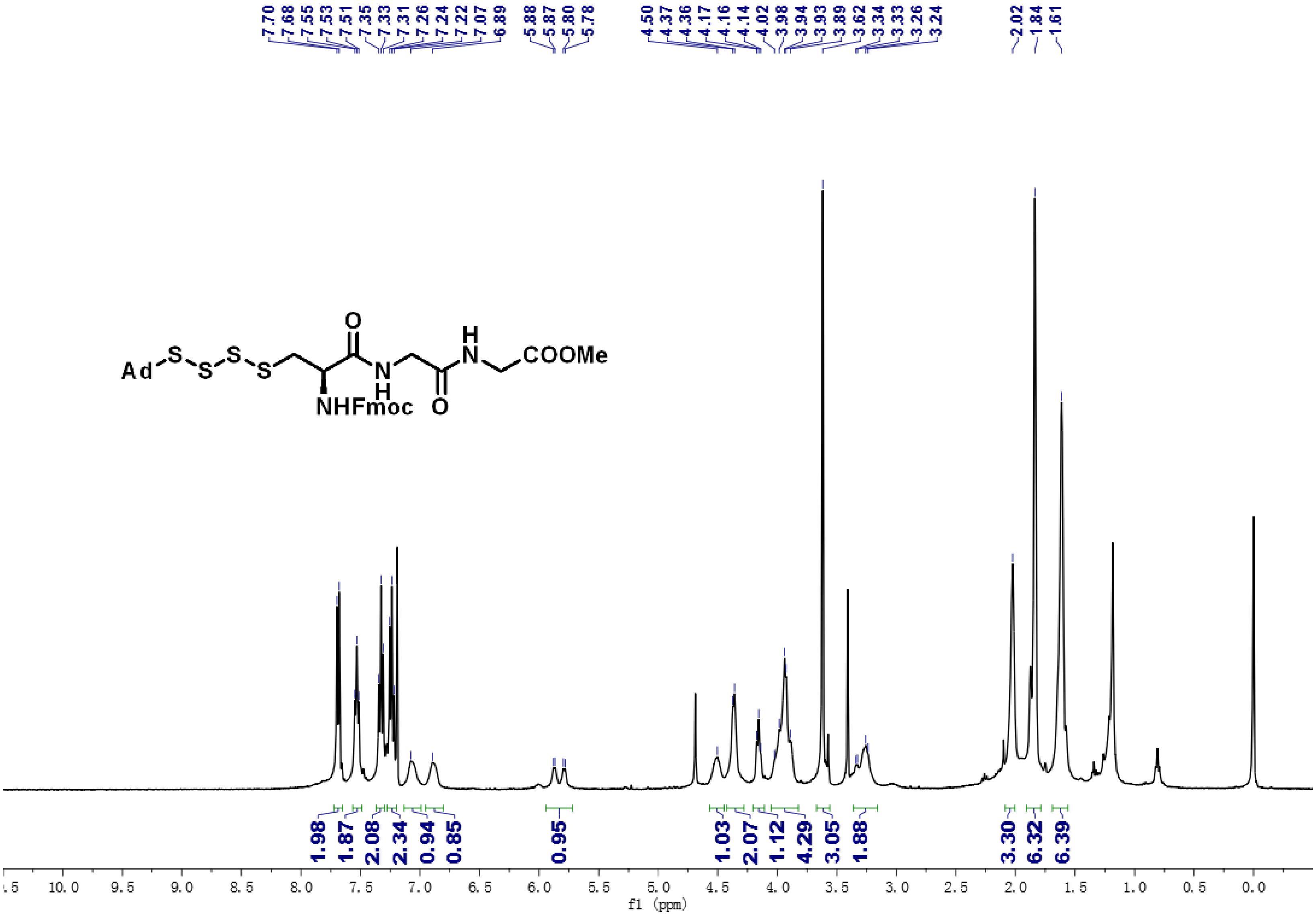

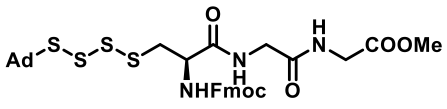

9f

^{13}C NMR ($CDCl_3$)

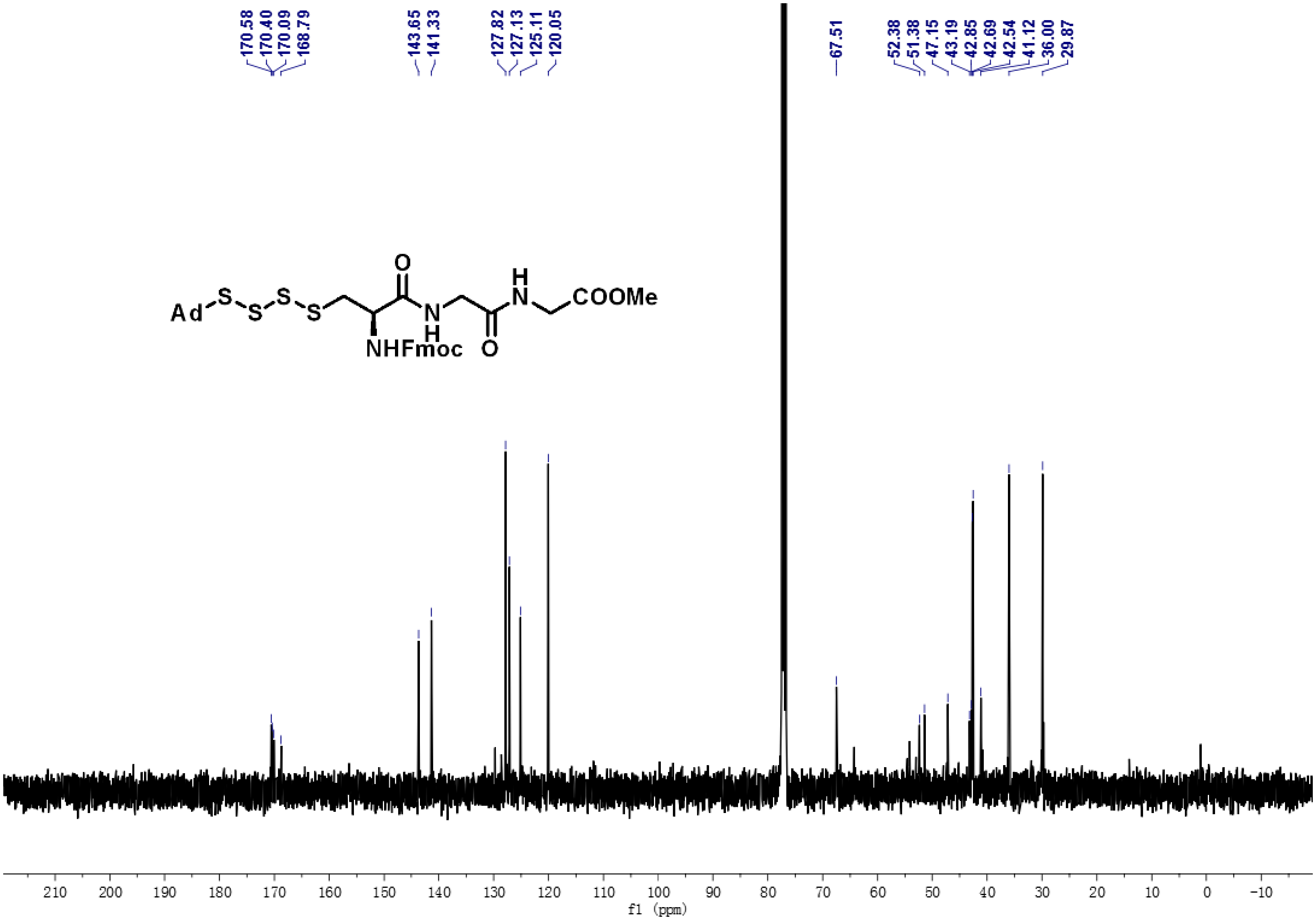

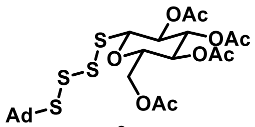

9g

H NMR ($CDCl_3$)

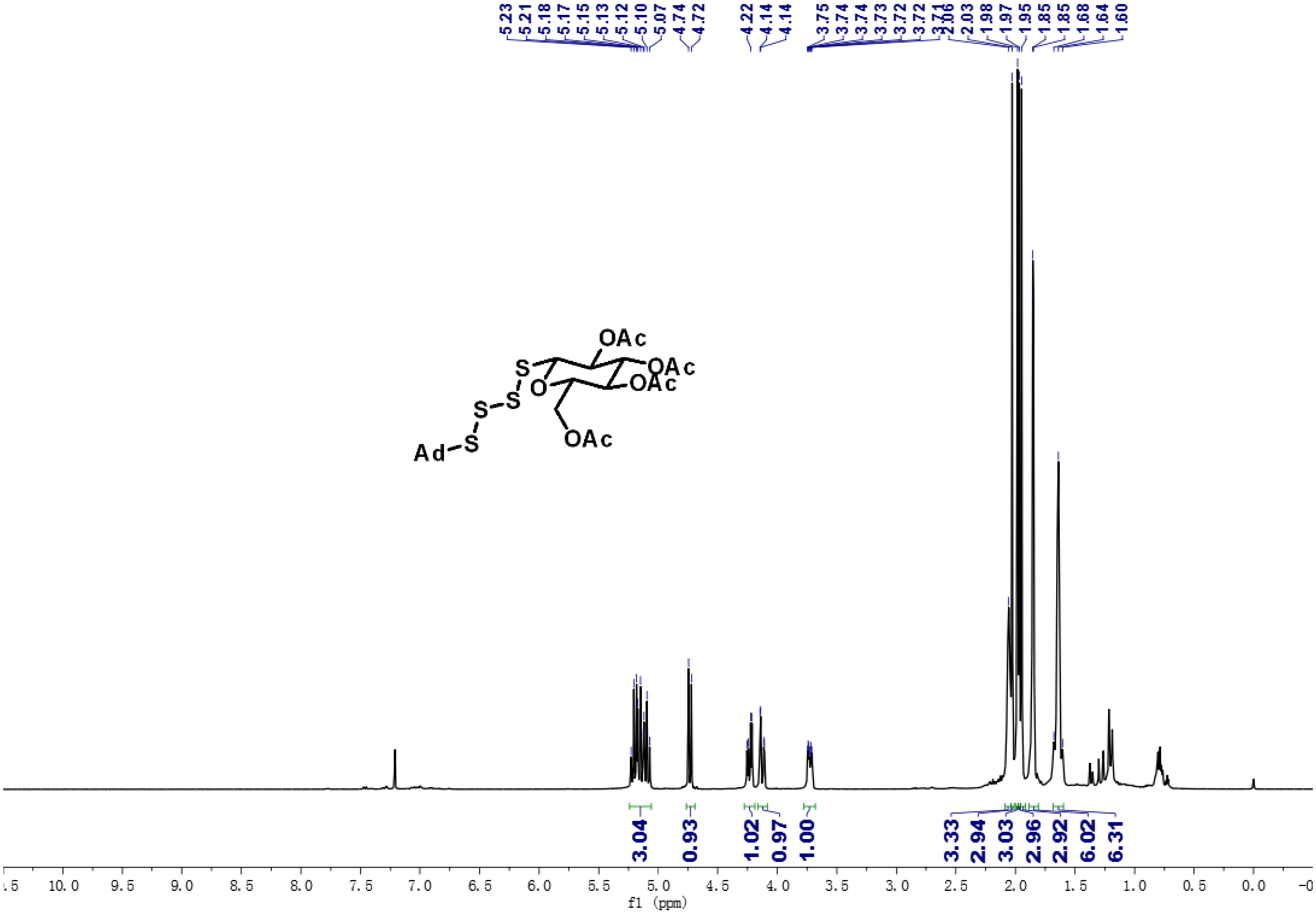

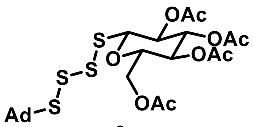

9g

^{13}C NMR ($CDCl_3$)

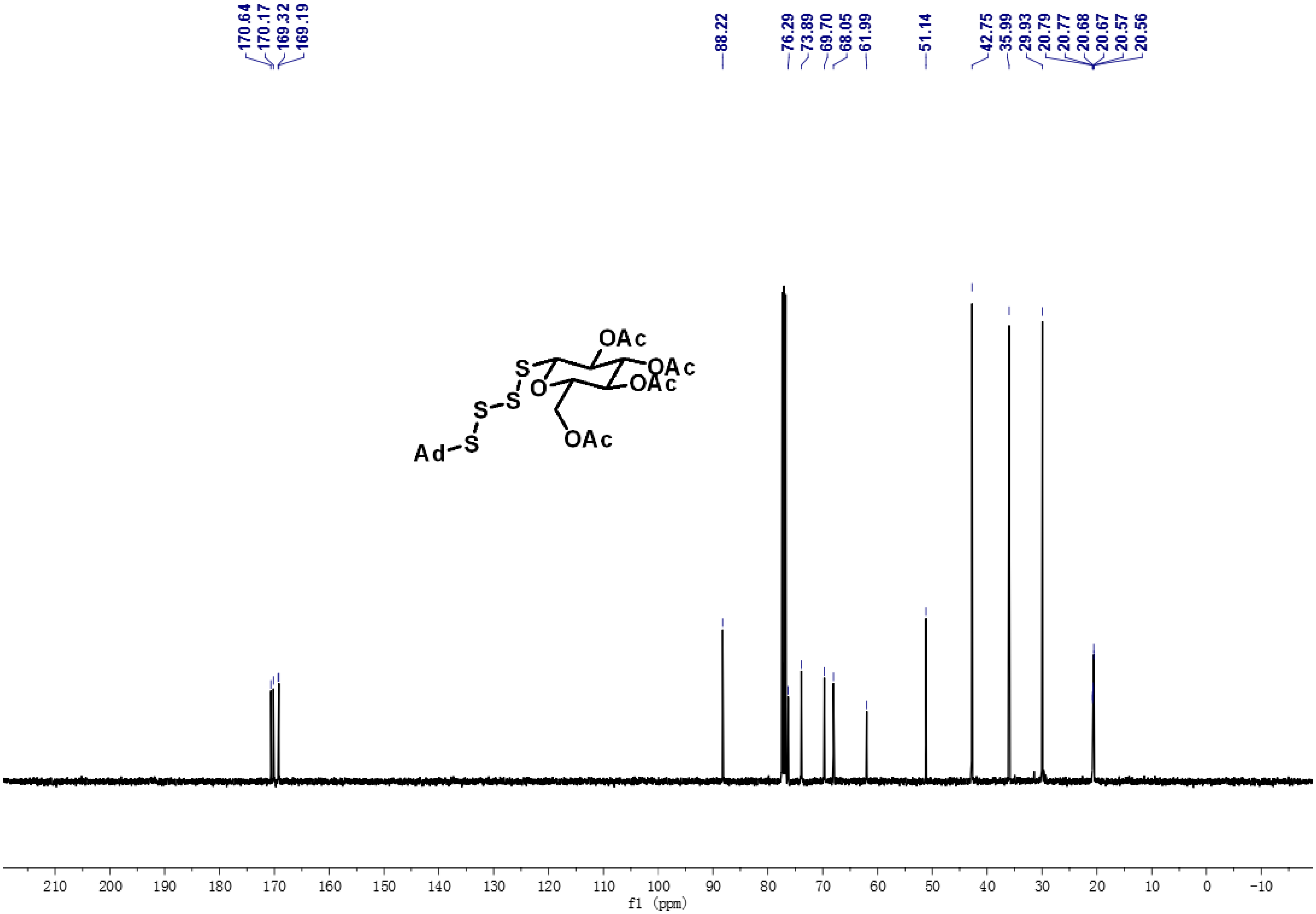

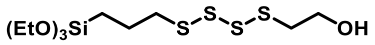

9h

^{1}H NMR ($CDCl_3$)

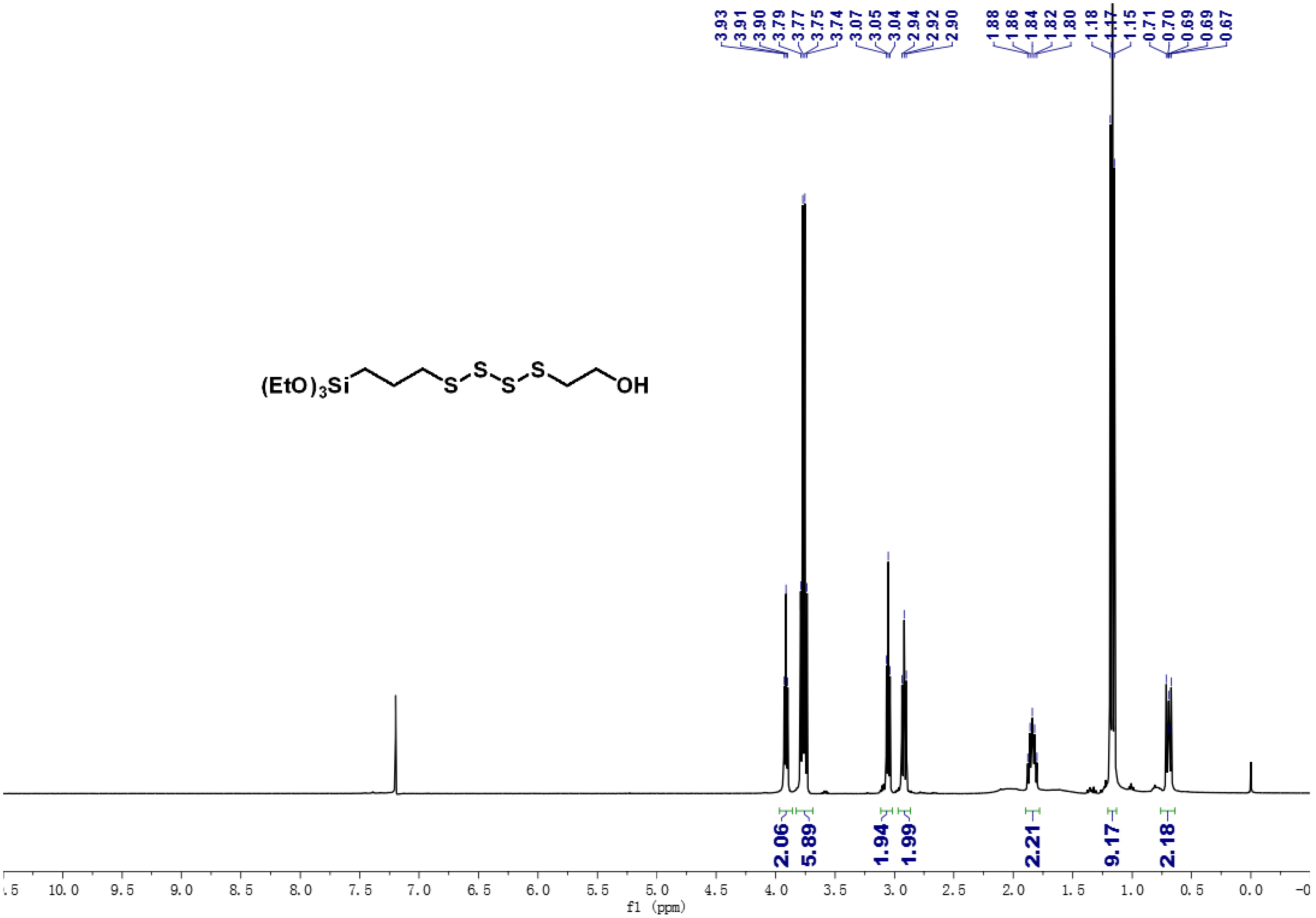

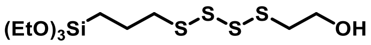

9h

^{13}C NMR ($CDCl_3$)

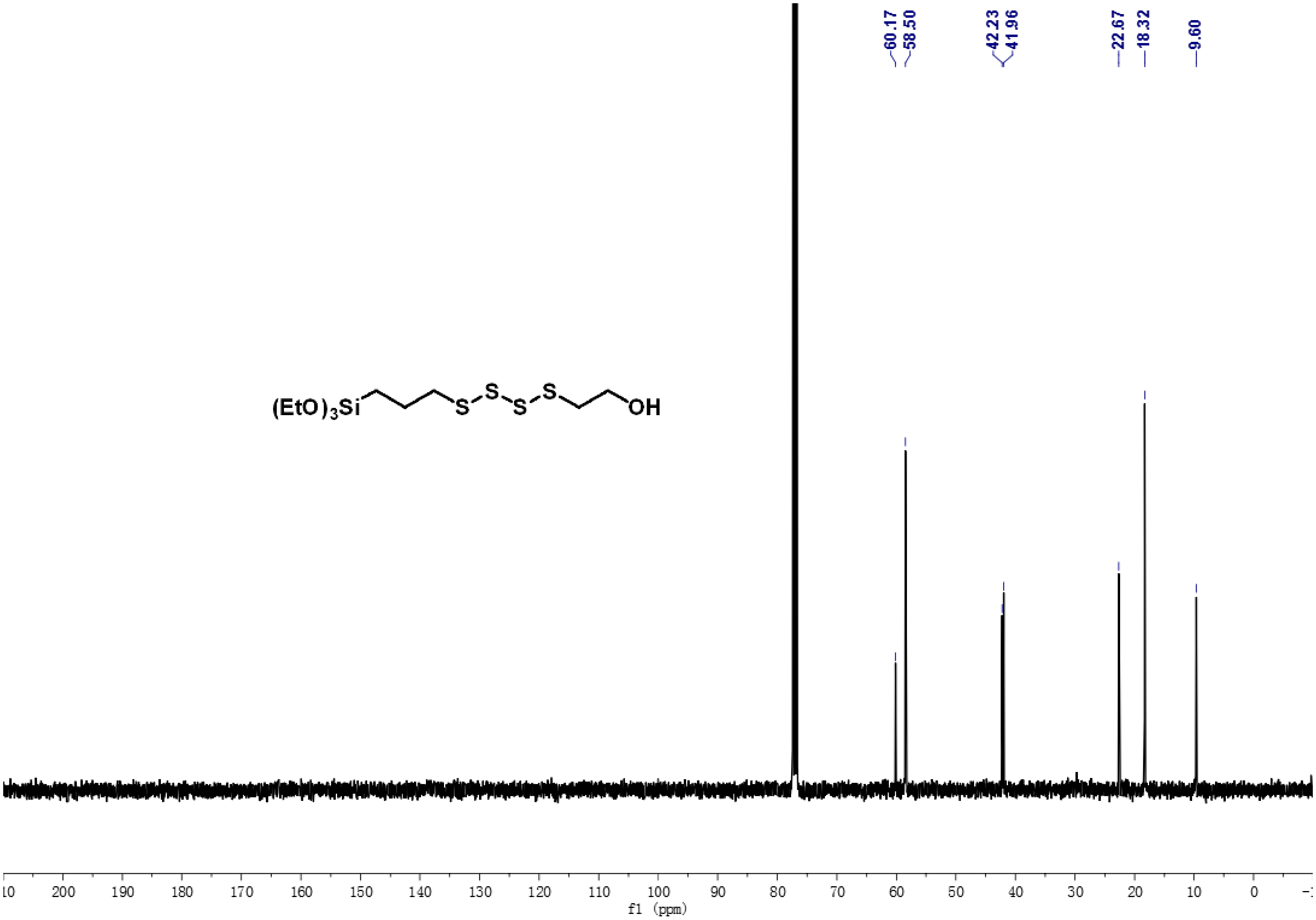

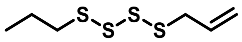

9i

H NMR ($CDCl_3$)

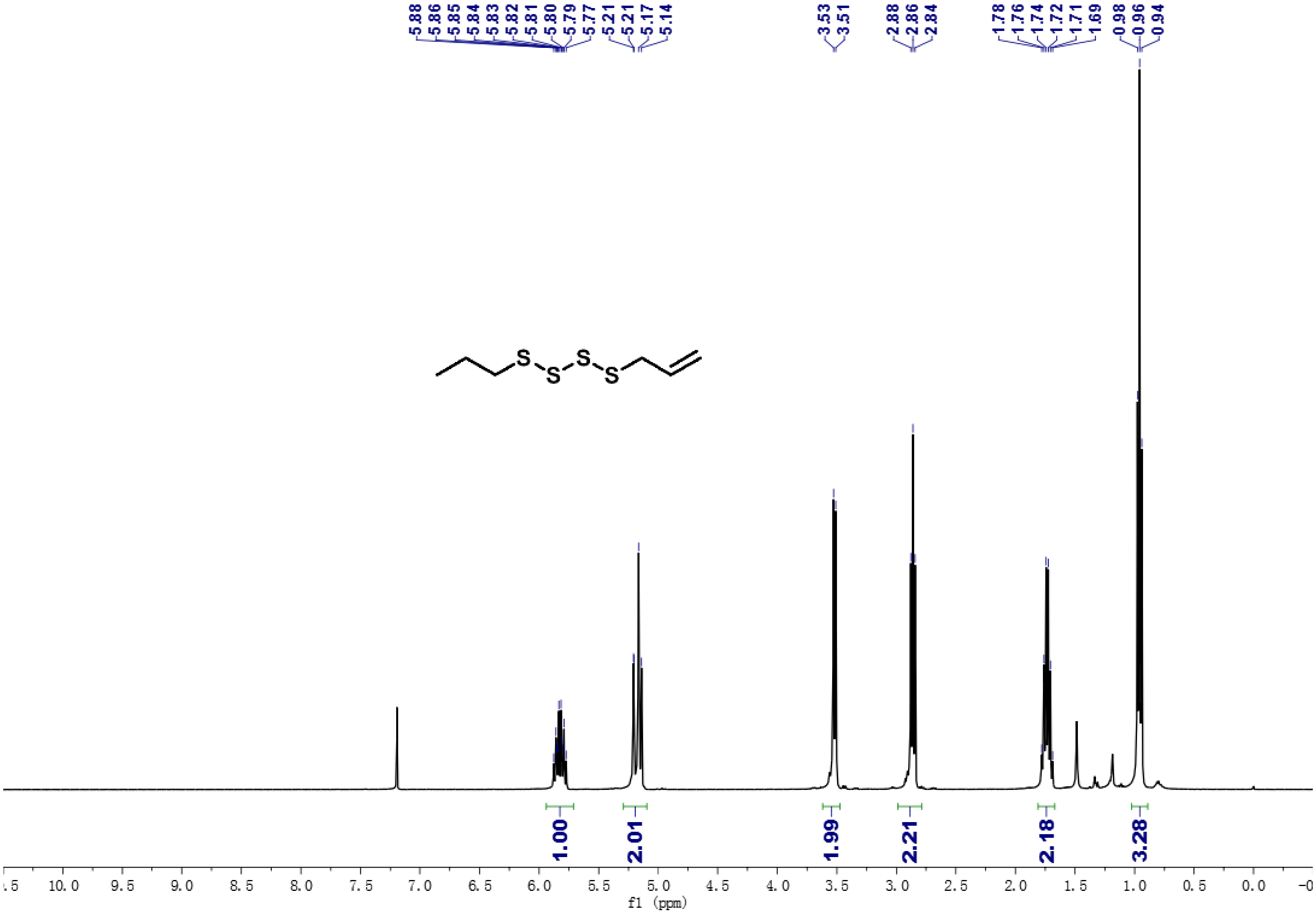

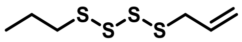

9i

^{13}C NMR ($CDCl_3$)

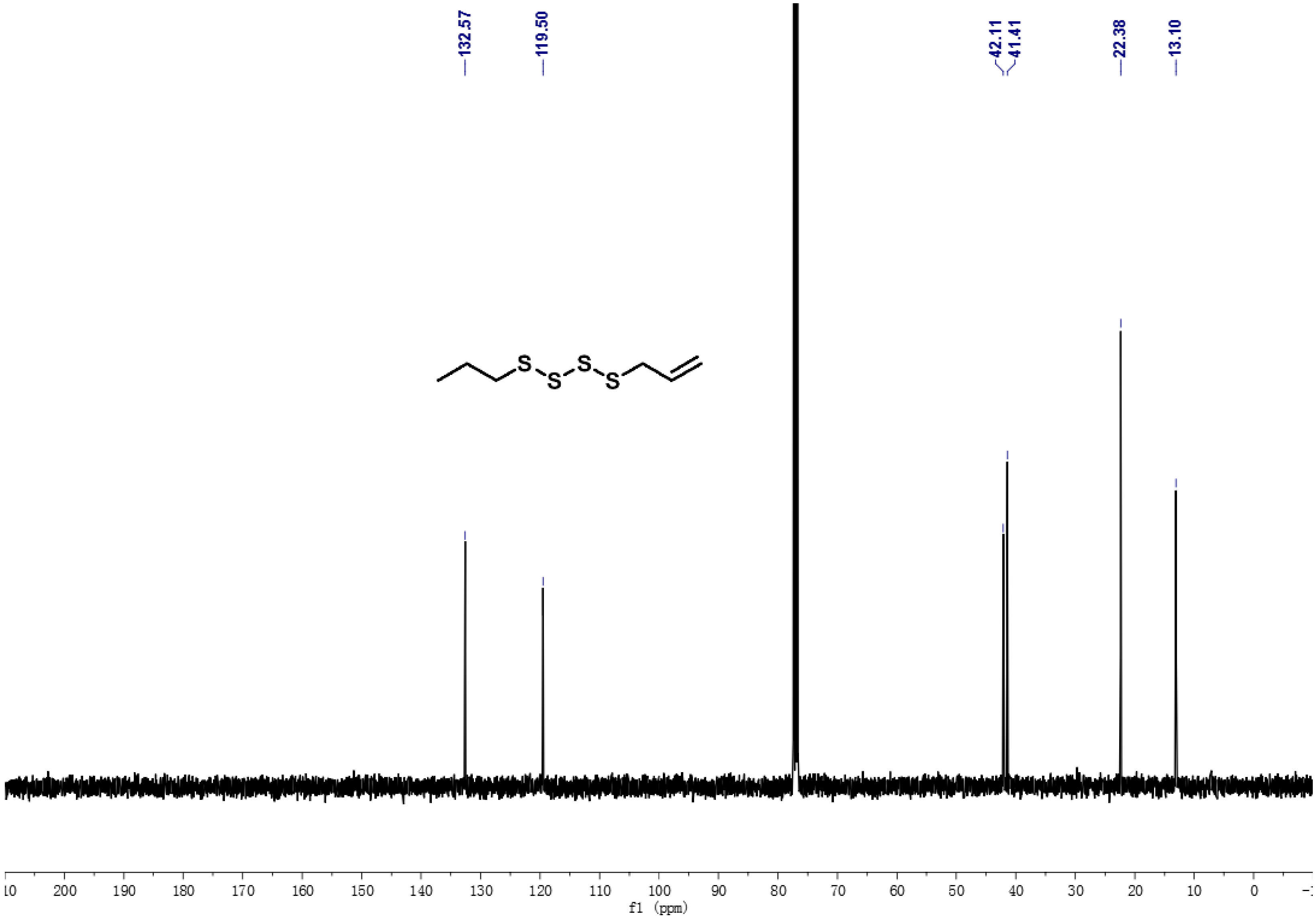

Supplementary Reference

1. Fieser, L. F. & Fieser, M. Reagents for organic synthesis; John Wiley and Sons, Inc.: New York, 1967; Vol. 1; p. 1122.
2. Tardif, S. L., Williams, C. R. & Harpp, D. N. Diatomic sulfur transfer from stable alkoxy disulfides. *J. Am. Chem. Soc.* **117**, 9067–9068 (1995).
3. Zhu, D. et al. *N*-Difluoromethylthiophthalimide: a shelf-stable, electrophilic reagent for difluoromethylthiolation. *Org. Lett.* **137**, 10547–10553 (2015).
4. Zysman-Colman, E. et al. Crossover point between dialkoxy disulfides (ROSSOR) and thionosulfites ($(RO)_2S{=}S$): prediction, synthesis, and structure. *J. Am. Chem. Soc.* **128**, 291–304 (2006).
5. Czepukojc, B. et al. Tetrasulfanes as selective modulators of the cellular thiolstat. Phosphorus, Sulfur, and Silicon. 188, 446–453 (2013).
